# Catalyst-controlled selective borocarbonylation of benzylidenecyclopropanes: regiodivergent synthesis of γ-vinylboryl ketones and β-cyclopropylboryl ketones†

**DOI:** 10.1039/d2sc00840h

**Published:** 2022-03-21

**Authors:** Fu-Peng Wu, Xiao-Feng Wu

**Affiliations:** Leibniz-Institut für Katalyse e.V. Albert-Einstein-Straße 29a 18059 Rostock Germany xiao-feng.wu@catalysis.de; Dalian National Laboratory for Clean Energy, Dalian Institute of Chemical Physics, Chinese Academy of Sciences 116023 Dalian Liaoning China xwu2020@dicp.ac.cn

## Abstract

Regioselective catalytic multi-functionalization reactions enable the rapid synthesis of complexed products from the same precursors. In this communication, we present a method for the regiodivergent borocarbonylation of benzylidenecyclopropanes with aryl iodides. Various γ-vinylboryl ketones and β-cyclopropylboryl ketones were produced in moderate to good yields with excellent regioselectivity from the same substrates. The choice of the catalyst is key for the regioselectivity control: γ-vinylboryl ketones were produced selectively with IPrCuCl and Pd(dppp)Cl_2_ as the catalytic system, while the corresponding β-cyclopropylboryl ketones were obtained in high regioselectivity with Cu(dppp)Cl, [Pd(*η*^3^-cinnamyl)Cl]_2_ and xantphos as the catalytic system. Moreover, γ-vinylboryl ketones and β-cyclopropylboryl ketones were successfully transformed into several other value-added products.

## Abstract 2

A novel procedure for regiodivergent borocarbonylation of benzylidenecyclopropanes has been developed. A variety of valuable γ-vinylboryl ketones and β-cyclopropylboryl ketones can be obtained selectively in excellent yields.

## Graphical abstract

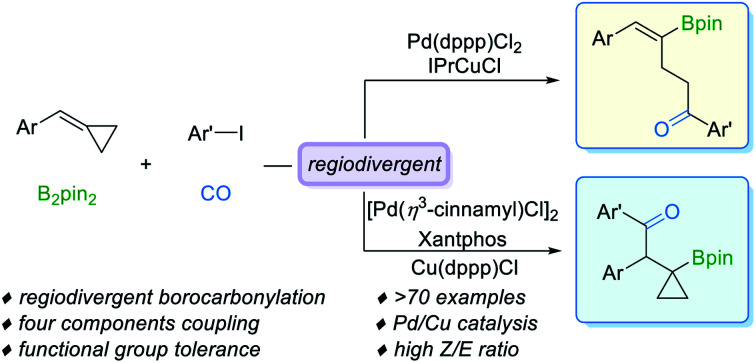

## Introduction

**Fig. 1:**
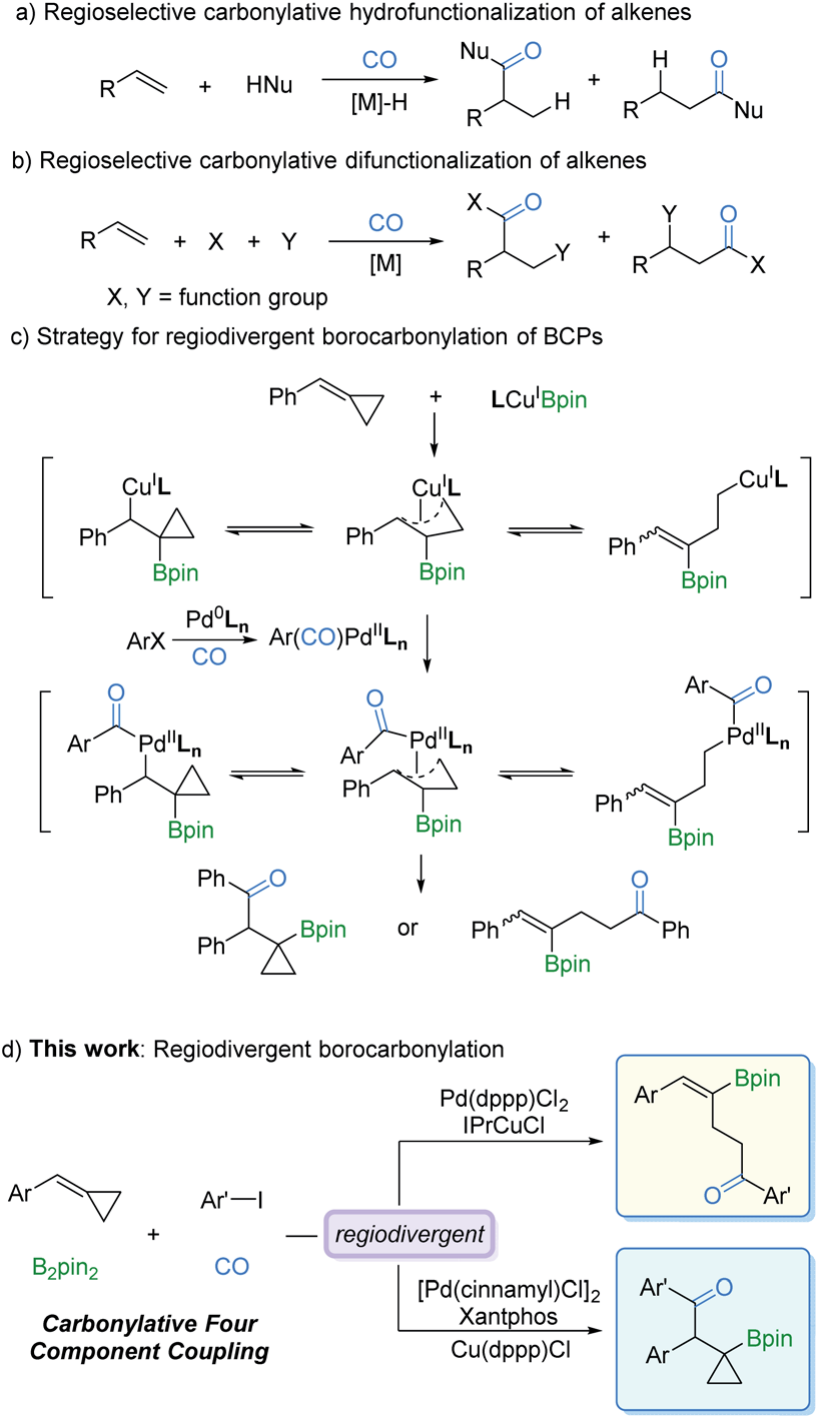
Strategies for regiodivergent borocarbonylation of BCPs.

Transition metal-catalyzed regioselective reaction of alkenes is of utmost importance for the synthesis of diverse organic products.^1,5^ One of the main advantages of this protocol is that by controlling the regioselectivity and molecular complexity, different regioisomers can be rapidly produced from simple precursors. In the last few decades, studies on metal catalysts and new ligands have provided more opportunities for regioselective reactions.^2^ Among the known transformations, carbonylation as one of the most effective synthetic tools for carbon chain prolongation by CO introduction has attracted extensive attention, especially its regioselective versions. As we expected, many novel regioselective carbonylations of alkenes have been reported. However, most of the developed procedures were focused on carbonylative hydrofunctionalization of alkenes (Fig. 1a).^3^ In contrast, carbonylative difunctionalization of alkenes remains a challenge, especially in controlling the regioselectivity (Fig. 1b).^4^ There are two possible reasons for this challenge: (i) CO coordinates with the metal catalyst and reduces its electron density which is essential for substrate activation; (ii) multiple reactivities can be evoked on the double bond with other reaction partners.^5^

The possibility of simultaneously generating C–B and C–C(O) bonds in a regioselective manner through insertion across the C=C bond is a sought-after goal in catalytic olefin borocarbonylation. The resulting organoboranes are useful synthons that increase functionality and complexity *via* oxidation, the Suzuki–Miyaura coupling reaction, vinylation, *etc.*^6^ To date, borocarbonylation reactions have been reported with alkenes, alkynes, and imines.^7^ The reported borocarbonylation of alkenes was limited to styrenes and did not allow for regioselectivity control.^7*b*^ Thus, variants involving methylenecyclopropanes^8^ are particularly attractive because of the potential to control the formation of various boryl ketones. As depicted in Fig. 1c, L*_n_*Cu^I^Bpin^9^ inserts into benzylidenecyclopropanes (BCPs) to form isomeric π-copper complexes. Subsequently, transmetallation between π-copper complexes and acyl-palladium species generates π-acyl-palladium species, which leads to the possibility of multiple isomers. Theoretically, it is possible to control the regioselectivity by adjusting the catalyst systems. Thus, the development of a new borocarbonylation process with BCPs that can selectively incorporate multiple compounds into one pot is highly desired. In this communication, we describe a process for the regiodivergent borocarbonylation of a variety of substituted BCPs by Cu/Pd catalytic systems to produce γ-vinylboryl ketones and β-cyclopropylboryl ketones (Fig. 1d).

## Results and discussion

**Fig. 2:**
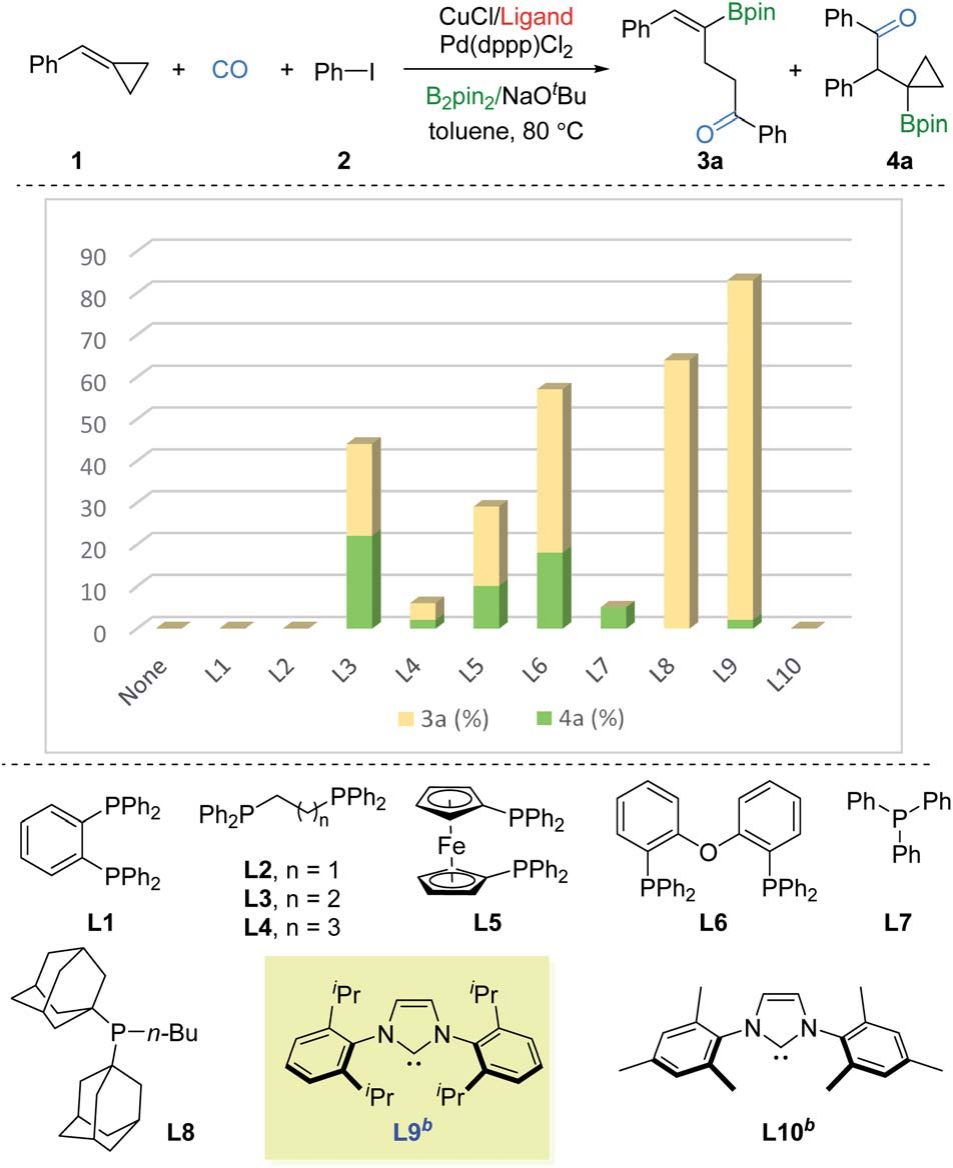
Optimization for product 3a. Reaction conditions: 1 (0.2 mmol), 2 (1.5 equiv., 0.3 mmol), CuCl (10 mol%), ligand (10 or 20 mmol%), Pd(dppp)Cl_2_ (2 mol%), B_2_pin_2_ (1.5 equiv., 0.3 mmol), NaO^*t*^Bu (1.5 equiv., 0.3 mmol), toluene (0.2 M), CO (10 bar), stirred at 80 °C for 20 h. Yields and ratios (3a : 4a) were determined by GC analysis using hexadecane as the internal standard. ^*b*^NHC-CuCl complex was used instead of CuCl.

We commenced our studies with BCP 1, iodobenzene 2, and B_2_pin_2_ as the model substates. Ancillary ligands of copper and palladium were thought to be the crucial factor for the borocarbonylation, thus we first screened ligands for copper with the use of Pd(dppp)Cl_2_. As shown in Fig. 2, no desired products were detected in the absence of ligand. Using DPPBz (L1) or DPPE (L2) as the ligand also failed to produce the γ-vinylboryl ketone 3a or β-cyclopropylboryl ketone 3b products. In contrast, when using DPPP (L3) as the ligand, we were able to obtain a total 44% yield of 3a and 3b but with poor selectivity. Then various mono or bisphosphine ligands (L4–L8) with a range of steric and electronic properties were screened, and the sterically bulky and electron-donating BuPAd_2_ (L8) was found to be able to deliver the desired 3a in 64% yield with high selectivity. Based on these primary results, we switched to testing strong electron-donating NHC ligands. Impressively, only γ-vinylboryl ketone 3a (81% yield, 3a : 4a > 20 : 1 selectivity) was obtained by using IPr ligand while no desired products were observed by employing IMes ligand. After fine-tuning the loading of 2, the yield of 3a was improved to 85% (see the ESI†). These results imply that the ligand with strong electron-donating and sterically bulky properties are essential for driving the tendency of the β-cyclopropylboryl alkyl-copper intermediate toward the γ-vinylboryl-alkyl-copper complex.

**Fig. 3:**
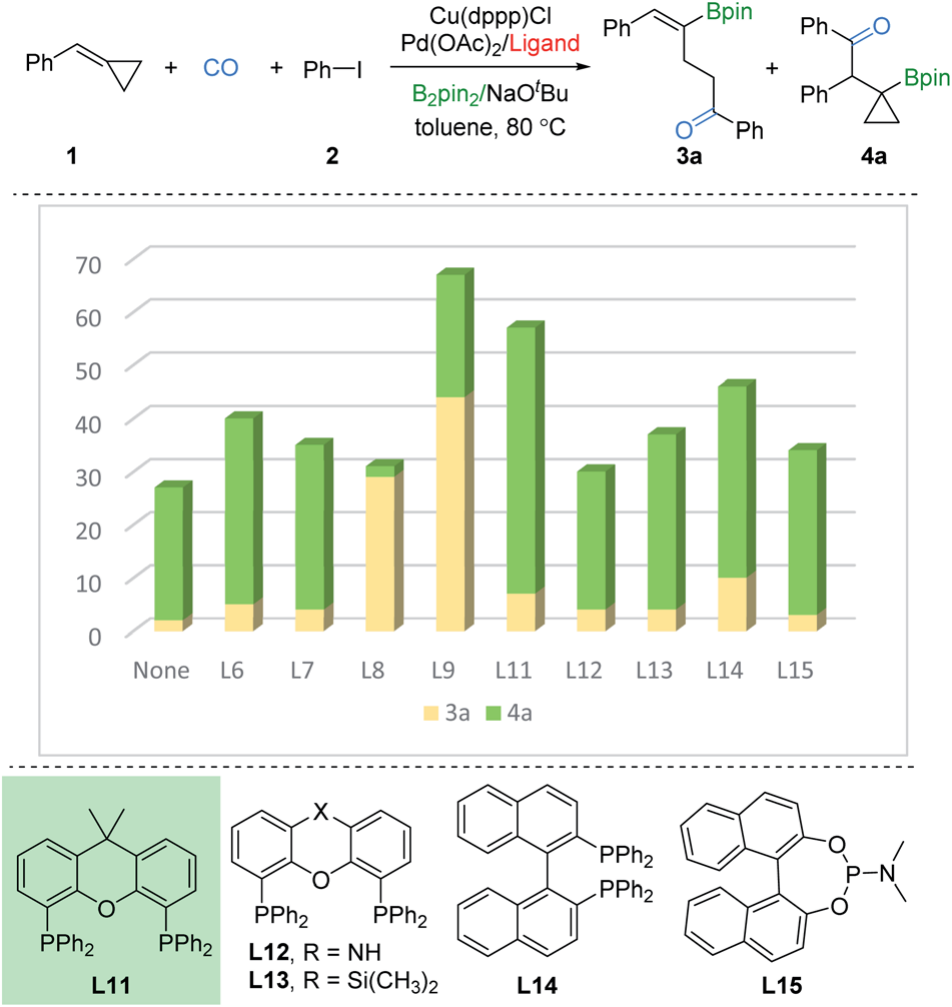
Optimization for product 4a. Reaction conditions: 1 (0.2 mmol), 2 (1.5 equiv., 0.3 mmol), Cu(dppp)Cl (10 mol%), Pd(OAc)_2_ (2 mol%), ligand (2 or 4 mmol%), B_2_pin_2_ (1.5 equiv., 0.3 mmol), NaO^*t*^Bu (1.5 equiv., 0.3 mmol), toluene (0.2 M), CO (10 bar), stirred at 80 °C for 20 h. Yields and ratios (3a : 4a) were determined by GC analysis using hexadecane as the internal standard.

In order to investigate the regioselective borocarbonylation more intensively, Cu(dppp)Cl and Pd(OAc)_2_ were chosen as the catalysts to further optimize the selectivity to obtain β-cyclopropylboryl ketone 4a (Fig. 3). In the absence of ligand, 4a was obtained in 31% yield with moderate selectivity. In addition, the use of L8 or L9, which are more susceptible to generation of 3a, delivered 4a in low yields. With xantphos (L11) as the ligand, the reaction smoothly proceeds to the target product 4a in good conversion with moderate selectivity (50% yield of 4a, 3a : 4a = 1 : 7). Other tested ligands, including xantphos-type (L12 and L13), DPEphos (L6), BINAP (L14), PPh_3_ (L7), and phosphoramidite (L15) were all less effective. After screening the palladium sources, the desired product 4a was afforded in 61% yield (see the ESI†).

**Fig. 4:**
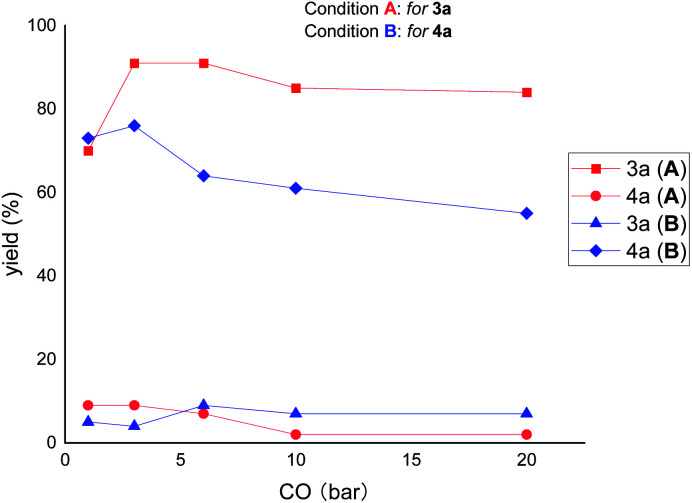
The effect of CO pressure. The *X*-axis represents carbon monoxide pressure, and the *Y*-axis represents yield. Condition A: 1 (0.2 mmol), 2 (1.7 equiv.), IPrCuCl (10 mol%), Pd(dppp)Cl_2_ (2 mol%), B_2_pin_2_ (1.5 equiv.), NaO^*t*^Bu (1.5 equiv.), toluene (0.2 M), stirred at 80 °C for 20 h; condition B: 1 (0.2 mmol), 2 (1.5 equiv.), Cu(dppp)Cl (10 mol%), [Pd(*η*^3^-cinnamyl)Cl]_2_ (1 mol%), xantphos (2 mmol%), B_2_pin_2_ (1.5 equiv.), NaO^*t*^Bu (1.5 equiv.), toluene (0.2 M), stirred at 80 °C for 20 h. The yields were determined by GC analysis.

Next, we were interested to find out the effect of CO pressure on the reactivity and selectivity. As shown in Fig. 4, increasing the pressure of CO (from 3 to 20 bar) decreased the reactivity but increased the selectivity of the reaction under the ring-opening conditions (for 3a). In contrast, under the ring-remaining conditions (for 4a), increasing the CO pressure simultaneously decreased the reactivity and selectivity of the reaction. These results suggest that high CO pressure has a significant deleterious effect on the reactivity of ring-remaining than ring-opening conditions.

**Fig. 5:**
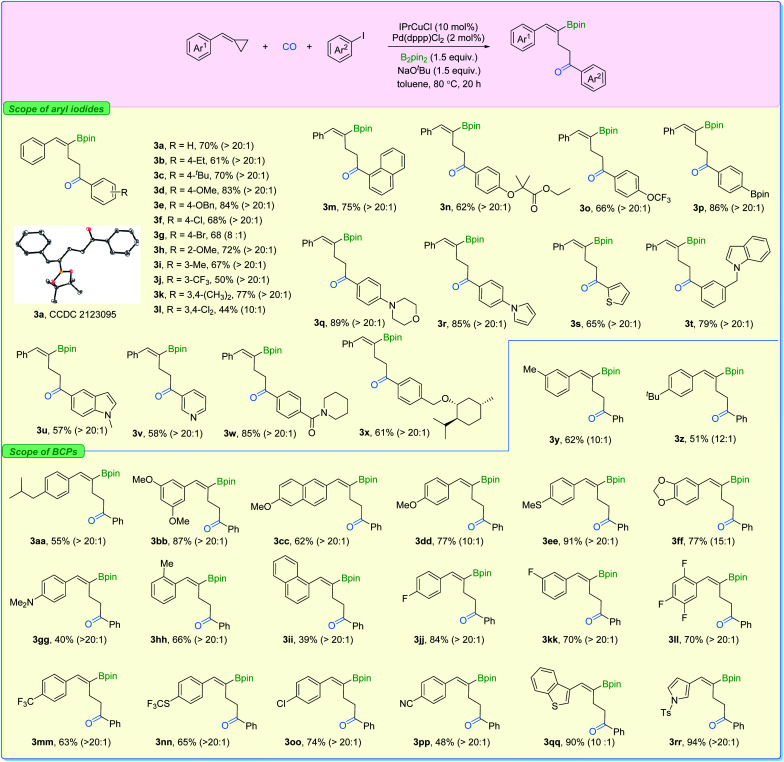
Substrate scope for product 3. Reaction conditions: BCP (0.2 mmol), aryl iodides (1.7 equiv.), IPrCuCl (10 mol%), Pd(dppp)Cl_2_ (2 mol%), B_2_pin_2_ (1.5 equiv.), NaO^*t*^Bu (1.5 equiv.), toluene (0.2 M), CO (10 bar), stirred at 80 °C for 20 h, isolated yield; *Z*/*E* > 20 : 1 was observed in all cases; r.r. (3 : 4) and *Z*/*E* values were measured in crude mixtures by NMR and gas chromatography analysis. Displacement ellipsoid plot (30% probability level, without H).

With the two sets of optimized reaction conditions in hand, we firstly explored the feasibility of substrates on aryl iodides for ring-opening product formation (Fig. 5). With IPrCuCl and Pd(dppp)Cl_2_ as the supporting catalysts, in most of the cases, we observed the γ-vinylboryl ketones with selectivity greater than 20 : 1. The absolute configuration of compound 3a was clearly confirmed by X-ray crystallography. Aryl iodides bearing electron-donating groups at the *para*- (3b–3e), *ortho*- (3h), and *meta*- (3i) position were all tolerated, affording the corresponding products with high activity and high levels of regioselectivity. Electron-withdrawing groups such as Cl and CF_3_ (3f and 3j) on the aryl iodides were suitable as well, while two examples showed moderate levels of selectivity (3g and 3l). In addition, functional groups including ester (3n), Bpin (3p), morpholine (3q), pyrrole (3r), indole (3t), amide (3w), and the highly lipophilic OCF_3_ (3o) group were all compatible with the reaction conditions, producing the desired products in moderate to good yields.

Additionally, the transformation proved to be tolerant of heterocyclic iodides (3s, 3u, and 3v) and gave the corresponding products in good yields with excellent selectivity. Various substituted-BCPs were also successfully transformed using this protocol (3y–3ii). In particular, phenyl rings containing fluoride groups were also efficiently converted to the desired products in good yields (3jj–3nn). Benzothiophene (3qq) and pyrrole (3rr) were also competent substrates here and gave excellent yields of the corresponding products. It is important to mention that no desired product could be detected when (cyclobutylidenemethyl)benzene or (1-cyclopropylideneethyl)benzene was evaluated under our standard conditions.

**Fig. 6:**
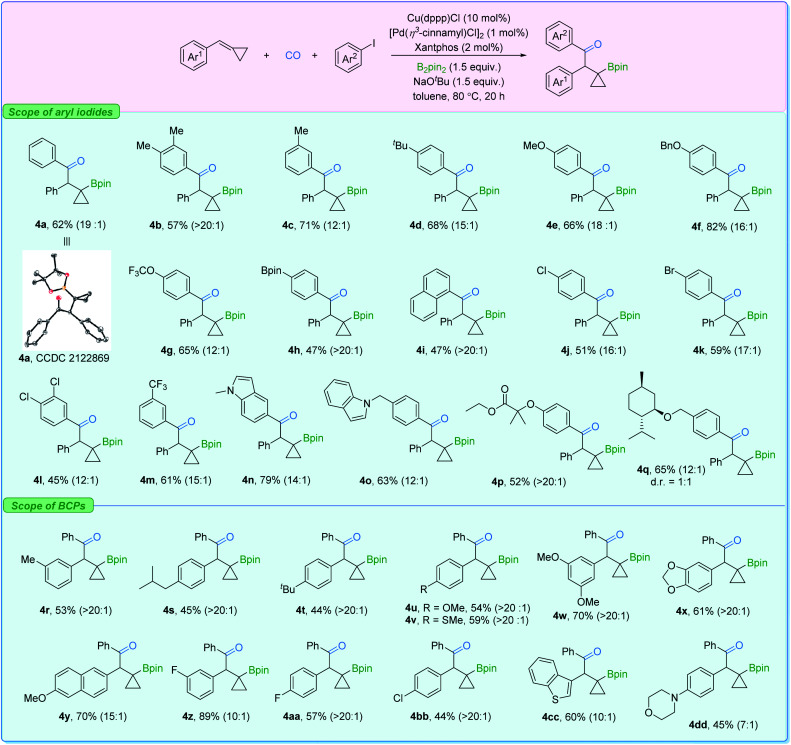
Substrate scope for product 4. BCP (0.2 mmol), aryl iodide (1.5 equiv.), Cu(dppp)Cl (10 mol%), [Pd(*η*^3^-cinnamyl)Cl]_2_ (1 mol%), xantphos (2 mmol%), B_2_pin_2_ (1.5 equiv.), NaO^*t*^Bu (1.5 equiv.), toluene (0.2 M), CO (3 bar), stirred at 80 °C for 20 h. r.r. values (4 : 3) were measured in crude mixtures by NMR or gas chromatography analysis. Displacement ellipsoid plot (30% probability level, without H).

Subsequently, the substrate scope for β-cyclopropylboryl ketone production was investigated (Fig. 6). Similarly, aryl iodides bearing a set of groups can be utilized without any problem (4a–4f). Polar functional groups at different positions on the aryl iodides such as OCF_3_, Bpin, Cl, Br, CF_3_, and indole (4g–4q) could also be employed. Furthermore, BCPs with electron-donating or -withdrawing groups showed good reactivity as well (4r–4bb). Heterocyclic cyclopropylidenemethanes (4cc and 4dd) were also suitable reactants here. However, BCPs with *ortho*-substituted or sterically bulky groups, which facilitate the β-carbon elimination on the β-cyclopropylboryl copper complex, gave poor regioselectivity in this transformation (see the ESI†).

**Fig. 7:**
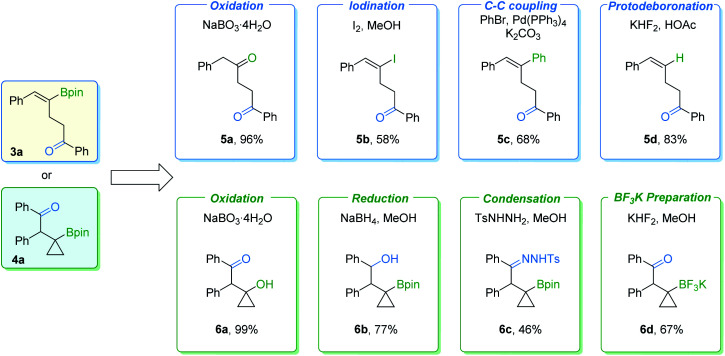
Derivatization of γ-vinylboryl ketone 3a and β-cyclopropylboryl ketone 4a.

In order to further demonstrate the synthetic value of these procedures, transformations of γ-vinylboryl ketone 3a and β-cyclopropylboryl ketone 4a were carried out (Fig. 7). γ-Vinylboryl ketone 3a can be oxidized into 1,4-diketone 5a in a one-pot manner. Vinylborane 3a can also be transformed with moderate to good yields of the corresponding products by other conversions, including iodination (5b), the Suzuki–Miyaura coupling reaction (5c), and protodeboronation (5d). Furthermore, cyclopropylboryl ketone 4a was successfully transformed into high-value cyclopropane-containing products (6a–6d) in moderate to excellent yields *via* oxidation, reduction, condensation, or react with KHF_2_. However, we failed in our attempt to transform the Bpin group of the cyclopropylboryl ketone into an amine group according to a reported method.^10^ Low conversion of the cyclopropylboryl ketone starting material was obtained.

## Conclusions

In summary, we have developed a novel catalyst-controlled borocarbonylation for the selective synthesis of γ-vinylboryl ketones and β-cyclopropylboryl ketones from benzylidenecyclopropanes and aryl iodides. In this catalyst system, choosing the appropriate catalytic system is the key for the regioselectivity control: γ-vinylboryl ketones were produced selectively in good yields with IPrCuCl and Pd(dppp)Cl_2_ as the catalyst source, and especially the IPr ligand improved the β-carbon elimination of the π-copper complex; the corresponding β-cyclopropylboryl ketones were obtained in high regioselectivity with Cu(dppp)Cl, [Pd(*η*^3^-cinnamyl)Cl]_2_ and xantphos as the catalysts. Synthetic transformations of the produced γ-vinylboryl ketones and β-cyclopropylboryl ketones clearly demonstrate the utility of this process.

## Author contributions

X.-F. W. conceived and directed the project. F.-P. W. performed all the experiments. F.-P. W. and X.-F. W. wrote the manuscript and ESI.†

## Conflicts of interest

There are no conflicts to declare.

## Supplementary Material

SC-013-D2SC00840H-s001

SC-013-D2SC00840H-s002
