# Supplementary material for: Catalyst-controlled selective borocarbonylation of benzylidenecyclopropanes: regiodivergent synthesis of γ-vinylboryl ketones and β-cyclopropylboryl ketones
Source: Chem Sci. 2022 Mar 21;13(15):4321–6. doi: 10.1039/d2sc00840h (PMC9006926; doi:10.1039/d2sc00840h)

Electronic Supplementary Material (ESI) for Chemical Science.
This journal is © The Royal Society of Chemistry 2022

Catalyst-Controlled Selective Borocarbonylation of Benzylidenecyclopropanes: Regiodivergent Synthesis of *γ*-vinylboryl ketones and *β*-cyclopropylboryl ketones

Fu-Peng Wu and Xiao-Feng Wu*

[a] Leibniz-Institut für Katalyse e.V., Albert-Einstein-Straße 29a, 18059 Rostock, Germany, E-mail: xiao-feng.wu@catalysis.de
[b] Dalian National Laboratory for Clean Energy, Dalian Institute of Chemical Physics, Chinese Academy of Sciences, 116023, Dalian, Liaoning, China, E-mail: xwu2020@dicp.ac.cn

Table of Contents

1. General information........S2
2. Optimization of borocarbonylation........S3
3. General procedure of borocarbonylation........S7
4. General procedure for preparing BCPs........S9
5. Spectroscopic data of products........S13
6. Derivatization of *γ*-vinylboryl ketone 3a and *β*-cyclopropylboryl ketone 4a........S51
7. References........S55
8. NMR Spectra of the *γ*-vinylboryl ketones, *β*-cyclopropylboryl ketones and BCPs........S56
9. Crystallographic reports for the x-ray structures........S225

1. General information

Reagents, solvents, and analytical methods:

Unless otherwise noted, all reactions were carried out under a carbon monoxide or nitrogen atmosphere. The benzylidenecyclopropanes were synthesized according to existing method, and reagents were ordered from Sigma-Aldrich, TCI, ABCR, and Acros, and used without purification. All solvents were dried by standard techniques and distilled prior to use. Column chromatography was performed on silica gel (200-300 meshes) using *N*-pentane (bp. 36.1 ^{o}C), dichloromethane and ethyl acetate as eluent. All NMR spectra were recorded at ambient temperature using Bruker Avance III HD 300 NMR (^{1}H, 300 MHz; ^{13}C{^{1}H}, 75 MHz; ^{11}B, 96 MHz, ^{19}F, 282 MHz), Bruker ARX 400 NMR spectrometers (^{1}H, 400 MHz; ^{13}C{^{1}H}, 101 MHz, ^{11}B, 128 MHz, ^{19}F 376 MHz). ^{1}H NMR chemical shifts are reported relative to TMS and were referenced via residual proton resonances of the corresponding deuterated solvent ($CDCl_3$: 7.26 ppm) whereas ^{13}C{^{1}H} NMR spectra are reported relative to TMS via the carbon signals of the deuterated solvent ($CDCl_3$: 77.0 ppm). Data for ^{1}H are reported as follows: chemical shift (δ ppm), multiplicity (s = singlet, d = doublet, t = triplet, q = quartet, quint = quintet, m = multiplet, br = broad), coupling constant (Hz), and integration. All ^{13}C NMR spectra were broad-band ^{1}H decoupled. However, signals for the carbon attach to boron, C(alkyl)-B or C(vinyl)-B, are usually too broad to observe in the ^{13}C{^{1}H} NMR spectra. High resolution mass spectra (HRMS) were recorded on an Agilent 6210 system. Gas chromatography (GC) analyses were performed on an Agilent HP-7890A instrument with an FID detector and HP-5 capillary column (polydimethylsiloxane with 5% phenyl groups, 30 m, 0.32 mm i.d. 0.25 μm film thickness) using argon as carrier gas.

Because of the high toxicity of carbon monoxide, all the reactions should be performed in an autoclave. The laboratory should be well-equipped with a CO detector and alarm system.

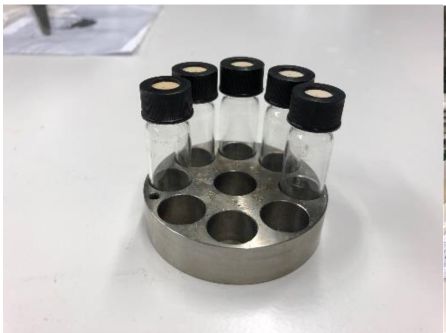

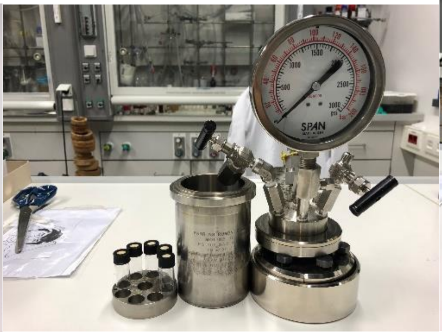

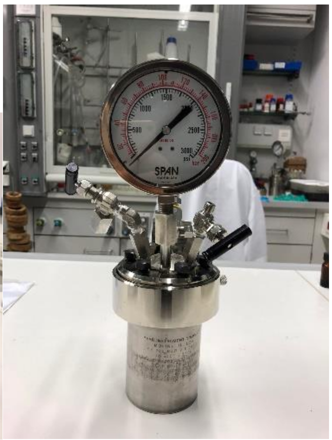

2. Optimization of borocarbonylation

2.1 Optimization of ligand for γ-vinylboryl ketones **3a**.

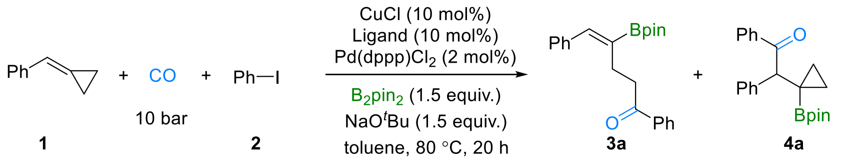

Entry	Ligand	**3a** (%)	**4a** (%)	r.r. (**3a**:**4a**)
1	None	0	0	-
2	DPPBz (**L1**)	0	0	-
3	DPPE (**L2**)	0	0	-
4	DPPP (**L3**)	22	22	1:1
5	DPPB (**L4**)	4	2	2:1
6	DPPF (**L5**)	19	10	2:1
7	DPEphos (**L6**)	39	18	2:1
8	PPh_3 (**L7**)	0	5	-
9	$BuPAd_2$ (**L8**)	64	0	>20:1
10	IPr (**L9**)	81	2	>20:1
11	IMes (**L10**)	0	0	-

2.2 Optimization of palladium source for γ-vinylboryl ketones **3a**.

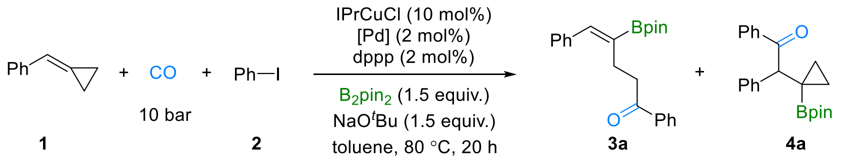

Entry	[Pd]	**3a** (%)	**4a** (%)	r.r. (**3a**:**4a**)
1	$Pd(OAc)_2$	80	5	16:1
2	$Pd(TFA)_2$	79	5	20:1
3	$PdCl_2$	58	0	>20:1
4	PdI_2	58	0	>20:1
5	$[Pd(\eta^3\text{-cinnamyl})Cl]_2$ (1 mol%)	64	0	>20:1
6	$[Pd(\eta^3\text{-}C_3H_5)Cl]_2$ (1 mol%)	77	5	15:1

2.3 Optimization of temperature for γ-vinylboryl ketones **3a**.

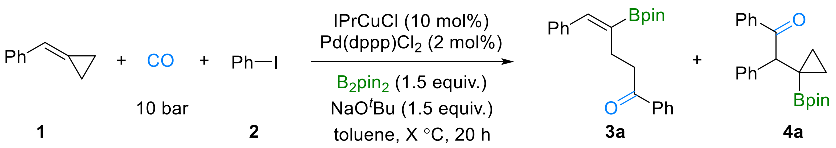

Entry	temperature	**3a** (%)	**4a** (%)	r.r. (**3a**:**4a**)
1	90 ºC	63	5	12:1
2	80 ºC	81	2	>20:1
3	70 ºC	62	2	>20:1
4	60 ºC	47	11	4:1

2.4 Optimization of the loading of B_2pin_2, NaO^{t}Bu and PhI for γ-vinylboryl ketones **3a**.

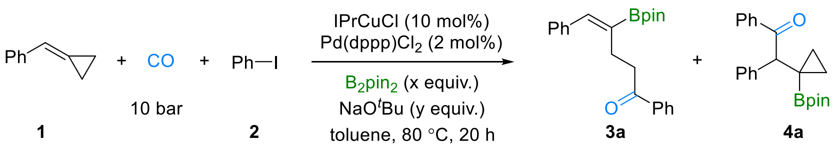

Entry	B_2pin_2	NaO^{t}Bu	PhI	**3a** (%)	**4a** (%)	r.r. (**3a**:**4a**)
1	1.2	1.5	1.5	73	5	15:1
2	1.7	1.5	1.5	74	3	>20:1
3	2.0	1.5	1.5	75	3	>20:1
4	1.5	1.2	1.5	50	5	10:1
5	1.5	1.7	1.5	82	3	>20:1
6	1.5	2.0	1.5	75	1	>20:1
7	1.5	1.5	1.2	72	2	>20:1
8	1.5	1.5	1.5	81	2	>20:1
9	1.5	1.5	1.7	85	2	>20:1
10	1.5	1.5	2.0	76	5	13:1

2.5 Optimization of ligand for *β*-cyclopropylboryl ketone **4a**.

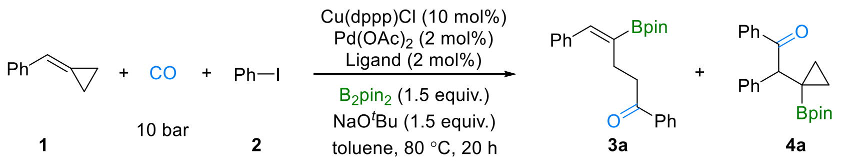

Entry	Ligand	**3a** (%)	**4a** (%)	r.r. (**3a**:**4a**)
1	None	2	25	1:13
2	DPEphos (**L6**)	5	35	1:7
3	PPh_3 (**L7**)	4	31	1:8
4	$BuPAd_2$ (**L8**)	29	2	10:1
5	IPr·HCl (**L9**)	44	23	2:1
6	Xantphos (**L11**)	7	50	1:7
7	Nixantphos (**L12**)	4	26	1:7
8	Sixantphos (**L13**)	4	33	1:8
9	BINAP (**L14**)	10	36	1:4
10	**L15**	3	31	1:10
11	**L16**	4	26	1:7
12	**L17**	2	17	1:9
13	**L18**	3	21	1:7
14	**L19**	4	26	1:7
15	**L20**	4	37	1:9
16	**L21**	4	21	1:5
17	**L22**	3	21	1:7
18	**L23**	3	36	1:12
19	**L24**	4	38	1:10
20	**L25**	2	29	1:15
21	(S,S)-Ph-BPE (**L26**)	2	0	-

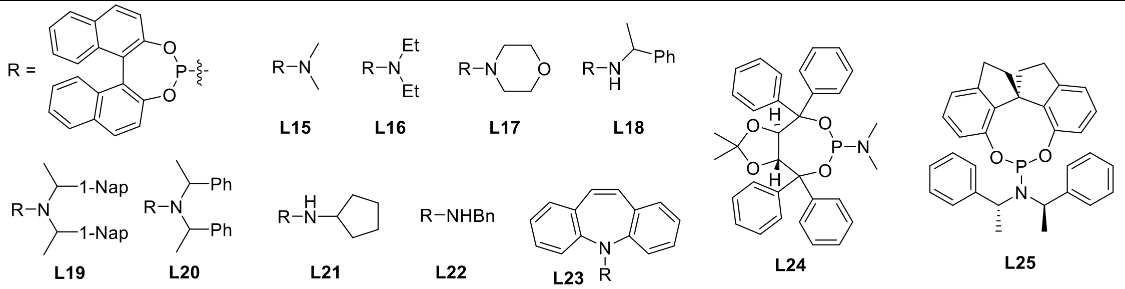

2.6 Optimization of palladium source for *β*-cyclopropylboryl ketone **4a**.

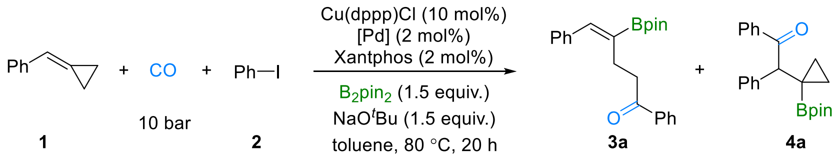

Entry	[Pd]	**3a** (%)	**4a** (%)	r.r. (**3a**:**4a**)
1	$Pd(OAc)_2$	7	50	1:8
2	$Pd(TFA)_2$	7	57	1:8
3	$PdCl_2$	8	29	1:4
4	$[Pd(cinnamyl)Cl]_2$ (1 mol%)	7	61	1:9
5	$[Pd(\eta^3\text{-}C_3H_5)Cl]_2$ (1 mol%)	6	54	1:9

2.7 Optimization of CO pressure.

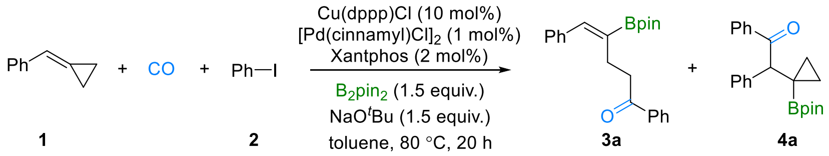

Entry	CO (bar)	**3a** (%)	**4a** (%)	r.r. (**3a**:**4a**)
1	20	7	55	1:8
2	10	7	61	1:9
3	6	9	64	1:7
4	3	4	76	1:19
5	1	5	73	1:15

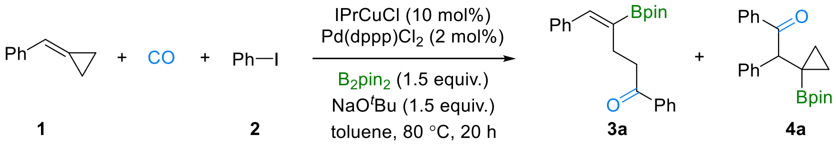

Entry	CO (bar)	**3a** (%)	**4a** (%)	r.r. (**3a**:**4a**)
1	20	84	2	>20:1
2	10	85	2	>20:1
3	6	91	7	13:1
4	3	91	9	10:1
5	1	70	9	8:1

3. General procedure of borocarbonylation

3.1 general borocarbonylation I (ring-opening).

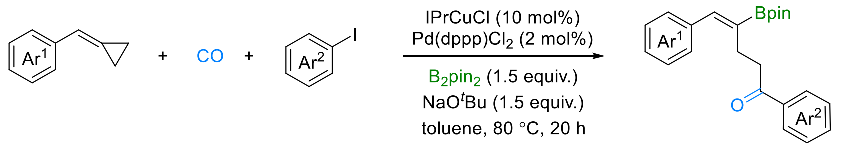

A 4 mL screw-cap vial was charged with IPrCuCl (9.7 mg, 10 mol%), Pd(dppp)Cl_2 (2.4 mg, 2 mol%), B_2pin_2 (76.2 mg, 1.5 equiv.), NaO^{t}Bu (28.8 mg, 1.5 equiv.) and an oven-dried stir bar. The vial was closed with a Teflon septum and cap and connected to the atmosphere via a needle. After toluene (1.0 mL), aryl iodides (1.7 equiv.) and BCP (0.2 mmol) were added with a syringe under argon atmosphere, the vial was moved to an alloy plate and put into a Parr 4560 series autoclave (300 mL) under argon atmosphere. At room temperature, the autoclave was flushed with CO three times and charged with 10 bar CO. The autoclave was placed on a heating plate equipped with a magnetic stirrer and an aluminum block. The reaction mixture was heated to 80 °C for 20 h. The reaction was then quenched upon addition of water (10 mL) and the mixture was extracted with EA (10 mL). The combined organic was dried using Na_2SO_4 and then concentrated in vacuo. The crude product was purified by column chromatography on silica gel to afford the corresponding product.

3.2 general borocarbonylation II (ring-remaining).

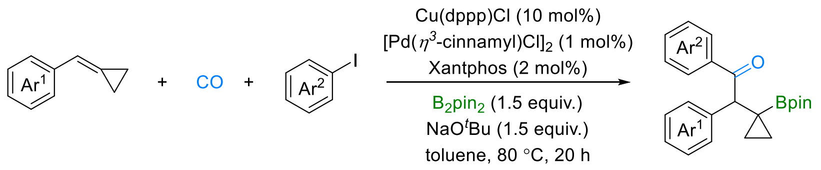

A 4 mL screw-cap vial was charged with Cu(dppp)Cl (10.2 mg, 10 mol%), [Pd(η^3-cinnamyl)Cl]$_2$ (1.0 mg, 1 mol%), Xantphos (2.3 mg, 2 mol%) B_2pin_2 (76.2 mg, 1.5 equiv.), NaO^{t}Bu (28.8 mg, 1.5 equiv.) and an oven-dried stir bar. The vial was closed with a Teflon septum and cap and connected to the atmosphere via a needle. After toluene (1.0 mL), aryl iodides (1.5 equiv.) and BCP (0.2 mmol) were added with a syringe under argon atmosphere, the vial was moved to an alloy plate and put into a Parr 4560 series autoclave (300 mL) under argon atmosphere. At room temperature, the autoclave was flushed with CO three times and charged with 10 bar CO. The autoclave was placed on a heating plate equipped with a magnetic stirrer and an aluminum block. The reaction mixture was heated to 80 °C for 20 h. The reaction was then quenched upon addition of water (10 mL) and the mixture was extracted with EA (10 mL). The combined organic was dried using Na_2SO_4 and then concentrated in vacuo. The crude product was purified by column chromatography on silica gel to afford the corresponding product.

3.3 general borocarbonylation III (ring- remaining).

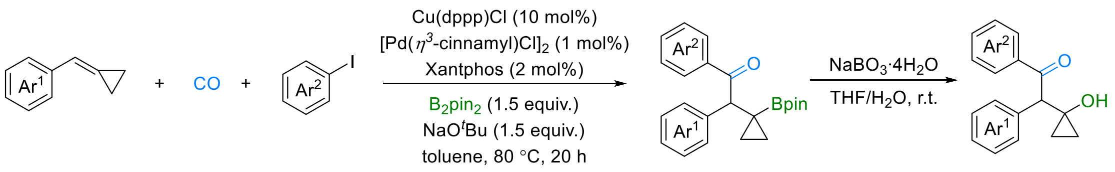

A 4 mL screw-cap vial was charged with Cu(dppp)Cl (10.2 mg, 10 mol%), [Pd(η^3-cinnamyl)Cl]$_2$ (1.0 mg, 1 mol%), Xantphos (2.3 mg, 2 mol%) B_2pin_2 (76.2 mg, 1.5 equiv.), NaO^{t}Bu (28.8 mg, 1.5 equiv.) and an oven-dried stir bar. The vial was closed with a Teflon septum and cap and connected to the atmosphere via a needle. After toluene (1.0 mL), aryl iodides (1.5 equiv.) and BCP (0.2 mmol) were added with a syringe under argon atmosphere, the vial was moved to an alloy plate and put into a Parr 4560 series autoclave (300 mL) under argon atmosphere. At room temperature, the autoclave was flushed with CO three times and charged with 10 bar CO. The autoclave was placed on a heating plate equipped with a magnetic stirrer and an aluminum block. The reaction mixture was heated to 80 ^{o}C for 20 h. The reaction was then quenched upon addition of water (10 mL) and the mixture was extracted with EA (10 mL) and then concentrated in vacuo. The crude product in THF (2 mL) and water (2 mL) was added $NaBO_3{\cdot}4H_2O$ (153.0 mg. 5 equiv). The reaction mixture was stirred vigorously at room temperature and determined by TLC analysis (2 h – 12 h). The reaction mixture was quenched with water and then extracted with ethyl acetate (5 mL). The combined organic layers were washed with brine (15 mL), dried over Na_2SO_4 and concentrated. The crude product was purified by column chromatography on silica gel to afford the corresponding product.

4. General procedure for preparing BCPs

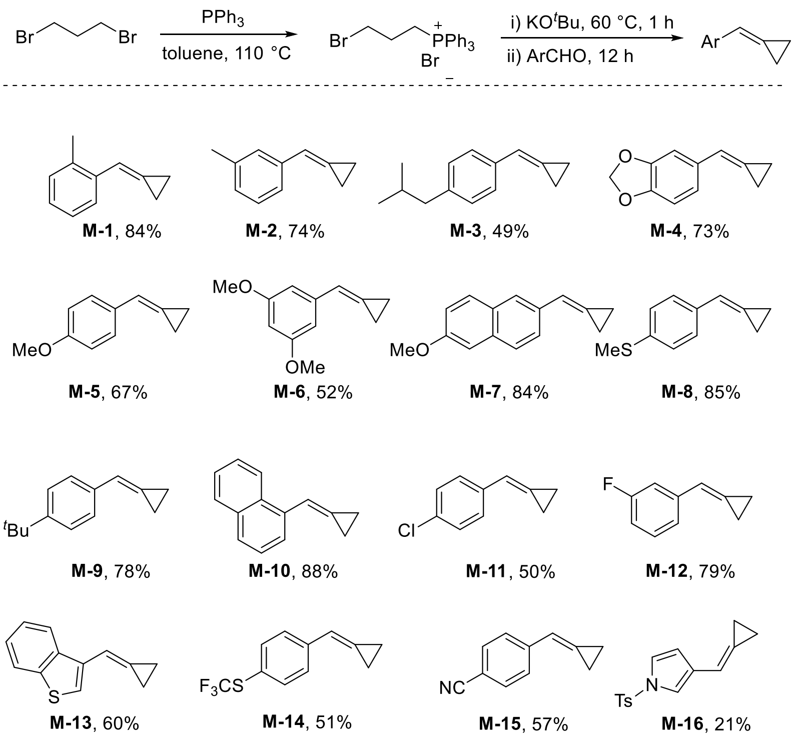

Representative Procedure[1]: To a 500 mL round bottom flask, 1,3-dibromopropane (41.6 g, 200 mmol), triphenylphosphine (52.5 g, 200 mmol) and toluene (100 mL) were added. The mixture was heated at 110 °C and maintained with stirring for 16 h. The white precipitate was filtered and washed with additional 50 mL of toluene and dried overnight in a vacuum stove, yielding 3-bromopropyltriphenylphosphonium bromide quantitatively. A flame-dried scintillation vial equipped with a stir bar was charged with (3-bromopropyl)triphenylphosphonium bromide (1.40 g, 3.0 mmol, 1.2 equiv) and KO^tBu (700 mg, 6.0 mmol, 2.4 equiv). The solids were suspended in THF (10 mL), and the mixture was allowed to stir at 60 °C for 1 h. After the solution had cooled to room temperature, benzaldehyde (265 mg, 2.5 mmol, 1.0 equiv) was added to the suspension dropwise over 2 min. The vial was sealed, and the suspension was allowed to stir at 60 °C for 12 h. The reaction mixture was cooled to room temperature and filtered through a celite pad, which was subsequently rinsed with Et2O (3 × 20 mL). The filtrate was concentrated, and the resulting crude mixture was purified by flash chromatography (*n*-pentane) to provide BCP substrate **1a** (195 mg, 60% yield) as a colorless oil.

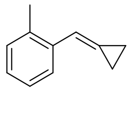

1-(Cyclopropylidenemethyl)-2-methylbenzene (M-1)

^{1}H NMR (300 MHz, CDCl$_3$) δ 7.75 (ddd, *J* = 7.4, 1.3, 0.6 Hz, 1H), 7.24 – 7.09 (m, 3H), 6.98 (p, *J* = 2.1 Hz, 1H), 2.41 (s, 3H), 1.46 – 1.39 (m, 2H), 1.23 – 1.17 (m, 2H).

^{13}C NMR (75 MHz, CDCl$_3$) δ 136.6, 134.9, 130.3, 126.6, 125.9, 125.8, 125.0, 115.5, 19.7, 4.1, 0.8.

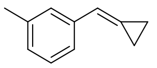

1-(Cyclopropylidenemethyl)-3-methylbenzene (M-2)

^{1}H NMR (300 MHz, CDCl$_3$) δ 7.34 – 7.27 (m, 2H), 7.21 – 7.13 (m, 1H), 7.02 – 6.95 (m, 1H), 6.68 (p, *J* = 2.1 Hz, 1H), 2.32 (s, 3H), 1.42 – 1.34 (m, 2H), 1.15 – 1.09 (m, 2H).

^{13}C NMR (75 MHz, CDCl$_3$) δ 138.2, 137.9, 128.3, 127.5, 127.3, 124.0, 123.7, 118.3, 21.5, 4.2, 0.5.

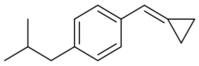

1-(Cyclopropylidenemethyl)-4-isobutylbenzene (M-3)

^{1}H NMR (300 MHz, CDCl$_3$) δ 7.47 – 7.43 (m, 2H), 7.13 – 7.09 (m, 2H), 6.73 (q, *J* = 2.0 Hz, 1H), 2.47 (d, *J* = 7.2 Hz, 2H), 1.94 – 1.80 (m, 1H), 1.46 – 1.38 (m, 2H), 1.20 – 1.13 (m, 2H), 0.92 (s, 3H), 0.90 (s, 3H).

^{13}C NMR (75 MHz, CDCl$_3$) δ 140.3, 135.7, 129.2, 126.3, 123.1, 118.1, 45.2, 30.2, 22.4, 4.1, 0.5.

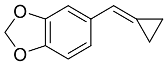

5-(Cyclopropylidenemethyl)benzo[*d*][1,3]dioxole (M-4)

^{1}H NMR (400 MHz, CDCl$_3$) δ 7.04 (d, *J* = 1.7 Hz, 1H), 6.80 (dd, *J* = 7.8, 1.5 Hz, 1H), 6.66 (d, *J* = 8.4 Hz, 1H), 6.56 (q, *J* = 2.1 Hz, 1H), 5.82 (s, 2H), 1.27 (ddd, *J* = 9.4, 5.3, 2.2 Hz, 2H), 1.07 – 1.02 (m, 2H).

^{13}C NMR (101 MHz, CDCl$_3$) δ 147.8, 146.4, 132.8, 122.1, 120.8, 117.8, 108.1, 106.1, 100.8, 4.0, 0.4.

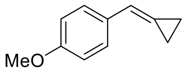

1-(Cyclopropylidenemethyl)-4-methoxybenzene (M-5)

^{1}H NMR (300 MHz, CDCl$_3$) δ 7.55 – 7.45 (m, 2H), 6.95 – 6.86 (m, 2H), 6.72 (t, *J* = 2.1 Hz, 1H), 3.83 (s, 3H), 1.45 – 1.36 (m, 2H), 1.23 – 1.13 (m, 2H).

^{13}C NMR (75 MHz, CDCl$_3$) δ 158.5, 131.2, 127.6, 121.6, 117.5, 113.9, 55.2, 4.0, 0.4.

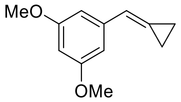

1-(Cyclopropylidenemethyl)-3,5-dimethoxybenzene (M-6)

^{1}H NMR (400 MHz, CDCl$_3$) δ 6.70 (dd, *J* = 13.0, 2.1 Hz, 3H), 6.37 (t, *J* = 2.3 Hz, 1H), 3.81 (s, 6H), 1.45 – 1.41 (m, 2H), 1.19 (ddd, *J* = 10.3, 5.9, 2.1 Hz, 2H).

^{13}C NMR (101 MHz, CDCl$_3$) δ 160.8, 140.2, 125.0, 118.2, 104.7, 98.9, 55.2, 26.9, 4.2, 0.6.

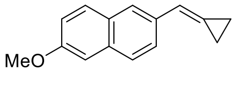

2-(Cyclopropylidenemethyl)-6-methoxynaphthalene (M-7)

^{1}H NMR (400 MHz, CDCl$_3$) δ 7.84 – 7.75 (m, 2H), 7.75 – 7.66 (m, 2H), 7.18 – 7.09 (m, 2H), 6.89 (p, *J* = 2.1 Hz, 1H), 3.92 (s, 3H), 1.50 (ddd, *J* = 9.5, 5.6, 2.3 Hz, 2H), 1.24 – 1.20 (m, 2H).

^{13}C NMR (101 MHz, CDCl$_3$) δ 157.4, 133.8, 133.6, 129.4, 129.1, 126.8, 125.6, 125.0, 123.7, 118.8, 118.4, 105.8, 55.3, 4.3, 0.6.

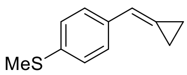

(4-(Cyclopropylidenemethyl)phenyl)(methyl)sulfane (M-8)

^{1}H NMR (400 MHz, CDCl$_3$) δ 7.48 – 7.44 (m, 2H), 7.25 – 7.21 (m, 2H), 6.71 (p, *J* = 2.1 Hz, 1H), 2.49 (s, 3H), 1.44 – 1.39 (m, 2H), 1.18 (ddd, *J* = 10.1, 5.7, 2.1 Hz, 2H).

^{13}C NMR (101 MHz, CDCl$_3$) δ 136.4, 135.4, 127.0, 126.8, 123.8, 117.6, 16.0, 4.1, 0.5.

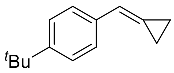

1-(*tert*-Butyl)-4-(cyclopropylidenemethyl)benzene (M-9)

^{1}H NMR (300 MHz, CDCl$_3$) δ 7.45 – 7.39 (m, 2H), 7.34 – 7.28 (m, 2H), 6.72 – 6.64 (m, 1H), 1.34 (dd, *J* = 14.4, 2.2 Hz, 2H), 1.27 (s, 9H), 1.12 – 1.07 (m, 2H).

^{13}C NMR (75 MHz, CDCl$_3$) δ 149.6, 135.5, 126.3, 125.3, 123.3, 117.9, 34.5, 31.3, 4.1, 0.5.

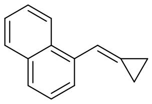

1-(Cyclopropylidenemethyl)naphthalene (M-10)

^{1}H NMR (300 MHz, CDCl$_3$) δ 8.25 (dd, *J* = 8.9, 1.1 Hz, 1H), 7.93 – 7.84 (m, 2H), 7.76 (d, *J* = 8.2 Hz, 1H), 7.60 – 7.45 (m, 4H), 1.52 – 1.44 (m, 2H), 1.35 – 1.27 (m, 2H).

^{13}C NMR (75 MHz, CDCl$_3$) δ 134.6, 133.8, 131.0, 128.6, 127.1, 126.7, 125.8, 125.6, 125.5, 123.8, 123.6, 114.7, 4.1, 1.5.

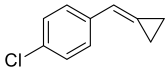

1-Chloro-4-(cyclopropylidenemethyl)benzene (M-11)

^{1}H NMR (300 MHz, CDCl$_3$) δ 7.49 – 7.42 (m, 2H), 7.35 – 7.24 (m, 2H), 6.71 (p, *J* = 2.1 Hz, 1H), 1.45 – 1.38 (m, 2H), 1.22 – 1.15 (m, 2H).

^{13}C NMR (75 MHz, CDCl$_3$) δ 136.7, 132.2, 128.6, 127.7, 125.2, 117.2, 4.1, 0.6.

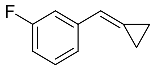

1-(Cyclopropylidenemethyl)-3-fluorobenzene (M-12)

^{1}H NMR (300 MHz, CDCl$_3$) δ 7.27 – 7.19 (m, 3H), 6.94 – 6.81 (m, 1H), 6.70 (p, *J* = 1.2 Hz, 1H), 1.45 – 1.37 (m, 2H), 1.21 – 1.14 (m, 2H).

^{13}C NMR (75 MHz, CDCl$_3$) δ 163.1 (d, *J* = 244.4 Hz), 140.6 (d, *J* = 7.7 Hz), 129.8 (d, *J* = 8.6 Hz), 126.0, 122.5 (d, *J* = 2.5 Hz), 117.4 (d, *J* = 2.8 Hz), 113.4 (d, *J* = 21.6 Hz), 112.8 (d, *J* = 21.8 Hz), 4.2, 0.6.

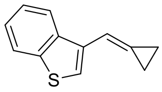

3-(Cyclopropylidenemethyl)benzo[b]thiophene (M-13)

^{1}H NMR (400 MHz, CDCl$_3$) δ 7.97 – 7.94 (m, 1H), 7.84 (ddd, *J* = 7.8, 1.3, 0.7 Hz, 1H), 7.51 (s, 1H), 7.40 – 7.30 (m, 2H), 7.06 (q, *J* = 2.0 Hz, 1H), 1.40 – 1.35 (m, 2H), 1.26 (ddd, *J* = 8.7, 4.9, 1.5 Hz, 2H).

^{13}C NMR (101 MHz, CDCl$_3$) δ 140.1, 137.9, 134.0, 125.2, 124.2, 124.0, 122.7, 121.7, 120.8, 110.5, 4.2, 2.0.

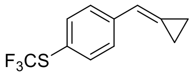

(4-(Cyclopropylidenemethyl)phenyl)(trifluoromethyl)sulfane (M-14)

^{1}H NMR (400 MHz, CDCl$_3$) δ 7.59 (q, *J* = 8.4 Hz, 4H), 6.77 (p, *J* = 2.0 Hz, 1H), 1.50 – 1.40 (m, 2H), 1.23 (ddd, *J* = 9.8, 5.7, 1.5 Hz, 2H).

^{13}C NMR (101 MHz, CDCl$_3$) δ 140.9, 136.5, 129.6 (q, *J* = 308.3 Hz), 127.8, 127.4, 121.8 (q, *J* = 2.2 Hz), 117.3, 4.3, 0.7.

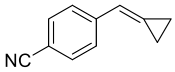

4-(Cyclopropylidenemethyl)benzonitrile (M-15)

^{1}H NMR (300 MHz, CDCl$_3$) δ 7.58 (s, 4H), 6.77 (p, *J* = 1.9 Hz, 1H), 1.51 – 1.42 (m, 2H), 1.29 – 1.19 (m, 2H).

^{13}C NMR (75 MHz, CDCl$_3$) δ 142.6, 132.2, 129.7, 126.9, 119.2, 117.2, 109.7, 4.4, 0.8.

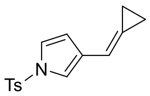

3-(Cyclopropylidenemethyl)-1-tosyl-1*H*-pyrrole (M-16)

^{1}H NMR (300 MHz, CDCl$_3$) δ 7.62 – 7.55 (m, 2H), 7.20 – 7.13 (m, 3H), 7.07 (dd, *J* = 2.4, 1.8 Hz, 1H), 6.37 (ddq, *J* = 3.5, 1.7, 0.6 Hz, 1H), 6.15 (td, *J* = 3.3, 0.7 Hz, 1H), 2.27 (s, 3H), 1.12 – 1.04 (m, 4H).

^{13}C NMR (75 MHz, CDCl$_3$) δ 144.8, 136.3, 133.6, 129.9, 126.8, 125.5, 122.0, 112.3, 111.3, 107.5, 21.6, 3.5, 2.2.

5. Spectroscopic data of products

5.1 Failed substrates.

Failed substrates:

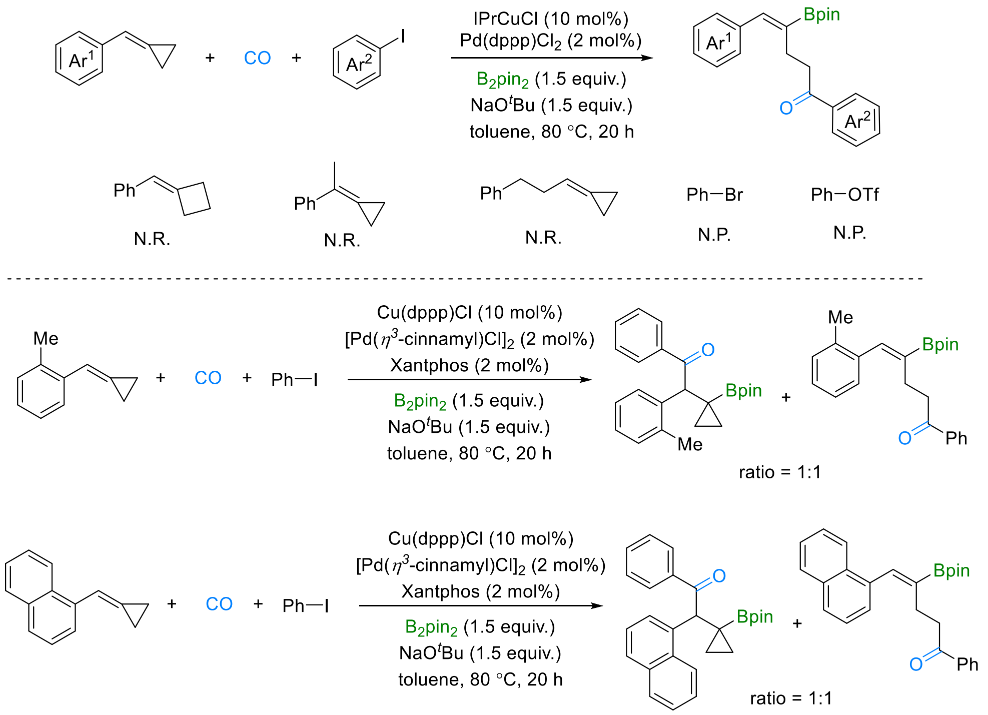

5.2 date of products.

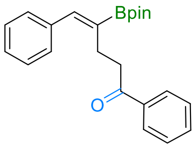

(*Z*)-1,5-Diphenyl-4-(4,4,5,5-tetramethyl-1,3,2-dioxaborolan-2-yl)pent-4-en-1-one (3a)

The title compound was prepared from (cyclopropylidenemethyl)benzene (27 uL, 0.2 mmol) and iodobenzene (39 uL, 0.34 mmol), according to general borocarbonylation I (ring open). The crude residue was purified by flash chromatography (pentane/EA = 20:1, Rf = 0.35) to give the product as a white solid (47.7 mg, 70%). X-ray (single-crystal) colorless block crystals of X-ray diffraction quality were obtained by slow evaporation of saturated solution of **3a** in ethyl acetate/*n*-pentane (CCDC 2123095).

^{1}H NMR (300 MHz, CDCl$_3$) δ 7.90 – 7.83 (m, 2H), 7.45 – 7.38 (m, 1H), 7.35 – 7.27 (m, 2H), 7.23 – 7.16 (m, 5H), 7.15 – 7.09 (m, 1H), 3.08 – 2.97 (m, 2H), 2.76 – 2.64 (m, 2H), 1.20 (s, 12H).

^{13}C NMR (75 MHz, CDCl$_3$) δ 200.0, 143.3, 137.5, 136.8, 132.8, 128.9, 128.4, 128.2, 128.2, 127.3, 83.5, 38.8, 24.8, 24.7.

^{11}B NMR (96 MHz, CDCl$_3$) δ 30.3.

HRMS (ESI): calcd for $[M+Na]^+$ $C_{23}H_{27}BO_3$ 384.1987, found: 384.1991.

IR (ATR): 2920, 2851, 1678, 1448, 1351, 1313, 1142, 747, 721, 688 cm^{-1}.

Mp: 56-58 °C

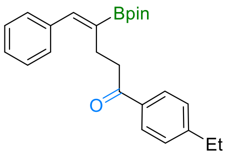

(*Z*)-1-(4-Ethylphenyl)-5-phenyl-4-(4,4,5,5-tetramethyl-1,3,2-dioxaborolan-2-yl)pent-4-en-1-one (3b)

The title compound was prepared from (cyclopropylidenemethyl)benzene (27 uL, 0.2 mmol) and 1-ethyl-4-iodobenzene (49 uL, 0.34 mmol), according to general borocarbonylation I (ring open). The crude residue was purified by flash chromatography (pentane/EA = 20:1, Rf = 0.36) to give the product as a colorless oil (47.8 mg, 61%).

^{1}H NMR (300 MHz, CDCl$_3$) δ 7.98 – 7.85 (m, 2H), 7.36 – 7.28 (m, 5H), 7.27 (d, J = 0.6 Hz, 1H), 7.26 – 7.18 (m, 2H), 3.21 – 3.04 (m, 2H), 2.88 – 2.76 (m, 2H), 2.70 (q, J = 7.6 Hz, 2H), 1.33 (s, 12H), 1.26 (t, J = 7.6 Hz, 3H).

^{13}C NMR (75 MHz, CDCl$_3$) δ 199.7, 149.7, 143.2, 137.5, 134.6, 128.9, 128.4, 128.2, 127.9, 127.3, 83.5, 38.8, 28.9, 24.9, 24.8, 15.2.

^{11}B NMR (96 MHz, CDCl$_3$) δ 30.3.

HRMS (ESI): calcd for $[M+Na]^+$ $C_{25}H_{31}BO_3$ 412.2300, found: 412.2305.

IR (ATR): 2970, 1673, 1350, 1319, 1142, 701 cm^{-1}.

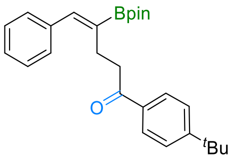

(*Z*)-1-(4-(*tert*-Butyl)phenyl)-5-phenyl-4-(4,4,5,5-tetramethyl-1,3,2-dioxaborolan-2-yl)pent-4-en-1-one (3c)

The title compound was prepared from (cyclopropylidenemethyl)benzene (27 uL, 0.2 mmol) and 1-(*tert*-butyl)-4-iodobenzene (60 uL, 0.34 mmol), according to general borocarbonylation I (ring open). The crude residue was purified by flash chromatography (pentane/EA = 20:1, Rf = 0.38) to give the product as a white solid (58.9 mg, 70%).

^{1}H NMR (300 MHz, CDCl$_3$) δ 8.00 – 7.86 (m, 2H), 7.49 – 7.41 (m, 2H), 7.36 – 7.28 (m, 5H), 7.25 – 7.13 (m, 1H), 3.19 – 3.07 (m, 2H), 2.90 – 2.76 (m, 2H), 1.35 (s, 9H), 1.33 (s, 12H).

^{13}C NMR (75 MHz, CDCl$_3$) δ 199.7, 156.4, 143.2, 137.5, 134.3, 128.9, 128.2, 128.2, 127.3, 125.4, 83.5, 38.7, 35.0, 31.1, 24.8, 24.8.

^{11}B NMR (96 MHz, CDCl$_3$) δ 30.2.

HRMS (ESI): calcd for $[M+Na]^+$ $C_{27}H_{35}BO_3$ 440.2612, found: 440.2616.

IR (ATR): 2965, 1679, 1370, 1313, 1266, 1140, 750, 703, 683, 492 cm^{-1}.

Mp: 115-117 ºC

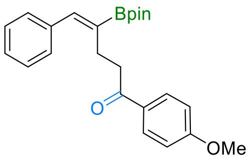

(*Z*)-1-(4-Methoxyphenyl)-5-phenyl-4-(4,4,5,5-tetramethyl-1,3,2-dioxaborolan-2-yl)pent-4-en-1-one (3d)

The title compound was prepared from (cyclopropylidenemethyl)benzene (27 uL, 0.2 mmol) and 1-iodo-4-methoxybenzene (79.6 mg, 0.34 mmol), according to general borocarbonylation I (ring open). The crude residue was purified by flash chromatography (pentane/EA = 15:1, Rf = 0.30) to give the product as a yellow oil (65.4 mg, 83%).

^{1}H NMR (300 MHz, $CDCl_3$) δ 8.04 – 7.88 (m, 2H), 7.36 – 7.28 (m, 5H), 7.26 – 7.19 (m, 1H), 6.96 – 6.84 (m, 2H), 3.86 (s, 3H), 3.17 – 3.03 (m, 2H), 2.88 – 2.72 (m, 2H), 1.32 (s, 12H).

^{13}C NMR (75 MHz, $CDCl_3$) δ 198.7, 163.2, 143.1, 137.5, 130.5, 130.0, 128.9, 128.2, 127.3, 113.6, 83.5, 55.4, 38.6, 25.0, 24.8.

^{11}B NMR (96 MHz, $CDCl_3$) δ 30.1.

HRMS (ESI): calcd for $[M+Na]^+$ $C_{24}H_{29}BO_4$ 414.2092, found: 414.2090.

IR (ATR): 1671, 1597, 1254, 1167, 1027, 698 cm^{-1}.

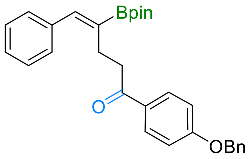

(*Z*)-1-(4-(Benzyloxy)phenyl)-5-phenyl-4-(4,4,5,5-tetramethyl-1,3,2-dioxaborolan-2-yl)pent-4-en-1-one (3e)

The title compound was prepared from (cyclopropylidenemethyl)benzene (27 uL, 0.2 mmol) and 1-(benzyloxy)-4-iodobenzene (105.4 mg, 0.34 mmol), according to general borocarbonylation I (ring open). The crude residue was purified by flash chromatography (pentane/EA = 15:1, Rf = 0.30) to give the product as a colorless oil (78.6 mg, 84%).

^{1}H NMR (300 MHz, $CDCl_3$) δ 8.02 – 7.94 (m, 2H), 7.47 – 7.28 (m, 10H), 7.27 – 7.21 (m, 1H), 7.03 – 6.96 (m, 2H), 5.13 (s, 2H), 3.16 – 3.04 (m, 2H), 2.90 – 2.74 (m, 2H), 1.33 (s, 12H).

^{13}C NMR (75 MHz, $CDCl_3$) δ 198.6, 162.3, 143.1, 137.4, 136.2, 130.5, 130.1, 128.9, 128.6, 128.2, 128.2, 127.4, 127.2, 114.4, 83.5, 70.0, 38.6, 25.0, 24.8.

^{11}B NMR (96 MHz, $CDCl_3$) δ 30.1.

HRMS (ESI): calcd for $[M+Na]^+$ $C_{30}H_{33}BO_4$ 490.2405, found: 490.2402.

IR (ATR): 2976, 1674, 1598, 1256, 1206, 1168, 1140, 964, 740, 696, 685 cm^{-1}.

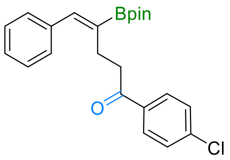

(*Z*)-1-(4-Chlorophenyl)-5-phenyl-4-(4,4,5,5-tetramethyl-1,3,2-dioxaborolan-2-yl)pent-4-en-1-one (3f)

The title compound was prepared from (cyclopropylidenemethyl)benzene (27 uL, 0.2 mmol) and 1-chloro-4-iodobenzene (80.9 mg, 0.34 mmol), according to general borocarbonylation I (ring open). The crude residue was purified by flash chromatography (pentane/EA = 20:1, Rf = 0.38) to give the product as a white solid (54.0 mg, 68%).

^{1}H NMR (300 MHz, CDCl$_3$) δ 7.98 – 7.86 (m, 2H), 7.43 – 7.36 (m, 2H), 7.36 – 7.28 (m, 5H), 7.26 – 7.19 (m, 1H), 3.16 – 3.06 (m, 2H), 2.86 – 2.74 (m, 2H), 1.32 (s, 12H).

^{13}C NMR (75 MHz, CDCl$_3$) δ 198.8, 143.5, 139.2, 137.4, 135.1, 129.7, 128.8, 128.7, 128.3, 127.3, 83.6, 38.8, 24.8, 24.8.

^{11}B NMR (96 MHz, CDCl$_3$) δ 30.3.

HRMS (ESI): calcd for $[M+Na]^+$ $C_{23}H_{26}BClO_3$ 418.1597, found: 418.1600.

IR (ATR): 2974, 1683, 1380, 1350, 1317, 1201, 1139, 1082, 698, 682 cm^{-1}.

Mp: 82-83 ℃

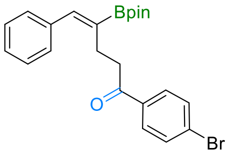

(*Z*)-1-(4-Bromophenyl)-5-phenyl-4-(4,4,5,5-tetramethyl-1,3,2-dioxaborolan-2-yl)pent-4-en-1-one (3g)

The title compound was prepared from (cyclopropylidenemethyl)benzene (27 uL, 0.2 mmol) and 1-bromo-4-iodobenzene (95.9 mg, 0.34 mmol), according to general borocarbonylation I (ring open). The crude residue was purified by flash chromatography (pentane/EA = 20:1, Rf = 0.37) to give the product as a colorless oil (59.8 mg, 68%).

^{1}H NMR (300 MHz, CDCl$_3$) δ 7.88 – 7.79 (m, 2H), 7.61 – 7.52 (m, 2H), 7.37 – 7.28 (m, 5H), 7.27 – 7.21 (m, 1H), 3.14 – 3.03 (m, 2H), 2.84 – 2.70 (m, 2H), 1.32 (s, 12H).

^{13}C NMR (75 MHz, CDCl$_3$) δ 199.0, 143.5, 137.4, 135.5, 131.7, 129.8, 128.8, 128.3, 127.9, 127.3, 83.6, 38.8, 24.8, 24.8.

^{11}B NMR (96 MHz, CDCl$_3$) δ 30.0.

HRMS (ESI): calcd for $[M+Na]^+$ $C_{23}H_{26}BBrO_3$ 462.1092, found: 462.1097.

IR (ATR): 2975, 1683, 1584, 1370, 1351, 1310, 1141, 1069, 1009, 750, 697 cm^{-1}.

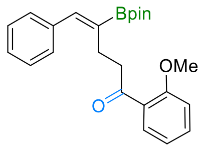

(*Z*)-1-(2-Methoxyphenyl)-5-phenyl-4-(4,4,5,5-tetramethyl-1,3,2-dioxaborolan-2-yl)pent-4-en-1-one (3h)

The title compound was prepared from (cyclopropylidenemethyl)benzene (27 uL, 0.2 mmol) and 1-iodo-2-methoxybenzene (75.9 mg, 0.34 mmol), according to general borocarbonylation I (ring open). The crude residue was purified by flash chromatography (pentane/EA = 15:1, Rf = 0.30) to give the product as a yellow oil (56.5 mg, 72%).

^{1}H NMR (300 MHz, CDCl$_3$) δ 7.66 (dd, *J* = 7.7, 2.2 Hz, 1H), 7.46 – 7.39 (m, 1H), 7.35 – 7.27 (m, 5H), 7.22 (ddd, *J* = 6.7, 5.6, 2.3 Hz, 1H), 6.97 (td, *J* = 7.4, 1.0 Hz, 1H), 6.92 (dd, *J* = 8.3, 1.1 Hz, 1H), 3.81 (s, 3H), 3.25 – 3.10 (m, 2H), 2.86 – 2.68 (m, 2H), 1.30 (s, 12H).

^{13}C NMR (75 MHz, CDCl$_3$) δ 202.3, 158.4, 142.6, 137.6, 133.0, 130.2, 129.0, 128.6, 128.1, 127.1, 120.5, 111.4, 83.4, 55.3, 43.6, 24.8, 24.5.

^{11}B NMR (96 MHz, CDCl$_3$) δ 30.3.

HRMS (ESI): calcd for [M+Na]$^+$ $C_{24}H_{29}BO_4$ 414.2092, found: 414.2093.

IR (ATR): 2973, 1670, 1596, 1484, 1464, 1436, 1284, 1243, 1179, 1156, 1111, 1022, 948, 754, 698 cm^{-1}.

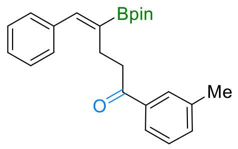

(*Z*)-5-Phenyl-4-(4,4,5,5-tetramethyl-1,3,2-dioxaborolan-2-yl)-1-(*m*-tolyl)pent-4-en-1-one (3i)

The title compound was prepared from (cyclopropylidenemethyl)benzene (27 uL, 0.2 mmol) and 1-iodo-3-methylbenzene (49 uL, 0.34 mmol), according to general borocarbonylation I (ring open). The crude residue was purified by flash chromatography (pentane/EA = 20:1, Rf = 0.35) to give the product as a white solid (50.6 mg, 67%).

^{1}H NMR (300 MHz, CDCl$_3$) δ 7.83 – 7.74 (m, 2H), 7.38 – 7.26 (m, 7H), 7.25 – 7.14 (m, 1H), 3.20 – 3.07 (m, 2H), 2.88 – 2.74 (m, 2H), 2.40 (s, 3H), 1.33 (s, 12H).

^{13}C NMR (75 MHz, CDCl$_3$) δ 200.2, 143.2, 138.2, 137.5, 136.9, 133.5, 128.9, 128.7, 128.3, 128.2, 127.3, 125.4, 83.5, 38.9, 24.8, 24.8, 21.3.

^{11}B NMR (96 MHz, CDCl$_3$) δ 30.1.

HRMS (ESI): calcd for [M+Na]$^+$ $C_{24}H_{29}BO_3$ 398.2143, found: 398.2151.

IR (ATR): 2979, 1677, 1371, 1339, 1315, 1282, 1156, 1141, 771, 758, 701, 690, 684 cm^{-1}.

Mp: 94-96 ºC

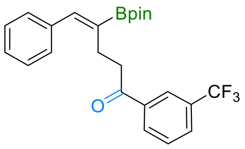

(*Z*)-5-Phenyl-4-(4,4,5,5-tetramethyl-1,3,2-dioxaborolan-2-yl)-1-(3-(trifluoromethyl)phenyl)pent-4-en-1-one (3j)

The title compound was prepared from (cyclopropylidenemethyl)benzene (27 uL, 0.2 mmol) and 1-iodo-3-(trifluoromethyl)benzene (49 uL, 0.34 mmol), according to general borocarbonylation I (ring open). The crude residue was purified by flash chromatography (pentane/EA = 20:1, Rf = 0.35) to give the product as a colorless oil (43.0 mg, 50%).

^{1}H NMR (300 MHz, $CDCl_3$) δ 8.24 (dt, *J* = 1.8, 1.1 Hz, 1H), 8.17 – 8.13 (m, 1H), 7.79 (ddt, *J* = 7.8, 1.2, 0.6 Hz, 1H), 7.57 (tt, *J* = 7.8, 0.8 Hz, 1H), 7.35 – 7.29 (m, 5H), 7.26 – 7.20 (m, 1H), 3.22 – 3.11 (m, 2H), 2.89 – 2.76 (m, 2H), 1.32 (s, 12H).

^{13}C NMR (75 MHz, $CDCl_3$) δ 198.7, 143.7, 137.4, 137.3, 131.4 (q. *J* = 1.3 Hz), 131.1 (q, *J* = 32.9 Hz), 130.1, 129.2 (d, *J* = 3.7 Hz), 129.1, 128.3, 127.4, 125.1 (q, *J* = 3.6 Hz), 123.7 (d, *J* = 272.5 Hz), 83.7, 38.9, 24.8, 24.1.

^{11}B NMR (96 MHz, $CDCl_3$) δ 30.3.

^{19}F NMR (282 MHz, $CDCl_3$) δ -62.7.

HRMS (ESI): calcd for $[M+Na]^+$ $C_{24}H_{26}BF_3O_3$ 452.1860, found: 452.1865.

IR (ATR): 2977, 1689, 1372, 1327, 1255, 1165, 1125, 1071, 755, 693, 654 cm^{-1}.

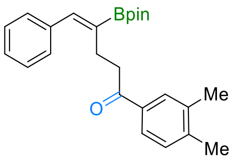

(*Z*)-1-(3,4-Dimethylphenyl)-5-phenyl-4-(4,4,5,5-tetramethyl-1,3,2-dioxaborolan-2-yl)pent-4-en-1-one (3k)

The title compound was prepared from (cyclopropylidenemethyl)benzene (27 uL, 0.2 mmol) and 4-iodo-1,2-dimethylbenzene (48 uL, 0.34 mmol), according to general borocarbonylation I (ring open). The crude residue was purified by flash chromatography (pentane/EA = 20:1, Rf = 0.32) to give the product as a white solid (55.0 mg, 71%).

^{1}H NMR (300 MHz, $CDCl_3$) δ 7.82 – 7.65 (m, 2H), 7.40 – 7.27 (m, 5H), 7.26 – 7.21 (m, 1H), 7.19 (d, *J* = 7.6 Hz, 1H), 3.18 – 3.06 (m, 2H), 2.88 – 2.75 (m, 2H), 2.30 (s, 6H), 1.33 (s, 12H).

^{13}C NMR (75 MHz, $CDCl_3$) δ 200.0, 143.1, 142.2, 137.5, 136.7, 134.8, 129.7, 129.3, 128.9, 128.2, 127.3, 126.0, 83.5, 38.8, 25.0, 24.8, 20.0, 19.8.

^{11}B NMR (96 MHz, $CDCl_3$) δ 30.1.

HRMS (ESI): calcd for $[M+Na]^+$ $C_{25}H_{31}BO_3$ 412.2300, found: 412.2300.

IR (ATR): 2977, 1681, 1377, 1352, 1341, 1315, 1278, 1140, 699, 686 cm^{-1}.

Mp: 114-116 ºC

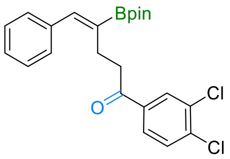

(*Z*)-1-(3,4-Dichlorophenyl)-5-phenyl-4-(4,4,5,5-tetramethyl-1,3,2-dioxaborolan-2-yl)pent-4-en-1-one (3l)

The title compound was prepared from (cyclopropylidenemethyl)benzene (27 uL, 0.2 mmol) and 1,2-dichloro-4-iodobenzene (92.8 mg, 0.34 mmol), according to general borocarbonylation I (ring open). The crude residue was purified by flash chromatography (pentane/EA = 20:1, Rf = 0.40) to give the product as a colorless oil (37.9 mg, 44%).

^{1}H NMR (300 MHz, $CDCl_3$) δ 8.08 (d, *J* = 2.0 Hz, 1H), 7.79 (dd, *J* = 8.4, 2.1 Hz, 1H), 7.50 (d, *J* = 8.4 Hz, 1H), 7.36 – 7.28 (m, 5H), 7.26 – 7.20 (m, 1H), 3.11 – 3.03 (m, 2H), 2.78 (dd, *J* = 8.3, 6.5 Hz, 2H), 1.33 (s, 12H).

^{13}C NMR (75 MHz, $CDCl_3$) δ 197.9, 143.7, 137.3, 136.3, 133.1, 130.6, 130.4, 128.8, 128.3, 127.4, 127.3, 83.7, 39.0, 24.9, 24.8.

^{11}B NMR (96 MHz, $CDCl_3$) δ 30.5.

HRMS (ESI): calcd for $[M+Na]^+$ $C_{23}H_{25}BCl_2O_3$ 452.1207, found: 452.1204.

IR (ATR): 2975, 1686, 1372, 1350, 1310, 1269, 1140, 1029, 752, 698, 674 cm^{-1}.

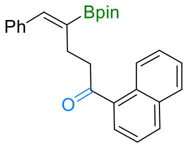

(*Z*)-1-(Naphthalen-1-yl)-5-phenyl-4-(4,4,5,5-tetramethyl-1,3,2-dioxaborolan-2-yl)pent-4-en-1-one (3m)

The title compound was prepared from (cyclopropylidenemethyl)benzene (27 uL, 0.2 mmol) and 1-iodonaphthalene (50 uL, 0.34 mmol), according to general borocarbonylation I (ring open). The crude residue was purified by flash chromatography (pentane/EA = 20:1, Rf = 0.35) to give the product as a yellow oil (61.6 mg, 75%).

^{1}H NMR (300 MHz, $CDCl_3$) δ 8.61 (ddd, *J* = 8.0, 1.7, 0.8 Hz, 1H), 7.99 – 7.93 (m, 1H), 7.91 – 7.84 (m, 2H), 7.62 – 7.51 (m, 2H), 7.51 – 7.45 (m, 1H), 7.38 – 7.28 (m, 5H), 7.27 – 7.19 (m, 1H), 3.33 – 3.20 (m, 2H), 2.99 – 2.80 (m, 2H), 1.32 (s, 12H).

^{13}C NMR (75 MHz, $CDCl_3$) δ 204.3, 143.2, 137.4, 135.9, 133.9, 132.3, 130.2, 128.9, 128.3, 128.2, 127.7, 127.6, 127.3, 126.3, 125.9, 124.3, 83.5, 42.2, 25.0, 24.8.

^{11}B NMR (96 MHz, $CDCl_3$) δ 30.0.

HRMS (ESI): calcd for $[M+Na]^+$ $C_{27}H_{29}BO_3$ 434.2163, found: 434.2141.

IR (ATR): 2972, 1677, 1380, 1371, 1313, 1282, 1136, 1098, 787, 772, 749, 698, 686, 482 cm^{-1}.

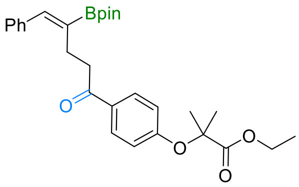

Ethyl (*Z*)-2-methyl-2-(4-(5-phenyl-4-(4,4,5,5-tetramethyl-1,3,2-dioxaborolan-2-yl)pent-4-enoyl)phenoxy)propanoate (3n)

The title compound was prepared from (cyclopropylidenemethyl)benzene (27 uL, 0.2 mmol) and ethyl 2-(4-iodophenoxy)-2-methylpropanoate (113.6 mg, 0.34 mmol), according to general borocarbonylation I (ring open). The crude residue was purified by flash chromatography (pentane/EA = 15:1, Rf = 0.25) to give the product as a colorless oil (61.2 mg, 62%).

^{1}H NMR (300 MHz, $CDCl_3$) δ 7.94 – 7.87 (m, 2H), 7.35 – 7.27 (m, 5H), 7.26 – 7.19 (m, 1H), 6.85 – 6.75 (m, 2H), 4.22 (q, *J* = 7.2 Hz, 2H), 3.14 – 3.02 (m, 2H), 2.78 (dd, *J* = 9.8, 7.9 Hz, 2H), 1.65 (s, 6H), 1.31 (s, 12H), 1.21 (t, *J* = 7.2 Hz, 3H).

^{13}C NMR (75 MHz, $CDCl_3$) δ 198.6, 173.7, 159.5, 143.2, 137.4, 130.5, 130.0, 128.9, 128.2, 127.3, 117.3, 83.5, 79.2, 61.6, 38.6, 25.3, 24.9, 24.8, 14.0.

^{11}B NMR (96 MHz, $CDCl_3$) δ 29.8.

HRMS (ESI): calcd for $[M+H]^+$ $C_{29}H_{37}BO_6$ 492.2798, found: 492.2809.

IR (ATR): 2979, 1732, 1676, 1597, 1367, 1286, 1244, 1169, 1137, 1021, 966, 835, 699, 493 cm^{-1}.

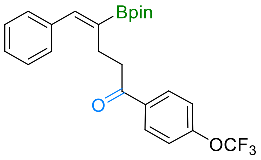

(*Z*)-5-Phenyl-4-(4,4,5,5-tetramethyl-1,3,2-dioxaborolan-2-yl)-1-(4-(trifluoromethoxy)phenyl)pent-4-en-1-one (3o)

The title compound was prepared from (cyclopropylidenemethyl)benzene (27 uL, 0.2 mmol) and 1-iodo-4-(trifluoromethoxy)benzene (51 uL, 0.34 mmol), according to general borocarbonylation I (ring open). The crude residue was purified by flash chromatography (pentane/EA = 20:1, Rf = 0.40) to give the product as a colorless oil (59.0 mg, 66%).

^{1}H NMR (400 MHz, $CDCl_3$) δ 8.01 – 7.85 (m, 2H), 7.28 – 7.20 (m, 5H), 7.19 – 7.12 (m, 3H), 3.08 – 2.98 (m, 2H), 2.77 – 2.66 (m, 2H), 1.23 (s, 12H).

^{13}C NMR (101 MHz, $CDCl_3$) δ 198.5, 152.4 (q, *J* = 1.8 Hz), 143.5, 137.4, 135.0, 130.2, 128.8, 128.3, 127.4, 120.3, 120.3 (q, *J* = 258.5 Hz), 83.6, 38.9, 24.8, 24.7.

^{11}B NMR (128 MHz, $CDCl_3$) δ 30.6.

^{19}F NMR (376 MHz, $CDCl_3$) δ -57.59.

HRMS (ESI): calcd for $[M+Na]^+$ $C_{24}H_{26}BF_3O_4$ 468.1810, found: 468.1813.

IR (ATR): 1686, 1372, 1251, 1204, 1162, 1141, 1109, 1082, 859, 750, 698 cm^{-1}.

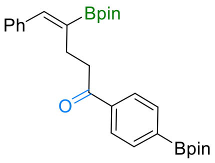

(*Z*)-5-Phenyl-4-(4,4,5,5-tetramethyl-1,3,2-dioxaborolan-2-yl)-1-(4-(4,4,5,5-tetramethyl-1,3,2-dioxaborolan-2-yl)phenyl)pent-4-en-1-one (3p)

The title compound was prepared from (cyclopropylidenemethyl)benzene (27 uL, 0.2 mmol) and 2-(4-iodophenyl)-4,4,5,5-tetramethyl-1,3,2-dioxaborolane (111.2 mg, 0.34 mmol), according to general borocarbonylation I (ring open). The crude residue was purified by flash chromatography (pentane/EA = 20:1, Rf = 0.18) to give the product as a yellow oil (83.8 mg, 86%).

^{1}H NMR (300 MHz, $CDCl_3$) δ 7.98 – 7.93 (m, 2H), 7.90 – 7.84 (m, 2H), 7.36 – 7.29 (m, 5H), 7.26 – 7.19 (m, 1H), 3.20 – 3.09 (m, 2H), 2.81 (dd, J = 9.6, 6.9 Hz, 2H), 1.36 (s, 12H), 1.32 (s, 12H).

^{13}C NMR (75 MHz, $CDCl_3$) δ 200.3, 143.3, 138.8, 137.5, 134.8, 128.9, 128.2, 127.3, 127.2, 124.2, 84.1, 83.6, 39.0, 25.0, 24.9, 24.8, 24.8.

^{11}B NMR (96 MHz, $CDCl_3$) δ 30.1.

HRMS (ESI): calcd for $[M+Na]^+$ $C_{29}H_{38}B_2O_5$ 509.2875, found: 509.2886.

IR (ATR): 2976, 1678, 1598, 1367, 1204, 1153, 1108, 948, 882, 828, 743, 697, 550, 495 cm^{-1}.

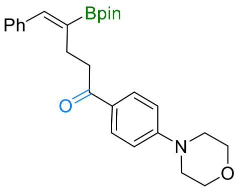

(*Z*)-1-(4-Morpholinophenyl)-5-phenyl-4-(4,4,5,5-tetramethyl-1,3,2-dioxaborolan-2-yl)pent-4-en-1-one (3q)

The title compound was prepared from (cyclopropylidenemethyl)benzene (27 uL, 0.2 mmol) and 4-(4-iodophenyl)morpholine (97.9 mg, 0.34 mmol), according to general borocarbonylation I (ring open). The crude residue was purified by flash chromatography (pentane/EA = 10:1, Rf = 0.25) to give the product as a yellow oil (80.0 mg, 89%).

^{1}H NMR (300 MHz, $CDCl_3$) δ 7.96 – 7.87 (m, 2H), 7.37 – 7.28 (m, 5H), 7.26 – 7.19 (m, 1H), 6.88 – 6.81 (m, 2H), 3.88 – 3.82 (m, 4H), 3.32 – 3.24 (m, 4H), 3.11 – 3.03 (m, 2H), 2.86 – 2.71 (m, 2H), 1.32 (s, 12H).

^{13}C NMR (75 MHz, $CDCl_3$) δ 198.3, 154.0, 142.9, 137.5, 130.2, 128.9, 128.2, 127.8, 127.2, 113.3, 83.5, 66.5, 47.6, 38.3, 25.1, 24.8.

^{11}B NMR (96 MHz, $CDCl_3$) δ 30.0.

HRMS (ESI): calcd for $[M+H]^+$ $C_{27}H_{34}BNO_4$ 447.2695, found: 447.2703.

IR (ATR): 2973, 1666, 1596, 1186, 1140, 1112, 926 cm^{-1}.

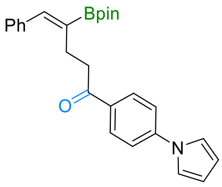

(*Z*)-1-(4-(1*H*-Pyrrol-1-yl)phenyl)-5-phenyl-4-(4,4,5,5-tetramethyl-1,3,2-dioxaborolan-2-yl)pent-4-en-1-one (3r)

The title compound was prepared from (cyclopropylidenemethyl)benzene (27 uL, 0.2 mmol) and 1-(4-iodophenyl)-1*H*-pyrrole (91.1 mg, 0.34 mmol), according to general borocarbonylation I (ring open). The crude residue was purified by flash chromatography (pentane/EA = 20:1, Rf = 0.30) to give the product as a yellow oil (72.6 mg, 85%).

^{1}H NMR (300 MHz, CDCl$_3$) δ 8.10 – 8.02 (m, 2H), 7.48 – 7.39 (m, 2H), 7.34 (d, *J* = 4.5 Hz, 5H), 7.28 – 7.21 (m, 1H), 7.19 – 7.14 (m, 2H), 6.43 – 6.34 (m, 2H), 3.20 – 3.07 (m, 2H), 2.92 – 2.76 (m, 2H), 1.33 (s, 12H).

^{13}C NMR (75 MHz, CDCl$_3$) δ 198.7, 143.8, 143.4, 137.4, 133.7, 130.1, 128.9, 128.3, 127.3, 119.3, 119.0, 111.5, 83.6, 38.8, 25.0, 24.8.

^{11}B NMR (96 MHz, CDCl$_3$) δ 30.1.

HRMS (ESI): calcd for [M+Na]$^+$ $C_{27}H_{30}BNO_3$ 449.2252, found: 449.2260.

IR (ATR): 1610, 1517, 1351, 1329, 1145, 1130, 1062, 1045, 840, 730, 696 cm^{-1}.

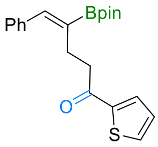

(*Z*)-5-Phenyl-4-(4,4,5,5-tetramethyl-1,3,2-dioxaborolan-2-yl)-1-(thiophen-2-yl)pent-4-en-1-one (3s)

The title compound was prepared from (cyclopropylidenemethyl)benzene (27 uL, 0.2 mmol) and 2-iodothiophene (38 uL, 0.34 mmol), according to general borocarbonylation I (ring open). The crude residue was purified by flash chromatography (pentane/EA = 20:1, Rf = 0.25) to give the product as a yellow oil (47.6 mg, 65%).

^{1}H NMR (300 MHz, CDCl$_3$) δ 7.73 (dd, *J* = 3.8, 1.1 Hz, 1H), 7.60 (dd, *J* = 5.0, 1.2 Hz, 1H), 7.37 – 7.28 (m, 5H), 7.27 – 7.21 (m, 1H), 7.10 (dd, *J* = 5.0, 3.9 Hz, 1H), 3.14 – 3.03 (m, 2H), 2.87 – 2.76 (m, 2H), 1.32 (s, 12H).

^{13}C NMR (75 MHz, CDCl$_3$) δ 193.0, 144.4, 143.4, 137.4, 133.3, 131.9, 128.9, 128.3, 127.9, 127.3, 83.6, 39.5, 25.1, 24.8.

^{11}B NMR (96 MHz, CDCl$_3$) δ 30.4.

HRMS (ESI): calcd for [M+Na]$^+$ $C_{21}H_{25}BO_3S$ 390.1551, found: 390.1552.

IR (ATR): 2975, 1659, 1413, 1371, 1351, 1310, 1267, 1141, 1081, 755, 722, 699 cm^{-1}.

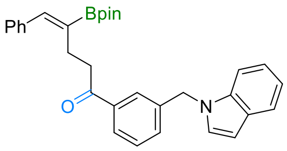

(*Z*)-1-(4-((1*H*-Indol-1-yl)methyl)phenyl)-5-phenyl-4-(4,4,5,5-tetramethyl-1,3,2-dioxaborolan-2-yl)pent-4-en-1-one (3t)

The title compound was prepared from (cyclopropylidenemethyl)benzene (27 uL, 0.2 mmol) and 1-(3-iodobenzyl)-1*H*-indole (113.2 mg, 0.34 mmol), according to general borocarbonylation I (ring open). The crude residue was purified by flash chromatography (pentane/EA = 20:1, Rf = 0.15) to give the product as a colorless oil (78.0 mg, 79%).

^{1}H NMR (300 MHz, $CDCl_3$) δ 7.82 – 7.75 (m, 2H), 7.58 (ddd, *J* = 7.4, 1.5, 0.8 Hz, 1H), 7.29 – 7.21 (m, 6H), 7.21 – 7.13 (m, 2H), 7.13 – 7.01 (m, 4H), 6.49 (dd, *J* = 3.2, 0.9 Hz, 1H), 5.27 (s, 2H), 3.09 – 2.97 (m, 2H), 2.74 (dd, *J* = 9.1, 6.6 Hz, 2H), 1.23 (s, 12H).

^{13}C NMR (75 MHz, $CDCl_3$) δ 199.6, 143.3, 138.1, 137.4, 137.2, 136.1, 131.0, 129.0, 128.9, 128.7, 128.2, 128.0, 127.6, 127.3, 126.5, 121.8, 121.0, 119.6, 109.5, 102.0, 83.5, 49.8, 38.8, 24.8, 24.6.

^{11}B NMR (96 MHz, $CDCl_3$) δ 29.9.

HRMS (ESI): calcd for $[M+Na]^+$ $C_{32}H_{34}BNO_3$ 513.2565, found: 513.2568.

IR (ATR): 1681, 1462, 1348, 1311, 1254, 1141, 738, 690, 425 cm^{-1}.

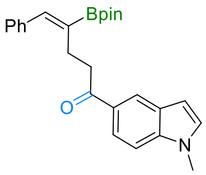

(*Z*)-1-(1-Methyl-1*H*-indol-5-yl)-5-phenyl-4-(4,4,5,5-tetramethyl-1,3,2-dioxaborolan-2-yl)pent-4-en-1-one (3u)

The title compound was prepared from (cyclopropylidenemethyl)benzene (27 uL, 0.2 mmol) and 5-iodo-1-methyl-1*H*-indole (87.0 mg, 0.34 mmol), according to general borocarbonylation I (ring open). The crude residue was purified by flash chromatography (pentane/EA = 5:1, Rf = 0.25) to give the product as a red solid (56.9 mg, 57%).

^{1}H NMR (300 MHz, $CDCl_3$) δ 8.37 (dd, *J* = 1.7, 0.7 Hz, 1H), 7.94 (dd, *J* = 8.7, 1.7 Hz, 1H), 7.40 – 7.29 (m, 6H), 7.26 – 7.19 (m, 1H), 7.11 (d, *J* = 3.1 Hz, 1H), 6.59 (dd, *J* = 3.2, 0.9 Hz, 1H), 3.81 (s, 3H), 3.29 – 3.19 (m, 2H), 2.94 – 2.81 (m, 2H), 1.35 (s, 12H).

^{13}C NMR (75 MHz, $CDCl_3$) δ 200.1, 142.9, 139.0, 137.5, 130.2, 129.0, 128.9, 128.2, 127.8, 127.2, 123.0, 121.9, 109.0, 102.9, 83.5, 38.8, 33.0, 25.4, 24.8.

^{11}B NMR (96 MHz, $CDCl_3$) δ 30.4.

HRMS (ESI): calcd for $[M+H]^+$ $C_{26}H_{30}BNO_3$ 415.2433, found: 415.2441.

IR (ATR): 1666, 1346, 1312, 1299, 1137, 732, 685 cm^{-1}.

Mp: 99-101 ºC

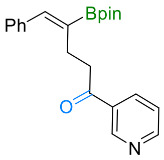

(*Z*)-5-Phenyl-1-(pyridin-3-yl)-4-(4,4,5,5-tetramethyl-1,3,2-dioxaborolan-2-yl)pent-4-en-1-one (3v)

The title compound was prepared from (cyclopropylidenemethyl)benzene (27 uL, 0.2 mmol) and 3-iodopyridine (69.7 mg, 0.34 mmol), according to general borocarbonylation I (ring open). The crude residue was purified by flash chromatography (pentane/EA = 5:1, Rf = 0.20) to give the product as a yellow oil (41.9 mg, 58%).

^{1}H NMR (300 MHz, DMSO) δ 9.08 (br, 1H), 8.29 (d, *J* = 7.8 Hz, 1H), 7.63 (br, 1H), 7.41 – 7.23 (m, 5H), 7.18 (s, 1H), 3.30 – 3.14 (m, 2H), 2.66 (t, *J* = 7.3 Hz, 2H), 1.23 (s, 12H).

^{13}C NMR (75 MHz, DMSO) δ 198.9, 142.6, 136.8, 135.6, 128.8, 128.4, 127.6, 83.4, 38.3, 24.6, 24.1.

^{11}B NMR (96 MHz, CDCl$_3$) δ 30.4.

HRMS (ESI): calcd for $[M+Na]^+$ $C_{22}H_{26}BNO_3$ 386.1902, found: 386.1903.

IR (ATR): 1685, 1373, 1314, 1144, 1052, 1023, 1004, 821, 758, 704, 620 cm^{-1}.

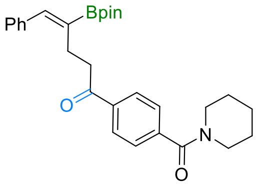

(*Z*)-5-Phenyl-1-(4-(piperidine-1-carbonyl)phenyl)-4-(4,4,5,5-tetramethyl-1,3,2-dioxaborolan-2-yl)pent-4-en-1-one (3w)

The title compound was prepared from (cyclopropylidenemethyl)benzene (27 uL, 0.2 mmol) and (4-iodophenyl)(piperidin-1-yl)methanone (107.1 mg, 0.34 mmol), according to general borocarbonylation I (ring open). The crude residue was purified by flash chromatography (pentane/EA = 2:1, Rf = 0.20) to give the product as a brown oil (80.7 mg, 85%).

^{1}H NMR (300 MHz, CDCl$_3$) δ 8.03 – 7.97 (m, 2H), 7.47 – 7.41 (m, 2H), 7.32 (d, *J* = 4.2 Hz, 5H), 7.26 – 7.20 (m, 1H), 3.71 (br, 2H), 3.29 (br, 2H), 3.18 – 3.09 (m, 2H), 2.87 – 2.75 (m, 2H), 1.68 – 1.52 (m, 6H), 1.31 (s, 12H).

^{13}C NMR (75 MHz, CDCl$_3$) δ 199.4, 169.2, 143.4, 140.6, 137.4, 137.3, 128.8, 128.4, 128.3, 127.3, 126.9, 83.6, 48.6, 39.0, 25.6, 24.8, 24.8, 24.5.

^{11}B NMR (96 MHz, CDCl$_3$) δ 29.7.

HRMS (ESI): calcd for $[M+H]^+$ $C_{29}H_{36}BNO_4$ 473.2852, found: 473.2861.

IR (ATR): 2934, 1682, 1618, 1371, 1350, 1310, 1272, 1141, 751, 699 cm^{-1}.

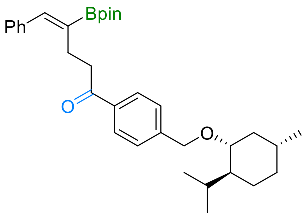

(*Z*)-1-(4-((((1R,2S,5R)-2-Isopropyl-5-methylcyclohexyl)oxy)methyl)phenyl)-5-phenyl-4-(4,4,5,5-tetramethyl-1,3,2-dioxaborolan-2-yl)pent-4-en-1-one (3x)

The title compound was prepared from (cyclopropylidenemethyl)benzene (27 uL, 0.2 mmol) and 1-iodo-4-((((1R,2S,5R)-2-isopropyl-5-methylcyclohexyl)oxy)methyl)benzene (126.4 mg, 0.34 mmol), according to general borocarbonylation I (ring open). The crude residue was purified by flash chromatography (pentane/EA = 20:1, Rf = 0.30) to give the product as a colorless oil (65.0 mg, 61%).

^{1}H NMR (300 MHz, CDCl$_3$) δ 8.01 – 7.90 (m, 2H), 7.46 – 7.37 (m, 2H), 7.37 – 7.28 (m, 5H), 7.27 – 7.20 (m, 1H), 4.71 (d, *J* = 12.4 Hz, 1H), 4.45 (d, *J* = 12.4 Hz, 1H), 3.27 – 3.07 (m, 3H), 2.81 (dd, *J* = 9.8, 6.9 Hz, 2H), 2.30 (pd, *J* = 7.0, 2.7 Hz, 1H), 2.24 – 2.13 (m, 1H), 1.72 – 1.58 (m, 2H), 1.32 (s, 14H), 0.97 – 0.87 (m, 9H), 0.74 (d, *J* = 7.0 Hz, 3H).

^{13}C NMR (75 MHz, CDCl$_3$) δ 199.7, 144.4, 143.2, 137.4, 135.9, 128.9, 128.3, 128.2, 127.4, 127.3, 83.5, 79.2, 69.7, 48.3, 40.2, 38.9, 34.5, 31.5, 25.6, 24.8, 23.2, 22.3, 21.0, 16.1.

^{11}B NMR (96 MHz, CDCl$_3$) δ 29.9.

HRMS (ESI): calcd for [M+Na]$^+$ $C_{34}H_{47}BO_4$ 552.3500, found: 552.3511.

IR (ATR): 2952, 2920, 1681, 1370, 1351, 1309, 1269, 1142, 1107, 1084, 962, 751, 698 cm^{-1}.

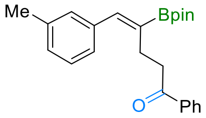

(*Z*)-1-Phenyl-4-(4,4,5,5-tetramethyl-1,3,2-dioxaborolan-2-yl)-5-(*o*-tolyl)pent-4-en-1-one (3y)

The title compound was prepared from 1-(cyclopropylidenemethyl)-3-methylbenzene (32 uL, 0.2 mmol) and iodobenzene (39 uL, 0.34 mmol), according to general borocarbonylation I (ring open). The crude residue was purified by flash chromatography (pentane/EA = 20:1, Rf = 0.35) to give the product as a colorless oil (46.9 mg, 62%).

^{1}H NMR (300 MHz, CDCl$_3$) δ 8.06 – 7.89 (m, 2H), 7.58 – 7.50 (m, 1H), 7.48 – 7.39 (m, 2H), 7.29 (d, *J* = 1.1 Hz, 1H), 7.22 (dd, *J* = 8.2, 7.3 Hz, 1H), 7.17 – 7.11 (m, 2H), 7.09 – 7.02 (m, 1H), 3.20 – 3.08 (m, 2H), 2.81 (dd, *J* = 7.7, 6.7 Hz, 2H), 2.32 (s, 3H), 1.32 (s, 12H).

^{13}C NMR (75 MHz, CDCl$_3$) δ 200.1, 143.4, 137.7, 137.4, 136.9, 132.8, 129.7, 128.4, 128.2, 128.1, 128.1, 125.9, 83.5, 38.9, 24.8, 24.8, 21.4.

^{11}B NMR (96 MHz, CDCl$_3$) δ 30.1.

HRMS (ESI): calcd for [M+Na]$^+$ $C_{24}H_{29}BO_3$ 398.2143, found: 398.2149.

IR (ATR): 2974, 1682, 1371, 1340, 1309, 1267, 1141, 1079, 963, 743, 687 cm^{-1}.

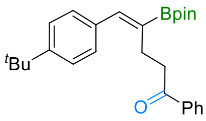

(*Z*)-5-(4-(*tert*-Butyl)phenyl)-1-phenyl-4-(4,4,5,5-tetramethyl-1,3,2-dioxaborolan-2-yl)pent-4-en-1-one (3z)

The title compound was prepared from 1-(tert-butyl)-4-(cyclopropylidenemethyl)benzene (40 uL, 0.2 mmol) and iodobenzene (39 uL, 0.34 mmol), according to general borocarbonylation I (ring open). The crude residue was purified by flash chromatography (pentane/EA = 20:1, Rf = 0.35) to give the product as a white solid (42.7 mg, 51%).

^{1}H NMR (300 MHz, CDCl$_3$) δ 8.06 – 7.95 (m, 2H), 7.58 – 7.51 (m, 1H), 7.48 – 7.41 (m, 2H), 7.35 (d, *J* = 8.1 Hz, 2H), 7.32 – 7.26 (m, 3H), 3.25 – 3.11 (m, 2H), 2.92 – 2.76 (m, 2H), 1.32 (s, 12H), 1.31 (s, 9H).

^{13}C NMR (75 MHz, CDCl$_3$) δ 200.2, 150.3, 143.1, 136.9, 134.5, 132.8, 128.8, 128.4, 128.2, 125.2, 83.5, 38.9, 34.5, 31.3, 24.9, 24.8.

^{11}B NMR (96 MHz, CDCl$_3$) δ 30.1.

HRMS (ESI): calcd for [M+Na]$^+$ $C_{27}H_{35}BO_3$ 440.2612, found: 440.2613.

IR (ATR): 1684, 1378, 1335, 1319, 1280, 1204, 1139, 1078, 746, 686 cm^{-1}.

Mp: 147-149 ºC

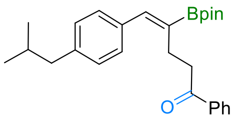

(*Z*)-5-(4-Isobutylphenyl)-1-phenyl-4-(4,4,5,5-tetramethyl-1,3,2-dioxaborolan-2-yl)pent-4-en-1-one (3aa)

The title compound was prepared from 1-(cyclopropylidenemethyl)-4-isobutylbenzene (37.2 mg, 0.2 mmol) and iodobenzene (39 uL, 0.34 mmol), according to general borocarbonylation I (ring open). The crude residue was purified by flash chromatography (pentane/EA = 20:1, Rf = 0.35) to give the product as a colorless oil (46.0 mg, 55%).

^{1}H NMR (300 MHz, CDCl$_3$) δ 7.93 – 7.86 (m, 2H), 7.47 – 7.39 (m, 1H), 7.37 – 7.29 (m, 2H), 7.19 – 7.15 (m, 2H), 7.14 (d, *J* = 1.7 Hz, 1H), 7.02 – 6.96 (m, 2H), 3.11 – 2.99 (m, 2H), 2.83 – 2.63 (m, 2H), 2.34 (d, *J* = 7.2 Hz, 2H), 1.74 (hept, *J* = 6.6 Hz, 1H), 1.20 (s, 12H), 0.78 (d, *J* = 6.6 Hz, 6H).

^{13}C NMR (75 MHz, CDCl$_3$) δ 200.1, 143.3, 141.0, 136.9, 134.8, 132.8, 129.0, 128.8, 128.4, 128.2, 83.5, 45.2, 38.8, 30.1, 24.8, 22.4.

^{11}B NMR (96 MHz, CDCl$_3$) δ 30.3.

HRMS (ESI): calcd for [M+Na]$^+$ $C_{27}H_{35}BO_3$ 440.2612, found: 440.2611.

IR (ATR): 2953, 1681, 1371, 1346, 1309, 1268, 1141, 688 cm^{-1}.

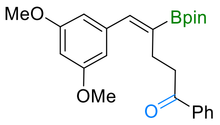

(*Z*)-5-(3,5-Dimethoxyphenyl)-1-phenyl-4-(4,4,5,5-tetramethyl-1,3,2-dioxaborolan-2-yl)pent-4-en-1-one (3bb)

The title compound was prepared from 1-(cyclopropylidenemethyl)-3,5-dimethoxybenzene (38 uL, 0.2 mmol) and iodobenzene (39 uL, 0.34 mmol), according to general borocarbonylation I (ring open). The crude residue was purified by flash chromatography (pentane/EA = 20:1, Rf = 0.15) to give the product as a yellow oil (73.2 mg, 87%).

^{1}H NMR (300 MHz, $CDCl_3$) δ 8.05 – 7.92 (m, 2H), 7.59 – 7.49 (m, 1H), 7.48 – 7.39 (m, 2H), 7.24 (s, 1H), 6.49 (dd, *J* = 2.3, 0.7 Hz, 2H), 6.37 (t, *J* = 2.3 Hz, 1H), 3.76 (s, 6H), 3.25 – 3.06 (m, 2H), 2.91 – 2.73 (m, 2H), 1.32 (s, 12H).

^{13}C NMR (75 MHz, $CDCl_3$) δ 200.0, 160.5, 143.2, 139.3, 136.8, 132.8, 128.4, 128.2, 106.7, 99.9, 83.6, 55.3, 38.9, 25.1, 24.8.

^{11}B NMR (128 MHz, $CDCl_3$) δ 30.8.

HRMS (ESI): calcd for $[M+Na]^+$ $C_{25}H_{31}BO_5$ 444.2198, found: 444.2204.

IR (ATR): 1680, 1588, 1449, 1371, 1290, 1203, 1140, 1060, 967, 832, 747, 686 cm^{-1}.

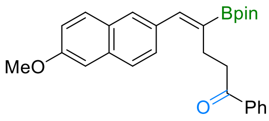

(*Z*)-5-(6-Methoxynaphthalen-2-yl)-1-phenyl-4-(4,4,5,5-tetramethyl-1,3,2-dioxaborolan-2-yl)pent-4-en-1-one (3cc)

The title compound was prepared from 2-(cyclopropylidenemethyl)-6-methoxynaphthalene (42.0 mg, 0.2 mmol) and iodobenzene (39 uL, 0.34 mmol), according to general borocarbonylation I (ring open). The crude residue was purified by flash chromatography (pentane/EA = 20:1, Rf = 0.15) to give the product as a colorless oil (55.1 mg, 62%).

^{1}H NMR (400 MHz, $CDCl_3$) δ 7.99 (dd, *J* = 8.4, 1.3 Hz, 2H), 7.75 (s, 1H), 7.69 (dd, *J* = 8.8, 3.8 Hz, 2H), 7.56 – 7.50 (m, 1H), 7.48 – 7.38 (m, 4H), 7.16 – 7.08 (m, 2H), 3.92 (s, 3H), 3.24 – 3.14 (m, 2H), 2.97 – 2.87 (m, 2H), 1.34 (s, 12H).

^{13}C NMR (101 MHz, $CDCl_3$) δ 200.1, 157.9, 143.3, 136.8, 133.7, 132.8, 132.8, 129.8, 128.7, 128.4, 128.2, 128.1, 127.5, 126.6, 118.9, 105.5, 83.5, 55.3, 38.8, 24.9, 24.8.

^{11}B NMR (128 MHz, $CDCl_3$) δ 30.5.

HRMS (ESI): calcd for $[M+Na]^+$ $C_{28}H_{31}BO_4$ 464.2249, found: 464.2252.

IR (ATR): 1680, 1595, 1370, 1352, 1306, 1263, 1140, 849, 687 cm^{-1}.

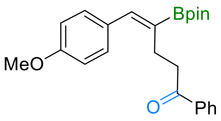

(*Z*)-5-(4-Methoxyphenyl)-1-phenyl-4-(4,4,5,5-tetramethyl-1,3,2-dioxaborolan-2-yl)pent-4-en-1-one (3dd)

The title compound was prepared from 1-(cyclopropylidenemethyl)-4-methoxybenzene (28 uL, 0.2 mmol) and iodobenzene (39 uL, 0.34 mmol), according to general borocarbonylation I (ring open). The crude residue was purified by flash chromatography (pentane/EA = 20:1, Rf = 0.20) to give the product as a white solid (60.6 mg, 77%).

^{1}H NMR (300 MHz, $CDCl_3$) δ 8.04 – 7.97 (m, 2H), 7.58 – 7.50 (m, 1H), 7.48 – 7.40 (m, 2H), 7.35 – 7.28 (m, 2H), 7.24 (s, 1H), 6.90 – 6.83 (m, 2H), 3.80 (s, 3H), 3.20 – 3.12 (m, 2H), 2.90 – 2.78 (m, 2H), 1.31 (s, 12H).

^{13}C NMR (75 MHz, $CDCl_3$) δ 200.1, 158.8, 142.8, 136.9, 132.8, 130.5, 130.1, 128.4, 128.2, 113.7, 83.4, 55.2, 38.8, 24.8, 24.7.

^{11}B NMR (96 MHz, $CDCl_3$) δ 30.2.

HRMS (EI): calcd for [M] $C_{24}H_{29}BO_4$ 392.21534, found: 392.21632.

IR (ATR): 1681, 1602, 1508, 1347, 1248, 1175, 1140, 688 cm^{-1}.

Mp: 62-63 ºC

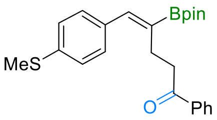

(*Z*)-5-(4-(Methylthio)phenyl)-1-phenyl-4-(4,4,5,5-tetramethyl-1,3,2-dioxaborolan-2-yl)pent-4-en-1-one (3ee)

The title compound was prepared from (4-(cyclopropylidenemethyl)phenyl)(methyl)sulfane (35.2 mg, 0.2 mmol) and iodobenzene (39 uL, 0.34 mmol), according to general borocarbonylation I (ring open). The crude residue was purified by flash chromatography (pentane/EA = 20:1, Rf = 0.20) to give the product as a yellow oil (74.5 mg, 91%).

^{1}H NMR (400 MHz, $CDCl_3$) δ 8.02 – 7.93 (m, 2H), 7.57 – 7.51 (m, 1H), 7.44 (tt, *J* = 6.7, 1.4 Hz, 2H), 7.30 – 7.26 (m, 2H), 7.24 (s, 1H), 7.22 – 7.17 (m, 2H), 3.20 – 3.11 (m, 2H), 2.87 – 2.77 (m, 2H), 2.47 (s, 3H), 1.31 (s, 12H).

^{13}C NMR (101 MHz, $CDCl_3$) δ 200.0, 142.5, 137.7, 136.8, 134.1, 132.8, 129.4, 128.4, 128.2, 126.1, 83.5, 38.7, 24.8, 24.7, 15.5.

^{11}B NMR (128 MHz, $CDCl_3$) δ 30.6.

HRMS (ESI): calcd for [M+Na]$^+$ $C_{24}H_{29}BO_3S$ 430.1864, found: 430.1861.

IR (ATR): 1685, 1371, 1309, 1140, 1076, 964, 742, 685, 502 cm^{-1}.

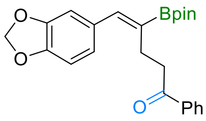

(*Z*)-5-(Benzo[d][1,3]dioxol-5-yl)-1-phenyl-4-(4,4,5,5-tetramethyl-1,3,2-dioxaborolan-2-yl)pent-4-en-1-one (3ff)

The title compound was prepared from 5-(cyclopropylidenemethyl)benzo[d][1,3]dioxole (34.8 mg, 0.2 mmol) and iodobenzene (39 uL, 0.34 mmol), according to general borocarbonylation I (ring open). The crude residue was purified by flash chromatography (pentane/EA = 20:1, Rf = 0.20) to give the product as a colorless oil (62.9mg, 77%).

^{1}H NMR (300 MHz, $CDCl_3$) δ 8.06 – 7.93 (m, 2H), 7.60 – 7.51 (m, 1H), 7.48 – 7.40 (m, 2H), 7.20 (s, 1H), 6.89 – 6.73 (m, 3H), 5.94 (s, 2H), 3.23 – 3.06 (m, 2H), 2.87 – 2.75 (m, 2H), 1.30 (s, 12H).

^{13}C NMR (75 MHz, $CDCl_3$) δ 200.0, 147.5, 146.8, 142.8, 136.9, 132.8, 131.6, 128.4, 128.2, 123.1, 109.1, 108.2, 101.0, 83.5, 38.7, 24.8, 24.7.

^{11}B NMR (96 MHz, $CDCl_3$) δ 30.1.

HRMS (ESI): calcd for $[M+Na]^+$ $C_{24}H_{27}BO_5$ 428.1885, found: 428.1887.

IR (ATR): 1681, 1487, 1444, 1358, 1307, 1235, 1203, 1141, 1101, 1036, 926, 744, 688 cm^{-1}.

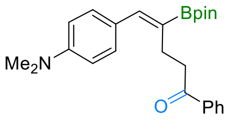

(*Z*)-5-(4-(dimethylamino)phenyl)-1-phenyl-4-(4,4,5,5-tetramethyl-1,3,2-dioxaborolan-2-yl)pent-4-en-1-one (3gg)

The title compound was prepared from 4-(cyclopropylidenemethyl)-*N*,*N*-dimethylaniline (34.6 mg, 0.2 mmol) and iodobenzene (39 uL, 0.34 mmol), according to general borocarbonylation I (ring open). The crude residue was purified by flash chromatography (pentane/EA = 10:1, Rf = 0.30) to give the product as a yellow oil (32.4 mg, 40%).

^{1}H NMR (300 MHz, $CDCl_3$) δ 8.13 – 7.96 (m, 2H), 7.61 – 7.49 (m, 1H), 7.49 – 7.41 (m, 2H), 7.36 – 7.30 (m, 2H), 7.20 (s, 1H), 6.68 (d, J = 9.0 Hz, 2H), 3.23 – 3.12 (m, 2H), 2.96 (s, 6H), 2.91 – 2.84 (m, 2H), 1.31 (s, 12H).

^{13}C NMR (101 MHz, $CDCl_3$) δ 200.4, 149.6, 143.3, 137.0, 132.7, 130.6, 128.5, 128.4, 128.3, 111.9, 83.3, 40.3, 38.8, 24.9, 24.8.

^{11}B NMR (128 MHz, $CDCl_3$) δ 30.6.

HRMS (ESI): calcd for $[M+Na]^+$ $C_{25}H_{32}BNO_3$ 427.2408, found: 427.2414.

IR (ATR): 1682, 1595, 1519, 1348, 1163, 1141, 946, 815, 747, 689 cm^{-1}.

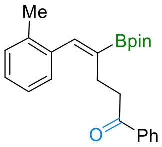

(*Z*)-1-Phenyl-4-(4,4,5,5-tetramethyl-1,3,2-dioxaborolan-2-yl)-5-(o-tolyl)pent-4-en-1-one (3hh)

The title compound was prepared from 1-(cyclopropylidenemethyl)-2-methylbenzene (32 uL, 0.2 mmol) and iodobenzene (39 uL, 0.34 mmol), according to general borocarbonylation I (ring open). The crude residue was purified by flash chromatography (pentane/EA = 20:1, Rf = 0.33) to give the product as a colorless oil (49.2 mg, 66%).

^{1}H NMR (300 MHz, $CDCl_3$) δ 7.98 – 7.85 (m, 2H), 7.56 – 7.48 (m, 1H), 7.44 – 7.37 (m, 2H), 7.35 (s, 1H), 7.20 – 7.10 (m, 4H), 3.11 – 2.98 (m, 2H), 2.70 – 2.56 (m, 2H), 2.26 (s, 3H), 1.33 (s, 12H).

^{13}C NMR (75 MHz, $CDCl_3$) δ 200.0, 143.3, 137.0, 136.8, 136.0, 132.7, 129.8, 128.5, 128.4, 128.2, 127.2, 125.4, 83.5, 39.0, 25.0, 24.8, 19.9.

^{11}B NMR (96 MHz, $CDCl_3$) δ 30.3.

HRMS (ESI): calcd for $[M+Na]^+$ $C_{24}H_{29}BO_3$ 398.2143, found: 398.2147.

IR (ATR): 1683, 1370, 1330, 1312, 1284, 1139, 1080, 744, 684 cm^{-1}.

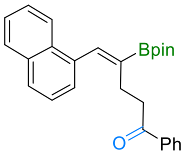

(*Z*)-5-(Naphthalen-1-yl)-1-phenyl-4-(4,4,5,5-tetramethyl-1,3,2-dioxaborolan-2-yl)pent-4-en-1-one (3ii)

The title compound was prepared from 1-(cyclopropylidenemethyl)naphthalene (36.0 mg, 0.2 mmol) and iodobenzene (39 uL, 0.34 mmol), according to general borocarbonylation I (ring open). The crude residue was purified by flash chromatography (pentane/EA = 20:1, Rf = 0.20) to give the product as a colorless oil (32.0 mg, 39%).

^{1}H NMR (300 MHz, $CDCl_3$) δ 8.00 – 7.94 (m, 1H), 7.88 – 7.75 (m, 5H), 7.52 – 7.45 (m, 3H), 7.42 (d, J = 8.5 Hz, 1H), 7.37 – 7.30 (m, 3H), 3.11 – 2.97 (m, 2H), 2.71 – 2.54 (m, 2H), 1.37 (s, 12H).

^{13}C NMR (75 MHz, $CDCl_3$) δ 200.0, 142.2, 136.7, 135.0, 133.5, 132.6, 131.5, 128.3, 128.3, 128.1, 127.6, 125.9, 125.8, 125.8, 125.3, 125.2, 83.6, 39.1, 25.4, 24.9.

^{11}B NMR (96 MHz, $CDCl_3$) δ 29.9.

HRMS (ESI): calcd for $[M+Na]^+$ $C_{27}H_{29}BO_3$ 434.2143, found: 434.2145.

IR (ATR): 1679, 1370, 1350, 1309, 1263, 1139, 781, 741, 687 cm^{-1}.

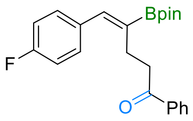

(*Z*)-5-(4-Fuorophenyl)-1-phenyl-4-(4,4,5,5-tetramethyl-1,3,2-dioxaborolan-2-yl)pent-4-en-1-one (3jj)

The title compound was prepared from 1-(cyclopropylidenemethyl)-4-fluorobenzene (28 uL, 0.2 mmol) and iodobenzene (39 uL, 0.34 mmol), according to general borocarbonylation I (ring open). The crude residue was purified by flash chromatography (pentane/EA = 20:1, Rf = 0.35) to give the product as a colorless oil (64.1 mg, 84%).

^{1}H NMR (300 MHz, $CDCl_3$) δ 8.06 – 7.90 (m, 2H), 7.59 – 7.51 (m, 1H), 7.49 – 7.40 (m, 2H), 7.34 – 7.24 (m, 3H), 7.07 – 6.95 (m, 2H), 3.25 – 3.08 (m, 2H), 2.79 (dd, *J* = 8.8, 6.4 Hz, 2H), 1.31 (s, 12H).

^{13}C NMR (75 MHz, $CDCl_3$) δ 199.9, 161.9 (d, *J* = 247.1 Hz), 142.0, 136.8, 133.5 (d, *J* = 3.3 Hz), 132.9, 130.6 (d, *J* = 8.1 Hz), 128.5, 128.2, 115.2 (d, *J* = 21.2 Hz), 83.6, 38.6, 24.8, 24.6.

^{11}B NMR (128 MHz, $CDCl_3$) δ 30.5.

^{19}F NMR (282 MHz, $CDCl_3$) δ -114.31 (ddd, *J* = 14.5, 9.2, 5.3 Hz).

HRMS (ESI): calcd for $[M+Na]^+$ $C_{23}H_{26}BFO_3$ 402.1892, found: 402.1896.

IR (ATR): 1686, 1505, 1344, 1312, 1203, 1140, 1024, 742, 687, 524 cm^{-1}.

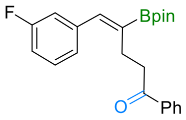

(*Z*)-5-(3-Fluorophenyl)-1-phenyl-4-(4,4,5,5-tetramethyl-1,3,2-dioxaborolan-2-yl)pent-4-en-1-one (3kk)

The title compound was prepared from 1-(cyclopropylidenemethyl)-3-fluorobenzene (28 uL, 0.2 mmol) and iodobenzene (39 uL, 0.34 mmol), according to general borocarbonylation I (ring open). The crude residue was purified by flash chromatography (pentane/EA = 20:1, Rf = 0.35) to give the product as a colorless oil (53.5 mg, 70%).

^{1}H NMR (300 MHz, $CDCl_3$) δ 7.85 – 7.70 (m, 2H), 7.39 – 7.30 (m, 1H), 7.24 (ddt, *J* = 8.3, 6.8, 1.2 Hz, 2H), 7.13 – 7.03 (m, 2H), 6.90 (ddd, *J* = 8.4, 1.7, 0.9 Hz, 1H), 6.85 – 6.78 (m, 1H), 6.77 – 6.69 (m, 1H), 3.00 – 2.91 (m, 2H), 2.65 – 2.55 (m, 2H), 1.12 (s, 12H).

^{13}C NMR (75 MHz, $CDCl_3$) δ 199.8, 162.63 (d, *J* = 245.4 Hz), 141.8 (d, *J* = 2.4 Hz), 139.7 (d, *J* = 7.4 Hz), 136.8, 132.8, 129.7 (d, *J* = 8.4 Hz), 128.5, 128.2, 124.5 (d, *J* = 2.7 Hz), 115.6 (d, *J* = 21.5 Hz), 114.1 (d, *J* = 21.2 Hz), 83.7, 38.6, 24.8, 24.7.

^{11}B NMR (96 MHz, $CDCl_3$) δ 30.0.

HRMS (ESI): calcd for $[M+Na]^+$ $C_{23}H_{26}BFO_3$ 402.1892, found: 402.1901.

IR (ATR): 2976, 1682, 1579, 1371, 1341, 1313, 1270, 1248, 1204, 1139, 1081, 964, 784, 743, 687 cm^{-1}.

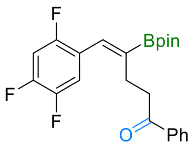

(Z)-1-Phenyl-4-(4,4,5,5-tetramethyl-1,3,2-dioxaborolan-2-yl)-5-(2,4,5-trifluorophenyl)pent-4-en-1-one (3ll)

The title compound was prepared from 1-(cyclopropylidenemethyl)-2,4,5-trifluorobenzene (36.8 mg, 0.2 mmol) and iodobenzene (39 uL, 0.34 mmol), according to general borocarbonylation I (ring open). The crude residue was purified by flash chromatography (pentane/EA = 20:1, Rf = 0.35) to give the product as a colorless oil (58.0 mg, 70%).

^{1}H NMR (400 MHz, $CDCl_3$) δ 7.99 – 7.93 (m, 2H), 7.57 – 7.52 (m, 1H), 7.47 – 7.41 (m, 2H), 7.16 – 7.05 (m, 2H), 6.90 (ddd, *J* = 10.3, 9.1, 6.7 Hz, 1H), 3.18 – 3.10 (m, 2H), 2.71 – 2.60 (m, 2H), 1.31 (s, 12H).

^{13}C NMR (101 MHz, $CDCl_3$) δ 199.7, 154.9 (ddd, *J* = 246.7, 9.4, 2.0 Hz), 149.4 (ddd, *J* = 251.8, 14.4, 12.6 Hz), 146.4 (ddd, *J* = 245.0, 12.5, 3.9 Hz), 136.8, 133.8, 132.9, 128.5, 128.1, 121.5 (d, *J* = 18.0 Hz), 117.9 (dd, *J* = 19.3, 4.9 Hz), 105.6 (dd, *J* = 28.7, 20.6 Hz), 83.9, 38.1, 25.2, 24.8.

^{11}B NMR (128 MHz, $CDCl_3$) δ 30.5.

HRMS (ESI): calcd for $[M+Na]^+$ $C_{23}H_{24}BF_3O_3$ 438.1704, found: 438.1712.

IR (ATR): 1683, 1504, 1372, 1312, 1268, 1200, 1141, 1078, 843, 744, 688 cm^{-1}.

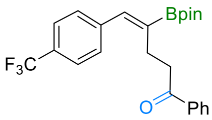

(Z)-1-Phenyl-4-(4,4,5,5-tetramethyl-1,3,2-dioxaborolan-2-yl)-5-(4-(trifluoromethyl)phenyl)pent-4-en-1-one (3mm)

The title compound was prepared from 1-(cyclopropylidenemethyl)-4-(trifluoromethyl)benzene (35 uL, 0.2 mmol) and iodobenzene (39 uL, 0.34 mmol), according to general borocarbonylation I (ring open). The crude residue was purified by flash chromatography (pentane/EA = 20:1, Rf = 0.35) to give the product as a colorless oil (54.0 mg, 63%).

^{1}H NMR (400 MHz, $CDCl_3$) δ 7.99 – 7.93 (m, 2H), 7.60 – 7.52 (m, 3H), 7.46 – 7.39 (m, 4H), 7.31 (s, 1H), 3.20 – 3.10 (m, 2H), 2.86 – 2.75 (m, 2H), 1.32 (s, 12H).

^{13}C NMR (101 MHz, $CDCl_3$) δ 199.7, 141.6, 141.0, 136.7, 132.9, 129.1 (q, *J* = 32.3 Hz), 129.0, 128.5, 128.1, 125.2 (q, *J* = 4.0 Hz), 124.1 (q, *J* = 272.0 Hz), 83.8, 38.5, 24.8, 24.7.

^{11}B NMR (128 MHz, $CDCl_3$) δ 30.6.

^{19}F NMR (376 MHz, $CDCl_3$) δ -62.5.

HRMS (EI): calcd for [M] $C_{24}H_{26}BF_3O_3$ 430.19216, found: 430.19251.

IR (ATR): 1683, 1372, 1320, 1268, 1163, 1141, 1121, 1065, 1016, 962, 833, 743, 688, 596 cm^{-1}.

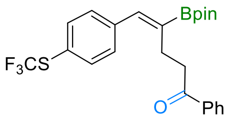

(*Z*)-1-Phenyl-4-(4,4,5,5-tetramethyl-1,3,2-dioxaborolan-2-yl)-5-(4-((trifluoromethyl)thio)phenyl)pent-4-en-1-one (3nn)

The title compound was prepared from (4-(cyclopropylidenemethyl)phenyl)(trifluoromethyl)sulfane (46.0 mg, 0.2 mmol) and iodobenzene (39 uL, 0.34 mmol), according to general borocarbonylation I (ring open). The crude residue was purified by flash chromatography (pentane/EA = 20:1, Rf = 0.35) to give the product as a colorless oil (60.1 mg, 65%).

^{1}H NMR (400 MHz, $CDCl_3$) δ 8.00 – 7.94 (m, 2H), 7.60 (d, *J* = 8.2 Hz, 2H), 7.57 – 7.52 (m, 1H), 7.44 (tt, *J* = 7.7, 1.8 Hz, 2H), 7.38 – 7.33 (m, 2H), 7.28 (s, 1H), 3.20 – 3.12 (m, 2H), 2.80 (dd, *J* = 8.9, 7.0 Hz, 2H), 1.32 (s, 12H).

^{13}C NMR (101 MHz, $CDCl_3$) δ 199.7, 141.5, 140.1, 136.7, 136.1, 132.9, 129.8, 129.5 (q, *J* = 308.3 Hz), 128.5, 128.1, 122.9 (q, *J* = 2.2 Hz), 83.8, 38.5, 24.8, 24.7.

^{11}B NMR (128 MHz, $CDCl_3$) δ 30.8.

^{19}F NMR (376 MHz, $CDCl_3$) δ -42.6.

HRMS (EI): calcd for [M] $C_{24}H_{26}BF_3O_3S$ 462.16423, found: 462.16553.

IR (ATR): 1683, 1372, 1342, 1314, 1266, 1112, 1083, 962, 744, 688 cm^{-1}.

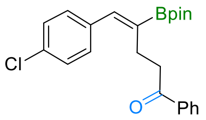

(*Z*)-5-(4-Chlorophenyl)-1-phenyl-4-(4,4,5,5-tetramethyl-1,3,2-dioxaborolan-2-yl)pent-4-en-1-one (3oo)

The title compound was prepared from 1-chloro-4-(cyclopropylidenemethyl)benzene (32.8 mg, 0.2 mmol) and iodobenzene (39 uL, 0.34 mmol), according to general borocarbonylation I (ring open). The crude residue was purified by flash chromatography (pentane/EA = 20:1, Rf = 0.35) to give the product as a colorless oil (58.4 mg, 74%).

^{1}H NMR (300 MHz, $CDCl_3$) δ 7.90 – 7.75 (m, 2H), 7.44 – 7.37 (m, 1H), 7.33 – 7.26 (m, 2H), 7.17 – 7.08 (m, 5H), 3.05 – 2.92 (m, 2H), 2.71 – 2.58 (m, 2H), 1.16 (s, 12H).

^{13}C NMR (75 MHz, $CDCl_3$) δ 199.8, 141.8, 136.8, 135.8, 133.1, 132.9, 130.2, 128.5, 128.4, 128.2, 83.7, 38.6, 24.8, 24.6.

^{11}B NMR (96 MHz, $CDCl_3$) δ 30.2.

HRMS (ESI): calcd for $[M+Na]^+$ $C_{23}H_{26}BClO_3$ 418.1597, found: 418.1600.

IR (ATR): 2974, 1683, 1370, 1339, 1314, 1281, 1137, 1091, 1076, 812, 746, 686, 501 cm^{-1}.

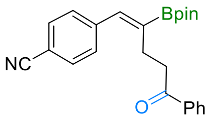

(*Z*)-4-(5-Oxo-5-phenyl-2-(4,4,5,5-tetramethyl-1,3,2-dioxaborolan-2-yl)pent-1-en-1-yl)benzonitrile (3pp)

The title compound was prepared from 4-(cyclopropylidenemethyl)benzonitrile (31.0 mg, 0.2 mmol) and iodobenzene (39 uL, 0.34 mmol), according to general borocarbonylation I (ring open). The crude residue was purified by flash chromatography (pentane/EA = 10:1, Rf = 0.20) to give the product as a white solid (37.4 mg, 48%).

^{1}H NMR (300 MHz, CDCl$_3$) δ 8.04 – 7.89 (m, 2H), 7.64 – 7.57 (m, 2H), 7.57 – 7.51 (m, 1H), 7.47 – 7.37 (m, 4H), 7.26 (d, *J* = 1.7 Hz, 1H), 3.21 – 3.07 (m, 2H), 2.83 – 2.71 (m, 2H), 1.31 (s, 12H).

^{13}C NMR (75 MHz, CDCl$_3$) δ 199.5, 142.1, 141.0, 136.7, 133.0, 132.0, 129.4, 128.5, 128.1, 118.9, 110.7, 83.9, 38.3, 24.8, 24.7.

^{11}B NMR (96 MHz, CDCl$_3$) δ 30.0.

^{19}F NMR (376 MHz, CDCl$_3$) δ -115.16 (ddt, *J* = 15.0, 10.9, 4.8 Hz), -134.23 (dtd, *J* = 22.5, 9.5, 4.4 Hz), -142.80 (dddd, *J* = 21.8, 14.3, 10.9, 6.8 Hz).

HRMS (ESI): calcd for $[M+H]^+$ $C_{24}H_{26}BNO_3$ 387.2120, found: 387.2122.

IR (ATR): 2222, 1678, 1088, 853, 745, 688, 553 cm^{-1}.

decomposition: 177 °C

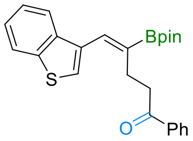

(*Z*)-5-(Benzo[b]thiophen-3-yl)-1-phenyl-4-(4,4,5,5-tetramethyl-1,3,2-dioxaborolan-2-yl)pent-4-en-1-one (3qq)

The title compound was prepared from 3-(cyclopropylidenemethyl)benzo[b]thiophene (37.2 mg, 0.2 mmol) and iodobenzene (39 uL, 0.34 mmol), according to general borocarbonylation I (ring open). The crude residue was purified by flash chromatography (pentane/EA = 20:1, Rf = 0.20) to give the product as a colorless oil (76.1 mg, 90%).

^{1}H NMR (400 MHz, CDCl$_3$) δ 7.96 (dd, *J* = 8.4, 1.3 Hz, 2H), 7.90 – 7.81 (m, 2H), 7.57 – 7.51 (m, 1H), 7.48 (d, *J* = 3.1 Hz, 2H), 7.45 – 7.33 (m, 4H), 3.29 – 3.09 (m, 2H), 2.97 – 2.79 (m, 2H), 1.34 (s, 12H).

^{13}C NMR (101 MHz, CDCl$_3$) δ 200.0, 139.5, 139.0, 136.8, 134.8, 132.8, 132.5, 128.4, 128.2, 124.6, 124.4, 124.1, 122.5, 122.3, 83.6, 38.6, 25.8, 24.8.

^{11}B NMR (128 MHz, CDCl$_3$) δ 30.9.

HRMS (ESI): calcd for $[M+Na]^+$ $C_{25}H_{27}BO_3S$ 440.1707, found: 440.1707.

IR (ATR): 1687, 1355, 1315, 1136, 760, 744, 732, 688, 678, 667 cm^{-1}.

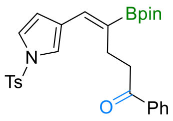

(*Z*)-1-Phenyl-4-(4,4,5,5-tetramethyl-1,3,2-dioxaborolan-2-yl)-5-(1-tosyl-1H-pyrrol-3-yl)pent-4-en-1-one (3rr)

The title compound was prepared from 3-(cyclopropylidenemethyl)-1-tosyl-1*H*-pyrrole (54.6 mg, 0.2 mmol) and iodobenzene (39 uL, 0.34 mmol), according to general borocarbonylation I (ring open). The crude residue was purified by flash chromatography (pentane/EA = 10:1, Rf = 0.30) to give the product as a yellow oil (94.8 mg, 94%).

^{1}H NMR (300 MHz, CDCl$_3$) δ 8.00 – 7.87 (m, 2H), 7.80 – 7.66 (m, 2H), 7.60 – 7.48 (m, 2H), 7.48 – 7.36 (m, 3H), 7.23 (dd, *J* = 8.7, 0.7 Hz, 2H), 6.43 – 6.31 (m, 1H), 6.25 (td, *J* = 3.4, 0.6 Hz, 1H), 2.99 – 2.88 (m, 2H), 2.66 – 2.54 (m, 2H), 2.35 (s, 3H), 1.31 (s, 12H).

^{13}C NMR (75 MHz, CDCl$_3$) δ 199.8, 144.9, 136.7, 135.8, 132.8, 130.9, 130.6, 129.6, 128.4, 128.1, 127.5, 123.1, 116.2, 111.8, 83.5, 37.8, 25.0, 24.8, 21.6.

^{11}B NMR (96 MHz, CDCl$_3$) δ 30.6.

HRMS (EI): calcd for [M] $C_{28}H_{32}BNO_5S$ 505.20888, found: 505.20969.

IR (ATR): 1681, 1596, 1348, 1172, 1141, 1088, 733, 689, 669, 586, 542 cm^{-1}.

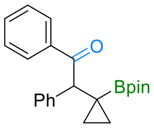

1,2-Diphenyl-2-(1-(4,4,5,5-tetramethyl-1,3,2-dioxaborolan-2-yl)cyclopropyl)ethan-1-one (4a)

The title compound was prepared from (cyclopropylidenemethyl)benzene (27 uL, 0.2 mmol) and iodobenzene (34 uL, 0.3 mmol), according to general borocarbonylation II (ring close). The crude residue was purified by flash chromatography (pentane/EA = 20:1, Rf = 0.36) to give the product as a white solid (44.9 mg, 62%). X-ray (single-crystal) colorless block crystals of X-ray diffraction quality were obtained by slow evaporation of saturated solution of **4a** in ethyl acetate/*n*-pentane (CCDC 2122869).

^{1}H NMR (300 MHz, CDCl$_3$) δ 7.77 – 7.69 (m, 2H), 7.31 – 7.24 (m, 1H), 7.21 – 7.14 (m, 2H), 7.13 – 7.08 (m, 2H), 7.04 (tt, *J* = 8.0, 1.7 Hz, 3H), 4.78 (s, 1H), 1.07 (s, 6H), 1.01 (s, 6H), 0.74 (ddd, *J* = 8.8, 5.9, 4.1 Hz, 1H), 0.40 (ddd, *J* = 8.8, 5.7, 3.7 Hz, 1H), 0.28 – 0.20 (m, 1H), 0.06 – -0.05 (m, 1H).

^{13}C NMR (75 MHz, CDCl$_3$) δ 200.6, 137.3, 136.1, 132.1, 130.1, 128.7, 128.2, 128.1, 126.9, 83.1, 56.0, 24.7, 24.1, 6.5, 6.3.

^{11}B NMR (96 MHz, CDCl$_3$) δ 33.3.

HRMS (EI): calcd for [M] $C_{23}H_{27}BO_3$ 362.20478, found: 362.20569.

IR (ATR): 2977, 1677, 1409, 1314, 1244, 1219, 1135, 961, 845, 749, 710, 695, 684, 627 cm^{-1}.

Mp: 101-102 °C

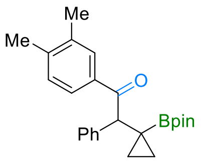

1-(3,4-Dimethylphenyl)-2-phenyl-2-(1-(4,4,5,5-tetramethyl-1,3,2-dioxaborolan-2-yl)cyclopropyl)ethan-1-one (4b)

The title compound was prepared from (cyclopropylidenemethyl)benzene (27 uL, 0.2 mmol) and 4-iodo-1,2-dimethylbenzene (42 uL, 0.3 mmol), according to general borocarbonylation II (ring close). The crude residue was purified by flash chromatography (pentane/EA = 20:1, Rf = 0.33) to give the product as a white solid (44.3 mg, 57%).

^{1}H NMR (300 MHz, CDCl$_3$) δ 7.66 (d, *J* = 1.9 Hz, 1H), 7.57 (dd, *J* = 8.0, 1.8 Hz, 1H), 7.26 – 7.19 (m, 2H), 7.19 – 7.11 (m, 3H), 7.04 (d, *J* = 8.0 Hz, 1H), 4.86 (s, 1H), 2.22 (s, 6H), 1.19 (s, 6H), 1.14 (s, 6H), 0.84 (ddd, *J* = 8.8, 5.9, 4.1 Hz, 1H), 0.51 (ddd, *J* = 8.8, 5.6, 3.7 Hz, 1H), 0.39 – 0.28 (m, 1H), 0.18 – 0.06 (m, 1H).

^{13}C NMR (75 MHz, CDCl$_3$) δ 200.3, 141.5, 136.6, 136.4, 135.1, 130.1, 130.0, 129.4, 128.2, 126.8, 126.6, 83.1, 56.0, 24.7, 24.2, 19.9, 19.7, 6.6, 6.4.

^{11}B NMR (96 MHz, CDCl$_3$) δ 32.9.

HRMS (EI): calcd for [M] $C_{25}H_{31}BO_3$ 390.23608, found: 390.23642.

IR (ATR): 1671, 1406, 1316, 1241, 1134, 977, 965, 864, 704, 670 cm^{-1}.

Mp: 107-109 ℃

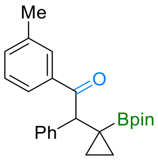

2-Phenyl-2-(1-(4,4,5,5-tetramethyl-1,3,2-dioxaborolan-2-yl)cyclopropyl)-1-(*m*-tolyl)ethan-1-one (4c)

The title compound was prepared from (cyclopropylidenemethyl)benzene (27 uL, 0.2 mmol) and 1-iodo-3-methylbenzene (39 uL, 0.3 mmol), according to general borocarbonylation II (ring close). The crude residue was purified by flash chromatography (pentane/EA = 20:1, Rf = 0.36) to give the product as a white solid (53.7 mg, 71%).

^{1}H NMR (300 MHz, CDCl$_3$) δ 7.68 (ddt, *J* = 1.8, 1.4, 0.7 Hz, 1H), 7.66 – 7.58 (m, 1H), 7.27 – 7.11 (m, 7H), 4.87 (s, 1H), 2.31 (d, *J* = 0.6 Hz, 3H), 1.19 (s, 6H), 1.14 (s, 6H), 0.84 (ddd, *J* = 8.9, 5.9, 4.2 Hz, 1H), 0.51 (ddd, *J* = 8.8, 5.6, 3.7 Hz, 1H), 0.40 – 0.28 (m, 1H), 0.17 – 0.07 (m, 1H).

^{13}C NMR (75 MHz, CDCl$_3$) δ 200.8, 137.8, 137.4, 136.3, 132.9, 130.1, 129.3, 128.2, 128.0, 126.9, 126.1, 83.2, 56.1, 24.8, 24.2, 21.3, 6.6, 6.4.

^{11}B NMR (96 MHz, CDCl$_3$) δ 33.0.

HRMS (EI): calcd for [M] $C_{24}H_{29}BO_3$ 376.22043, found: 376.22110.

IR (ATR): 2964, 2922, 1674, 1408, 1313, 1250, 1156, 1136, 964, 703, 679 cm^{-1}.

Mp: 100-101 °C

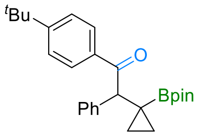

1-(4-(*tert*-Butyl)phenyl)-2-phenyl-2-(1-(4,4,5,5-tetramethyl-1,3,2-dioxaborolan-2-yl)cyclopropyl)ethan-1-one (4d)

The title compound was prepared from (cyclopropylidenemethyl)benzene (27 uL, 0.2 mmol) and 1-(tert-butyl)-4-iodobenzene (53 uL, 0.3 mmol), according to general borocarbonylation II (ring close). The crude residue was purified by flash chromatography (pentane/EA = 20:1, Rf = 0.40) to give the product as a white solid (57.0 mg, 68%).

^{1}H NMR (400 MHz, $CDCl_3$) δ 7.84 – 7.74 (m, 2H), 7.34 – 7.29 (m, 2H), 7.27 – 7.21 (m, 2H), 7.20 – 7.12 (m, 3H), 4.84 (s, 1H), 1.27 (s, 9H), 1.19 (s, 6H), 1.13 (s, 6H), 0.83 (ddd, J = 8.8, 5.9, 4.2 Hz, 1H), 0.57 – 0.46 (m, 1H), 0.37 – 0.29 (m, 1H), 0.17 – 0.08 (m, 1H).

^{13}C NMR (101 MHz, $CDCl_3$) δ 200.1, 155.6, 136.6, 134.6, 130.1, 128.8, 128.2, 126.8, 125.1, 83.1, 56.2, 34.9, 31.0, 24.7, 24.2, 6.6, 6.3.

^{11}B NMR (128 MHz, $CDCl_3$) δ 33.4.

HRMS (EI): calcd for [M] $C_{27}H_{35}BO_3$ 418.26738, found: 418.26713.

IR (ATR): 2961, 1689, 1410, 1326, 1138, 971, 962, 705, 669, 556 cm^{-1}.

Mp: 129-131 °C

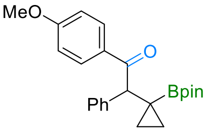

1-(4-Methoxyphenyl)-2-phenyl-2-(1-(4,4,5,5-tetramethyl-1,3,2-dioxaborolan-2-yl)cyclopropyl)ethan-1-one (4e)

The title compound was prepared from (cyclopropylidenemethyl)benzene (27 uL, 0.2 mmol) and 1-iodo-4-methoxybenzene (70.2 mg, 0.3 mmol), according to general borocarbonylation II (ring close). The crude residue was purified by flash chromatography (pentane/EA = 15:1, Rf = 0.31) to give the product as a white solid (51.9 mg, 66%).

^{1}H NMR (300 MHz, $CDCl_3$) δ 7.87 – 7.80 (m, 2H), 7.26 – 7.20 (m, 2H), 7.20 – 7.10 (m, 3H), 6.84 – 6.72 (m, 2H), 4.83 (s, 1H), 3.79 (s, 3H), 1.19 (s, 6H), 1.13 (s, 6H), 0.83 (ddd, J = 8.8, 5.9, 4.1 Hz, 1H), 0.58 – 0.44 (m, 1H), 0.40 – 0.25 (m, 1H), 0.18 – 0.02 (m, 1H).

^{13}C NMR (75 MHz, $CDCl_3$) δ 199.0, 162.7, 136.7, 131.1, 130.2, 130.1, 128.2, 126.8, 113.3, 83.1, 55.9, 55.3, 24.8, 24.1, 6.5, 6.3.

^{11}B NMR (96 MHz, $CDCl_3$) δ 30.3.

HRMS (EI): calcd for [M] $C_{24}H_{29}BO_4$ 392.21534, found: 392.21566.

IR (ATR): 1668, 1602, 1406, 1307, 1250, 1176, 1134, 1029, 709 cm^{-1}.

Mp: 113-114 ºC

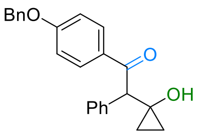

1-(4-(Benzyloxy)phenyl)-2-phenyl-2-(1-(4,4,5,5-tetramethyl-1,3,2-dioxaborolan-2-yl)cyclopropyl)ethan-1-one (4f)

The title compound was prepared from (cyclopropylidenemethyl)benzene (27 uL, 0.2 mmol) and 1-(benzyloxy)-4-iodobenzene (34 uL, 0.3 mmol), according to general borocarbonylation III (ring close). The crude residue was purified by flash chromatography (pentane/EA = 10:1, Rf = 0.20) to give the product as a colorless oil (58.9 mg, 82%).

^{1}H NMR (300 MHz, CDCl$_3$) δ 7.93 – 7.86 (m, 2H), 7.44 – 7.25 (m, 10H), 6.98 – 6.89 (m, 2H), 5.10 (s, 2H), 4.29 (d, *J* = 1.0 Hz, 1H), 4.20 (s, 1H), 1.01 – 0.92 (m, 1H), 0.89 – 0.79 (m, 1H), 0.72 – 0.62 (m, 1H), 0.54 (ddd, *J* = 10.6, 6.8, 5.2 Hz, 1H).

^{13}C NMR (75 MHz, CDCl$_3$) δ 200.4, 162.8, 136.8, 136.0, 131.3, 129.2, 128.9, 128.8, 128.7, 128.3, 127.4, 127.4, 114.6, 70.1, 59.1, 57.3, 13.3, 11.4.

HRMS (EI): calcd for [M] $C_{24}H_{22}O_3$ 358.15635, found: 358.15654.

IR (ATR): 1657, 1595, 1250, 1217, 1164, 1010, 728, 696, 647, 513 cm^{-1}.

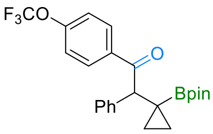

2-Phenyl-2-(1-(4,4,5,5-tetramethyl-1,3,2-dioxaborolan-2-yl)cyclopropyl)-1-(4-(trifluoromethoxy)phenyl)ethan-1-one (4g)

The title compound was prepared from (cyclopropylidenemethyl)benzene (27 uL, 0.2 mmol) and 1-iodo-4-(trifluoromethoxy)benzene (45 uL, 0.3 mmol), according to general borocarbonylation II (ring close). The crude residue was purified by flash chromatography (pentane/EA = 20:1, Rf = 0.41) to give the product as a colorless solid (58.2 mg, 65%).

^{1}H NMR (300 MHz, CDCl$_3$) δ 7.93 – 7.84 (m, 2H), 7.30 – 7.17 (m, 3H), 7.16 – 7.07 (m, 4H), 4.86 (s, 1H), 1.19 (s, 6H), 1.13 (s, 6H), 0.87 (ddd, *J* = 8.9, 5.9, 4.2 Hz, 1H), 0.50 (ddd, *J* = 9.4, 5.7, 3.8 Hz, 1H), 0.41 – 0.28 (m, 1H), 0.15 – 0.03 (m, 1H).

^{13}C NMR (75 MHz, CDCl$_3$) δ 199.2, 151.8, 135.6, 135.5, 130.7, 130.1, 128.4, 127.2, 120.2 (q, *J* = 258.5 Hz), 120.0, 83.3, 56.1, 24.8, 24.1, 6.5, 6.2.

^{11}B NMR (96 MHz, CDCl$_3$) δ 33.8.

^{19}F NMR (282 MHz, CDCl$_3$) δ -57.6.

HRMS (EI): calcd for [M] $C_{24}H_{26}BF_3O_4$ 446.18708, found: 446.18792.

IR (ATR): 1680, 1410, 1321, 1256, 1210, 1168, 1133, 972, 961, 874, 841, 705, 671 cm^{-1}.

Mp: 122-124 °C

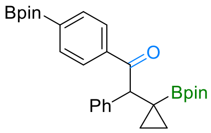

2-Phenyl-2-(1-(4,4,5,5-tetramethyl-1,3,2-dioxaborolan-2-yl)cyclopropyl)-1-(4-(4,4,5,5-tetramethyl-1,3,2-dioxaborolan-2-yl)phenyl)ethan-1-one (4h)

The title compound was prepared from (cyclopropylidenemethyl)benzene (27 uL, 0.2 mmol) and 2-(4-iodophenyl)-4,4,5,5-tetramethyl-1,3,2-dioxaborolane (98.1 mg, 0.3 mmol), according to general borocarbonylation II (ring close). The crude residue was purified by flash chromatography (pentane/EA = 20:1, Rf = 0.20) to give the product as a white solid (45.8 mg, 47%).

^{1}H NMR (300 MHz, CDCl$_3$) δ 7.85 – 7.79 (m, 2H), 7.77 – 7.72 (m, 2H), 7.25 – 7.08 (m, 5H), 4.90 (s, 1H), 1.31 (s, 12H), 1.19 (s, 6H), 1.12 (s, 6H), 0.85 (ddd, *J* = 8.8, 5.9, 4.2 Hz, 1H), 0.50 (ddd, *J* = 8.9, 5.6, 3.7 Hz, 1H), 0.39 – 0.28 (m, 1H), 0.15 – 0.05 (m, 1H).

^{13}C NMR (75 MHz, CDCl$_3$) δ 200.9, 139.3, 135.9, 134.5, 130.2, 128.2, 127.8, 126.9, 84.0, 83.2, 56.1, 24.8, 24.8, 24.8, 24.1, 6.5, 6.3.

^{11}B NMR (96 MHz, CDCl$_3$) δ 30.0.

HRMS (EI): calcd for [M] $C_{29}H_{38}B_2O_5$ 487.29362, found: 487.29403.

IR (ATR): 2980, 1678, 1398, 1380, 1362, 1315, 1133, 1092, 960, 708, 651 cm^{-1}.

Mp: 182-184 °C

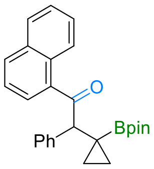

1-(Naphthalen-1-yl)-2-phenyl-2-(1-(4,4,5,5-tetramethyl-1,3,2-dioxaborolan-2-yl)cyclopropyl)ethan-1-one (4i)

The title compound was prepared from (cyclopropylidenemethyl)benzene (27 uL, 0.2 mmol) and 1-iodonaphthalene (44 uL, 0.3 mmol), according to general borocarbonylation II (ring close). The crude residue was purified by flash chromatography (pentane/EA = 20:1, Rf = 0.36) to give the product as a colorless oil (38.8 mg, 47%).

^{1}H NMR (300 MHz, CDCl$_3$) δ 8.63 (ddt, *J* = 8.5, 1.5, 0.8 Hz, 1H), 7.86 – 7.76 (m, 3H), 7.54 (ddd, *J* = 8.5, 6.8, 1.6 Hz, 1H), 7.47 (ddd, *J* = 8.1, 6.8, 1.4 Hz, 1H), 7.38 – 7.31 (m, 1H), 7.23 – 7.08 (m, 5H), 4.95 (s, 1H), 1.23 (s, 6H), 1.15 (s, 6H), 0.98 – 0.90 (m, 1H), 0.62 – 0.46 (m, 2H), 0.18 – 0.07 (m, 1H).

^{13}C NMR (75 MHz, $CDCl_3$) δ 204.5, 137.0, 135.8, 133.8, 131.6, 130.6, 130.2, 128.1, 127.4, 127.3, 126.9, 126.1, 126.1, 124.2, 83.3, 59.0, 24.8, 24.3, 7.1, 6.9.

^{11}B NMR (96 MHz, $CDCl_3$) δ 33.2.

HRMS (EI): calcd for [M] $C_{27}H_{29}BO_3$ 412.22043, found: 412.22118.

IR (ATR): 2975, 1669, 1449, 1405, 1388, 1371, 1316, 1226, 1132, 848, 777, 704, 671 cm^{-1}.

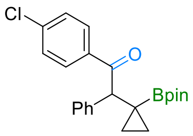

1-(4-Chlorophenyl)-2-phenyl-2-(1-(4,4,5,5-tetramethyl-1,3,2-dioxaborolan-2-yl)cyclopropyl)ethan-1-one (4j)

The title compound was prepared from (cyclopropylidenemethyl)benzene (27 uL, 0.2 mmol) and 1-chloro-4-iodobenzene (71.4 mg, 0.3 mmol), according to general borocarbonylation II (ring close). The crude residue was purified by flash chromatography (pentane/EA = 20:1, Rf = 0.41) to give the product as a white solid (40.4 mg, 51%).

^{1}H NMR (400 MHz, $CDCl_3$) δ 7.83 – 7.75 (m, 2H), 7.32 – 7.16 (m, 5H), 7.13 – 7.07 (m, 2H), 4.85 (s, 1H), 1.19 (s, 6H), 1.13 (s, 6H), 0.86 (ddd, *J* = 8.8, 6.0, 4.3 Hz, 1H), 0.55 – 0.46 (m, 1H), 0.37 – 0.30 (m, 1H), 0.13 – 0.04 (m, 1H).

^{13}C NMR (101 MHz, $CDCl_3$) δ 199.4, 138.5, 135.6, 135.6, 130.2, 130.1, 128.5, 128.4, 127.1, 83.2, 56.0, 24.8, 24.1, 6.5, 6.2.

^{11}B NMR (128 MHz, $CDCl_3$) δ 33.3.

HRMS (EI): calcd for [M] $C_{23}H_{26}BClO_3$ 396.16580, found: 396.16635.

IR (ATR): 1674, 1408, 1318, 1245, 1135, 1094, 961, 707, 676 cm^{-1}.

Mp: 132-134 °C

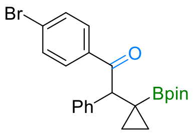

1-(4-Bromophenyl)-2-phenyl-2-(1-(4,4,5,5-tetramethyl-1,3,2-dioxaborolan-2-yl)cyclopropyl)ethan-1-one (4k)

The title compound was prepared from (cyclopropylidenemethyl)benzene (27 uL, 0.2 mmol) and 1-bromo-4-iodobenzene (84.5 mg, 0.3 mmol), according to general borocarbonylation II (ring close). The crude residue was purified by flash chromatography (pentane/EA = 20:1, Rf = 0.38) to give the product as a white solid (51.9 mg, 59%).

^{1}H NMR (300 MHz, $CDCl_3$) δ 7.73 – 7.67 (m, 2H), 7.49 – 7.39 (m, 2H), 7.29 – 7.15 (m, 3H), 7.13 – 7.06 (m, 2H), 4.84 (s, 1H), 1.19 (s, 6H), 1.12 (s, 6H), 0.86 (ddd, *J* = 8.8, 5.9, 4.2 Hz, 1H), 0.59 – 0.41 (m, 1H), 0.38 – 0.28 (m, 1H), 0.13 – 0.03 (m, 1H).

^{13}C NMR (75 MHz, $CDCl_3$) δ 199.6, 136.0, 135.6, 131.5, 130.4, 130.1, 128.4, 127.2, 127.1, 83.3, 56.0, 24.8, 24.1, 6.5, 6.2.

^{11}B NMR (96 MHz, $CDCl_3$) δ 33.2.

HRMS (EI): calcd for [M] $C_{23}H_{26}BBrO_3$ 441.11687, found: 441.11713.

IR (ATR): 1675, 1407, 1318, 1245, 1135, 962, 751, 708 cm^{-1}.

Mp: 139-141 °C

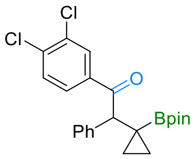

1-(3,4-Dichlorophenyl)-2-phenyl-2-(1-(4,4,5,5-tetramethyl-1,3,2-dioxaborolan-2-yl)cyclopropyl)ethan-1-one (4l)

The title compound was prepared from (cyclopropylidenemethyl)benzene (27 uL, 0.2 mmol) and 1,2-dichloro-4-iodobenzene (81.9 uL, 0.3 mmol), according to general borocarbonylation II (ring close). The crude residue was purified by flash chromatography (pentane/EA = 20:1, Rf = 0.41) to give the product as a colorless oil (38.7 mg, 45%).

^{1}H NMR (300 MHz, $CDCl_3$) δ 7.97 (d, J = 2.0 Hz, 1H), 7.65 (dd, J = 8.4, 2.1 Hz, 1H), 7.40 (d, J = 8.3 Hz, 1H), 7.34 – 7.19 (m, 3H), 7.11 (dd, J = 7.9, 1.7 Hz, 2H), 4.85 (s, 1H), 1.22 (s, 6H), 1.15 (s, 6H), 0.89 (ddd, J = 8.8, 5.9, 4.2 Hz, 1H), 0.59 – 0.44 (m, 1H), 0.42 – 0.31 (m, 1H), 0.09 (ddd, J = 8.7, 5.9, 3.8 Hz, 1H).

^{13}C NMR (75 MHz, $CDCl_3$) δ 198.5, 136.9, 136.6, 135.1, 132.8, 130.7, 130.3, 130.1, 128.5, 127.8, 127.3, 83.4, 56.0, 24.8, 24.1, 6.5, 6.2.

^{11}B NMR (96 MHz, $CDCl_3$) δ 33.6.

HRMS (EI): calcd for [M] $C_{23}H_{25}BCl_2O_3$ 432.12388, found: 432.12456.

IR (ATR): 1680, 1406, 1318, 1205, 1134, 979, 965, 847, 733, 704, 668 cm^{-1}.

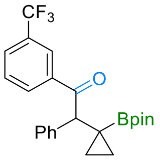

2-Phenyl-2-(1-(4,4,5,5-tetramethyl-1,3,2-dioxaborolan-2-yl)cyclopropyl)-1-(3-(trifluoromethyl)phenyl)ethan-1-one (4m)

The title compound was prepared from (cyclopropylidenemethyl)benzene (27 uL, 0.2 mmol) and 1-iodo-3-(trifluoromethyl)benzene (43 uL, 0.3 mmol), according to general borocarbonylation II (ring close). The crude residue was purified by flash chromatography (pentane/EA = 20:1, Rf = 0.37) to give the product as a colorless oil (52.8 mg, 61%).

^{1}H NMR (300 MHz, CDCl$_3$) δ 8.13 (dt, *J* = 1.8, 1.1 Hz, 1H), 7.97 (dddd, *J* = 7.9, 1.7, 1.1, 0.6 Hz, 1H), 7.66 (dddd, *J* = 7.8, 1.9, 1.2, 0.6 Hz, 1H), 7.43 (tt, *J* = 7.9, 0.7 Hz, 1H), 7.30 – 7.16 (m, 3H), 7.15 – 7.09 (m, 2H), 4.89 (s, 1H), 1.19 (s, 6H), 1.13 (s, 6H), 0.88 (ddd, *J* = 8.8, 5.9, 4.2 Hz, 1H), 0.59 – 0.46 (m, 1H), 0.43 – 0.33 (m, 1H), 0.09 (ddd, *J* = 8.7, 5.9, 3.7 Hz, 1H).

^{13}C NMR (75 MHz, CDCl$_3$) δ 199.5, 138.0, 135.2, 131.8, 131.2 (q, *J* = 33.0 Hz), 130.1, 128.8, 128.5 (q, *J* = 3.7 Hz), 128.5, 127.3, 125.6 (q, *J* = 3.7 Hz), 123.7 (q, *J* = 272.8 Hz), 83.4, 56.2, 24.8, 24.1, 6.6, 6.4.

^{11}B NMR (96 MHz, CDCl$_3$) δ 32.9.

^{19}F NMR (282 MHz, CDCl$_3$) δ -62.9.

HRMS (EI): calcd for [M] $C_{24}H_{26}BF_3O_3$ 429.19579, found: 429.19489.

IR (ATR): 1689, 1406, 1329, 1202, 1166, 1126, 1071, 692, 670 cm^{-1}.

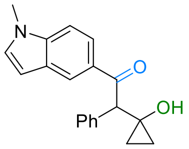

1-(1-Methyl-1*H*-indol-5-yl)-2-phenyl-2-(1-(4,4,5,5-tetramethyl-1,3,2-dioxaborolan-2-yl)cyclopropyl)ethan-1-one (4n)

The title compound was prepared from (cyclopropylidenemethyl)benzene (27 uL, 0.2 mmol) and 5-iodo-1-methyl-1*H*-indole (76.8 mg, 0.3 mmol), according to general borocarbonylation III (ring close). The crude residue was purified by flash chromatography (pentane/EA = 10:1, Rf = 0.20) to give the product as a colorless oil (48.3 mg, 79%).

^{1}H NMR (300 MHz, CDCl$_3$) δ 8.22 (d, *J* = 1.1 Hz, 1H), 7.85 (dd, *J* = 8.8, 1.7 Hz, 1H), 7.40 – 7.35 (m, 2H), 7.35 – 7.26 (m, 3H), 7.26 – 7.20 (m, 1H), 7.07 (d, *J* = 3.2 Hz, 1H), 6.53 (dd, *J* = 3.2, 0.9 Hz, 1H), 4.49 (s, 1H), 4.32 (s, 1H), 3.77 (s, 3H), 1.01 – 0.91 (m, 1H), 0.83 (ddd, *J* = 10.3, 6.5, 5.5 Hz, 1H), 0.76 – 0.62 (m, 1H), 0.55 (ddd, *J* = 10.3, 6.5, 4.8 Hz, 1H).

^{13}C NMR (75 MHz, CDCl$_3$) δ 202.1, 139.2, 137.3, 130.5, 129.0, 128.7, 128.2, 127.9, 127.3, 124.0, 122.5, 109.2, 103.2, 59.0, 57.6, 33.0, 13.4, 11.5.

HRMS (EI): calcd for [M] $C_{20}H_{19}NO_2$ 305.14103, found: 305.14076.

IR (ATR): 1648, 1600, 1340, 1249, 1146, 1097, 714, 700, 586, 424 cm^{-1}.

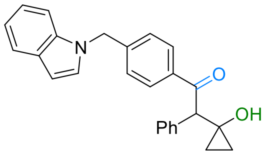

1-(4-((1*H*-Indol-1-yl)methyl)phenyl)-2-phenyl-2-(1-(4,4,5,5-tetramethyl-1,3,2-dioxaborolan-2-yl)cyclopropyl)ethan-1-one (4o)

The title compound was prepared from (cyclopropylidenemethyl)benzene (27 uL, 0.2 mmol) and 1-(4-iodobenzyl)-1*H*-indole (100 mg, 0.3 mmol), according to general borocarbonylation III (ring close).

The crude residue was purified by flash chromatography (pentane/EA = 10:1, Rf = 0.25) to give the product as a colorless oil (48.0 mg, 63%).

^{1}H NMR (300 MHz, CDCl$_3$) δ 7.91 – 7.84 (m, 1H), 7.82 – 7.76 (m, 1H), 7.74 – 7.70 (m, 1H), 7.43 – 7.21 (m, 10H), 7.15 (d, *J* = 3.1 Hz, 1H), 6.68 (d, *J* = 3.2 Hz, 1H), 5.39 (s, 2H), 4.19 (s, 1H), 4.11 (d, *J* = 0.9 Hz, 1H), 1.06 – 0.96 (m, 1H), 0.94 – 0.84 (m, 1H), 0.74 – 0.63 (m, 1H), 0.55 (ddd, *J* = 10.5, 6.4, 4.8 Hz, 1H).

^{13}C NMR (75 MHz, CDCl$_3$) δ 201.3, 138.2, 136.4, 136.0, 131.4, 129.1, 129.0, 128.9, 128.1, 128.0, 127.5, 127.1, 121.9, 121.1, 119.7, 109.4, 102.2, 59.7, 57.0, 49.6, 13.1, 11.4.

HRMS (EI): calcd for [M] $C_{26}H_{23}NO_2$ 381.17233, found: 381.17237.

IR (ATR): 1668, 1461, 1254, 1184, 728, 698, 658, 512, 460, 425 cm^{-1}.

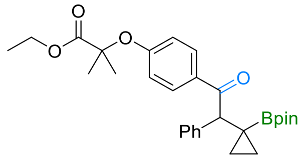

Ethyl 2-methyl-2-(4-(2-phenyl-2-(1-(4,4,5,5-tetramethyl-1,3,2-dioxaborolan-2-yl)cyclopropyl)acetyl)phenoxy)propanoate (4p)

The title compound was prepared from (cyclopropylidenemethyl)benzene (27 uL, 0.2 mmol) and ethyl 2-(4-iodophenoxy)-2-methylpropanoate (100 mg, 0.3 mmol), according to general borocarbonylation II (ring close). The crude residue was purified by flash chromatography (pentane/EA = 15:1, Rf = 0.26) to give the product as a colorless oil (51.1 mg, 52%).

^{1}H NMR (300 MHz, CDCl$_3$) δ 7.79 – 7.71 (m, 2H), 7.26 – 7.09 (m, 5H), 6.75 – 6.61 (m, 2H), 4.80 (s, 1H), 4.17 (qd, *J* = 7.2, 0.9 Hz, 2H), 1.59 (s, 3H), 1.59 (s, 3H), 1.18 (s, 6H), 1.15 (t, *J* = 7.2 Hz, 3H), 1.12 (s, 6H), 0.84 – 0.76 (m, 1H), 0.50 (ddd, *J* = 9.0, 5.7, 3.8 Hz, 1H), 0.39 – 0.27 (m, 1H), 0.17 – 0.04 (m, 1H).

^{13}C NMR (75 MHz, CDCl$_3$) δ 199.1, 173.8, 158.9, 136.6, 130.8, 130.6, 130.1, 128.2, 126.8, 117.0, 83.1, 79.1, 61.6, 55.9, 25.4, 25.3, 24.7, 24.1, 13.9, 6.5, 6.3.

^{11}B NMR (96 MHz, CDCl$_3$) δ 34.4.

HRMS (EI): calcd for [M] $C_{29}H_{37}BO_6$ 492.26777, found: 492.26874.

IR (ATR): 1731, 1671, 1594, 1413, 1221, 1172, 1136, 1113, 708, 621 cm^{-1}.

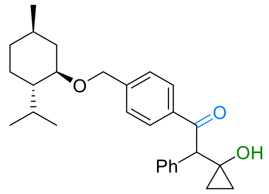

1-(4-((((1R,2S,5R)-2-Isopropyl-5-methylcyclohexyl)oxy)methyl)phenyl)-2-phenyl-2-(1-(4,4,5,5-tetramethyl-1,3,2-dioxaborolan-2-yl)cyclopropyl)ethan-1-one (4q)

The title compound was prepared from (cyclopropylidenemethyl)benzene (27 uL, 0.2 mmol) and 1-iodo-4-((((1R,2S,5R)-2-isopropyl-5-methylcyclohexyl)oxy)methyl)benzene (111.0 mg, 0.3 mmol), according to general borocarbonylation III (ring close). The crude residue was purified by flash chromatography (pentane/EA = 10:1, Rf = 0.20) to give the product as a colorless oil (54.2 mg, 65%).

^{1}H NMR (300 MHz, CDCl$_3$) δ 7.90 – 7.80 (m, 2H), 7.38 – 7.27 (m, 6H), 7.26 – 7.21 (m, 1H), 4.64 (dd, *J* = 12.4, 2.0 Hz, 1H), 4.37 (dd, *J* = 12.4, 2.0 Hz, 1H), 4.24 (s, 1H), 4.12 (d, *J* = 1.0 Hz, 1H), 3.14 (tdd, *J* = 10.5, 4.3, 1.7 Hz, 1H), 2.24 (dtt, *J* = 10.5, 6.8, 3.2 Hz, 1H), 2.17 – 2.07 (m, 1H), 1.70 – 1.57 (m, 2H), 1.38 – 1.26 (m, 2H), 0.93 (t, *J* = 6.1 Hz, 5H), 0.88 (dd, *J* = 7.1, 2.7 Hz, 5H), 0.83 – 0.76 (m, 1H),0.73 – 0.61 (m, 4H), 0.51 (ddd, *J* = 10.3, 6.5, 4.8 Hz, 1H).

^{13}C NMR (75 MHz, CDCl$_3$) δ 201.5, 145.2, 136.4, 129.1, 129.0, 128.8, 127.5, 127.4, 79.3, 69.6, 59.5, 57.2, 48.2, 40.2, 34.5, 31.5, 25.6, 23.2, 22.3, 21.0, 16.1, 13.2, 11.5.

HRMS (EI): calcd for [M] $C_{28}H_{36}O_3$ 420.26590, found: 420.26494.

IR (ATR): 2951, 2920, 2866, 1668, 1255, 1215, 1087, 1013, 729, 699, 590, 507 cm^{-1}.

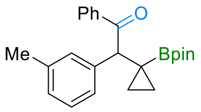

1-Phenyl-2-(1-(4,4,5,5-tetramethyl-1,3,2-dioxaborolan-2-yl)cyclopropyl)-2-(m-tolyl)ethan-1-one (4r)

The title compound was prepared from 1-(cyclopropylidenemethyl)-3-methylbenzene (32 uL, 0.2 mmol) and iodobenzene (34 uL, 0.3 mmol), according to general borocarbonylation II (ring close). The crude residue was purified by flash chromatography (pentane/EA = 20:1, Rf = 0.35) to give the product as a colorless oil (40.0 mg, 53%).

^{1}H NMR (300 MHz, CDCl$_3$) δ 7.95 – 7.78 (m, 2H), 7.45 – 7.37 (m, 1H), 7.32 (ddt, *J* = 8.3, 6.8, 1.3 Hz, 2H), 7.16 – 7.10 (m, 1H), 7.02 – 6.89 (m, 3H), 4.84 (s, 1H), 2.27 (d, *J* = 0.6 Hz, 3H), 0.85 (ddd, *J* = 8.8, 5.9, 4.1 Hz, 1H), 0.50 (ddd, *J* = 8.8, 5.7, 3.7 Hz, 1H), 0.42 – 0.30 (m, 1H), 0.19 – 0.04 (m, 1H).

^{13}C NMR (75 MHz, CDCl$_3$) δ 200.7, 137.7, 137.4, 135.9, 132.1, 130.7, 128.8, 128.1, 128.1, 127.7, 127.3, 83.1, 56.1, 24.8, 24.2, 21.4, 6.6, 6.4.

^{11}B NMR (96 MHz, CDCl$_3$) δ 33.5.

HRMS (EI): calcd for [M] $C_{24}H_{29}BO_3$ 376.22043, found: 376.22116.

IR (ATR): 1677, 1405, 1306, 1131, 960, 849, 751, 715, 698 cm^{-1}.

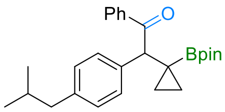

2-(4-Isobutylphenyl)-1-phenyl-2-(1-(4,4,5,5-tetramethyl-1,3,2-dioxaborolan-2-yl)cyclopropyl)ethan-1-one (4s)

The title compound was prepared from (cyclopropylidenemethyl)-4-isobutylbenzene (37.2 mg, 0.2 mmol) and iodobenzene (34 uL, 0.3 mmol), according to general borocarbonylation II (ring close). The crude residue was purified by flash chromatography (pentane/EA = 20:1, Rf = 0.36) to give the product as a colorless oil (37.9 mg, 45%).

^{1}H NMR (300 MHz, CDCl$_3$) δ 7.89 – 7.80 (m, 2H), 7.45 – 7.36 (m, 1H), 7.34 – 7.27 (m, 2H), 7.06 – 6.97 (m, 4H), 4.88 (s, 1H), 2.38 (d, *J* = 7.2 Hz, 2H), 1.79 (hept, *J* = 6.6 Hz, 1H), 1.19 (s, 6H), 1.13 (s, 6H), 0.84 (dd, *J* = 6.6, 0.6 Hz, 7H), 0.48 (ddd, *J* = 8.8, 5.7, 3.6 Hz, 1H), 0.39 – 0.30 (m, 1H), 0.17 – 0.06 (m, 1H).

^{13}C NMR (75 MHz, CDCl$_3$) δ 200.8, 140.3, 137.4, 133.1, 132.0, 129.9, 129.0, 128.8, 128.1, 83.2, 55.6, 45.0, 30.1, 24.8, 24.1, 22.3, 6.5, 6.1.

^{11}B NMR (96 MHz, CDCl$_3$) δ 33.6.

HRMS (EI): calcd for [M] $C_{27}H_{35}BO_3$ 418.26738, found: 418.26821.

IR (ATR): 1681, 1405, 1314, 1213, 1132, 960, 845, 689, 670 cm^{-1}.

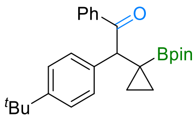

2-(4-(*tert*-Butyl)phenyl)-1-phenyl-2-(1-(4,4,5,5-tetramethyl-1,3,2-dioxaborolan-2-yl)cyclopropyl)ethan-1-one (4t)

The title compound was prepared from 1-(tert-butyl)-4-(cyclopropylidenemethyl)benzene (40 uL, 0.2 mmol) and iodobenzene (34 uL, 0.3 mmol), according to general borocarbonylation II (ring close). The crude residue was purified by flash chromatography (pentane/EA = 20:1, Rf = 0.36) to give the product as a colorless oil (37.0 mg, 44%).

^{1}H NMR (300 MHz, CDCl$_3$) δ 7.92 – 7.81 (m, 2H), 7.45 – 7.38 (m, 1H), 7.35 – 7.28 (m, 2H), 7.26 – 7.20 (m, 2H), 7.09 – 7.01 (m, 2H), 4.90 (s, 1H), 1.25 (s, 9H), 1.19 (s, 6H), 1.13 (s, 6H), 0.85 (ddd, *J* = 8.7, 6.0, 4.1 Hz, 1H), 0.54 – 0.44 (m, 1H), 0.41 – 0.30 (m, 1H), 0.17 – 0.06 (m, 1H).

^{13}C NMR (75 MHz, CDCl$_3$) δ 200.9, 149.7, 137.5, 132.6, 132.0, 129.7, 128.8, 128.1, 125.1, 83.2, 55.3, 34.3, 31.3, 24.8, 24.1, 6.6, 6.2.

^{11}B NMR (96 MHz, CDCl$_3$) δ 32.9.

HRMS (EI): calcd for [M] $C_{27}H_{35}BO_3$ 418.26738, found: 418.26794.

IR (ATR): 1685, 1405, 1314, 1215, 1133, 1111, 962, 847, 716, 688, 671, 653, 605 cm^{-1}.

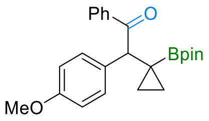

2-(4-Methoxyphenyl)-1-phenyl-2-(1-(4,4,5,5-tetramethyl-1,3,2-dioxaborolan-2-yl)cyclopropyl)ethan-1-one (4u)

The title compound was prepared from 1-(cyclopropylidenemethyl)-4-methoxybenzene (32.0 mg, 0.2 mmol) and iodobenzene (34 uL, 0.3 mmol), according to general borocarbonylation II (ring close). The crude residue was purified by flash chromatography (pentane/EA = 20:1, Rf = 0.21) to give the product as a colorless oil (42.4 mg, 54%).

^{1}H NMR (300 MHz, CDCl$_3$) δ 7.88 – 7.80 (m, 2H), 7.45 – 7.37 (m, 1H), 7.34 – 7.27 (m, 2H), 7.08 – 7.02 (m, 2H), 6.80 – 6.74 (m, 2H), 4.84 (s, 1H), 3.74 (s, 3H), 1.19 (s, 6H), 1.13 (s, 6H), 0.84 (ddd, J = 8.9, 5.9, 4.2 Hz, 1H), 0.59 – 0.42 (m, 1H), 0.38 – 0.28 (m, 1H), 0.16 – 0.05 (m, 1H).

^{13}C NMR (101 MHz, CDCl$_3$) δ 200.8, 158.5, 137.4, 132.1, 131.2, 128.8, 128.1, 128.0, 113.7, 83.1, 55.3, 55.1, 24.8, 24.1, 6.4, 6.2.

^{11}B NMR (128 MHz, CDCl$_3$) δ 33.1.

HRMS (EI): calcd for [M] $C_{24}H_{29}BO_4$ 392.21534, found: 392,21754.

IR (ATR): 1679, 1509, 1403, 1302, 1251, 1229, 1130, 1031, 841, 761, 698, 680, 669 cm^{-1}.

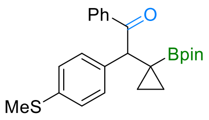

2-(4-(Methylthio)phenyl)-1-phenyl-2-(1-(4,4,5,5-tetramethyl-1,3,2-dioxaborolan-2-yl)cyclopropyl)ethan-1-one (4v)

The title compound was prepared from (4-(cyclopropylidenemethyl)phenyl)(methyl)sulfane (35.2 mg, 0.2 mmol) and iodobenzene (34 uL, 0.3 mmol), according to general borocarbonylation II (ring close). The crude residue was purified by flash chromatography (pentane/EA = 20:1, Rf = 0.21) to give the product as a colorless oil (47.8 mg, 59%).

^{1}H NMR (300 MHz, CDCl$_3$) δ 7.88 – 7.80 (m, 2H), 7.45 – 7.38 (m, 1H), 7.32 (ddt, J = 8.3, 6.8, 1.2 Hz, 2H), 7.15 – 7.03 (m, 4H), 4.82 (s, 1H), 2.42 (s, 3H), 1.19 (s, 6H), 1.13 (s, 6H), 0.84 (ddd, J = 8.8, 5.9, 4.2 Hz, 1H), 0.58 – 0.47 (m, 1H), 0.41 – 0.29 (m, 1H), 0.17 – 0.05 (m, 1H).

^{13}C NMR (75 MHz, CDCl$_3$) δ 200.5, 137.2, 137.0, 132.9, 132.2, 130.5, 128.8, 128.2, 126.2, 83.2, 55.6, 24.7, 24.2, 15.5, 6.6, 6.4.

^{11}B NMR (96 MHz, CDCl$_3$) δ 33.1.

HRMS (EI): calcd for [M] $C_{24}H_{29}BO_3S$ 408.19250, found: 408.19333.

IR (ATR): 2974, 1678, 1398, 1308, 1216, 1130, 960, 841, 713, 678 cm^{-1}.

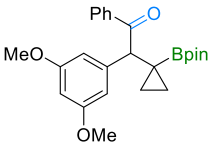

2-(3,5-Dimethoxyphenyl)-1-phenyl-2-(1-(4,4,5,5-tetramethyl-1,3,2-dioxaborolan-2-yl)cyclopropyl)ethan-1-one (4w)

The title compound was prepared from 1-(cyclopropylidenemethyl)-3,5-dimethoxybenzene (38 uL, 0.2 mmol) and iodobenzene (34 uL, 0.3 mmol), according to general borocarbonylation II (ring close). The crude residue was purified by flash chromatography (pentane/EA = 20:1, Rf = 0.16) to give the product as a colorless oil (58.5 mg, 70%).

^{1}H NMR (300 MHz, $CDCl_3$) δ 7.92 – 7.80 (m, 2H), 7.46 – 7.39 (m, 1H), 7.36 – 7.28 (m, 2H), 6.32 (d, *J* = 2.2 Hz, 2H), 6.31 – 6.26 (m, 1H), 4.71 (s, 1H), 3.71 (s, 6H), 1.20 (s, 6H), 1.14 (s, 6H), 0.84 (ddd, *J* = 8.8, 6.0, 4.0 Hz, 1H), 0.59 – 0.51 (m, 1H), 0.50 – 0.41 (m, 1H), 0.25 – 0.15 (m, 1H).

^{13}C NMR (75 MHz, $CDCl_3$) δ 200.4, 160.4, 138.5, 137.3, 132.2, 128.7, 128.2, 108.3, 99.0, 83.1, 56.9, 55.2, 25.0, 24.7, 24.2, 7.1, 7.1.

^{11}B NMR (96 MHz, $CDCl_3$) δ 32.2.

HRMS (EI): calcd for [M] $C_{25}H_{31}BO_5$ 422.22591, found: 422.22701.

IR (ATR): 1681, 1593, 1404, 1314, 1203, 1131, 1060, 848, 688 cm^{-1}.

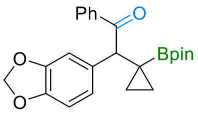

2-(Benzo[d][1,3]dioxol-5-yl)-1-phenyl-2-(1-(4,4,5,5-tetramethyl-1,3,2-dioxaborolan-2-yl)cyclopropyl)ethan-1-one (4x)

The title compound was prepared from 5-(cyclopropylidenemethyl)benzo[d][1,3]dioxole (31 uL, 0.2 mmol) and iodobenzene (34 uL, 0.3 mmol), according to general borocarbonylation II (ring close). The crude residue was purified by flash chromatography (pentane/EA = 20:1, Rf = 0.21) to give the product as a colorless oil (49.6 mg, 61%).

^{1}H NMR (300 MHz, $CDCl_3$) δ 7.89 – 7.83 (m, 2H), 7.47 – 7.39 (m, 1H), 7.33 (ddt, *J* = 8.3, 6.8, 1.2 Hz, 2H), 6.71 – 6.65 (m, 2H), 6.59 (dd, *J* = 8.0, 1.8 Hz, 1H), 5.91 – 5.86 (m, 2H), 4.77 (s, 1H), 1.19 (s, 6H), 1.13 (s, 6H), 0.85 (ddd, *J* = 8.8, 5.9, 4.1 Hz, 1H), 0.53 (ddd, *J* = 8.8, 5.6, 3.6 Hz, 1H), 0.46 – 0.30 (m, 1H), 0.21 – 0.12 (m, 1H).

^{13}C NMR (101 MHz, $CDCl_3$) δ 200.5, 147.5, 146.5, 137.3, 132.2, 129.8, 128.8, 128.2, 123.5, 110.4, 108.1, 100.9, 83.2, 55.8, 24.7, 24.2, 6.7, 6.6.

^{11}B NMR (128 MHz, $CDCl_3$) δ 32.9.

HRMS (EI): calcd for [M] $C_{24}H_{27}BO_5$ 406.19461, found: 406.19576.

IR (ATR): 1676, 1486, 1401, 1306, 1242, 1230, 1131, 1038, 931, 850, 699, 659 cm^{-1}.

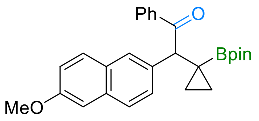

2-(6-Methoxynaphthalen-2-yl)-1-phenyl-2-(1-(4,4,5,5-tetramethyl-1,3,2-dioxaborolan-2-yl)cyclopropyl)ethan-1-one (4y)

The title compound was prepared from 2-(cyclopropylidenemethyl)-6-methoxynaphthalene (42.0 mg, 0.2 mmol) and iodobenzene (34 uL, 0.3 mmol), according to general borocarbonylation II (ring close). The crude residue was purified by flash chromatography (pentane/EA = 20:1, Rf = 0.16) to give the product as a colorless oil (61.7 mg, 70%).

^{1}H NMR (300 MHz, CDCl$_3$) δ 7.91 – 7.85 (m, 2H), 7.63 (dd, *J* = 8.9, 4.3 Hz, 2H), 7.51 (d, *J* = 2.0 Hz, 1H), 7.42 – 7.34 (m, 1H), 7.32 – 7.27 (m, 2H), 7.26 – 7.24 (m, 1H), 7.13 – 7.04 (m, 2H), 5.00 (s, 1H), 3.88 (s, 3H), 1.20 (s, 6H), 1.14 (s, 6H), 0.87 (ddd, *J* = 8.7, 5.9, 4.1 Hz, 1H), 0.57 – 0.46 (m, 1H), 0.45 – 0.35 (m, 1H), 0.19 – 0.08 (m, 1H).

^{13}C NMR (101 MHz, CDCl$_3$) δ 200.7, 157.6, 137.4, 133.5, 132.1, 131.3, 129.3, 128.8, 128.8, 128.7, 128.2, 126.8, 118.8, 105.4, 83.2, 56.1, 55.3, 24.8, 24.2, 6.7, 6.4.

^{11}B NMR (128 MHz, CDCl$_3$) δ 33.9.

HRMS (EI): calcd for [M] $C_{28}H_{31}BO_4$ 442.23099, found: 442.23141.

IR (ATR): 1680, 1604, 1405, 1390, 1314, 1258, 1222, 1204, 1163, 1133, 1028, 847, 679 cm^{-1}.

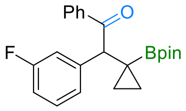

2-(4-Fluorophenyl)-1-phenyl-2-(1-(4,4,5,5-tetramethyl-1,3,2-dioxaborolan-2-yl)cyclopropyl)ethan-1-one (4z)

The title compound was prepared from 1-(cyclopropylidenemethyl)-3-fluorobenzene (28 uL, 0.2 mmol) and iodobenzene (34 uL, 0.3 mmol), according to general borocarbonylation II (ring close). The crude residue was purified by flash chromatography (pentane/EA = 20:1, Rf = 0.36) to give the product as a colorless oil (68.0 mg, 89%).

^{1}H NMR (300 MHz, CDCl$_3$) δ 7.92 – 7.74 (m, 2H), 7.48 – 7.41 (m, 1H), 7.38 – 7.30 (m, 2H), 7.21 (td, *J* = 7.9, 6.1 Hz, 1H), 7.02 – 6.82 (m, 3H), 4.80 (s, 1H), 1.18 (s, 6H), 1.13 (s, 6H), 0.85 (ddd, *J* = 8.9, 5.9, 4.2 Hz, 1H), 0.66 – 0.55 (m, 1H), 0.44 – 0.32 (m, 1H), 0.25 – 0.15 (m, 1H).

^{13}C NMR (75 MHz, CDCl$_3$) δ 200.0, 162.6 (d, *J* = 245.7 Hz), 139.2 (d, *J* = 7.1 Hz), 137.1, 132.4, 129.6 (d, *J* = 8.4 Hz), 128.7, 128.3, 125.7 (d, *J* = 3.0 Hz), 116.9 (d, *J* = 21.9 Hz), 113.9 (d, *J* = 21.2 Hz), 83.3, 56.0, 56.0, 24.7, 24.2, 7.1.

^{11}B NMR (96 MHz, CDCl$_3$) δ 32.7.

^{19}F NMR (282 MHz, CDCl$_3$) δ -112.98 (td, *J* = 9.2, 5.3 Hz).

HRMS (EI): calcd for [M] $C_{23}H_{26}BFO_3$ 380.19536, found: 380.19634.

IR (ATR): 1682, 1445, 1404, 1390, 1317, 1206, 1138, 1129, 961, 851, 737, 690 cm^{-1}.

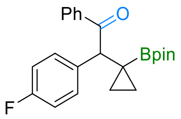

2-(4-Fluorophenyl)-1-phenyl-2-(1-(4,4,5,5-tetramethyl-1,3,2-dioxaborolan-2-yl)cyclopropyl)ethan-1-one (4aa)

The title compound was prepared from 1-(cyclopropylidenemethyl)-4-fluorobenzene (28 uL, 0.2 mmol) and iodobenzene (34 uL, 0.3 mmol), according to general borocarbonylation II (ring close). The crude residue was purified by flash chromatography (pentane/EA = 20:1, Rf = 0.36) to give the product as a colorless oil (43.0 mg, 57%).

^{1}H NMR (300 MHz, CDCl$_3$) δ 7.92 – 7.76 (m, 2H), 7.47 – 7.40 (m, 1H), 7.33 (ddt, *J* = 8.3, 6.8, 1.2 Hz, 2H), 7.18 – 7.09 (m, 2H), 6.99 – 6.87 (m, 2H), 4.82 (s, 1H), 1.18 (s, 6H), 1.13 (s, 6H), 0.84 (ddd, *J* = 8.8, 5.8, 4.2 Hz, 1H), 0.57 (ddd, *J* = 8.9, 5.6, 3.8 Hz, 1H), 0.38 – 0.28 (m, 1H), 0.20 – 0.09 (m, 1H).

^{13}C NMR (75 MHz, CDCl$_3$) δ 200.4, 161.9 (d, *J* = 245.7 Hz), 137.2, 132.3, 132.3 (d, *J* = 3.4 Hz), 131.5 (d, *J* = 7.7 Hz), 128.8, 128.2, 115.2 (d, *J* = 21.2 Hz), 83.2, 55.4, 24.7, 24.2, 6.8, 6.7.

^{11}B NMR (96 MHz, CDCl$_3$) δ 33.4.

^{19}F NMR (282 MHz, CDCl$_3$) δ -115.68 (ddd, *J* = 13.7, 8.4, 5.0 Hz).

HRMS (EI): calcd for [M] $C_{23}H_{26}BFO_3$ 380.19536, found: 380.19647.

IR (ATR): 1674, 1504, 1406, 1311, 1218, 1131, 844, 763, 696, 680, 609 cm^{-1}.

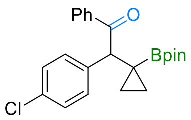

2-(4-Chlorophenyl)-1-phenyl-2-(1-(4,4,5,5-tetramethyl-1,3,2-dioxaborolan-2-yl)cyclopropyl)ethan-1-one (4bb)

The title compound was prepared from 1-chloro-4-(cyclopropylidenemethyl)benzene (32.8 mg, 0.2 mmol) and iodobenzene (34 uL, 0.3 mmol), according to general borocarbonylation II (ring close). The crude residue was purified by flash chromatography (pentane/EA = 20:1, Rf = 0.36) to give the product as a colorless oil (34.5 mg, 44%).

^{1}H NMR (300 MHz, CDCl$_3$) δ 7.90 – 7.75 (m, 2H), 7.49 – 7.40 (m, 1H), 7.37 – 7.30 (m, 2H), 7.25 – 7.18 (m, 2H), 7.15 – 7.08 (m, 2H), 4.78 (s, 1H), 1.18 (s, 6H), 1.13 (s, 6H), 0.84 (ddd, *J* = 8.8, 5.9, 4.3 Hz, 1H), 0.64 – 0.53 (m, 1H), 0.39 – 0.28 (m, 1H), 0.20 – 0.09 (m, 1H).

^{13}C NMR (75 MHz, CDCl$_3$) δ 200.2, 137.1, 135.2, 132.9, 132.4, 131.3, 128.8, 128.5, 128.3, 83.2, 55.6, 24.7, 24.2, 6.9, 6.9.

^{11}B NMR (96 MHz, CDCl$_3$) δ 32.5.

HRMS (EI): calcd for [M] $C_{23}H_{26}BClO_3$ 395.16944, found: 395.16836.

IR (ATR): 1685, 1401, 1314, 1232, 1216, 1132, 961, 816, 715, 690, 680, 670, 644 cm^{-1}.

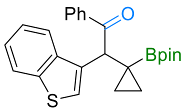

2-(Benzo[b]thiophen-3-yl)-1-phenyl-2-(1-(4,4,5,5-tetramethyl-1,3,2-dioxaborolan-2-yl)cyclopropyl)ethan-1-one (4cc)

The title compound was prepared from 3-(cyclopropylidenemethyl)benzo[b]thiophene (37.2 mg, 0.2 mmol) and iodobenzene (34 uL, 0.3 mmol), according to general borocarbonylation II (ring close). The crude residue was purified by flash chromatography (pentane/EA = 20:1, Rf = 0.21) to give the product as a colorless oil (49.7 mg, 60%).

^{1}H NMR (300 MHz, $CDCl_3$) δ 7.93 (d, J = 7.9 Hz, 1H), 7.88 – 7.79 (m, 3H), 7.48 – 7.36 (m, 3H), 7.35 – 7.26 (m, 2H), 7.11 (s, 1H), 5.42 (s, 1H), 1.20 (s, 6H), 1.16 (s, 6H), 0.93 – 0.85 (m, 1H), 0.58 – 0.46 (m, 2H), 0.08 – -0.03 (m, 1H).

^{13}C NMR (75 MHz, $CDCl_3$) δ 199.9, 140.2, 138.9, 137.1, 132.3, 130.6, 128.4, 128.3, 125.8, 124.4, 124.3, 123.0, 121.5, 83.3, 48.3, 24.8, 24.2, 7.8, 7.1.

^{11}B NMR (96 MHz, $CDCl_3$) δ 33.1.

HRMS (EI): calcd for [M] $C_{25}H_{27}BO_3S$ 418.17685, found: 418.17801.

IR (ATR): 1677, 1405, 1312, 1216, 1133, 959, 847, 755, 732, 721, 694, 672, 659 cm^{-1}.

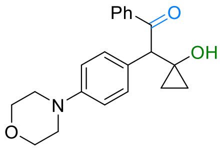

2-(4-Morpholinophenyl)-1-phenyl-2-(1-(4,4,5,5-tetramethyl-1,3,2-dioxaborolan-2-yl)cyclopropyl)ethan-1-one (4dd)

The title compound was prepared from 4-(4-(cyclopropylidenemethyl)phenyl)morpholine (43.0 mg, 0.2 mmol) and iodobenzene (34 uL, 0.3 mmol), according to general borocarbonylation III (ring close). The crude residue was purified by flash chromatography (pentane/EA = 3:1, Rf = 0.20) to give the product as a red oil (47.7 mg, 70%).

^{1}H NMR (300 MHz, $CDCl_3$) δ 7.91 – 7.85 (m, 2H), 7.51 – 7.44 (m, 1H), 7.40 – 7.32 (m, 2H), 7.22 – 7.15 (m, 2H), 6.88 – 6.82 (m, 2H), 4.22 (s, 1H), 4.06 (s, 1H), 3.84 – 3.79 (m, 4H), 3.14 – 3.10 (m, 4H), 0.96 – 0.87 (m, 1H), 0.85 – 0.75 (m, 1H), 0.67 – 0.58 (m, 1H), 0.48 (ddd, J = 10.2, 6.4, 4.7 Hz, 1H).

^{13}C NMR (75 MHz, $CDCl_3$) δ 201.9, 150.4, 136.2, 133.2, 129.8, 128.9, 128.5, 127.3, 115.8, 66.8, 58.7, 57.2, 48.9, 12.9, 11.4.

HRMS (EI): calcd for [M] $C_{21}H_{23}NO_3$ 337.16725, found: 337.16803.

IR (ATR): 1667, 1514, 1447, 1236, 1214, 1175, 1116, 924, 785, 733, 689, 658, 551 cm^{-1}.

6. Derivatization of γ-vinylboryl ketone 3a and β-cyclopropylboryl ketone 4a

6.1 Procedure of oxidation[2]

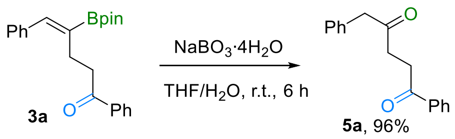

1,5-Diphenylpentane-1,4-dione (5a)

The title compound was synthesized according to the following procedure: To the boration product **3a** (36.2 mg, 0.1 mmol) in THF (2.0 mL) and water (2.0 mL) was added $NaBO_3 \cdot 4H_2O$ (76.5 mg. 5 equiv). The reaction mixture was stirred vigorously for 6 h at room temperature. The reaction mixture was quenched with water and then extracted with ethyl acetate (5 mL). The combined organic layers were washed with brine (15 mL), dried over Na_2SO_4 and concentrated. The crude product was purified by column chromatography on silica gel to afford the corresponding product **5a** as a colorless oil (24.2 mg, 96%).

^{1}H NMR (300 MHz, CDCl$_3$) δ 8.03 – 7.92 (m, 2H), 7.62 – 7.54 (m, 1H), 7.51 – 7.43 (m, 2H), 7.41 – 7.34 (m, 2H), 7.33 – 7.25 (m, 3H), 3.85 (s, 2H), 3.33 – 3.23 (m, 2H), 2.92 (dd, *J* = 6.9, 5.7 Hz, 2H).

^{13}C NMR (75 MHz, CDCl$_3$) δ 207.0, 198.5, 136.6, 134.2, 133.1, 129.5, 128.7, 128.5, 128.0, 127.0, 50.2, 35.6, 32.5.

IR (ATR): 1712, 1681, 1448, 1351, 1212, 761, 734, 689 cm^{-1}.

6.2 Procedure of iodination[3]

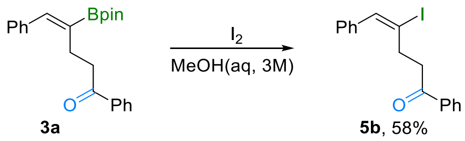

(*E*)-4-Iodo-1,5-diphenylpent-4-en-1-one (5b)

The title compound was synthesized according to the following procedure: To a solution of **3a** (36.2 mg, 0.1 mmol) in THF (1.0 mL) was added 0.1 mL of NaOH (aq. 3M, 0.3 mmol). After stirring for 10 minutes at room temperature, 0.1 mL of solution of I2 (2M, 0.2 mmol) in THF was added into the reaction mixture, and the resulting mixture was stirred for another 1 h. The mixture was then quenched with saturated aqueous $Na_2S_2O_4$ (5.0 mL) and extracted with diethyl ether for three times. The combined organic layers were washed with saturated aqueous $NHCO_3$ solution and brine, then dried over Na_2SO_4. The volatiles were removed under reduced pressure and the residue was purified by column chromatography on silica gel to afford the corresponding product **5b** as a colorless oil (21.0 mg, 58%).

^{1}H NMR (300 MHz, $CDCl_3$) δ 8.03 – 7.94 (m, 2H), 7.62 – 7.54 (m, 1H), 7.51 – 7.43 (m, 2H), 7.39 – 7.26 (m, 4H), 7.21 (ddd, J = 7.2, 1.9, 1.1 Hz, 2H), 3.36 – 3.25 (m, 2H), 3.10 – 2.96 (m, 2H).

^{13}C NMR (75 MHz, $CDCl_3$) δ 198.0, 141.9, 137.3, 136.5, 133.3, 128.7, 128.6, 128.1, 128.0, 127.5, 106.5, 39.2, 34.1.

IR (ATR): 2953, 2921, 1683, 1447, 1272, 1202, 1182, 1079, 1030, 968, 741, 688, 650 cm^{-1}.

6.3 Procedure of Suzuki-Miyaura coupling[4]

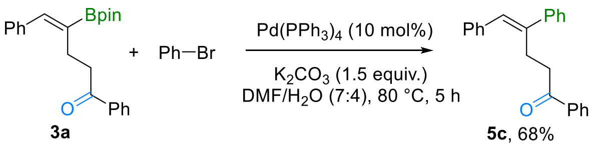

(*E*)-1,4,5-Triphenylpent-4-en-1-one (5c)

The title compound was synthesized according to the following procedure: To a 5 mL Schlenk flask charged with a magnetic stirring bar, were added the **3a** (72.4 mg, 0.2 mmol), PhBr (37.2 mg, 0.24 mmol), $Pd(PPh_3)_4$ (23.1 mg, 10 mol%), K_2CO_3 (41.7 mg, 0.3 mmol), DMF (0.7 mL) and H_2O (0.4 mL). The flask was sealed with a PTFE septum and allowed to react at 80 °C for 5 h. The reaction mixture was quenched with water and then extracted with ethyl acetate (5 mL) and concentrated. The crude product was purified by column chromatography on silica gel to afford the corresponding product **5c** as a colorless oil (42.2 mg, 68%).

^{1}H NMR (300 MHz, $CDCl_3$) δ 7.91 – 7.85 (m, 2H), 7.59 – 7.52 (m, 3H), 7.47 – 7.26 (m, 10H), 6.86 (s, 1H), 3.28 – 3.18 (m, 2H), 3.15 – 3.06 (m, 2H).

^{13}C NMR (75 MHz, $CDCl_3$) δ 199.3, 142.1, 141.4, 137.8, 136.6, 133.0, 129.2, 128.6, 128.6, 128.5, 128.4, 128.0, 127.5, 126.8, 126.6, 37.6, 24.7.

IR (ATR): 1681, 1595, 1447, 1267, 1201, 968, 757, 741, 733, 696, 689, 655, 643, 506 cm^{-1}.

6.4 Procedure of protodeboronation[5]

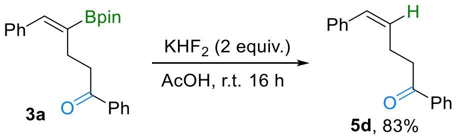

(*Z*)-1,5-Diphenylpent-4-en-1-one (5d)

The title compound was synthesized according to the following procedure: To a 5 mL Schlenk flask charged with a magnetic stirring bar, were added the **3a** (36.2 mg, 0.1 mmol), KHF2 (23.4 mg, 0.3 mmol) and HOAc (2 mL) under air. Then the reaction was allowed to stir at room temperature for 6 h. The reaction mixture was quenched with saturated $NaHCO_3$ solution slowly and resulting mixture was extracted with ethyl acetate (5 mL). The combined organic layers were washed with brine and dried over anhydrous Na_2SO_4. The volatiles were removed under reduced pressure and the residue was

purified by column chromatography on silica gel to afford the corresponding product **5d** as a colorless oil (19.6 mg, 83%).

^{1}H NMR (300 MHz, CDCl$_3$) δ 8.00 – 7.93 (m, 2H), 7.59 – 7.53 (m, 1H), 7.49 – 7.42 (m, 2H), 7.38 – 7.29 (m, 4H), 7.26 – 7.19 (m, 1H), 6.49 (dt, *J* = 11.6, 1.9 Hz, 1H), 5.72 (dt, *J* = 11.6, 7.3 Hz, 1H), 3.12 (t, *J* = 7.5 Hz, 2H), 2.79 (qd, *J* = 7.6, 2.1 Hz, 2H).

^{13}C NMR (75 MHz, CDCl$_3$) δ 199.3, 137.3, 136.8, 133.0, 130.9, 130.0, 128.7, 128.6, 128.2, 128.0, 126.7, 38.6, 23.2.

IR (ATR): 1682, 1447, 1202, 1179, 967, 767, 745, 688, 655 cm^{-1}.

6.5 Procedure of oxidation[2]

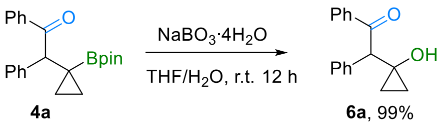

2-(1-Hydroxycyclopropyl)-1,2-diphenylethan-1-one (6a)

The title compound was synthesized according to the following procedure: To the boration product **4a** (72.4 mg, 0.2 mmol) in THF (3.0 mL) and water (3.0 mL) was added $NaBO_3 \cdot 4H_2O$ (153.0 mg. 5 equiv). The reaction mixture was stirred vigorously for 6 h at room temperature. The reaction mixture was quenched with water and then extracted with ethyl acetate (5 mL). The combined organic layers were washed with brine (15 mL), dried over Na_2SO_4 and concentrated. The crude product was purified by column chromatography on silica gel to afford the corresponding product **6a** as a colorless oil (50.3 mg, 99%).

^{1}H NMR (300 MHz, CDCl$_3$) δ 8.01 – 7.85 (m, 2H), 7.56 – 7.48 (m, 1H), 7.43 – 7.25 (m, 7H), 4.29 (s, 1H), 4.15 (d, *J* = 1.0 Hz, 1H), 0.98 (ddd, *J* = 10.6, 6.4, 4.9 Hz, 1H), 0.90 – 0.80 (m, 1H), 0.69 (dddd, *J* = 10.3, 6.3, 5.5, 1.0 Hz, 1H), 0.55 (ddd, *J* = 10.3, 6.5, 4.8 Hz, 1H).

^{13}C NMR (75 MHz, CDCl$_3$) δ 201.8, 136.3, 136.1, 133.4, 129.0, 128.9, 128.9, 128.6, 127.5, 59.5, 57.2, 13.2, 11.5.

IR (ATR): 1682, 1655, 1449, 1249, 1212, 1198, 1013, 759, 736, 700, 692, 681, 658, 545 cm^{-1}.

6.6 Procedure of reduction

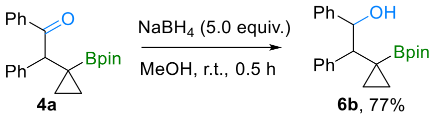

1,2-Diphenyl-2-(1-(4,4,5,5-tetramethyl-1,3,2-dioxaborolan-2-yl)cyclopropyl)ethan-1-ol (6b)

The title compound was synthesized according to the following procedure: To the boration product **4a** (36.2 mg, 0.2 mmol) in MeOH (3.0 mL) was added $NaBH_4$ (38.0 mg. 5 equiv). The reaction mixture was stirred vigorously for 0.5 h at room temperature. The reaction mixture was quenched with water

and then extracted with ethyl acetate (5 mL). The combined organic layers were washed with brine (15 mL), dried over Na_2SO_4 and concentrated. The crude product was purified by column chromatography on silica gel to afford the corresponding product **6b** as a colorless oil (27.9 mg, 77%).

^{1}H NMR (300 MHz, CDCl$_3$) δ 7.42 (dt, *J* = 8.1, 1.5 Hz, 4H), 7.36 – 7.21 (m, 6H), 5.57 (dd, *J* = 7.7, 1.8 Hz, 1H), 2.81 (d, *J* = 1.8 Hz, 1H), 1.85 (d, *J* = 7.7 Hz, 1H), 1.31 (s, 6H), 1.28 (s, 6H), 0.74 – 0.65 (m, 1H), 0.51 – 0.41 (m, 1H), 0.28 (ddd, *J* = 9.6, 5.9, 3.8 Hz, 1H), 0.00 (ddd, *J* = 8.4, 5.8, 3.8 Hz, 1H).

^{13}C NMR (75 MHz, CDCl$_3$) δ 143.8, 142.5, 129.2, 128.1, 127.8, 127.1, 126.9, 126.5, 83.5, 76.0, 64.1, 25.0, 24.2, 16.2, 11.6.

^{11}B NMR (96 MHz, CDCl$_3$) δ 33.0.

6.7 Procedure of condensation

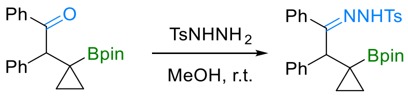

(*Z*)-*N*'-(1,2-Diphenyl-2-(1-(4,4,5,5-tetramethyl-1,3,2-dioxaborolan-2-yl)cyclopropyl)ethylidene)-4-methylbenzenesulfonohydrazide (6c)

The title compound was synthesized according to the following procedure: To the boration product **4a** (72.4 mg, 0.2 mmol) in MeOH (2.0 mL) was added Na_2SO_4 (56.8 mg, 2.0 equiv.) and $TsNHNH_2$ (56.0 mg, 1.5 equiv.). The reaction mixture was stirred vigorously for 12 h at room temperature. After the **4a** was consumed completely, the crude product was purified by column chromatography on silica gel to afford the corresponding product **6c** as a white solid (48.9 mg, 77%).

^{1}H NMR (300 MHz, CDCl$_3$) δ 7.99 – 7.86 (m, 2H), 7.38 (dt, *J* = 8.0, 0.6 Hz, 2H), 7.33 – 7.26 (m, 4H), 7.13 – 7.04 (m, 2H), 6.93 – 6.84 (m, 2H), 6.75 (dd, *J* = 8.3, 1.5 Hz, 2H), 4.05 (s, 1H), 2.50 (s, 3H), 1.22 (s, 6H), 1.14 (s, 6H), 0.92 – 0.85 (m, 1H), 0.43 – 0.26 (m, 2H), -0.13 – -0.25 (m, 1H).

^{13}C NMR (75 MHz, CDCl$_3$) δ 159.9, 143.8, 135.9, 135.6, 134.2, 130.4, 129.4, 129.2, 129.1, 128.6, 128.0, 127.6, 126.8, 83.2, 55.0, 25.2, 24.1, 21.6, 7.1, 6.7.

^{11}B NMR (96 MHz, CDCl$_3$) δ 33.5.

6.8 Procedure for trifluoroborate preparation

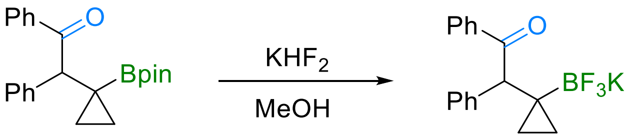

1,2-Diphenyl-2-(1-(trifluoro-l4-boraneyl)cyclopropyl)ethan-1-one, potassium salt (6d)

The title compound was synthesized according to the following procedure: To the boration product **4a** (72.4 mg, 0.2 mmol) in MeOH (5.0 mL) and H_2O (0.4 mL) was added KHF_2 (140.0 mg, 4.5 M). The reaction mixture was stirred vigorously for 3 h at room temperature. The resulting slurry was stirred

concentrated, then placed under high vacuum. The dried solids were triturated with hot acetone and filtered to remove inorganic salts. The resulting filtrate was concentrated to a minimal volume and Et2O was added to afford 6d as a white solid (45.6 mg, 67%).

^{1}H NMR (300 MHz, *d$_6$*-Acetone) δ 8.04 – 7.99 (m, 2H), 7.47 – 7.35 (m, 3H), 7.31 (ddd, *J* = 8.2, 1.7, 0.9 Hz, 2H), 7.24 – 7.18 (m, 2H), 7.15 – 7.08 (m, 1H), 5.18 (s, 1H), 0.46 – 0.35 (m, 1H), 0.14 (dd, *J* = 7.3, 1.8 Hz, 2H), -0.35 – -0.47 (m, 1H).
^{13}C NMR (75 MHz, *d$_6$*-Acetone) δ 203.7, 140.3, 140.2, 132.7, 131.1, 129.4, 129.0, 128.4, 126.8, 55.4, 5.7, 4.9.
^{11}B NMR (96 MHz, *d$_6$*-Acetone) δ 4.3.
IR (ATR): 1676, 1228, 1043, 1006, 972, 752, 700, 685, 649, 537, 482 cm^{-1}.

7. References

[1] Y. Wang, M. E. Muratore, Z. Rong, A. M. Echavarren, *Angew. Chem., Int. Ed.* **2014**, *53*, 14022.

[2] J.-E. Lee, J. Yun, *Angew. Chem. Int. Ed.* **2008**, *47*, 145.

[3] C. Wu, S. Ge, Chem. Sci. **2020**, *11*, 2783.

[4] K. Endo, M. Hirokami, T. Shibata, T. *J. Org. Chem.* 2010, **75**, 3469.

[5] M. Xiong, X. Xie, Y. Liu, *Org. Lett.* **2017**, *19*, 3398.

8. NMR Spectra of the *γ*-vinylboryl ketones, *β*-cyclopropylboryl ketones and BCPs

8.1 NMR spectra of the *γ*-vinylboryl ketones and *β*-cyclopropylboryl ketones

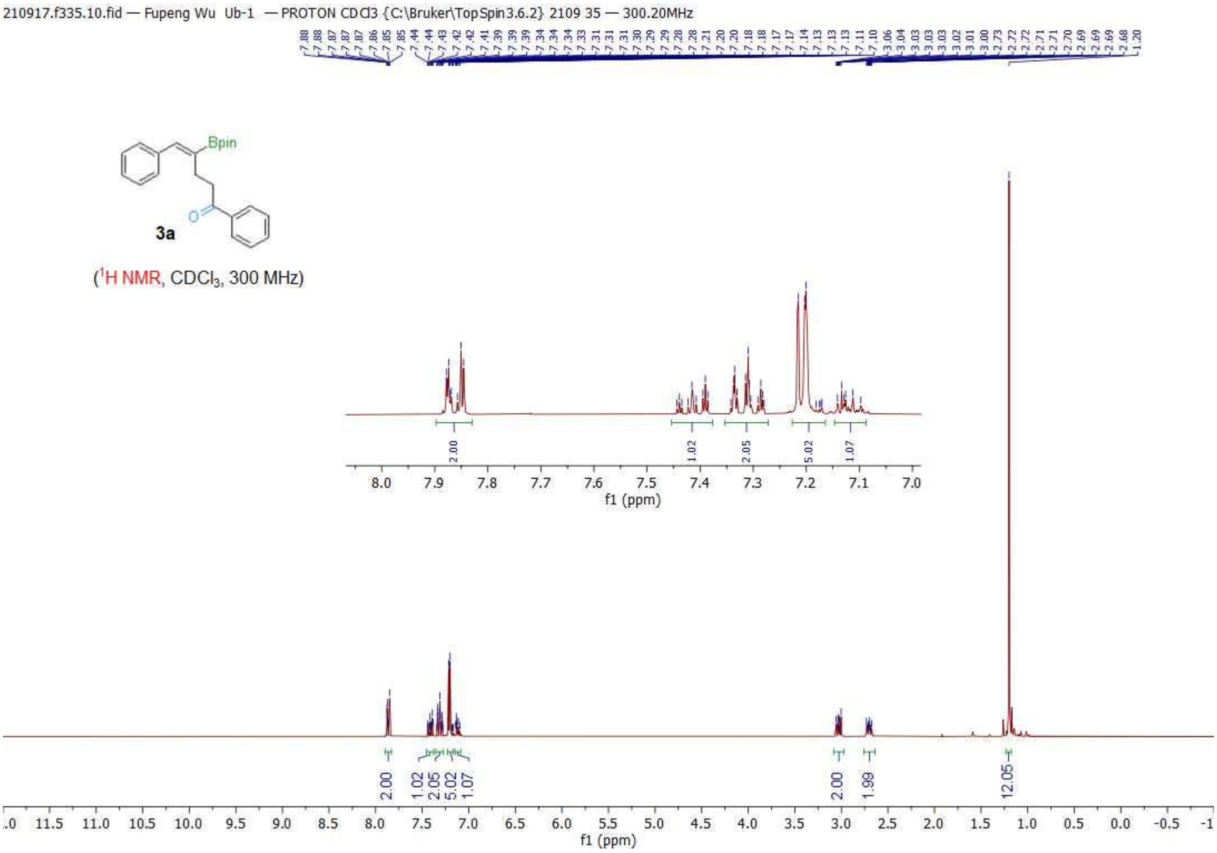

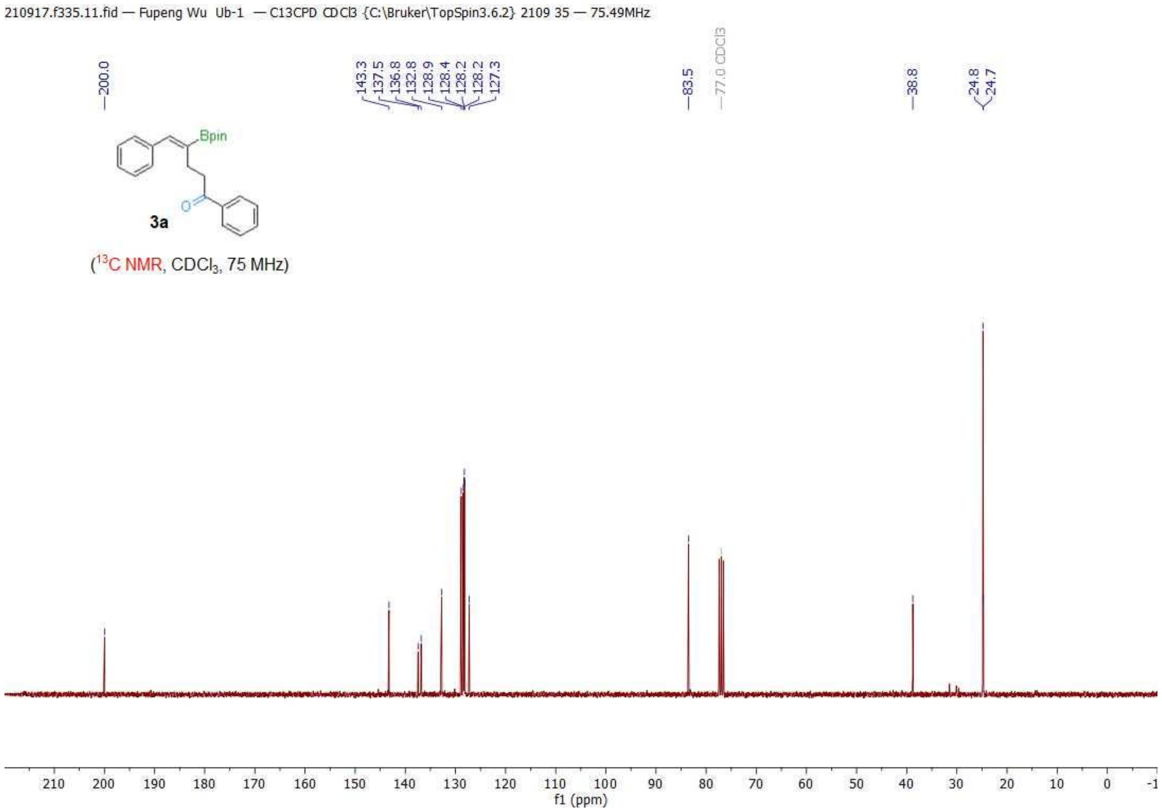

210917.f335.12.fid — Fupeng Wu Ub-1 — 11B CDCl3 {C:\Bruker\TopSpin3.6.2} 2109 35 — 96.32MHz

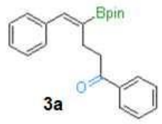

3a

(^{11}B NMR, $CDCl_3$, 96 MHz)

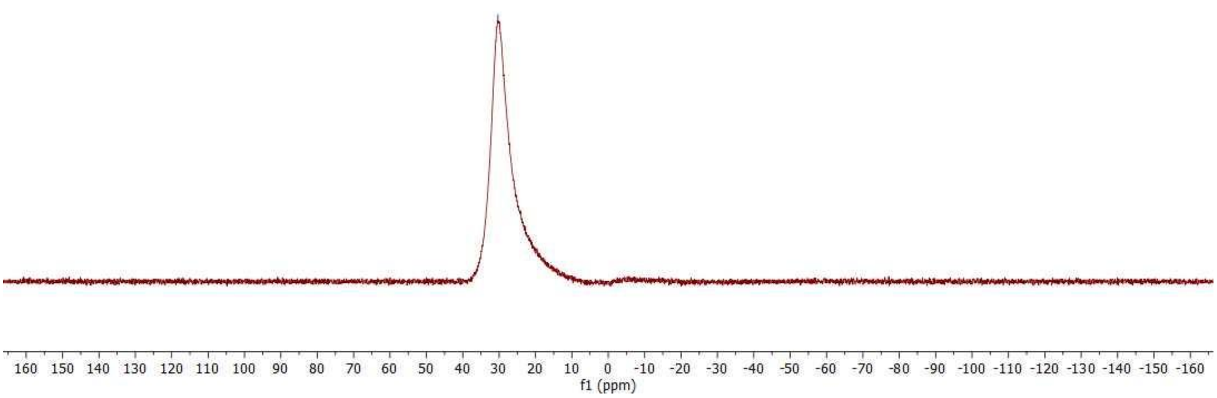

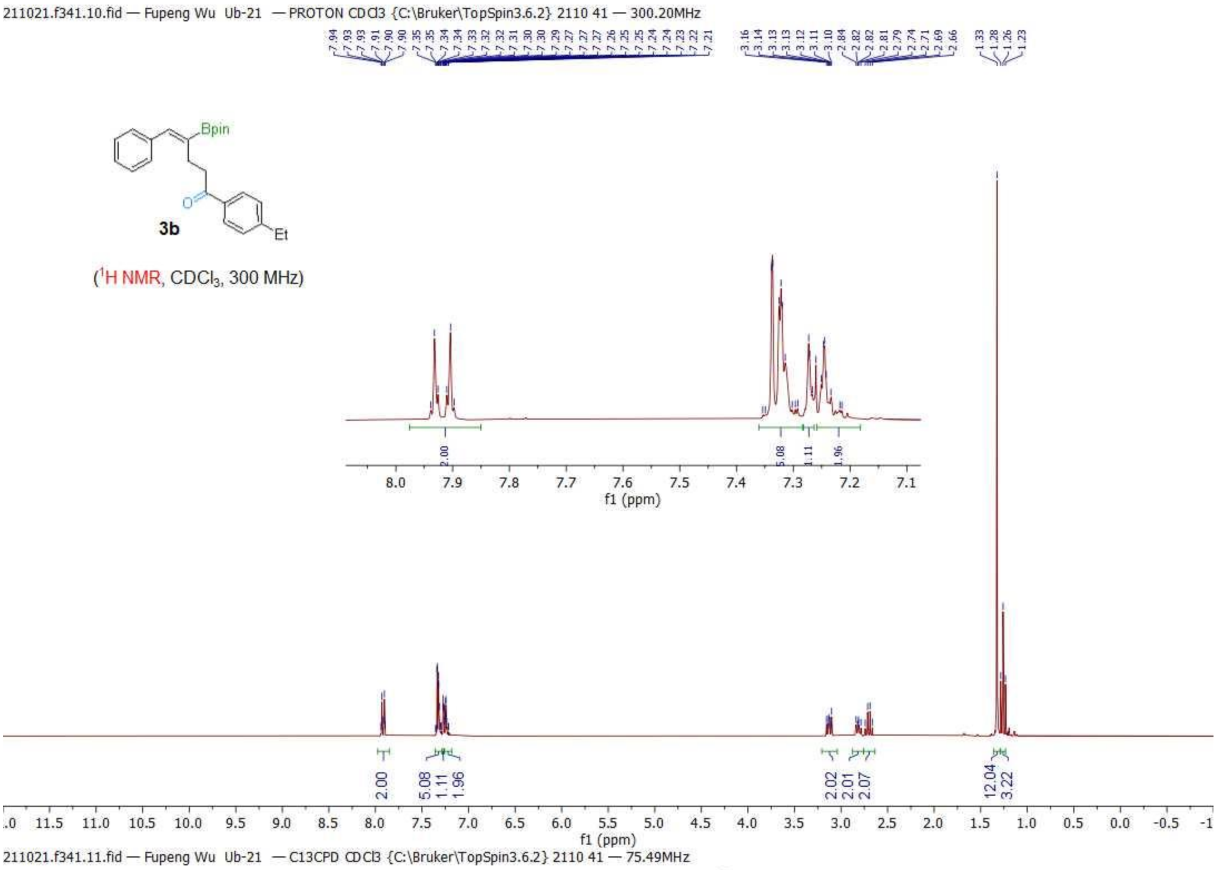
211021.f341.10.fid — Fupeng Wu Ub-21 — PROTON CDCl3 {C:\Bruker\TopSpin3.6.2} 2110 41 — 300.20MHz
Bpin
3b
Et
(1H NMR, CDCl3, 300 MHz)
f1 (ppm)

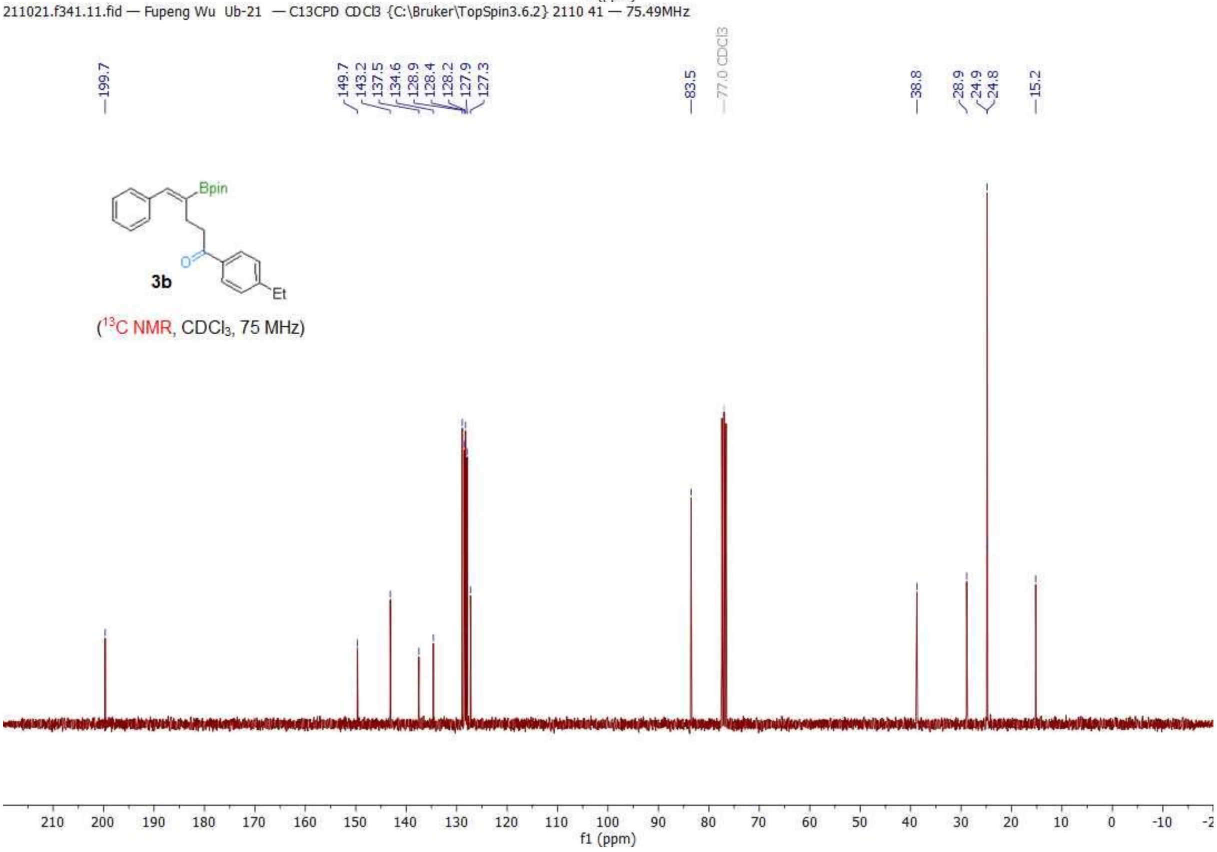
211021.f341.11.fid — Fupeng Wu Ub-21 — C13CPD CDCl3 {C:\Bruker\TopSpin3.6.2} 2110 41 — 75.49MHz
199.7
149.7
143.2
137.5
134.6
128.9
128.4
128.2
127.9
127.3
83.5
77.0 CDCl3
38.8
28.9
24.9
24.8
15.2
Bpin
3b
Et
(13C NMR, CDCl3, 75 MHz)
f1 (ppm)

211021.f341.12.fid — Fupeng Wu Ub-21 — 11B CDCl3 {C:\Bruker\TopSpin3.6.2} 2110 41 — 96.32MHz

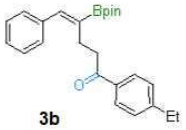

3b

(^{11}B NMR, $CDCl_3$, 96 MHz)

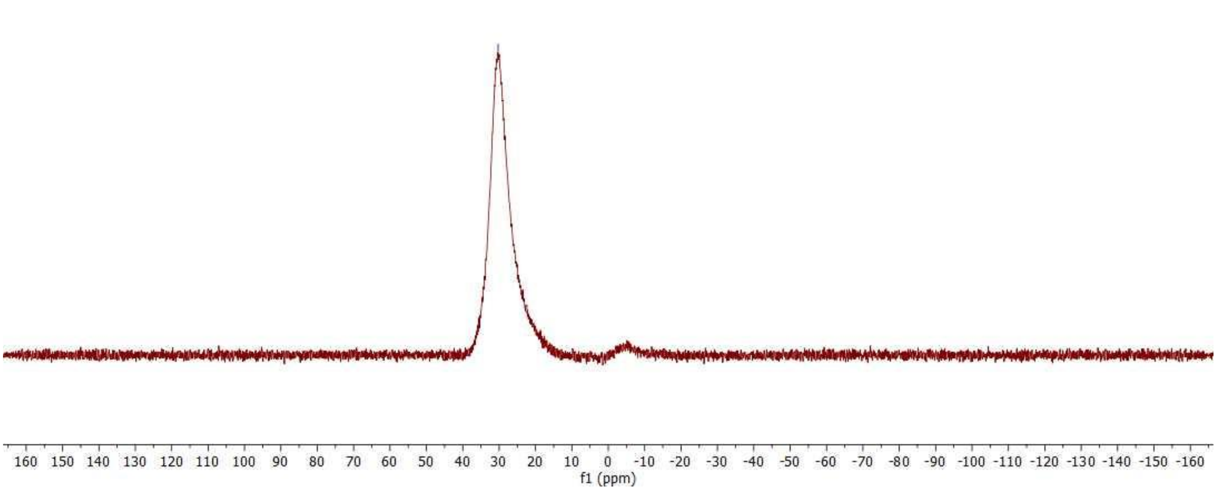

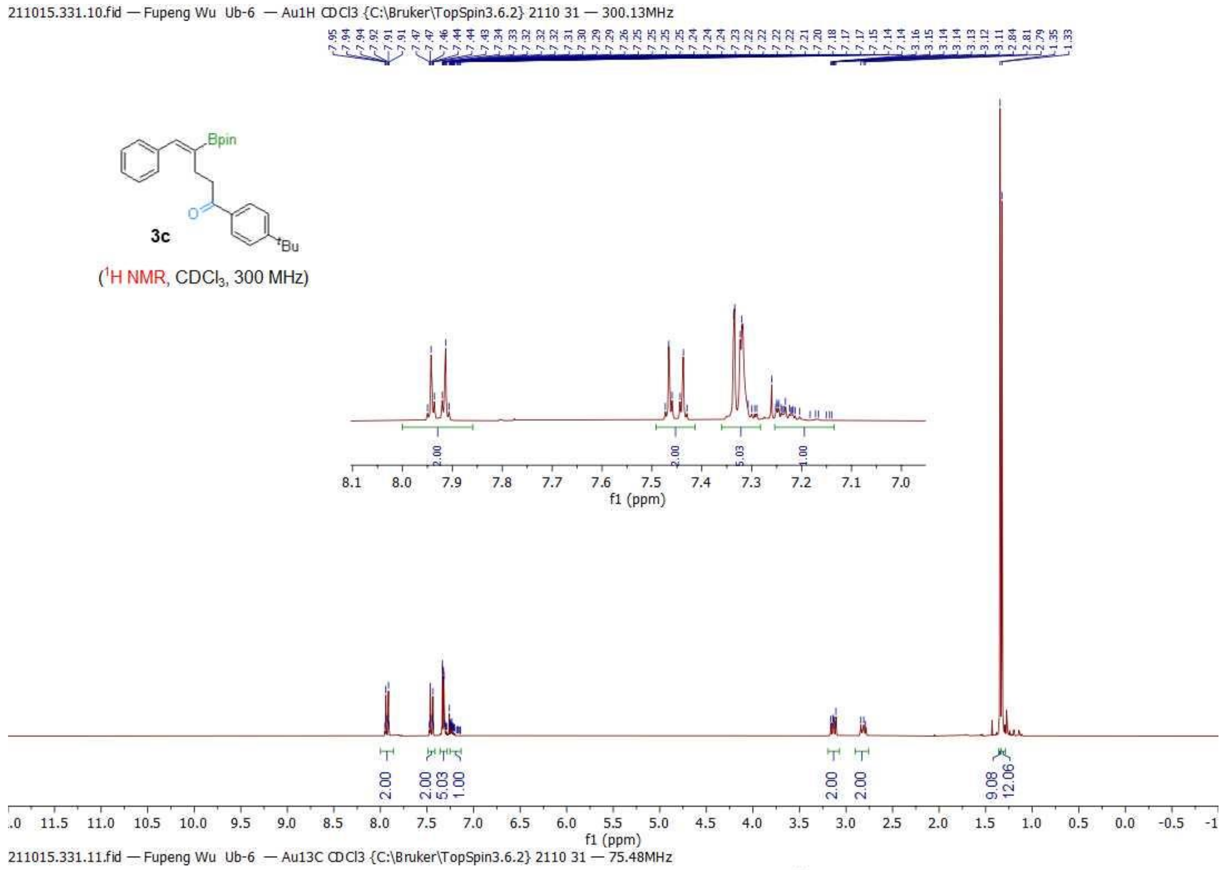
211015.331.10.fid — Fupeng Wu Ub-6 — Au1H CDCl3 {C:\Bruker\TopSpin3.6.2} 2110 31 — 300.13MHz
Bpin
O
3c
tBu
(1H NMR, CDCl3, 300 MHz)
2.00
2.00
5.03
1.00
2.00
2.00
9.08
12.06
f1 (ppm)

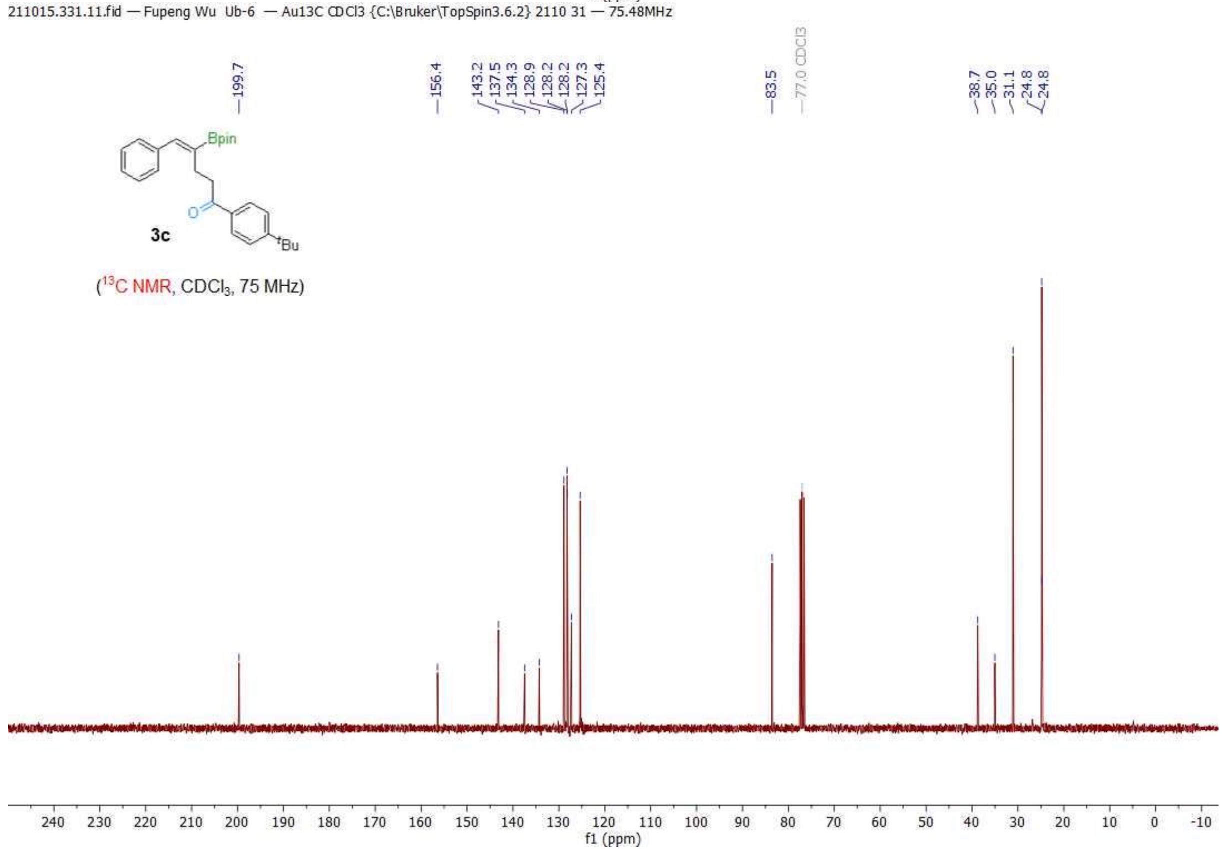
211015.331.11.fid — Fupeng Wu Ub-6 — Au13C CDCl3 {C:\Bruker\TopSpin3.6.2} 2110 31 — 75.48MHz
199.7
156.4
143.2
137.5
134.3
128.9
128.2
128.2
127.3
125.4
83.5
77.0 CDCl3
38.7
35.0
31.1
24.8
24.8
Bpin
O
3c
tBu
(13C NMR, CDCl3, 75 MHz)
f1 (ppm)

211015.331.12.fid — Fupeng Wu Ub-6 — Au11B CDCl3 {C:\Bruker\TopSpin3.6.2} 2110 31 — 96.29MHz

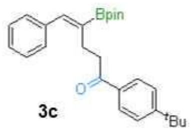

3c

(^{11}B NMR, $CDCl_3$, 96 MHz)

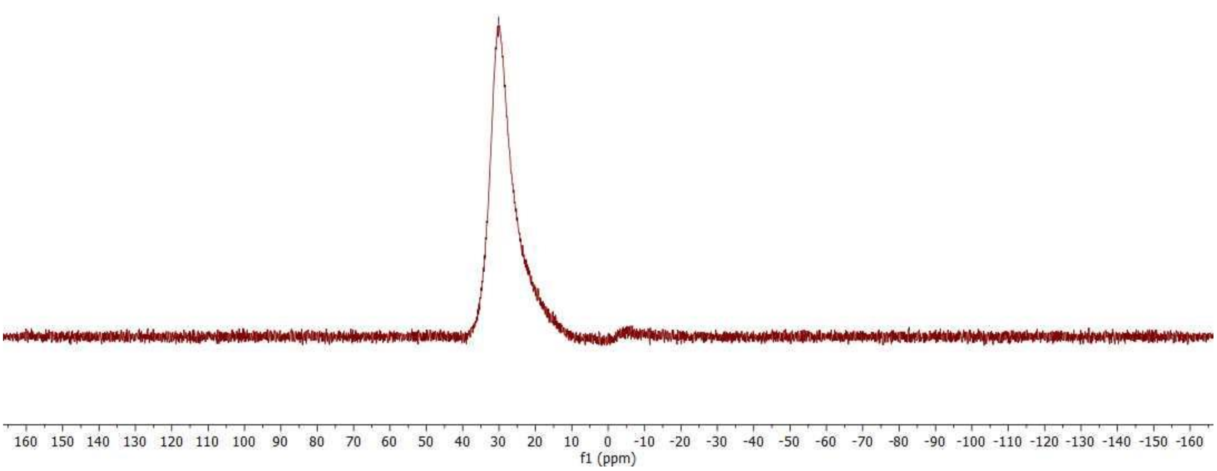

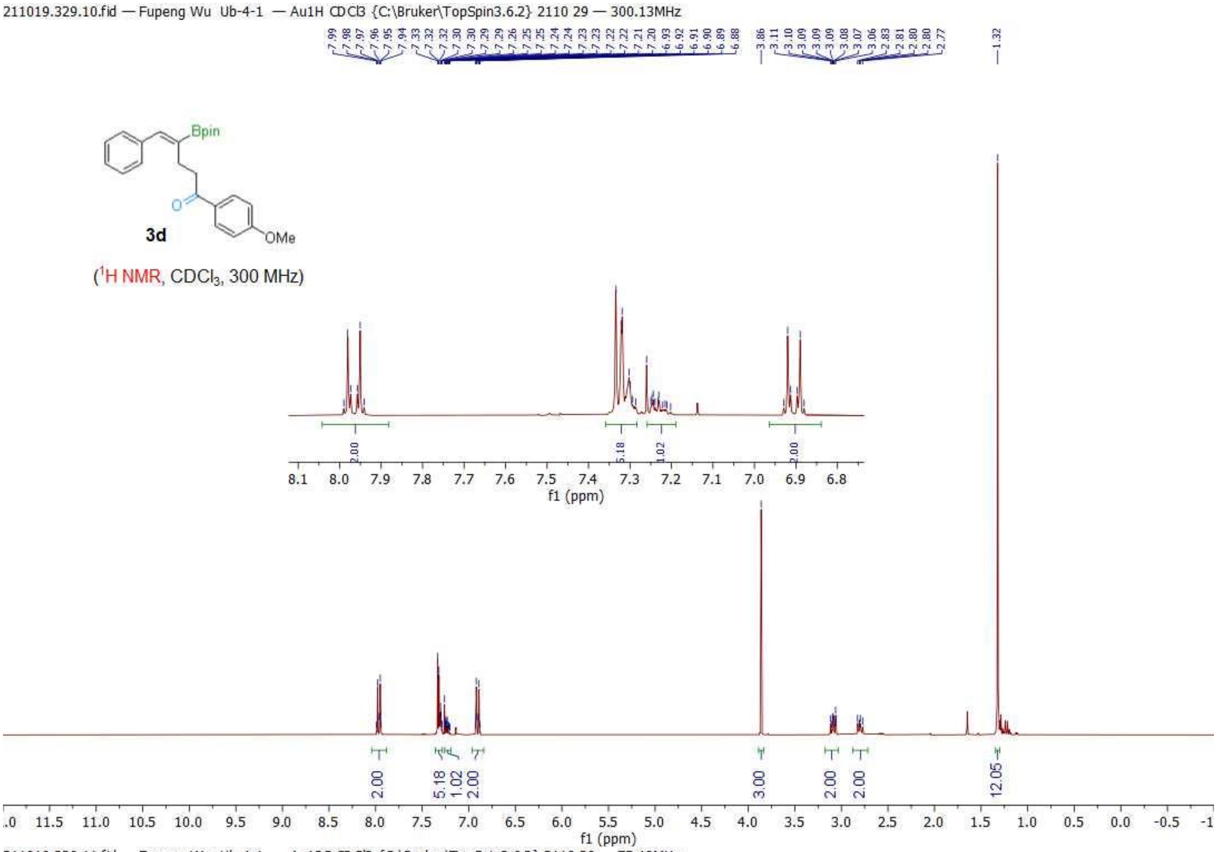

211019.329.10.fid — Fupeng Wu Ub-4-1 — Au1H CDCl3 {C:\Bruker\TopSpin3.6.2} 2110 29 — 300.13MHz
7.99 7.98 7.97 7.96 7.95 7.94 7.33 7.32 7.32 7.30 7.30 7.29 7.29 7.26 7.25 7.25 7.24 7.24 7.23 7.23 7.22 7.22 7.21 7.20 6.93 6.92 6.91 6.90 6.89 6.88
3.86 3.11 3.10 3.09 3.09 3.09 3.08 3.07 3.06 2.83 2.81 2.80 2.80 2.77
1.32
Bpin
O
OMe
3d
(¹H NMR, CDCl₃, 300 MHz)
2.00
5.18
1.02
2.00
3.00
2.00
2.00
12.05
f1 (ppm)

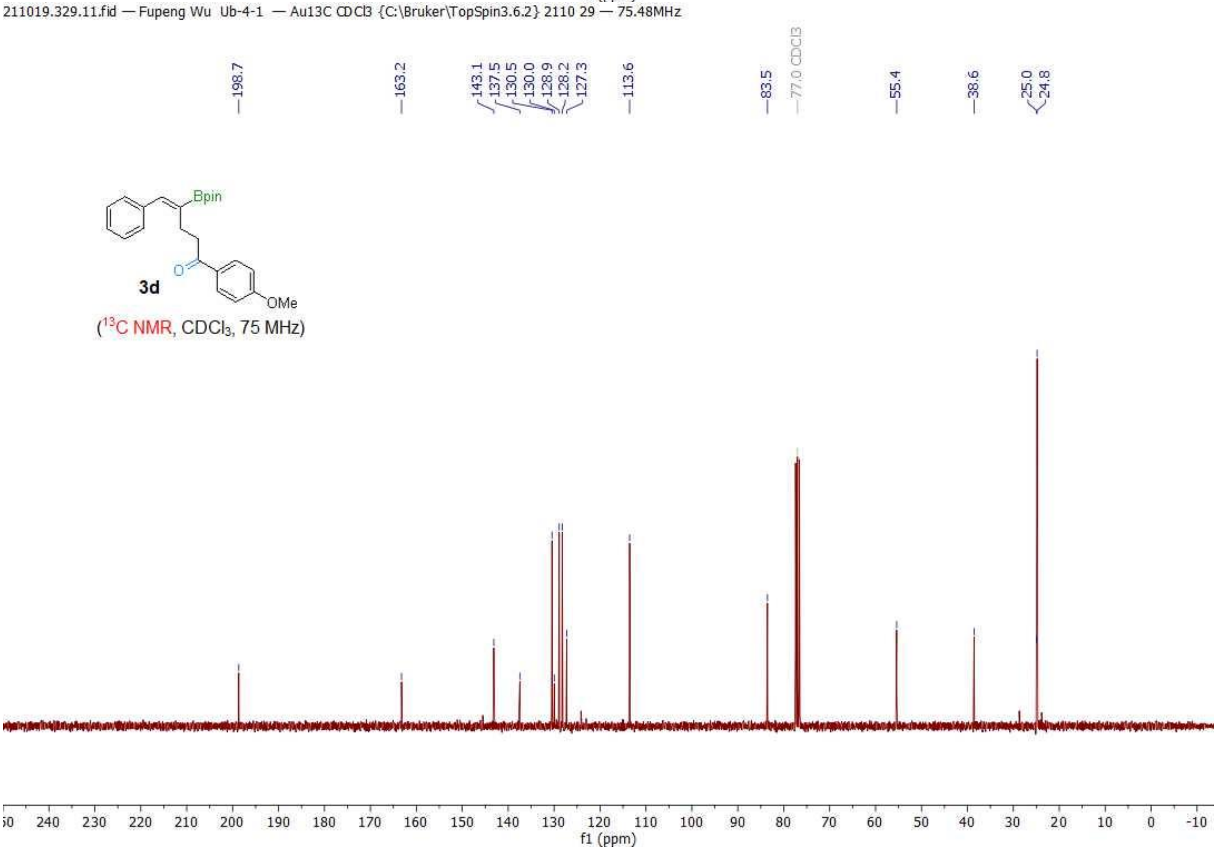

211019.329.11.fid — Fupeng Wu Ub-4-1 — Au13C CDCl3 {C:\Bruker\TopSpin3.6.2} 2110 29 — 75.48MHz
198.7
163.2
143.1 137.5 130.5 130.0 128.9 128.2 127.3
113.6
83.5
77.0 CDCl3
55.4
38.6
25.0 24.8
Bpin
O
OMe
3d
(¹³C NMR, CDCl₃, 75 MHz)
f1 (ppm)

211015.328.12.fid — Fupeng Wu Ub-4 — Au11B CDCl3 {C:\Bruker\TopSpin3.6.2} 2110 28 — 96.29MHz

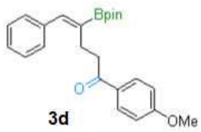

3d

(^{11}B NMR, $CDCl_3$, 96 MHz)

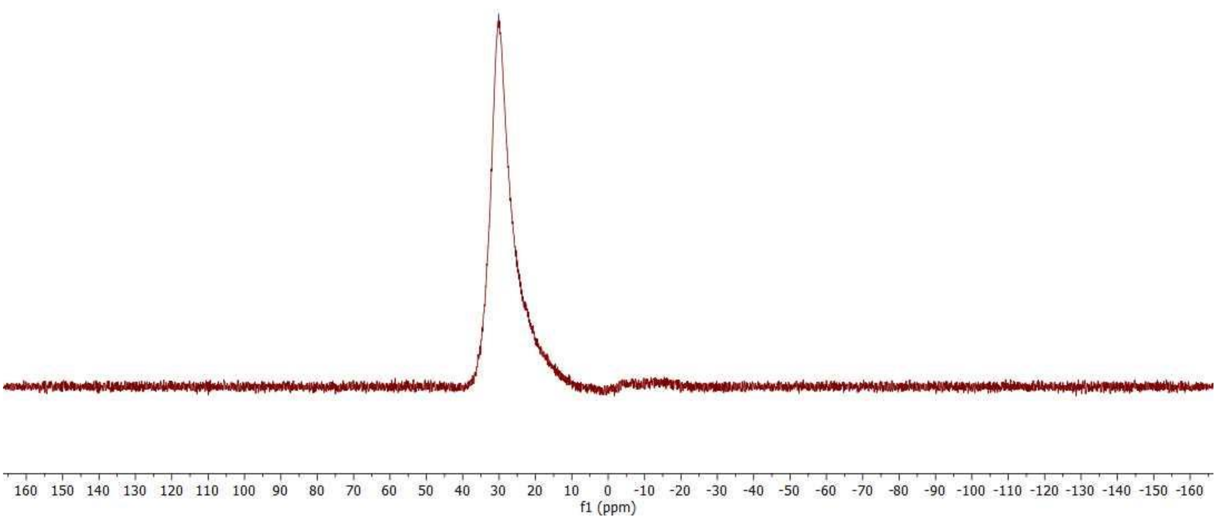

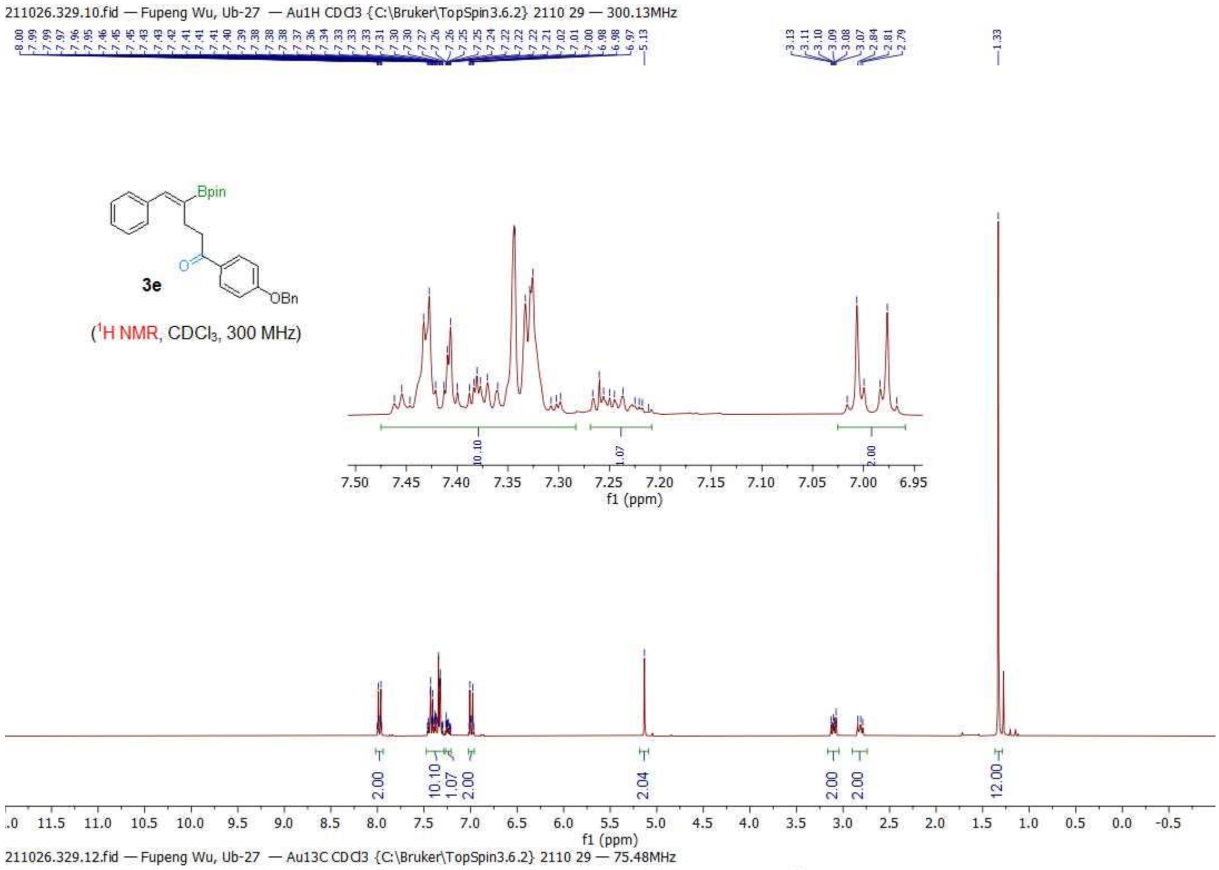

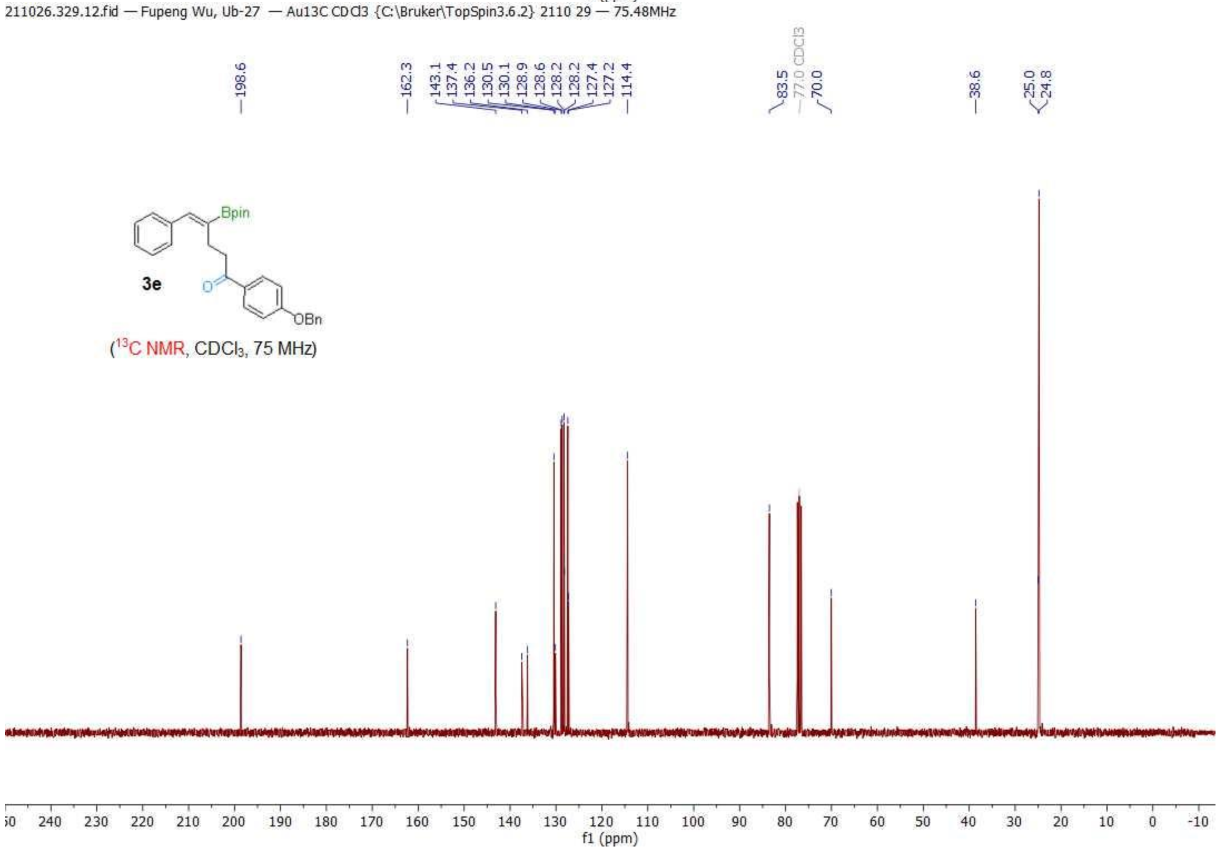

211026.329.11.fid — Fupeng Wu, Ub-27 — Au11B CDCl3 {C:\Bruker\TopSpin3.6.2} 2110 29 — 96.29MHz

—30.1

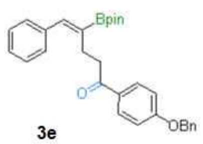

3e

(^{11}B NMR, $CDCl_3$, 96 MHz)

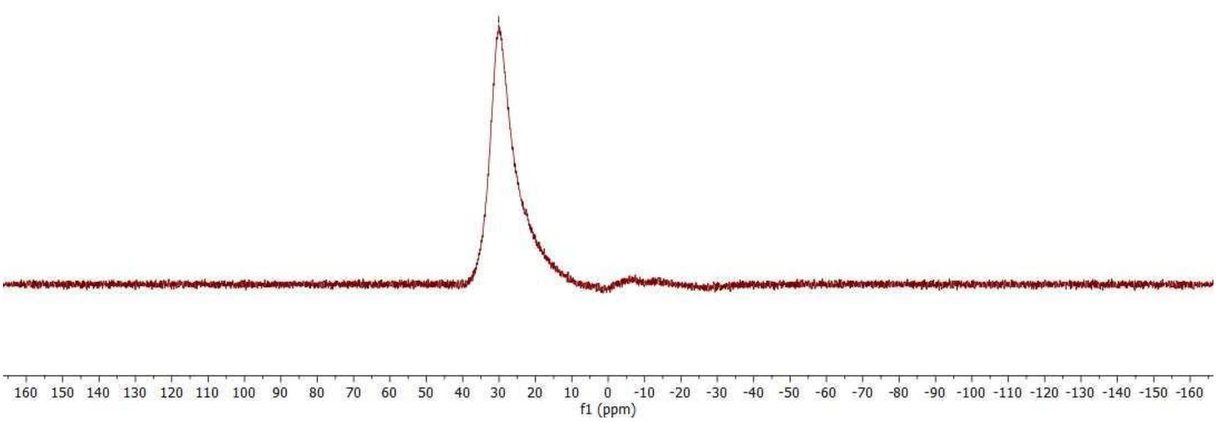

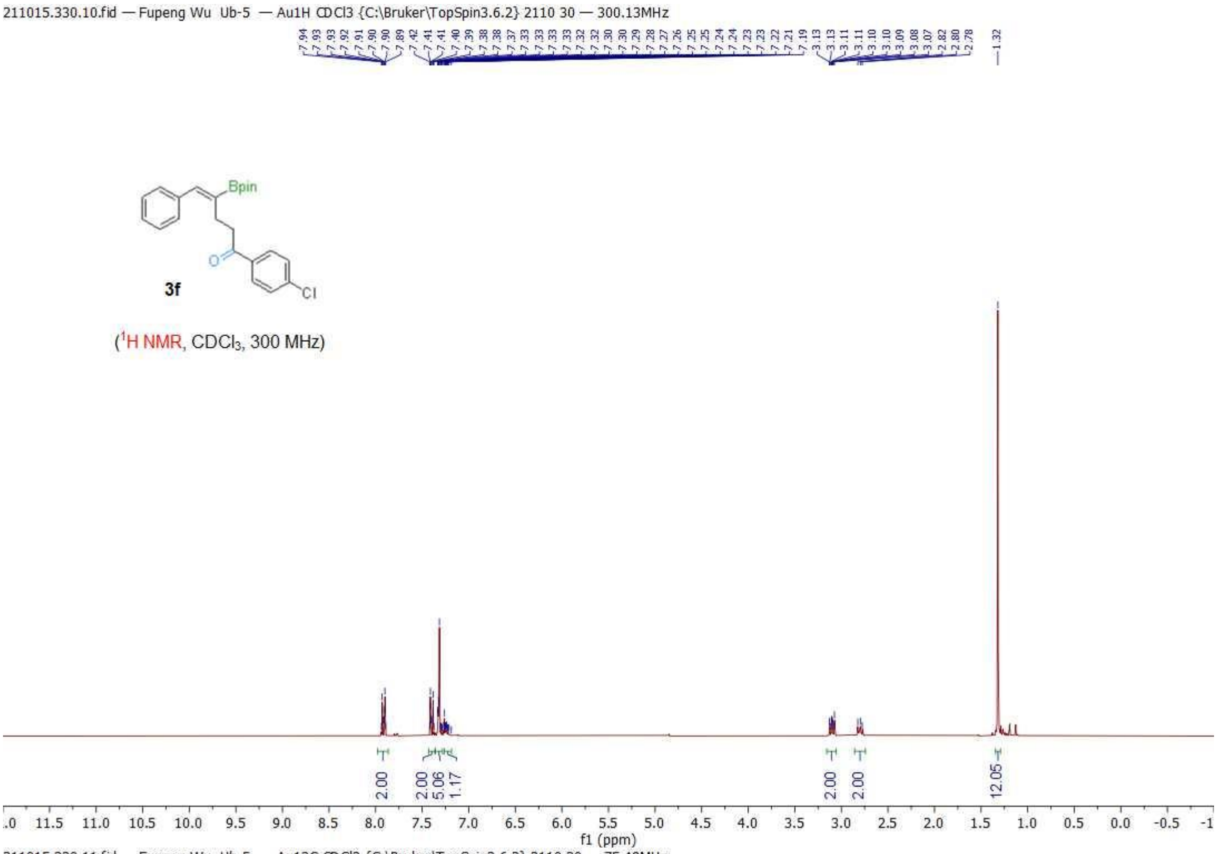
211015.330.10.fid — Fupeng Wu Ub-5 — Au1H CDCl3 {C:\Bruker\TopSpin3.6.2} 2110 30 — 300.13MHz
7.94 7.93 7.93 7.92 7.91 7.90 7.90 7.89 7.42 7.41 7.41 7.40 7.39 7.38 7.38 7.37 7.33 7.33 7.33 7.33 7.32 7.32 7.30 7.29 7.28 7.27 7.26 7.25 7.25 7.24 7.24 7.23 7.23 7.22 7.21 7.19 3.13 3.13 3.11 3.11 3.10 3.10 3.09 3.08 3.07 2.82 2.80 2.78 1.32
Bpin
O
Cl
3f
(^{1}H NMR, $CDCl_3$, 300 MHz)
2.00 2.00 5.06 1.17 2.00 2.00 12.05
f1 (ppm)

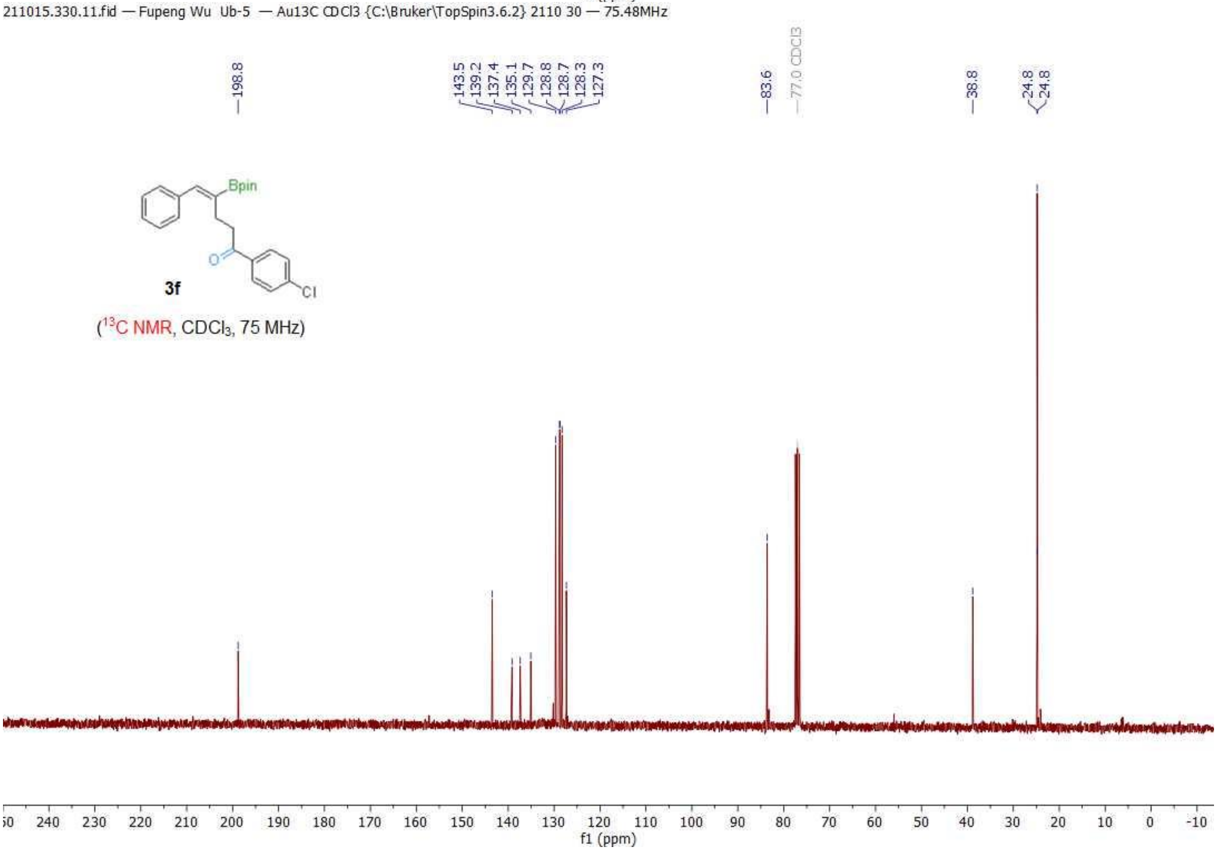
211015.330.11.fid — Fupeng Wu Ub-5 — Au13C CDCl3 {C:\Bruker\TopSpin3.6.2} 2110 30 — 75.48MHz
198.8
143.5 139.2 137.4 135.1 129.7 128.8 128.7 128.3 127.3
83.6
77.0 CDCl3
38.8
24.8 24.8
Bpin
O
Cl
3f
(^{13}C NMR, $CDCl_3$, 75 MHz)
f1 (ppm)

211015.330.12.fid — Fupeng Wu Ub-5 — Au11B CDCl3 {C:\Bruker\TopSpin3.6.2} 2110 30 — 96.29MHz

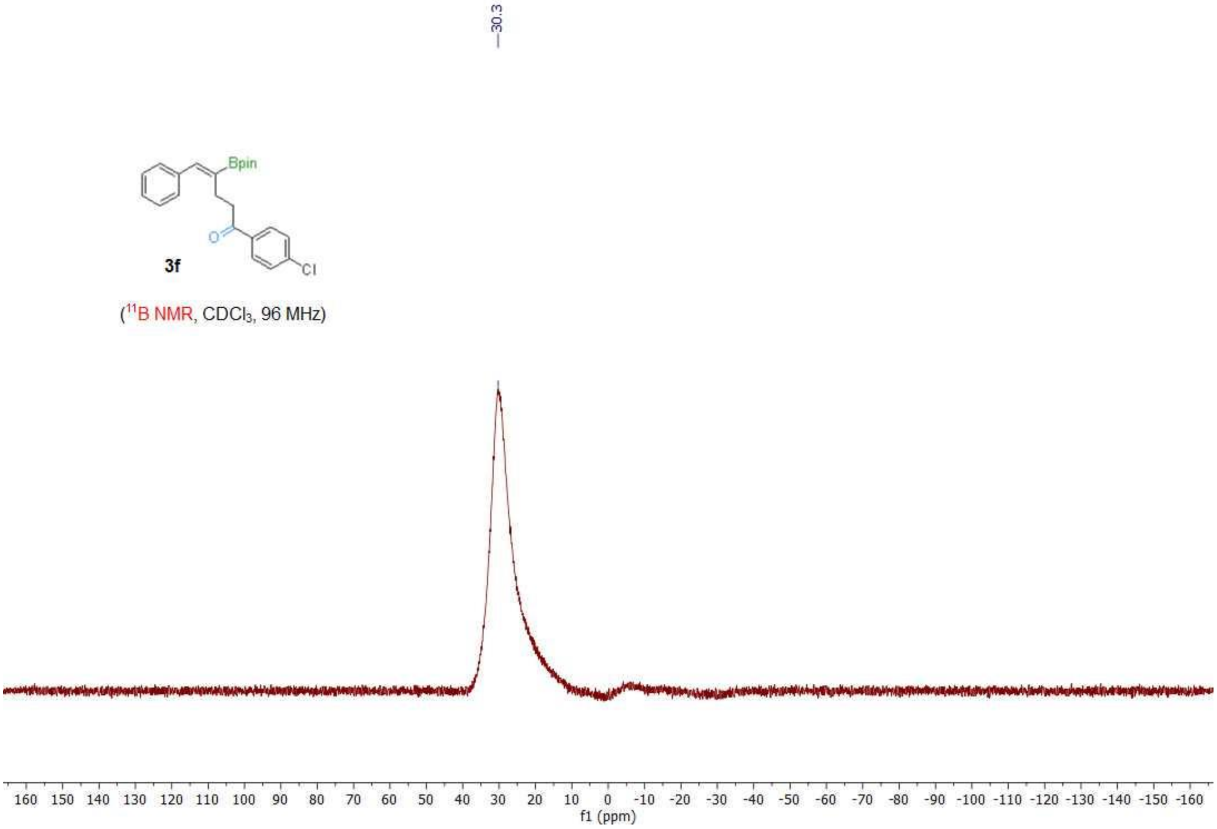

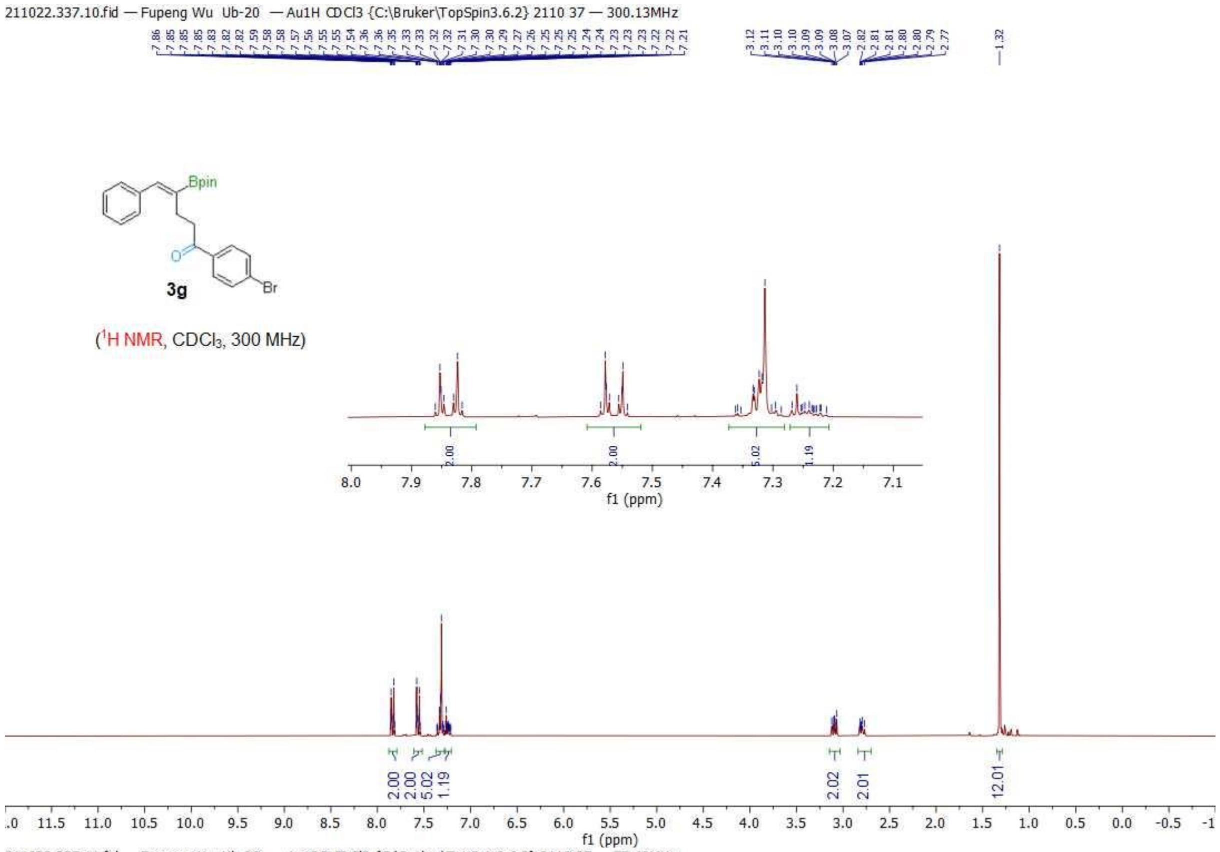
211022.337.10.fid — Fupeng Wu Ub-20 — Au1H CDCl3 {C:\Bruker\TopSpin3.6.2} 2110 37 — 300.13MHz
7.86 7.85 7.85 7.83 7.82 7.82 7.59 7.58 7.57 7.56 7.55 7.55 7.54 7.36 7.36 7.35 7.33 7.33 7.32 7.32 7.31 7.30 7.30 7.29 7.27 7.26 7.25 7.25 7.25 7.24 7.24 7.23 7.23 7.23 7.22 7.22 7.21
3.12 3.11 3.10 3.10 3.09 3.09 3.08 3.07 2.82 2.81 2.81 2.80 2.80 2.79 2.77
1.32
Bpin
O
Br
3g
(1H NMR, CDCl3, 300 MHz)
2.00 2.00 5.02 1.19 2.02 2.01 12.01
f1 (ppm)

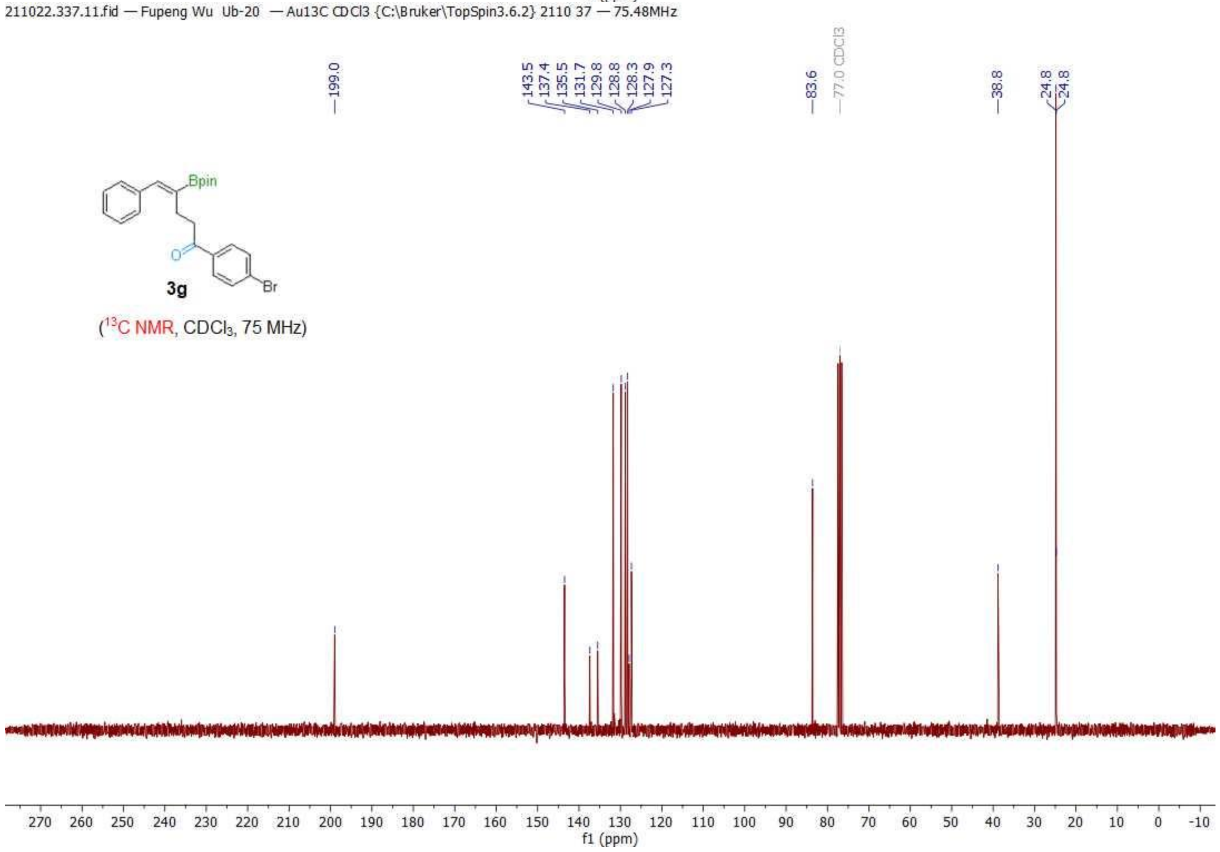
211022.337.11.fid — Fupeng Wu Ub-20 — Au13C CDCl3 {C:\Bruker\TopSpin3.6.2} 2110 37 — 75.48MHz
199.0
143.5 137.4 135.5 131.7 129.8 128.8 128.3 127.9 127.3
83.6
77.0 CDCl3
38.8
24.8 24.8
Bpin
O
Br
3g
(13C NMR, CDCl3, 75 MHz)
f1 (ppm)

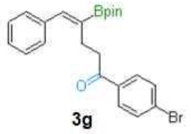

3g

(^{11}B NMR, $CDCl_3$, 96 MHz)

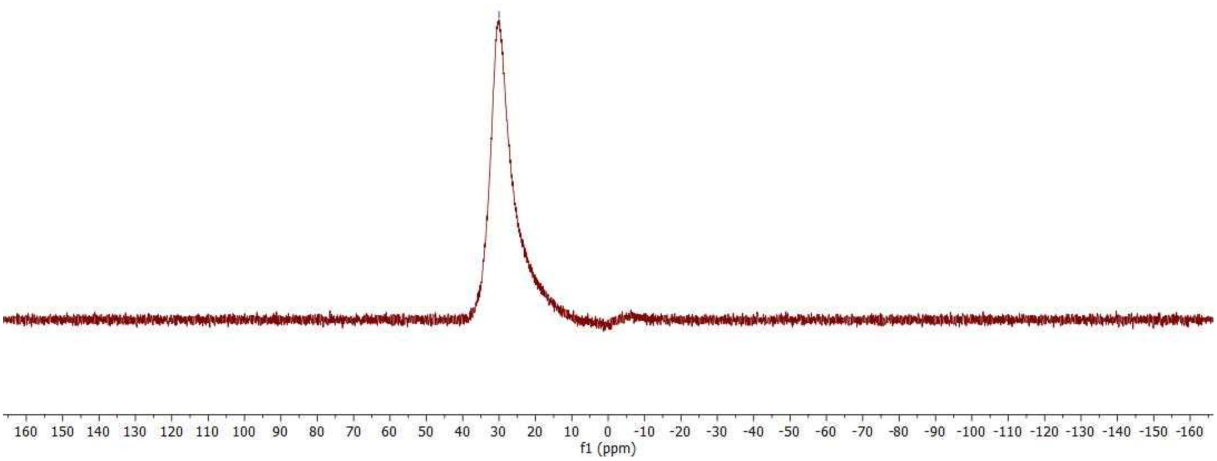

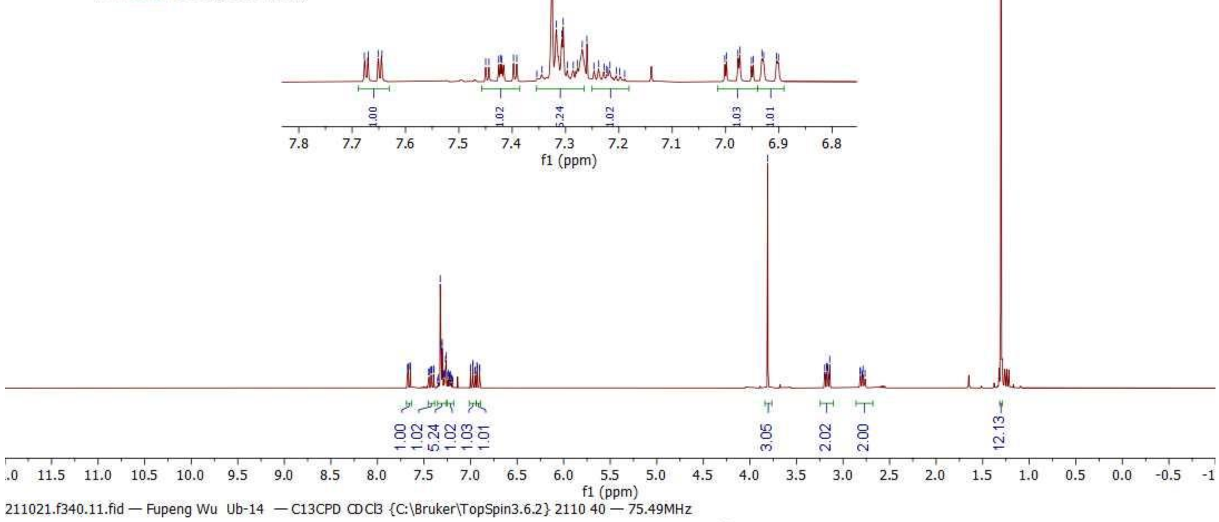

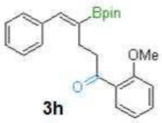

3h

(^{1}H NMR, $CDCl_3$, 300 MHz)

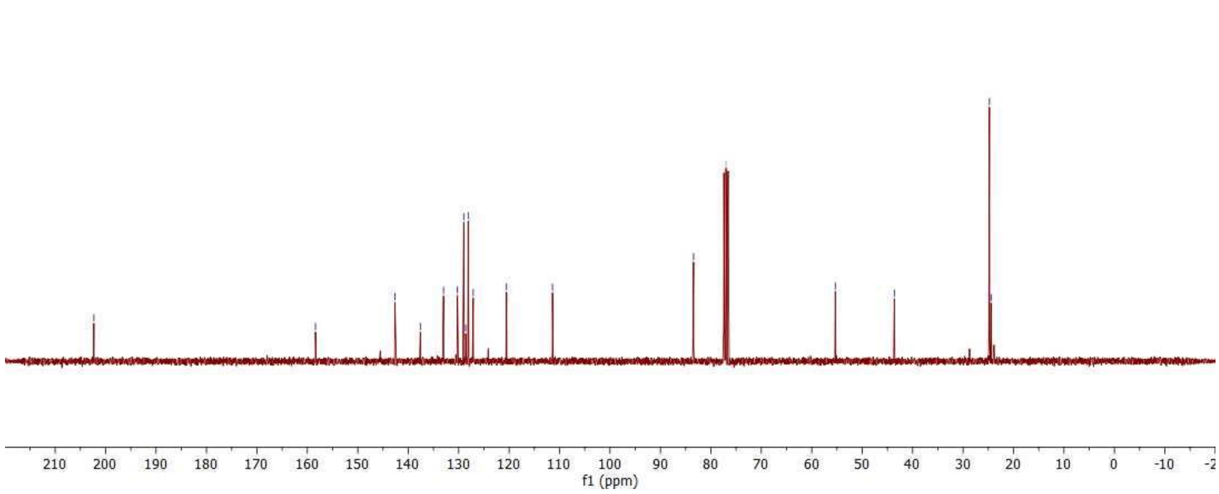

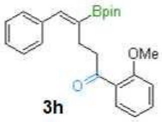

3h

(^{13}C NMR, $CDCl_3$, 75 MHz)

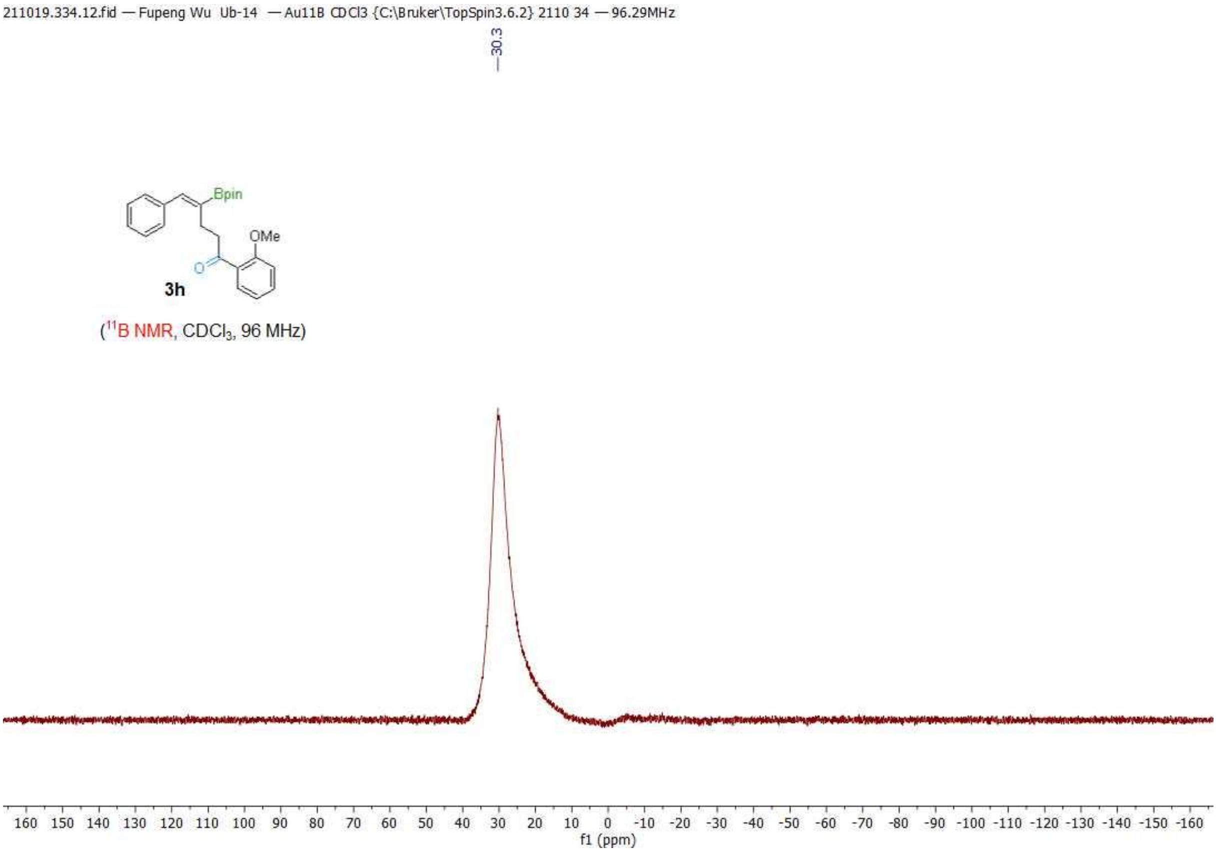
211019.334.12.fid — Fupeng Wu Ub-14 — Au11B CDCl3 {C:\Bruker\TopSpin3.6.2} 2110 34 — 96.29MHz
30.3
Bpin
OMe
O
3h
(11B NMR, CDCl3, 96 MHz)
160 150 140 130 120 110 100 90 80 70 60 50 40 30 20 10 0 -10 -20 -30 -40 -50 -60 -70 -80 -90 -100 -110 -120 -130 -140 -150 -160
f1 (ppm)

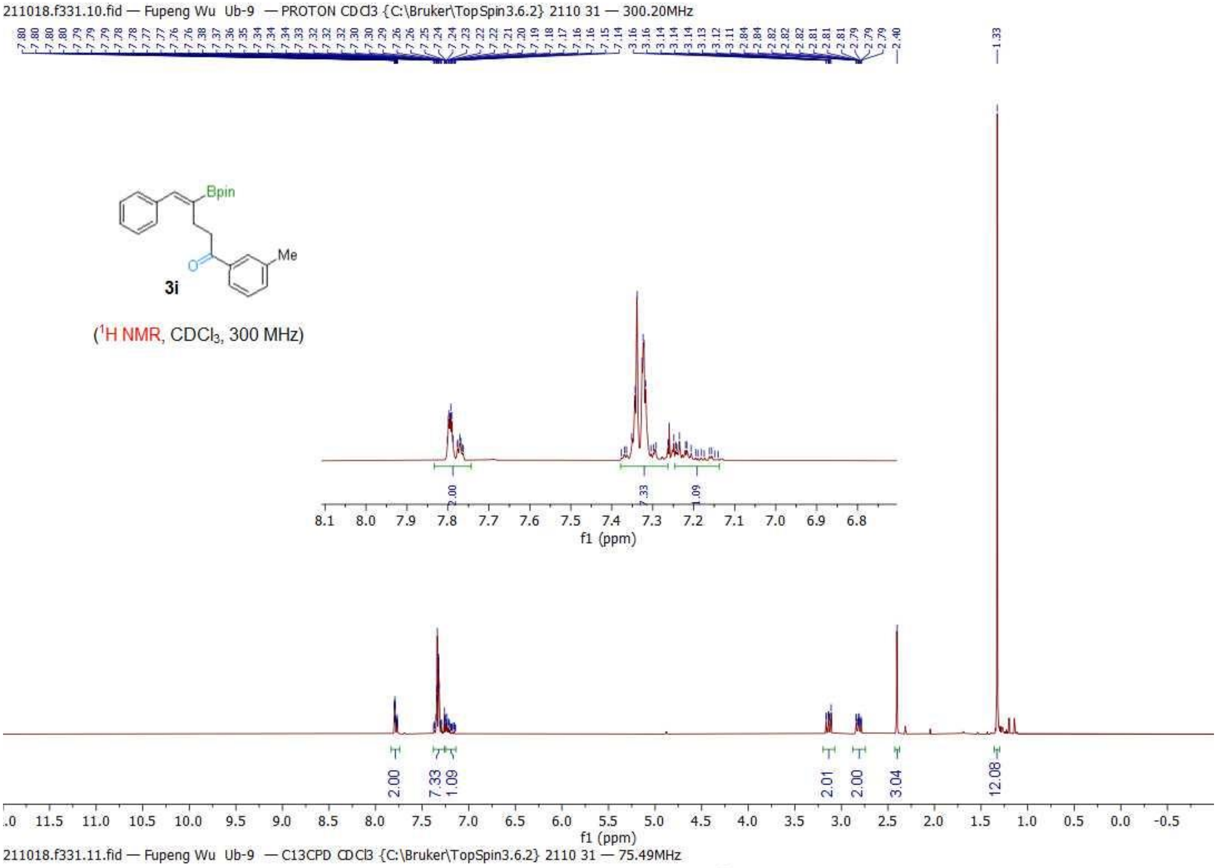
211018.f331.10.fid — Fupeng Wu Ub-9 — PROTON CDCl3 {C:\Bruker\TopSpin3.6.2} 2110 31 — 300.20MHz
Bpin
Me
O
3i
(¹H NMR, CDCl₃, 300 MHz)
f1 (ppm)

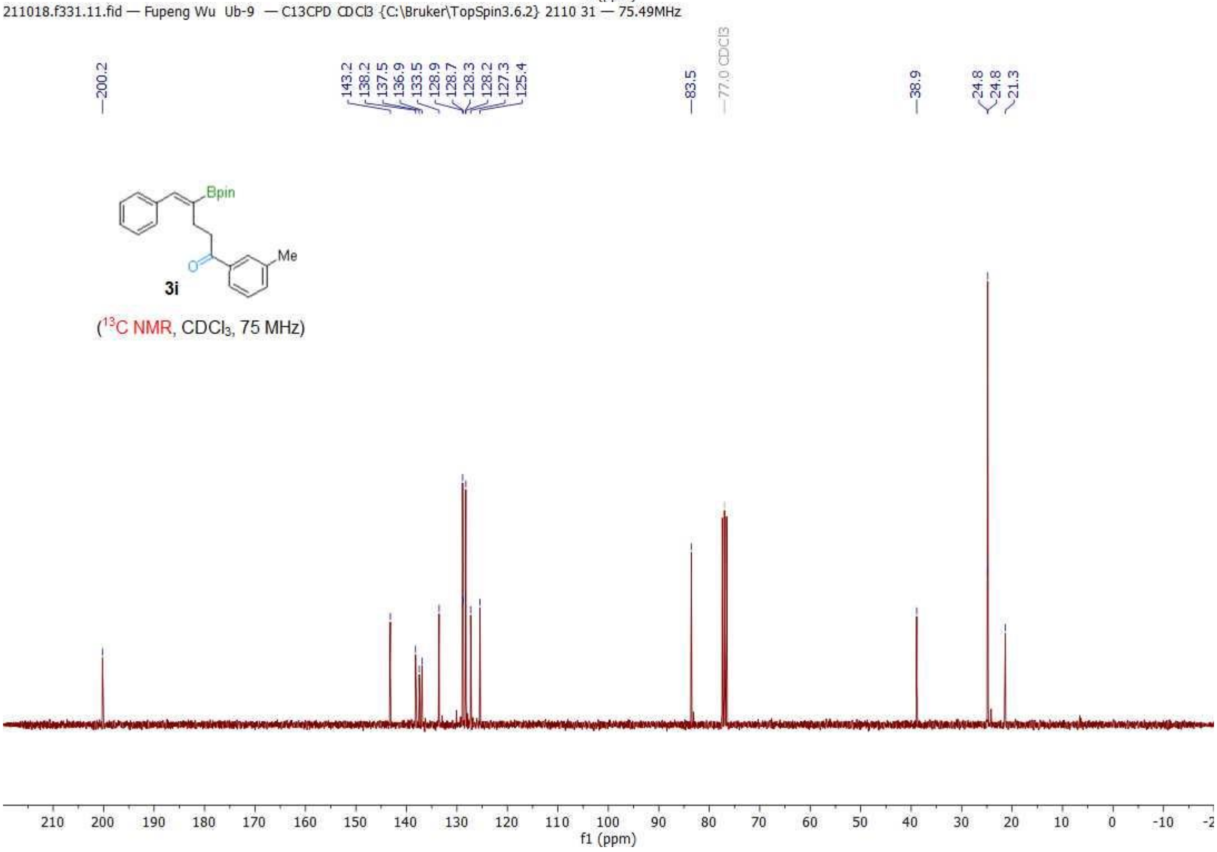
211018.f331.11.fid — Fupeng Wu Ub-9 — C13CPD CDCl3 {C:\Bruker\TopSpin3.6.2} 2110 31 — 75.49MHz
200.2
143.2
138.2
137.5
136.9
133.5
128.9
128.7
128.3
128.2
127.3
125.4
83.5
77.0 CDCl3
38.9
24.8
24.8
21.3
Bpin
Me
O
3i
(¹³C NMR, CDCl₃, 75 MHz)
f1 (ppm)

211018.f331.12.fid — Fupeng Wu Ub-9 — 11B CDCl3 {C:\Bruker\TopSpin3.6.2} 2110 31 — 96.32MHz

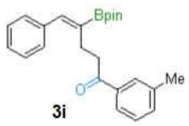

3i

(^{11}B NMR, $CDCl_3$, 96 MHz)

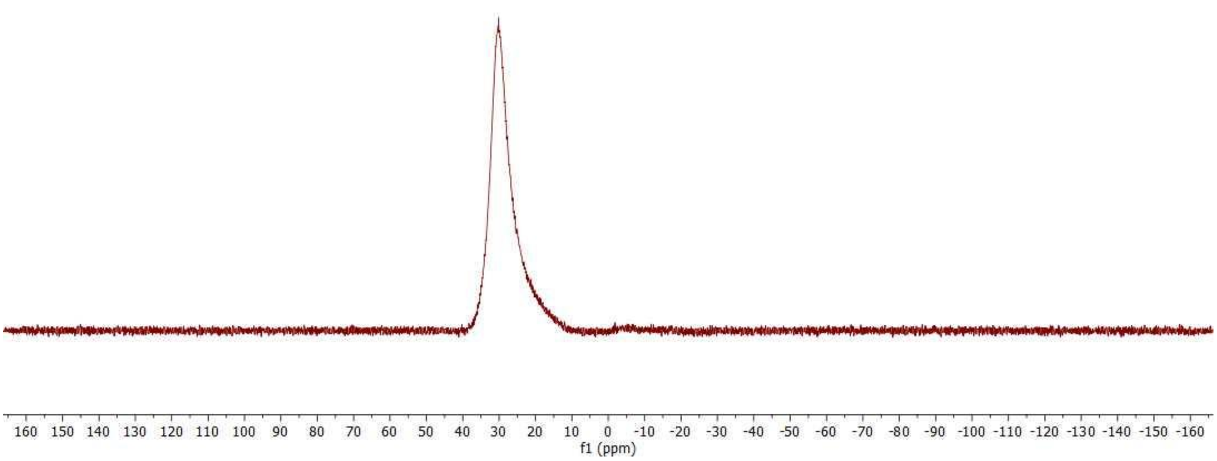

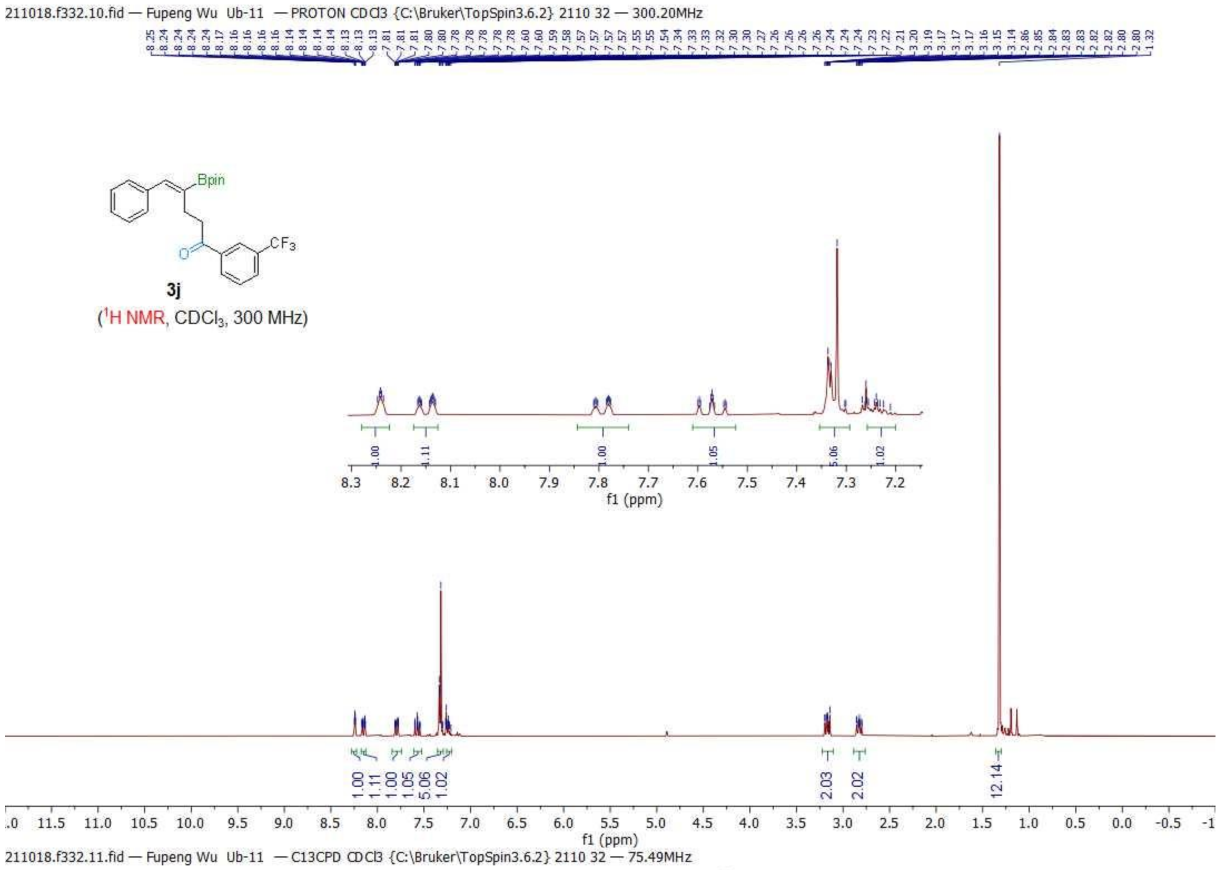
211018.f332.10.fid — Fupeng Wu Ub-11 — PROTON CDCl3 {C:\Bruker\TopSpin3.6.2} 2110 32 — 300.20MHz
Bpin
O
CF3
3j
(1H NMR, CDCl3, 300 MHz)
1.00
1.11
1.00
1.05
5.06
1.02
2.03
2.02
12.14
f1 (ppm)

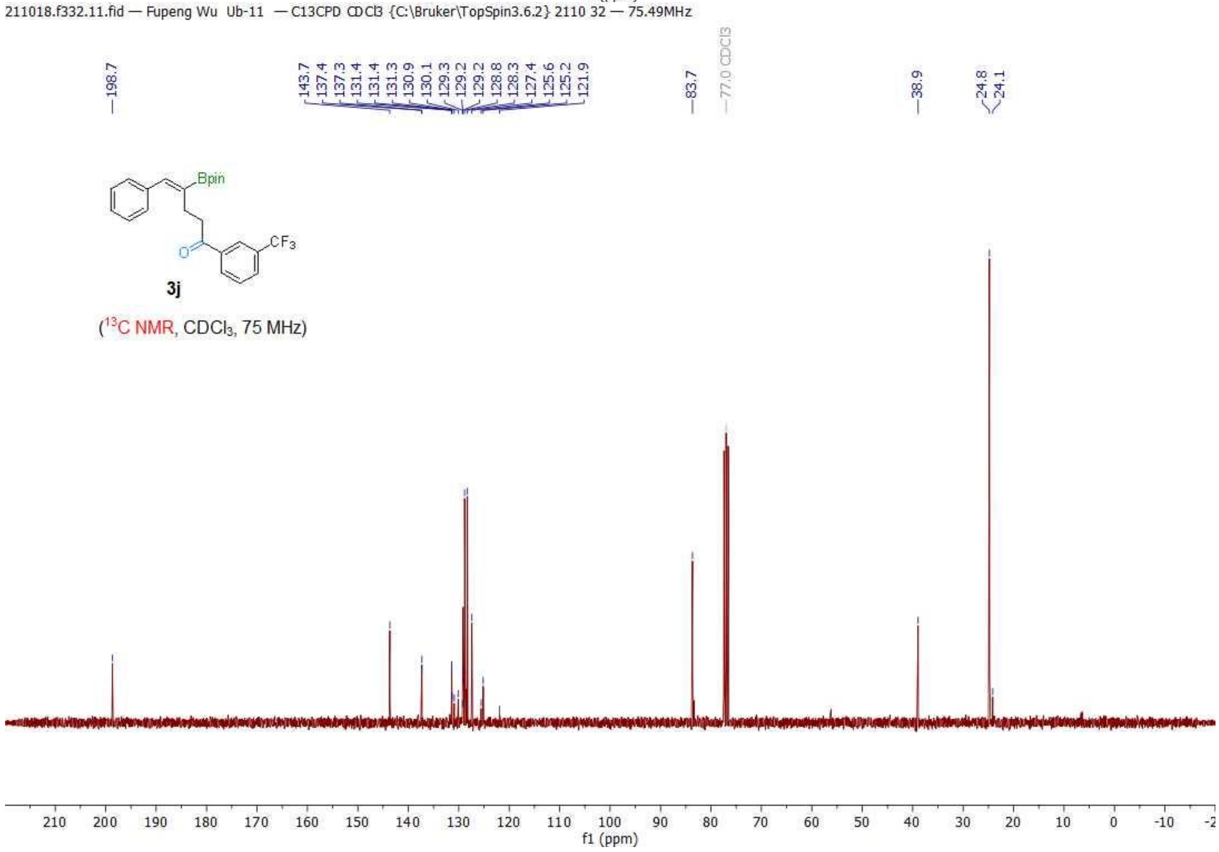
211018.f332.11.fid — Fupeng Wu Ub-11 — C13CPD CDCl3 {C:\Bruker\TopSpin3.6.2} 2110 32 — 75.49MHz
198.7
143.7
137.4
137.3
131.4
131.4
131.3
130.9
130.1
129.3
129.2
129.2
128.8
128.3
127.4
125.6
125.2
121.9
83.7
77.0 CDCl3
38.9
24.8
24.1
Bpin
O
CF3
3j
(13C NMR, CDCl3, 75 MHz)
f1 (ppm)

211018.f332.12.fid — Fupeng Wu Ub-11 — 11B CDCl3 {C:\Bruker\TopSpin3.6.2} 2110 32 — 96.32MHz

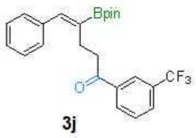

3j

(^{11}B NMR, $CDCl_3$, 96 MHz)

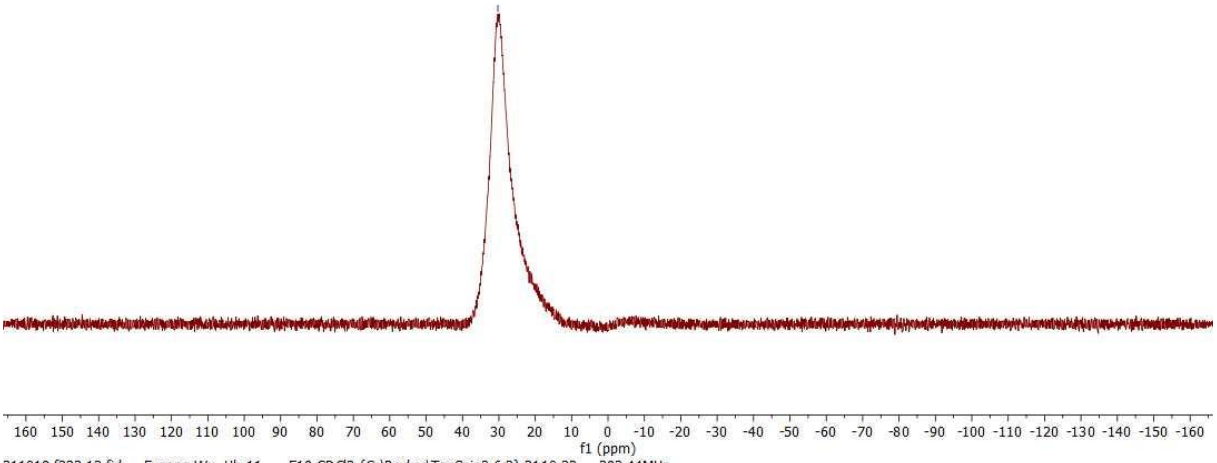

211018.f332.13.fid — Fupeng Wu Ub-11 — F19 CDCl3 {C:\Bruker\TopSpin3.6.2} 2110 32 — 282.44MHz

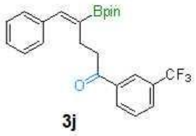

3j

(^{19}F NMR, $CDCl_3$, 282 MHz)

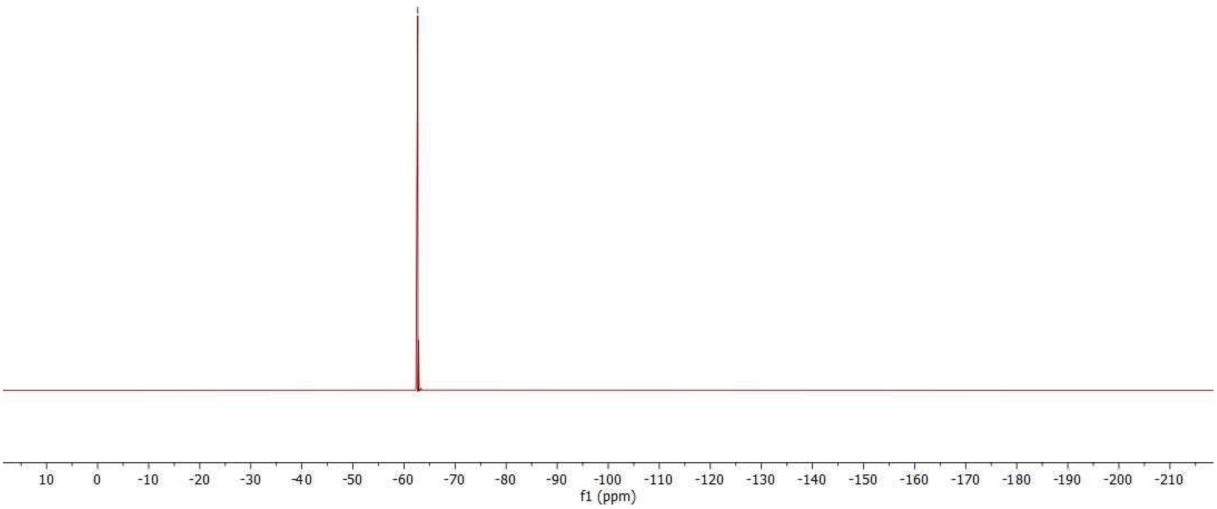

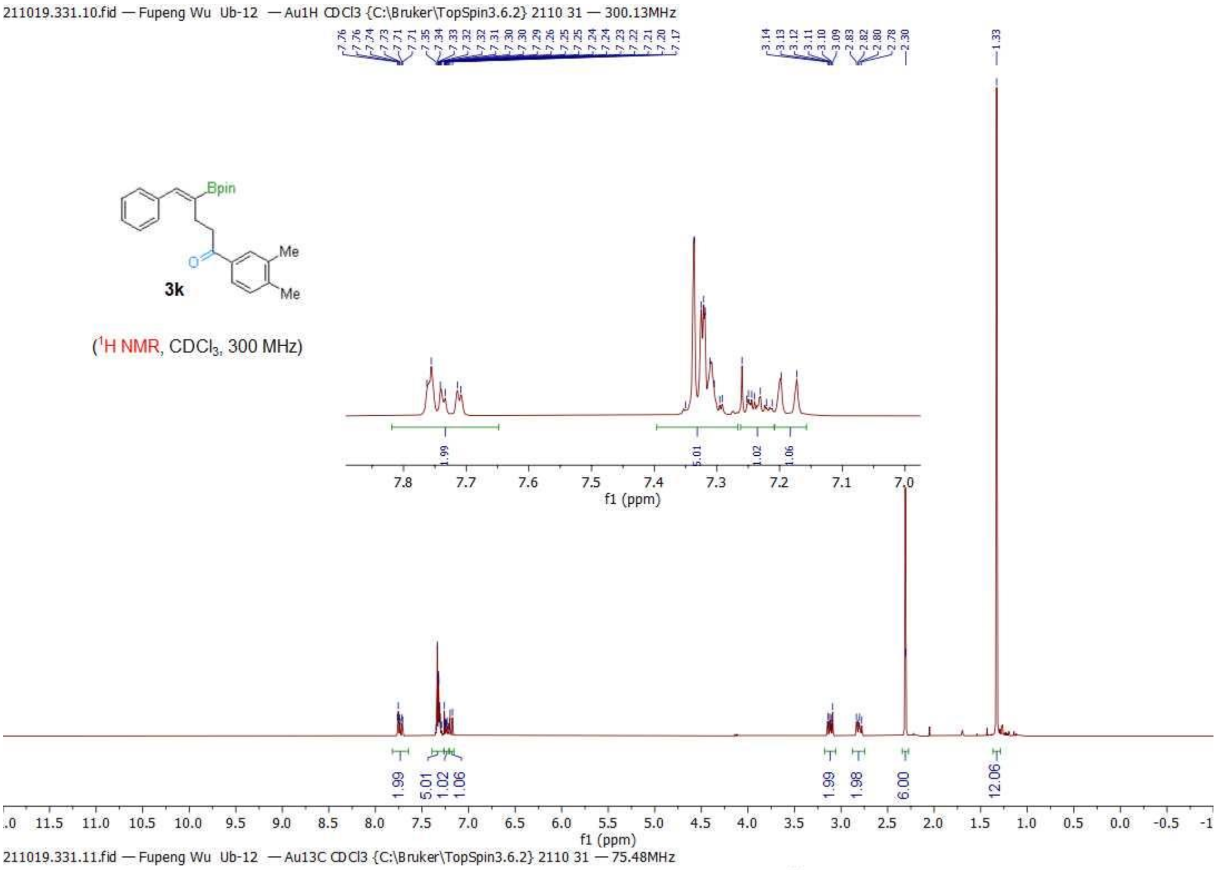

211019.331.10.fid — Fupeng Wu Ub-12 — Au1H CDCl3 {C:\Bruker\TopSpin3.6.2} 2110 31 — 300.13MHz
Bpin
O
Me
Me
3k
(1H NMR, CDCl3, 300 MHz)
f1 (ppm)

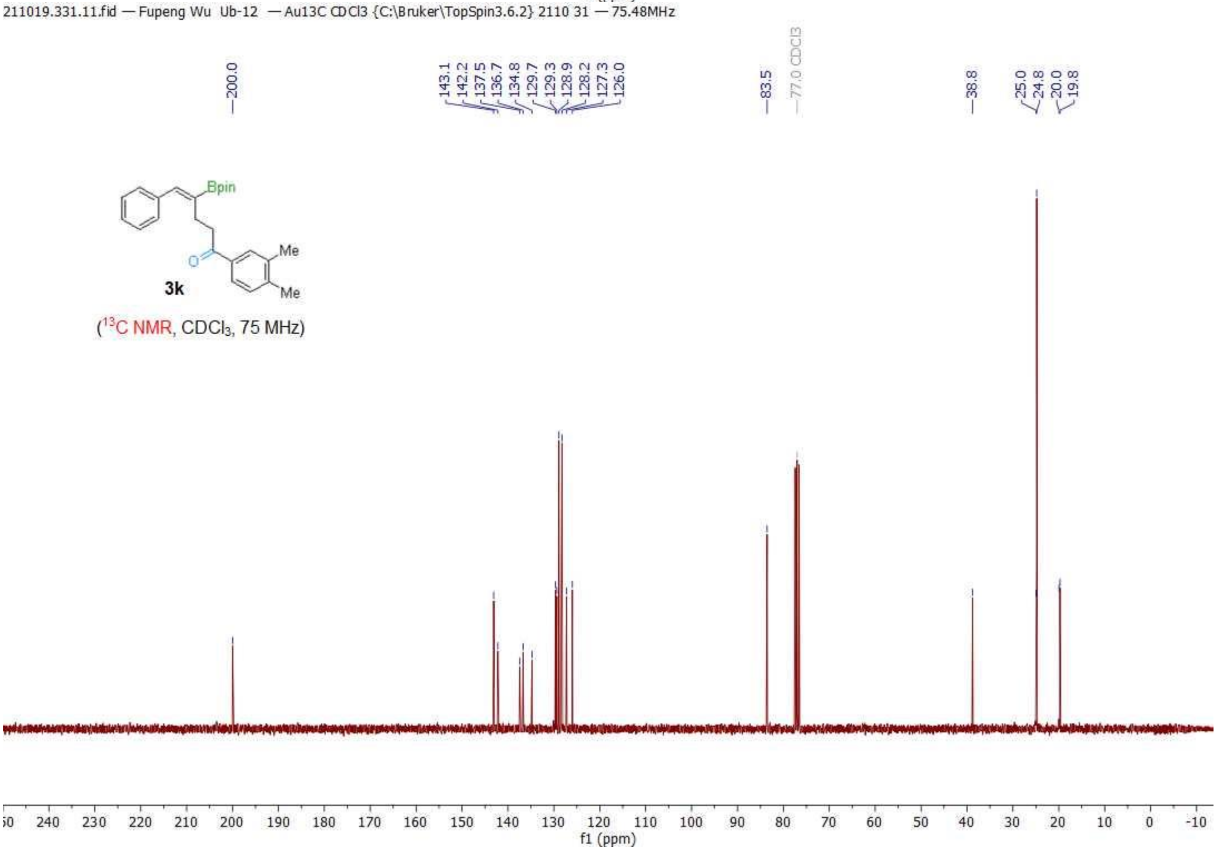

211019.331.11.fid — Fupeng Wu Ub-12 — Au13C CDCl3 {C:\Bruker\TopSpin3.6.2} 2110 31 — 75.48MHz
200.0
143.1
142.2
137.5
136.7
134.8
129.7
129.3
128.9
128.2
127.3
126.0
83.5
77.0 CDCl3
38.8
25.0
24.8
20.0
19.8
Bpin
O
Me
Me
3k
(13C NMR, CDCl3, 75 MHz)
f1 (ppm)

211019.331.12.fid — Fupeng Wu Ub-12 — Au11B CDCl3 {C:\Bruker\TopSpin3.6.2} 2110 31 — 96.29MHz

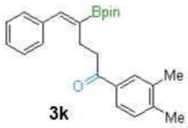

3k

(^{11}B NMR, $CDCl_3$, 96 MHz)

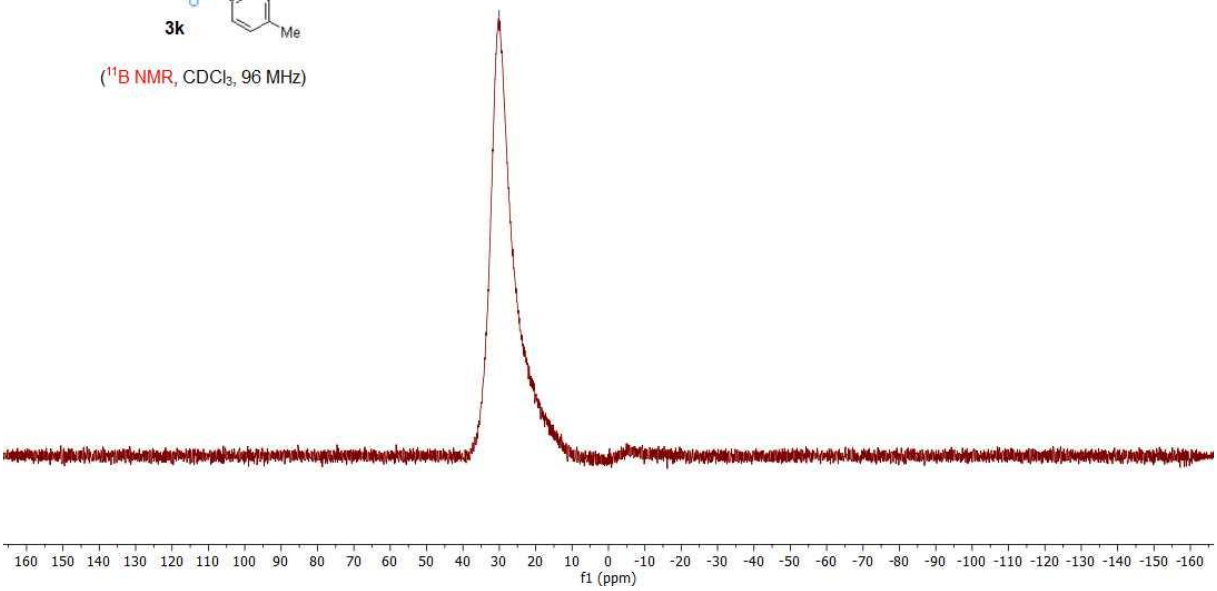

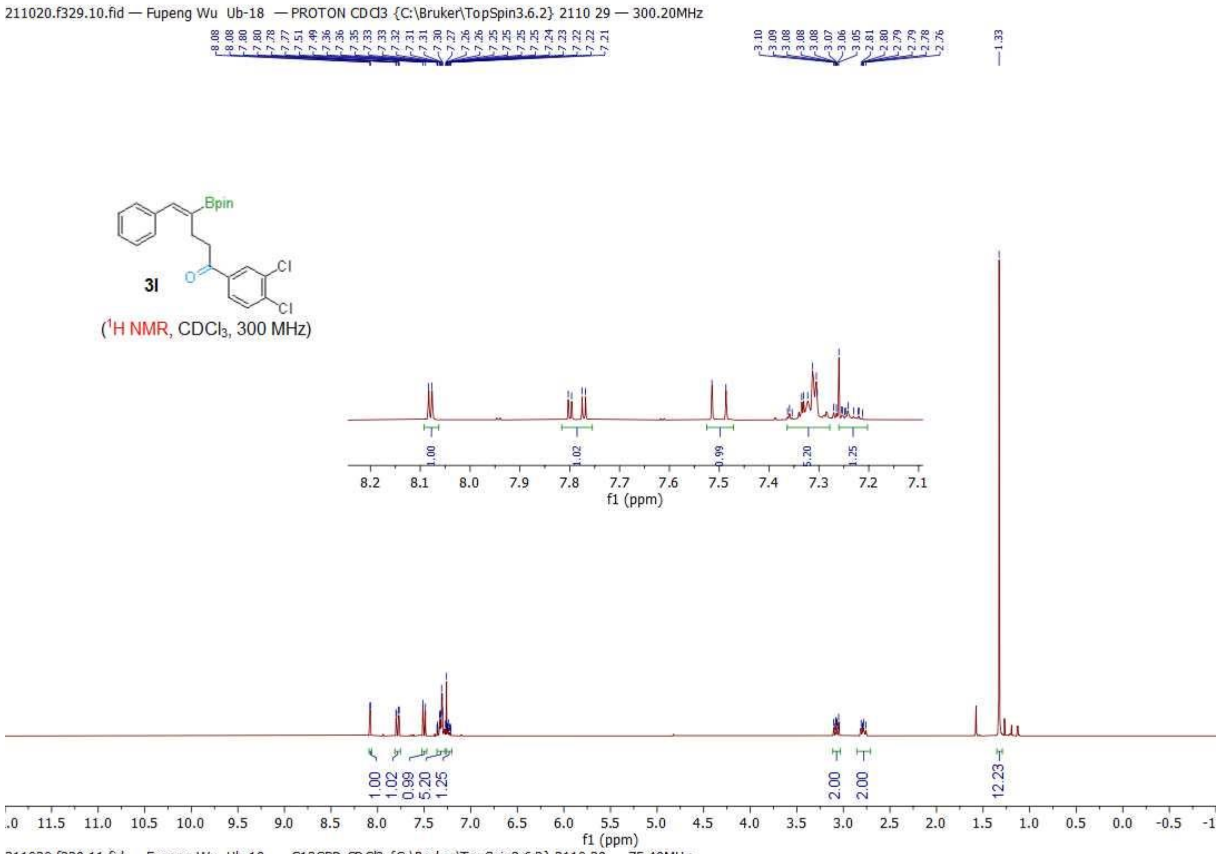
211020.f329.10.fid — Fupeng Wu Ub-18 — PROTON CDCl3 {C:\Bruker\TopSpin3.6.2} 2110 29 — 300.20MHz
Bpin
Cl
Cl
O
3l
(1H NMR, CDCl3, 300 MHz)
1.00
1.02
0.99
5.20
1.25
2.00
2.00
12.23
f1 (ppm)

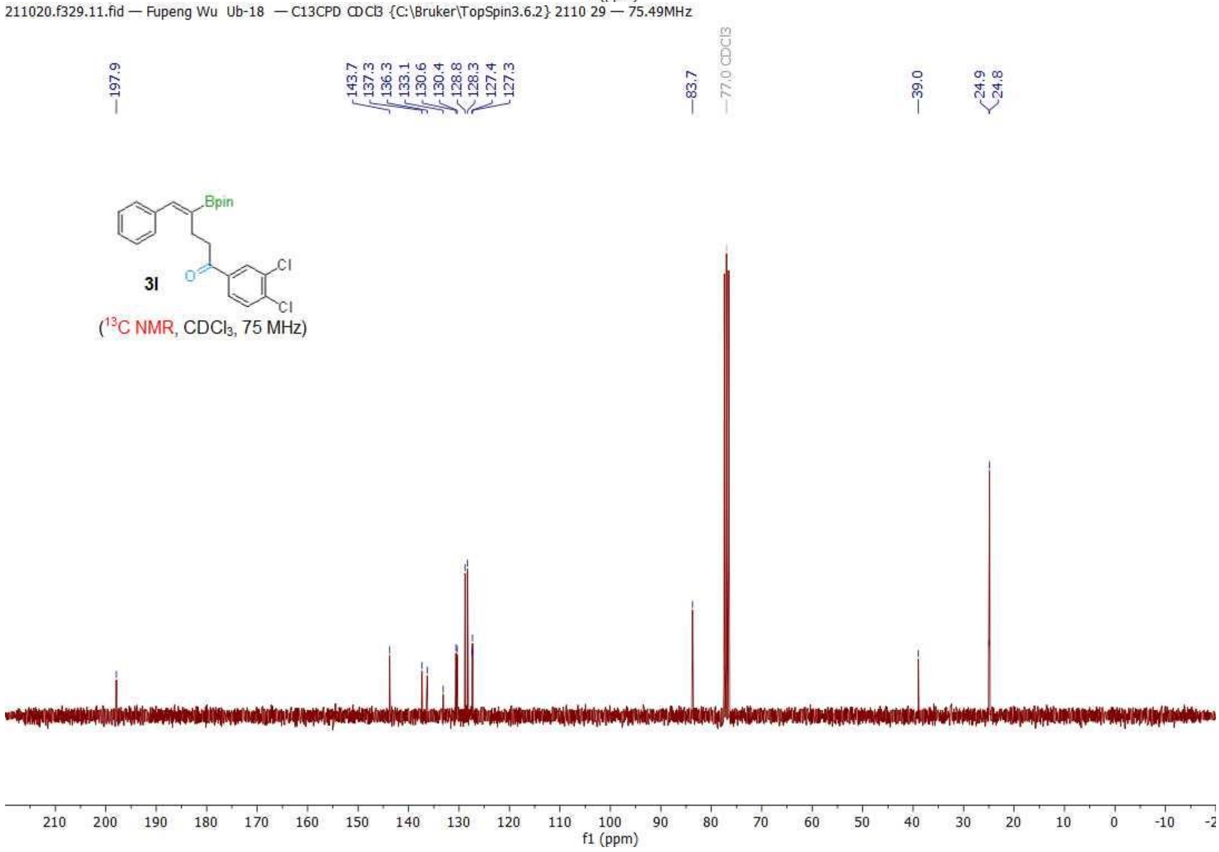
211020.f329.11.fid — Fupeng Wu Ub-18 — C13CPD CDCl3 {C:\Bruker\TopSpin3.6.2} 2110 29 — 75.49MHz
197.9
143.7
137.3
136.3
133.1
130.6
130.4
128.8
128.3
127.4
127.3
83.7
77.0 CDCl3
39.0
24.9
24.8
Bpin
Cl
Cl
O
3l
(13C NMR, CDCl3, 75 MHz)
f1 (ppm)

211020.f329.12.fid — Fupeng Wu Ub-18 — 11B CDCl3 {C:\Bruker\TopSpin3.6.2} 2110 29 — 96.32MHz

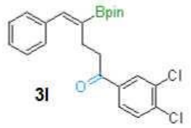

3l

(^{11}B NMR, $CDCl_3$, 96 MHz)

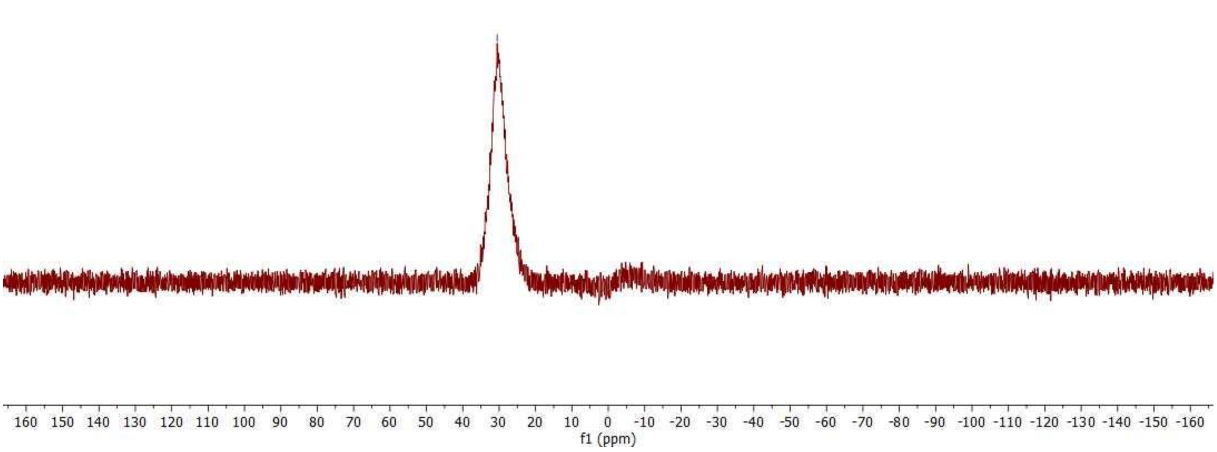

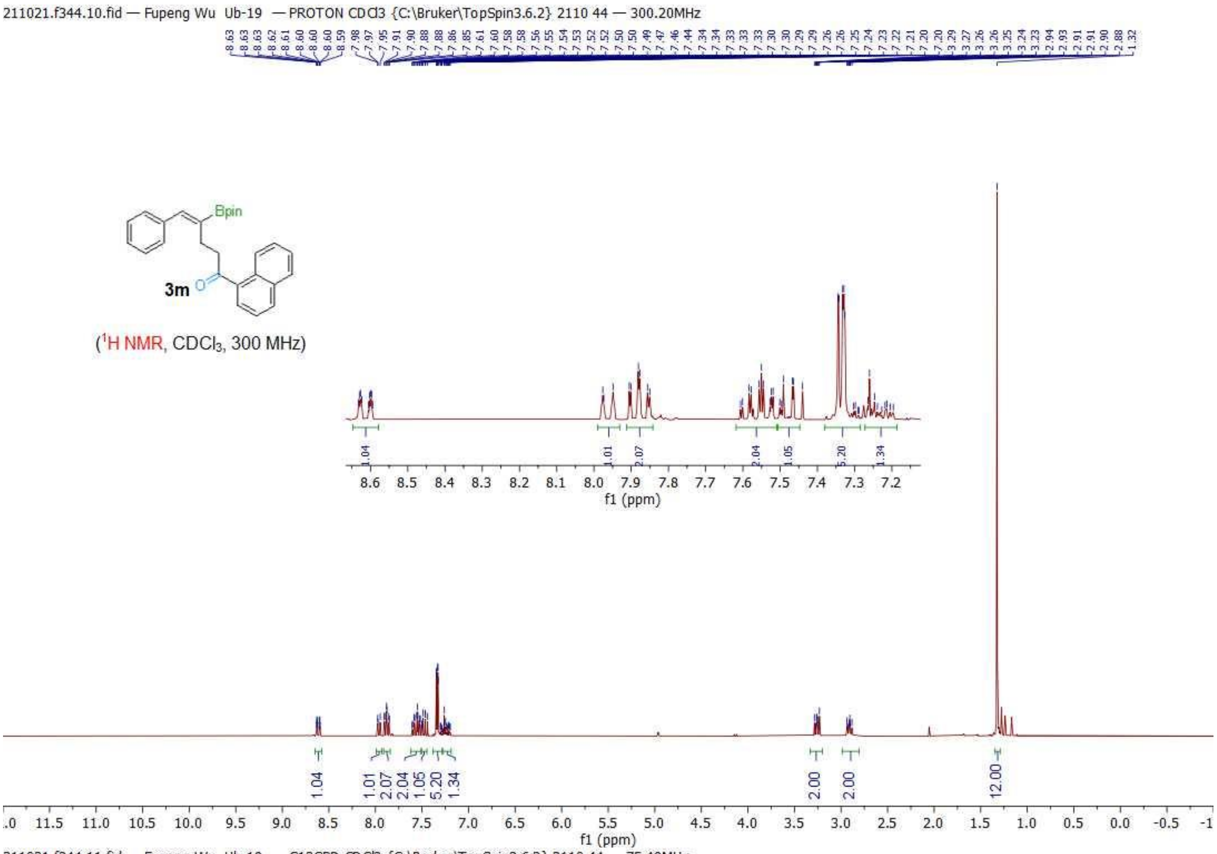
211021.f344.10.fid — Fupeng Wu Ub-19 — PROTON CDCl3 {C:\Bruker\TopSpin3.6.2} 2110 44 — 300.20MHz
8.63 8.63 8.62 8.61 8.60 8.60 8.60 8.59 7.98 7.97 7.95 7.91 7.90 7.88 7.88 7.86 7.85 7.61 7.60 7.58 7.58 7.56 7.55 7.54 7.53 7.52 7.52 7.50 7.50 7.49 7.47 7.46 7.44 7.34 7.34 7.33 7.33 7.33 7.30 7.30 7.29 7.29 7.26 7.26 7.25 7.24 7.23 7.22 7.22 7.21 7.20 7.20 7.20 3.29 3.27 3.26 3.26 3.25 3.24 3.23 2.94 2.93 2.91 2.91 2.90 2.88 1.32
Bpin
O
3m
(1H NMR, CDCl3, 300 MHz)
1.04 1.01 2.07 2.04 1.05 5.20 1.34
1.04 1.01 2.07 2.04 1.05 5.20 1.34 2.00 2.00 12.00
f1 (ppm)

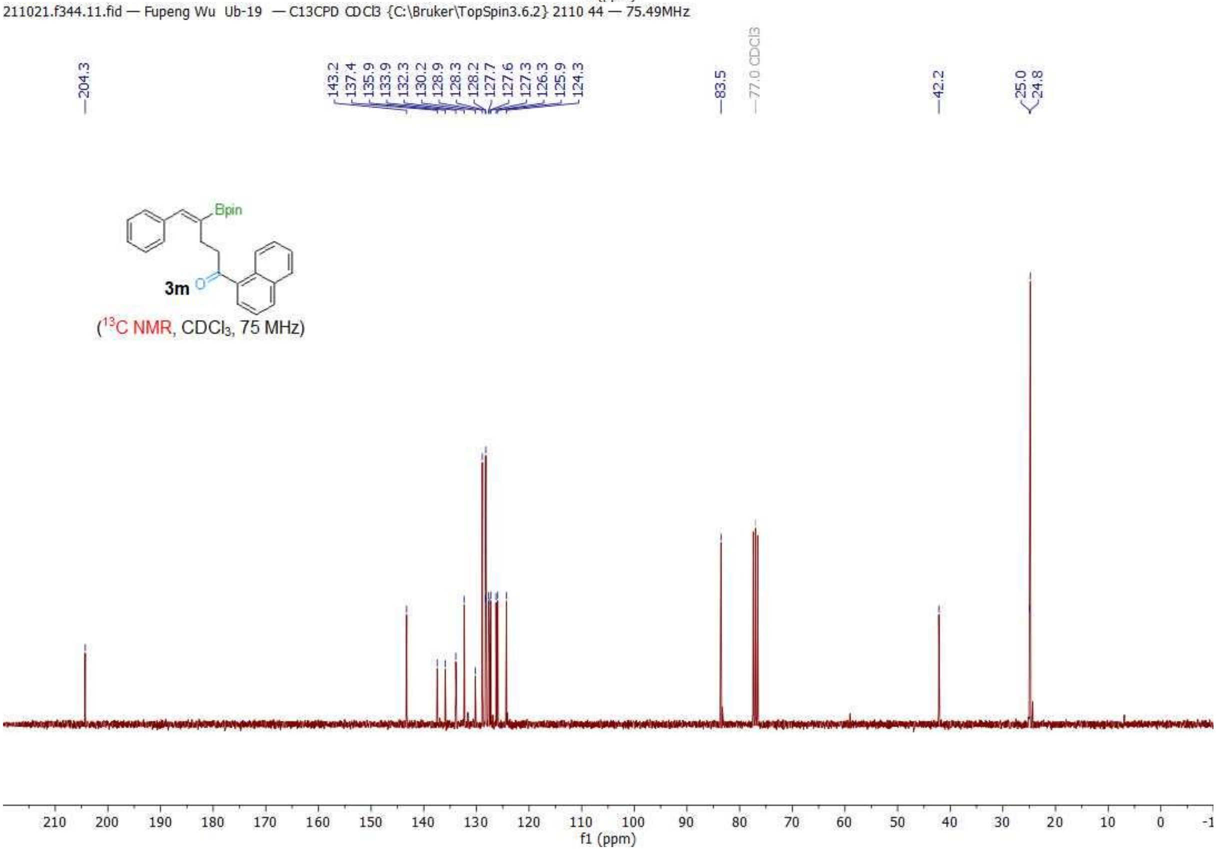
211021.f344.11.fid — Fupeng Wu Ub-19 — C13CPD CDCl3 {C:\Bruker\TopSpin3.6.2} 2110 44 — 75.49MHz
204.3
143.2 137.4 135.9 133.9 132.3 130.2 128.9 128.3 128.2 127.7 127.6 127.3 126.3 125.9 124.3
83.5
77.0 CDCl3
42.2
25.0 24.8
Bpin
O
3m
(13C NMR, CDCl3, 75 MHz)
f1 (ppm)

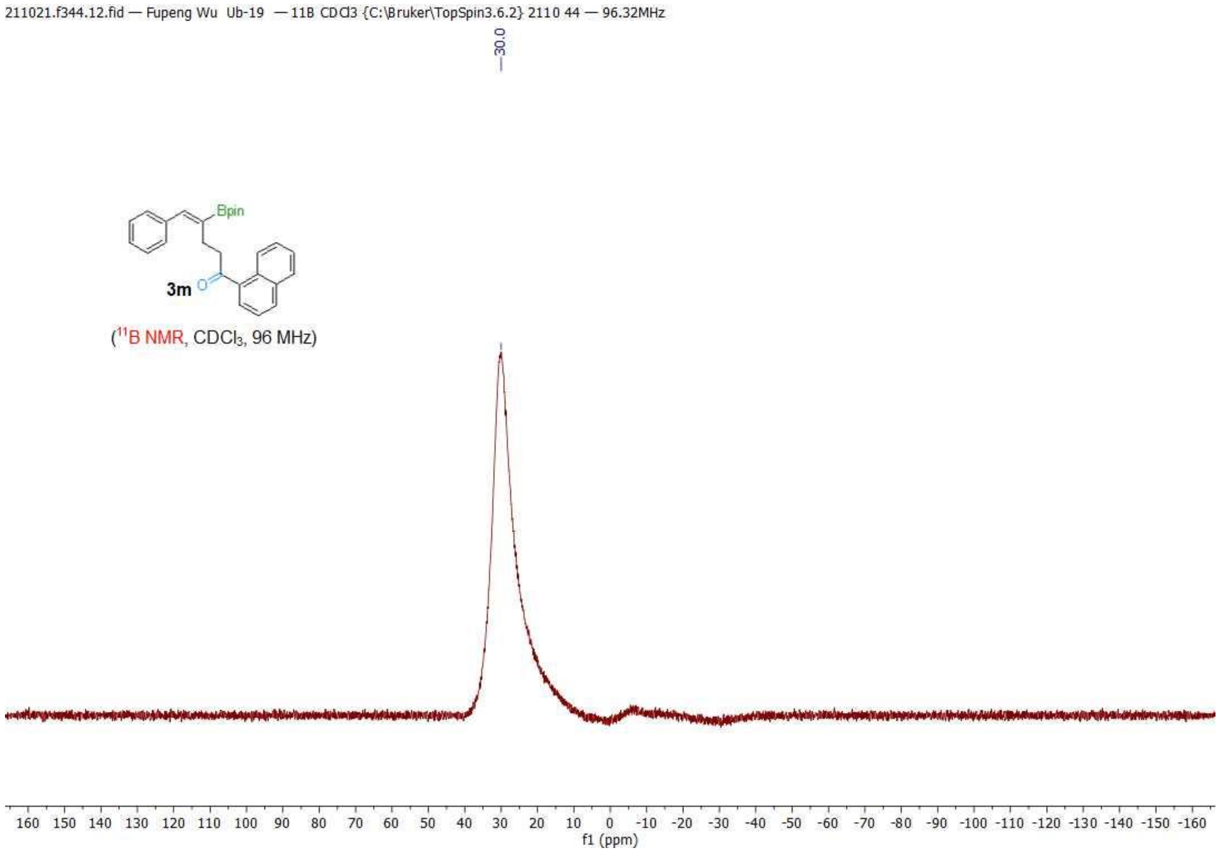
211021.f344.12.fid — Fupeng Wu Ub-19 — 11B CDCl3 {C:\Bruker\TopSpin3.6.2} 2110 44 — 96.32MHz
30.0
Bpin
3m
(11B NMR, CDCl3, 96 MHz)
160 150 140 130 120 110 100 90 80 70 60 50 40 30 20 10 0 -10 -20 -30 -40 -50 -60 -70 -80 -90 -100 -110 -120 -130 -140 -150 -160
f1 (ppm)

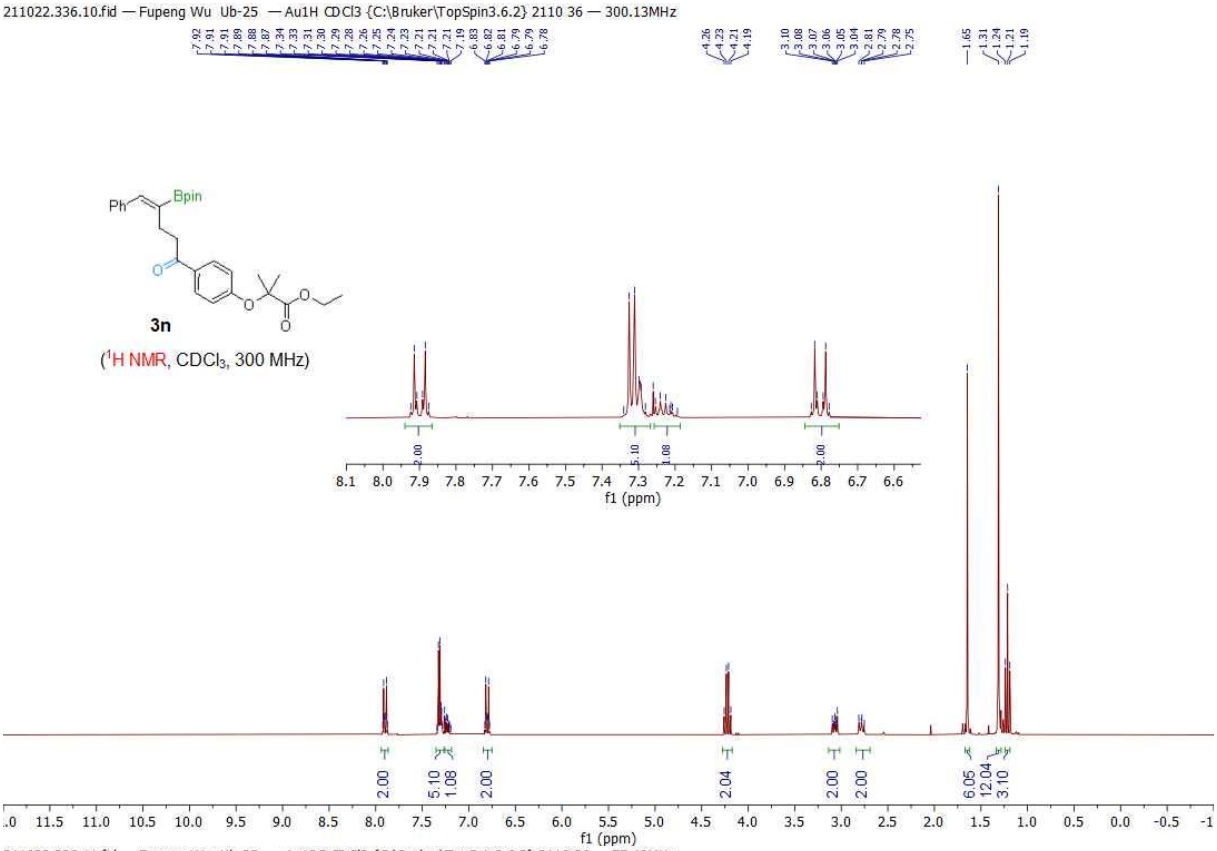
211022.336.10.fid — Fupeng Wu Ub-25 — Au1H CDCl3 {C:\Bruker\TopSpin3.6.2} 2110 36 — 300.13MHz
Ph
Bpin
3n
(1H NMR, CDCl3, 300 MHz)
f1 (ppm)

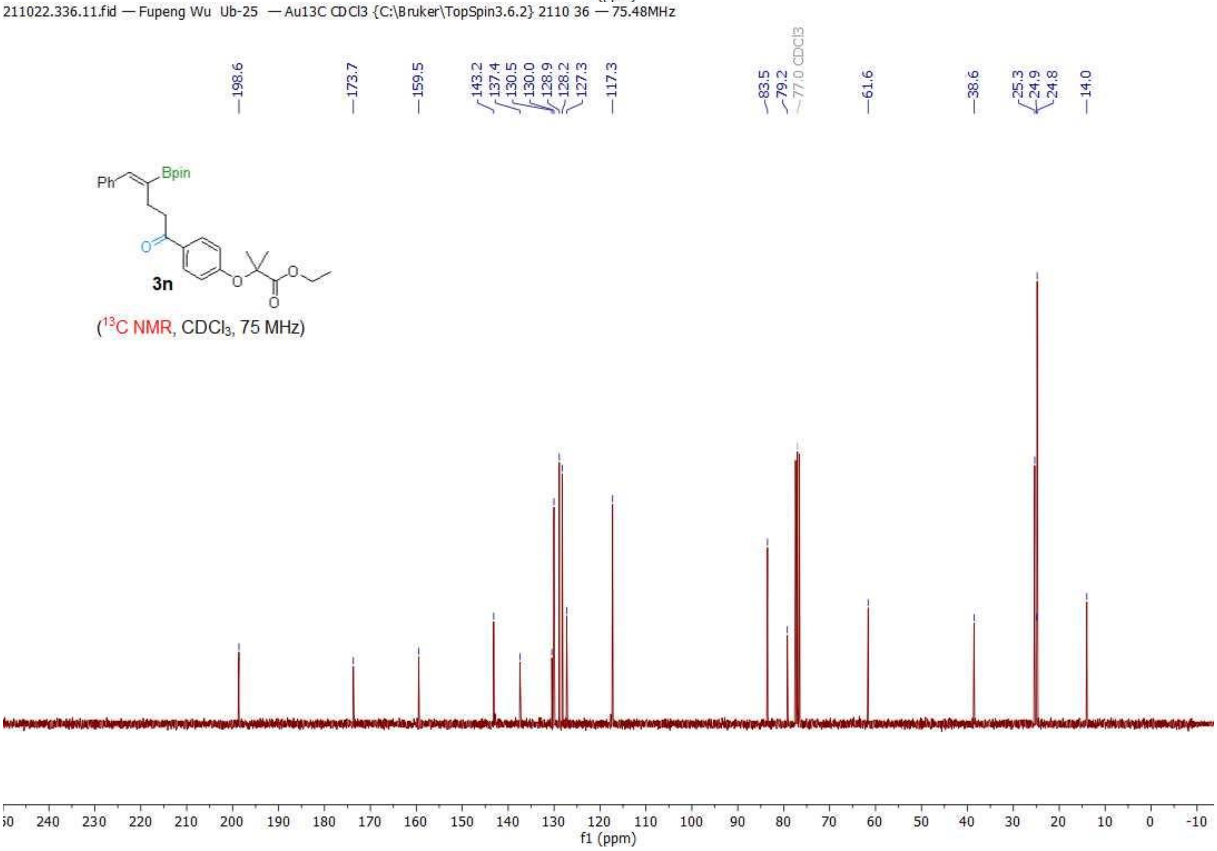
211022.336.11.fid — Fupeng Wu Ub-25 — Au13C CDCl3 {C:\Bruker\TopSpin3.6.2} 2110 36 — 75.48MHz
198.6
173.7
159.5
143.2
137.4
130.5
130.0
128.9
128.2
127.3
117.3
83.5
79.2
77.0 CDCl3
61.6
38.6
25.3
24.9
24.8
14.0
Ph
Bpin
3n
(13C NMR, CDCl3, 75 MHz)
f1 (ppm)

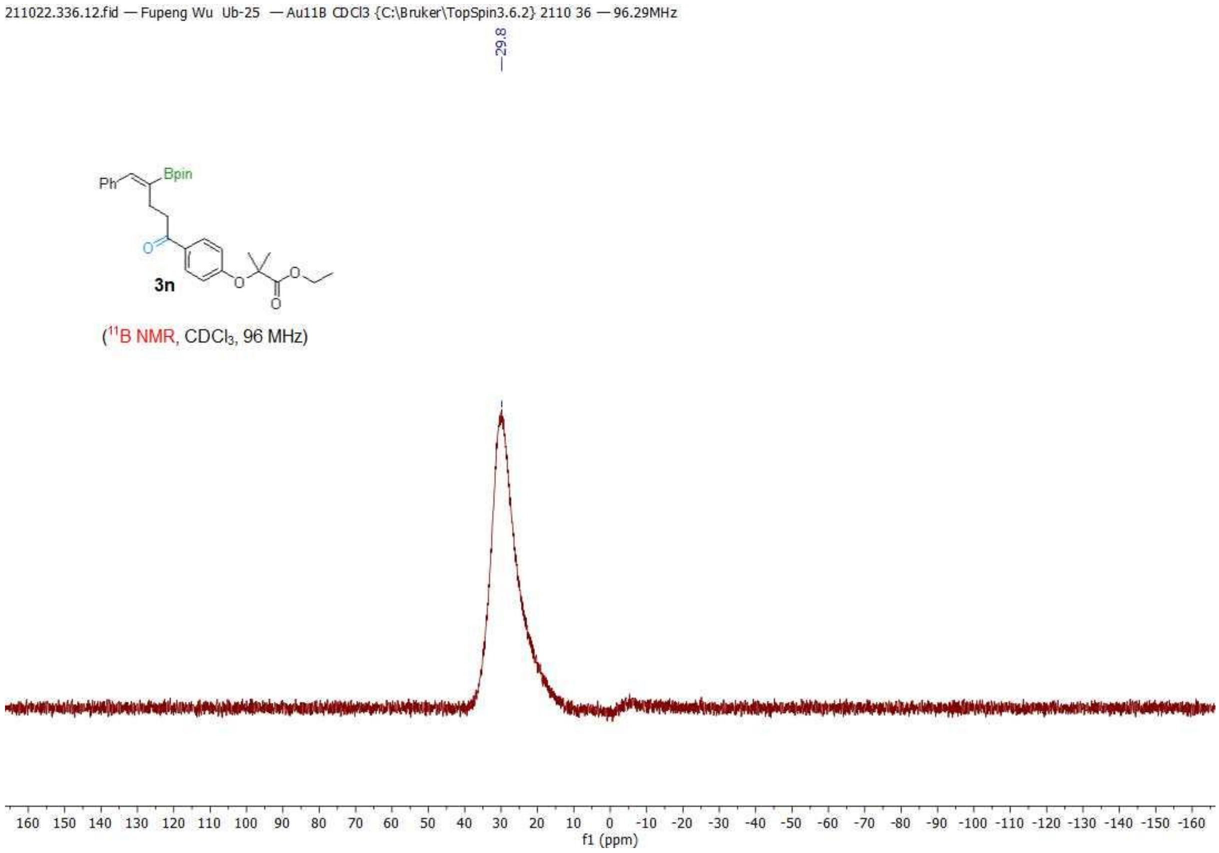

211022.336.12.fid — Fupeng Wu Ub-25 — Au11B CDCl3 {C:\Bruker\TopSpin3.6.2} 2110 36 — 96.29MHz
29.8
Ph
Bpin
O
O
O
O
3n
(^{11}B NMR, $CDCl_3$, 96 MHz)
160 150 140 130 120 110 100 90 80 70 60 50 40 30 20 10 0 -10 -20 -30 -40 -50 -60 -70 -80 -90 -100 -110 -120 -130 -140 -150 -160
f1 (ppm)

211105.443.10.fid — Fupeng Wu Ub-44 — Au1H CDCl3 {C:\Bruker\TopSpin3.5pl6} 2111 43 — 400.13MHz

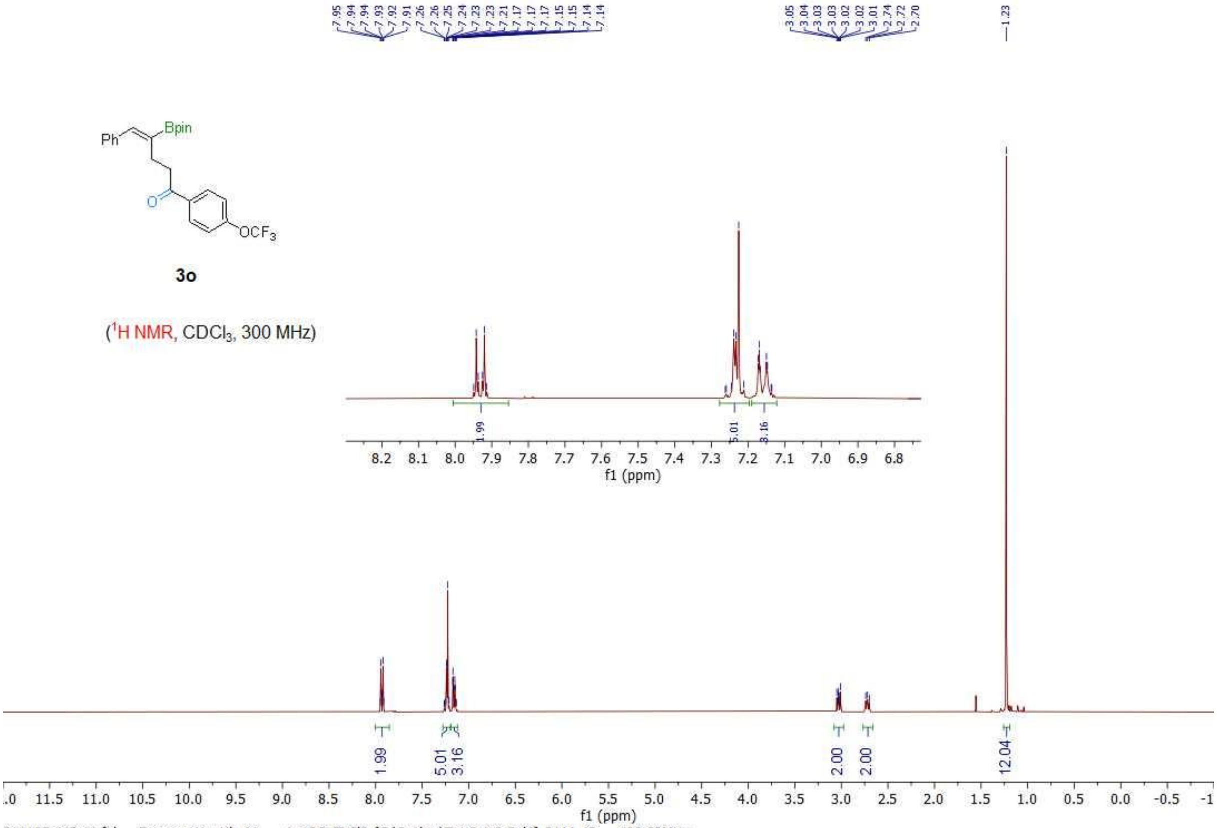

211105.443.11.fid — Fupeng Wu Ub-44 — Au13C CDCl3 {C:\Bruker\TopSpin3.5pl6} 2111 43 — 100.63MHz

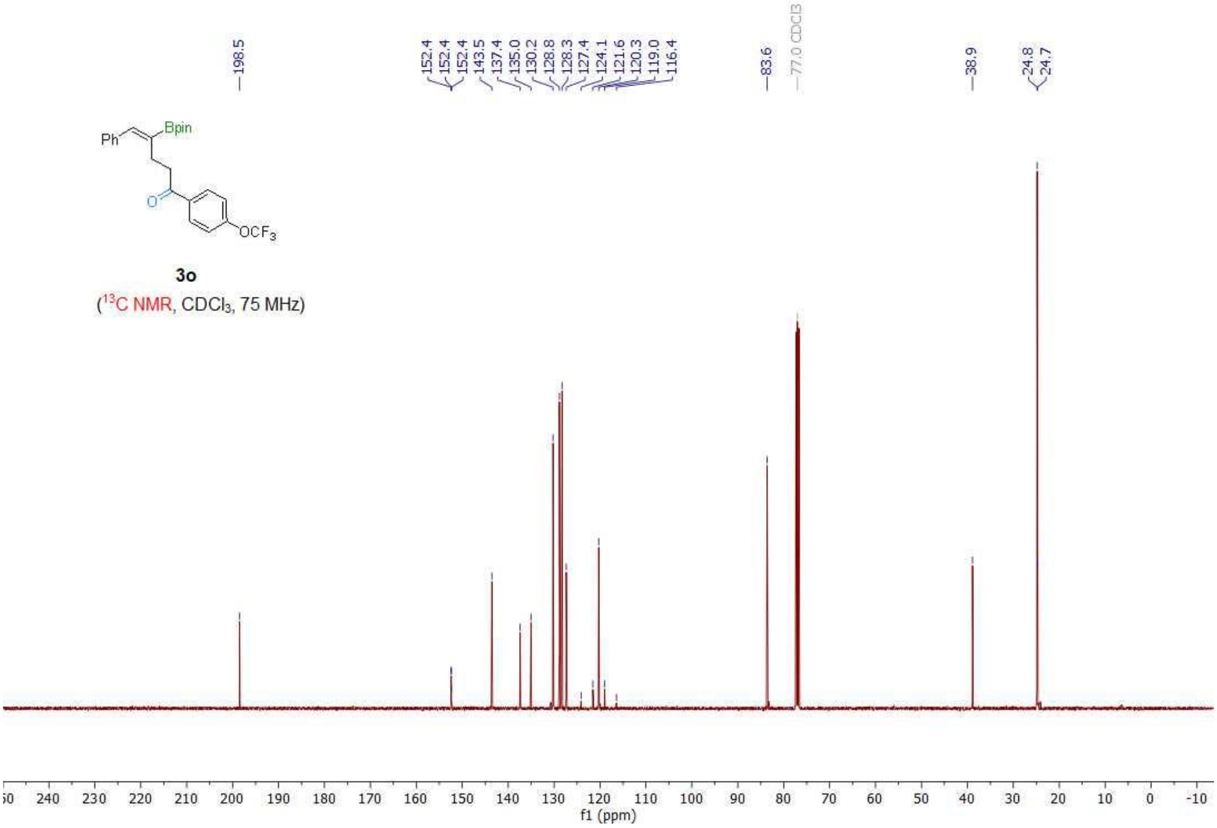

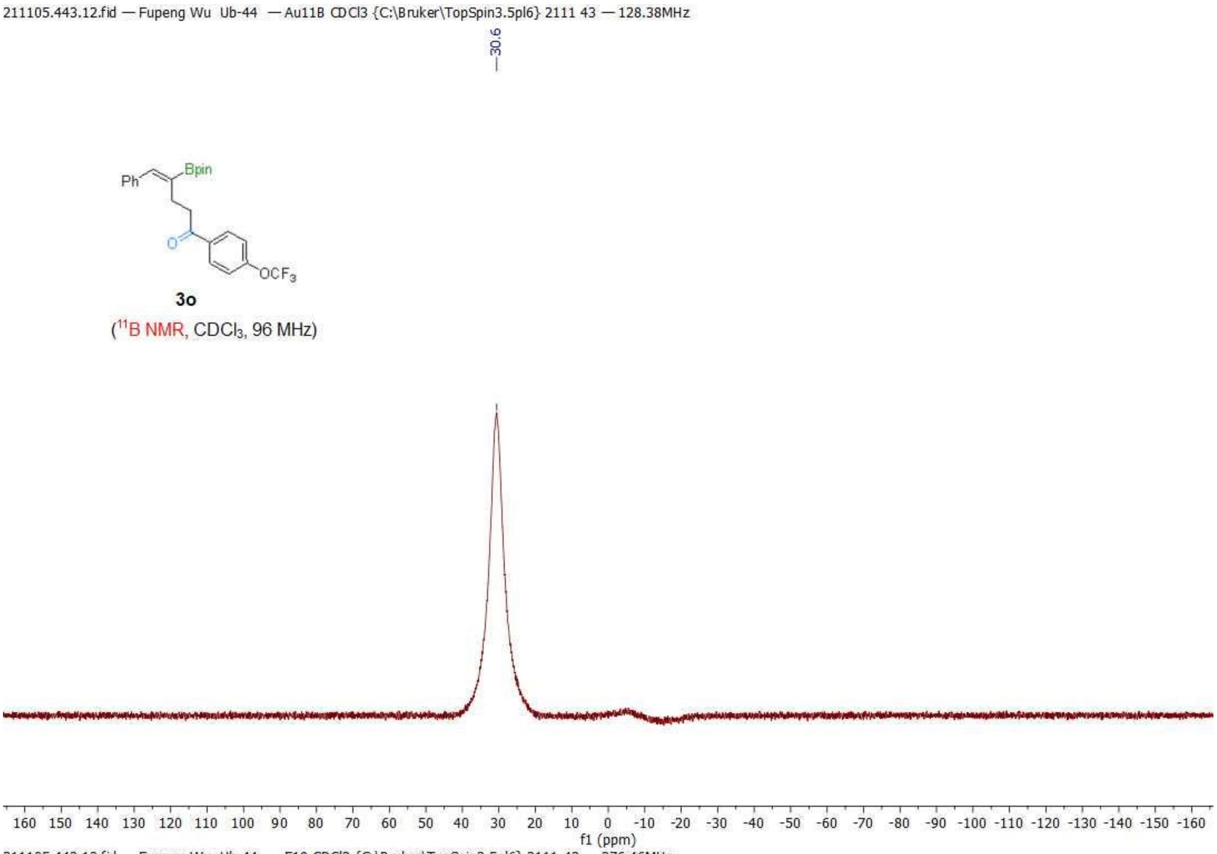
211105.443.12.fid — Fupeng Wu Ub-44 — Au11B CDCl3 {C:\Bruker\TopSpin3.5pl6} 2111 43 — 128.38MHz
30.6
Ph
Bpin
O
OCF3
3o
(11B NMR, CDCl3, 96 MHz)
f1 (ppm)

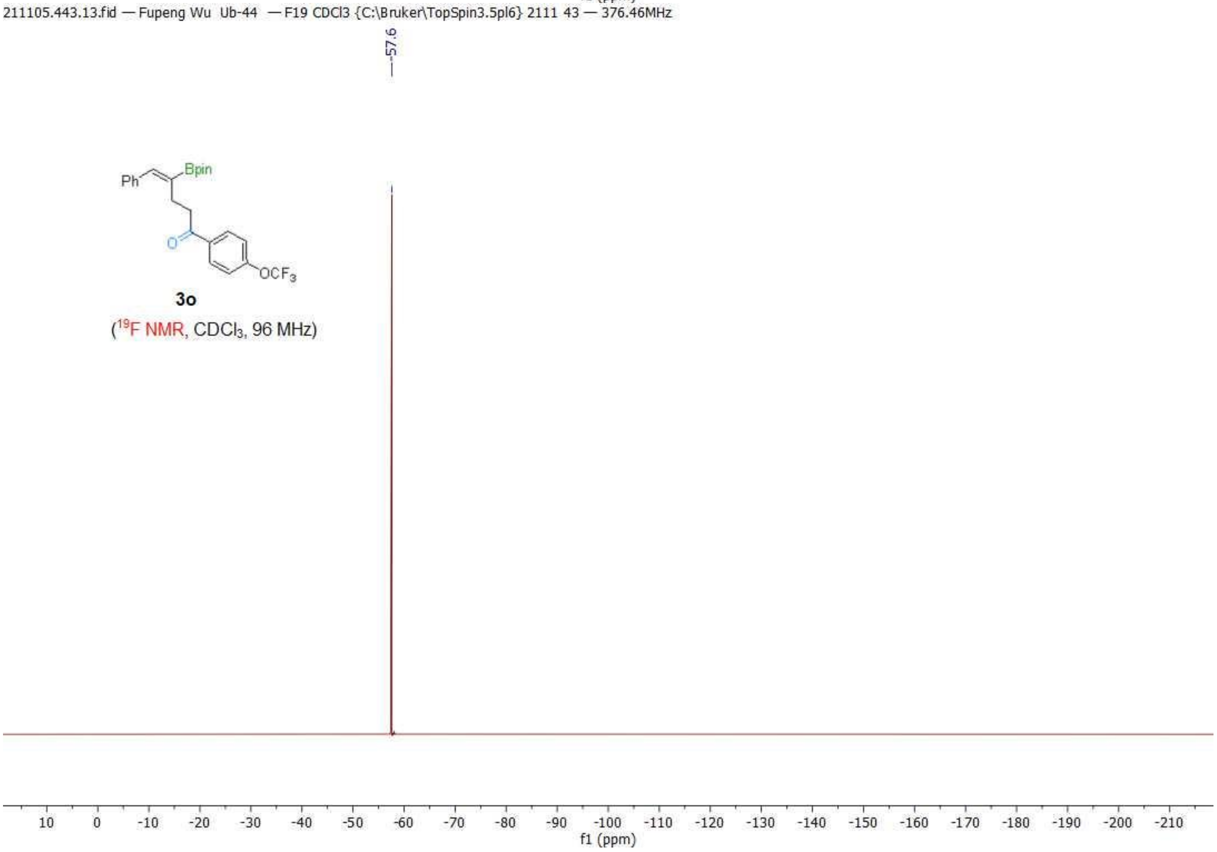
211105.443.13.fid — Fupeng Wu Ub-44 — F19 CDCl3 {C:\Bruker\TopSpin3.5pl6} 2111 43 — 376.46MHz
-57.6
Ph
Bpin
O
OCF3
3o
(19F NMR, CDCl3, 96 MHz)
f1 (ppm)

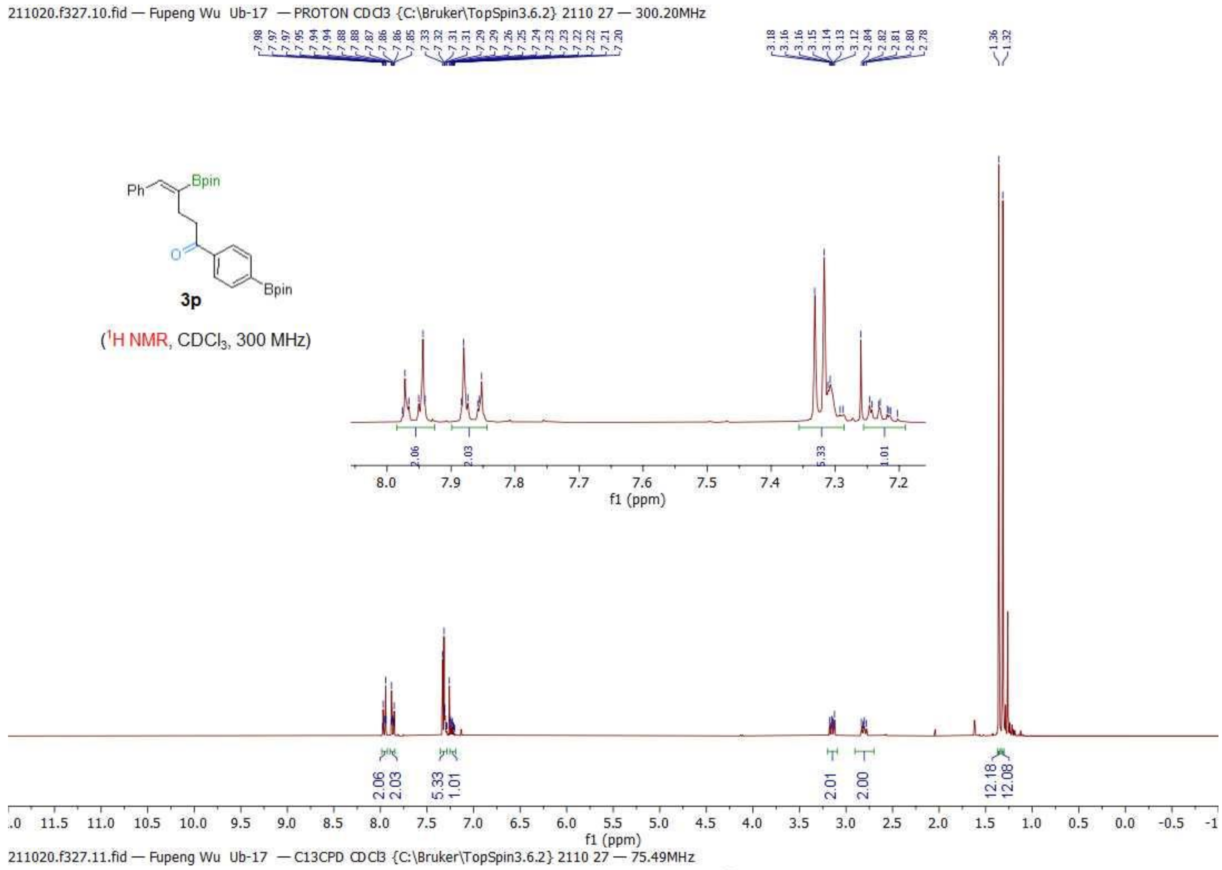
211020.f327.10.fid — Fupeng Wu Ub-17 — PROTON CDCl3 {C:\Bruker\TopSpin3.6.2} 2110 27 — 300.20MHz
Ph
Bpin
O
Bpin
3p
(1H NMR, CDCl3, 300 MHz)
2.06
2.03
5.33
1.01
2.01
2.00
12.18
12.08
f1 (ppm)

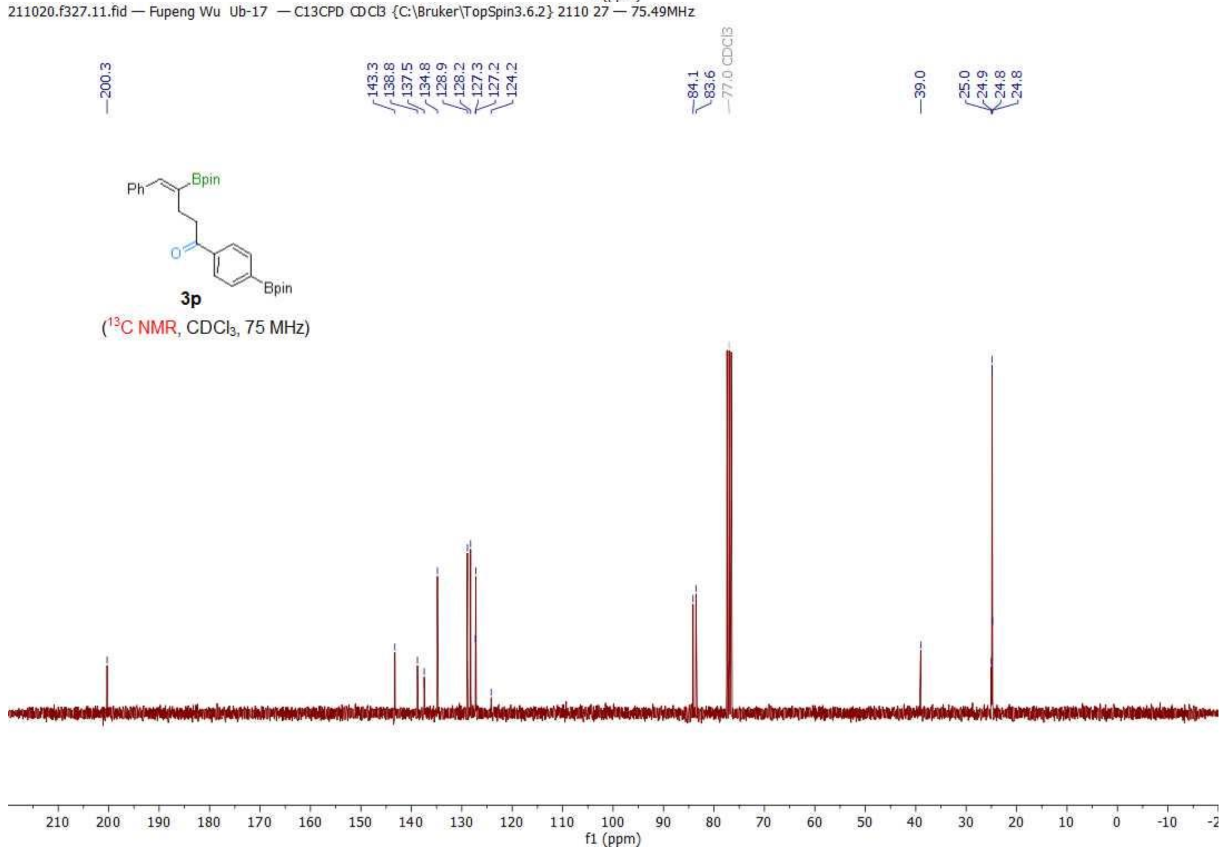
211020.f327.11.fid — Fupeng Wu Ub-17 — C13CPD CDCl3 {C:\Bruker\TopSpin3.6.2} 2110 27 — 75.49MHz
200.3
143.3
138.8
137.5
134.8
128.9
128.2
127.3
127.2
124.2
84.1
83.6
77.0 CDCl3
39.0
25.0
24.9
24.8
24.8
Ph
Bpin
O
Bpin
3p
(13C NMR, CDCl3, 75 MHz)
f1 (ppm)

211019.333.12.fid — Fupeng Wu Ub-17 — Au11B CDCl3 {C:\Bruker\TopSpin3.6.2} 2110 33 — 96.29MHz

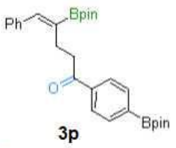

3p

(^{11}B NMR, $CDCl_3$, 96 MHz)

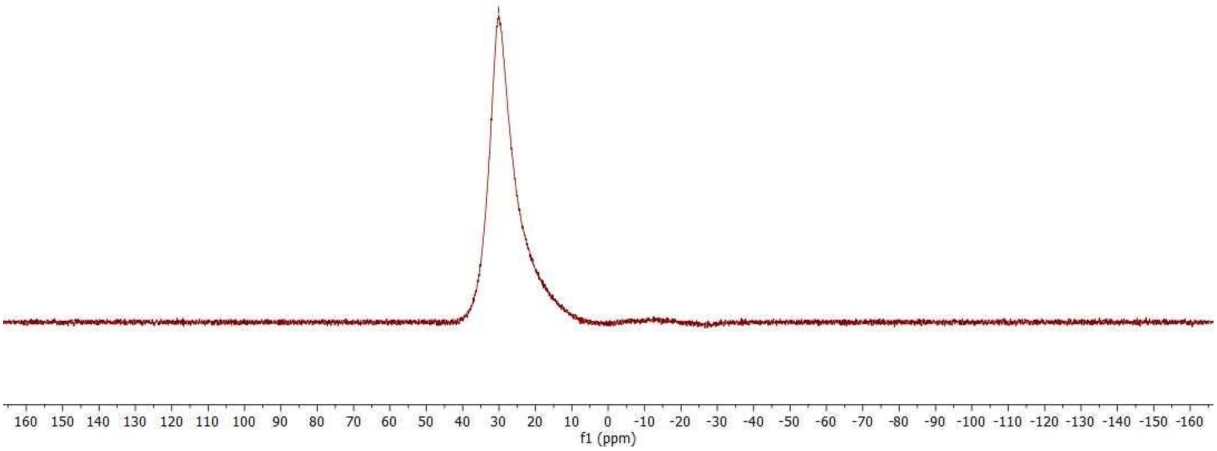

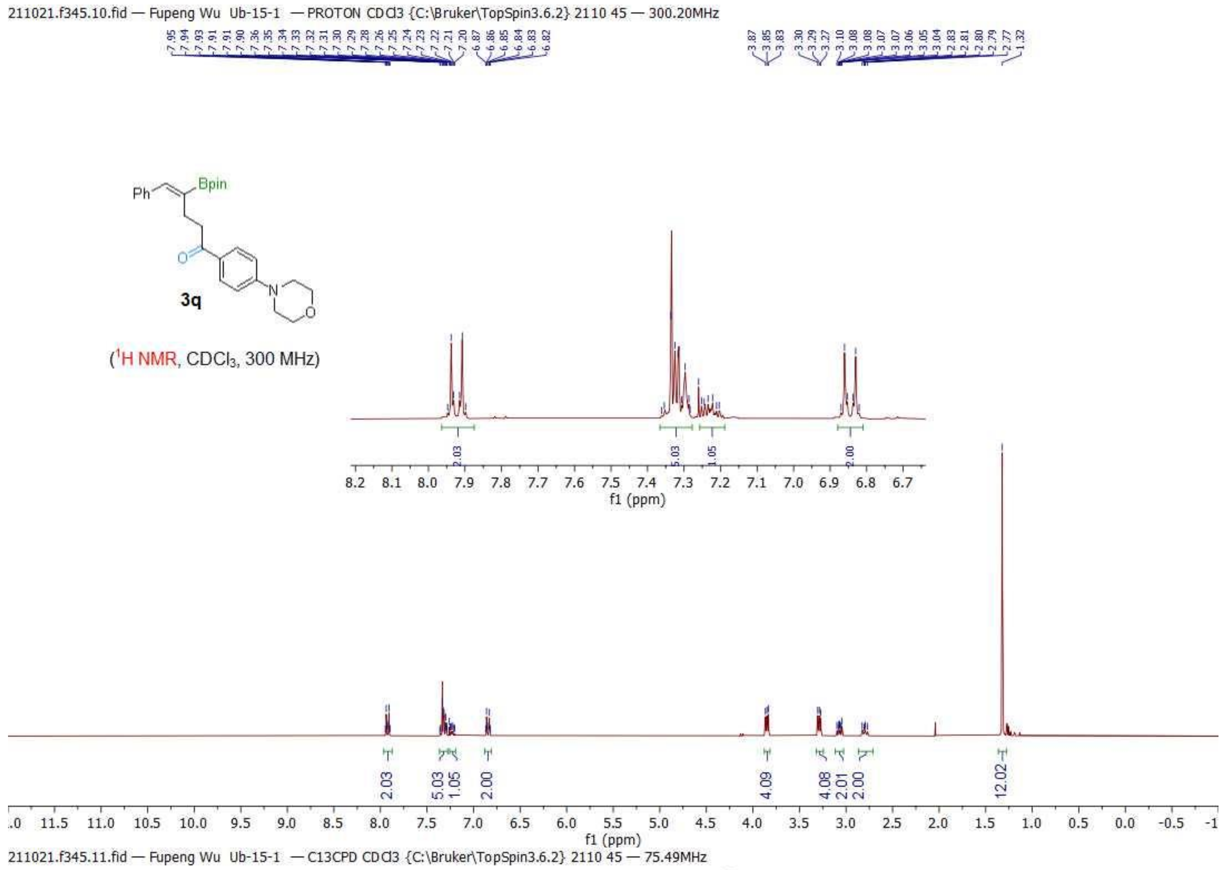
211021.f345.10.fid — Fupeng Wu Ub-15-1 — PROTON CDCl3 {C:\Bruker\TopSpin3.6.2} 2110 45 — 300.20MHz
7.95 7.94 7.93 7.91 7.91 7.90 7.36 7.35 7.34 7.33 7.32 7.31 7.30 7.29 7.28 7.26 7.25 7.24 7.23 7.22 7.21 7.20 6.87 6.86 6.85 6.84 6.83 6.82
3.87 3.85 3.83 3.30 3.29 3.27 3.10 3.08 3.08 3.07 3.06 3.05 3.04 2.83 2.81 2.80 2.79 2.77 1.32
Ph
Bpin
O
N
O
3q
(1H NMR, CDCl3, 300 MHz)
2.03
5.03
1.05
2.00
4.09
4.08
2.01
2.00
12.02
f1 (ppm)

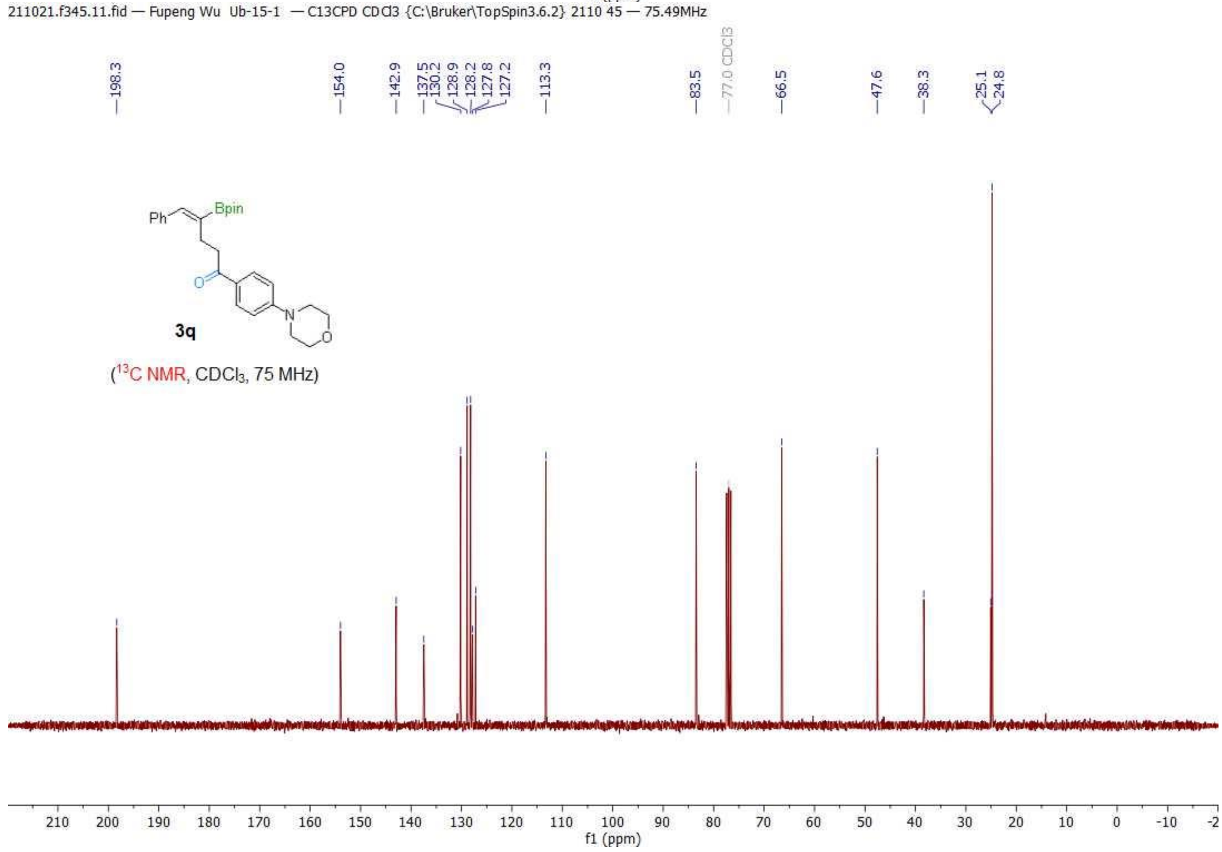
211021.f345.11.fid — Fupeng Wu Ub-15-1 — C13CPD CDCl3 {C:\Bruker\TopSpin3.6.2} 2110 45 — 75.49MHz
198.3
154.0
142.9
137.5
130.2
128.9
128.2
127.8
127.2
113.3
83.5
77.0 CDCl3
66.5
47.6
38.3
25.1
24.8
Ph
Bpin
O
N
O
3q
(13C NMR, CDCl3, 75 MHz)
f1 (ppm)

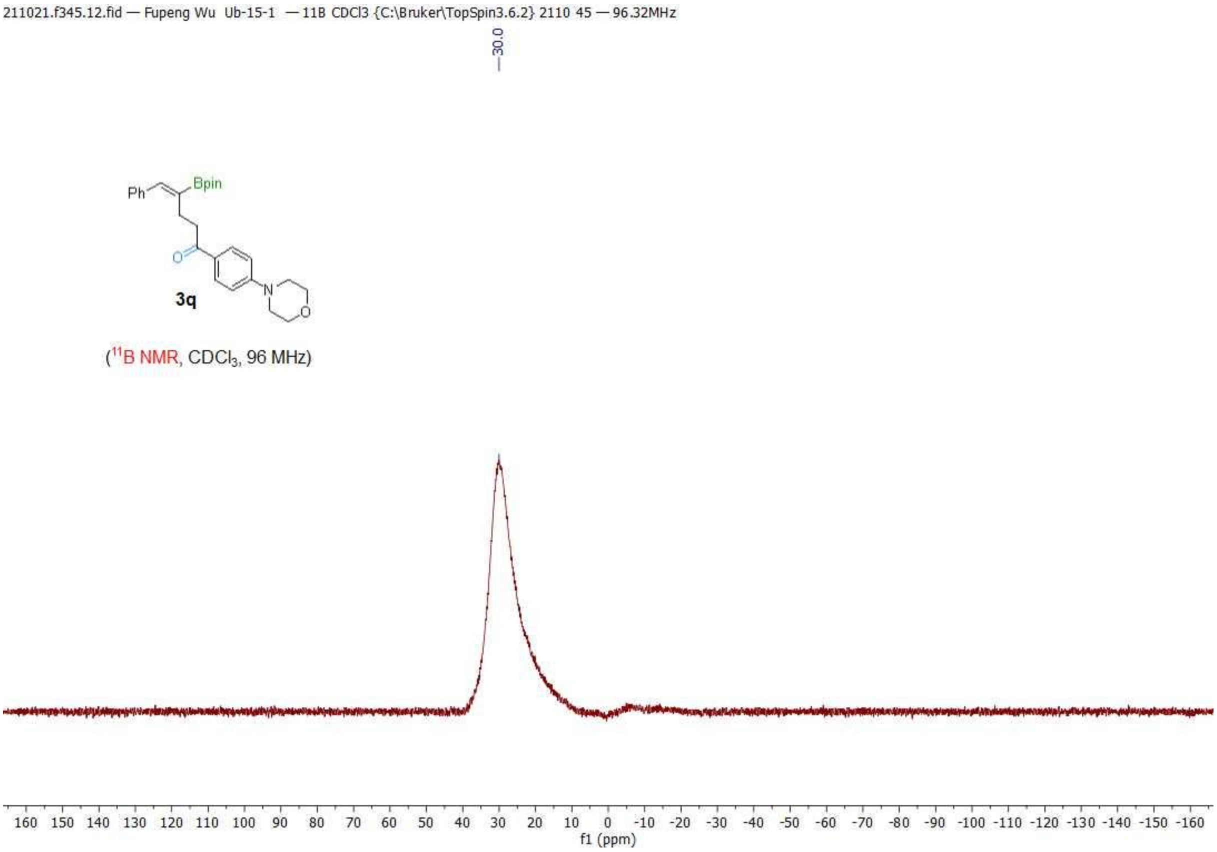

(^{11}B NMR, $CDCl_3$, 96 MHz)

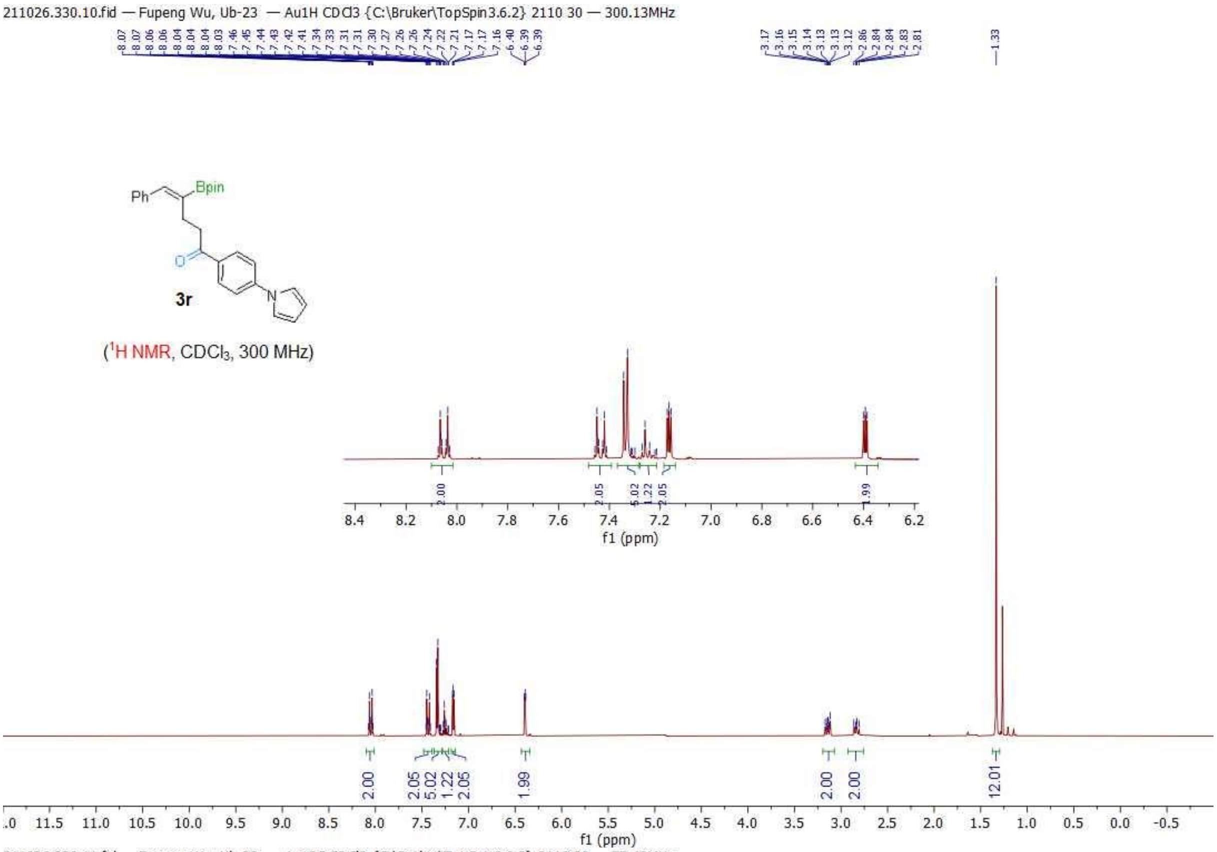
211026.330.10.fid — Fupeng Wu, Ub-23 — Au1H CDCl3 {C:\Bruker\TopSpin3.6.2} 2110 30 — 300.13MHz
8.07 8.07 8.06 8.06 8.04 8.04 8.03 7.46 7.45 7.44 7.43 7.42 7.41 7.34 7.33 7.31 7.31 7.30 7.27 7.26 7.26 7.24 7.22 7.21 7.17 7.17 7.16 6.40 6.39 6.39
3.17 3.16 3.15 3.14 3.13 3.13 3.12 2.86 2.84 2.84 2.83 2.81
1.33
Ph
Bpin
O
N
3r
(¹H NMR, CDCl₃, 300 MHz)
2.00 2.05 5.02 1.22 2.05 1.99
f1 (ppm)
2.00 2.05 5.02 1.22 2.05 1.99 2.00 2.00 12.01
f1 (ppm)

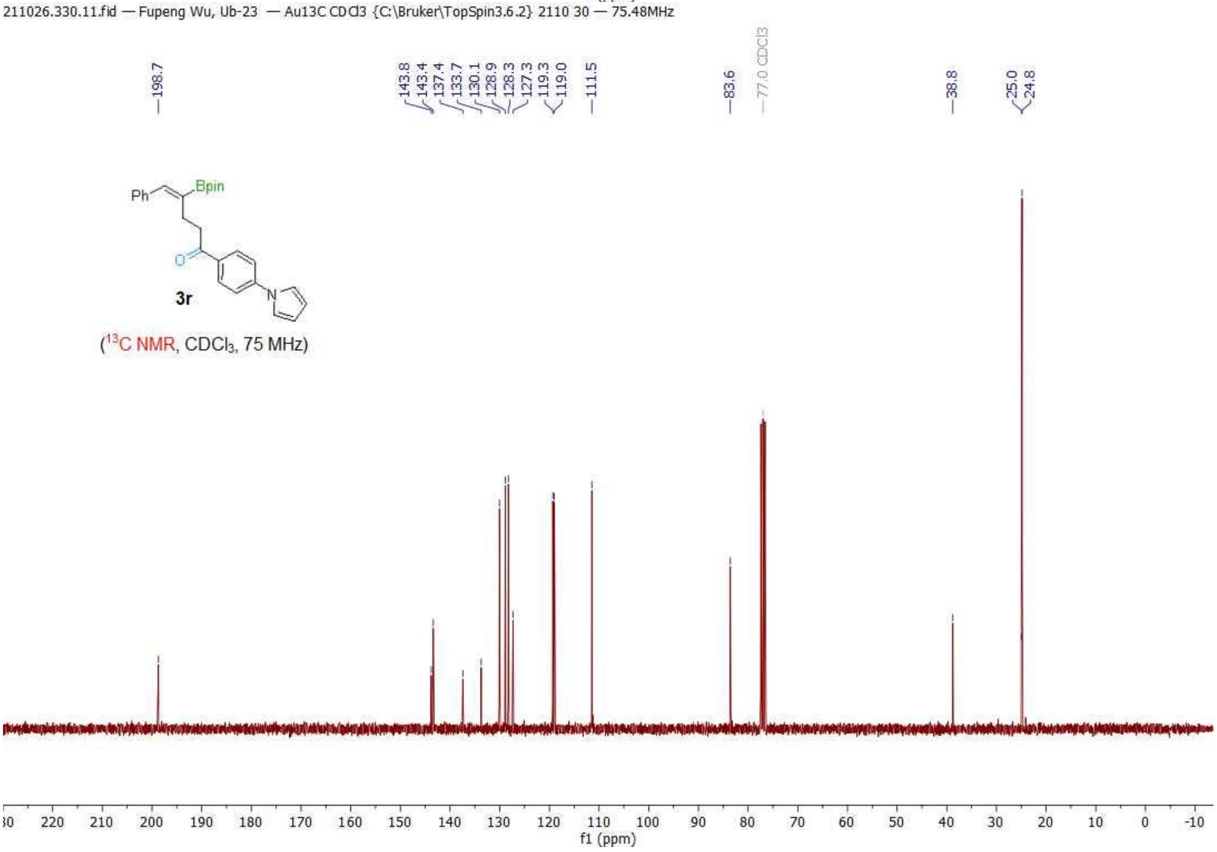
211026.330.11.fid — Fupeng Wu, Ub-23 — Au13C CDCl3 {C:\Bruker\TopSpin3.6.2} 2110 30 — 75.48MHz
198.7
143.8 143.4 137.4 133.7 130.1 128.9 128.3 127.3 119.3 119.0
111.5
83.6
77.0 CDCl3
38.8
25.0 24.8
Ph
Bpin
O
N
3r
(¹³C NMR, CDCl₃, 75 MHz)
f1 (ppm)

211021.f342.12.fid — Fupeng Wu Ub-23 — 11B CDCl3 {C:\Bruker\TopSpin3.6.2} 2110 42 — 96.32MHz

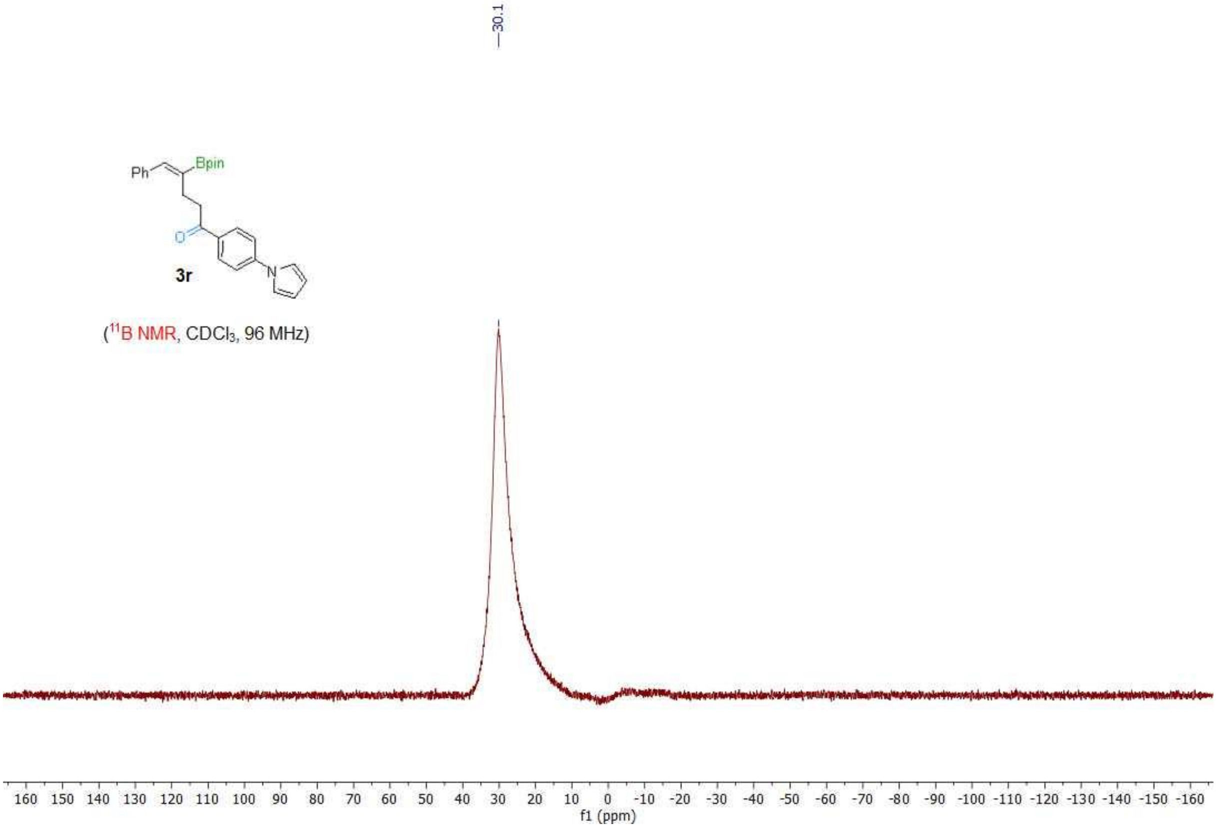

(^{11}B NMR, $CDCl_3$, 96 MHz)

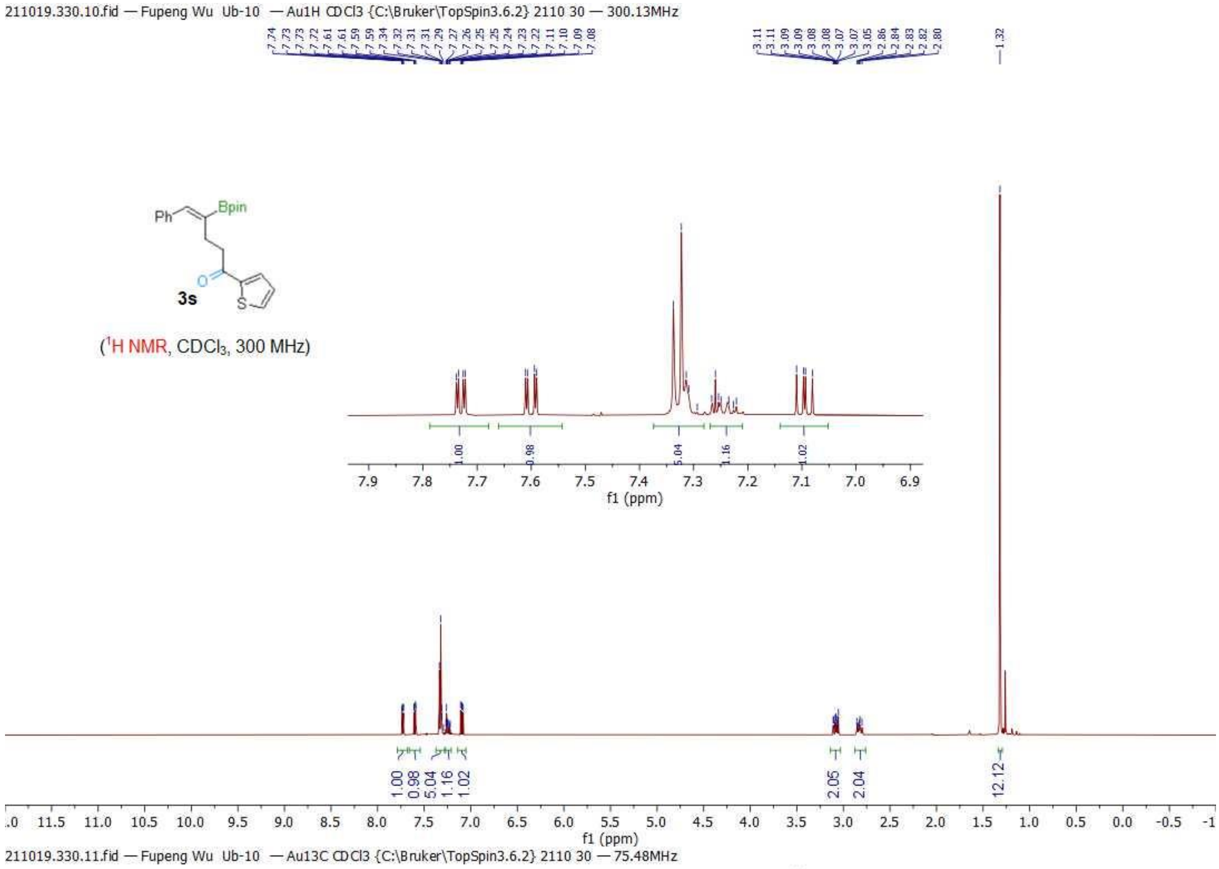
211019.330.10.fid — Fupeng Wu Ub-10 — Au1H CDCl3 {C:\Bruker\TopSpin3.6.2} 2110 30 — 300.13MHz
Ph
Bpin
O
S
3s
(1H NMR, CDCl3, 300 MHz)
f1 (ppm)

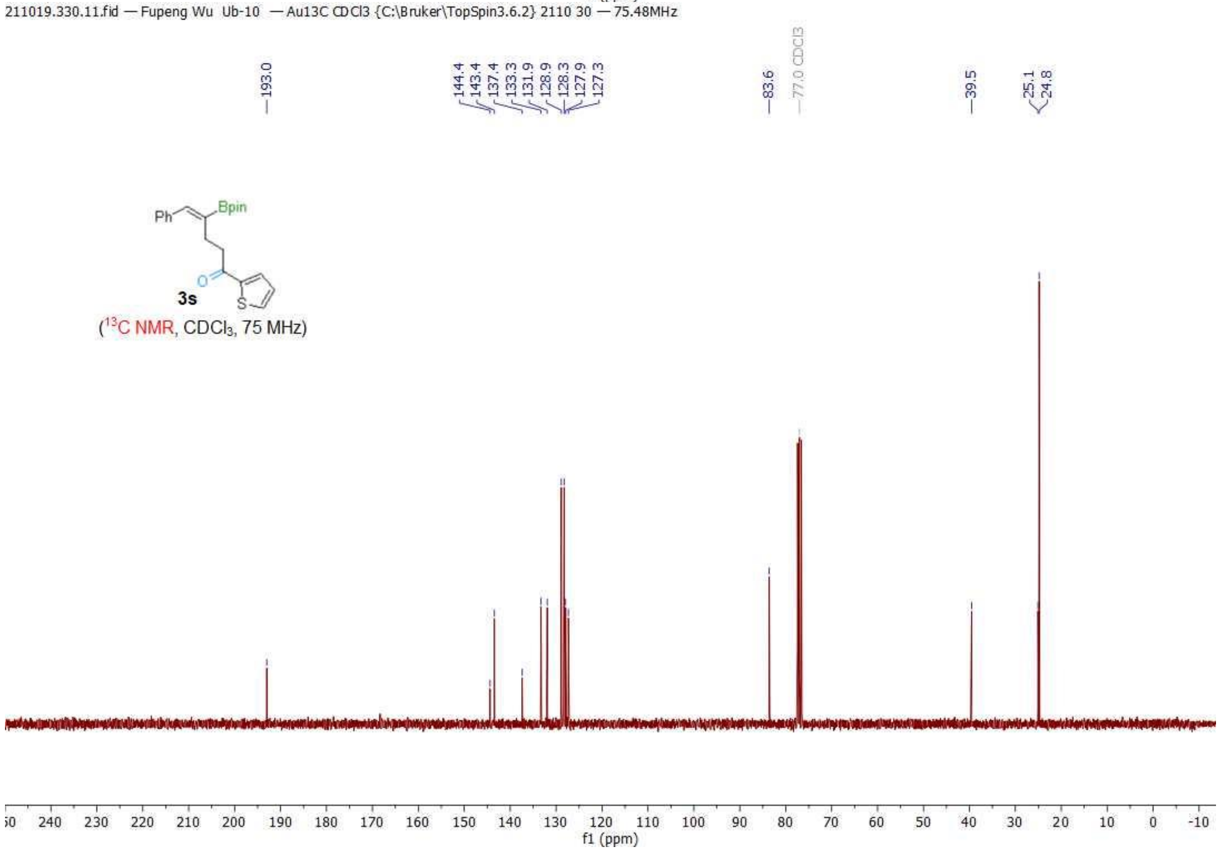
211019.330.11.fid — Fupeng Wu Ub-10 — Au13C CDCl3 {C:\Bruker\TopSpin3.6.2} 2110 30 — 75.48MHz
Ph
Bpin
O
S
3s
(13C NMR, CDCl3, 75 MHz)
f1 (ppm)

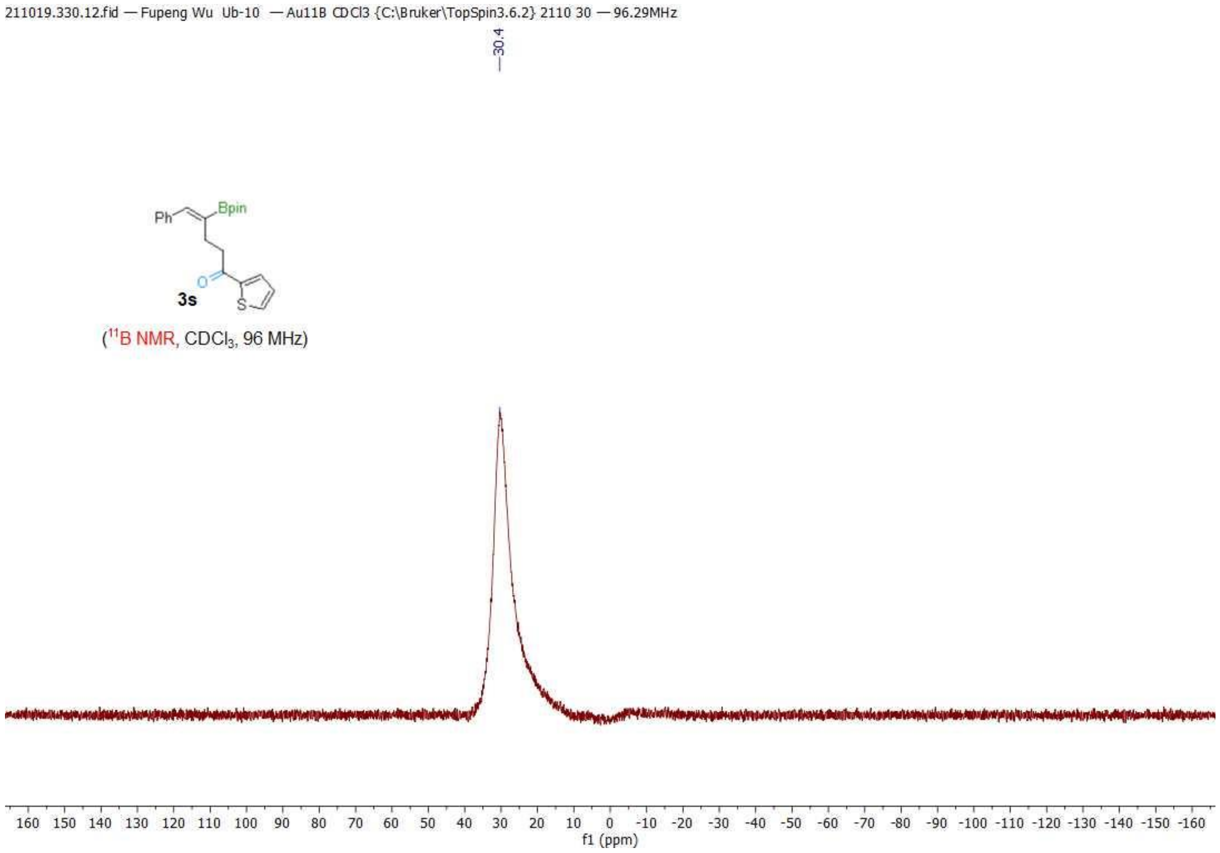
211019.330.12.fid — Fupeng Wu Ub-10 — Au11B CDCl3 {C:\Bruker\TopSpin3.6.2} 2110 30 — 96.29MHz
—30.4
Ph
Bpin
O
S
3s
(^{11}B NMR, CDCl$_3$, 96 MHz)
160 150 140 130 120 110 100 90 80 70 60 50 40 30 20 10 0 -10 -20 -30 -40 -50 -60 -70 -80 -90 -100 -110 -120 -130 -140 -150 -160
f1 (ppm)

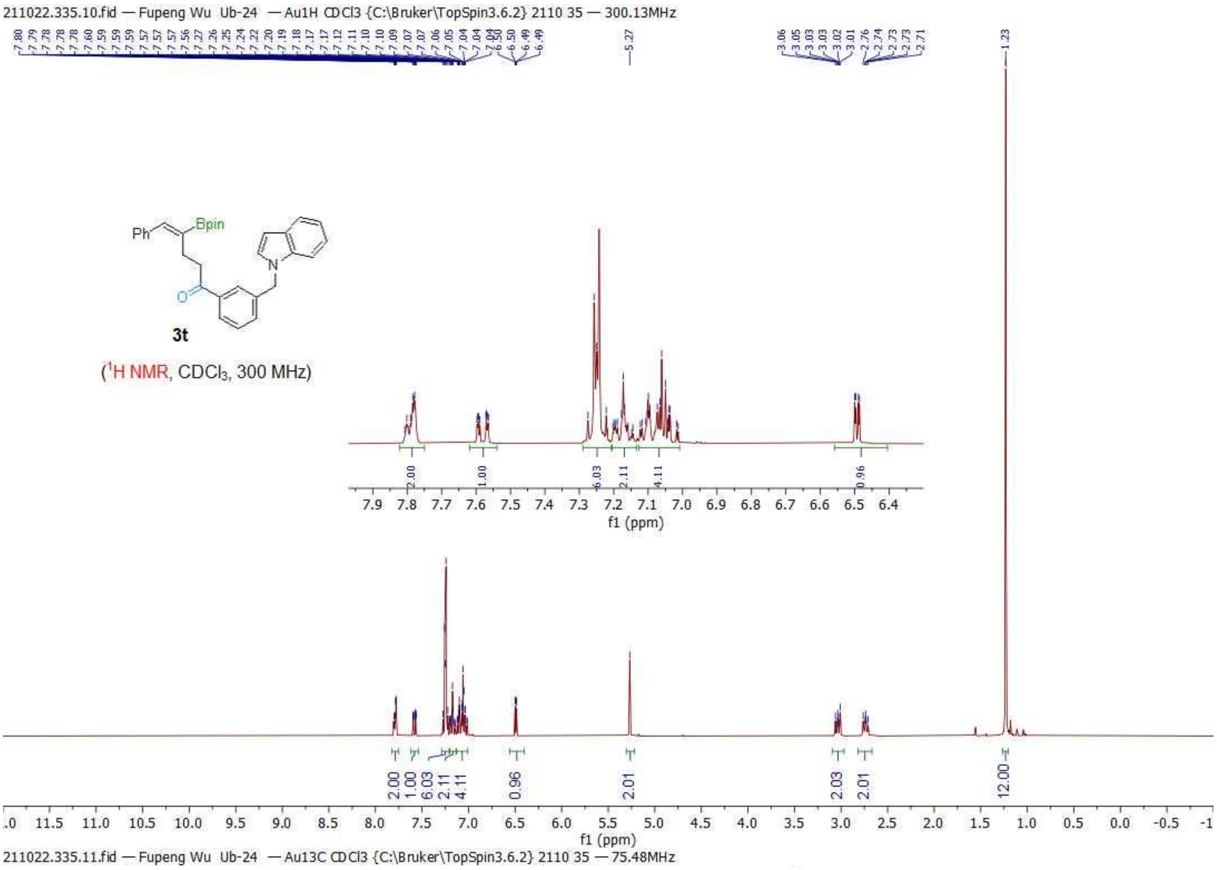

211022.335.10.fid — Fupeng Wu Ub-24 — Au1H CDCl3 {C:\Bruker\TopSpin3.6.2} 2110 35 — 300.13MHz
5.27
3.06 3.05 3.03 3.03 3.02 3.01 2.76 2.74 2.73 2.73 2.71
1.23
Ph
Bpin
N
O
3t
(1H NMR, CDCl3, 300 MHz)
2.00 1.00 6.03 2.11 4.11 0.96
2.00 1.00 6.03 2.11 4.11 0.96 2.01 2.03 2.01 12.00
f1 (ppm)

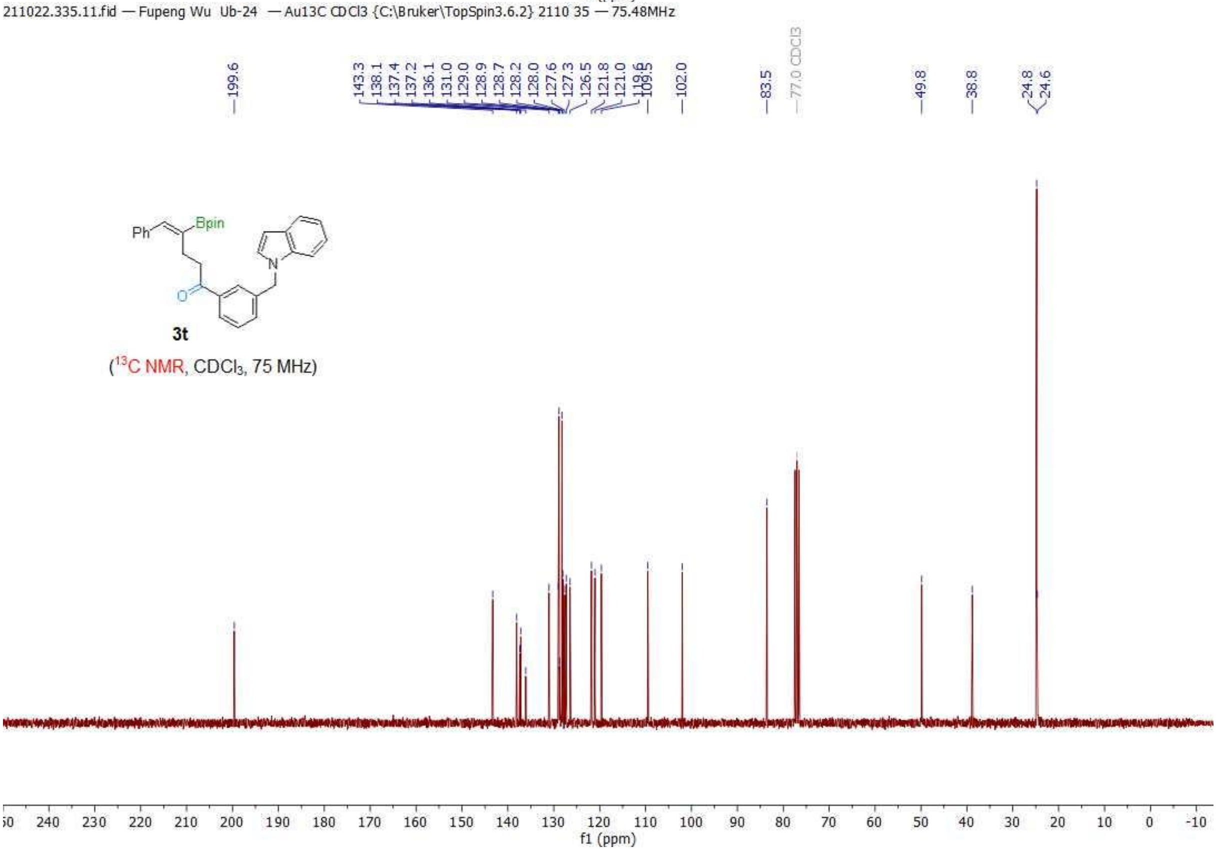

211022.335.11.fid — Fupeng Wu Ub-24 — Au13C CDCl3 {C:\Bruker\TopSpin3.6.2} 2110 35 — 75.48MHz
199.6
143.3 138.1 137.4 137.2 136.1 131.0 129.0 128.9 128.7 128.2 128.0 127.6 127.3 126.5 121.8 121.0 119.6 109.5
102.0
83.5
77.0 CDCl3
49.8
38.8
24.8 24.6
Ph
Bpin
N
O
3t
(13C NMR, CDCl3, 75 MHz)
f1 (ppm)

211022.335.12.fid — Fupeng Wu Ub-24 — Au11B CDCl3 {C:\Bruker\TopSpin3.6.2} 2110 35 — 96.29MHz

—29.9

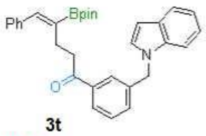

3t

(^{11}B NMR, $CDCl_3$, 96 MHz)

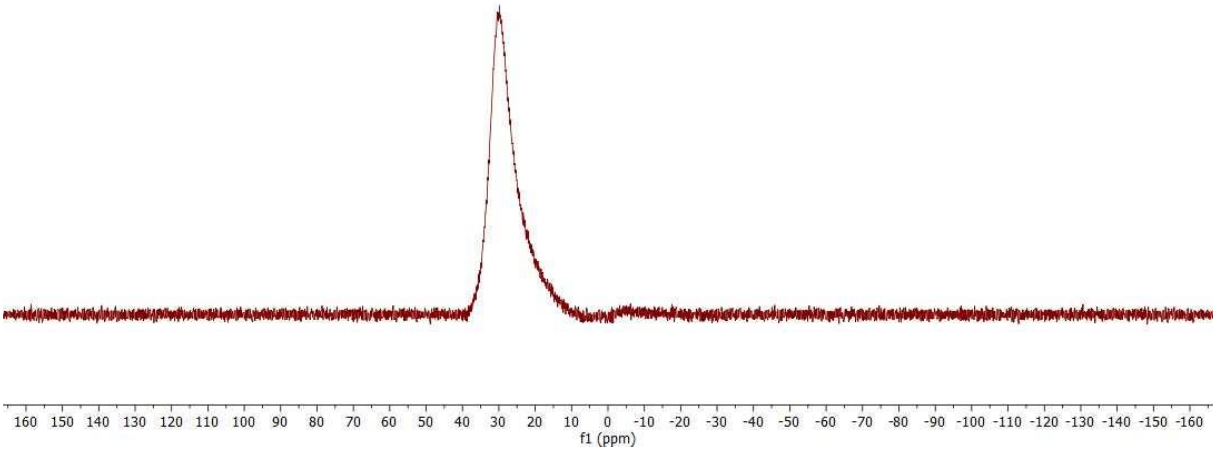

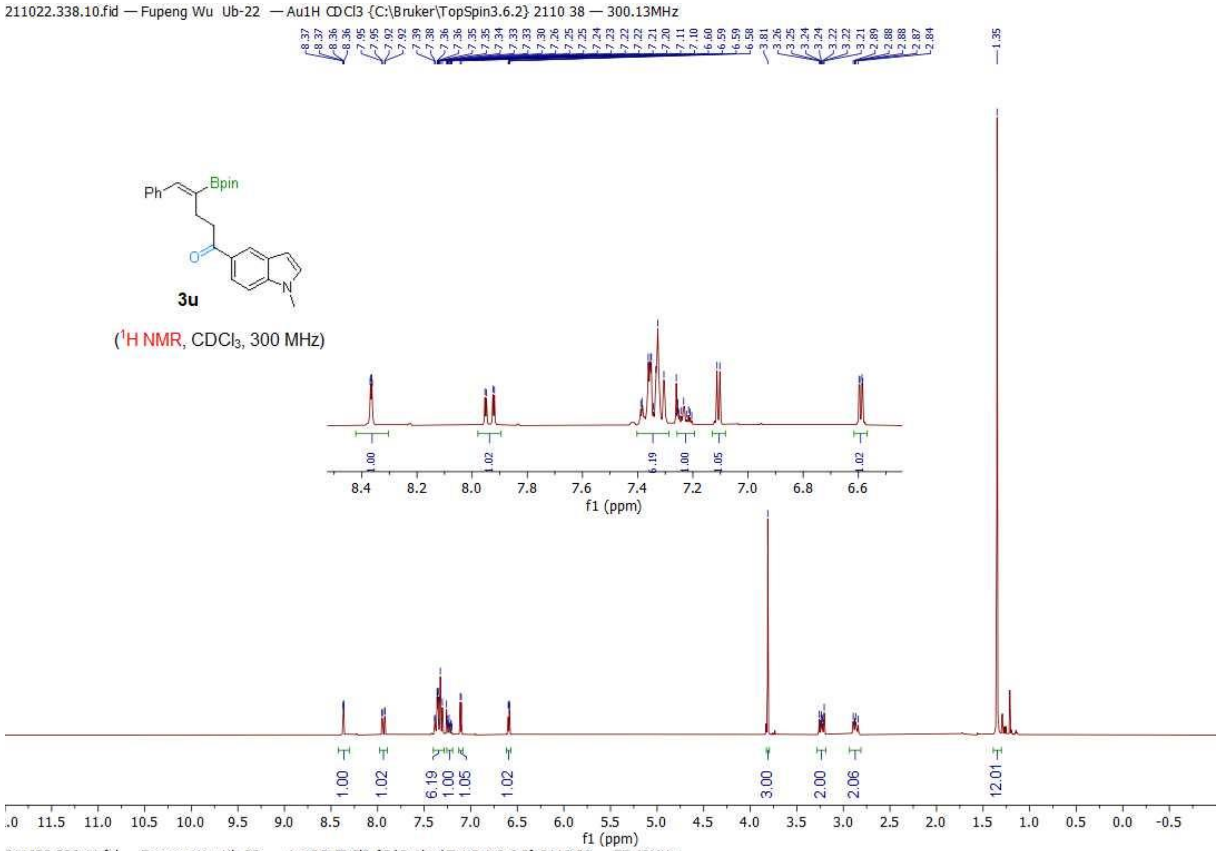

211022.338.10.fid — Fupeng Wu Ub-22 — Au1H CDCl3 {C:\Bruker\TopSpin3.6.2} 2110 38 — 300.13MHz
8.37 8.37 8.36 8.36 7.95 7.95 7.92 7.92 7.39 7.38 7.36 7.36 7.35 7.35 7.34 7.33 7.33 7.30 7.26 7.25 7.25 7.24 7.23 7.22 7.22 7.21 7.20 7.11 7.10 6.60 6.59 6.59 6.58 3.81 3.26 3.25 3.24 3.24 3.22 3.22 3.21 2.89 2.88 2.88 2.87 2.84 1.35
Ph
Bpin
O
N
3u
(^{1}H NMR, $CDCl_3$, 300 MHz)
1.00 1.02 6.19 1.00 1.05 1.02
f1 (ppm)
1.00 1.02 6.19 1.00 1.05 1.02 3.00 2.00 2.06 12.01
f1 (ppm)

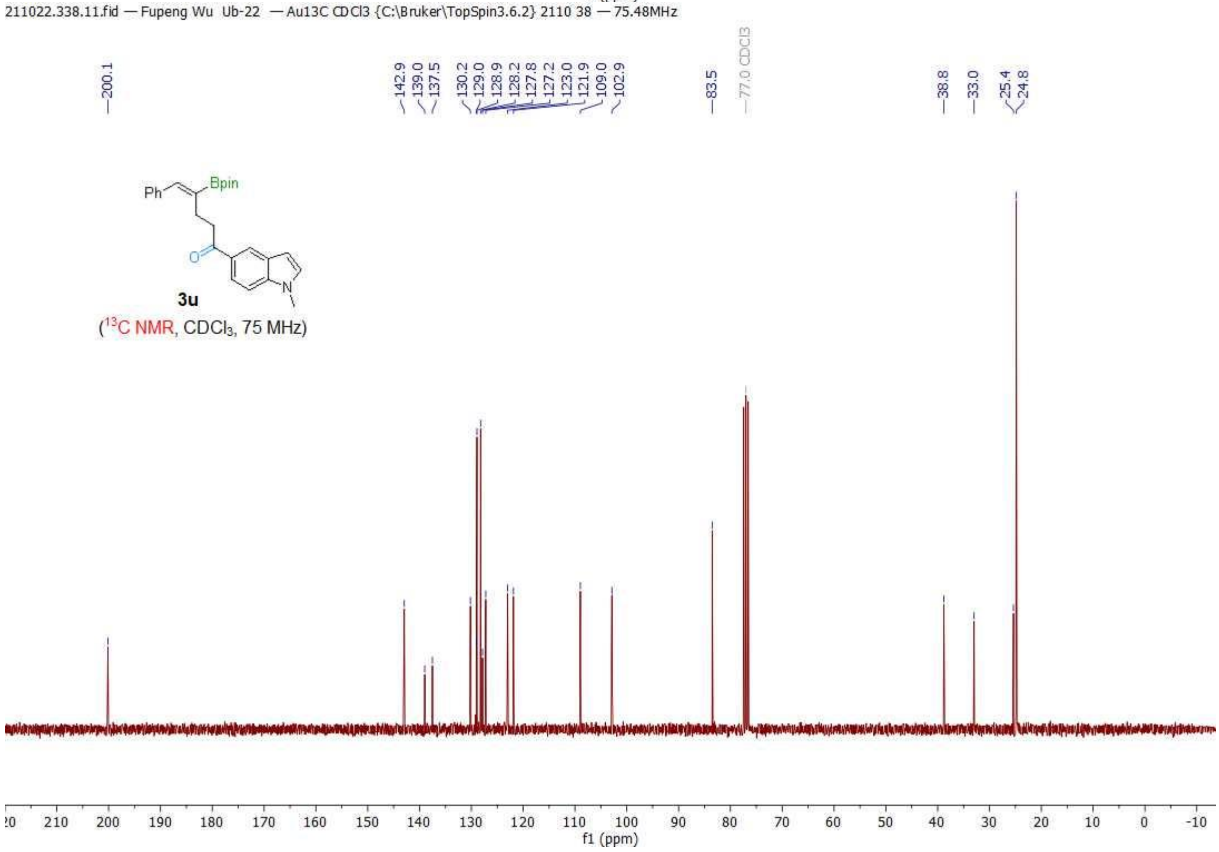

211022.338.11.fid — Fupeng Wu Ub-22 — Au13C CDCl3 {C:\Bruker\TopSpin3.6.2} 2110 38 — 75.48MHz
200.1
142.9 139.0 137.5 130.2 129.0 128.9 128.2 127.8 127.2 123.0 121.9 109.0 102.9
83.5
77.0 CDCl3
38.8 33.0 25.4 24.8
Ph
Bpin
O
N
3u
(^{13}C NMR, $CDCl_3$, 75 MHz)
f1 (ppm)

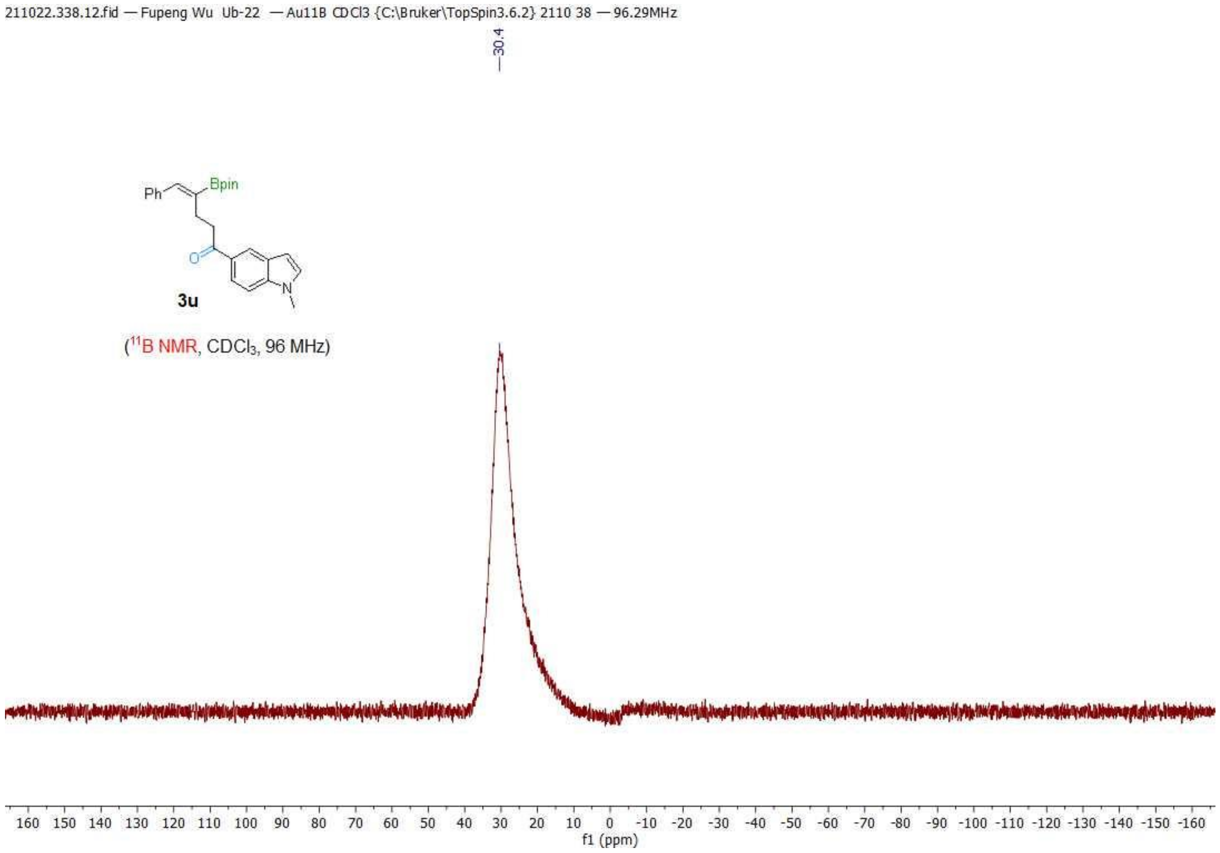

211022.338.12.fid — Fupeng Wu Ub-22 — Au11B CDCl3 {C:\Bruker\TopSpin3.6.2} 2110 38 — 96.29MHz
30.4
Ph
Bpin
O
N
3u
(11B NMR, CDCl3, 96 MHz)
160 150 140 130 120 110 100 90 80 70 60 50 40 30 20 10 0 -10 -20 -30 -40 -50 -60 -70 -80 -90 -100 -110 -120 -130 -140 -150 -160
f1 (ppm)

211027.f355.10.fid — Fupeng Wu u-16-3 — PROTON DMSO {C:\Bruker\TopSpin3.6.2} 2110 55 — 300.20MHz

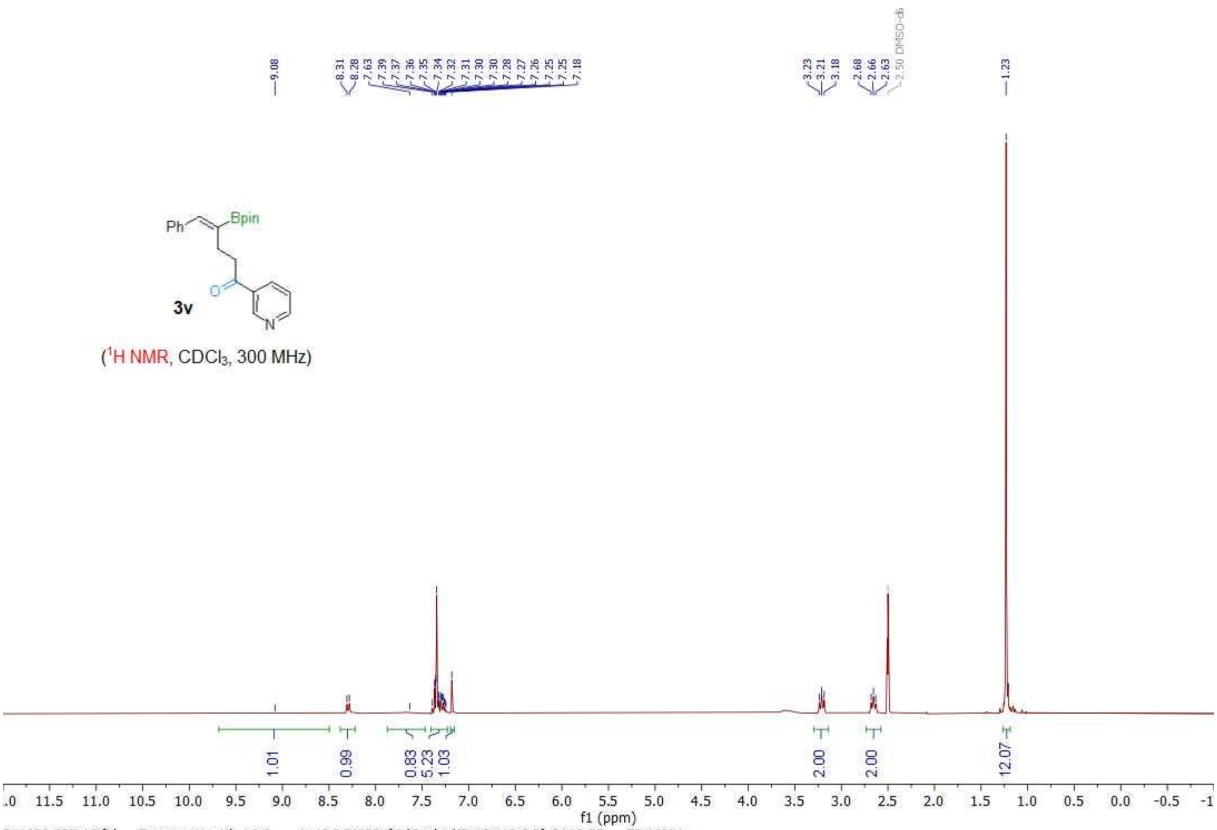

211029.327.10.fid — Fupeng Wu Ub-16-3 — Au13C DMSO {C:\Bruker\TopSpin3.6.2} 2110 27 — 75.48MHz

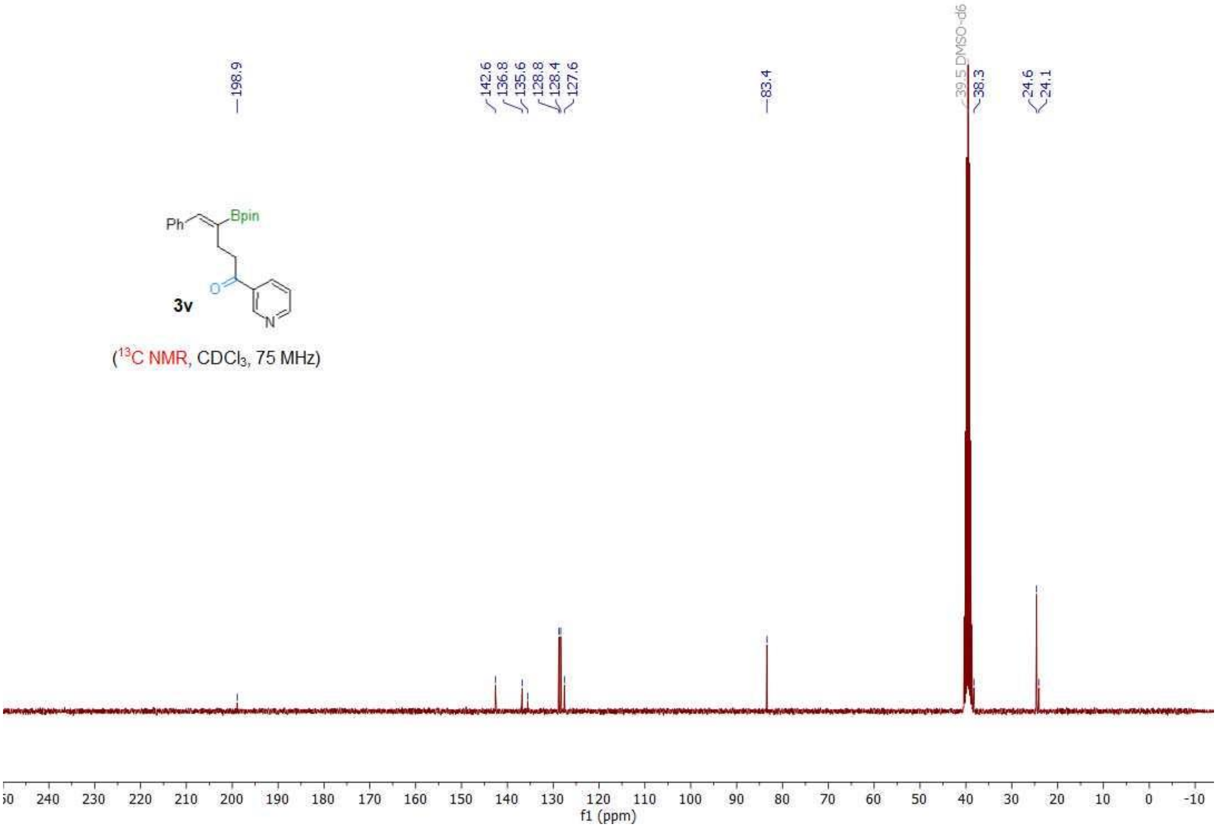

211021.f343.12.fid — Fupeng Wu Ub-16 — 11B CDCl3 {C:\Bruker\TopSpin3.6.2} 2110 43 — 96.32MHz

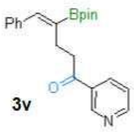

3v

(11B NMR, $CDCl_3$, 96 MHz)

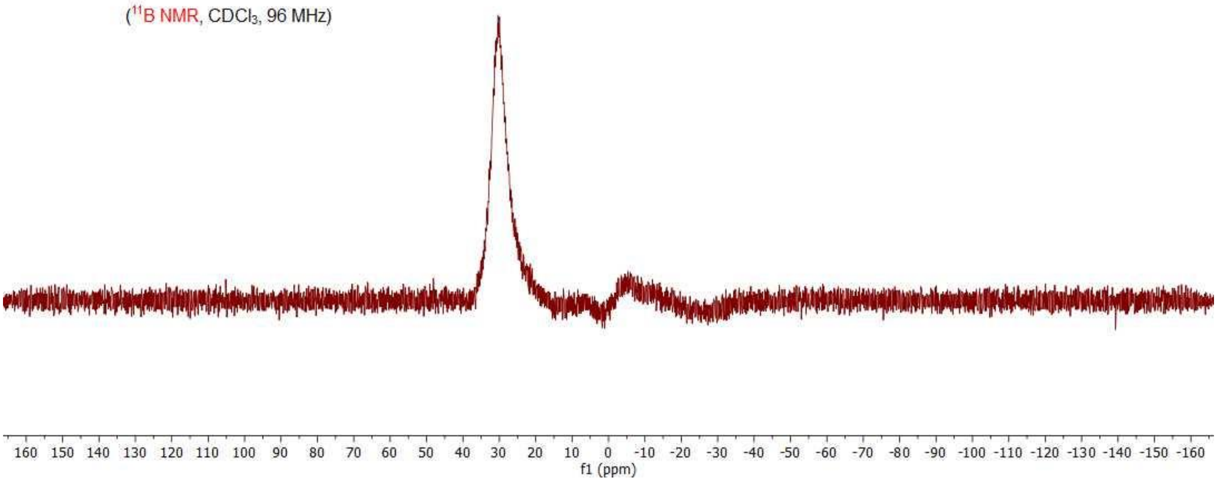

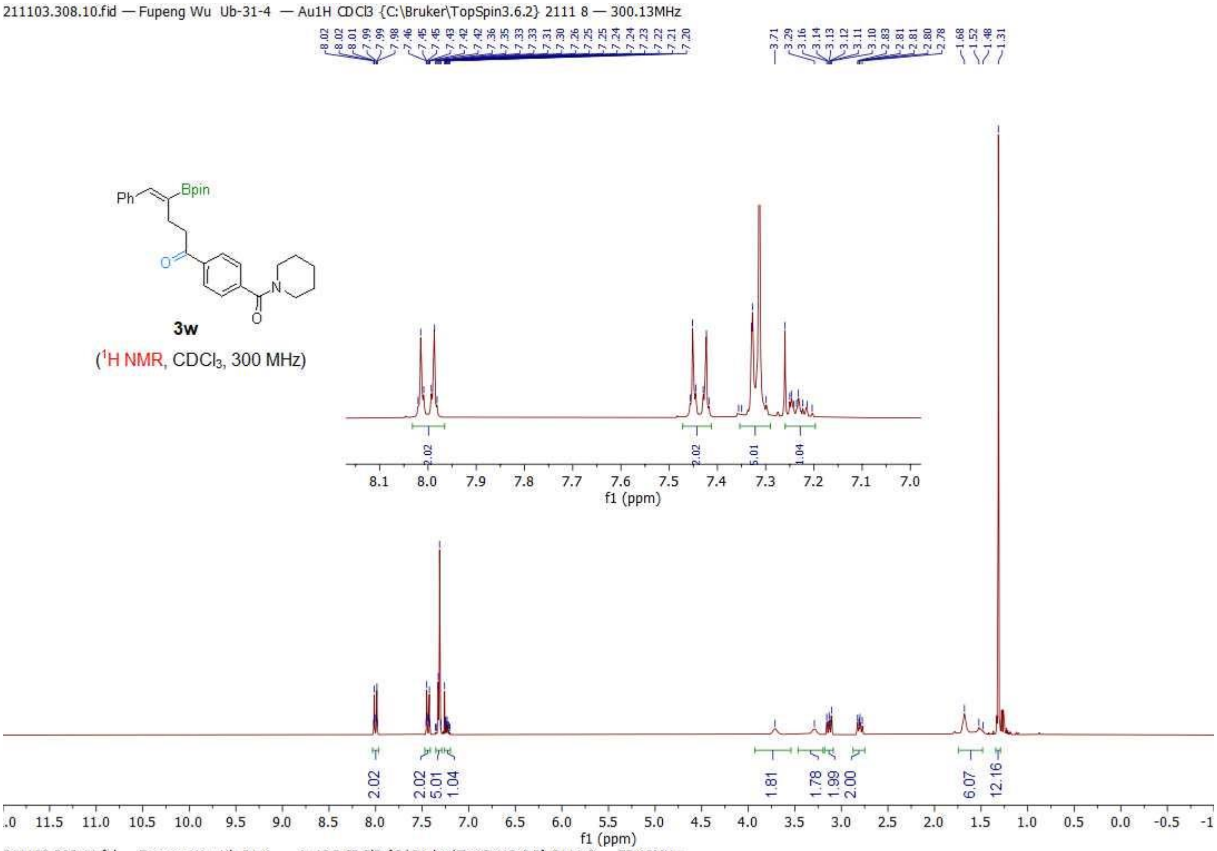
211103.308.10.fid — Fupeng Wu Ub-31-4 — Au1H CDCl3 {C:\Bruker\TopSpin3.6.2} 2111 8 — 300.13MHz
Ph
Bpin
O
N
O
3w
(1H NMR, CDCl3, 300 MHz)
2.02
2.02
5.01
1.04
1.81
1.78
1.99
2.00
6.07
12.16
f1 (ppm)

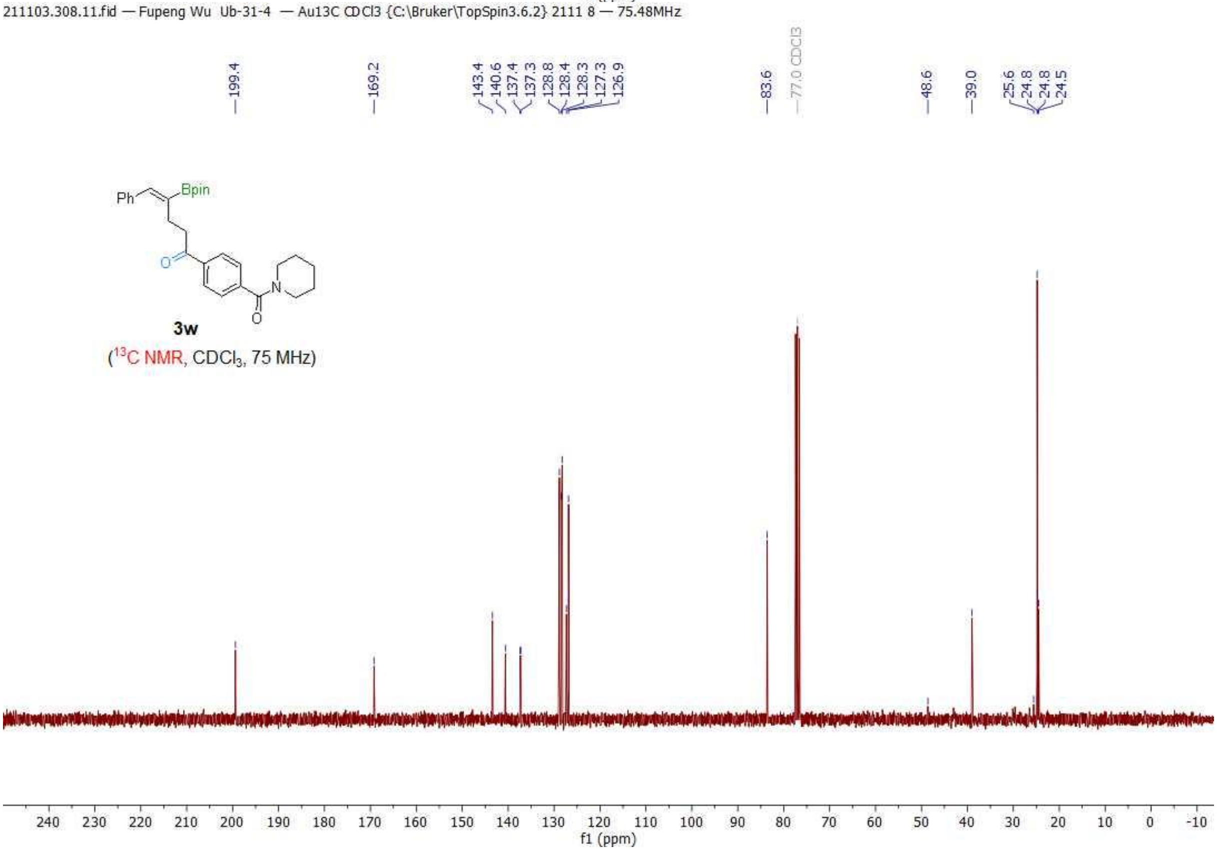
211103.308.11.fid — Fupeng Wu Ub-31-4 — Au13C CDCl3 {C:\Bruker\TopSpin3.6.2} 2111 8 — 75.48MHz
199.4
169.2
143.4
140.6
137.4
137.3
128.8
128.4
128.3
127.3
126.9
83.6
77.0 CDCl3
48.6
39.0
25.6
24.8
24.8
24.5
Ph
Bpin
O
N
O
3w
(13C NMR, CDCl3, 75 MHz)
f1 (ppm)

211026.328.12.fid — Fupeng Wu, Ub-31 — Au11B CDCl3 {C:\Bruker\TopSpin3.6.2} 2110 28 — 96.29MHz

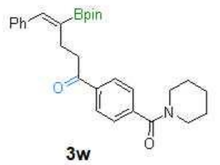

3w

(^{11}B NMR, $CDCl_3$, 96 MHz)

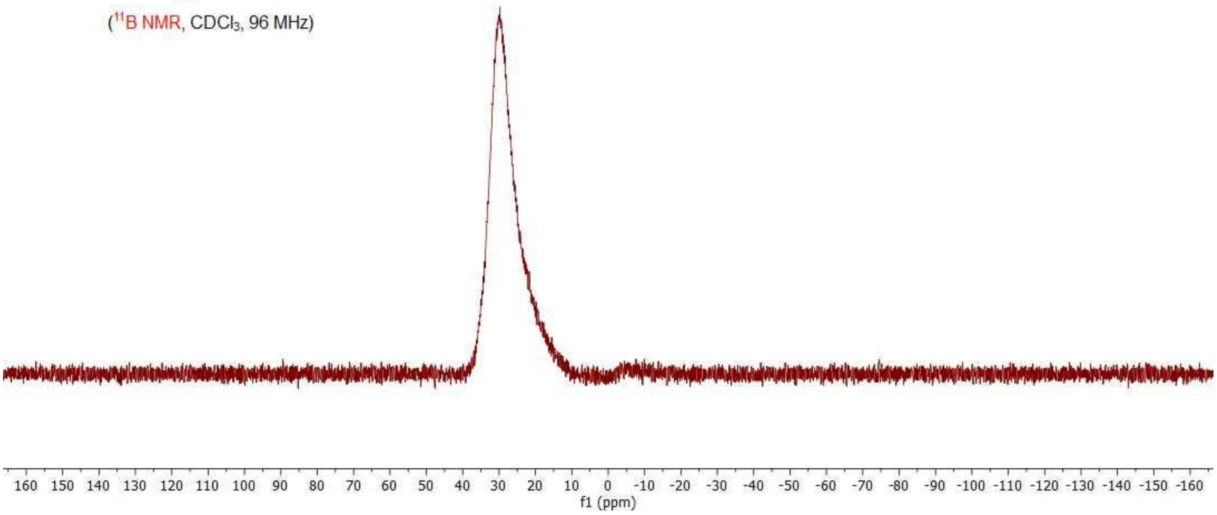

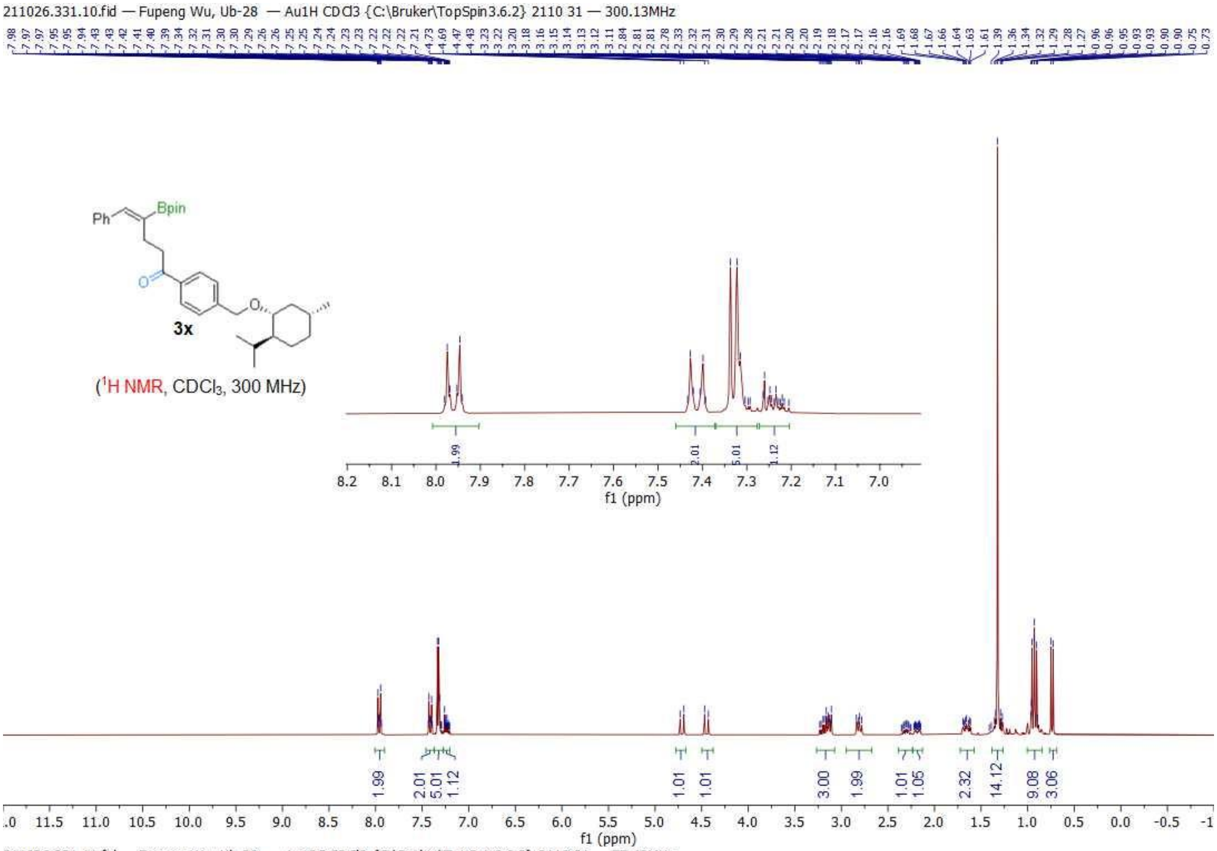
211026.331.10.fid — Fupeng Wu, Ub-28 — Au1H CDCl3 {C:\Bruker\TopSpin3.6.2} 2110 31 — 300.13MHz
Ph
Bpin
O
3x
(1H NMR, CDCl3, 300 MHz)
1.99
2.01
5.01
1.12
1.01
1.01
3.00
1.99
1.01
1.05
2.32
14.12
9.08
3.06
f1 (ppm)

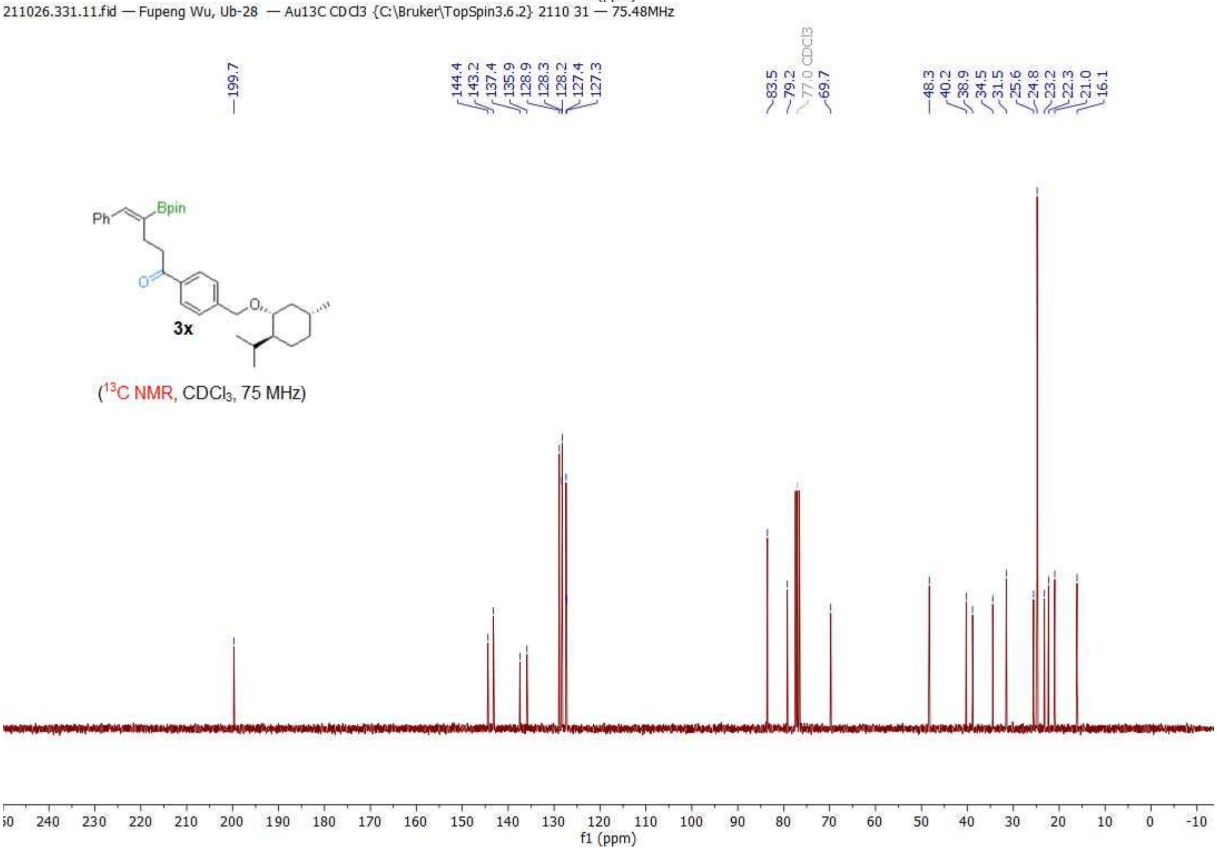
211026.331.11.fid — Fupeng Wu, Ub-28 — Au13C CDCl3 {C:\Bruker\TopSpin3.6.2} 2110 31 — 75.48MHz
199.7
144.4
143.2
137.4
135.9
128.9
128.3
128.2
127.4
127.3
83.5
79.2
77.0 CDCl3
69.7
48.3
40.2
38.9
34.5
31.5
25.6
24.8
23.2
22.3
21.0
16.1
Ph
Bpin
O
3x
(13C NMR, CDCl3, 75 MHz)
f1 (ppm)

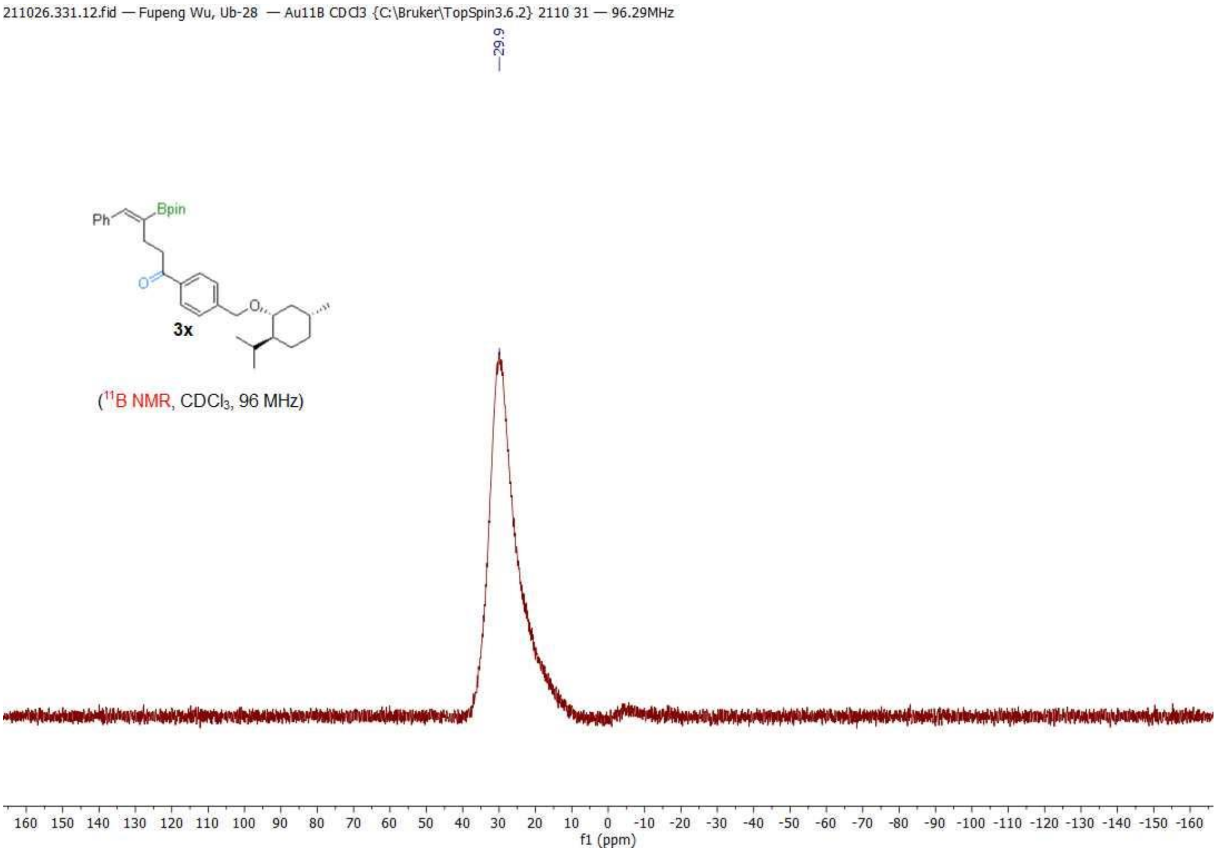
211026.331.12.fid — Fupeng Wu, Ub-28 — Au11B CDCl3 {C:\Bruker\TopSpin3.6.2} 2110 31 — 96.29MHz
29.9
Ph
Bpin
O
O
3x
(11B NMR, CDCl3, 96 MHz)
160 150 140 130 120 110 100 90 80 70 60 50 40 30 20 10 0 -10 -20 -30 -40 -50 -60 -70 -80 -90 -100 -110 -120 -130 -140 -150 -160
f1 (ppm)

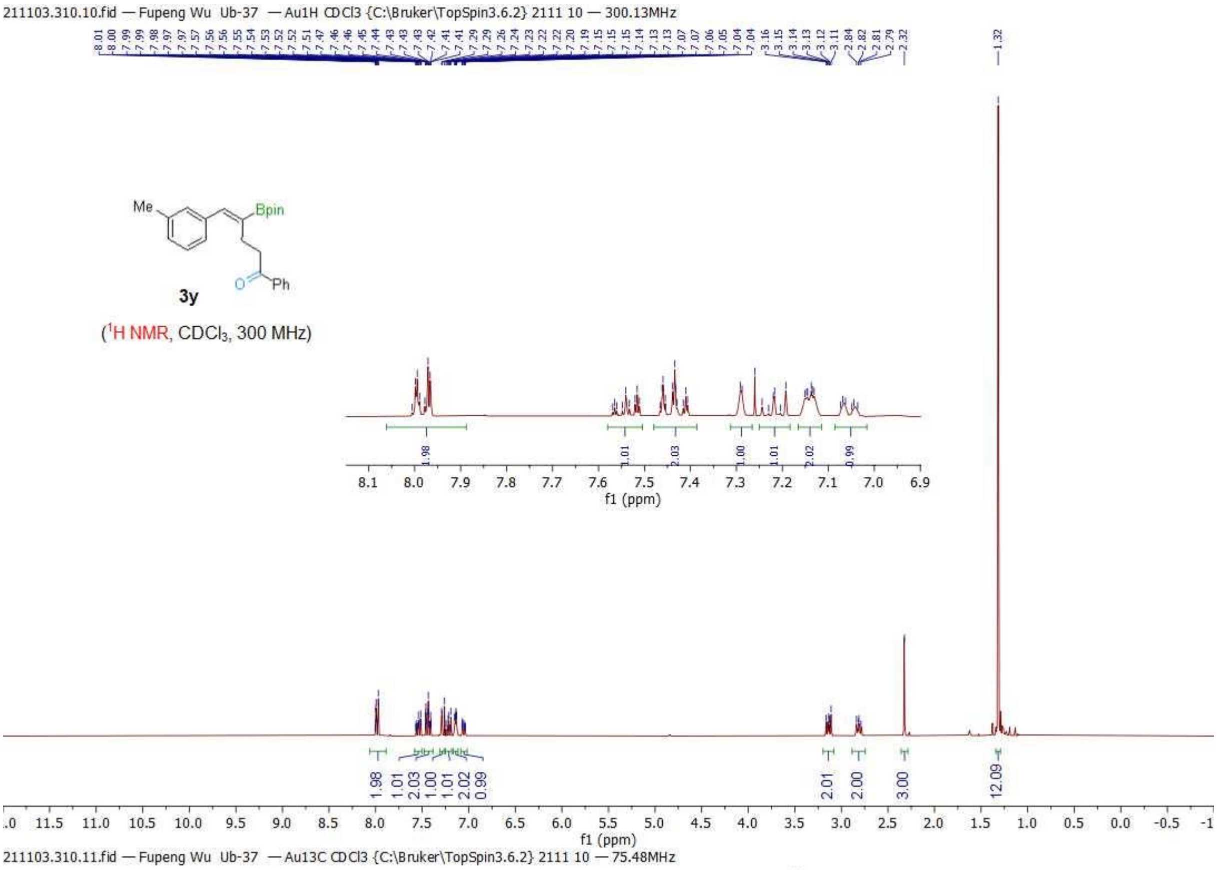

211103.310.10.fid — Fupeng Wu Ub-37 — Au1H CDCl3 {C:\Bruker\TopSpin3.6.2} 2111 10 — 300.13MHz
Me
Bpin
O
Ph
3y
(1H NMR, CDCl3, 300 MHz)
f1 (ppm)

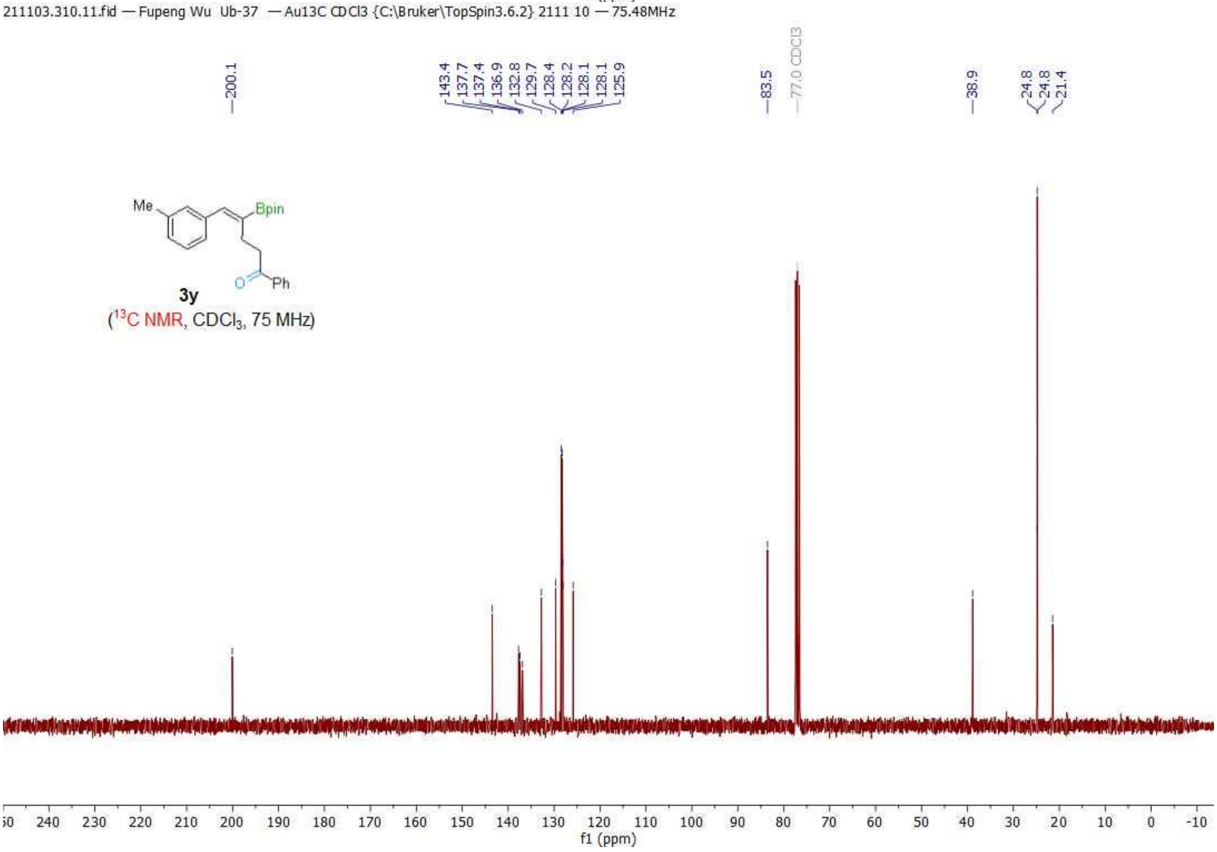

211103.310.11.fid — Fupeng Wu Ub-37 — Au13C CDCl3 {C:\Bruker\TopSpin3.6.2} 2111 10 — 75.48MHz
200.1
143.4
137.7
137.4
136.9
132.8
129.7
128.4
128.2
128.1
125.9
83.5
77.0 CDCl3
38.9
24.8
24.8
21.4
Me
Bpin
O
Ph
3y
(13C NMR, CDCl3, 75 MHz)
f1 (ppm)

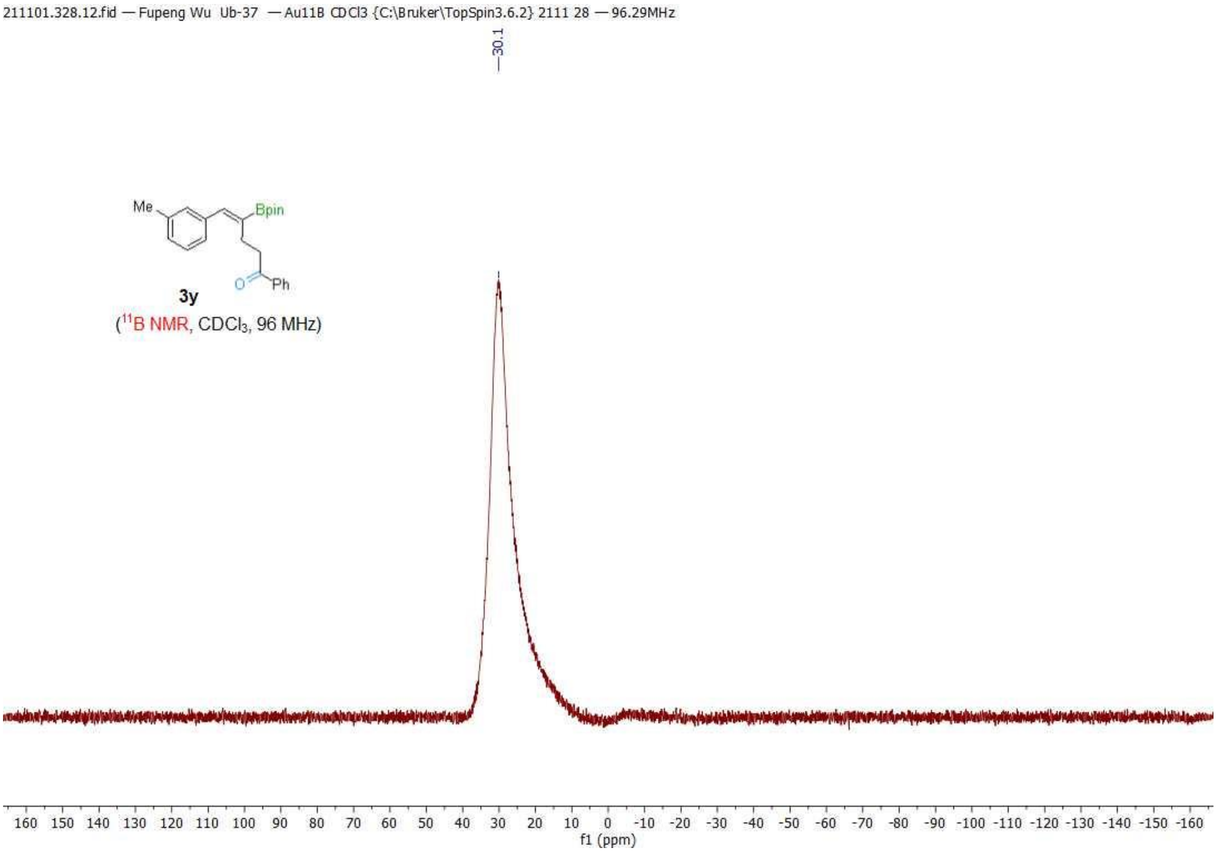

211101.328.12.fid — Fupeng Wu Ub-37 — Au11B CDCl3 {C:\Bruker\TopSpin3.6.2} 2111 28 — 96.29MHz
30.1
Me
Bpin
O
Ph
3y
(11B NMR, CDCl3, 96 MHz)
160 150 140 130 120 110 100 90 80 70 60 50 40 30 20 10 0 -10 -20 -30 -40 -50 -60 -70 -80 -90 -100 -110 -120 -130 -140 -150 -160
f1 (ppm)

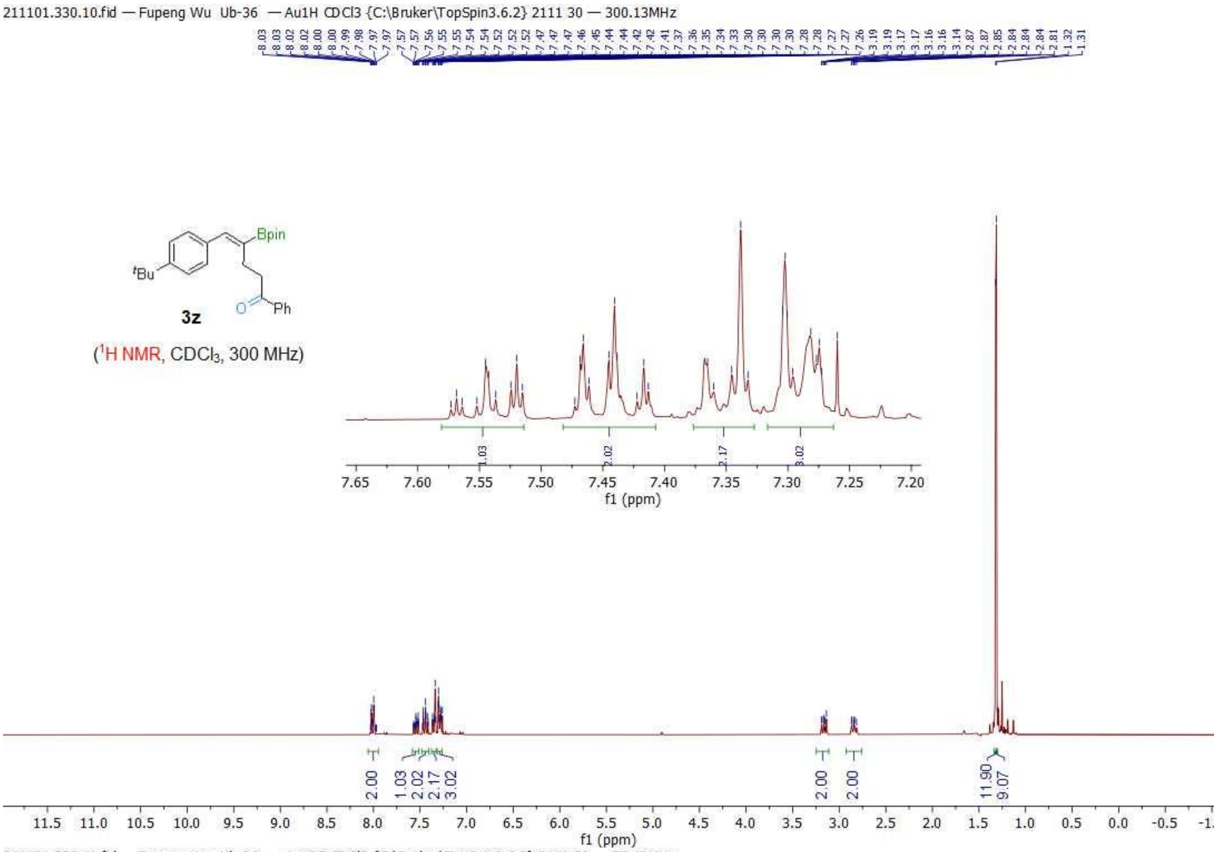
211101.330.10.fid — Fupeng Wu Ub-36 — Au1H CDCl3 {C:\Bruker\TopSpin3.6.2} 2111 30 — 300.13MHz
Bpin
^{t}Bu
O
Ph
3z
(^{1}H NMR, CDCl$_3$, 300 MHz)
f1 (ppm)

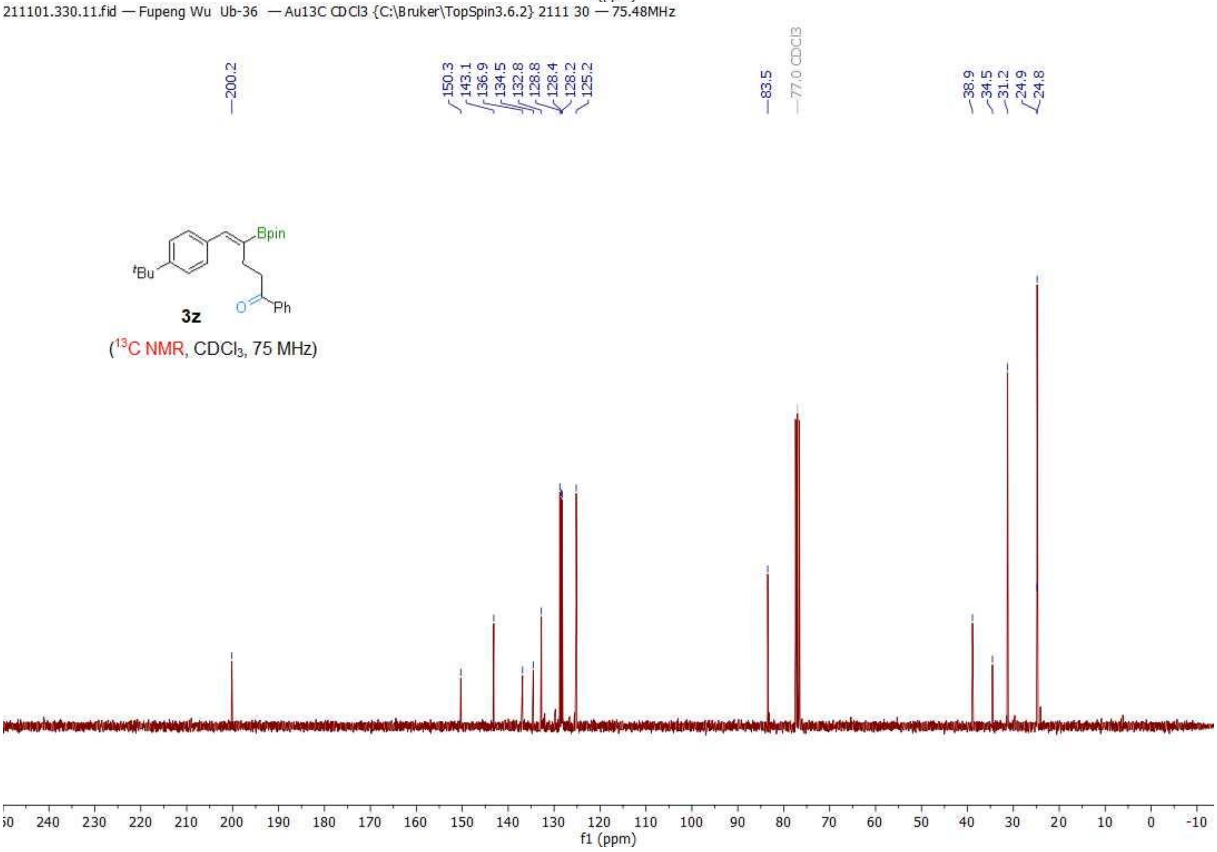
211101.330.11.fid — Fupeng Wu Ub-36 — Au13C CDCl3 {C:\Bruker\TopSpin3.6.2} 2111 30 — 75.48MHz
200.2
150.3
143.1
136.9
134.5
132.8
128.8
128.4
128.2
125.2
83.5
77.0 CDCl3
38.9
34.5
31.2
24.9
24.8
Bpin
^{t}Bu
O
Ph
3z
(^{13}C NMR, CDCl$_3$, 75 MHz)
f1 (ppm)

211101.330.12.fid — Fupeng Wu Ub-36 — Au11B CDCl3 {C:\Bruker\TopSpin3.6.2} 2111 30 — 96.29MHz

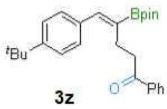

3z

(^{11}B NMR, $CDCl_3$, 96 MHz)

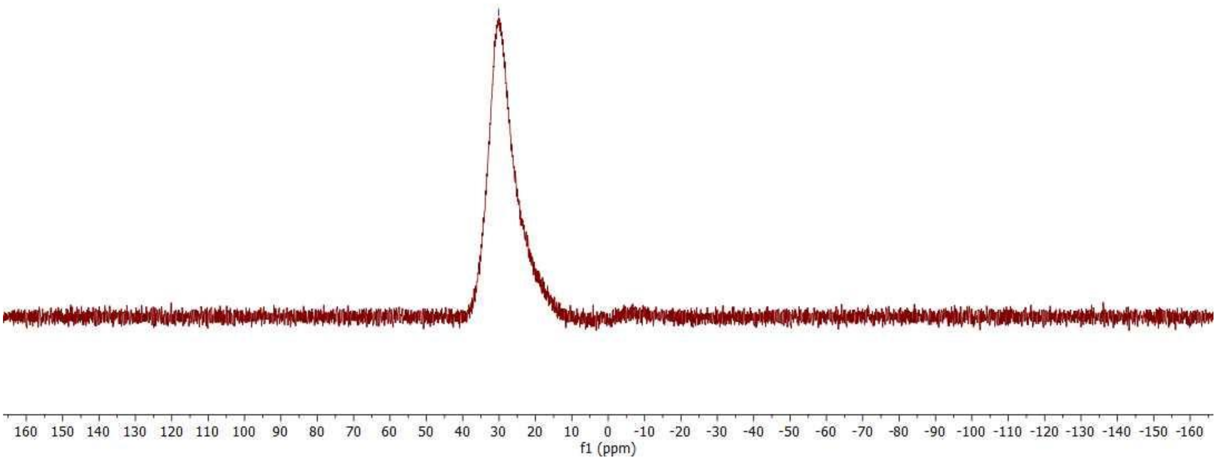

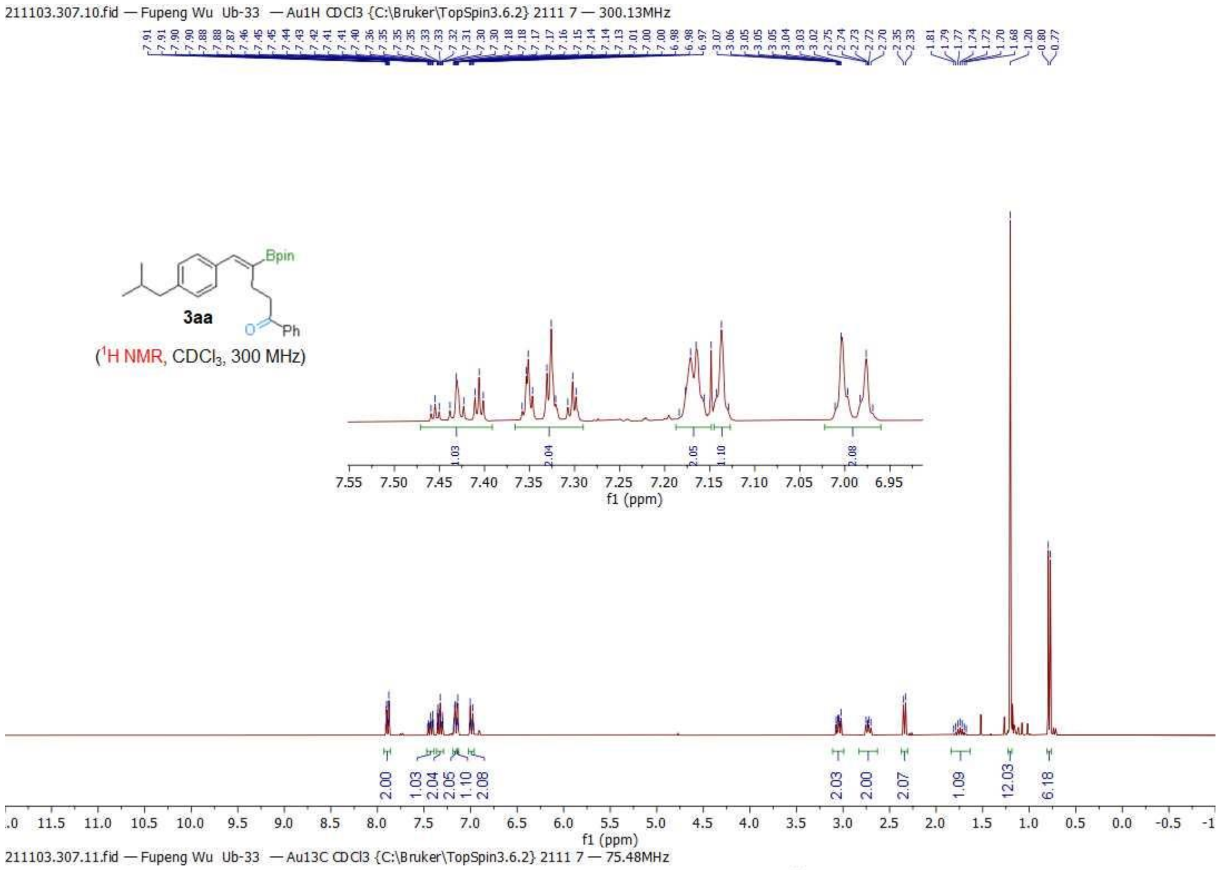
211103.307.10.fid — Fupeng Wu Ub-33 — Au1H CDCl3 {C:\Bruker\TopSpin3.6.2} 2111 7 — 300.13MHz
7.91 7.91 7.90 7.88 7.88 7.87 7.46 7.45 7.45 7.44 7.43 7.42 7.41 7.41 7.40 7.36 7.35 7.35 7.35 7.33 7.33 7.32 7.31 7.30 7.30 7.18 7.18 7.17 7.17 7.16 7.15 7.14 7.13 7.01 7.00 7.00 6.98 6.98 6.97 3.07 3.06 3.05 3.05 3.04 3.03 3.02 2.75 2.74 2.73 2.72 2.70 2.35 2.33 1.81 1.79 1.77 1.74 1.72 1.70 1.68 1.20 0.80 0.77
Bpin
O
Ph
3aa
(^{1}H NMR, CDCl$_3$, 300 MHz)
1.03
2.04
2.05
1.10
2.08
f1 (ppm)
2.00 1.03 2.04 2.05 1.10 2.08 2.03 2.00 2.07 1.09 12.03 6.18
f1 (ppm)

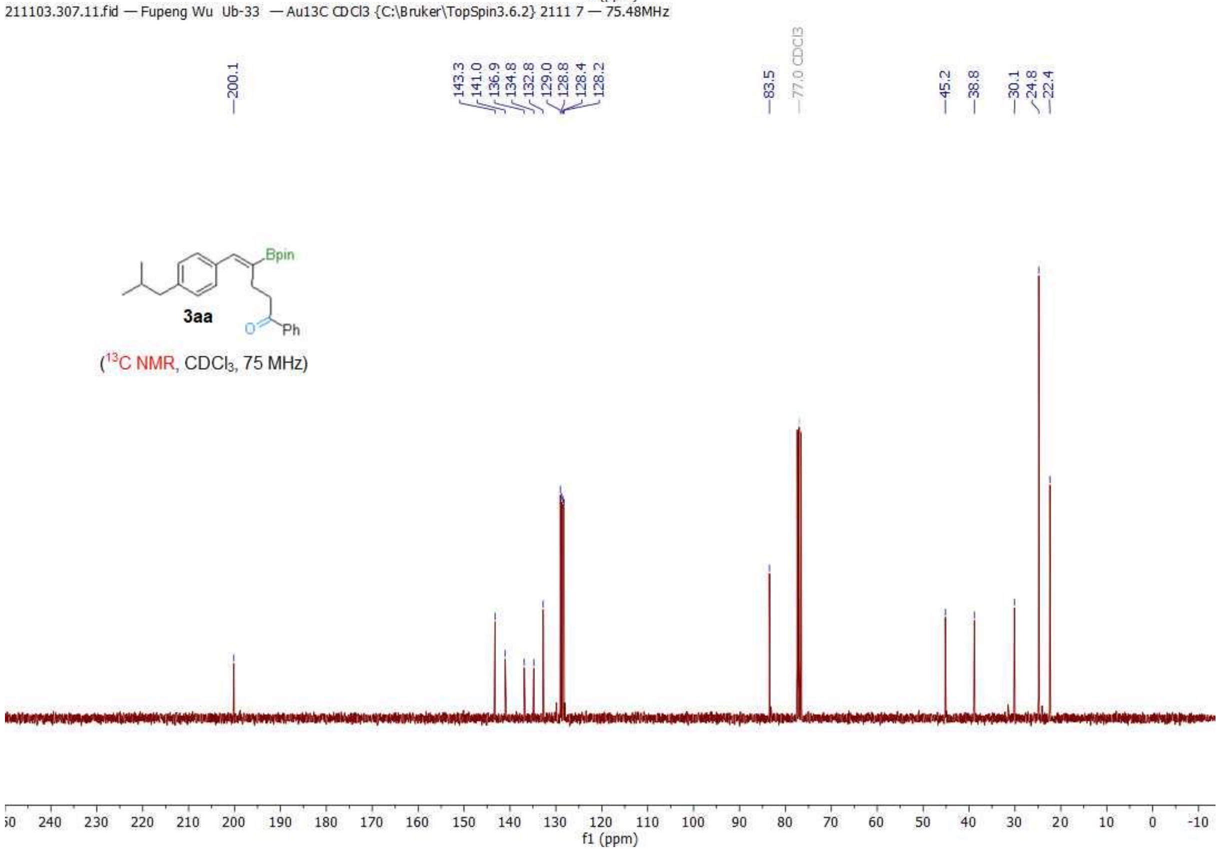
211103.307.11.fid — Fupeng Wu Ub-33 — Au13C CDCl3 {C:\Bruker\TopSpin3.6.2} 2111 7 — 75.48MHz
200.1
143.3 141.0 136.9 134.8 132.8 129.0 128.8 128.4 128.2
83.5
77.0 CDCl3
45.2
38.8
30.1 24.8 22.4
Bpin
O
Ph
3aa
(^{13}C NMR, CDCl$_3$, 75 MHz)
f1 (ppm)

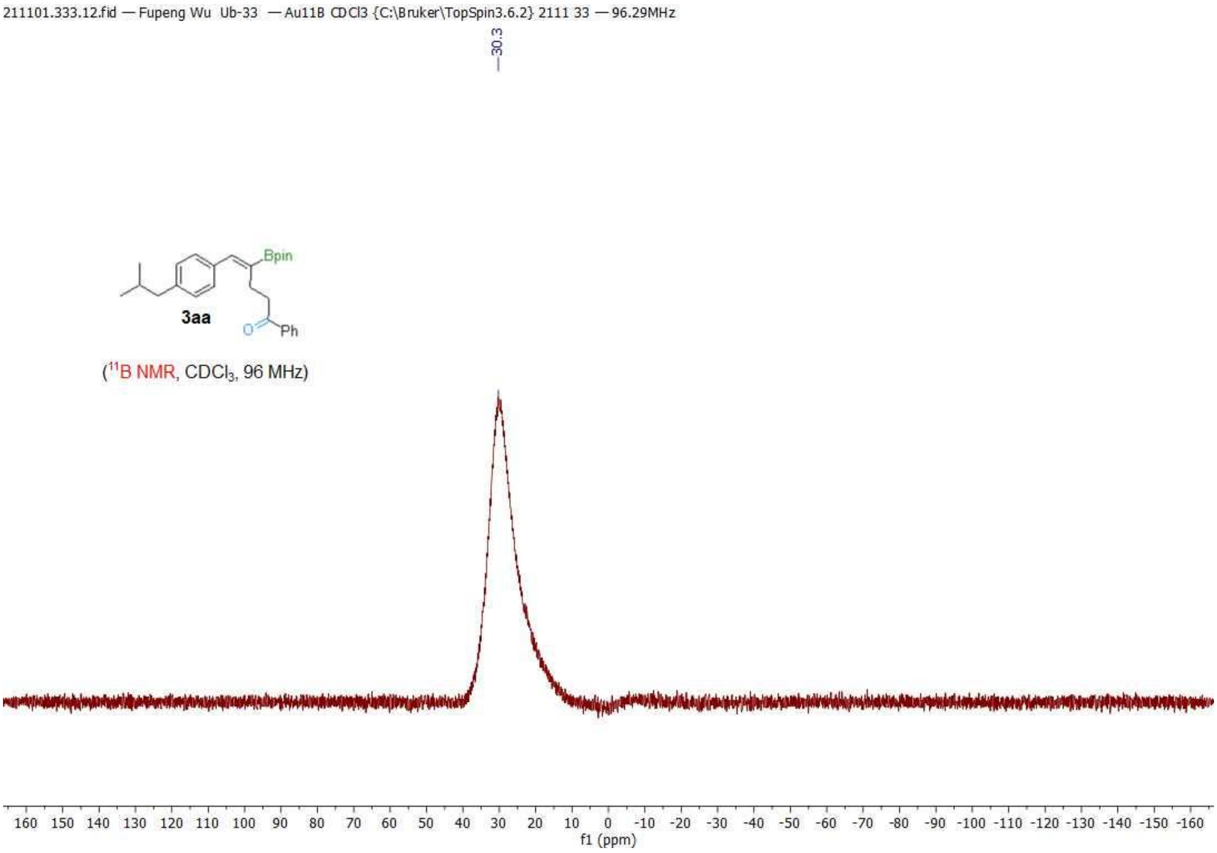
211101.333.12.fid — Fupeng Wu Ub-33 — Au11B CDCl3 {C:\Bruker\TopSpin3.6.2} 2111 33 — 96.29MHz
30.3
Bpin
3aa
O
Ph
(11B NMR, CDCl3, 96 MHz)
f1 (ppm)

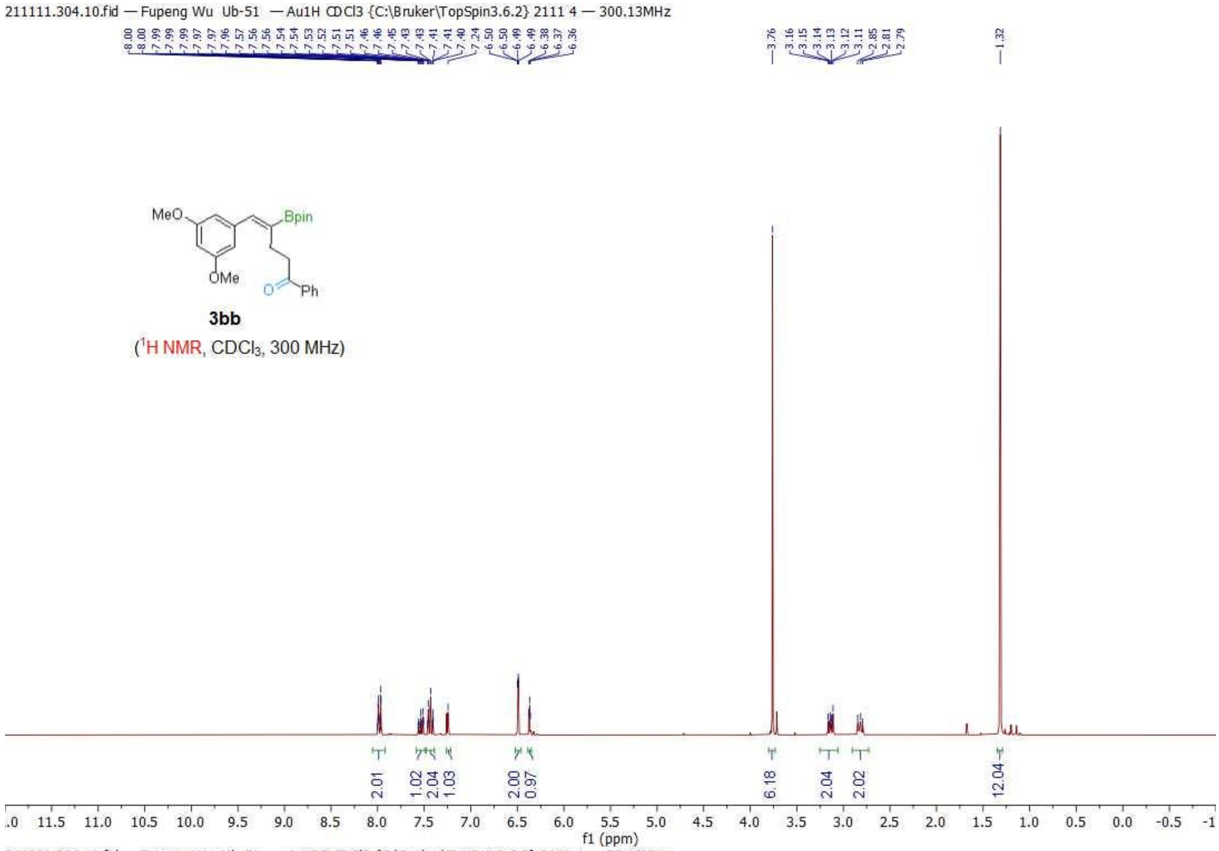
211111.304.10.fid — Fupeng Wu Ub-51 — Au1H CDCl3 {C:\Bruker\TopSpin3.6.2} 2111 4 — 300.13MHz
MeO
Bpin
OMe
O
Ph
3bb
(1H NMR, CDCl3, 300 MHz)
3.76
1.32
2.01
1.02
2.04
1.03
2.00
0.97
6.18
2.04
2.02
12.04
f1 (ppm)

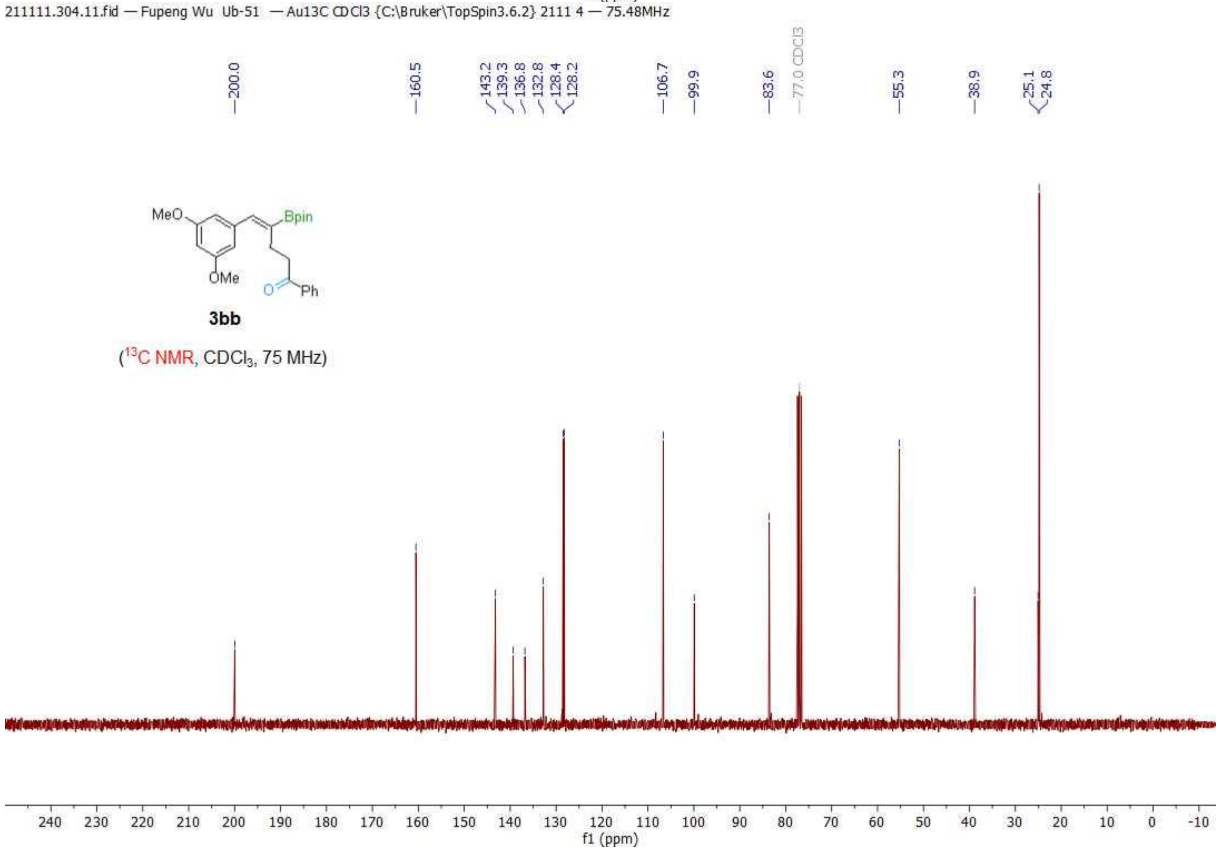
211111.304.11.fid — Fupeng Wu Ub-51 — Au13C CDCl3 {C:\Bruker\TopSpin3.6.2} 2111 4 — 75.48MHz
200.0
160.5
143.2
139.3
136.8
132.8
128.4
128.2
106.7
99.9
83.6
77.0 CDCl3
55.3
38.9
25.1
24.8
MeO
Bpin
OMe
O
Ph
3bb
(13C NMR, CDCl3, 75 MHz)
f1 (ppm)

211109.423.12.fid — Fupeng Wu Ub-51 — Au11B CDCl3 {C:\Bruker\TopSpin3.5pl6} 2111 23 — 128.38MHz

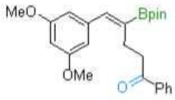

3bb
(^{11}B NMR, $CDCl_3$, 96 MHz)

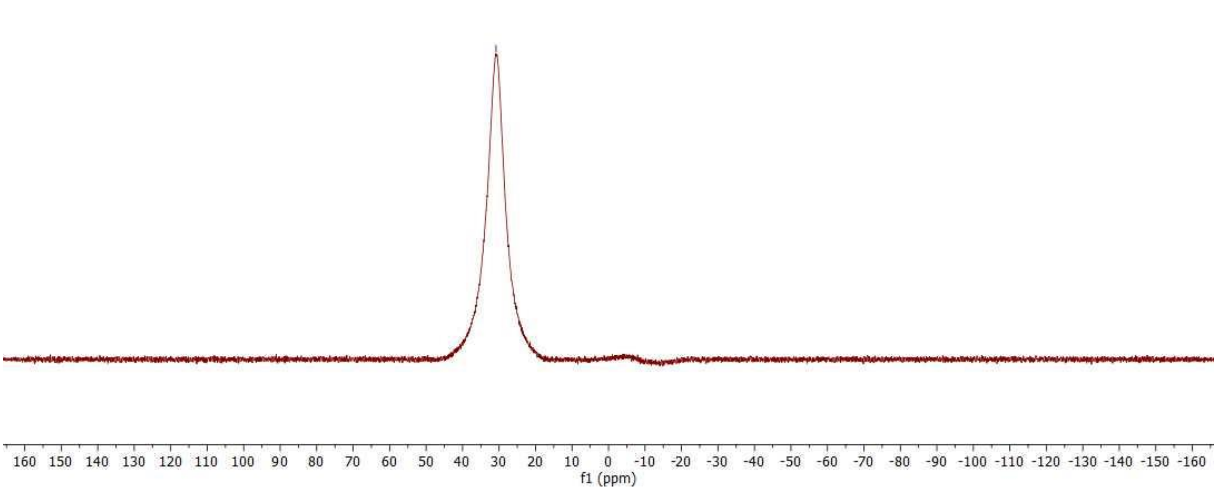

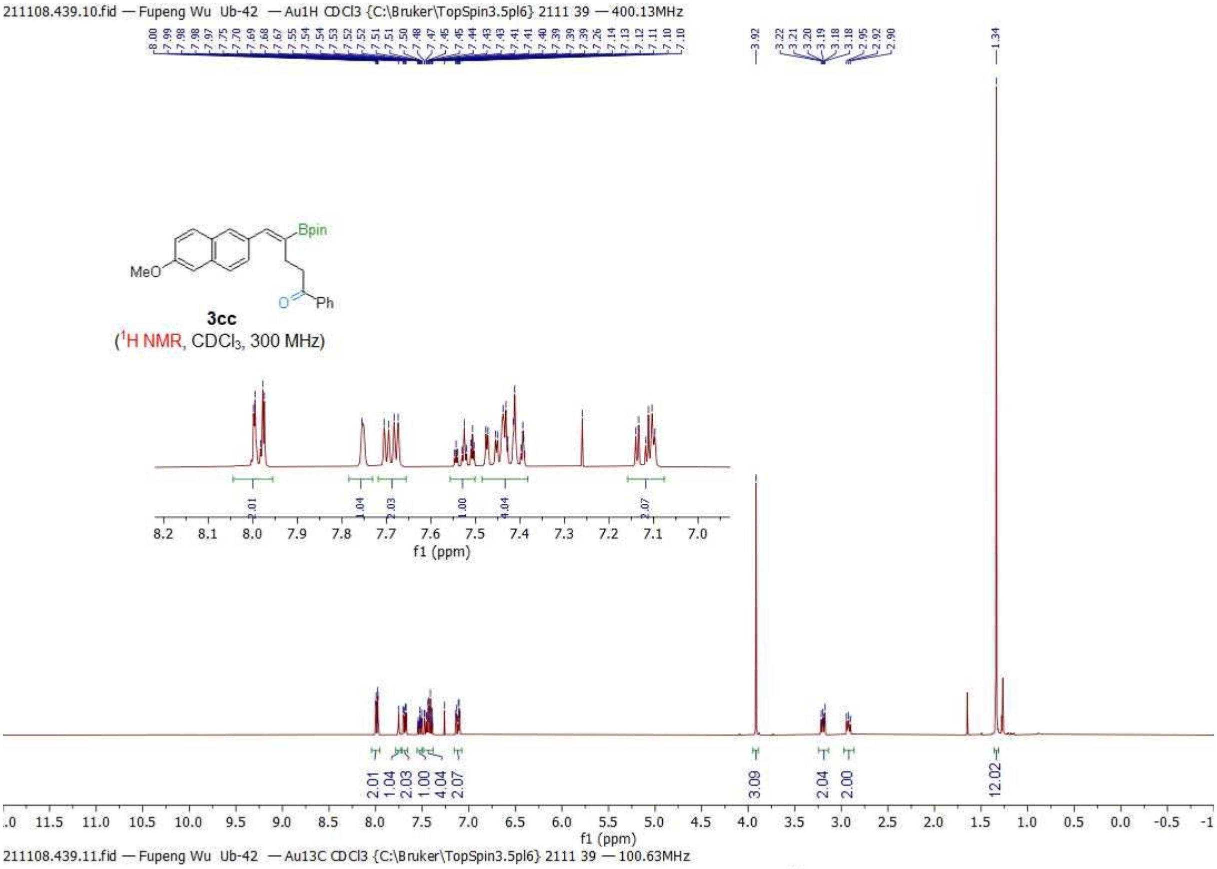
211108.439.10.fid — Fupeng Wu Ub-42 — Au1H CDCl3 {C:\Bruker\TopSpin3.5pl6} 2111 39 — 400.13MHz
MeO
Bpin
O
Ph
3cc
(^{1}H NMR, CDCl$_3$, 300 MHz)
3.92
1.34
f1 (ppm)

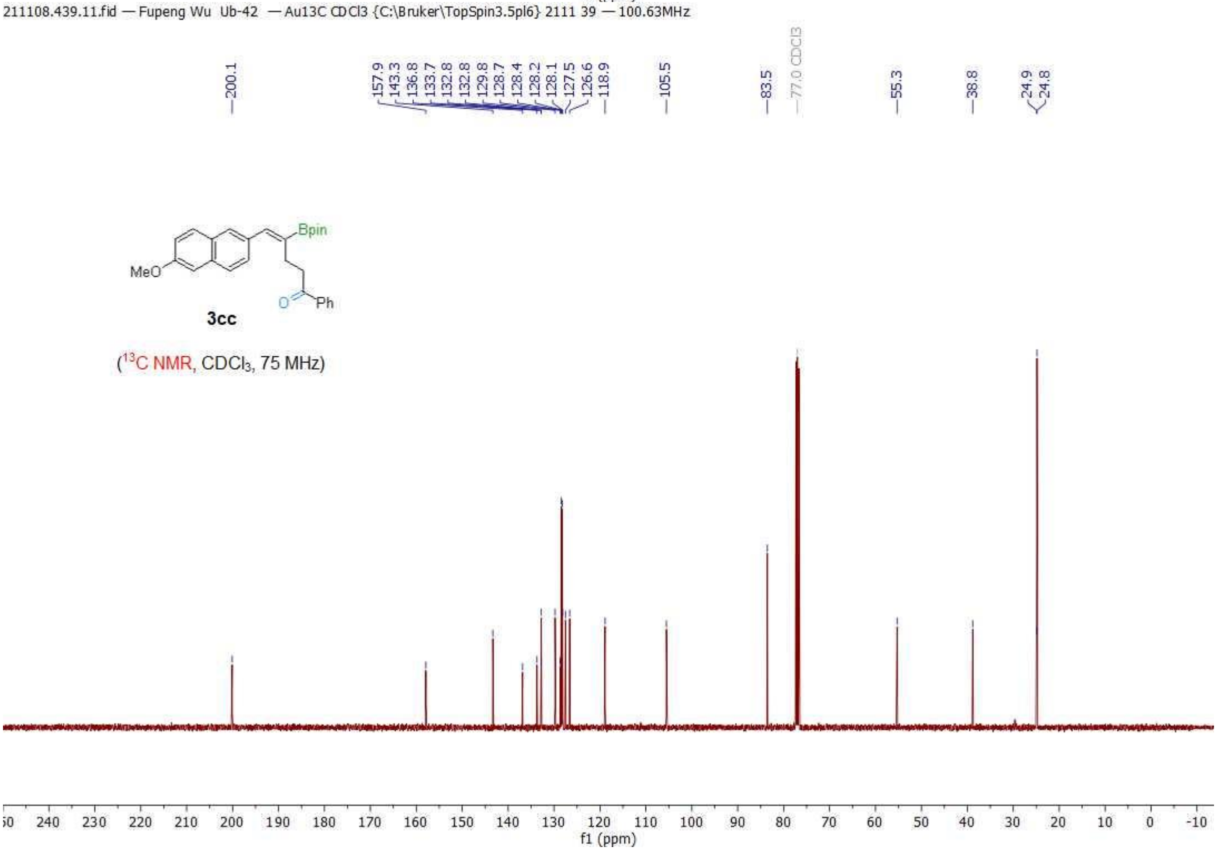
211108.439.11.fid — Fupeng Wu Ub-42 — Au13C CDCl3 {C:\Bruker\TopSpin3.5pl6} 2111 39 — 100.63MHz
200.1
157.9
143.3
136.8
133.7
132.8
129.8
128.7
128.4
128.2
128.1
127.5
126.6
118.9
105.5
83.5
77.0 CDCl3
55.3
38.8
24.9
24.8
MeO
Bpin
O
Ph
3cc
(^{13}C NMR, CDCl$_3$, 75 MHz)
f1 (ppm)

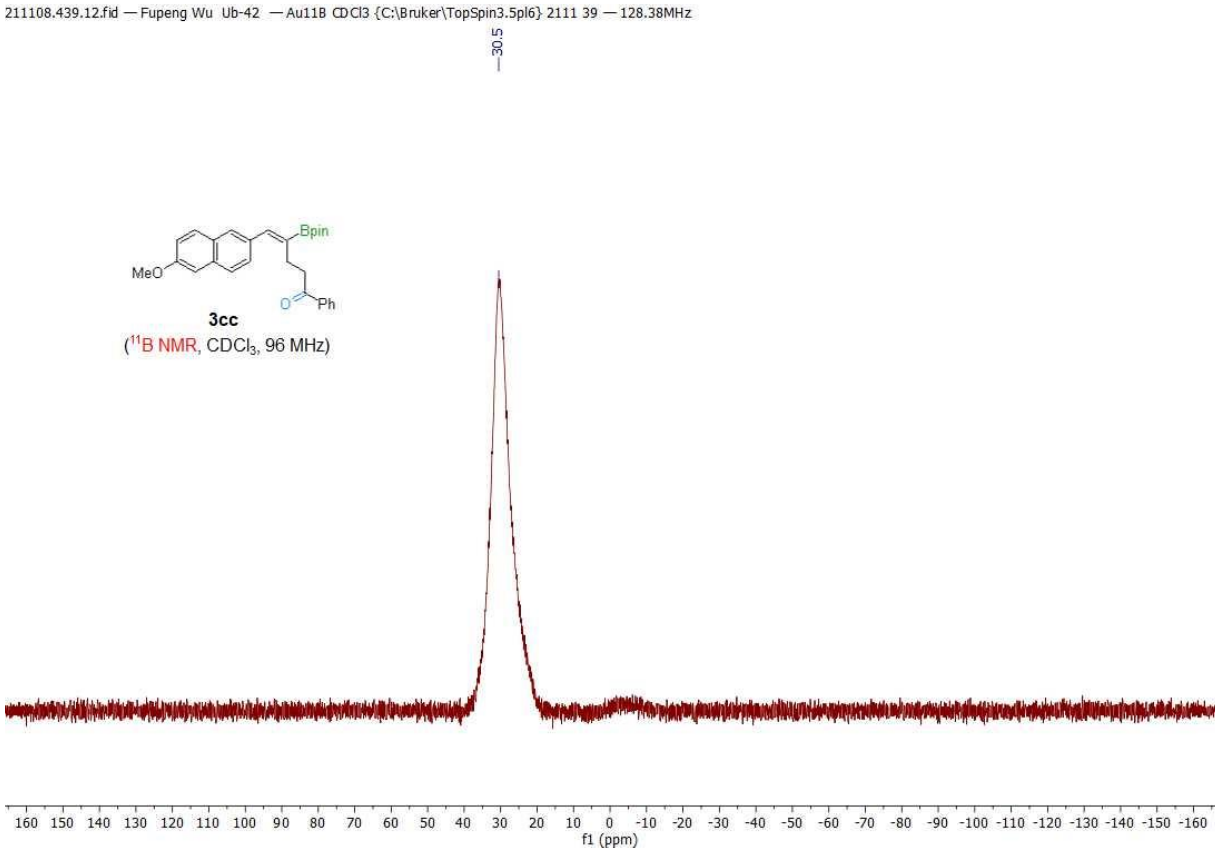
211108.439.12.fid — Fupeng Wu Ub-42 — Au11B CDCl3 {C:\Bruker\TopSpin3.5pl6} 2111 39 — 128.38MHz
30.5
Bpin
MeO
O
Ph
3cc
(11B NMR, CDCl3, 96 MHz)
160 150 140 130 120 110 100 90 80 70 60 50 40 30 20 10 0 -10 -20 -30 -40 -50 -60 -70 -80 -90 -100 -110 -120 -130 -140 -150 -160
f1 (ppm)

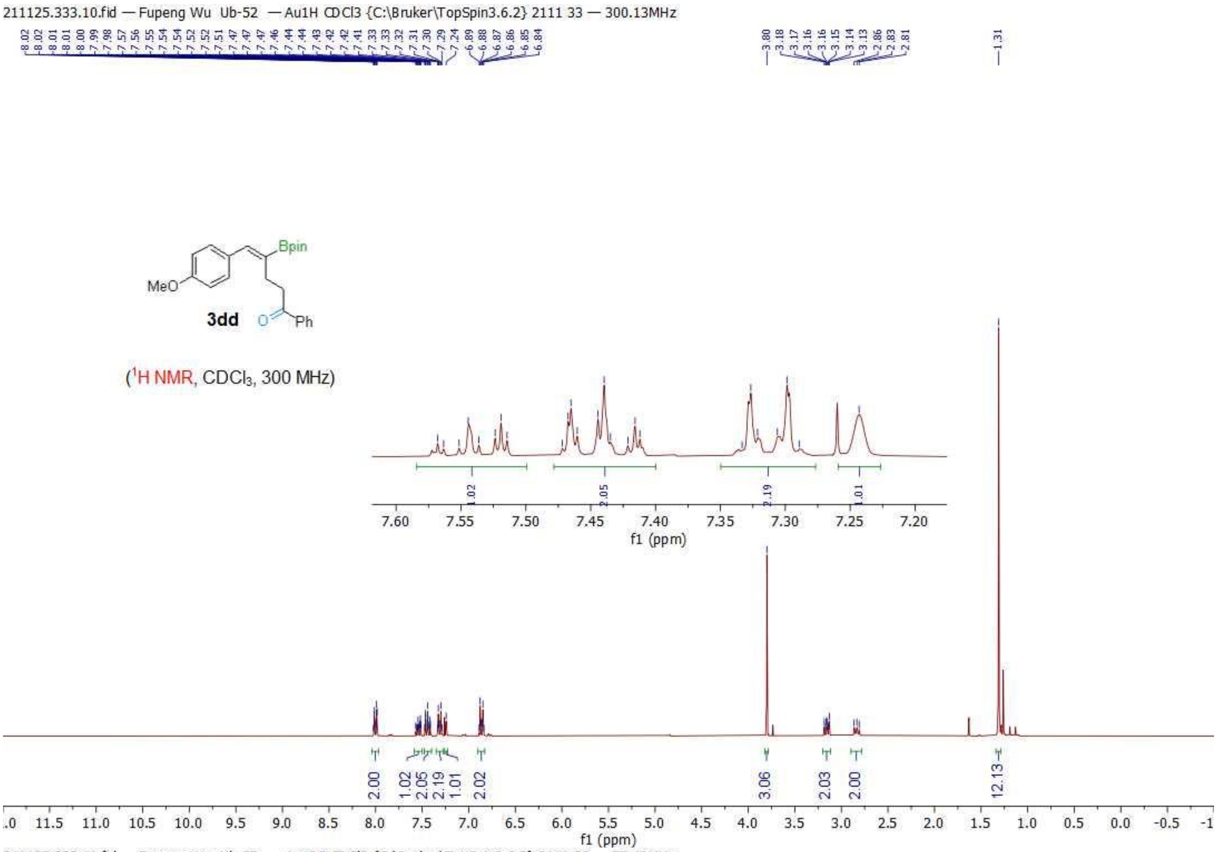
211125.333.10.fid — Fupeng Wu Ub-52 — Au1H CDCl3 {C:\Bruker\TopSpin3.6.2} 2111 33 — 300.13MHz
3dd
(1H NMR, CDCl3, 300 MHz)
f1 (ppm)

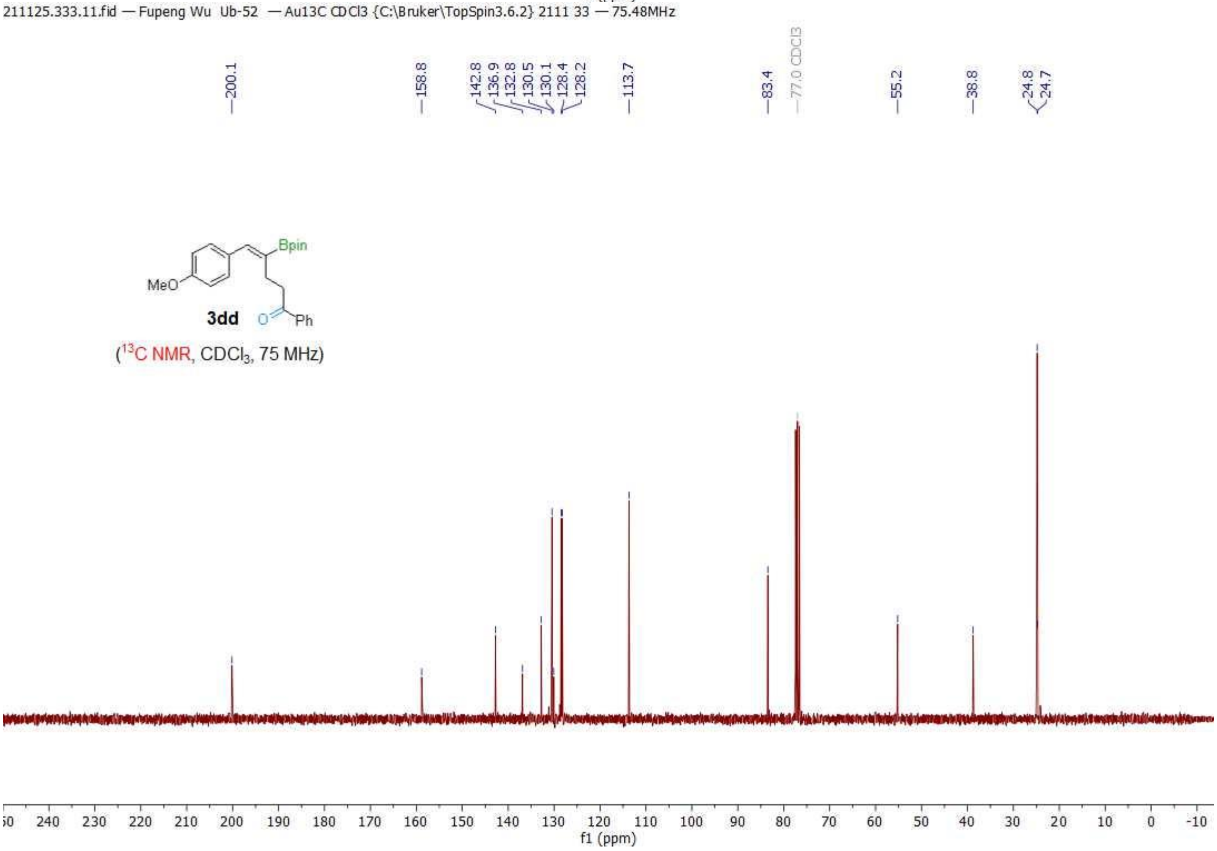
211125.333.11.fid — Fupeng Wu Ub-52 — Au13C CDCl3 {C:\Bruker\TopSpin3.6.2} 2111 33 — 75.48MHz
200.1
158.8
142.8
136.9
132.8
130.5
130.1
128.4
128.2
113.7
83.4
77.0 CDCl3
55.2
38.8
24.8
24.7
3dd
(13C NMR, CDCl3, 75 MHz)
f1 (ppm)

211125.333.12.fid — Fupeng Wu Ub-52 — Au11B CDCl3 {C:\Bruker\TopSpin3.6.2} 2111 33 — 96.29MHz

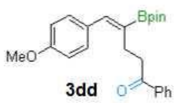

(11B NMR, $CDCl_3$, 96 MHz)

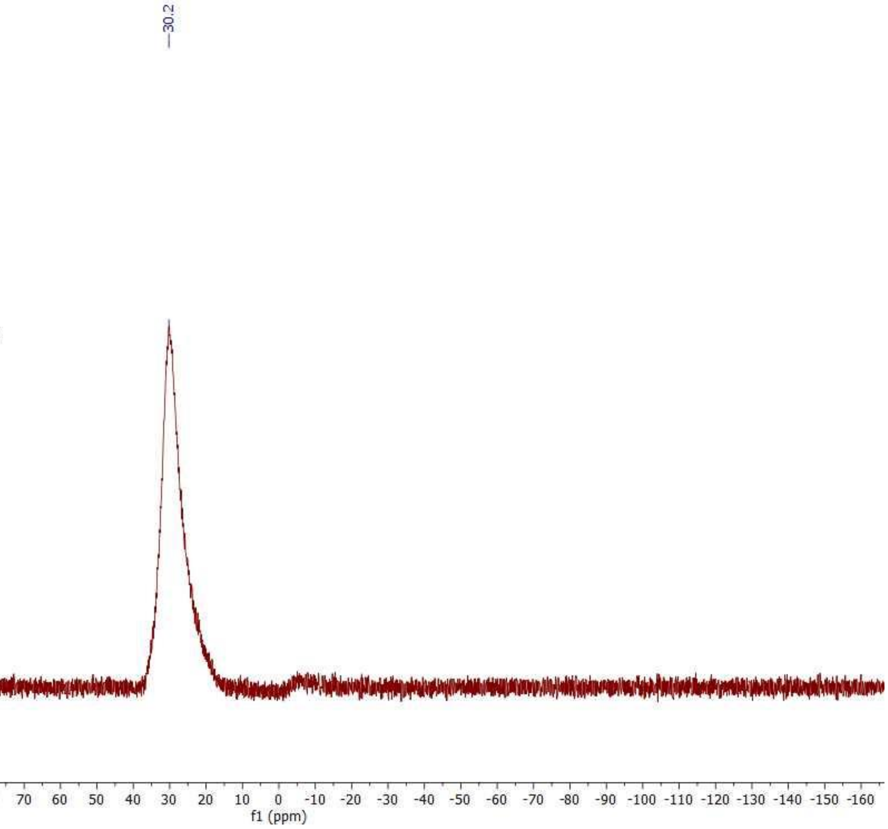

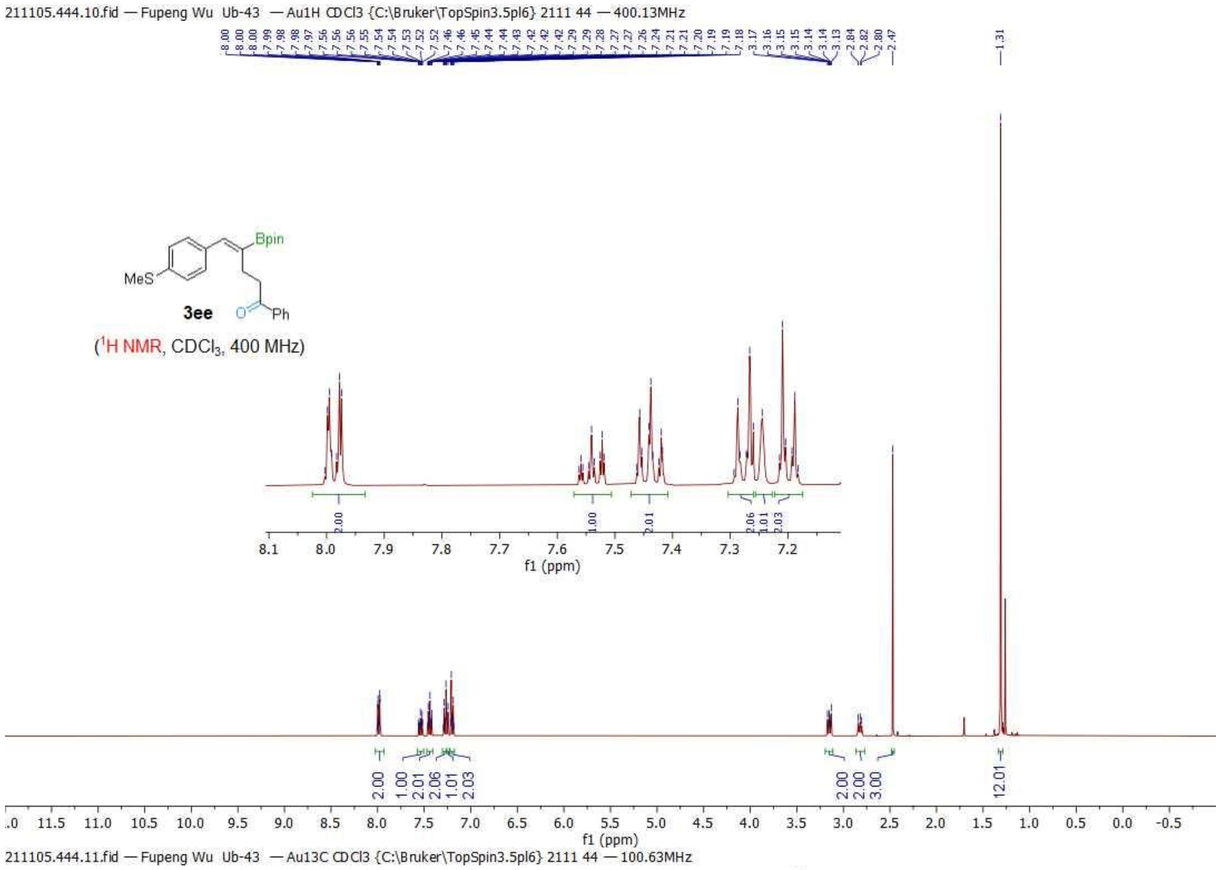
211105.444.10.fid — Fupeng Wu Ub-43 — Au1H CDCl3 {C:\Bruker\TopSpin3.5pl6} 2111 44 — 400.13MHz
Bpin
MeS
3ee
Ph
(1H NMR, CDCl3, 400 MHz)
f1 (ppm)

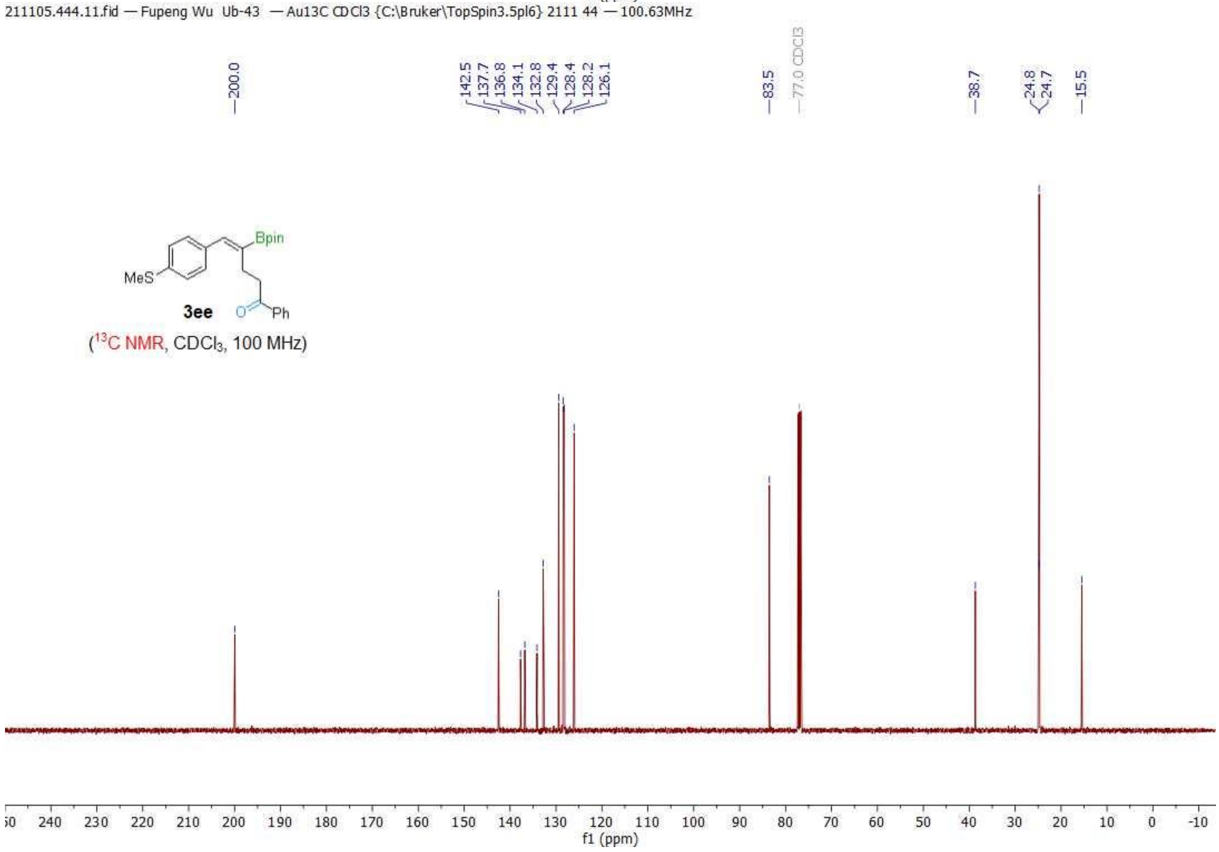
211105.444.11.fid — Fupeng Wu Ub-43 — Au13C CDCl3 {C:\Bruker\TopSpin3.5pl6} 2111 44 — 100.63MHz
200.0
142.5
137.7
136.8
134.1
132.8
129.4
128.4
128.2
126.1
83.5
77.0 CDCl3
38.7
24.8
24.7
15.5
Bpin
MeS
3ee
Ph
(13C NMR, CDCl3, 100 MHz)
f1 (ppm)

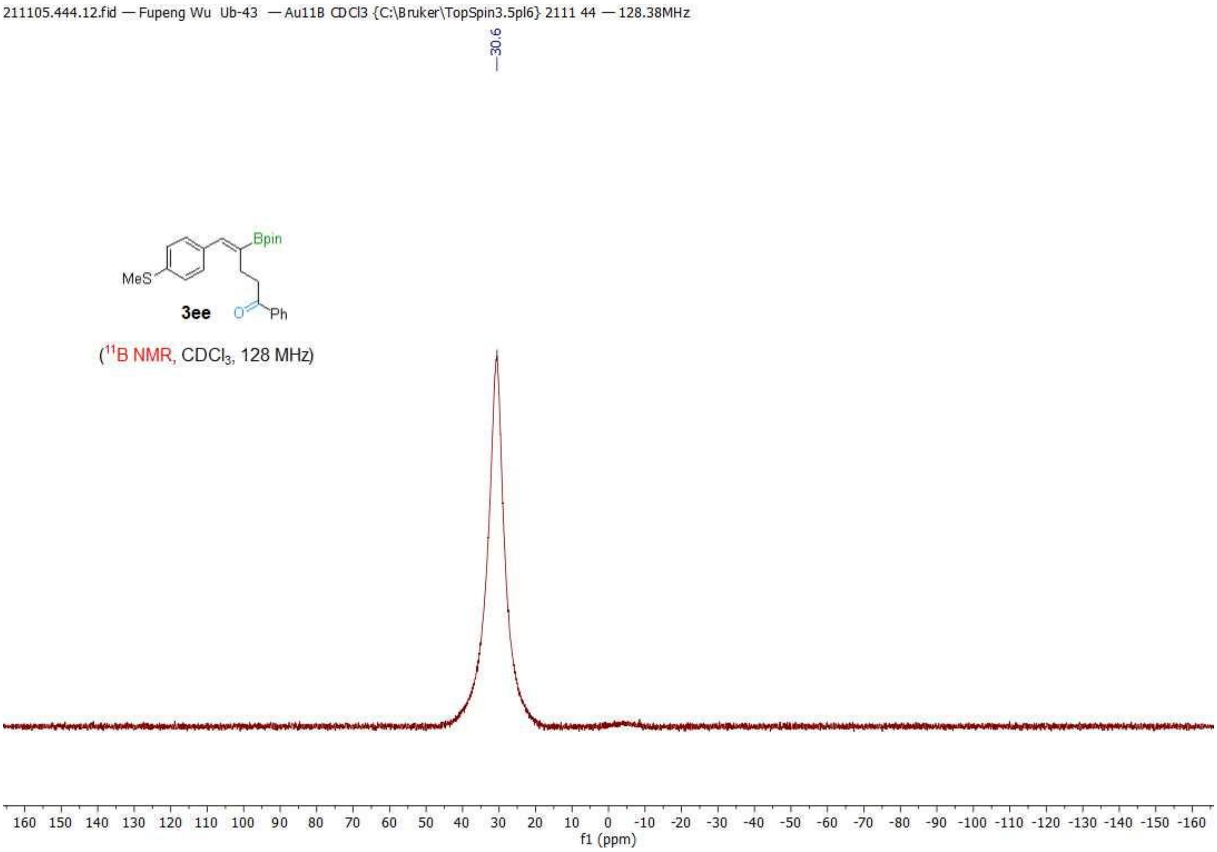
211105.444.12.fid — Fupeng Wu Ub-43 — Au11B CDCl3 {C:\Bruker\TopSpin3.5pl6} 2111 44 — 128.38MHz
30.6
Bpin
MeS
3ee
O
Ph
(11B NMR, CDCl3, 128 MHz)
160 150 140 130 120 110 100 90 80 70 60 50 40 30 20 10 0 -10 -20 -30 -40 -50 -60 -70 -80 -90 -100 -110 -120 -130 -140 -150 -160
f1 (ppm)

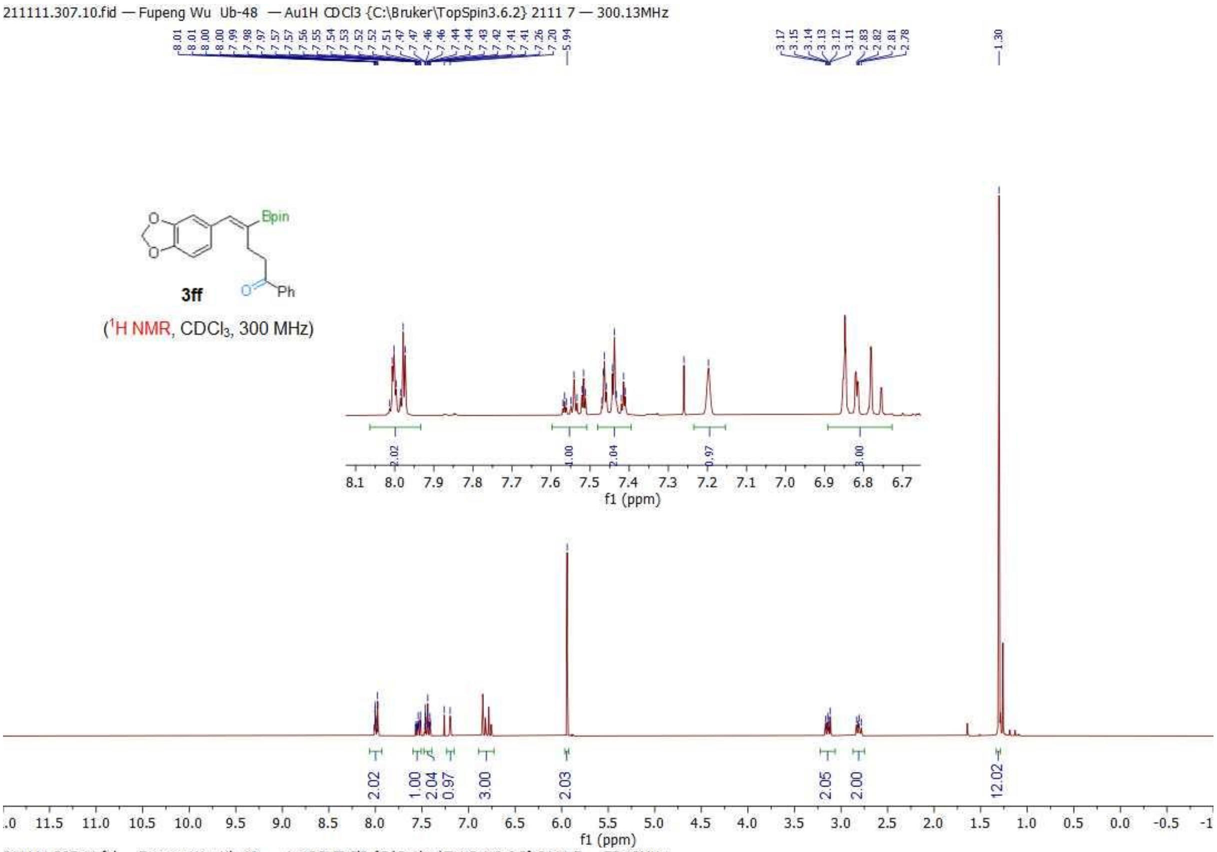
211111.307.10.fid — Fupeng Wu Ub-48 — Au1H CDCl3 {C:\Bruker\TopSpin3.6.2} 2111 7 — 300.13MHz
Bpin
3ff
O
Ph
(1H NMR, CDCl3, 300 MHz)
f1 (ppm)

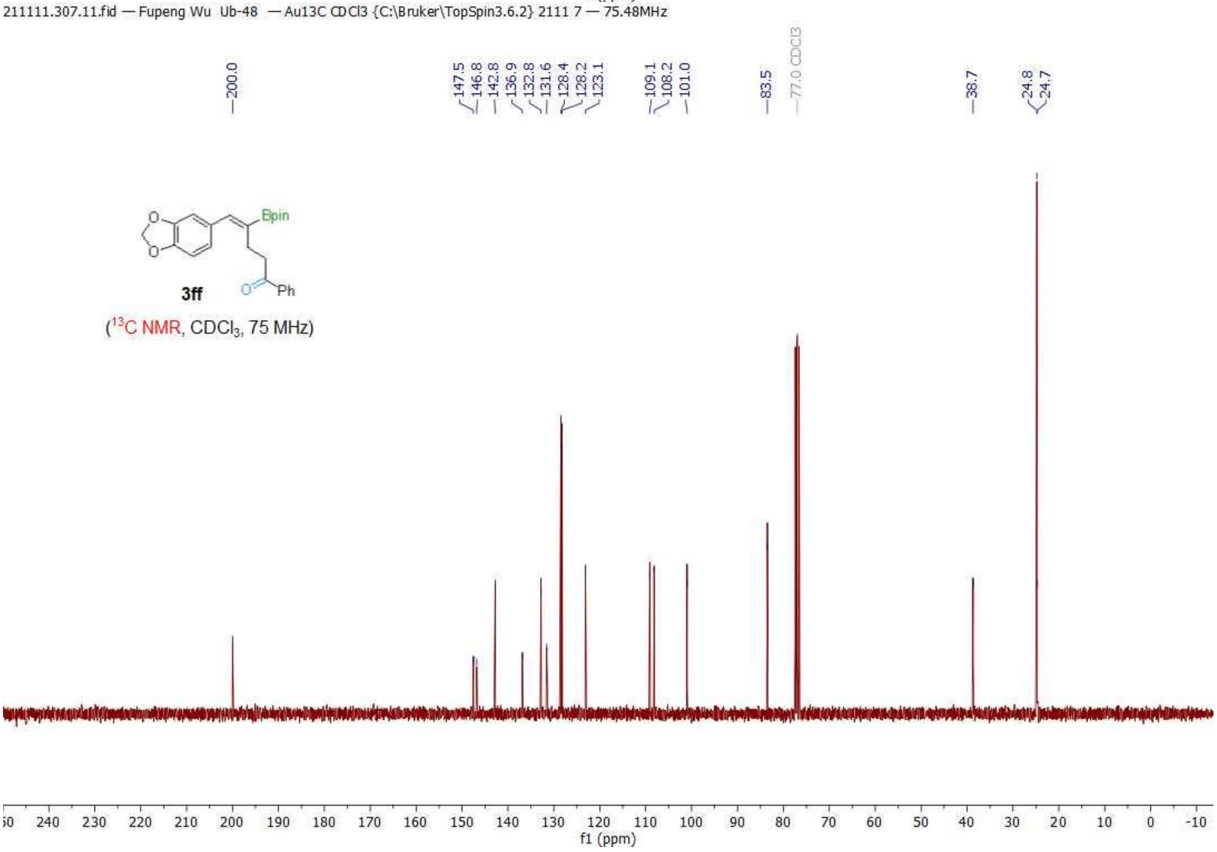
211111.307.11.fid — Fupeng Wu Ub-48 — Au13C CDCl3 {C:\Bruker\TopSpin3.6.2} 2111 7 — 75.48MHz
200.0
147.5
146.8
142.8
136.9
132.8
131.6
128.4
128.2
123.1
109.1
108.2
101.0
83.5
77.0 CDCl3
38.7
24.8
24.7
Bpin
3ff
O
Ph
(13C NMR, CDCl3, 75 MHz)
f1 (ppm)

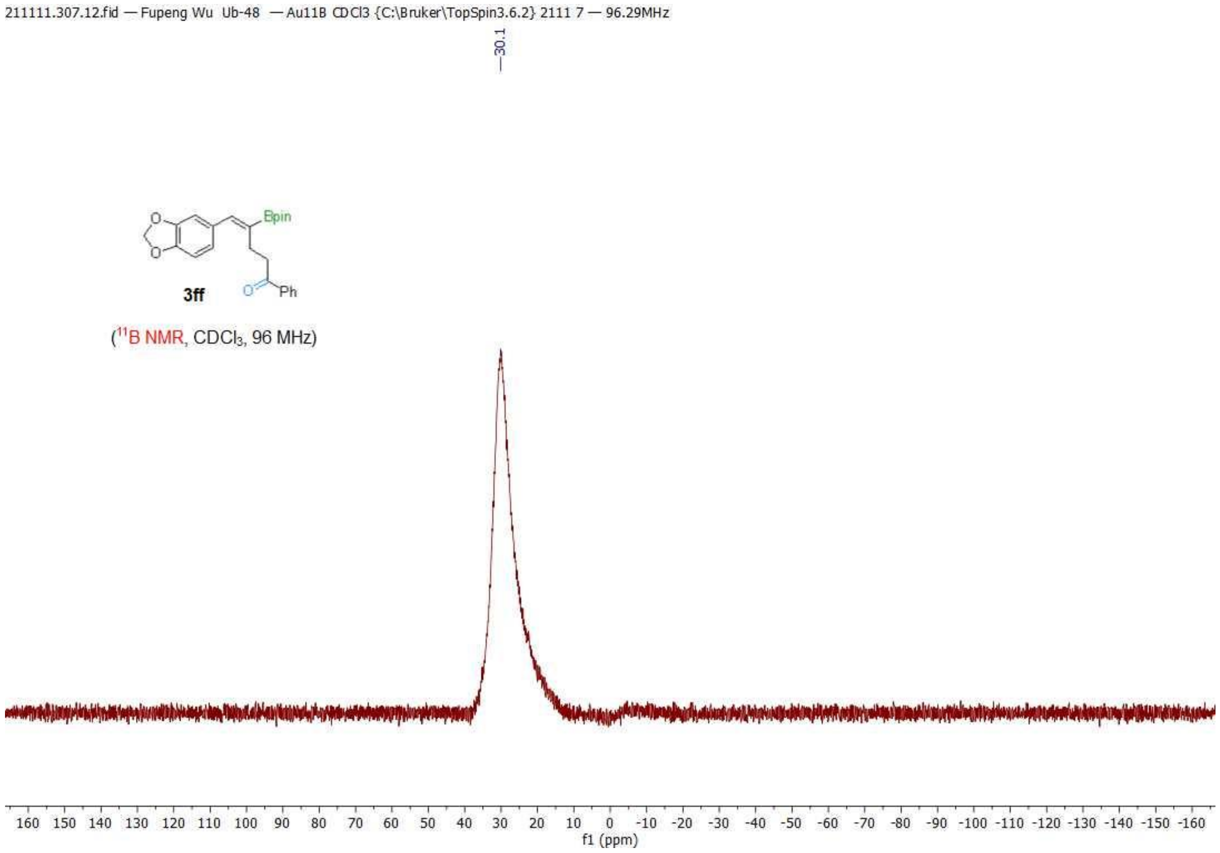
211111.307.12.fid — Fupeng Wu Ub-48 — Au11B CDCl3 {C:\Bruker\TopSpin3.6.2} 2111 7 — 96.29MHz
—30.1
Bpin
3ff
O
Ph
(11B NMR, CDCl3, 96 MHz)
160 150 140 130 120 110 100 90 80 70 60 50 40 30 20 10 0 -10 -20 -30 -40 -50 -60 -70 -80 -90 -100 -110 -120 -130 -140 -150 -160
f1 (ppm)

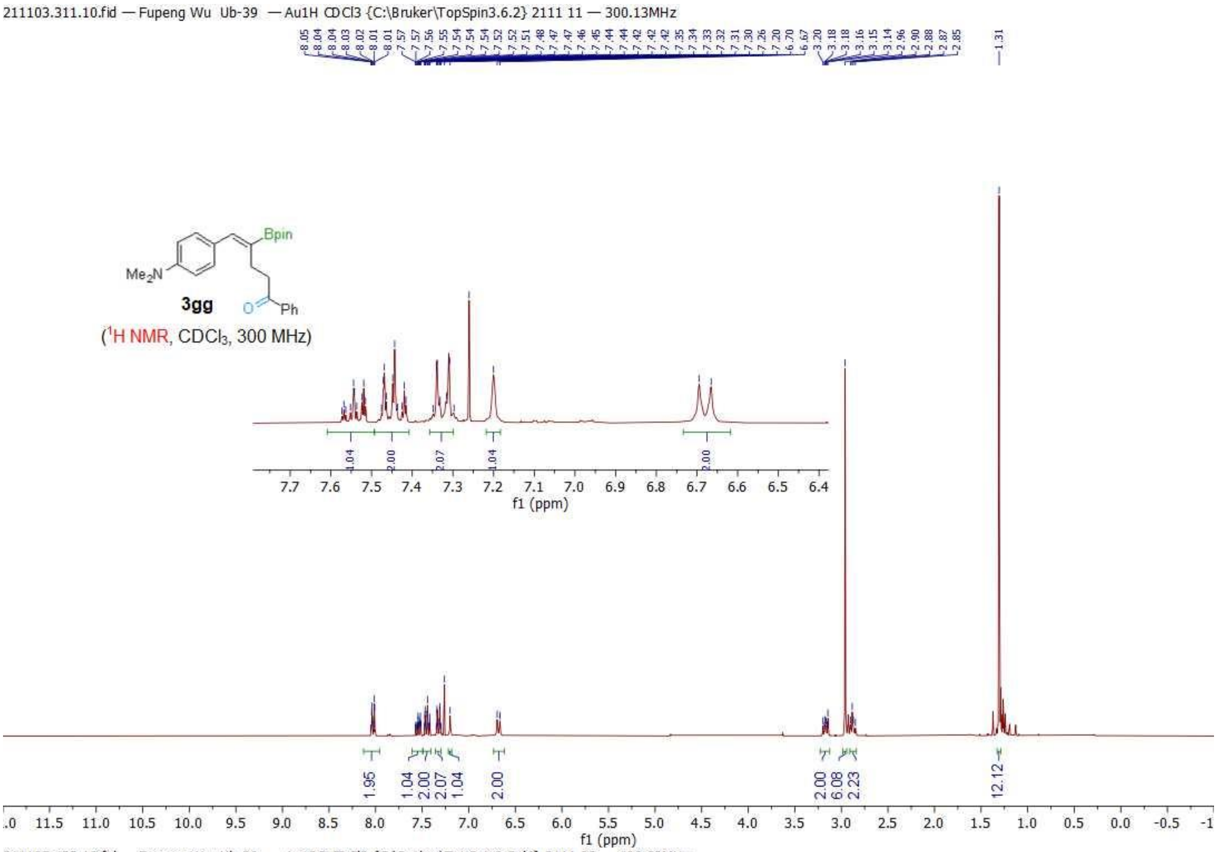
211103.311.10.fid — Fupeng Wu Ub-39 — Au1H CDCl3 {C:\Bruker\TopSpin3.6.2} 2111 11 — 300.13MHz
Bpin
Me2N
3gg
O
Ph
(1H NMR, CDCl3, 300 MHz)
1.31
1.95
1.04
2.00
2.07
1.04
2.00
2.00
6.08
2.23
12.12
f1 (ppm)

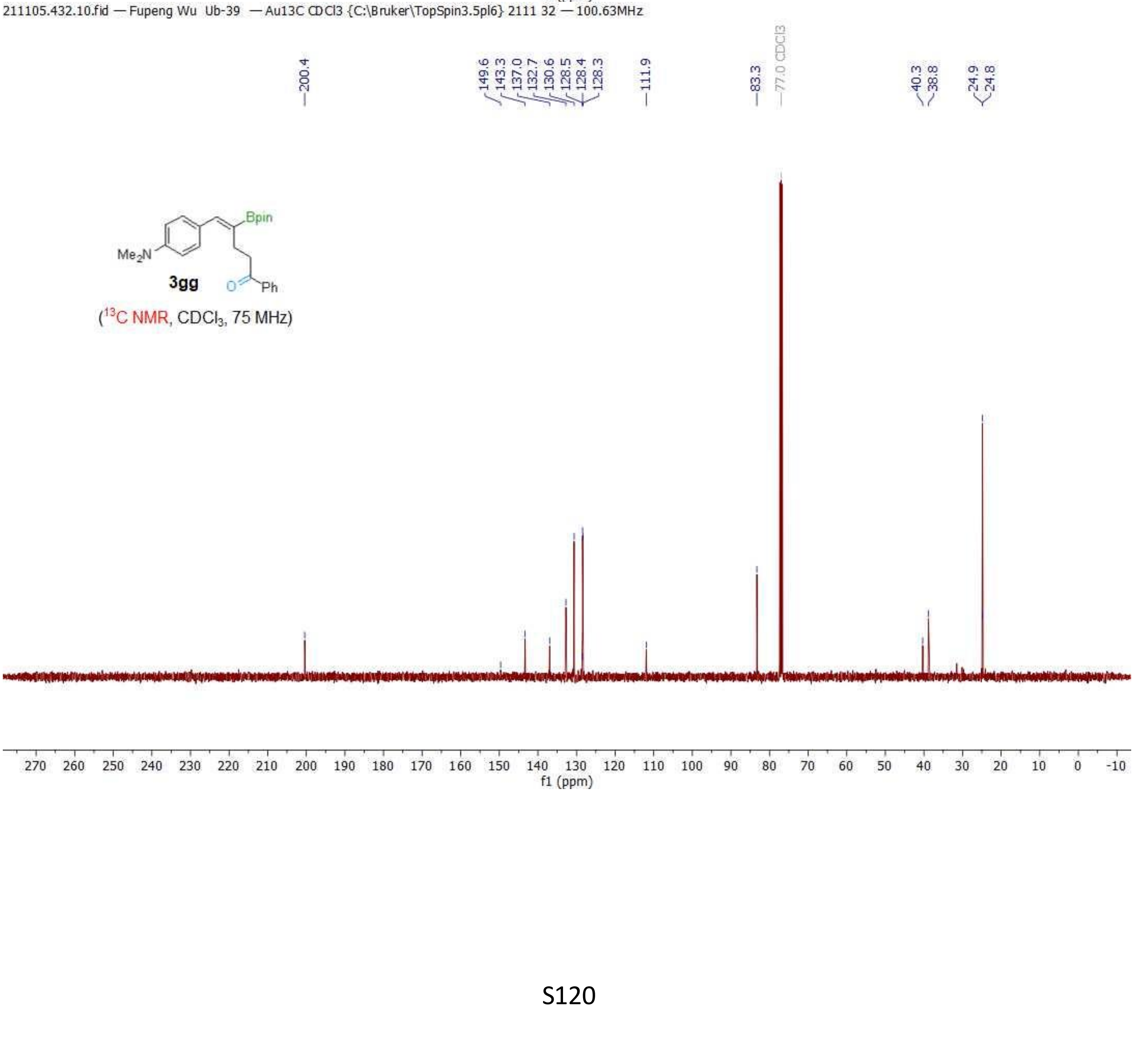
211105.432.10.fid — Fupeng Wu Ub-39 — Au13C CDCl3 {C:\Bruker\TopSpin3.5pl6} 2111 32 — 100.63MHz
200.4
149.6
143.3
137.0
132.7
130.6
128.5
128.4
128.3
111.9
83.3
77.0 CDCl3
40.3
38.8
24.9
24.8
Bpin
Me2N
3gg
O
Ph
(13C NMR, CDCl3, 75 MHz)
f1 (ppm)

211105.432.11.fid — Fupeng Wu Ub-39 — Au11B CDCl3 {C:\Bruker\TopSpin3.5pl6} 2111 32 — 128.38MHz

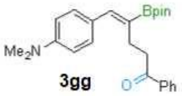

3gg

(^{11}B NMR, $CDCl_3$, 96 MHz)

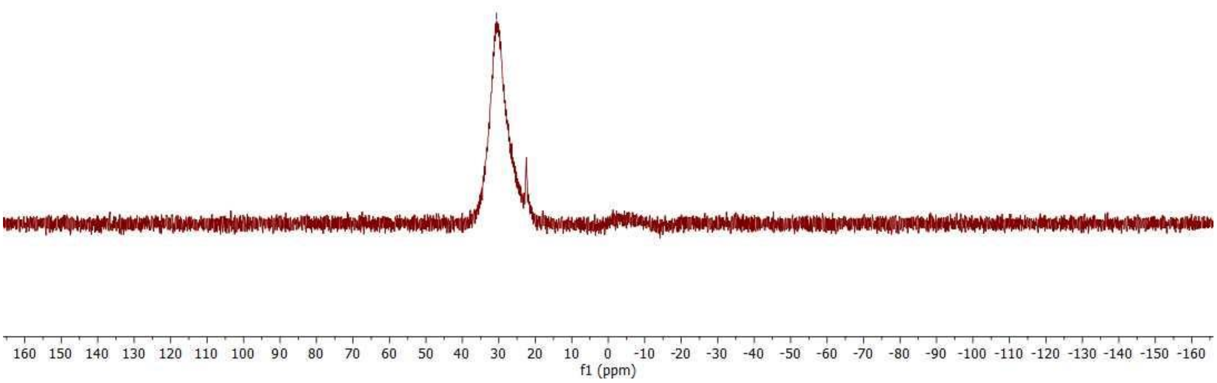

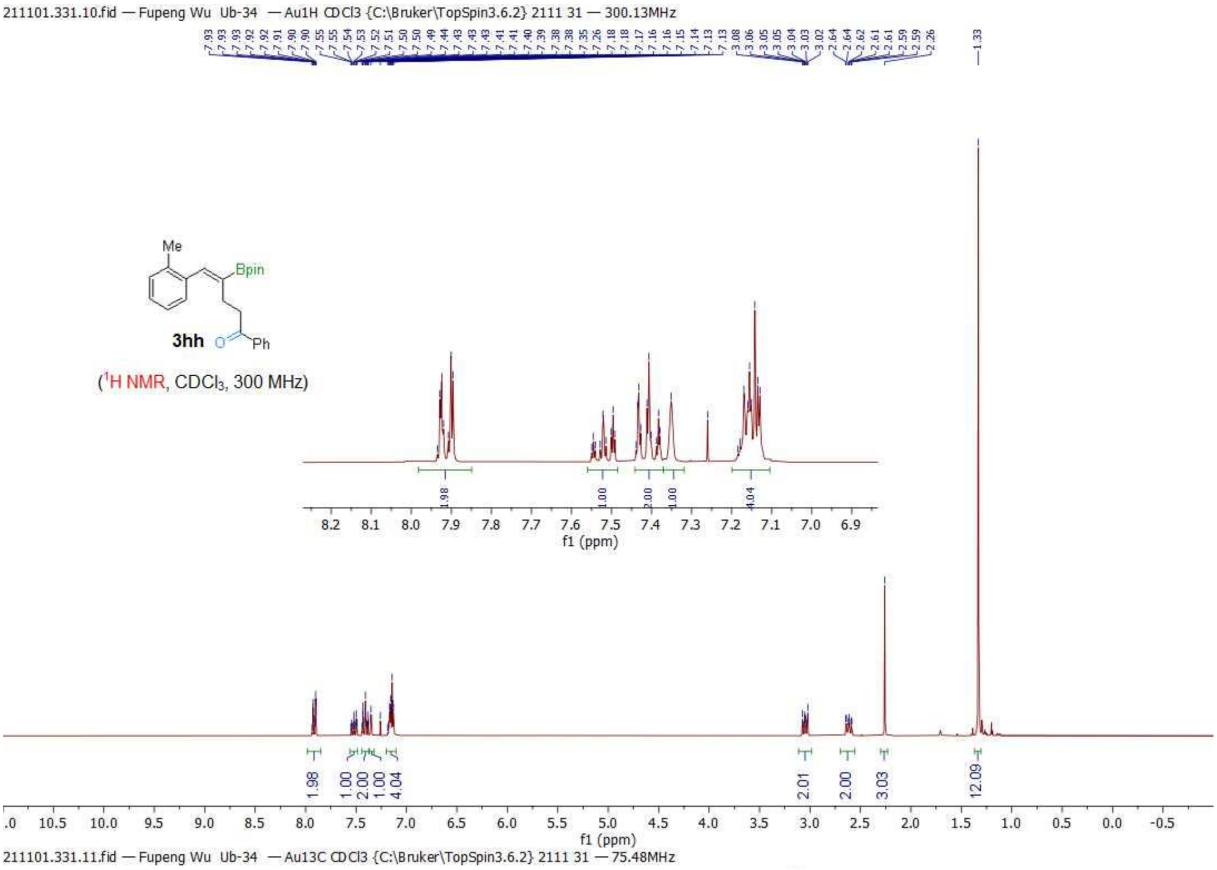
211101.331.10.fid — Fupeng Wu Ub-34 — Au1H CDCl3 {C:\Bruker\TopSpin3.6.2} 2111 31 — 300.13MHz
1.33
Me
Bpin
3hh
O
Ph
(1H NMR, CDCl3, 300 MHz)
1.98
1.00
2.00
1.00
4.04
2.01
2.00
3.03
12.09
f1 (ppm)

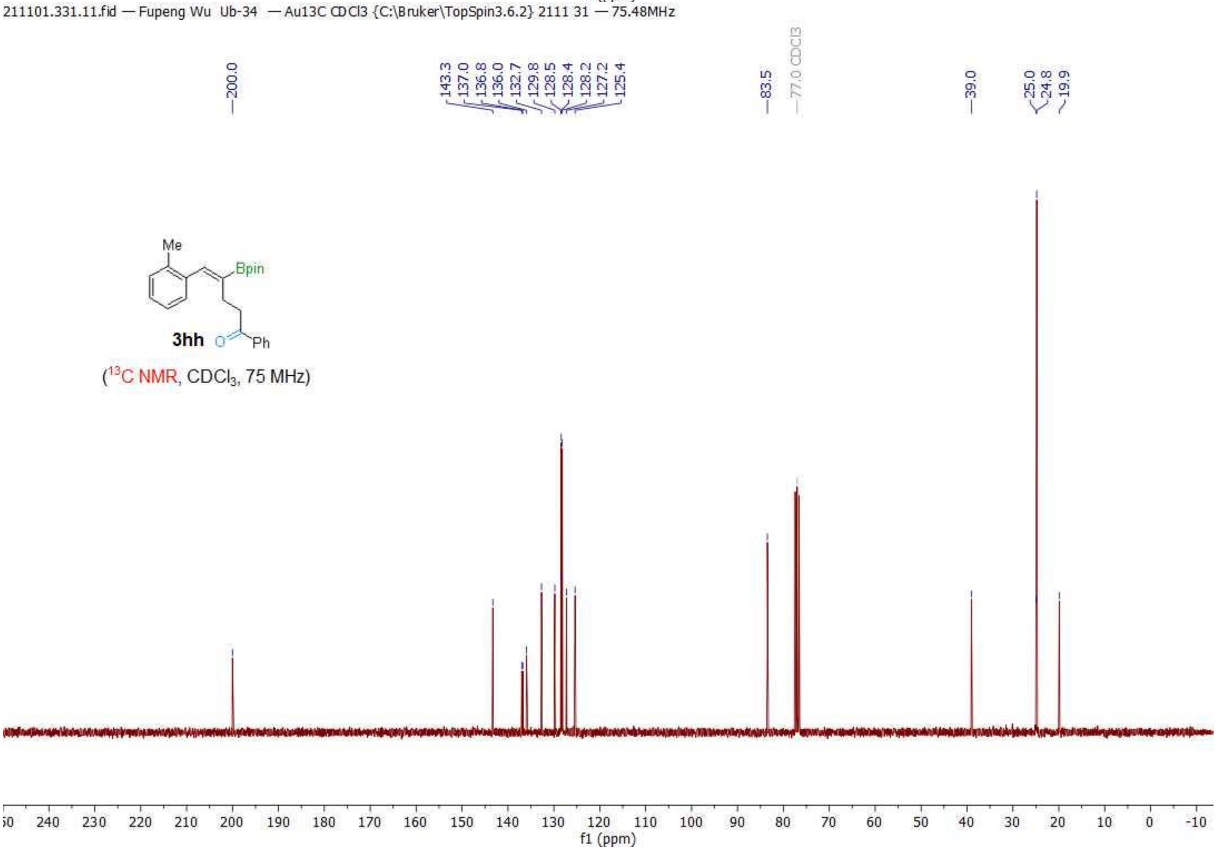
211101.331.11.fid — Fupeng Wu Ub-34 — Au13C CDCl3 {C:\Bruker\TopSpin3.6.2} 2111 31 — 75.48MHz
200.0
143.3
137.0
136.8
136.0
132.7
129.8
128.5
128.4
128.2
127.2
125.4
83.5
77.0 CDCl3
39.0
25.0
24.8
19.9
Me
Bpin
3hh
O
Ph
(13C NMR, CDCl3, 75 MHz)
f1 (ppm)

211101.331.12.fid — Fupeng Wu Ub-34 — Au11B CDCl3 {C:\Bruker\TopSpin3.6.2} 2111 31 — 96.29MHz

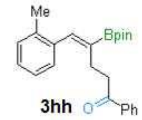

3hh

(^{11}B NMR, $CDCl_3$, 96 MHz)

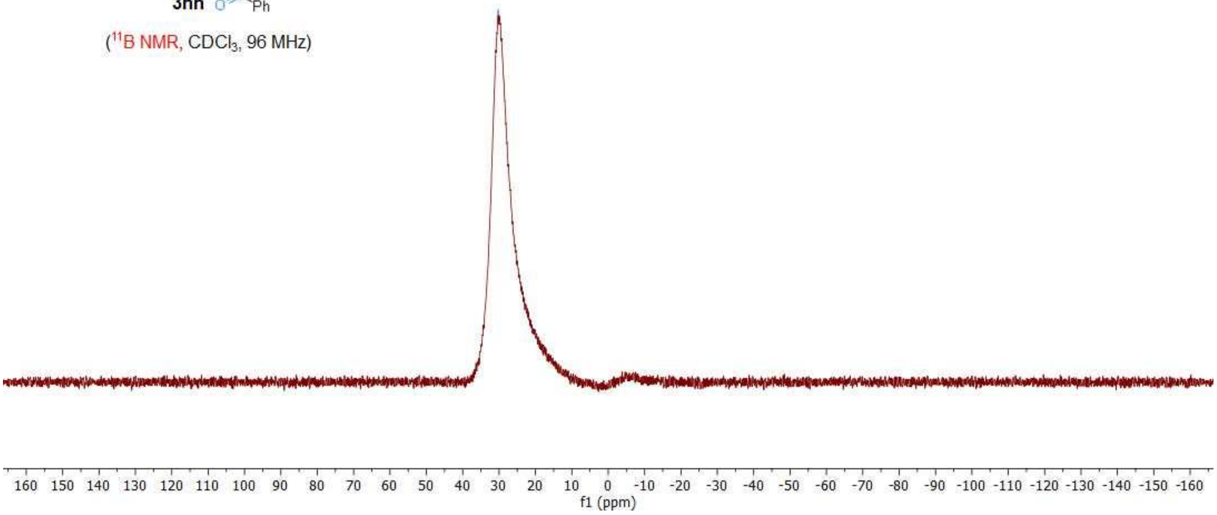

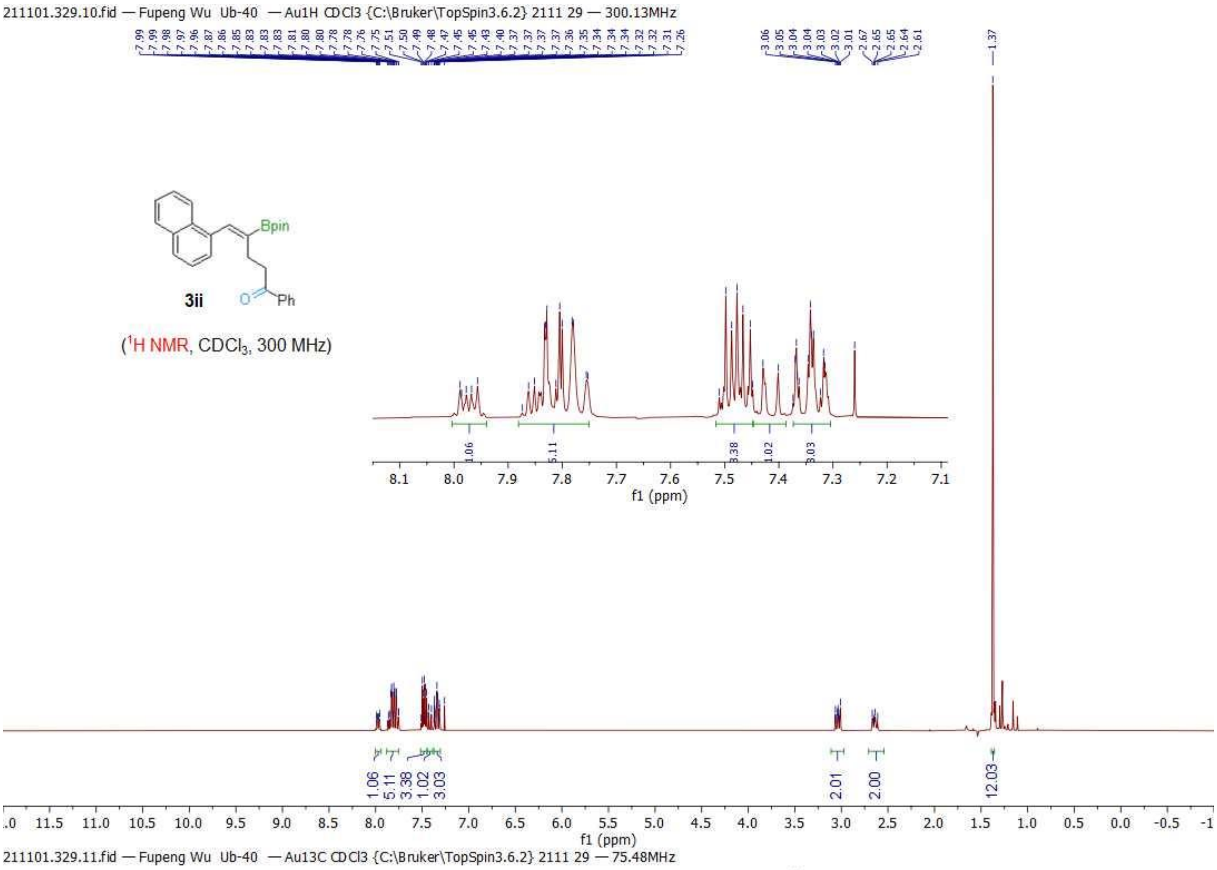
211101.329.10.fid — Fupeng Wu Ub-40 — Au1H CDCl3 {C:\Bruker\TopSpin3.6.2} 2111 29 — 300.13MHz
7.99 7.98 7.97 7.96 7.87 7.86 7.85 7.83 7.83 7.81 7.80 7.80 7.78 7.76 7.75 7.51 7.50 7.49 7.48 7.47 7.45 7.45 7.43 7.40 7.37 7.37 7.37 7.37 7.36 7.35 7.34 7.34 7.34 7.32 7.32 7.31 7.26
3.06 3.05 3.04 3.04 3.03 3.02 3.01 2.67 2.65 2.65 2.64 2.61
1.37
Bpin
3ii
O
Ph
(1H NMR, CDCl3, 300 MHz)
1.06
5.11
3.38
1.02
3.03
f1 (ppm)
2.01
2.00
12.03
f1 (ppm)

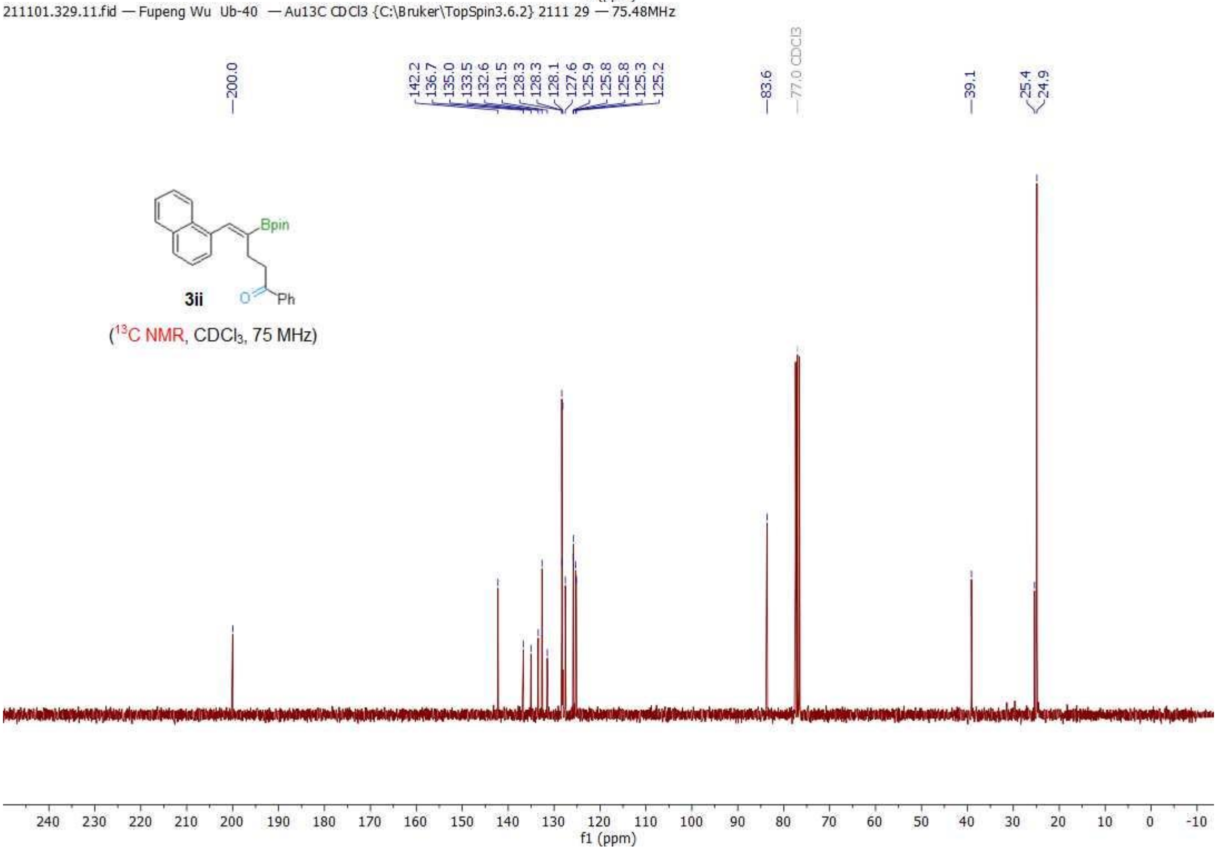
211101.329.11.fid — Fupeng Wu Ub-40 — Au13C CDCl3 {C:\Bruker\TopSpin3.6.2} 2111 29 — 75.48MHz
200.0
142.2 136.7 135.0 133.5 132.6 131.5 128.3 128.3 128.1 127.6 125.9 125.8 125.8 125.3 125.2
83.6
77.0 CDCl3
39.1
25.4
24.9
Bpin
3ii
O
Ph
(13C NMR, CDCl3, 75 MHz)
f1 (ppm)

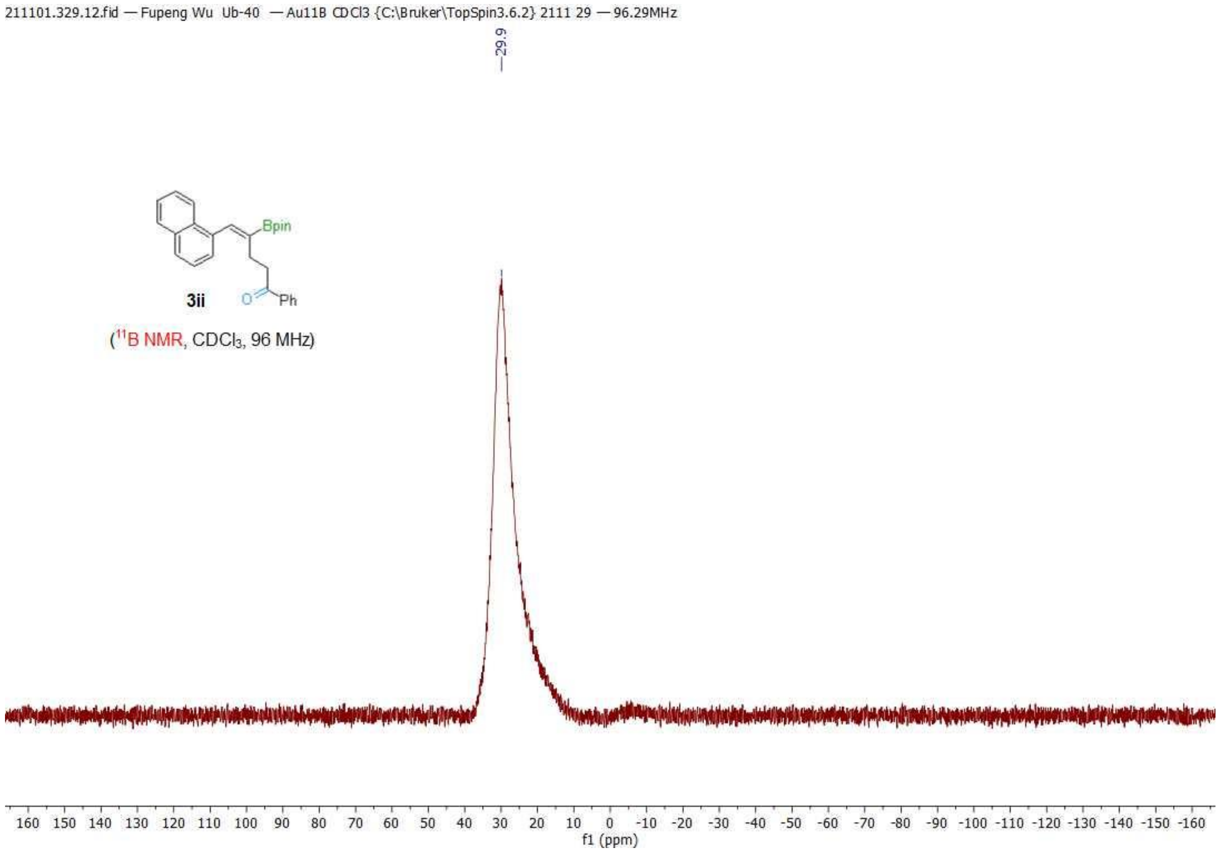
211101.329.12.fid — Fupeng Wu Ub-40 — Au11B CDCl3 {C:\Bruker\TopSpin3.6.2} 2111 29 — 96.29MHz
29.9
Bpin
O
Ph
3ii
(11B NMR, CDCl3, 96 MHz)
160 150 140 130 120 110 100 90 80 70 60 50 40 30 20 10 0 -10 -20 -30 -40 -50 -60 -70 -80 -90 -100 -110 -120 -130 -140 -150 -160
f1 (ppm)

211111.306.10.fid — Fupeng Wu Ub-47 — Au1H CDCl3 {C:\Bruker\TopSpin3.6.2} 2111 6 — 300.13MHz

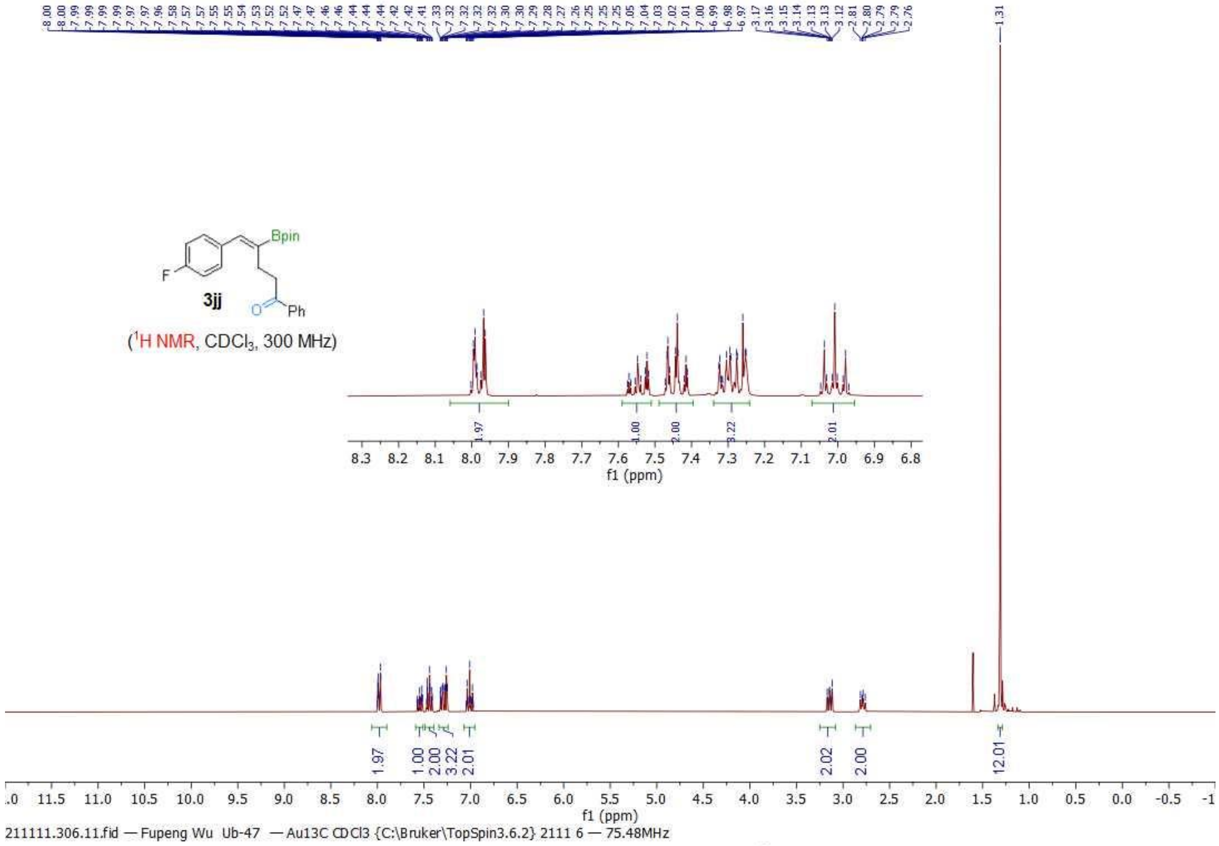

211111.306.11.fid — Fupeng Wu Ub-47 — Au13C CDCl3 {C:\Bruker\TopSpin3.6.2} 2111 6 — 75.48MHz

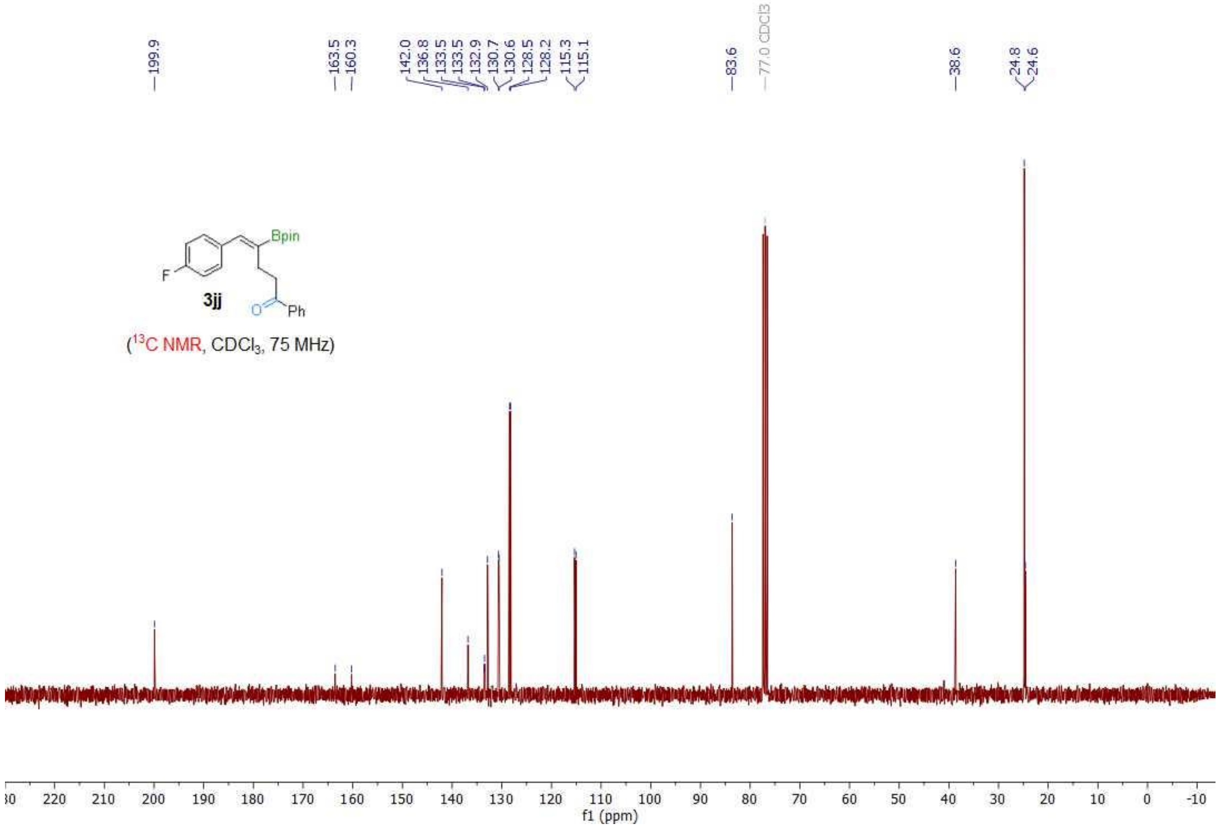

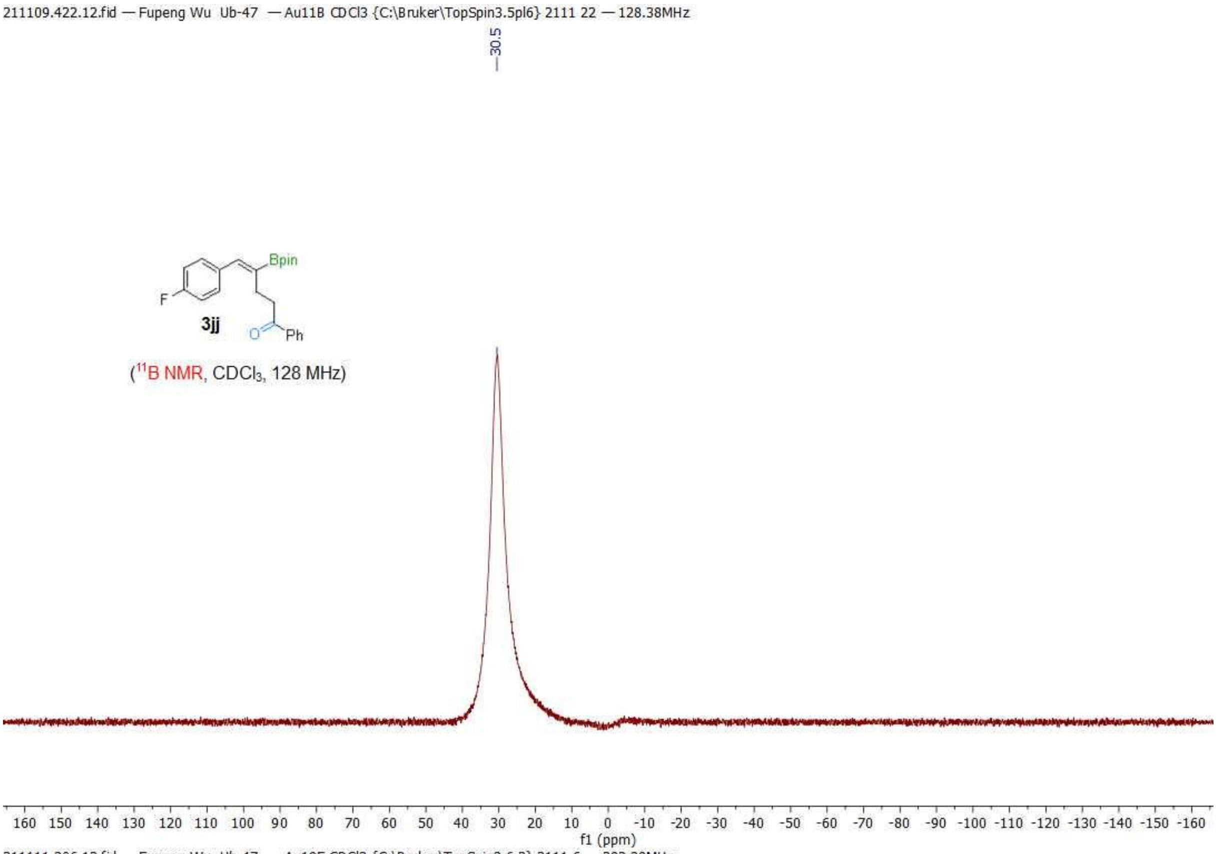
211109.422.12.fid — Fupeng Wu Ub-47 — Au11B CDCl3 {C:\Bruker\TopSpin3.5pl6} 2111 22 — 128.38MHz
30.5
Bpin
F
3jj
O
Ph
(11B NMR, CDCl3, 128 MHz)
160 150 140 130 120 110 100 90 80 70 60 50 40 30 20 10 0 -10 -20 -30 -40 -50 -60 -70 -80 -90 -100 -110 -120 -130 -140 -150 -160
f1 (ppm)

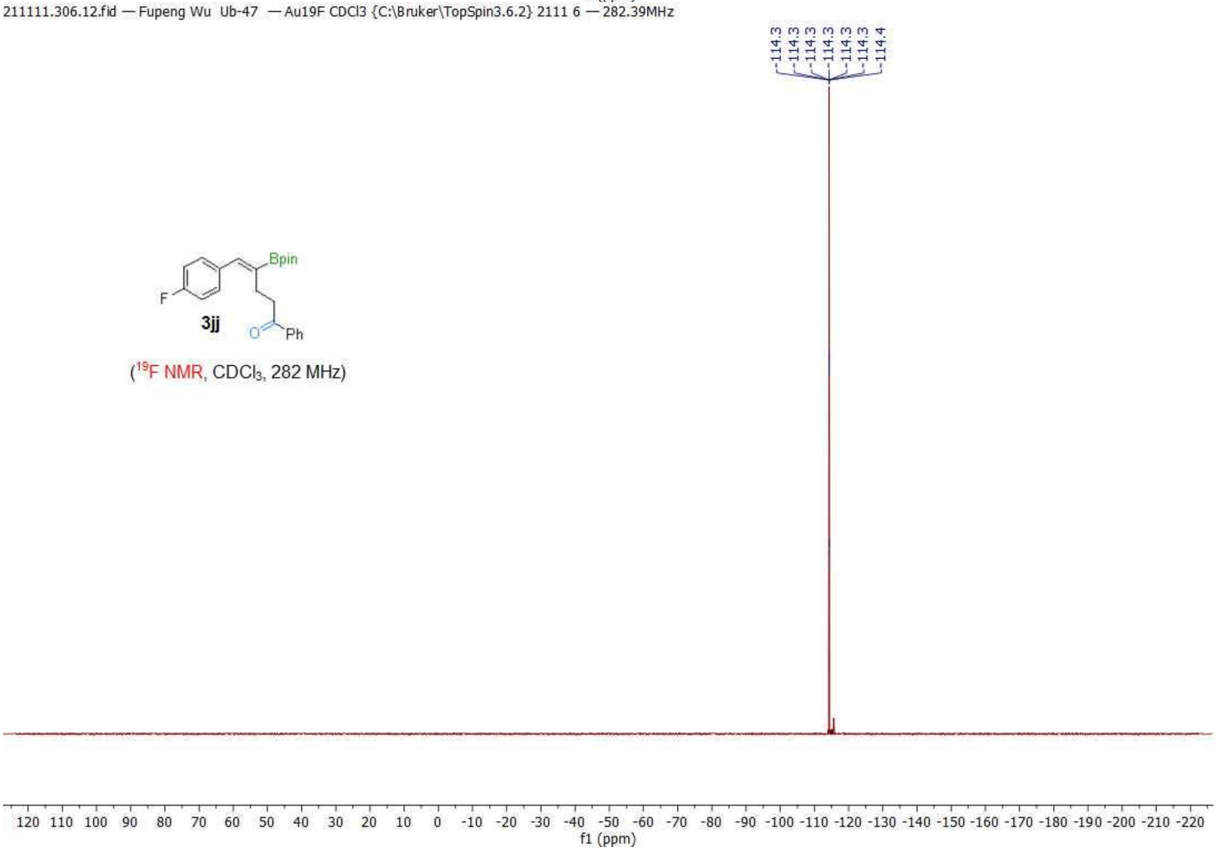
211111.306.12.fid — Fupeng Wu Ub-47 — Au19F CDCl3 {C:\Bruker\TopSpin3.6.2} 2111 6 — 282.39MHz
-114.3
-114.3
-114.3
-114.3
-114.3
-114.3
-114.4
Bpin
F
3jj
O
Ph
(19F NMR, CDCl3, 282 MHz)
120 110 100 90 80 70 60 50 40 30 20 10 0 -10 -20 -30 -40 -50 -60 -70 -80 -90 -100 -110 -120 -130 -140 -150 -160 -170 -180 -190 -200 -210 -220
f1 (ppm)

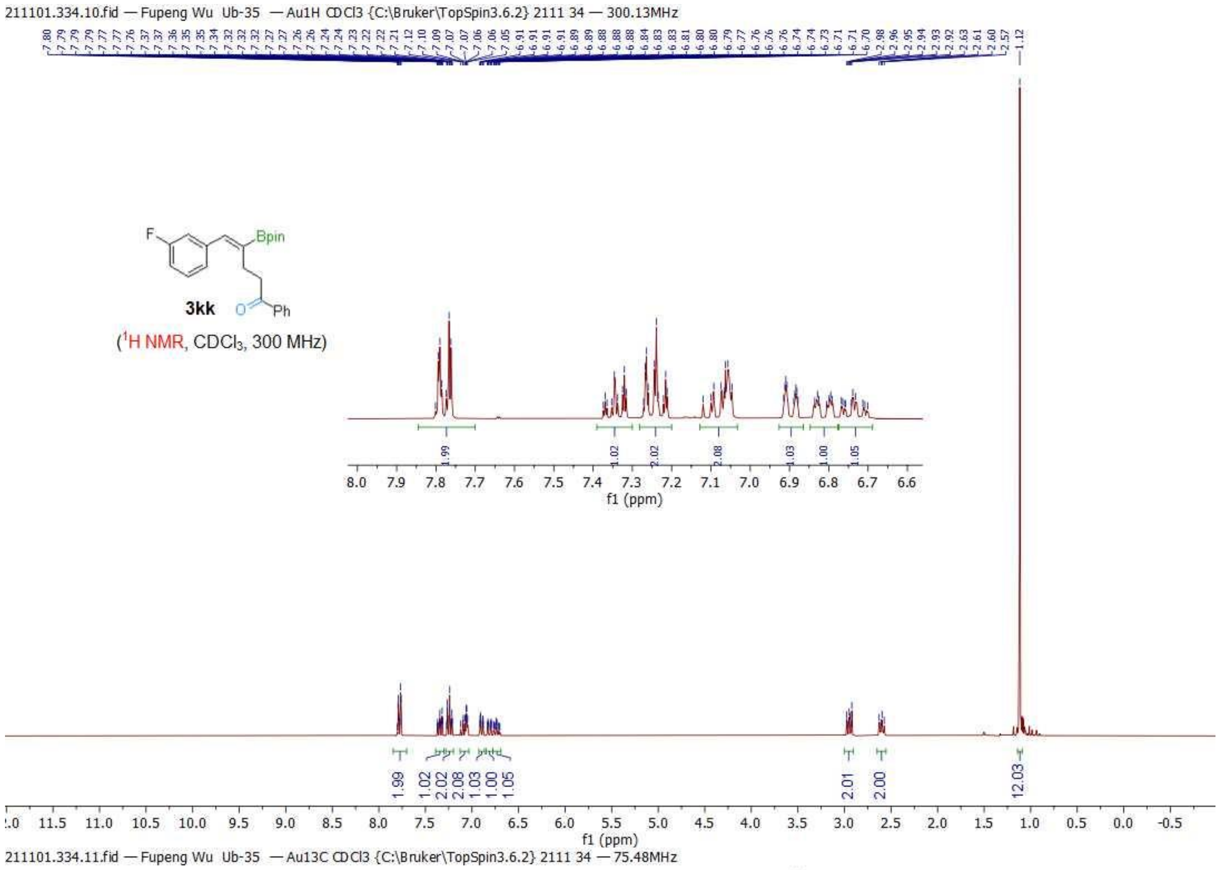

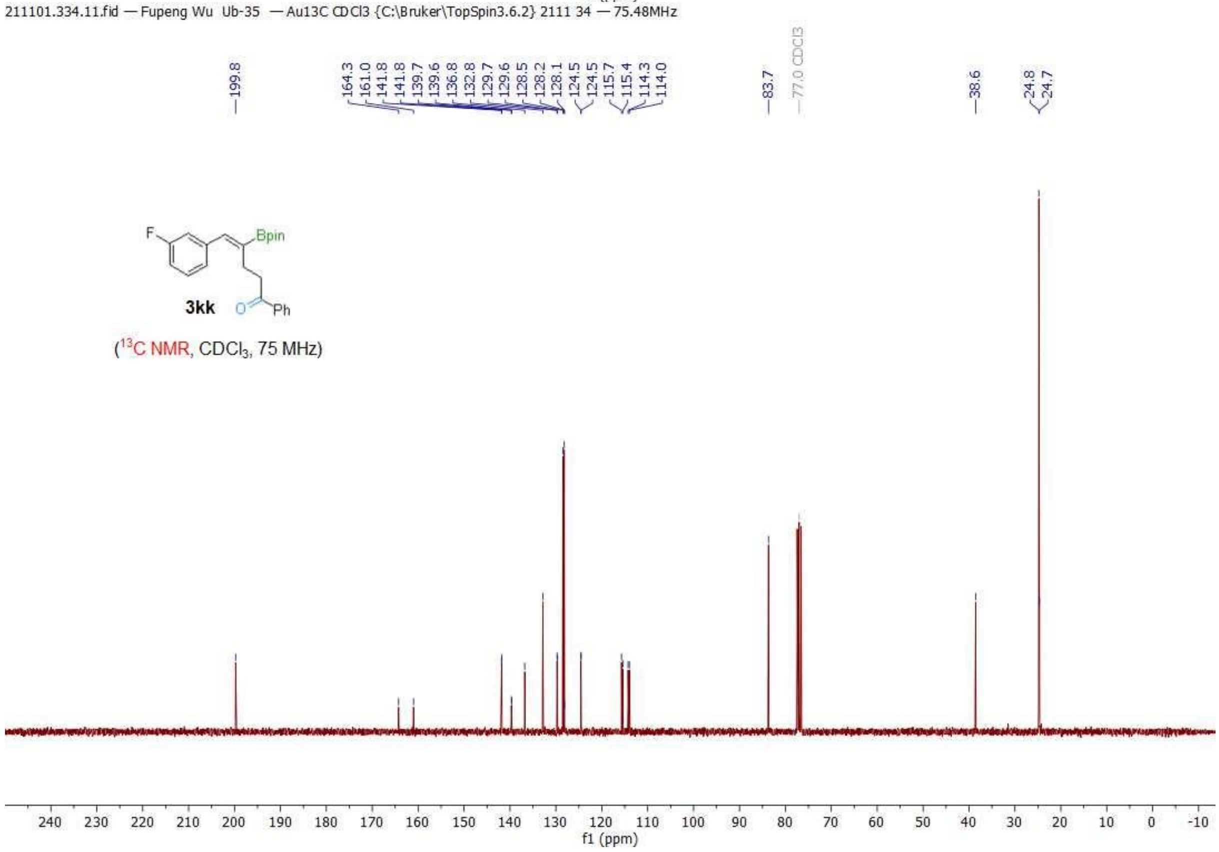

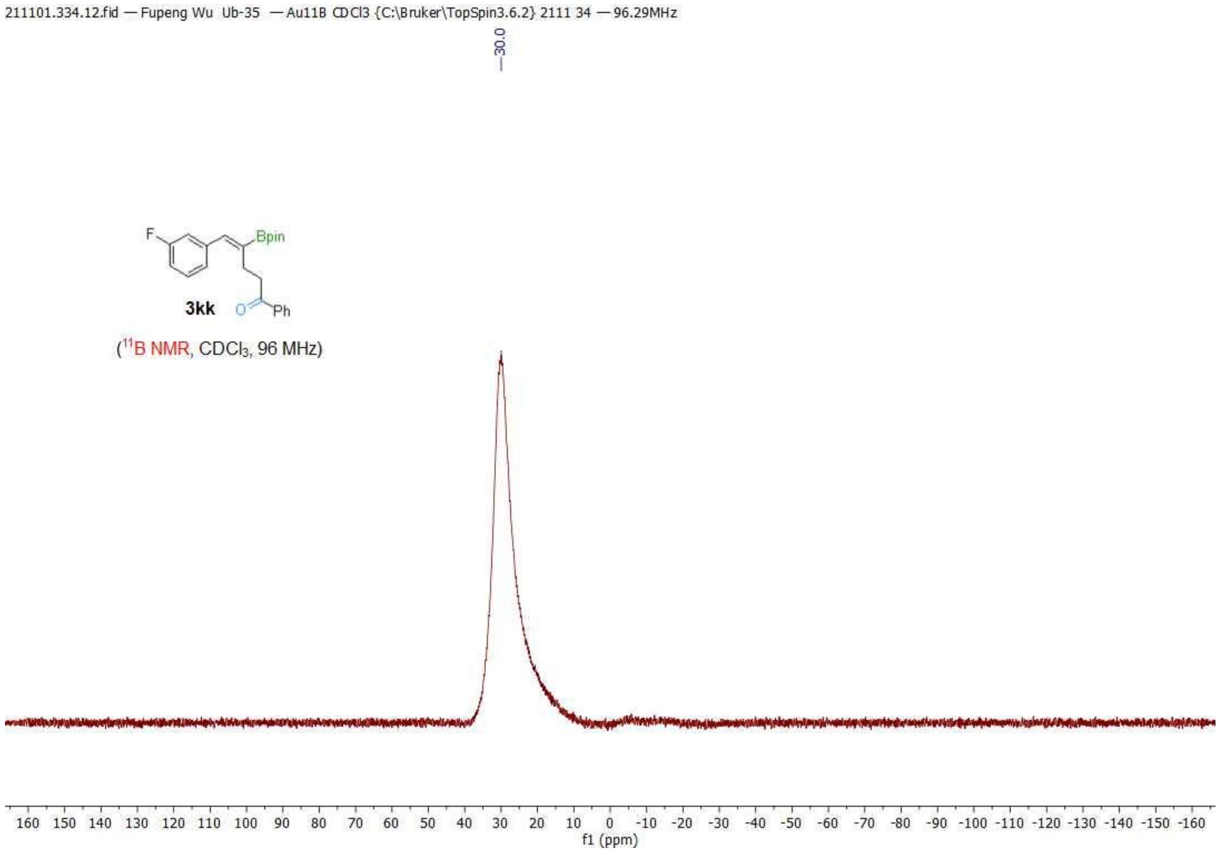
211101.334.12.fid — Fupeng Wu Ub-35 — Au11B CDCl3 {C:\Bruker\TopSpin3.6.2} 2111 34 — 96.29MHz
30.0
F
Bpin
3kk
O
Ph
(11B NMR, CDCl3, 96 MHz)
160 150 140 130 120 110 100 90 80 70 60 50 40 30 20 10 0 -10 -20 -30 -40 -50 -60 -70 -80 -90 -100 -110 -120 -130 -140 -150 -160
f1 (ppm)

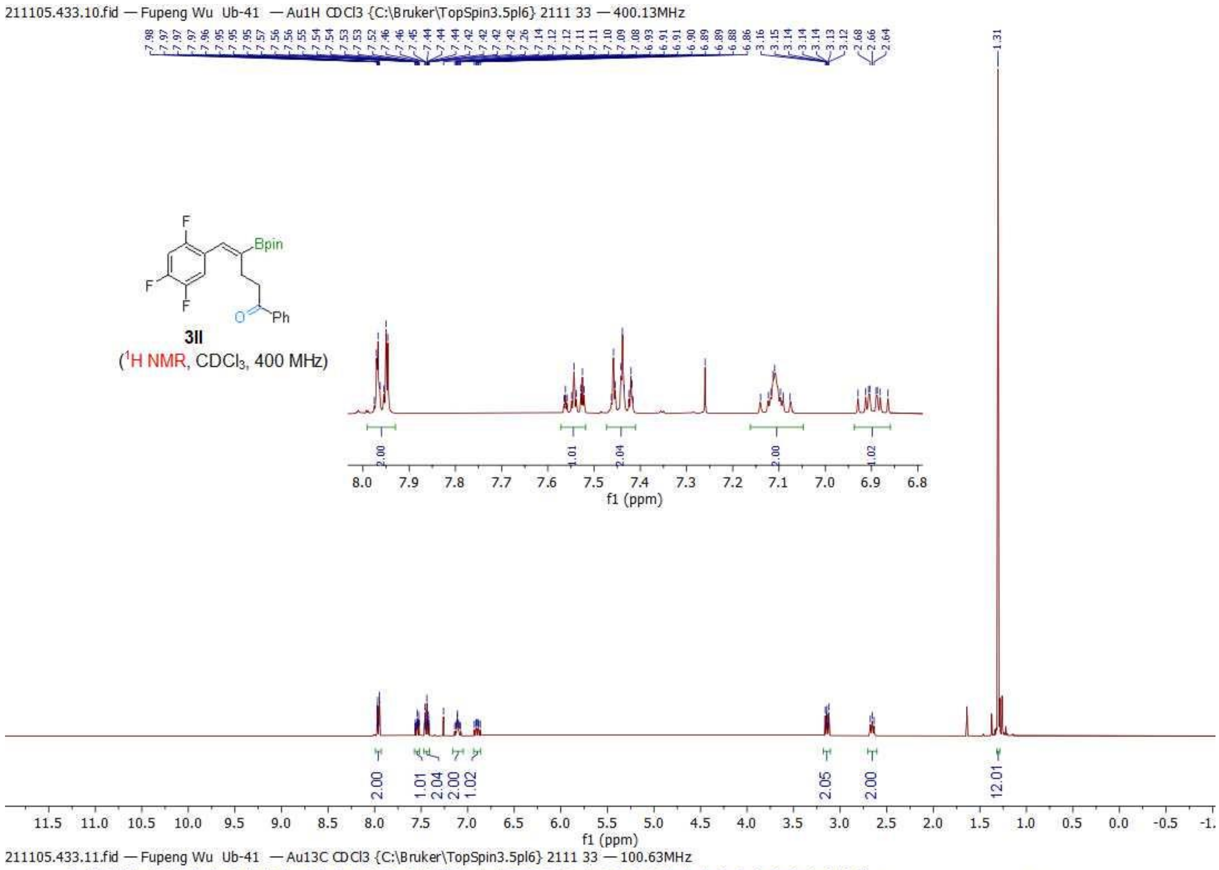
211105.433.10.fid — Fupeng Wu Ub-41 — Au1H CDCl3 {C:\Bruker\TopSpin3.5pl6} 2111 33 — 400.13MHz
3ll
(^{1}H NMR, CDCl$_3$, 400 MHz)
f1 (ppm)

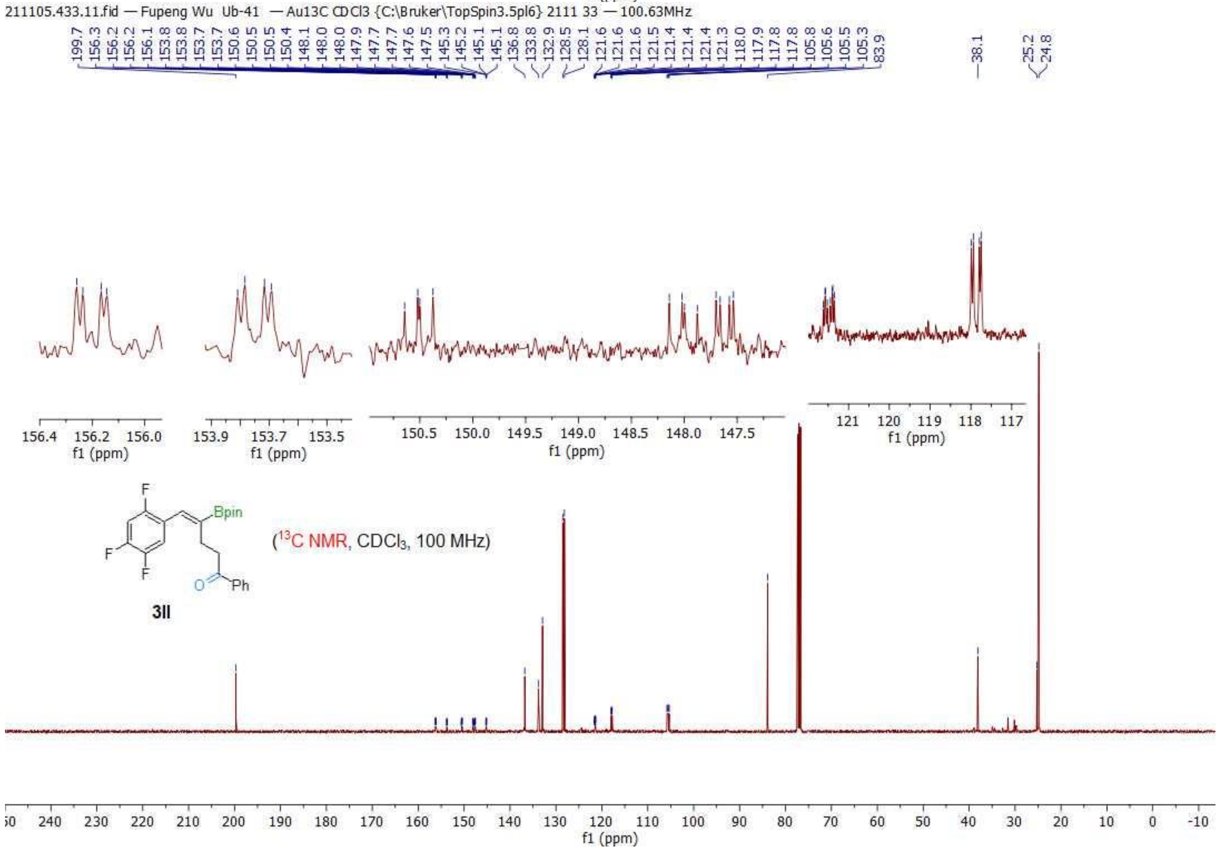
211105.433.11.fid — Fupeng Wu Ub-41 — Au13C CDCl3 {C:\Bruker\TopSpin3.5pl6} 2111 33 — 100.63MHz
(^{13}C NMR, CDCl$_3$, 100 MHz)
3ll
f1 (ppm)

211105.433.12.fid — Fupeng Wu Ub-41 — Au11B CDCl3 {C:\Bruker\TopSpin3.5pl6} 2111 33 — 128.38MHz

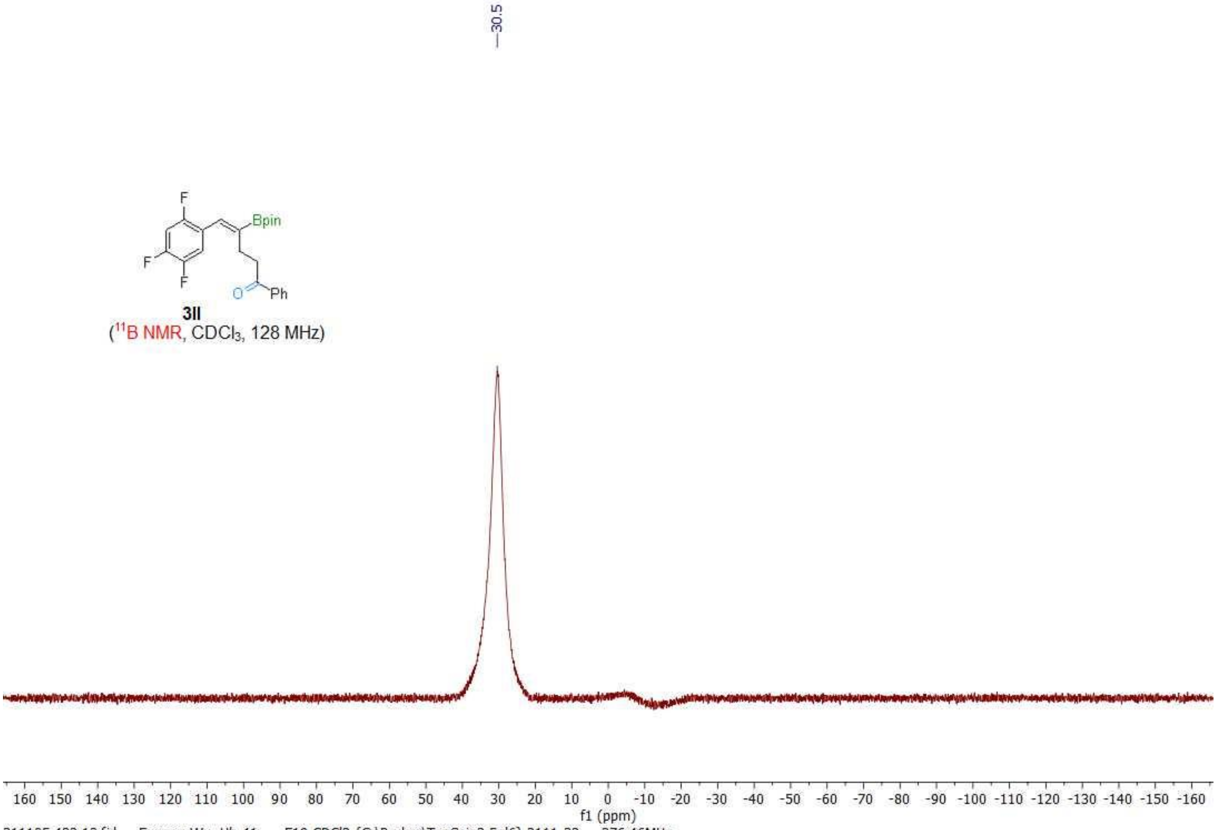

3ll
(^{11}B NMR, $CDCl_3$, 128 MHz)

211105.433.13.fid — Fupeng Wu Ub-41 — F19 CDCl3 {C:\Bruker\TopSpin3.5pl6} 2111 33 — 376.46MHz

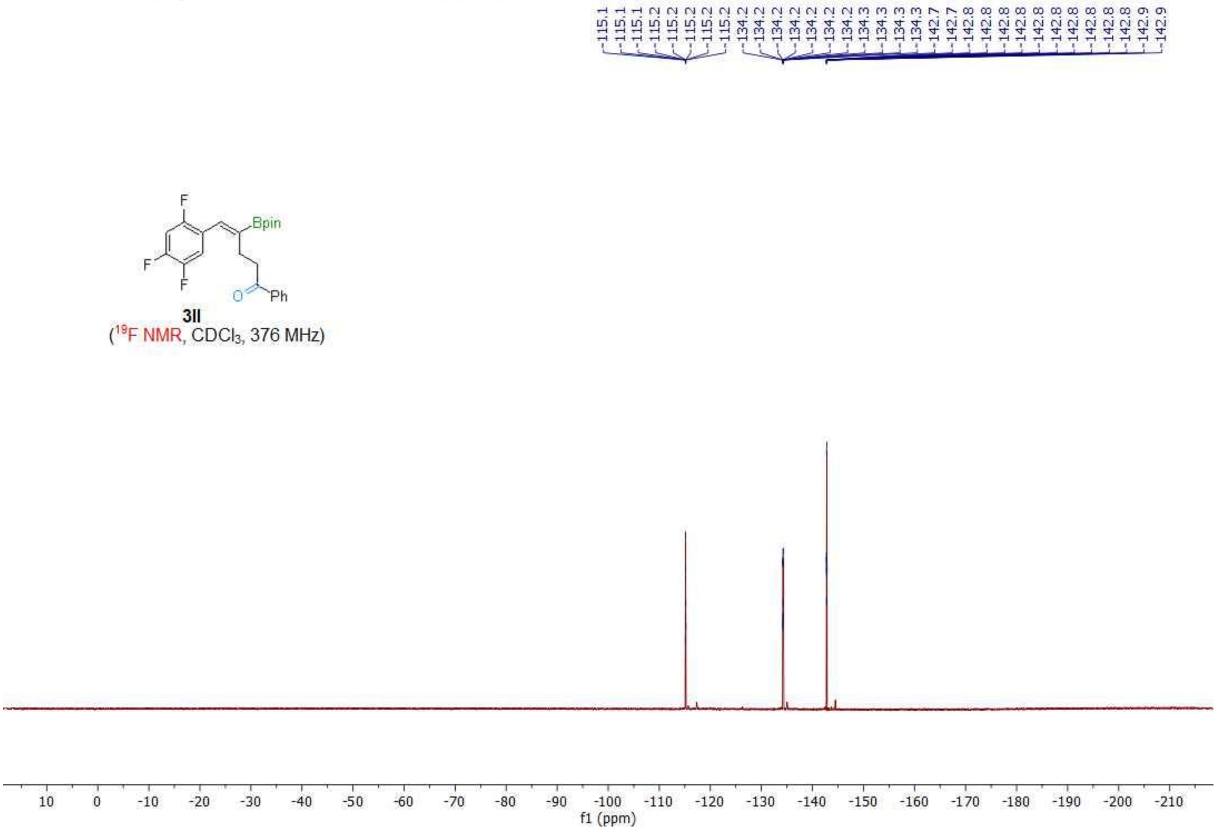

3ll
(^{19}F NMR, $CDCl_3$, 376 MHz)

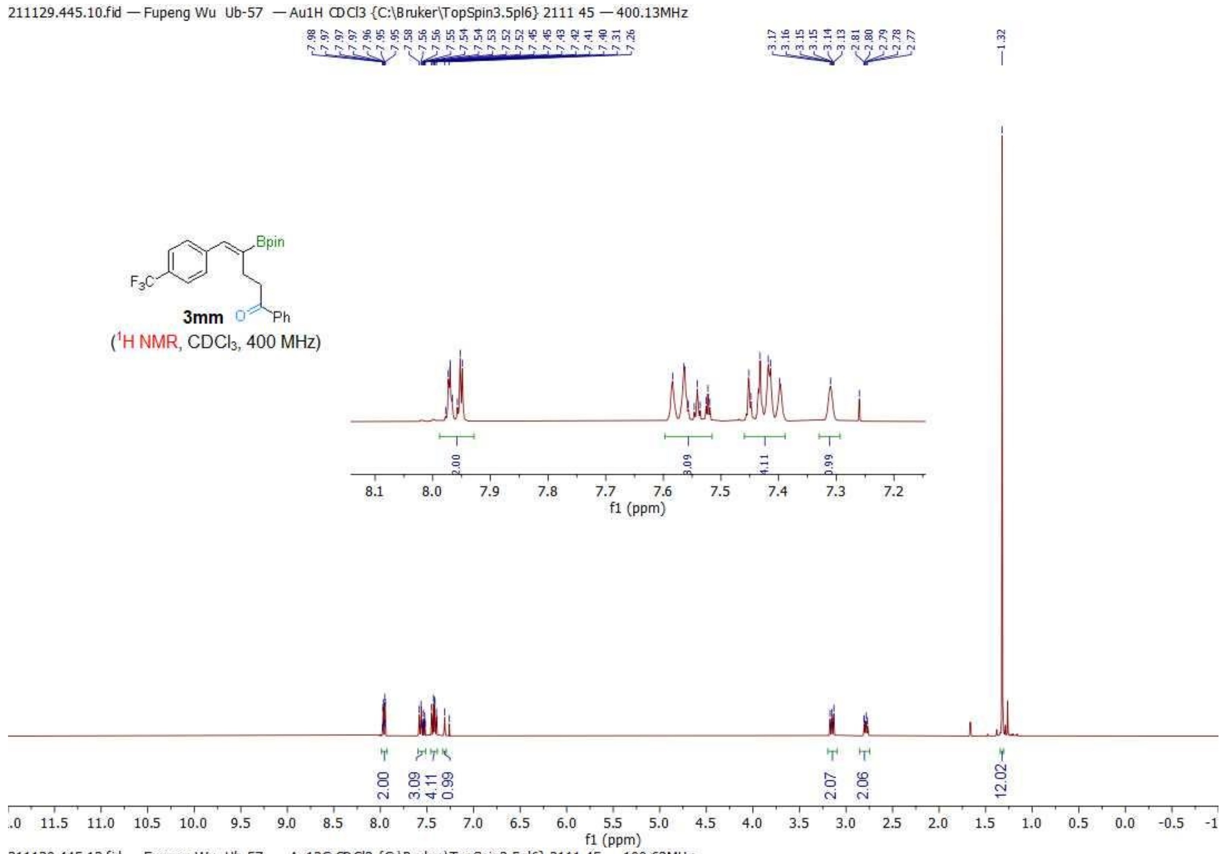
211129.445.10.fid — Fupeng Wu Ub-57 — Au1H CDCl3 {C:\Bruker\TopSpin3.5pl6} 2111 45 — 400.13MHz
7.98 7.97 7.97 7.97 7.96 7.95 7.95 7.58 7.56 7.56 7.55 7.54 7.54 7.53 7.52 7.52 7.45 7.43 7.42 7.41 7.40 7.31 7.26
3.17 3.16 3.15 3.15 3.14 3.13 2.81 2.80 2.79 2.78 2.77
1.32
Bpin
F3C
3mm
O
Ph
(1H NMR, CDCl3, 400 MHz)
2.00 3.09 4.11 0.99
f1 (ppm)
2.00 3.09 4.11 0.99 2.07 2.06 12.02
f1 (ppm)

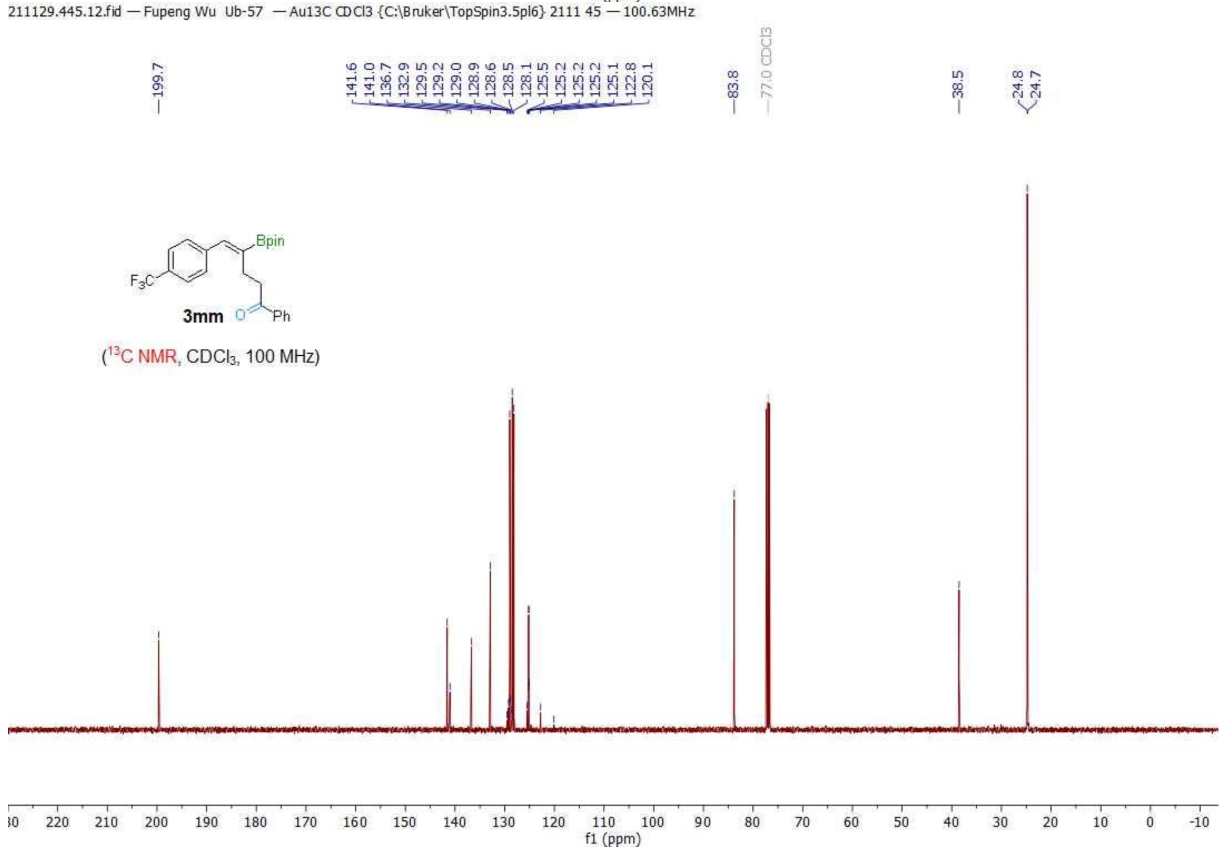
211129.445.12.fid — Fupeng Wu Ub-57 — Au13C CDCl3 {C:\Bruker\TopSpin3.5pl6} 2111 45 — 100.63MHz
199.7
141.6 141.0 136.7 132.9 129.5 129.2 129.0 128.9 128.6 128.5 128.1 125.5 125.2 125.2 125.2 125.1 122.8 120.1
83.8
77.0 CDCl3
38.5
24.8 24.7
Bpin
F3C
3mm
O
Ph
(13C NMR, CDCl3, 100 MHz)
f1 (ppm)

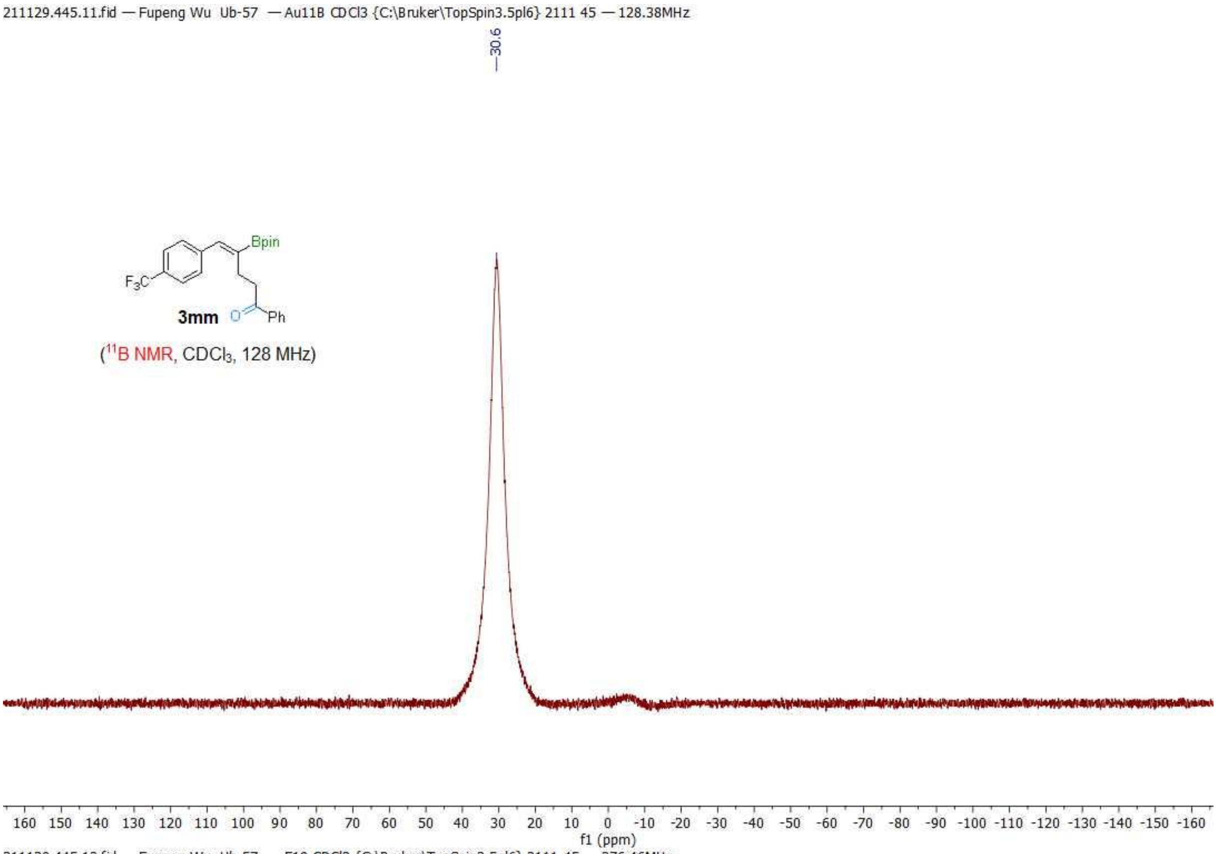
211129.445.11.fid — Fupeng Wu Ub-57 — Au11B CDCl3 {C:\Bruker\TopSpin3.5pl6} 2111 45 — 128.38MHz
30.6
Bpin
F3C
3mm
O
Ph
(11B NMR, CDCl3, 128 MHz)
f1 (ppm)

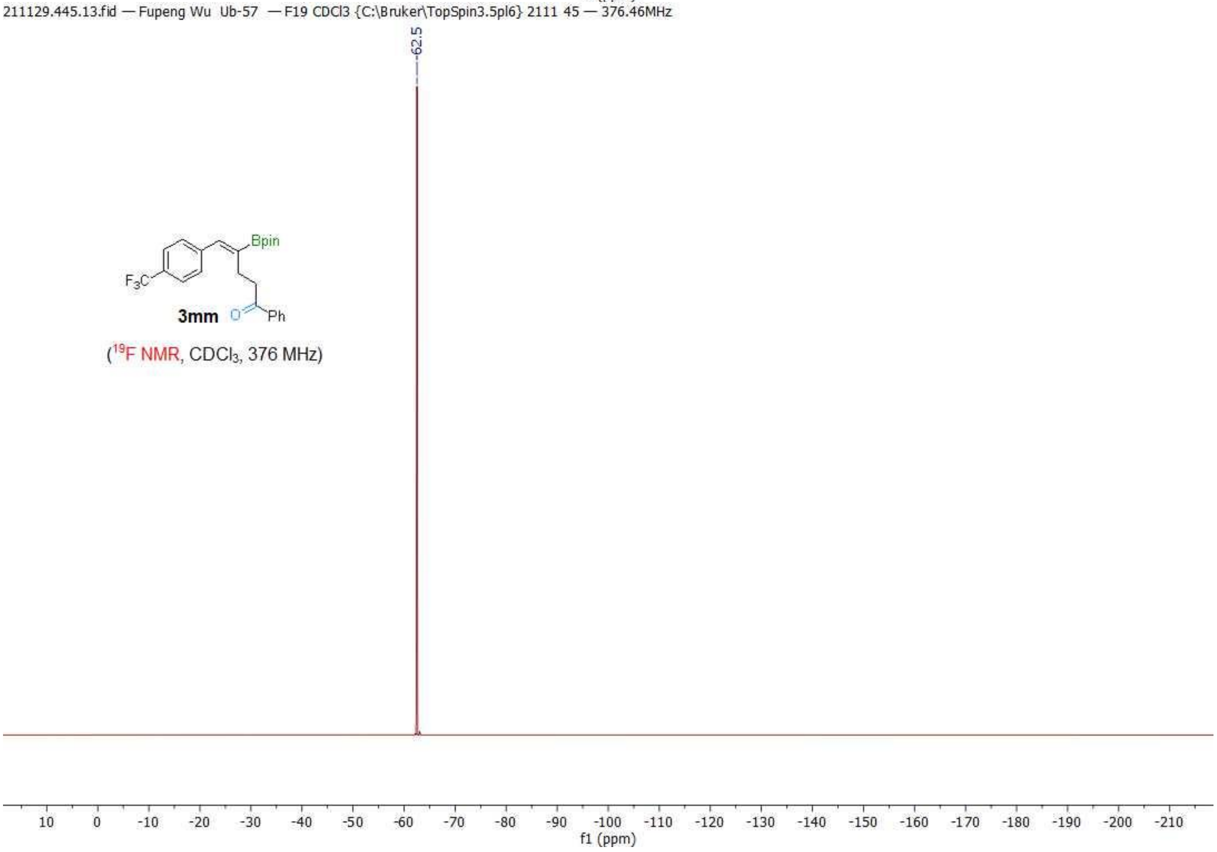
211129.445.13.fid — Fupeng Wu Ub-57 — F19 CDCl3 {C:\Bruker\TopSpin3.5pl6} 2111 45 — 376.46MHz
-62.5
Bpin
F3C
3mm
O
Ph
(19F NMR, CDCl3, 376 MHz)
f1 (ppm)

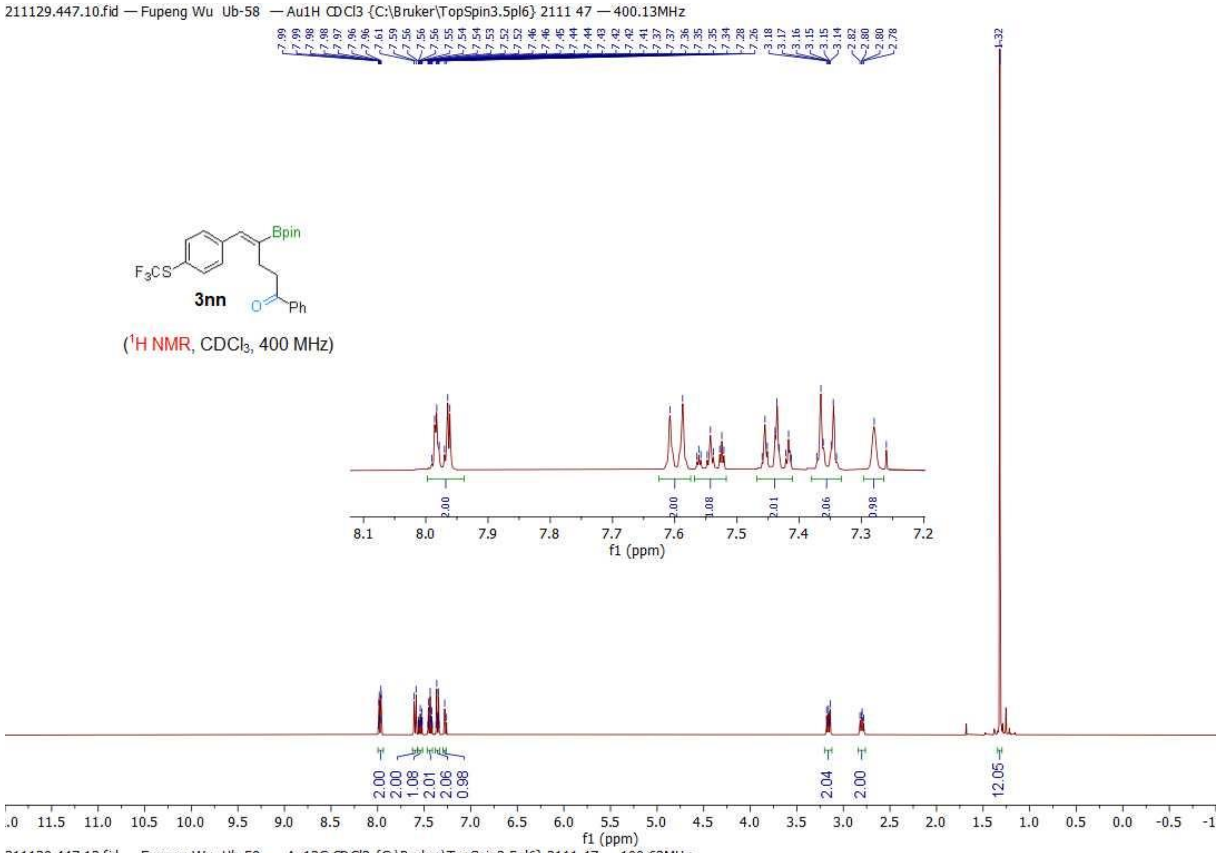
211129.447.10.fid — Fupeng Wu Ub-58 — Au1H CDCl3 {C:\Bruker\TopSpin3.5pl6} 2111 47 — 400.13MHz
7.99 7.99 7.98 7.97 7.97 7.96 7.96 7.61 7.59 7.56 7.56 7.56 7.55 7.54 7.54 7.53 7.52 7.52 7.46 7.45 7.44 7.43 7.42 7.42 7.41 7.37 7.36 7.35 7.35 7.34 7.28 7.26 3.18 3.17 3.16 3.15 3.15 3.14 2.82 2.80 2.80 2.78
1.32
Bpin
F3CS
3nn
O
Ph
(1H NMR, CDCl3, 400 MHz)
2.00 2.00 1.08 2.01 2.06 0.98
2.04 2.00 12.05
f1 (ppm)

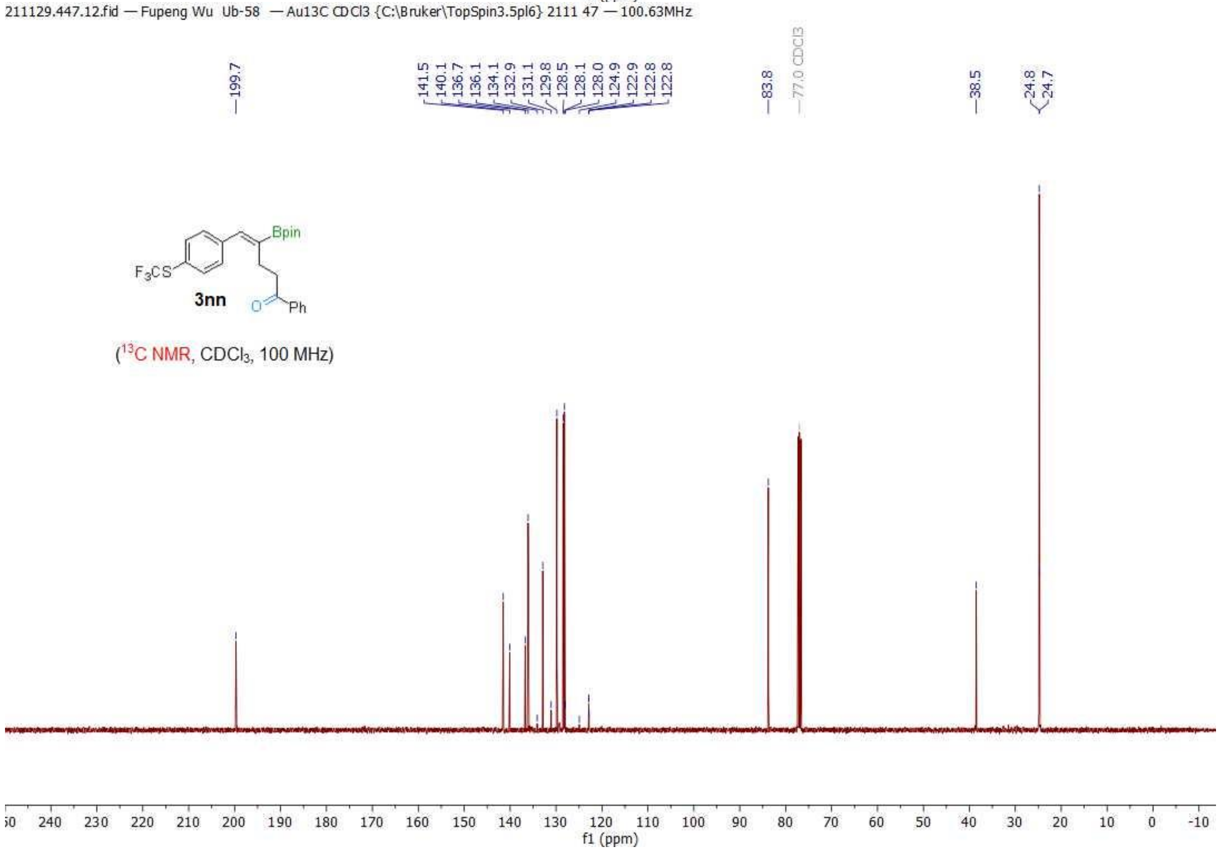
211129.447.12.fid — Fupeng Wu Ub-58 — Au13C CDCl3 {C:\Bruker\TopSpin3.5pl6} 2111 47 — 100.63MHz
199.7
141.5 140.1 136.7 136.1 134.1 132.9 131.1 129.8 128.5 128.1 128.0 124.9 122.9 122.8 122.8
83.8
77.0 CDCl3
38.5
24.8 24.7
Bpin
F3CS
3nn
O
Ph
(13C NMR, CDCl3, 100 MHz)
f1 (ppm)

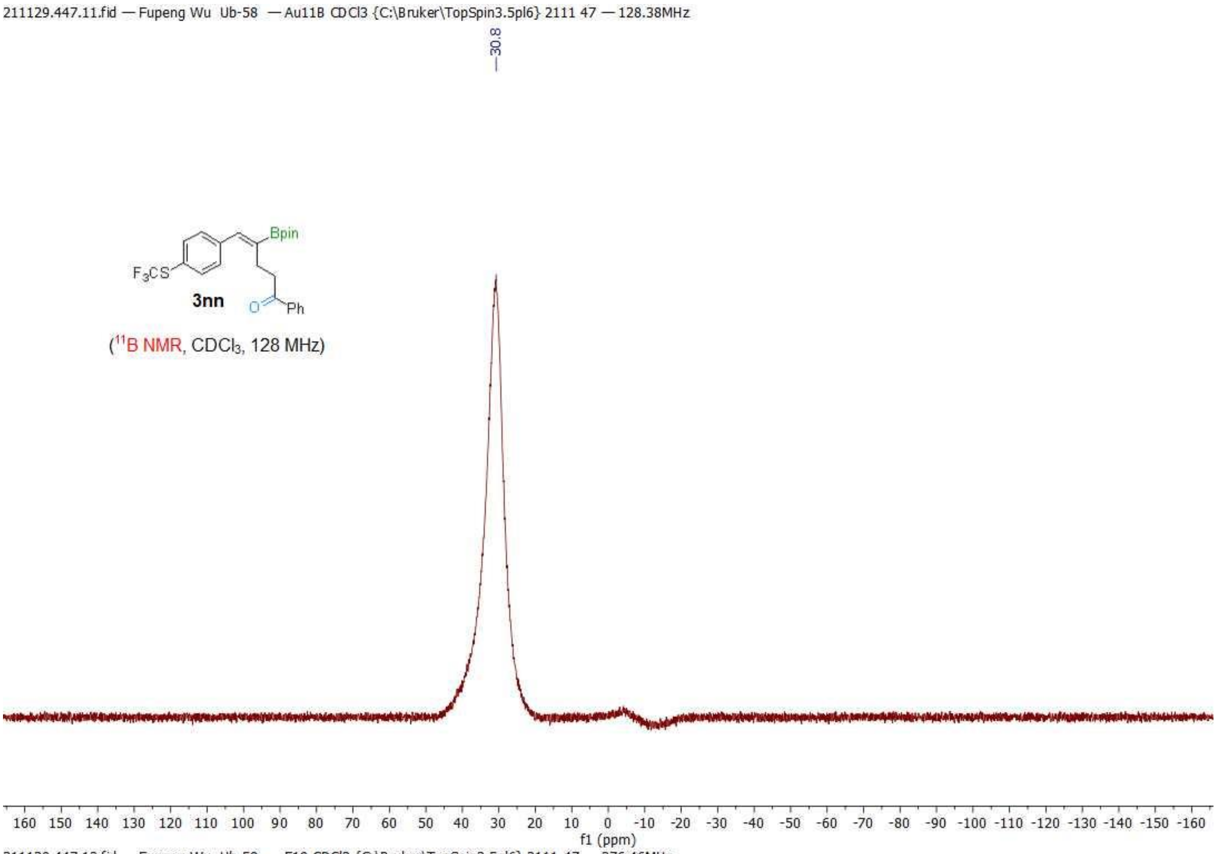
211129.447.11.fid — Fupeng Wu Ub-58 — Au11B CDCl3 {C:\Bruker\TopSpin3.5pl6} 2111 47 — 128.38MHz
30.8
Bpin
F3CS
3nn
O
Ph
(11B NMR, CDCl3, 128 MHz)
160 150 140 130 120 110 100 90 80 70 60 50 40 30 20 10 0 -10 -20 -30 -40 -50 -60 -70 -80 -90 -100 -110 -120 -130 -140 -150 -160
f1 (ppm)

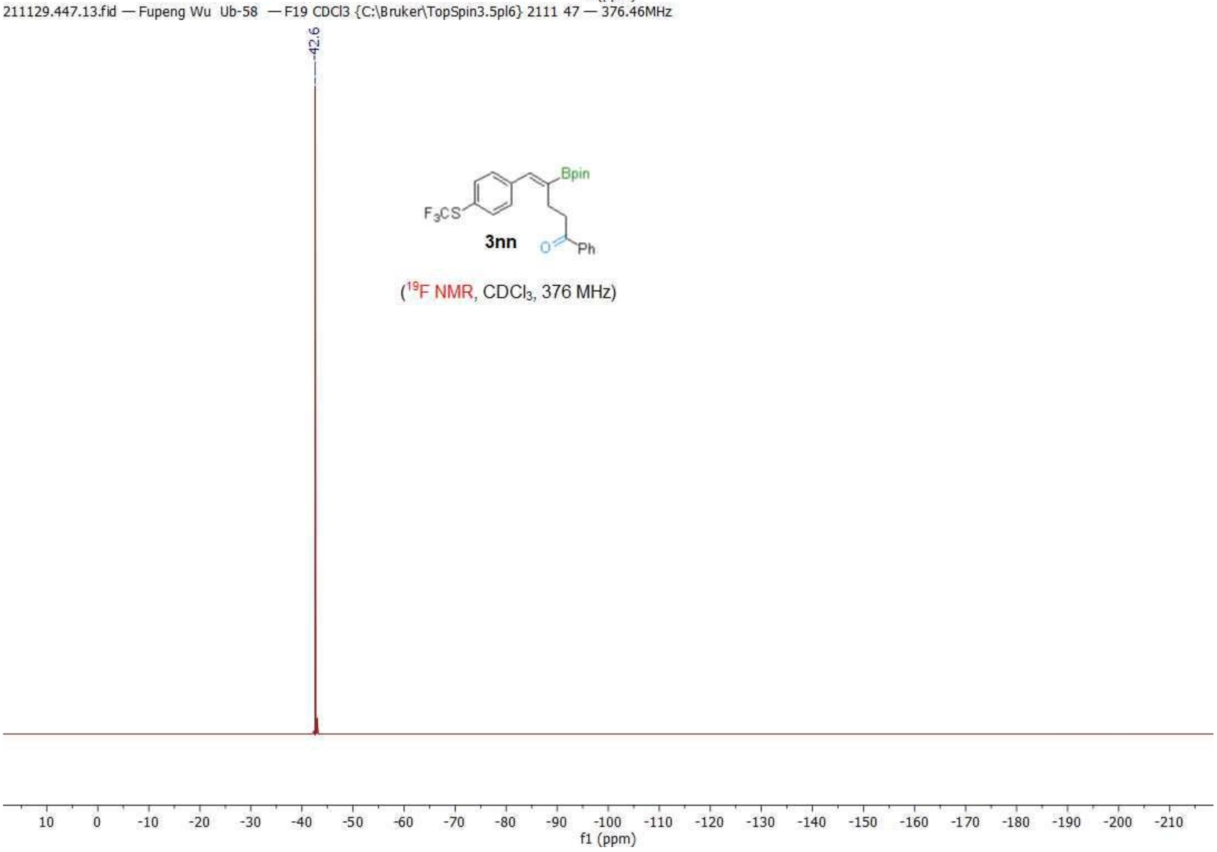
211129.447.13.fid — Fupeng Wu Ub-58 — F19 CDCl3 {C:\Bruker\TopSpin3.5pl6} 2111 47 — 376.46MHz
-42.6
Bpin
F3CS
3nn
O
Ph
(19F NMR, CDCl3, 376 MHz)
10 0 -10 -20 -30 -40 -50 -60 -70 -80 -90 -100 -110 -120 -130 -140 -150 -160 -170 -180 -190 -200 -210
f1 (ppm)

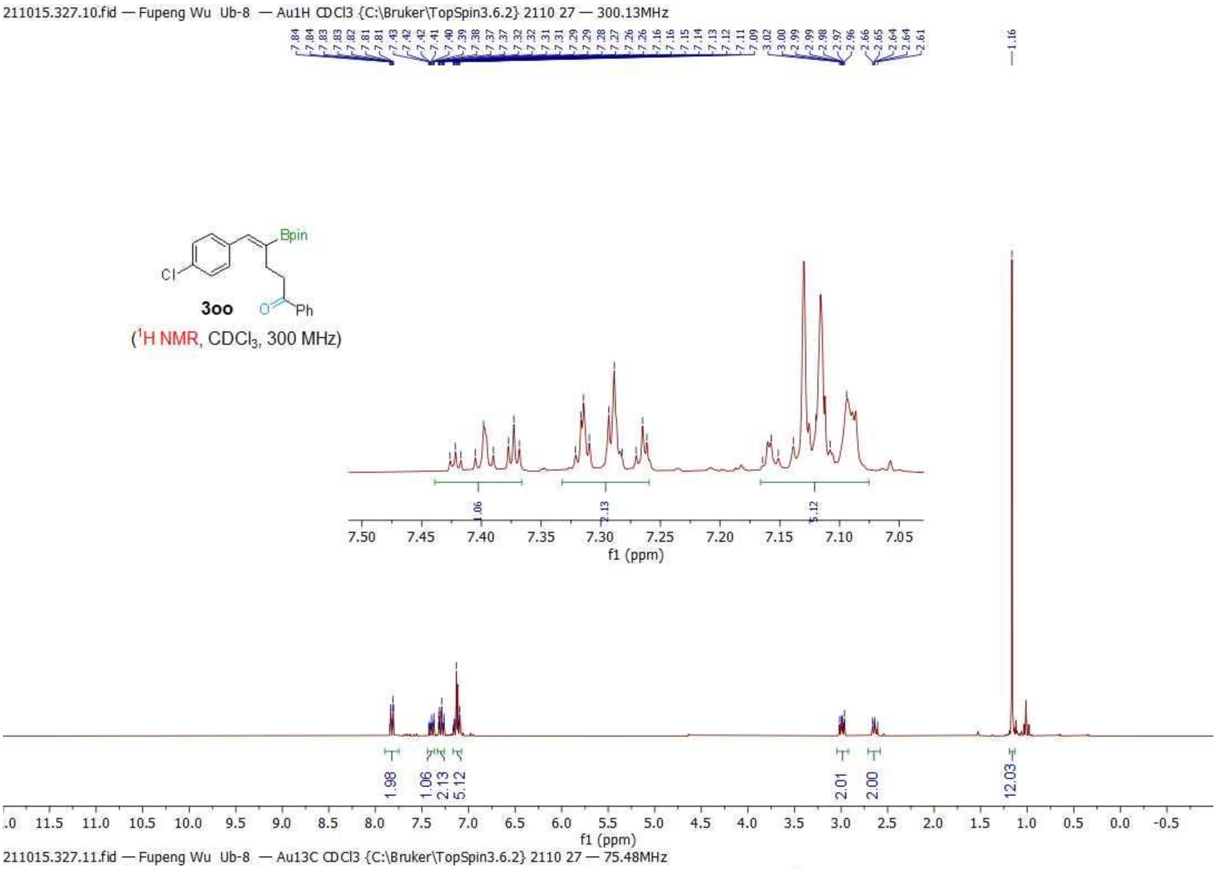
211015.327.10.fid — Fupeng Wu Ub-8 — Au1H CDCl3 {C:\Bruker\TopSpin3.6.2} 2110 27 — 300.13MHz
7.84 7.84 7.83 7.83 7.82 7.81 7.81 7.43 7.42 7.42 7.41 7.40 7.39 7.38 7.37 7.37 7.32 7.32 7.31 7.31 7.29 7.29 7.28 7.27 7.26 7.26 7.16 7.16 7.15 7.14 7.13 7.12 7.11 7.09 3.02 3.00 2.99 2.98 2.97 2.96 2.66 2.65 2.64 2.64 2.61 1.16
Bpin
Cl
3oo
O
Ph
(1H NMR, CDCl3, 300 MHz)
1.06
2.13
5.12
f1 (ppm)
1.98
1.06
2.13
5.12
2.01
2.00
12.03
f1 (ppm)

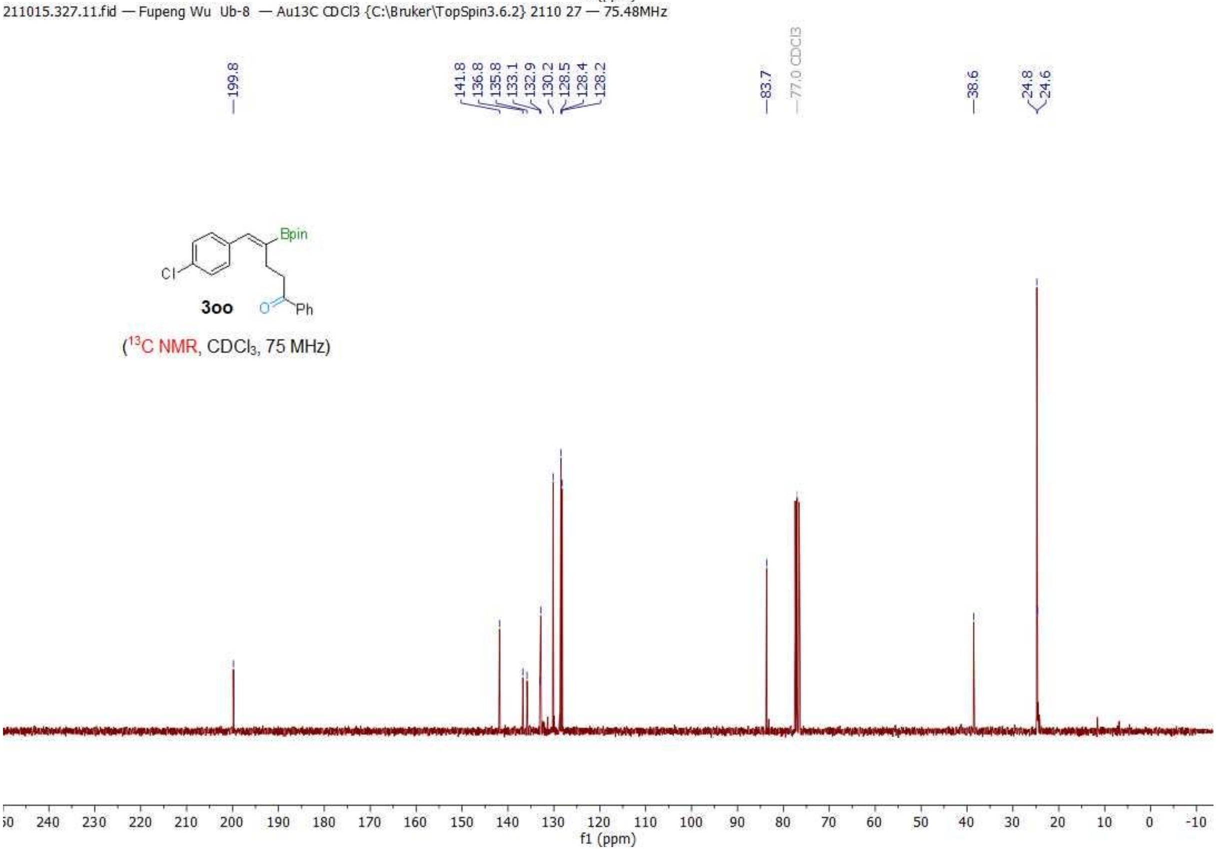
211015.327.11.fid — Fupeng Wu Ub-8 — Au13C CDCl3 {C:\Bruker\TopSpin3.6.2} 2110 27 — 75.48MHz
199.8
141.8 136.8 135.8 133.1 132.9 130.2 128.5 128.4 128.2
83.7
77.0 CDCl3
38.6
24.8 24.6
Bpin
Cl
3oo
O
Ph
(13C NMR, CDCl3, 75 MHz)
f1 (ppm)

211015.327.12.fid — Fupeng Wu Ub-8 — Au11B CDCl3 {C:\Bruker\TopSpin3.6.2} 2110 27 — 96.29MHz

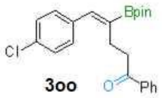

3oo

(^{11}B NMR, CDCl$_3$, 96 MHz)

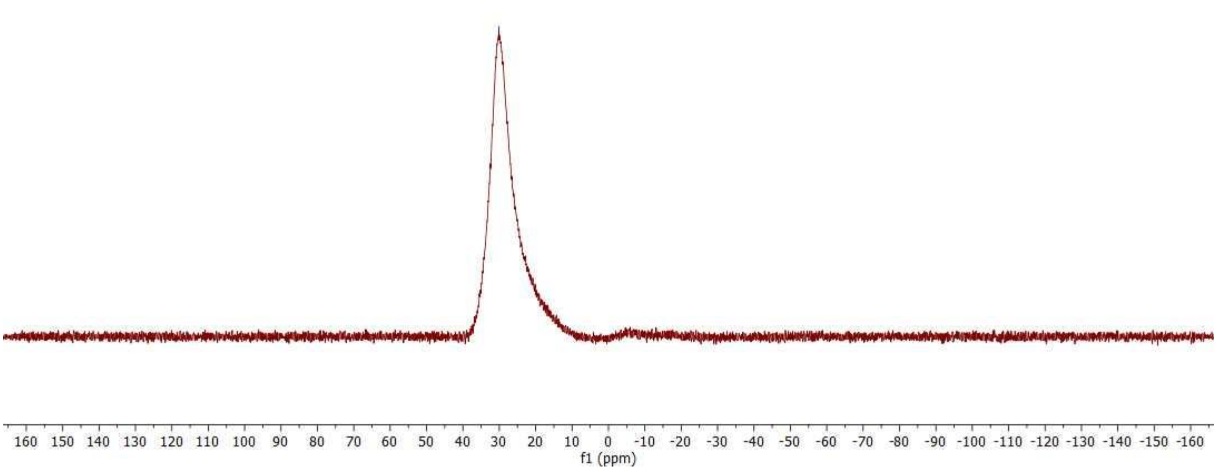

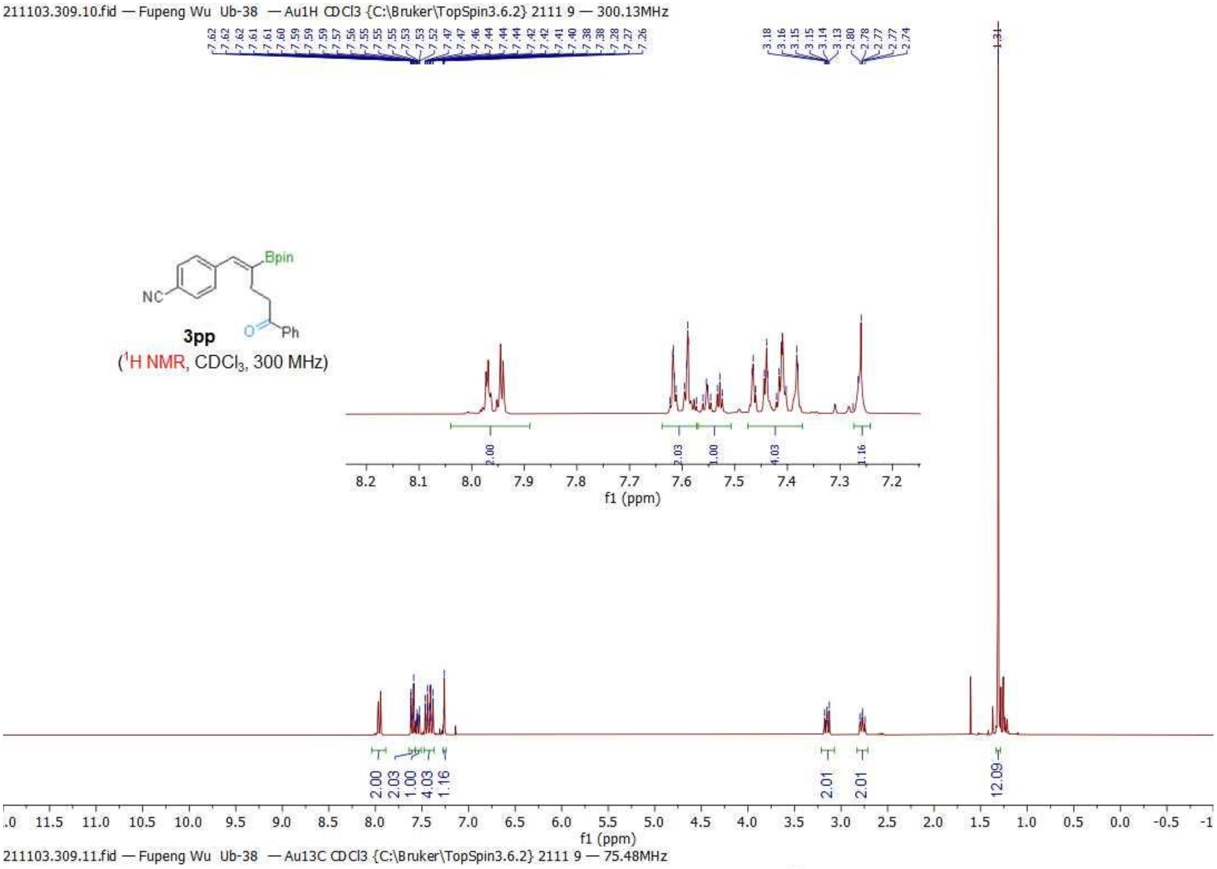
211103.309.10.fid — Fupeng Wu Ub-38 — Au1H CDCl3 {C:\Bruker\TopSpin3.6.2} 2111 9 — 300.13MHz
7.62 7.62 7.61 7.60 7.59 7.59 7.59 7.57 7.56 7.55 7.55 7.55 7.53 7.53 7.52 7.47 7.47 7.46 7.44 7.44 7.42 7.42 7.41 7.40 7.38 7.38 7.28 7.27 7.26
3.18 3.16 3.15 3.15 3.14 3.13 2.80 2.78 2.77 2.77 2.74
1.31
Bpin
NC
Ph
O
3pp
(1H NMR, CDCl3, 300 MHz)
2.00 2.03 1.00 4.03 1.16
2.01 2.01 12.09
f1 (ppm)

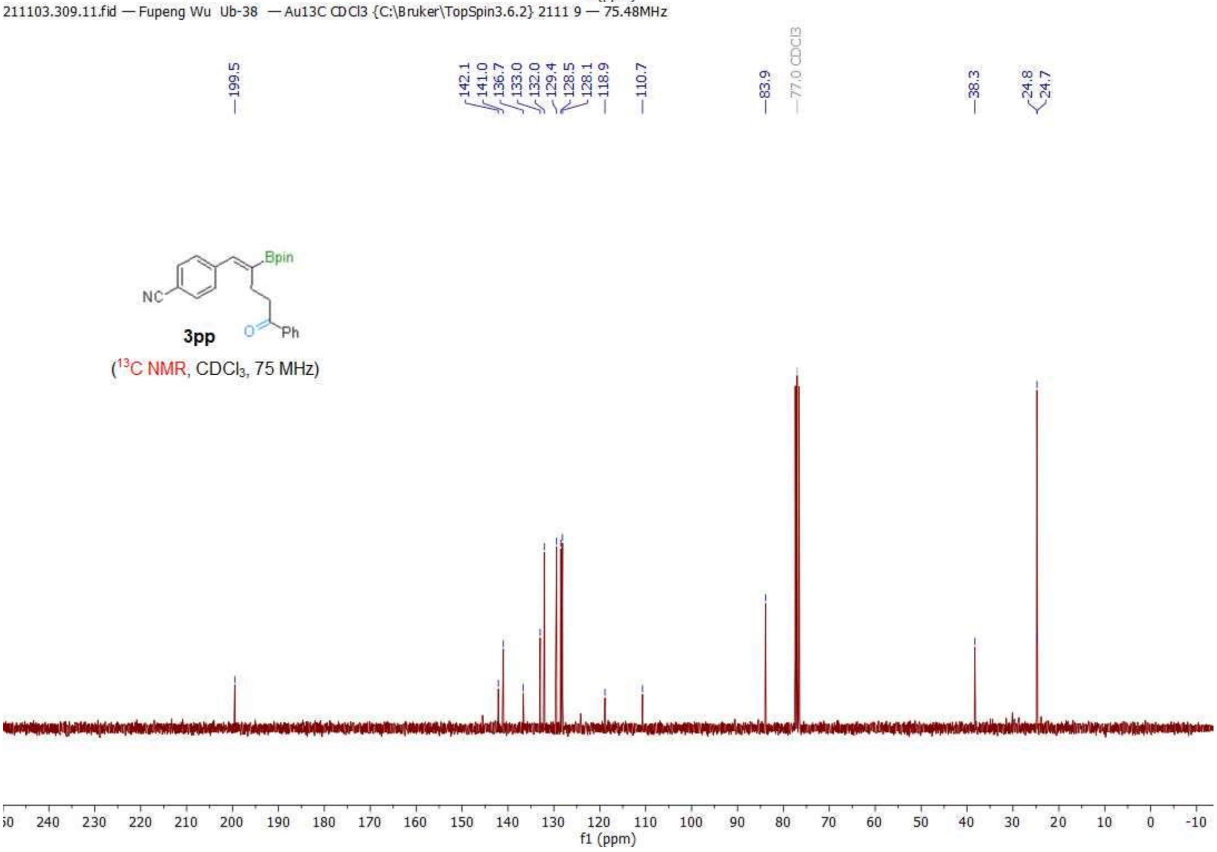
211103.309.11.fid — Fupeng Wu Ub-38 — Au13C CDCl3 {C:\Bruker\TopSpin3.6.2} 2111 9 — 75.48MHz
199.5
142.1 141.0 136.7 133.0 132.0 129.4 128.5 128.1 118.9
110.7
83.9
77.0 CDCl3
38.3
24.8 24.7
Bpin
NC
Ph
O
3pp
(13C NMR, CDCl3, 75 MHz)
f1 (ppm)

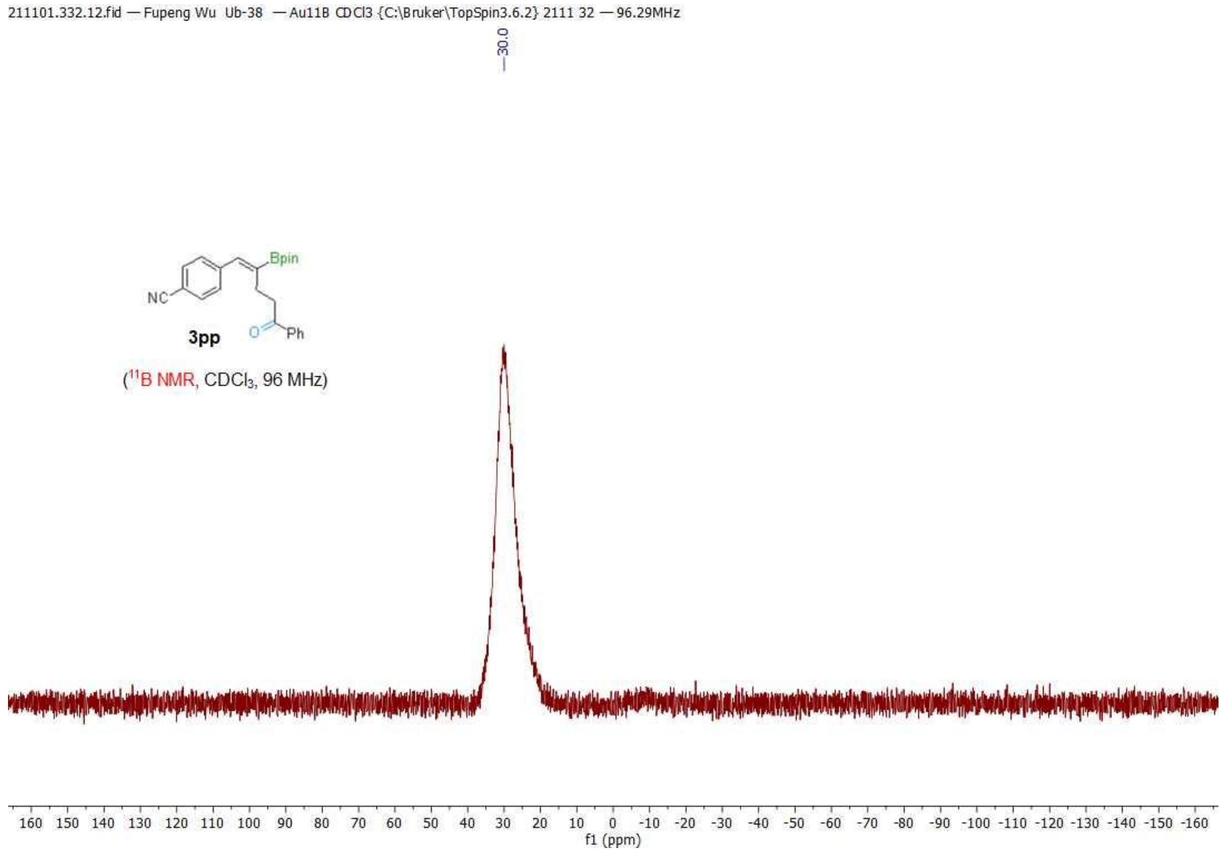
211101.332.12.fid — Fupeng Wu Ub-38 — Au11B CDCl3 {C:\Bruker\TopSpin3.6.2} 2111 32 — 96.29MHz
—30.0
Bpin
NC
O
Ph
3pp
(11B NMR, CDCl3, 96 MHz)
160 150 140 130 120 110 100 90 80 70 60 50 40 30 20 10 0 -10 -20 -30 -40 -50 -60 -70 -80 -90 -100 -110 -120 -130 -140 -150 -160
f1 (ppm)

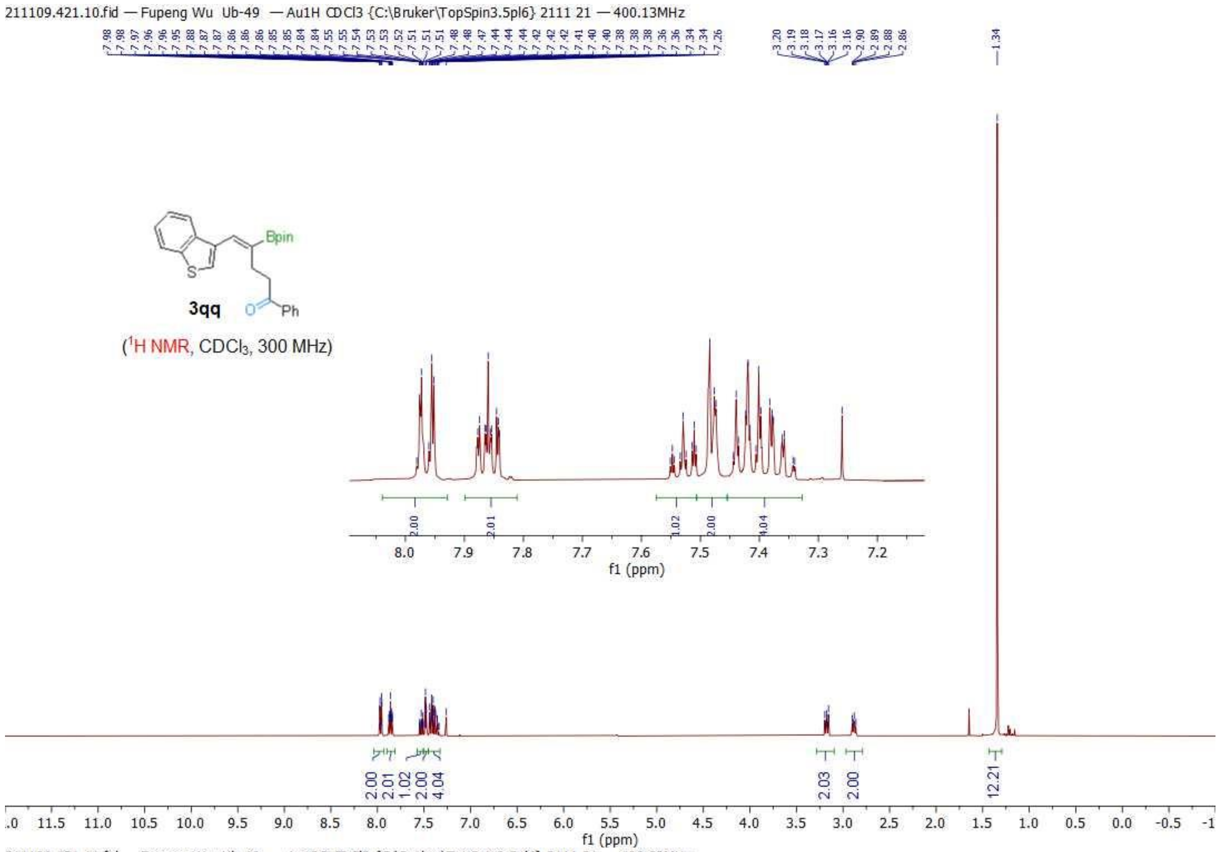
211109.421.10.fid — Fupeng Wu Ub-49 — Au1H CDCl3 {C:\Bruker\TopSpin3.5pl6} 2111 21 — 400.13MHz
Bpin
S
3qq
O
Ph
(1H NMR, CDCl3, 300 MHz)
2.00
2.01
1.02
2.00
4.04
2.03
2.00
12.21
f1 (ppm)

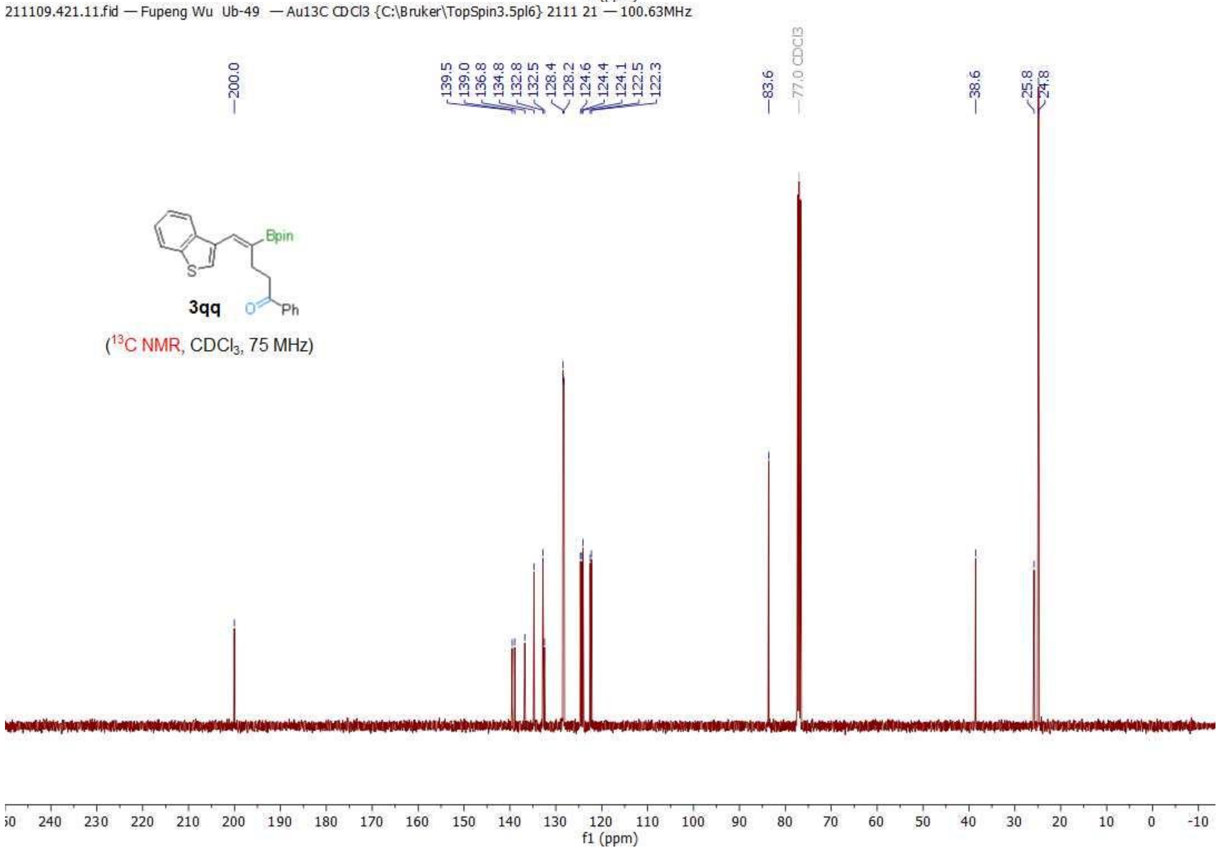
211109.421.11.fid — Fupeng Wu Ub-49 — Au13C CDCl3 {C:\Bruker\TopSpin3.5pl6} 2111 21 — 100.63MHz
200.0
139.5
139.0
136.8
134.8
132.8
132.5
128.4
128.2
124.6
124.4
124.1
122.5
122.3
83.6
77.0 CDCl3
38.6
25.8
24.8
Bpin
S
3qq
O
Ph
(13C NMR, CDCl3, 75 MHz)
f1 (ppm)

211109.421.12.fid — Fupeng Wu Ub-49 — Au11B CDCl3 {C:\Bruker\TopSpin3.5pl6} 2111 21 — 128.38MHz

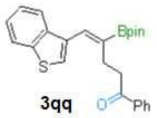

3qq

(^{11}B NMR, CDCl$_3$, 96 MHz)

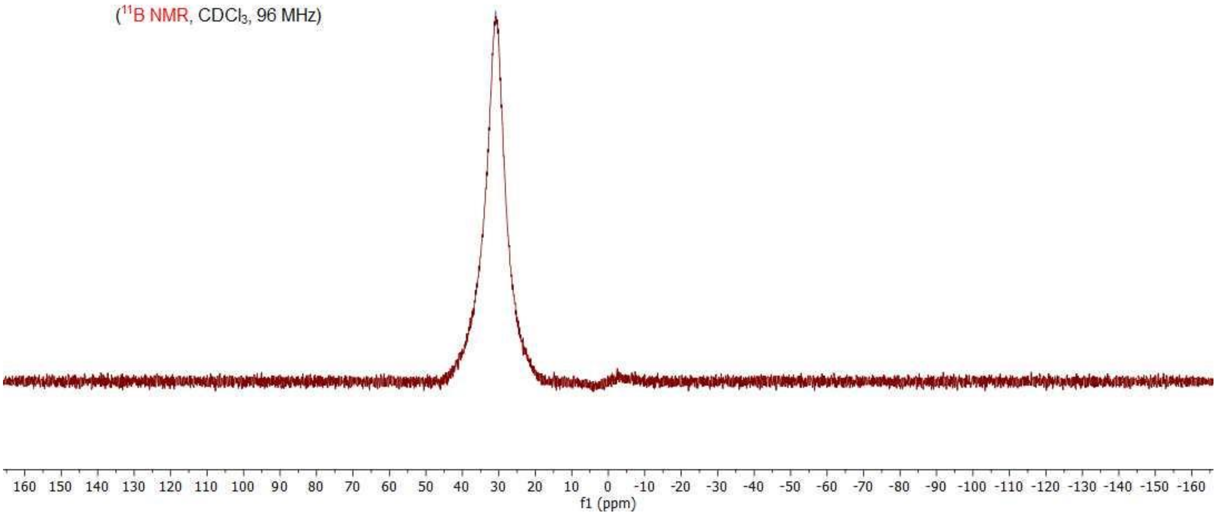

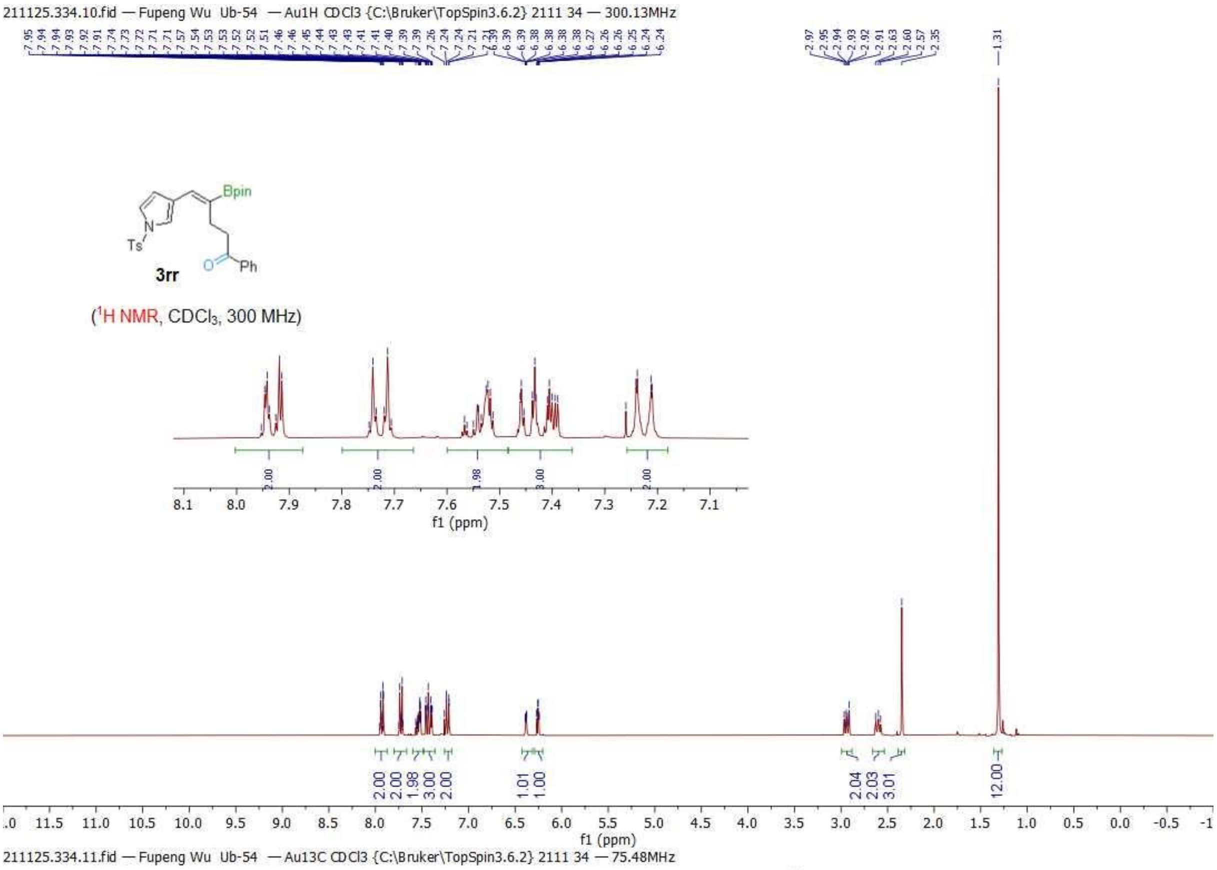
211125.334.10.fid — Fupeng Wu Ub-54 — Au1H CDCl3 {C:\Bruker\TopSpin3.6.2} 2111 34 — 300.13MHz
Bpin
N
Ts
O
Ph
3rr
(¹H NMR, CDCl₃, 300 MHz)
f1 (ppm)

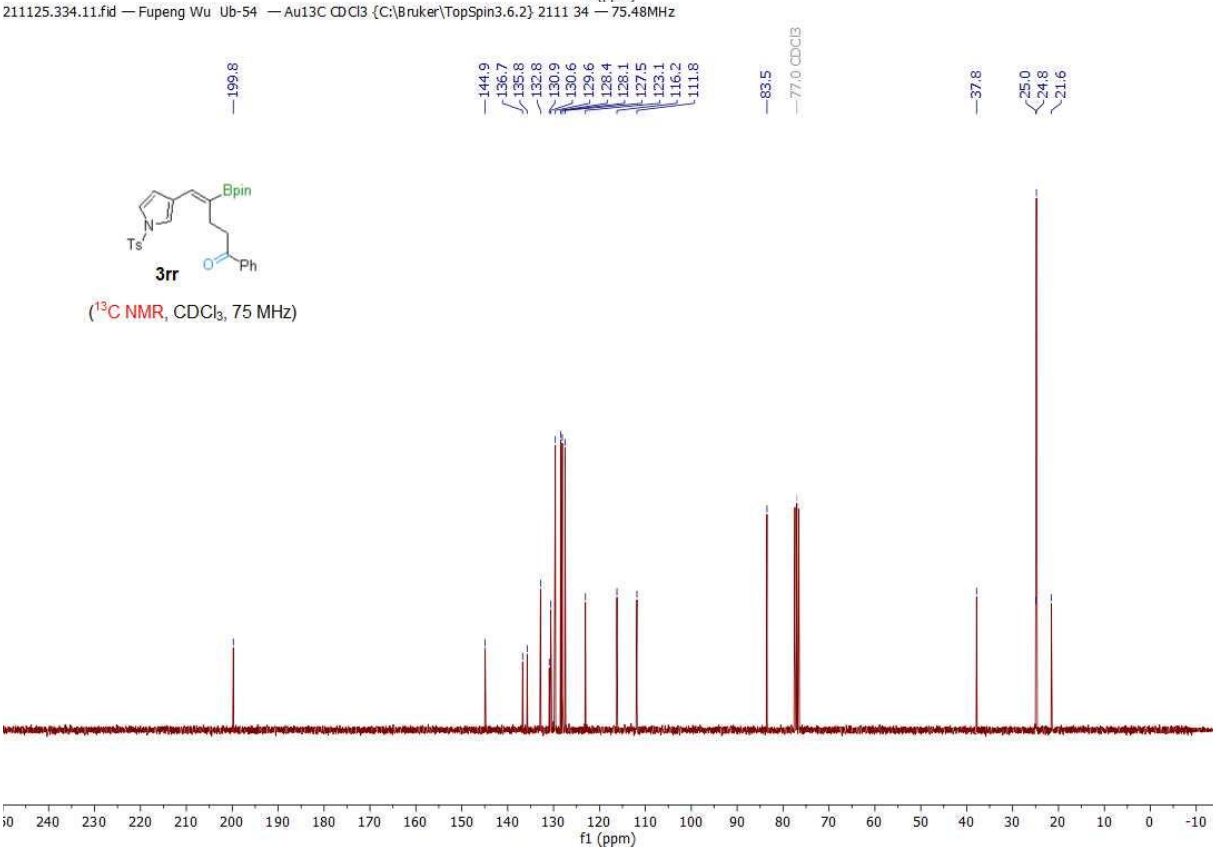
211125.334.11.fid — Fupeng Wu Ub-54 — Au13C CDCl3 {C:\Bruker\TopSpin3.6.2} 2111 34 — 75.48MHz
199.8
144.9
136.7
135.8
132.8
130.9
130.6
129.6
128.4
128.1
127.5
123.1
116.2
111.8
83.5
77.0 CDCl3
37.8
25.0
24.8
21.6
Bpin
N
Ts
O
Ph
3rr
(¹³C NMR, CDCl₃, 75 MHz)
f1 (ppm)

211125.334.12.fid — Fupeng Wu Ub-54 — Au11B CDCl3 {C:\Bruker\TopSpin3.6.2} 2111 34 — 96.29MHz

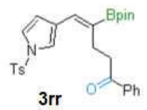

3rr

(^{11}B NMR, $CDCl_3$, 96 MHz)

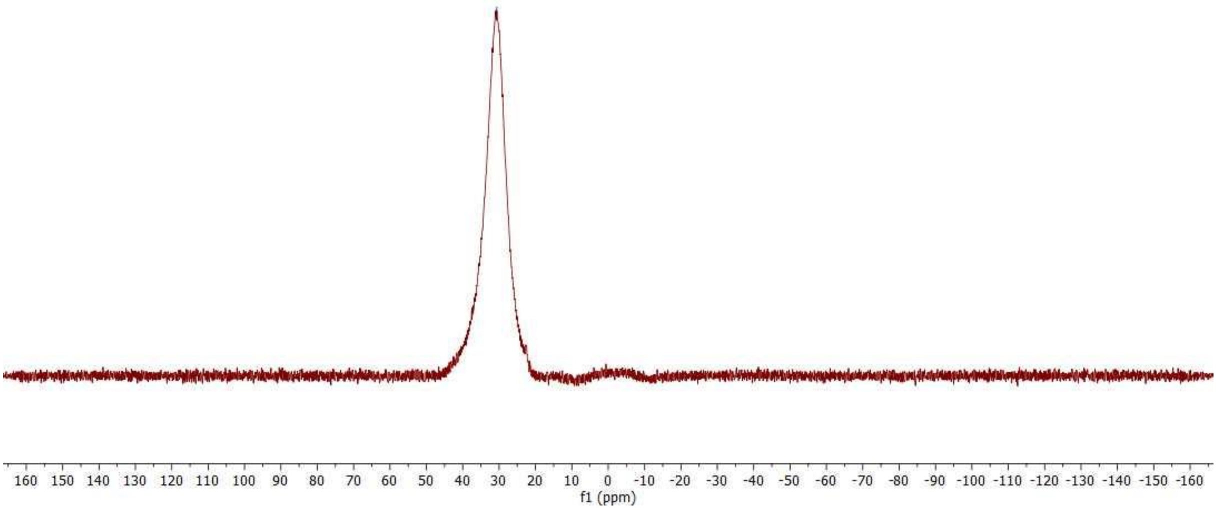

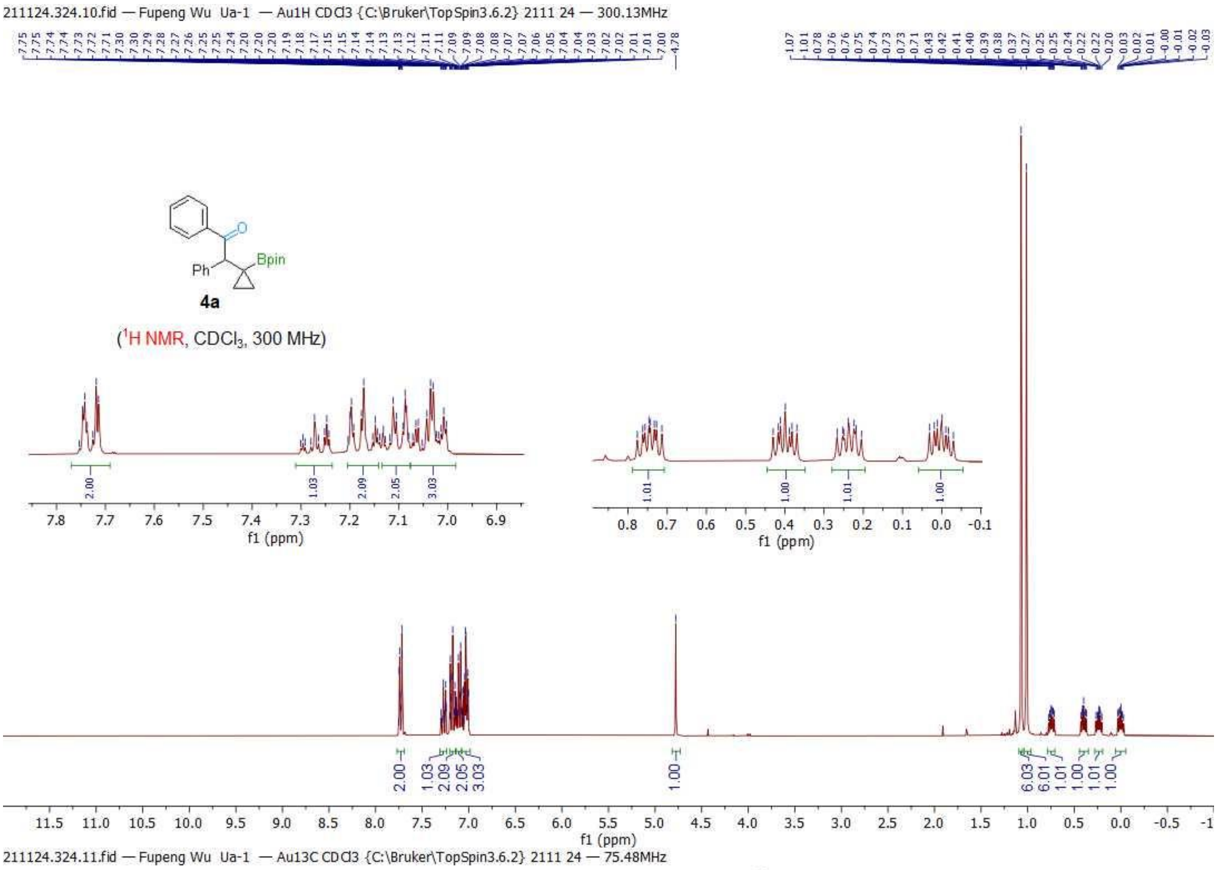
211124.324.10.fid — Fupeng Wu Ua-1 — Au1H CDCl3 {C:\Bruker\TopSpin3.6.2} 2111 24 — 300.13MHz
Ph
Bpin
O
4a
(1H NMR, CDCl3, 300 MHz)
f1 (ppm)

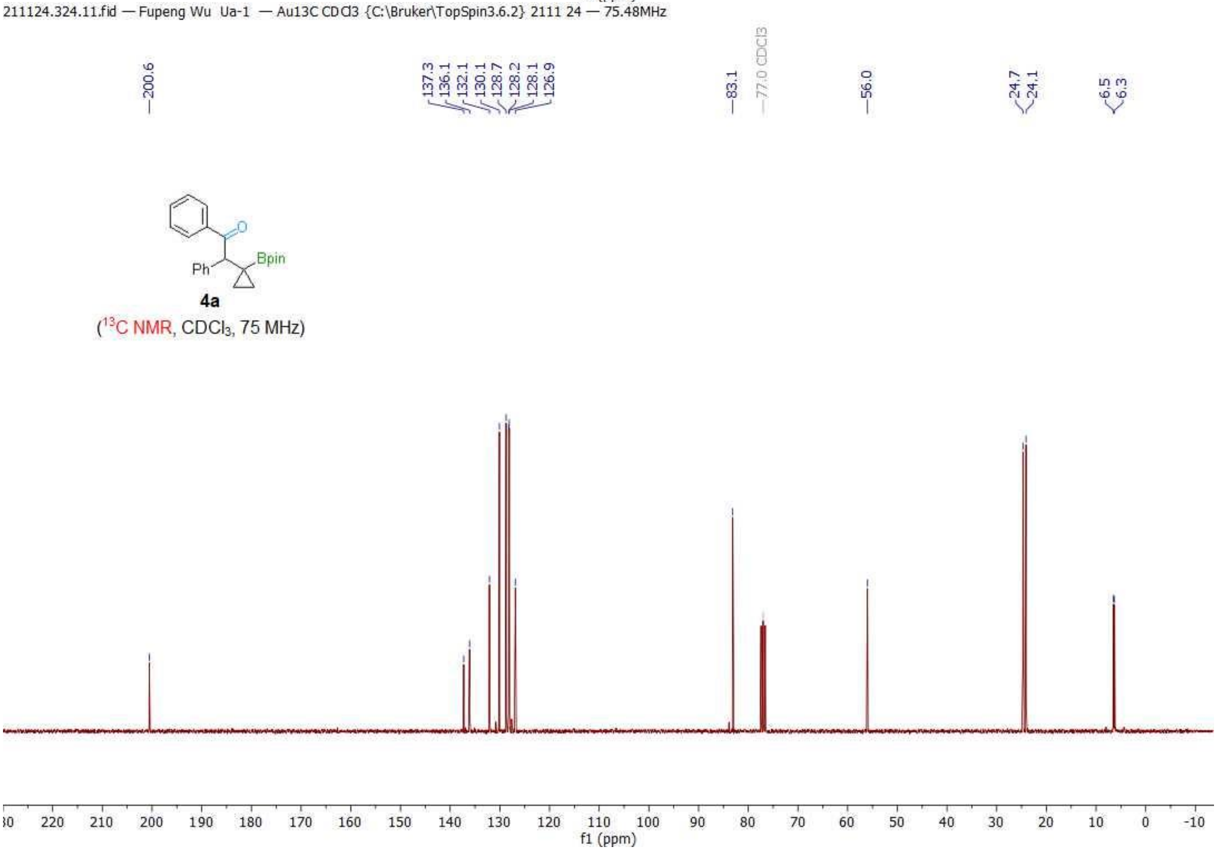
211124.324.11.fid — Fupeng Wu Ua-1 — Au13C CDCl3 {C:\Bruker\TopSpin3.6.2} 2111 24 — 75.48MHz
200.6
137.3
136.1
132.1
130.1
128.7
128.2
128.1
126.9
83.1
77.0 CDCl3
56.0
24.7
24.1
6.5
6.3
Ph
Bpin
O
4a
(13C NMR, CDCl3, 75 MHz)
f1 (ppm)

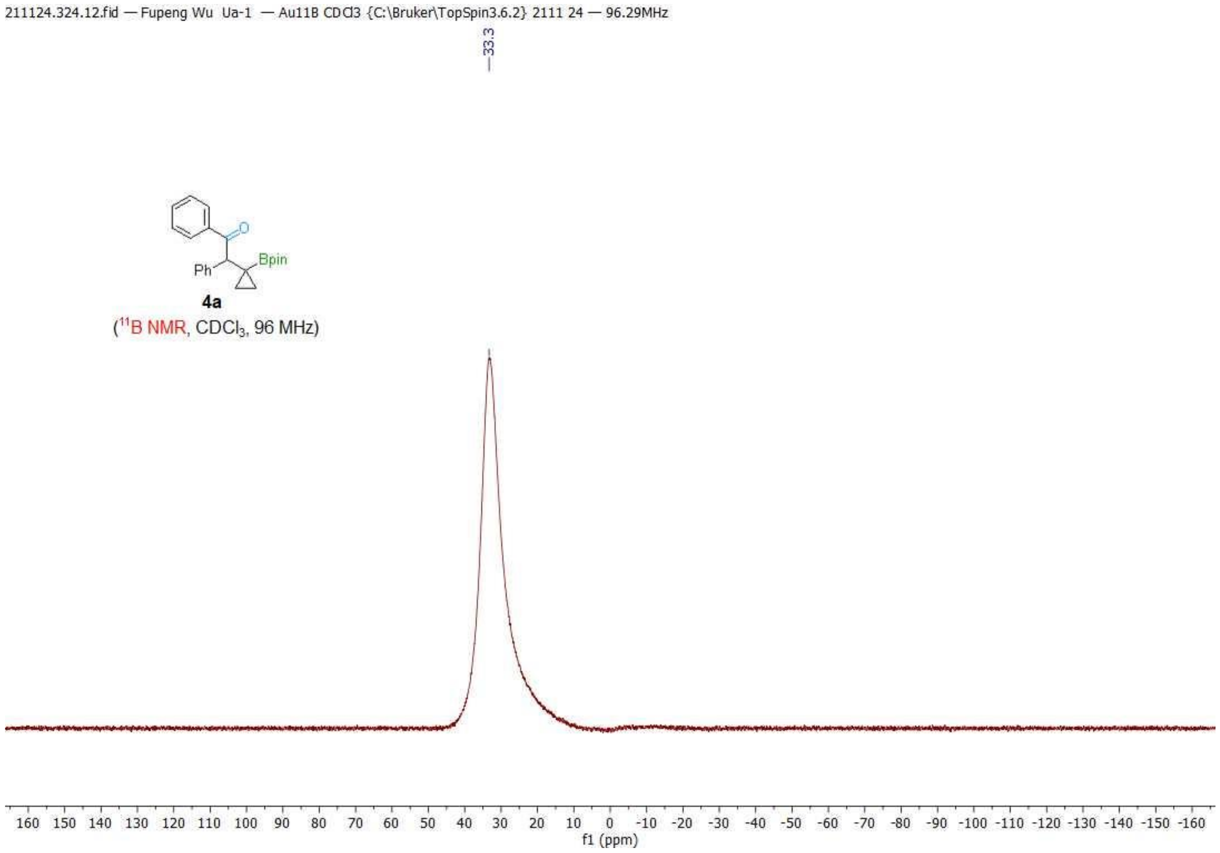

211124.324.12.fid — Fupeng Wu Ua-1 — Au11B CDCl3 {C:\Bruker\TopSpin3.6.2} 2111 24 — 96.29MHz
33.3
Ph
Bpin
O
4a
(11B NMR, CDCl3, 96 MHz)
160 150 140 130 120 110 100 90 80 70 60 50 40 30 20 10 0 -10 -20 -30 -40 -50 -60 -70 -80 -90 -100 -110 -120 -130 -140 -150 -160
f1 (ppm)

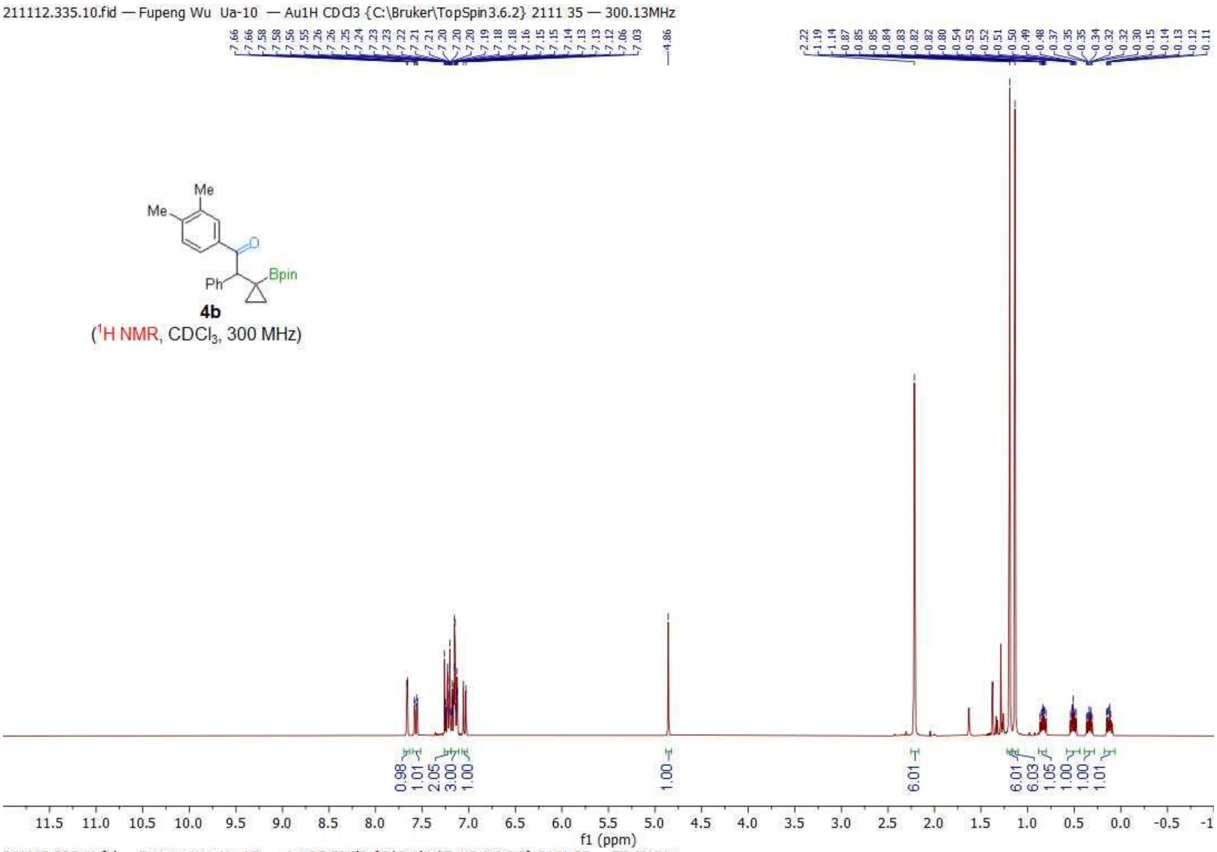
211112.335.10.fid — Fupeng Wu Ua-10 — Au1H CDCl3 {C:\Bruker\TopSpin3.6.2} 2111 35 — 300.13MHz
7.66 7.58 7.56 7.55 7.26 7.25 7.24 7.23 7.23 7.22 7.21 7.21 7.20 7.20 7.19 7.18 7.18 7.16 7.15 7.15 7.14 7.13 7.13 7.12 7.06 7.03
4.86
2.22 1.19 1.14 0.87 0.85 0.85 0.84 0.83 0.82 0.82 0.80 0.54 0.53 0.52 0.51 0.50 0.49 0.48 0.37 0.35 0.35 0.34 0.32 0.32 0.30 0.15 0.14 0.13 0.12 0.11
Me
Me
O
Ph
Bpin
4b
(1H NMR, CDCl3, 300 MHz)
0.98 1.01 2.05 3.00 1.00
1.00
6.01
6.01 6.03 1.05 1.00 1.00 1.01
f1 (ppm)

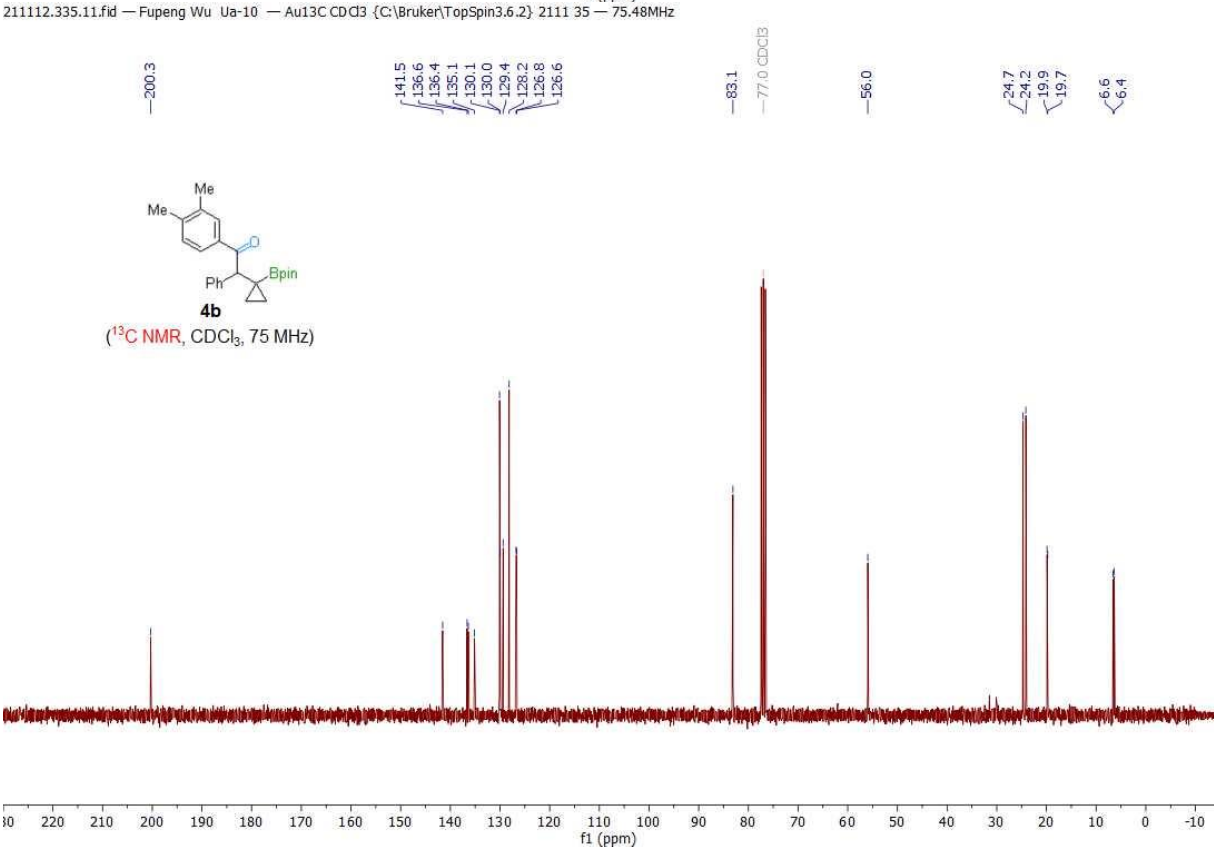
211112.335.11.fid — Fupeng Wu Ua-10 — Au13C CDCl3 {C:\Bruker\TopSpin3.6.2} 2111 35 — 75.48MHz
200.3
141.5 136.6 136.4 135.1 130.1 130.0 129.4 128.2 126.8 126.6
83.1
77.0 CDCl3
56.0
24.7 24.2 19.9 19.7
6.6 6.4
Me
Me
O
Ph
Bpin
4b
(13C NMR, CDCl3, 75 MHz)
f1 (ppm)

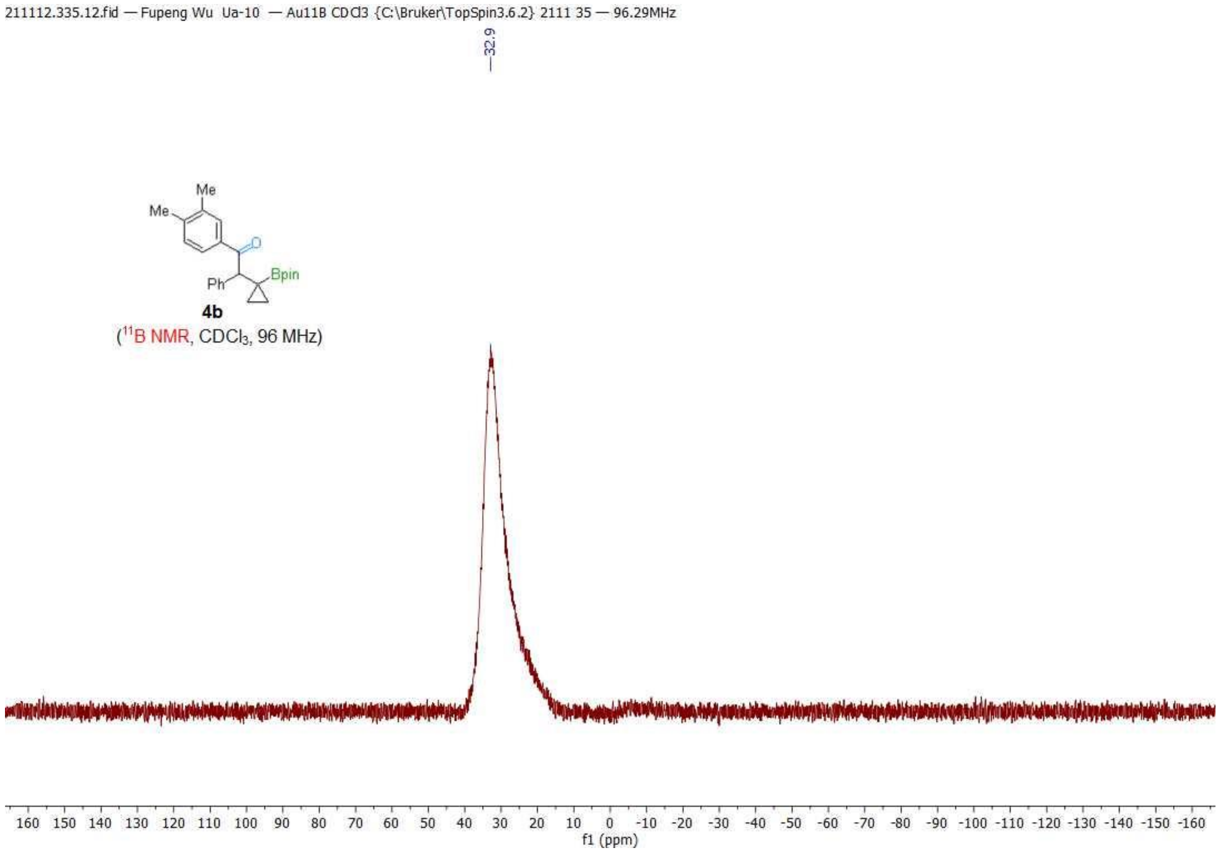
211112.335.12.fid — Fupeng Wu Ua-10 — Au11B CDCl3 {C:\Bruker\TopSpin3.6.2} 2111 35 — 96.29MHz
32.9
Me
Me
O
Bpin
Ph
4b
(11B NMR, CDCl3, 96 MHz)
160 150 140 130 120 110 100 90 80 70 60 50 40 30 20 10 0 -10 -20 -30 -40 -50 -60 -70 -80 -90 -100 -110 -120 -130 -140 -150 -160
f1 (ppm)

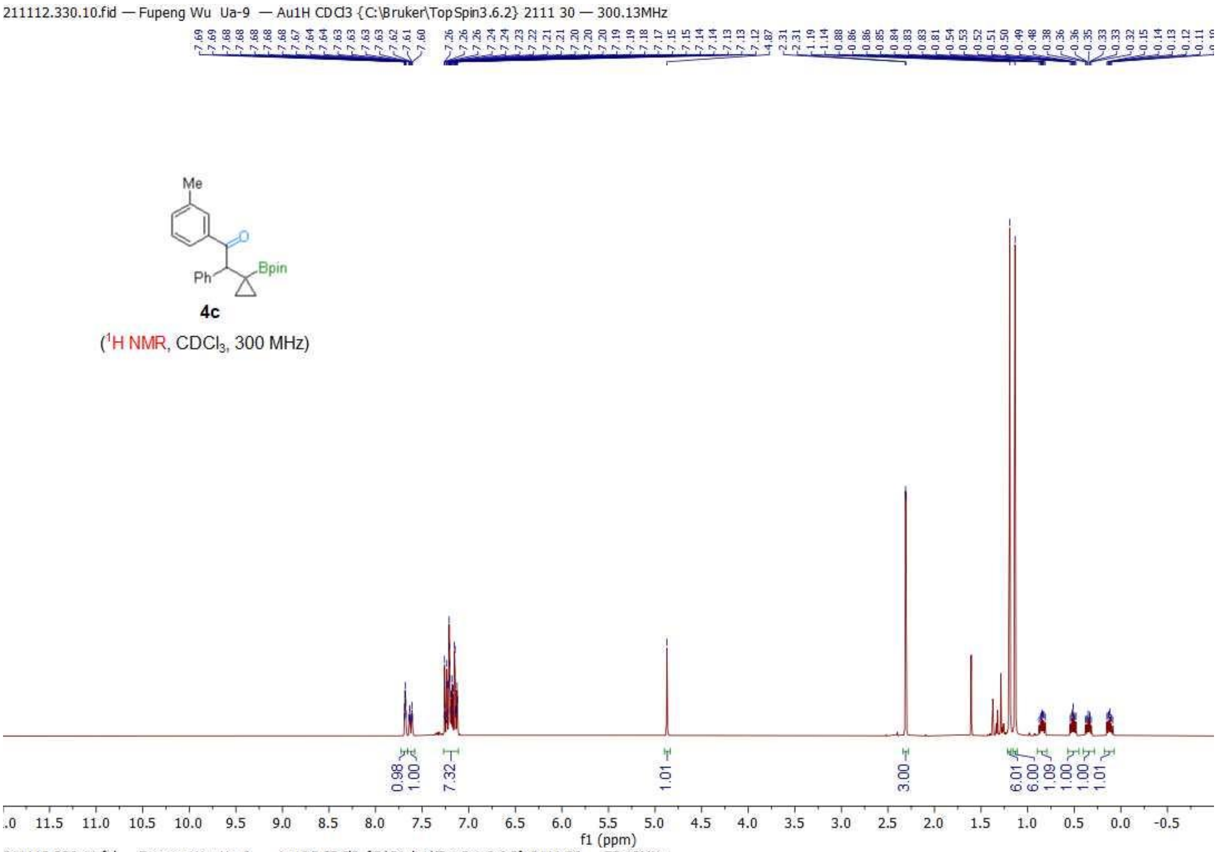
211112.330.10.fid — Fupeng Wu Ua-9 — Au1H CDCl3 {C:\Bruker\TopSpin3.6.2} 2111 30 — 300.13MHz
Me
O
Ph
Bpin
4c
(^{1}H NMR, CDCl$_3$, 300 MHz)
0.98
1.00
7.32
1.01
3.00
6.01
6.00
1.09
1.00
1.00
1.01
f1 (ppm)

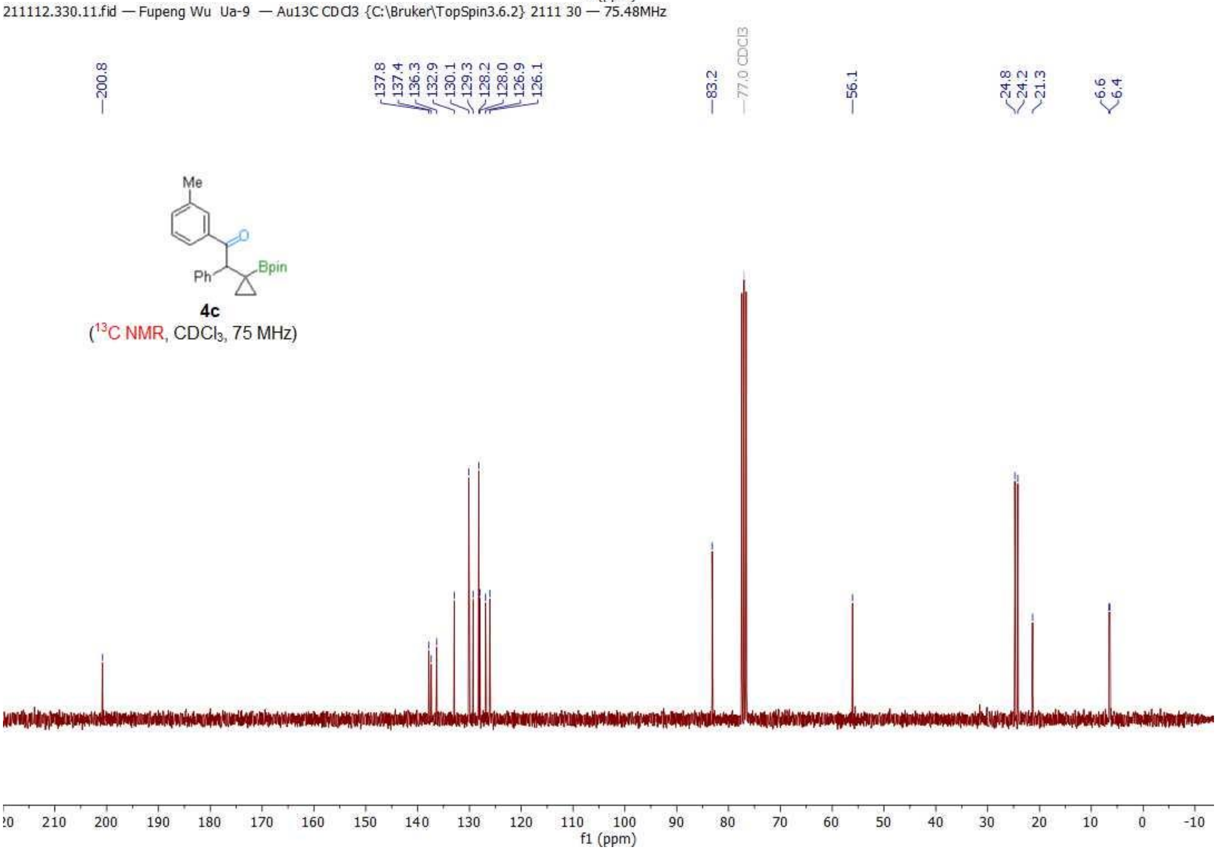
211112.330.11.fid — Fupeng Wu Ua-9 — Au13C CDCl3 {C:\Bruker\TopSpin3.6.2} 2111 30 — 75.48MHz
200.8
137.8
137.4
136.3
132.9
130.1
129.3
128.2
128.0
126.9
126.1
83.2
77.0 CDCl3
56.1
24.8
24.2
21.3
6.6
6.4
Me
O
Ph
Bpin
4c
(^{13}C NMR, CDCl$_3$, 75 MHz)
f1 (ppm)

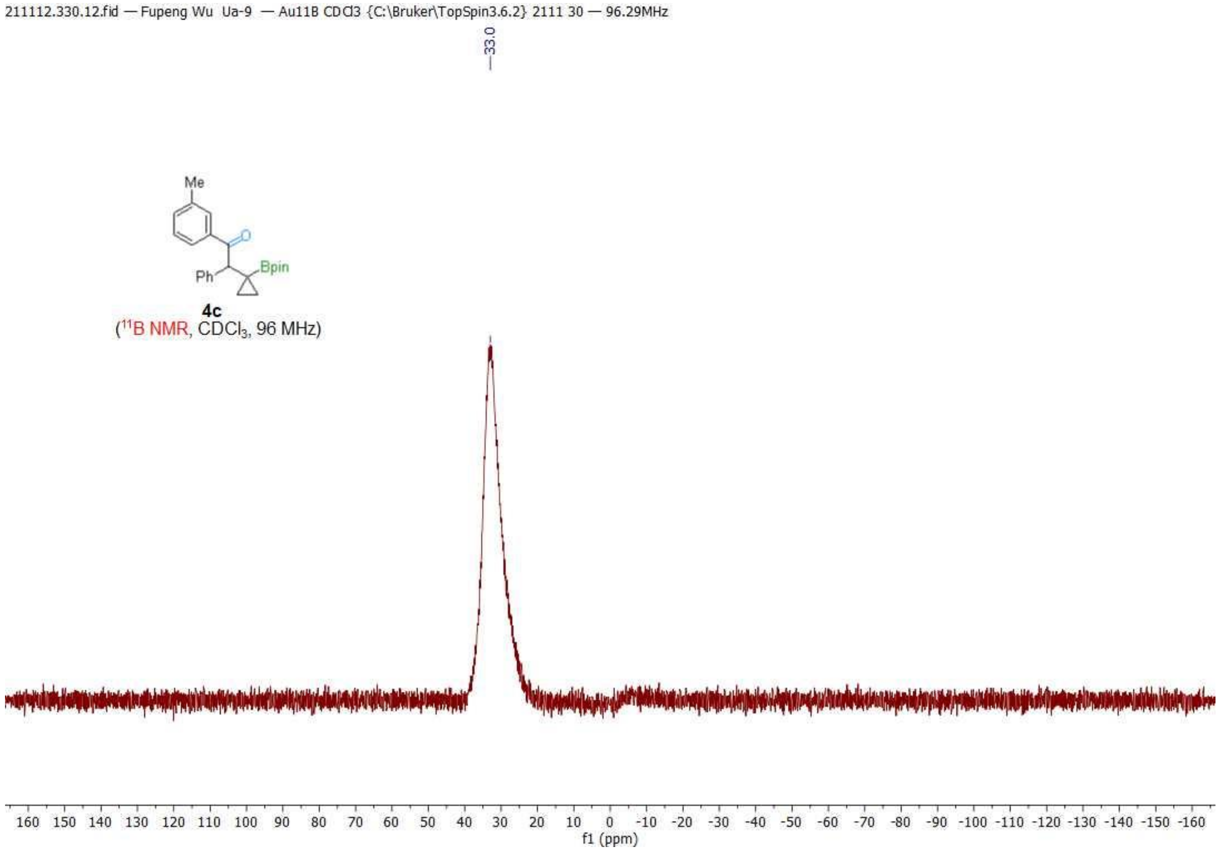
211112.330.12.fid — Fupeng Wu Ua-9 — Au11B CDCl3 {C:\Bruker\TopSpin3.6.2} 2111 30 — 96.29MHz
33.0
Me
O
Bpin
Ph
4c
(11B NMR, CDCl3, 96 MHz)
160 150 140 130 120 110 100 90 80 70 60 50 40 30 20 10 0 -10 -20 -30 -40 -50 -60 -70 -80 -90 -100 -110 -120 -130 -140 -150 -160
f1 (ppm)

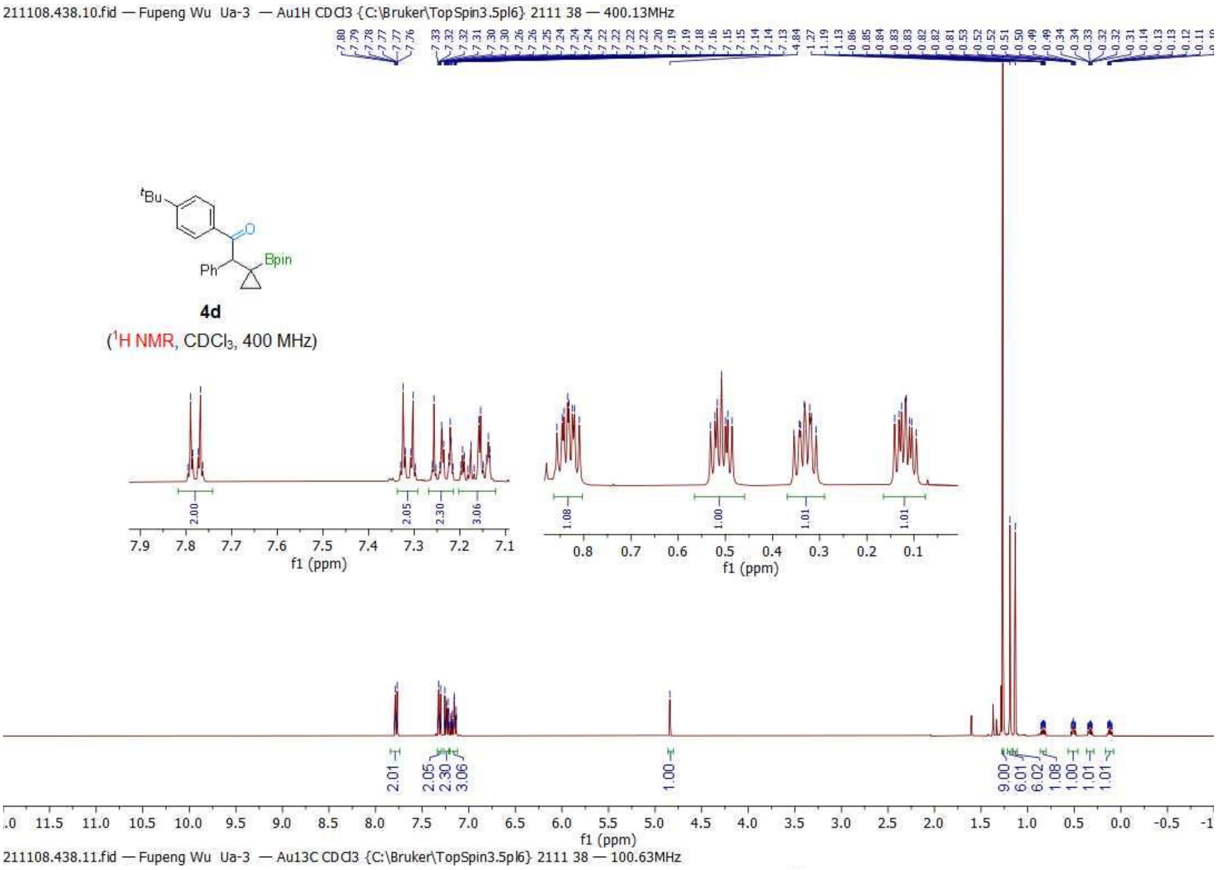
211108.438.10.fid — Fupeng Wu Ua-3 — Au1H CDCl3 {C:\Bruker\TopSpin3.5pl6} 2111 38 — 400.13MHz
tBu
O
Bpin
Ph
4d
(1H NMR, CDCl3, 400 MHz)
2.00
2.05
2.30
3.06
1.08
1.00
1.01
1.01
2.01
1.00
9.00
6.01
6.02
f1 (ppm)

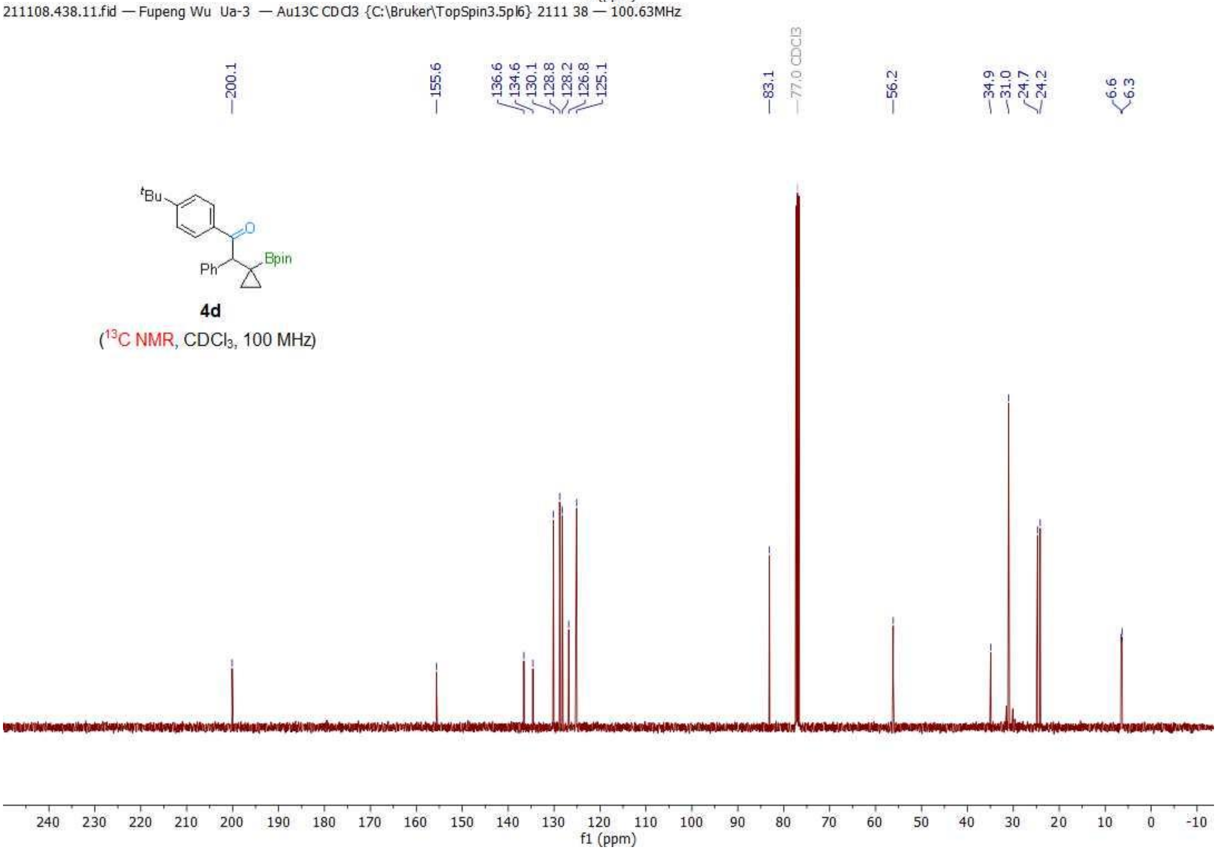
211108.438.11.fid — Fupeng Wu Ua-3 — Au13C CDCl3 {C:\Bruker\TopSpin3.5pl6} 2111 38 — 100.63MHz
200.1
155.6
136.6
134.6
130.1
128.8
128.2
126.8
125.1
83.1
77.0 CDCl3
56.2
34.9
31.0
24.7
24.2
6.6
6.3
tBu
O
Bpin
Ph
4d
(13C NMR, CDCl3, 100 MHz)
f1 (ppm)

211108.438.12.fid — Fupeng Wu Ua-3 — Au11B CDCl3 {C:\Bruker\TopSpin3.5pl6} 2111 38 — 128.38MHz

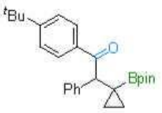

4d

(^{11}B NMR, $CDCl_3$, 128 MHz)

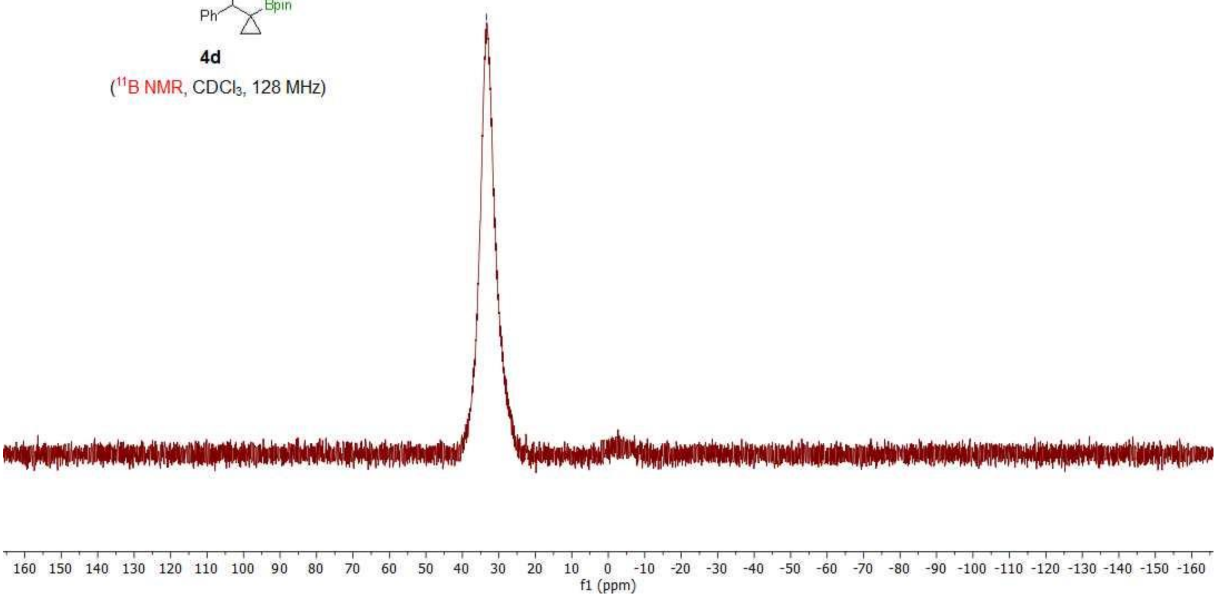

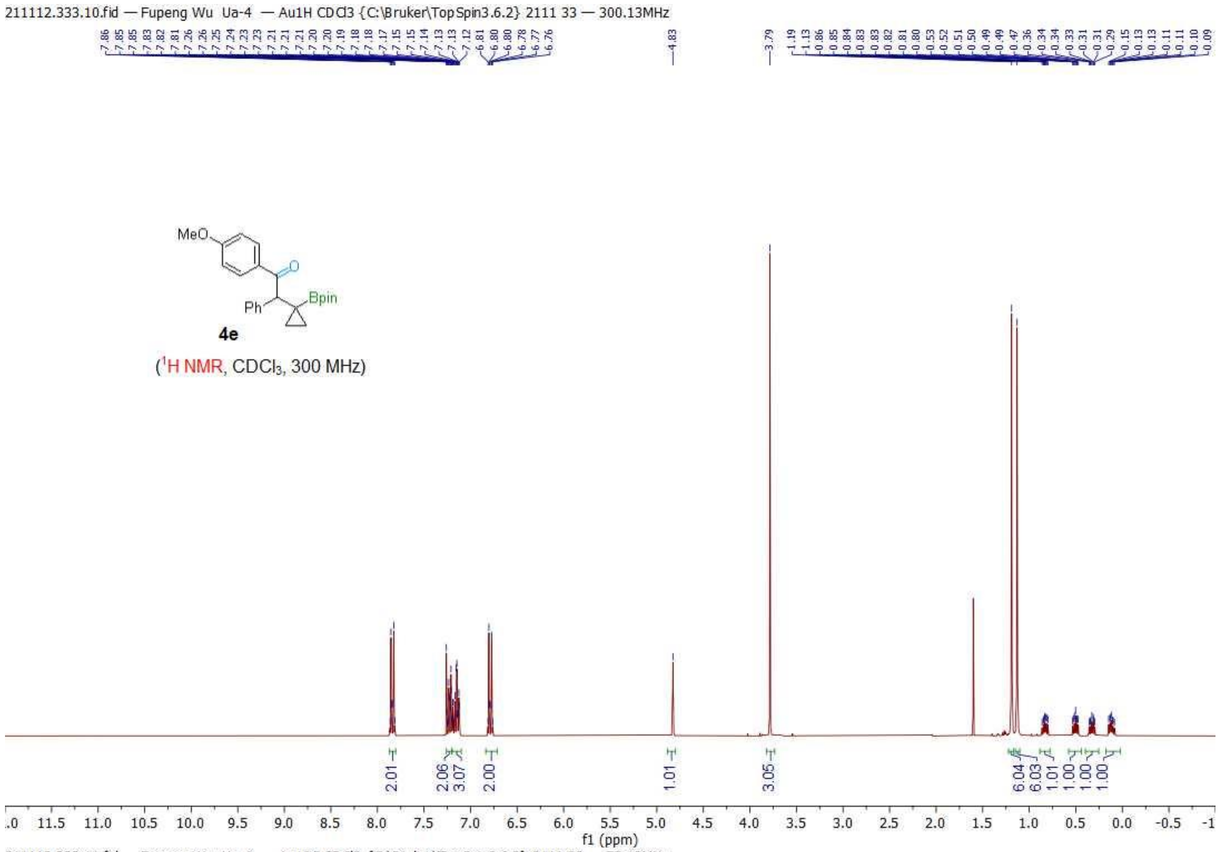
211112.333.10.fid — Fupeng Wu Ua-4 — Au1H CDCl3 {C:\Bruker\TopSpin3.6.2} 2111 33 — 300.13MHz
MeO
O
Ph
Bpin
4e
(1H NMR, CDCl3, 300 MHz)
2.01
2.06
3.07
2.00
1.01
3.05
6.04
6.03
1.01
1.00
1.00
1.00
f1 (ppm)

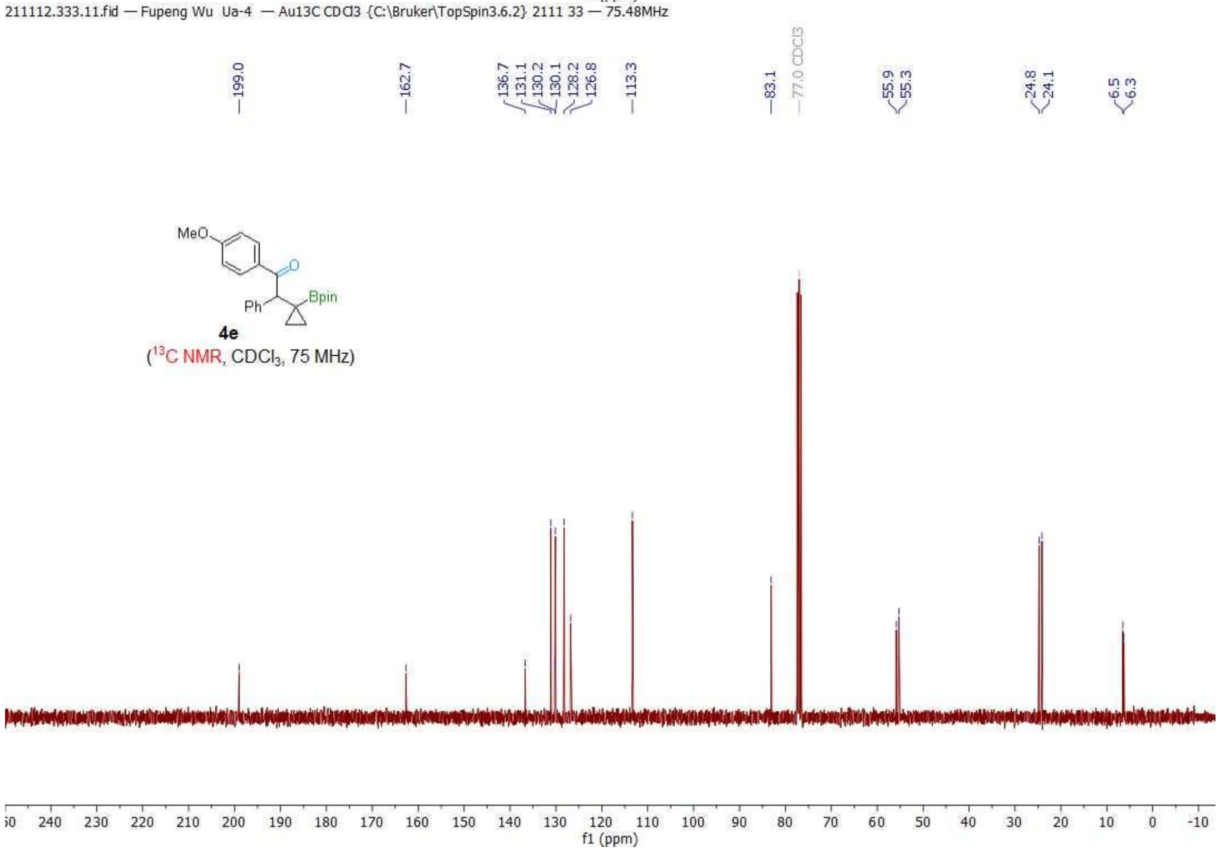
211112.333.11.fid — Fupeng Wu Ua-4 — Au13C CDCl3 {C:\Bruker\TopSpin3.6.2} 2111 33 — 75.48MHz
199.0
162.7
136.7
131.1
130.2
130.1
128.2
126.8
113.3
83.1
77.0 CDCl3
55.9
55.3
24.8
24.1
6.5
6.3
MeO
O
Ph
Bpin
4e
(13C NMR, CDCl3, 75 MHz)
f1 (ppm)

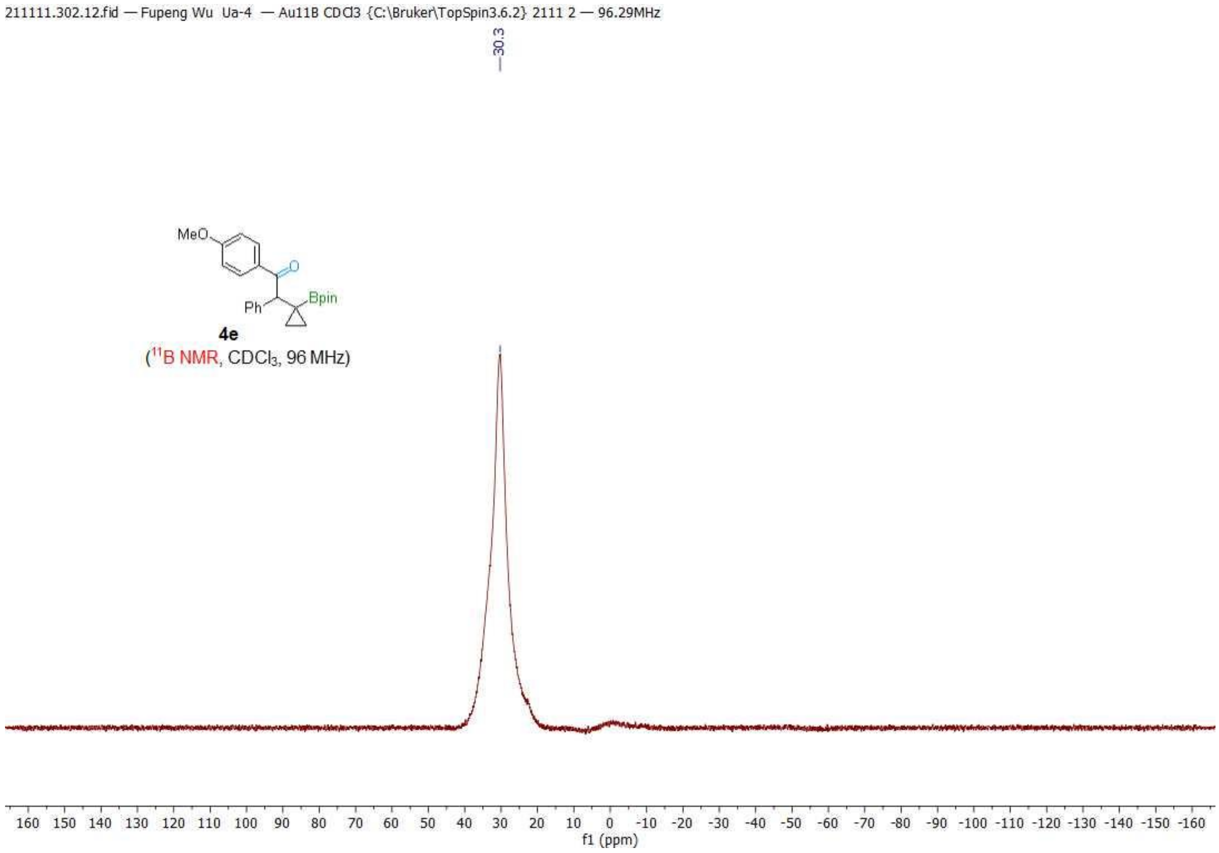
211111.302.12.fid — Fupeng Wu Ua-4 — Au11B CDCl3 {C:\Bruker\TopSpin3.6.2} 2111 2 — 96.29MHz
30.3
MeO
O
Bpin
Ph
4e
(11B NMR, CDCl3, 96 MHz)
160 150 140 130 120 110 100 90 80 70 60 50 40 30 20 10 0 -10 -20 -30 -40 -50 -60 -70 -80 -90 -100 -110 -120 -130 -140 -150 -160
f1 (ppm)

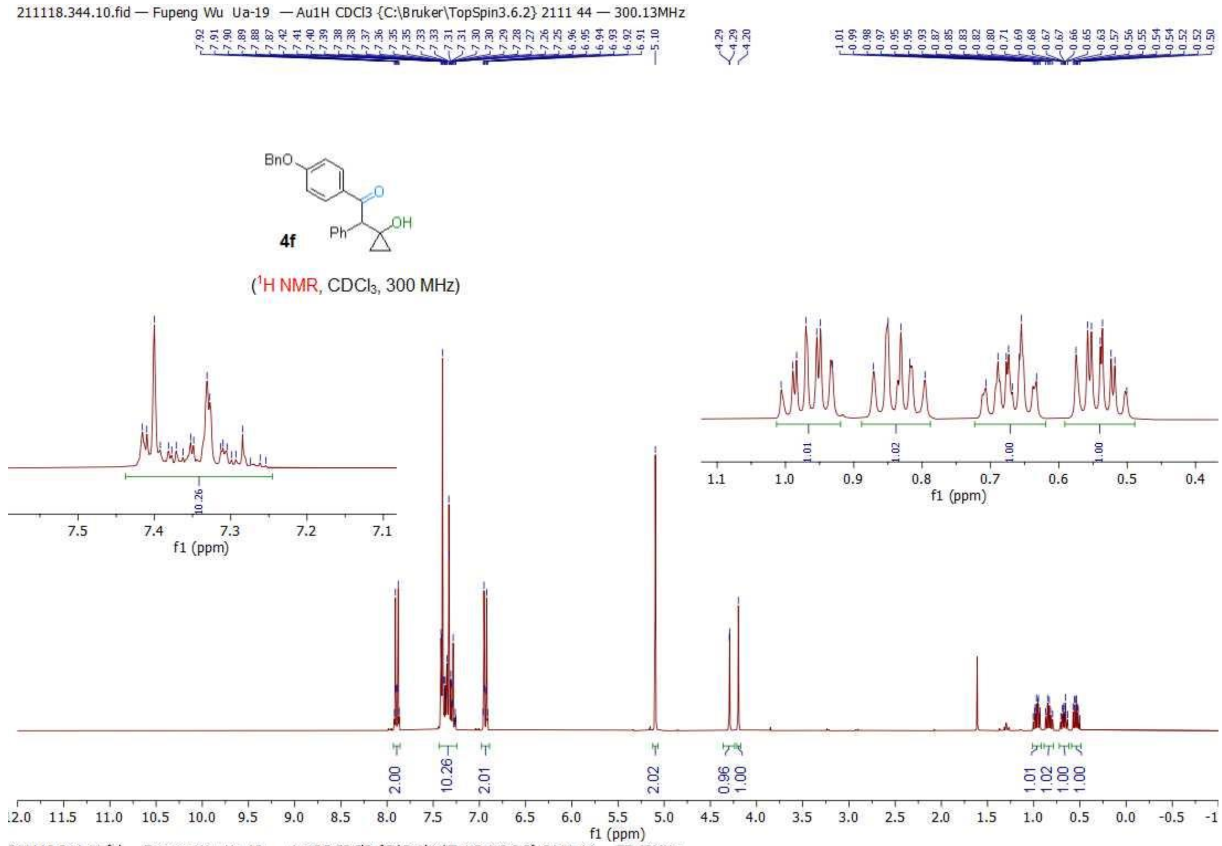
211118.344.10.fid — Fupeng Wu Ua-19 — Au1H CDCl3 {C:\Bruker\TopSpin3.6.2} 2111 44 — 300.13MHz
BnO
O
OH
Ph
4f
(1H NMR, CDCl3, 300 MHz)
10.26
1.01
1.02
1.00
1.00
2.00
10.26
2.01
2.02
0.96
1.00
f1 (ppm)

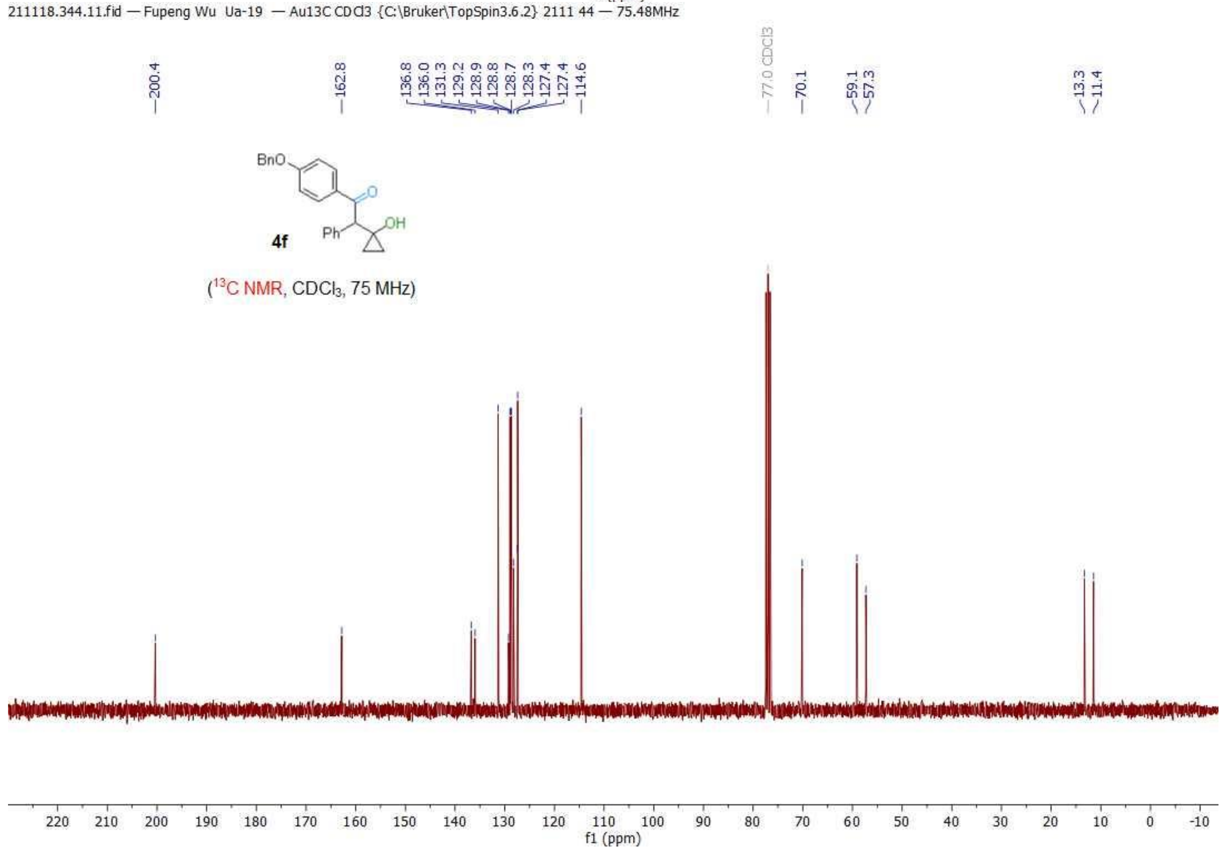
211118.344.11.fid — Fupeng Wu Ua-19 — Au13C CDCl3 {C:\Bruker\TopSpin3.6.2} 2111 44 — 75.48MHz
200.4
162.8
136.8
136.0
131.3
129.2
128.9
128.8
128.7
128.3
127.4
127.4
114.6
77.0 CDCl3
70.1
59.1
57.3
13.3
11.4
BnO
O
OH
Ph
4f
(13C NMR, CDCl3, 75 MHz)
f1 (ppm)

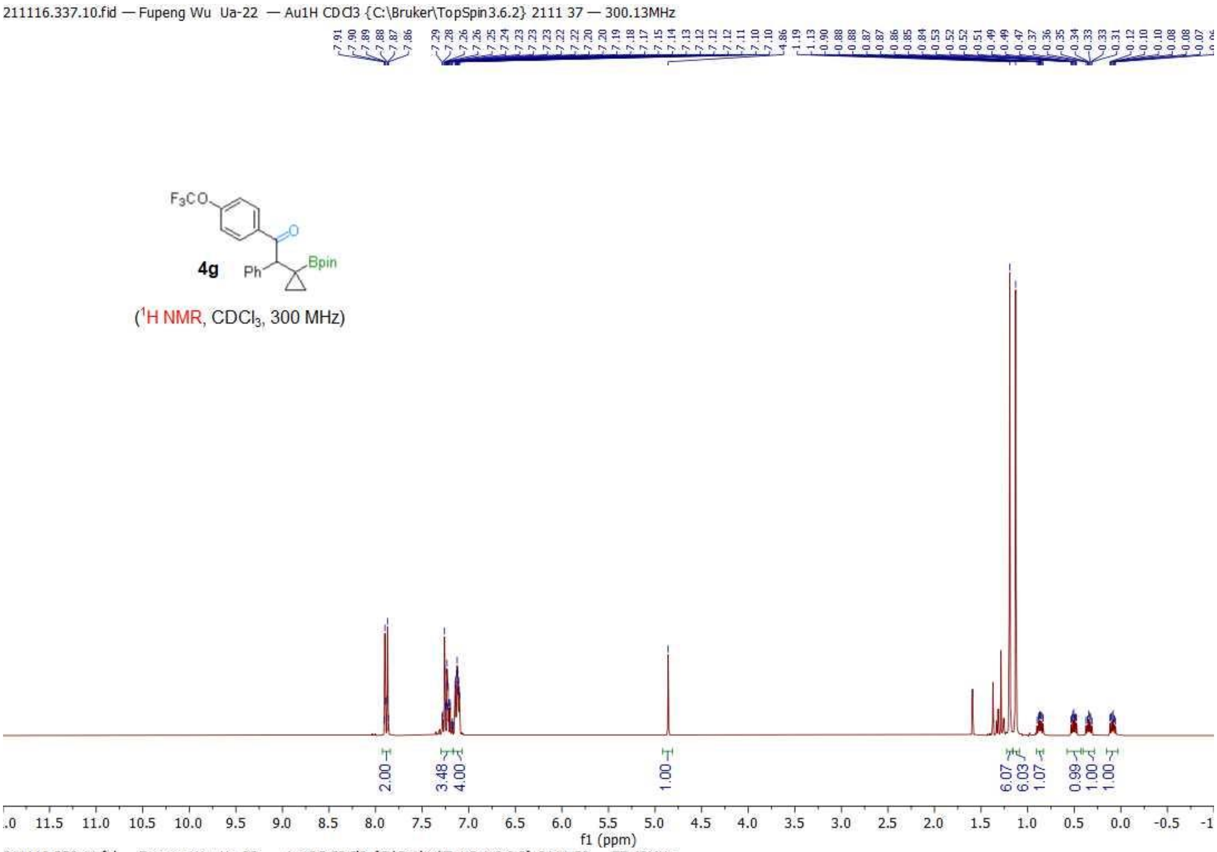
211116.337.10.fid — Fupeng Wu Ua-22 — Au1H CDCl3 {C:\Bruker\TopSpin3.6.2} 2111 37 — 300.13MHz
F3CO
O
Bpin
4g
Ph
(1H NMR, CDCl3, 300 MHz)
2.00
3.48
4.00
1.00
6.07
6.03
1.07
0.99
1.00
1.00
f1 (ppm)

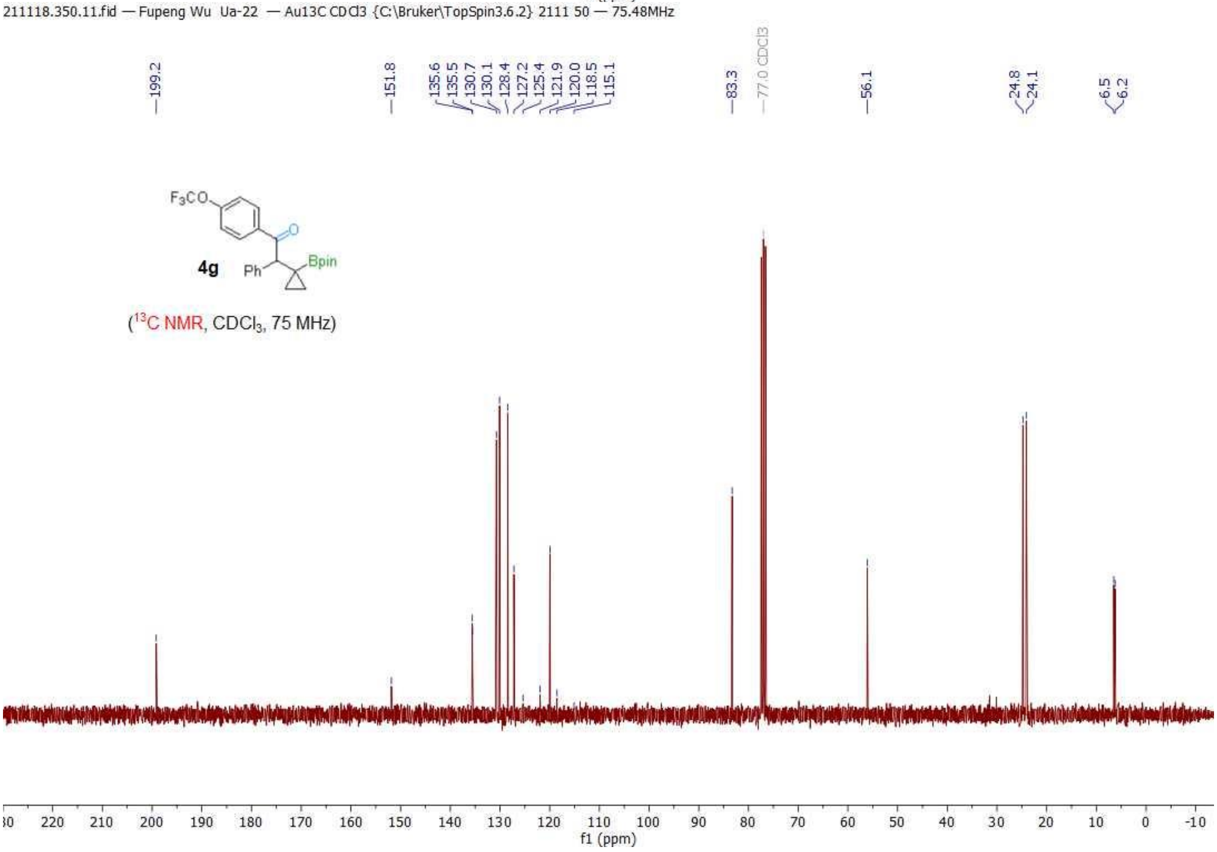
211118.350.11.fid — Fupeng Wu Ua-22 — Au13C CDCl3 {C:\Bruker\TopSpin3.6.2} 2111 50 — 75.48MHz
199.2
151.8
135.6
135.5
130.7
130.1
128.4
127.2
125.4
121.9
120.0
118.5
115.1
83.3
77.0 CDCl3
56.1
24.8
24.1
6.5
6.2
F3CO
O
Bpin
4g
Ph
(13C NMR, CDCl3, 75 MHz)
f1 (ppm)

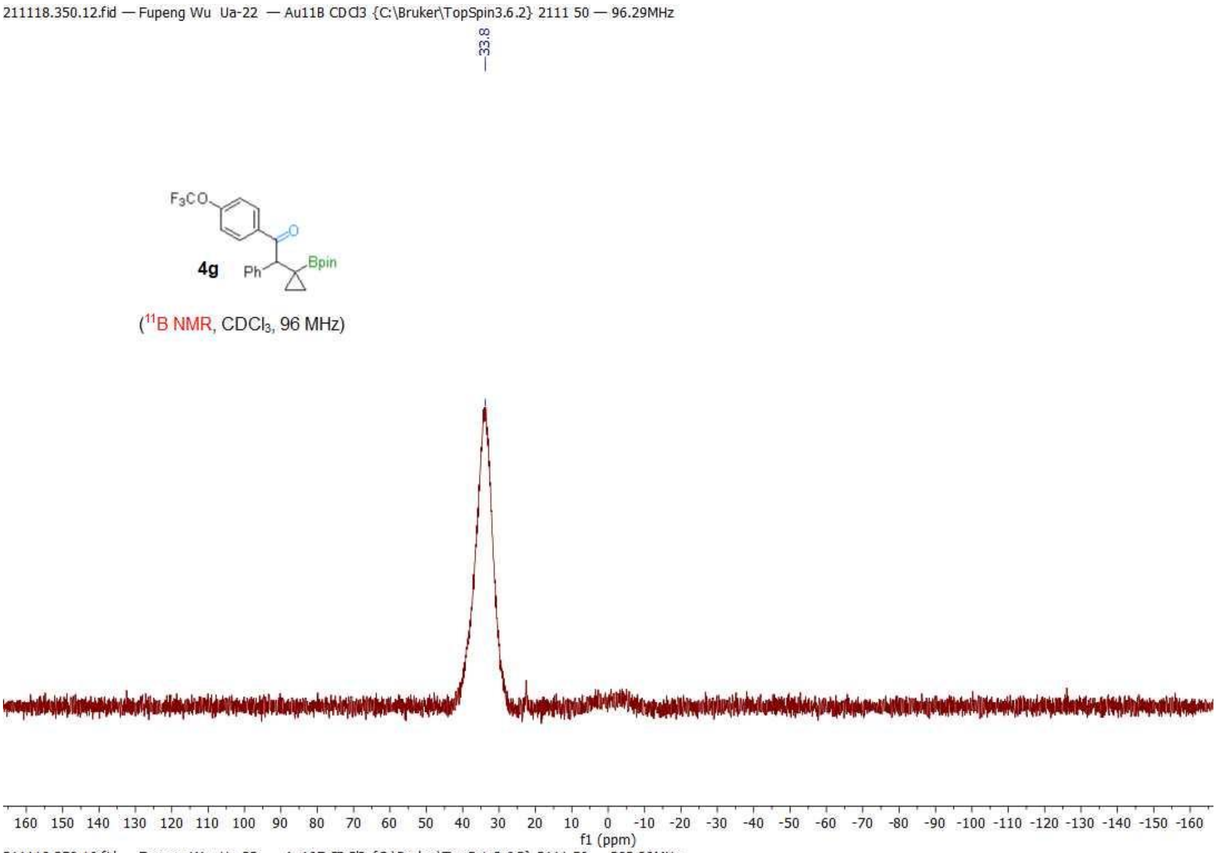
211118.350.12.fid — Fupeng Wu Ua-22 — Au11B CDCl3 {C:\Bruker\TopSpin3.6.2} 2111 50 — 96.29MHz
33.8
F3CO
O
Bpin
4g
Ph
(11B NMR, CDCl3, 96 MHz)
160 150 140 130 120 110 100 90 80 70 60 50 40 30 20 10 0 -10 -20 -30 -40 -50 -60 -70 -80 -90 -100 -110 -120 -130 -140 -150 -160
f1 (ppm)

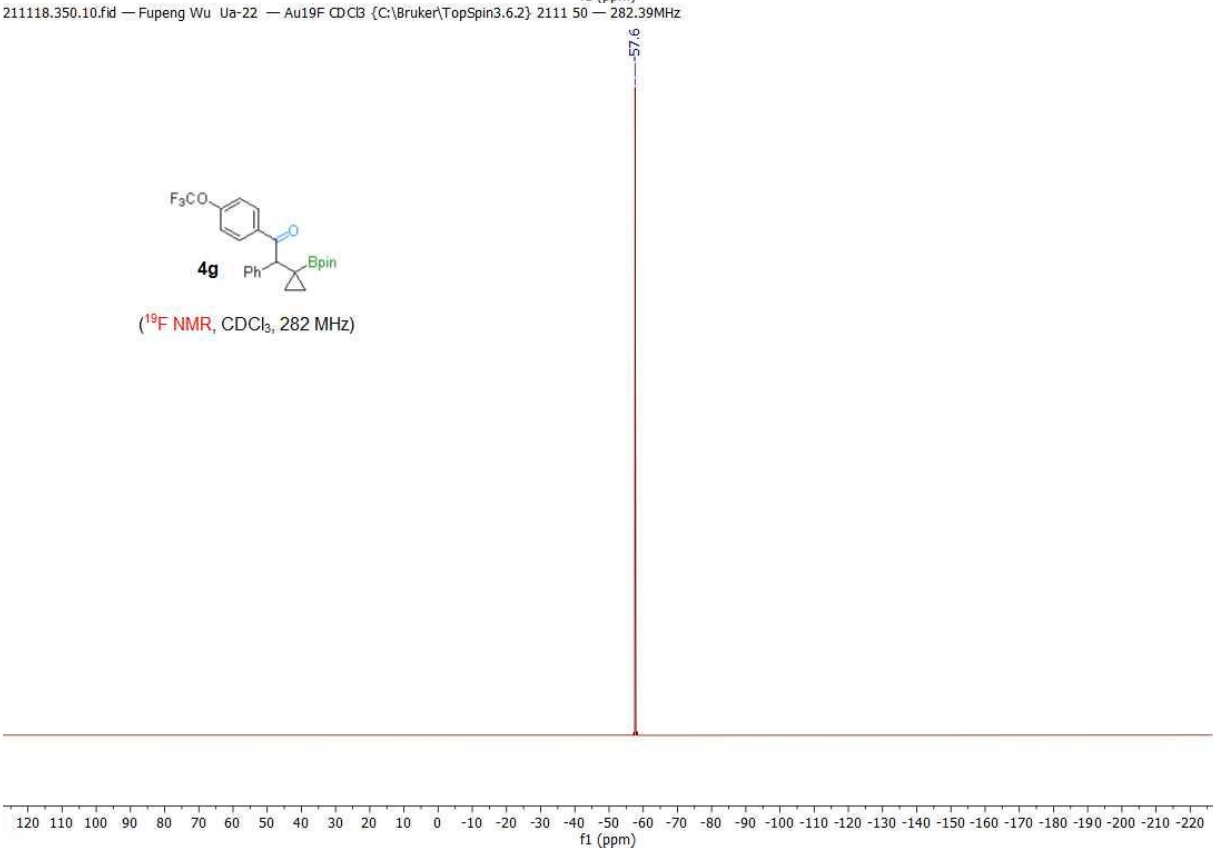
211118.350.10.fid — Fupeng Wu Ua-22 — Au19F CDCl3 {C:\Bruker\TopSpin3.6.2} 2111 50 — 282.39MHz
-57.6
F3CO
O
Bpin
4g
Ph
(19F NMR, CDCl3, 282 MHz)
120 110 100 90 80 70 60 50 40 30 20 10 0 -10 -20 -30 -40 -50 -60 -70 -80 -90 -100 -110 -120 -130 -140 -150 -160 -170 -180 -190 -200 -210 -220
f1 (ppm)

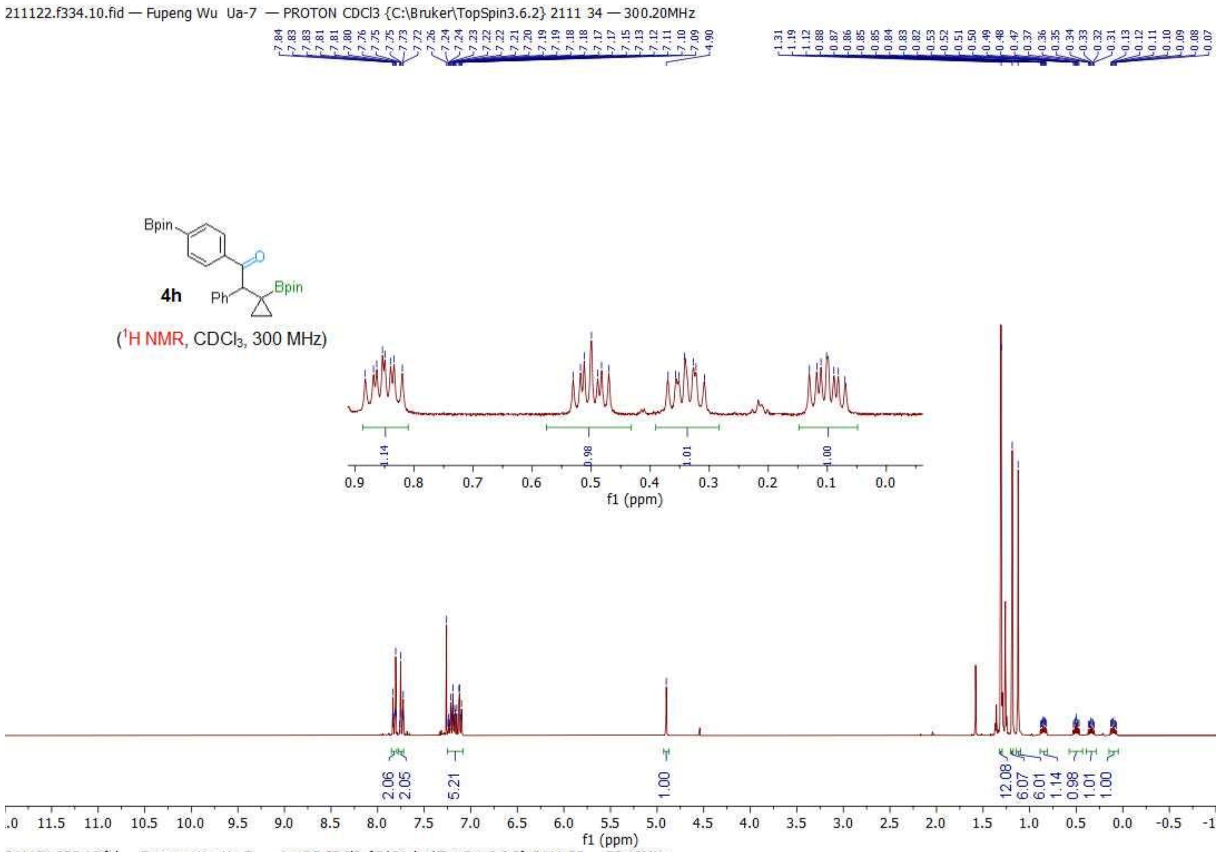
211122.f334.10.fid — Fupeng Wu Ua-7 — PROTON CDCl3 {C:\Bruker\TopSpin3.6.2} 2111 34 — 300.20MHz
Bpin
O
Bpin
4h
Ph
(1H NMR, CDCl3, 300 MHz)
1.14
0.98
1.01
1.00
2.06
2.05
5.21
1.00
12.08
6.07
6.01
1.14
0.98
1.01
1.00
f1 (ppm)

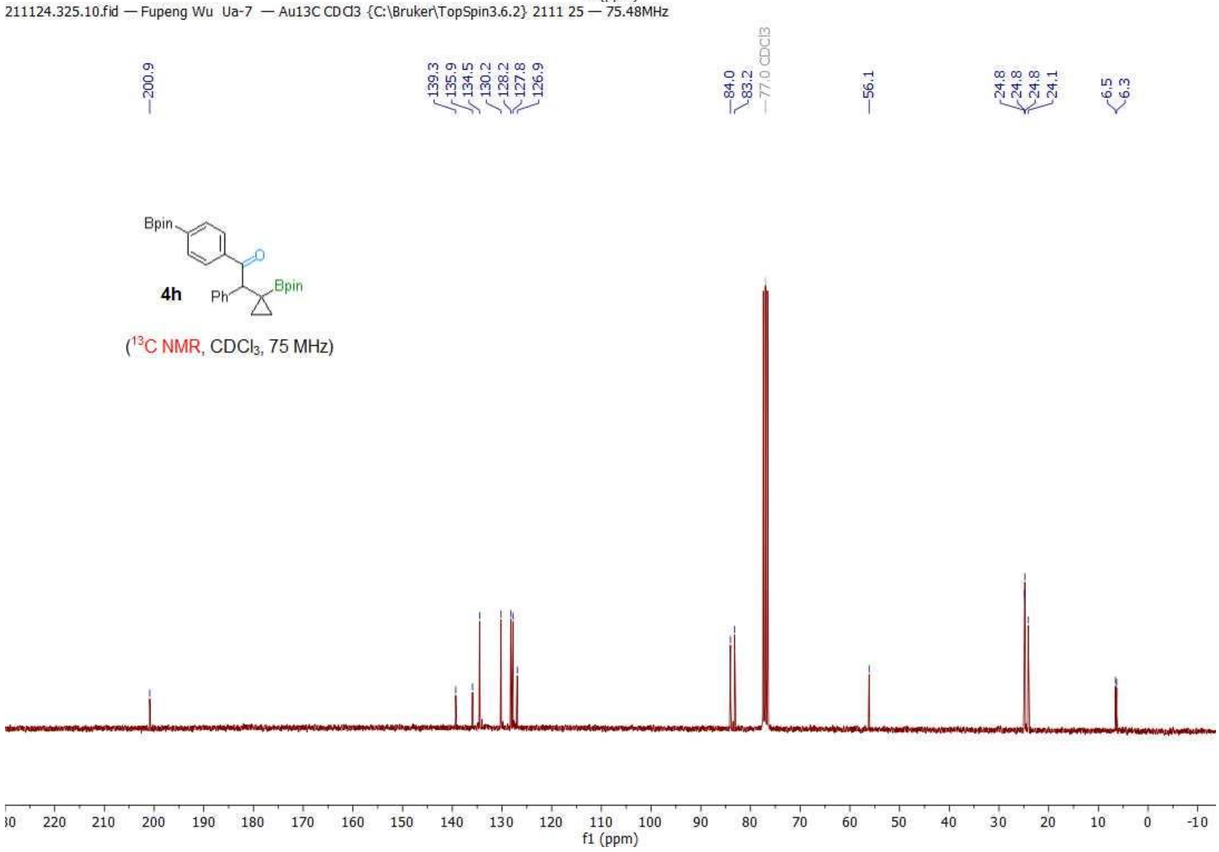
211124.325.10.fid — Fupeng Wu Ua-7 — Au13C CDCl3 {C:\Bruker\TopSpin3.6.2} 2111 25 — 75.48MHz
200.9
139.3
135.9
134.5
130.2
128.2
127.8
126.9
84.0
83.2
77.0 CDCl3
56.1
24.8
24.8
24.8
24.1
6.5
6.3
Bpin
O
Bpin
4h
Ph
(13C NMR, CDCl3, 75 MHz)
f1 (ppm)

211119.349.12.fid — Fupeng Wu Ua-7 — Au11B CDCl3 {C:\Bruker\TopSpin3.6.2} 2111 49 — 96.29MHz

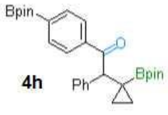

4h

(^{11}B NMR, $CDCl_3$, 96 MHz)

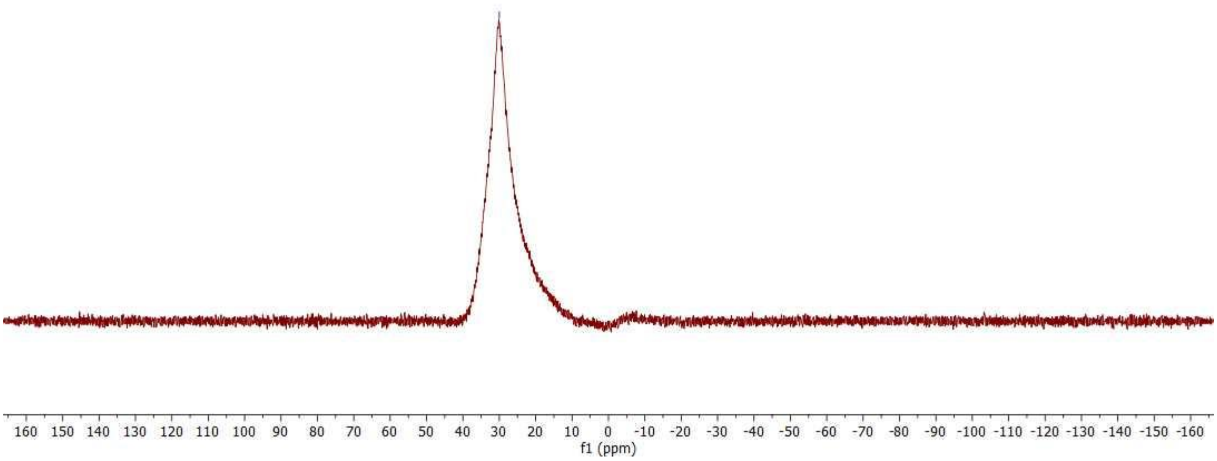

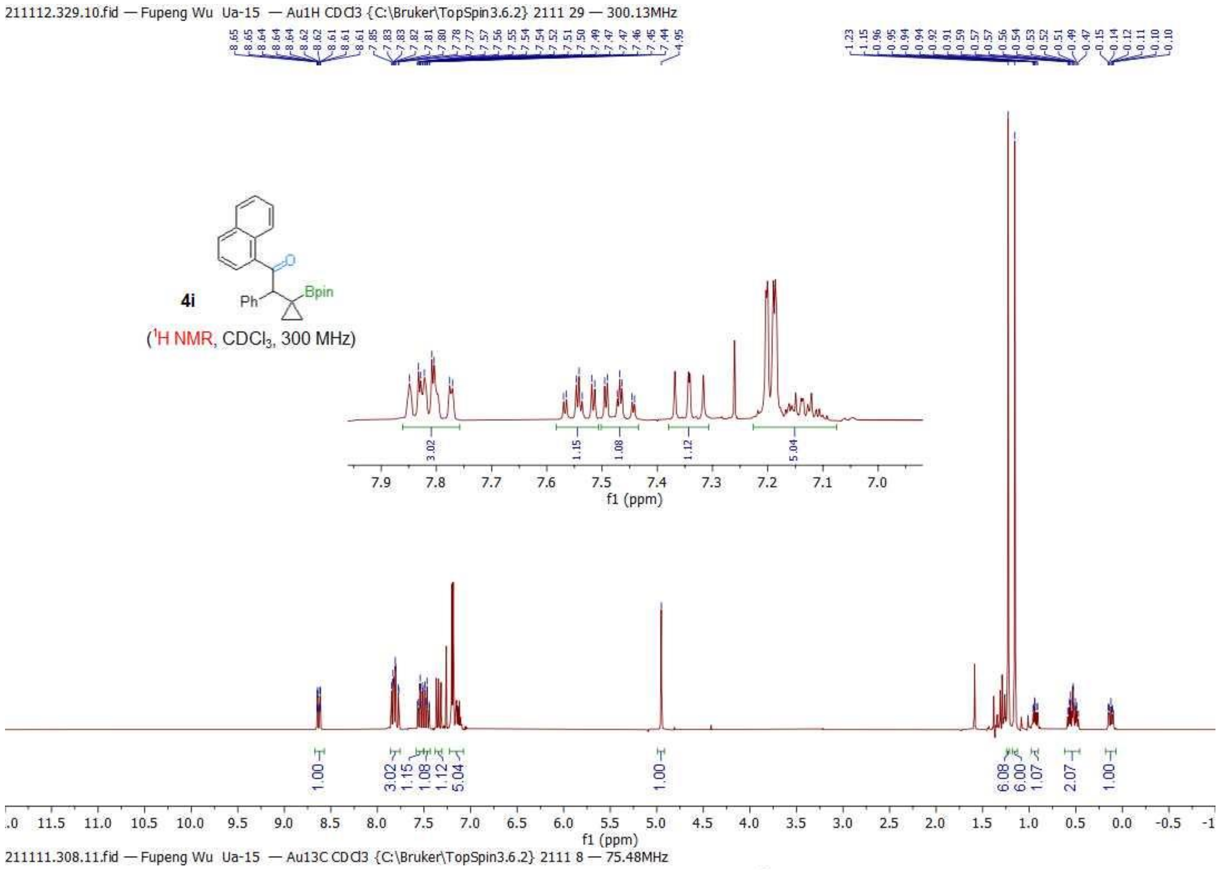
211112.329.10.fid — Fupeng Wu Ua-15 — Au1H CDCl3 {C:\Bruker\TopSpin3.6.2} 2111 29 — 300.13MHz
8.65 8.65 8.64 8.64 8.62 8.62 8.61 8.61 8.61 7.85 7.83 7.83 7.82 7.81 7.80 7.78 7.77 7.57 7.56 7.55 7.54 7.54 7.52 7.51 7.50 7.49 7.47 7.47 7.46 7.45 7.44 4.95
1.23 1.15 0.96 0.95 0.94 0.94 0.92 0.91 0.59 0.57 0.57 0.56 0.54 0.53 0.52 0.51 0.49 0.47 0.15 0.14 0.12 0.11 0.10 0.10
4i
Ph
Bpin
(1H NMR, CDCl3, 300 MHz)
3.02 1.15 1.08 1.12 5.04
1.00 3.02 1.15 1.08 1.12 5.04 1.00 6.08 6.00 1.07 2.07 1.00
f1 (ppm)

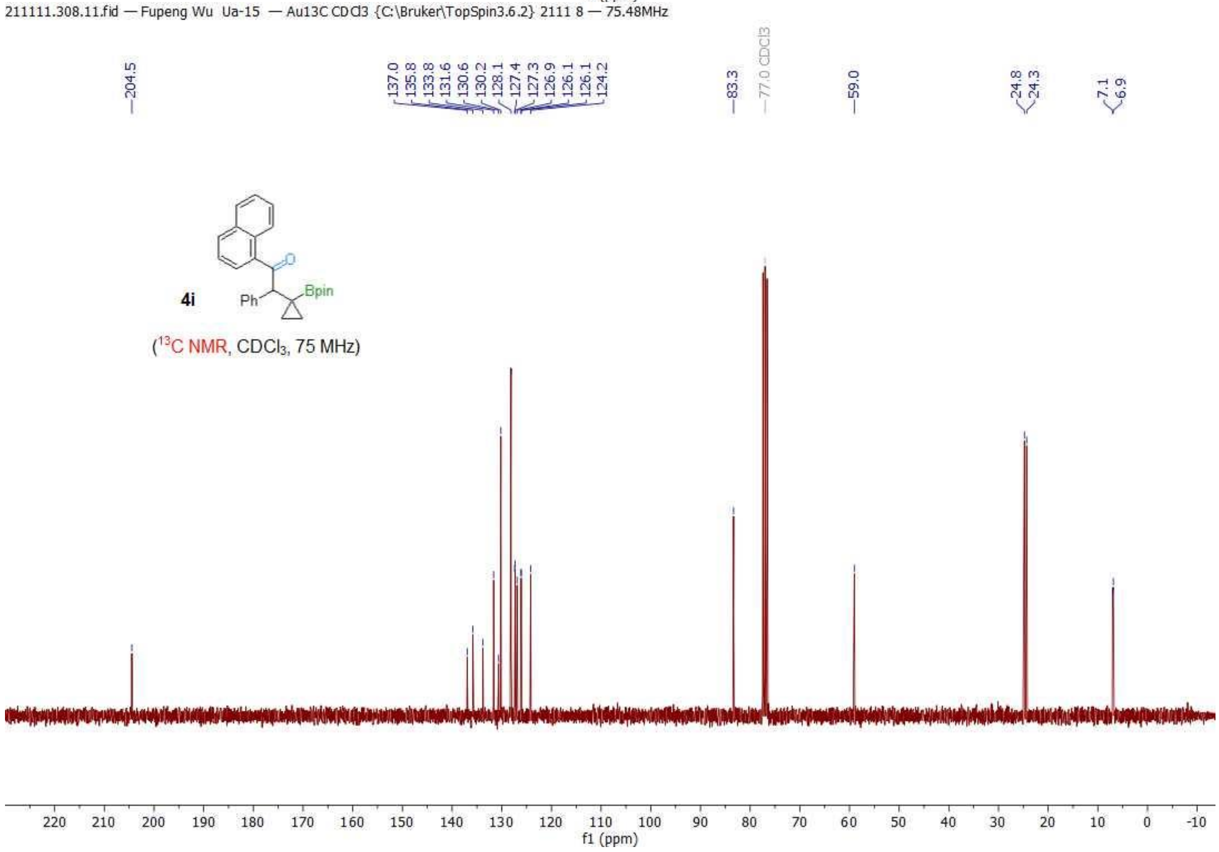
211111.308.11.fid — Fupeng Wu Ua-15 — Au13C CDCl3 {C:\Bruker\TopSpin3.6.2} 2111 8 — 75.48MHz
204.5
137.0 135.8 133.8 131.6 130.6 130.2 128.1 127.4 127.3 126.9 126.1 126.1 124.2
83.3
77.0 CDCl3
59.0
24.8 24.3
7.1 6.9
4i
Ph
Bpin
(13C NMR, CDCl3, 75 MHz)
f1 (ppm)

211111.308.12.fid — Fupeng Wu Ua-15 — Au11B CDCl3 {C:\Bruker\TopSpin3.6.2} 2111 8 — 96.29MHz

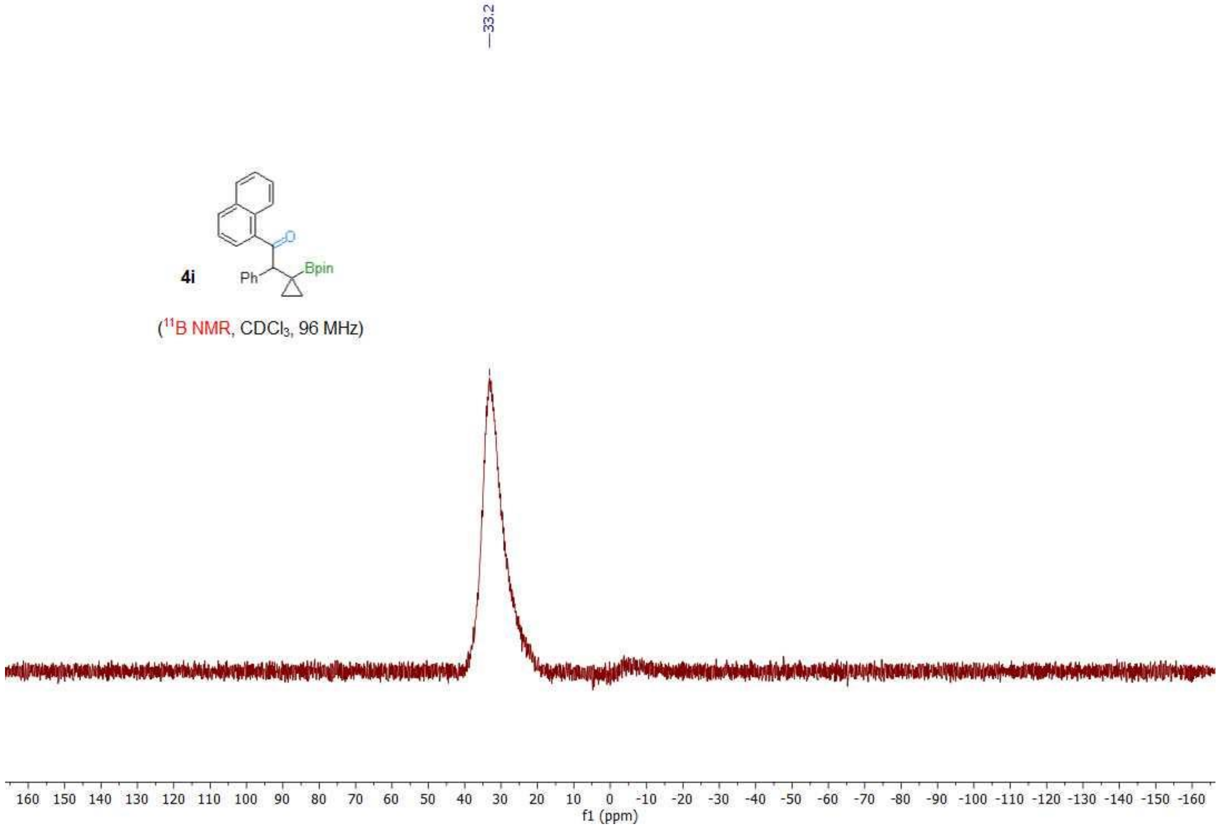

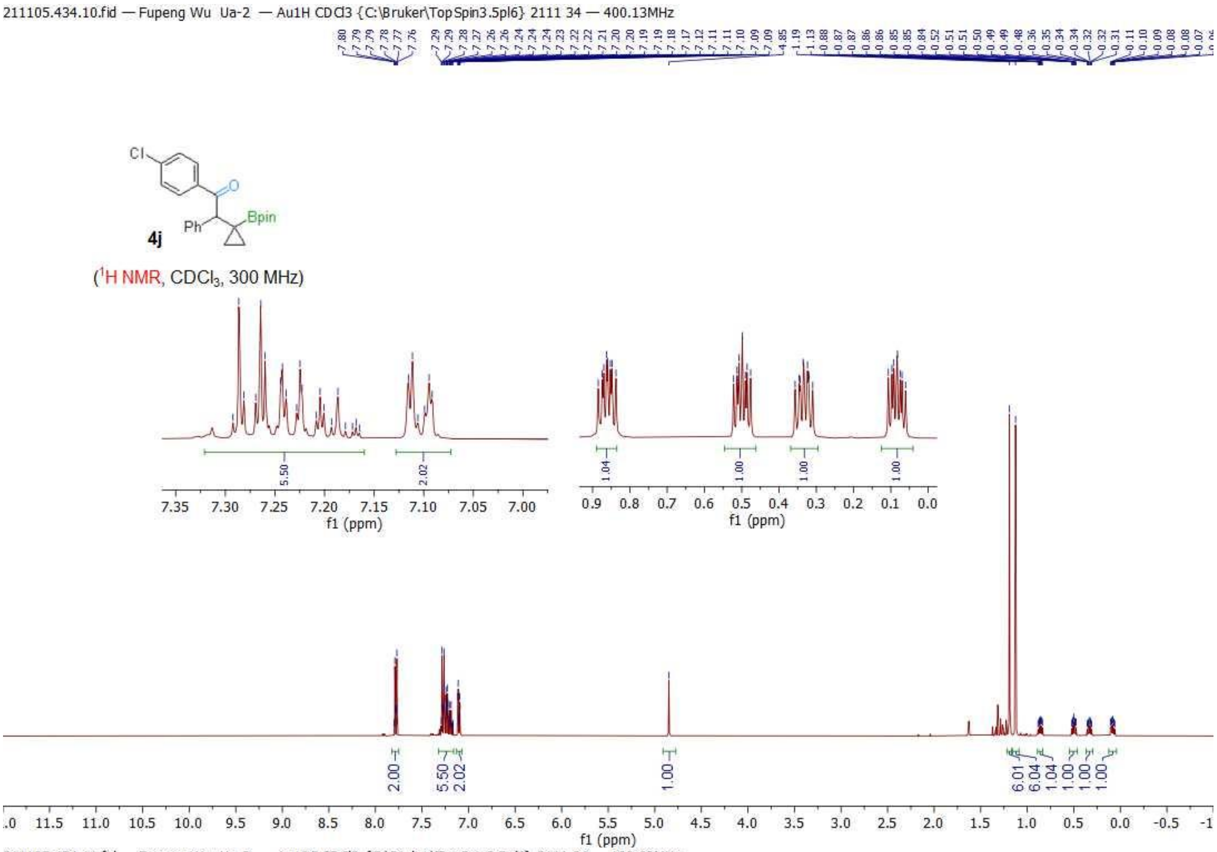
211105.434.10.fid — Fupeng Wu Ua-2 — Au1H CDCl3 {C:\Bruker\TopSpin3.5pl6} 2111 34 — 400.13MHz
Cl
O
Bpin
Ph
4j
(1H NMR, CDCl3, 300 MHz)
5.50
2.02
1.04
1.00
1.00
1.00
f1 (ppm)
2.00
5.50
2.02
1.00
6.01
6.04
1.04
1.00
1.00
1.00
f1 (ppm)

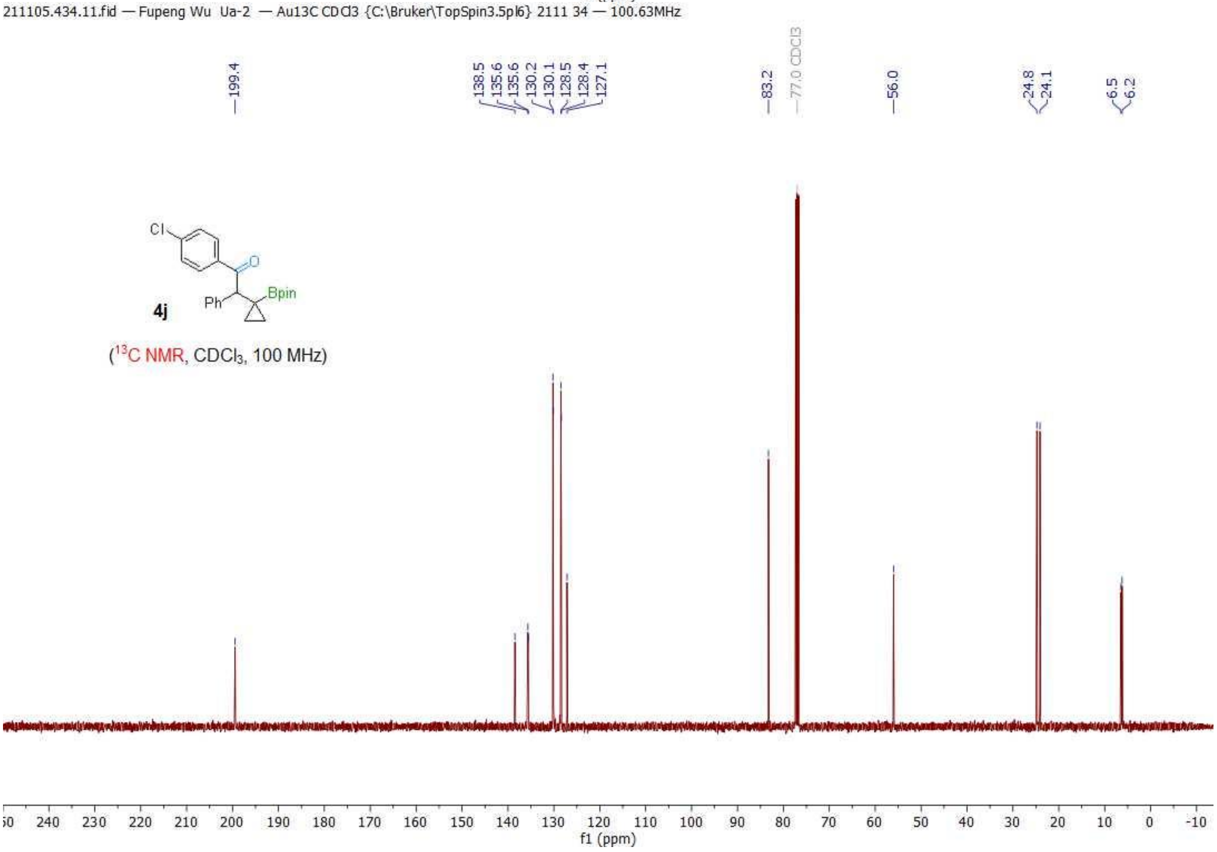
211105.434.11.fid — Fupeng Wu Ua-2 — Au13C CDCl3 {C:\Bruker\TopSpin3.5pl6} 2111 34 — 100.63MHz
199.4
138.5
135.6
135.6
130.2
130.1
128.5
128.4
127.1
83.2
77.0 CDCl3
56.0
24.8
24.1
6.5
6.2
Cl
O
Bpin
Ph
4j
(13C NMR, CDCl3, 100 MHz)
f1 (ppm)

211105.434.12.fid — Fupeng Wu Ua-2 — Au11B CDCl3 {C:\Bruker\TopSpin3.5pl6} 2111 34 — 128.38MHz

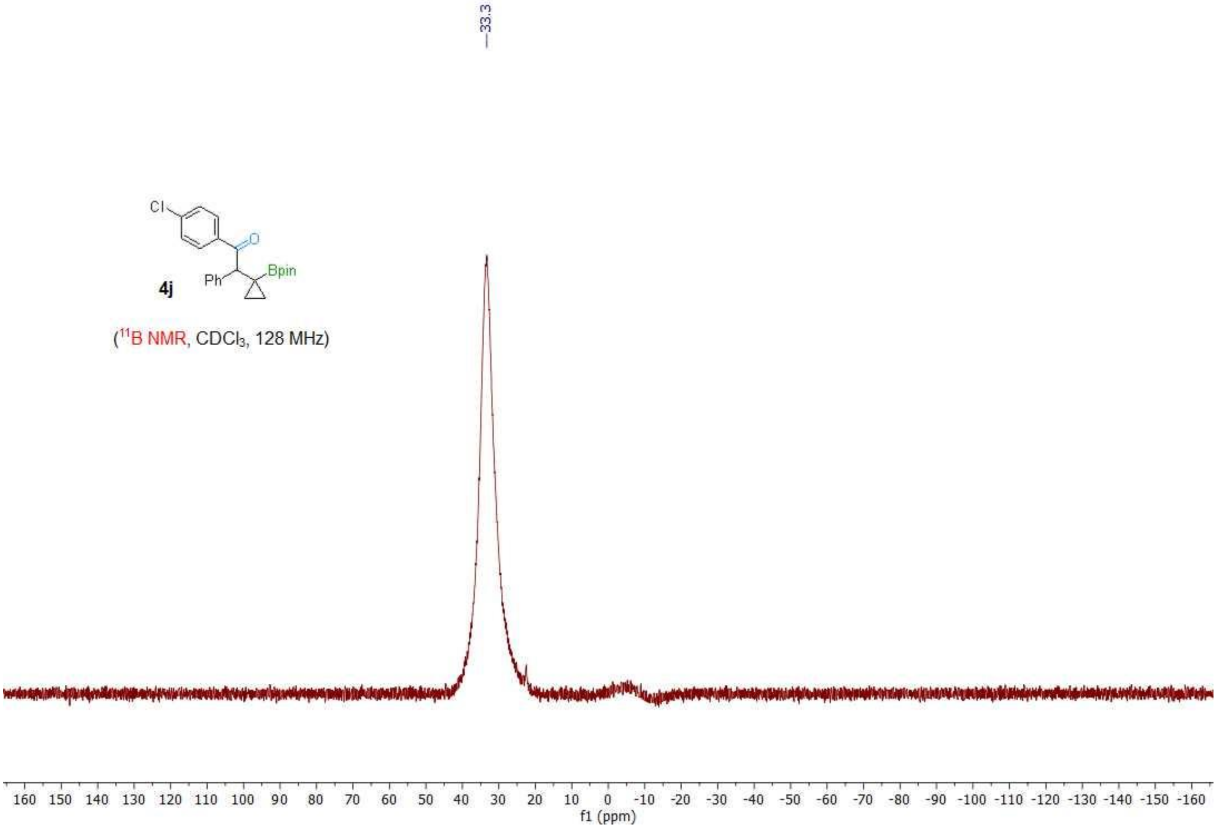

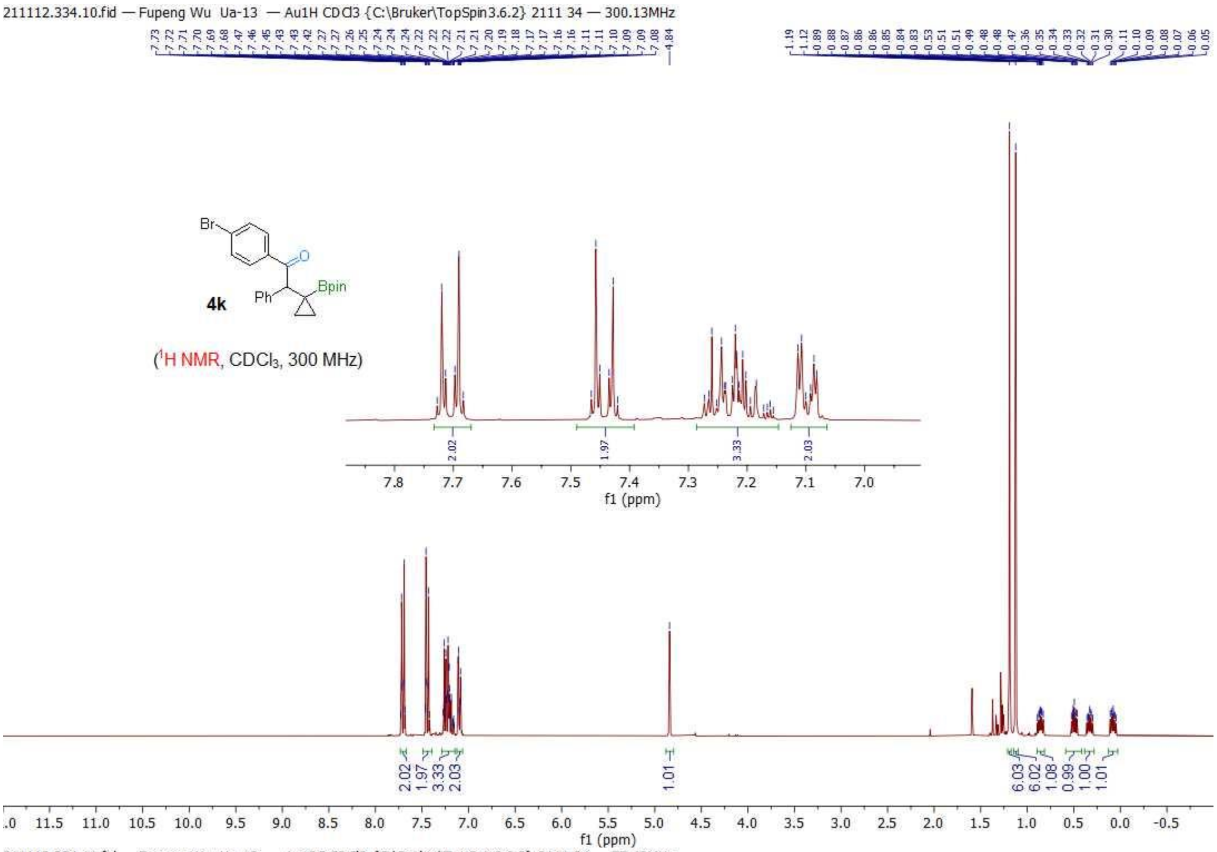
211112.334.10.fid — Fupeng Wu Ua-13 — Au1H CDCl3 {C:\Bruker\TopSpin3.6.2} 2111 34 — 300.13MHz
4k
Bpin
Ph
(^{1}H NMR, CDCl3, 300 MHz)
f1 (ppm)

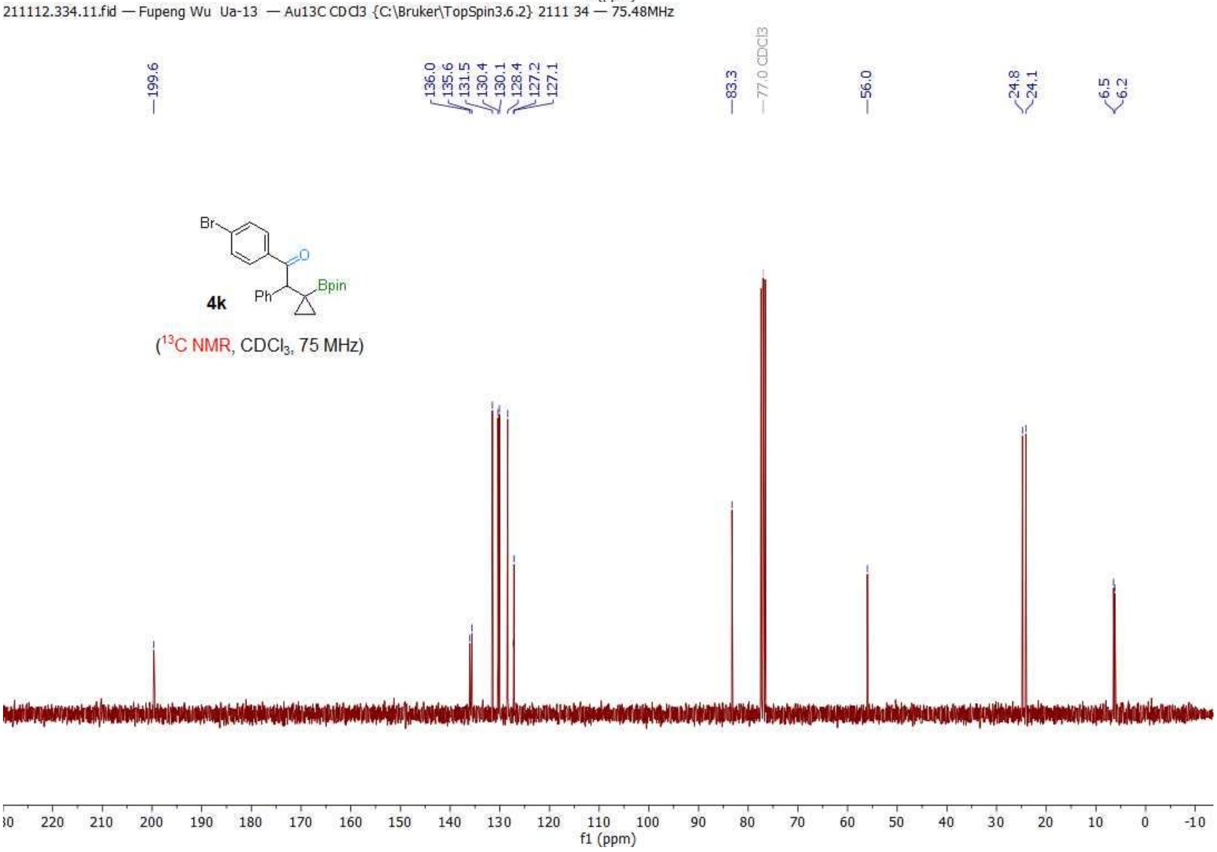
211112.334.11.fid — Fupeng Wu Ua-13 — Au13C CDCl3 {C:\Bruker\TopSpin3.6.2} 2111 34 — 75.48MHz
199.6
136.0
135.6
131.5
130.4
130.1
128.4
127.2
127.1
83.3
77.0 CDCl3
56.0
24.8
24.1
6.5
6.2
4k
Bpin
Ph
(^{13}C NMR, CDCl3, 75 MHz)
f1 (ppm)

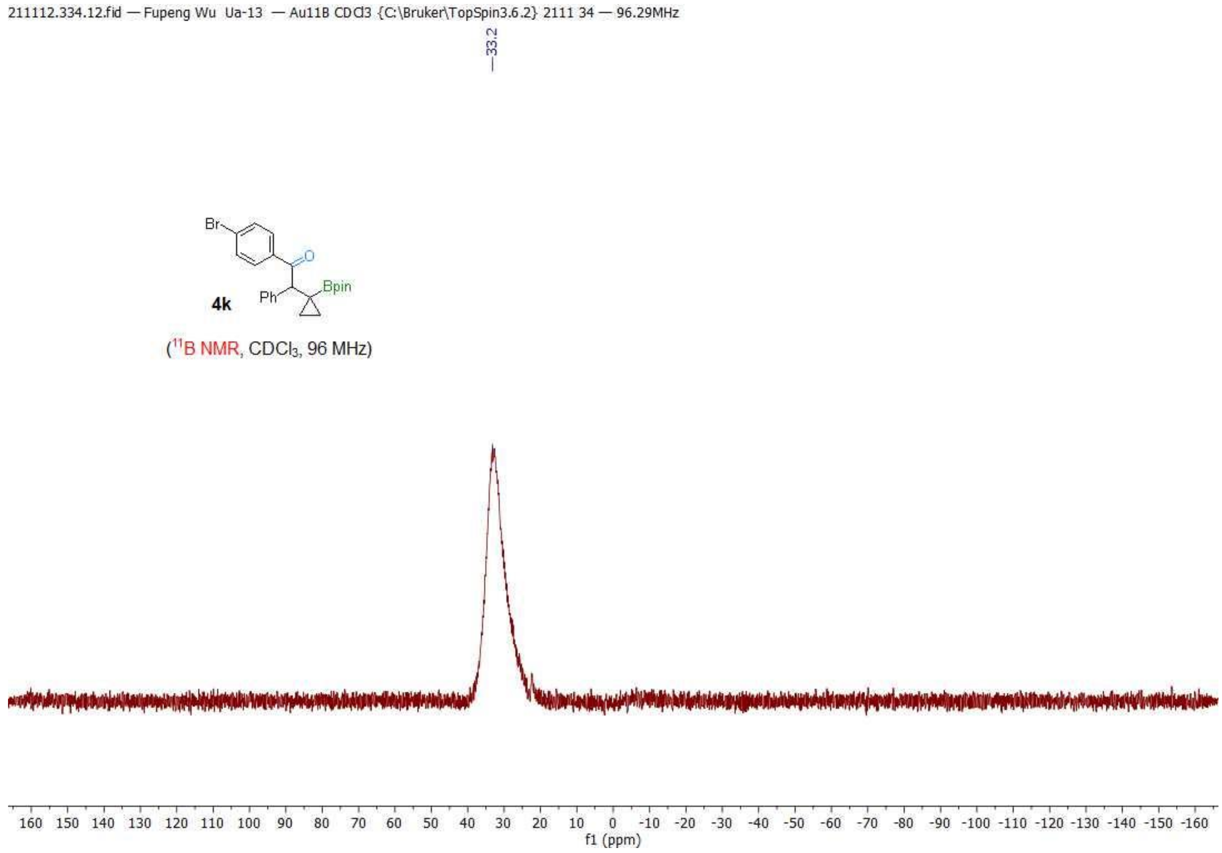
211112.334.12.fid — Fupeng Wu Ua-13 — Au11B CDCl3 {C:\Bruker\TopSpin3.6.2} 2111 34 — 96.29MHz
33.2
Br
O
Bpin
Ph
4k
(11B NMR, CDCl3, 96 MHz)
160 150 140 130 120 110 100 90 80 70 60 50 40 30 20 10 0 -10 -20 -30 -40 -50 -60 -70 -80 -90 -100 -110 -120 -130 -140 -150 -160
f1 (ppm)

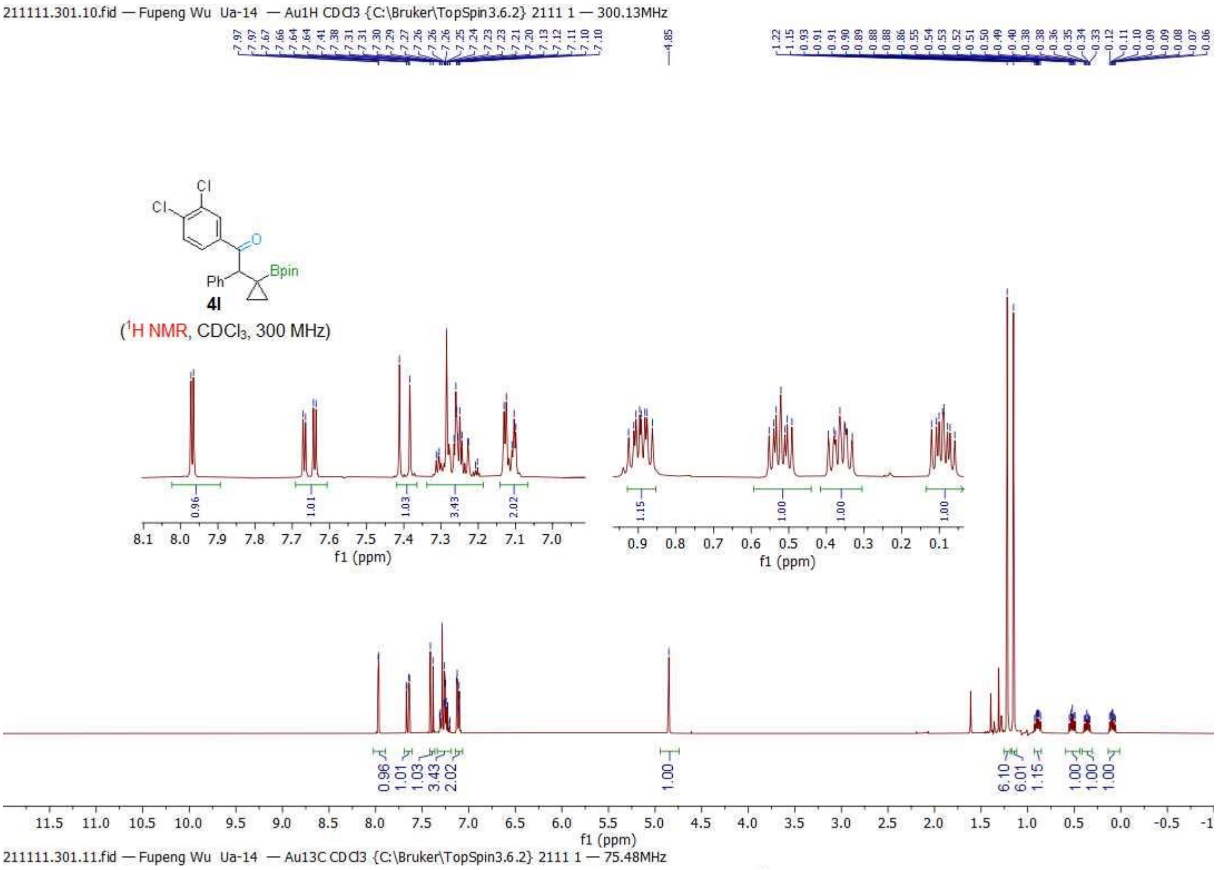
211111.301.10.fid — Fupeng Wu Ua-14 — Au1H CDCl3 {C:\Bruker\TopSpin3.6.2} 2111 1 — 300.13MHz
Cl
Cl
O
Ph
Bpin
4l
(1H NMR, CDCl3, 300 MHz)
f1 (ppm)

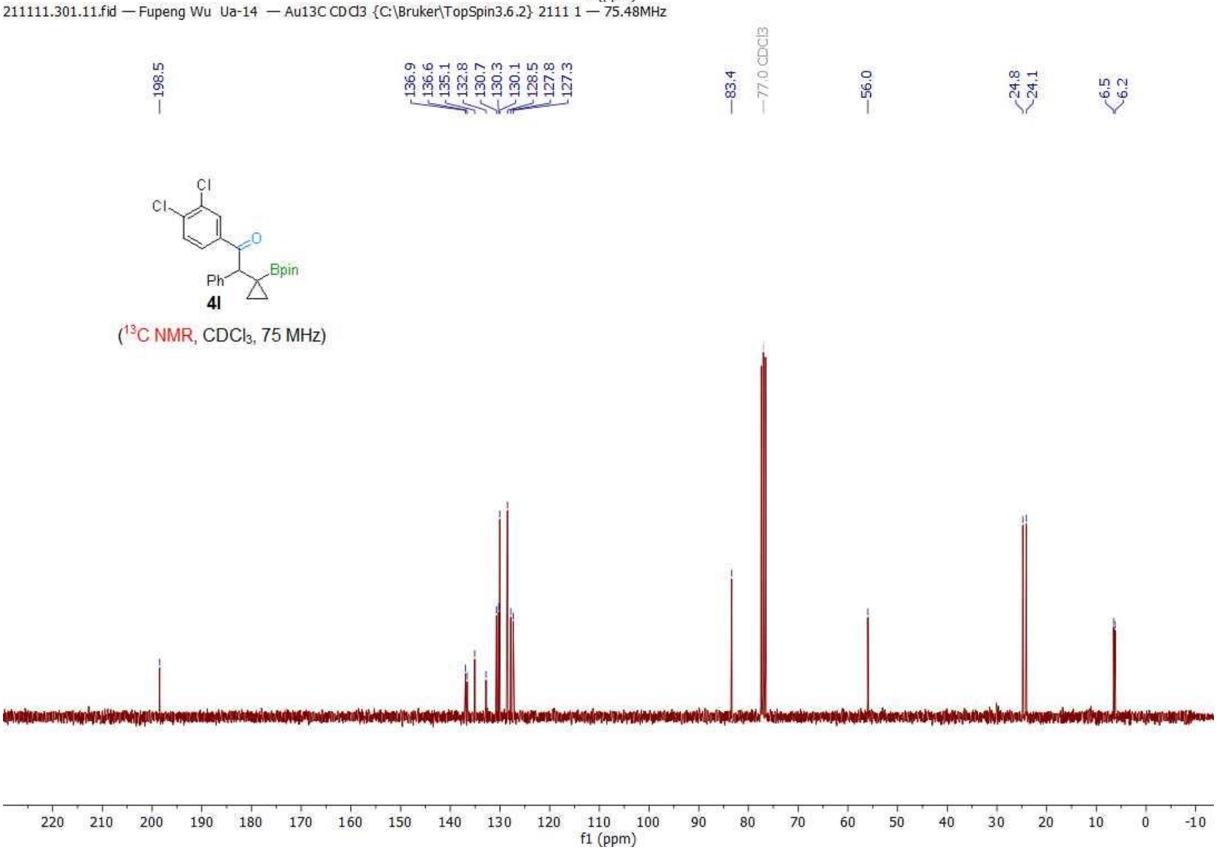
211111.301.11.fid — Fupeng Wu Ua-14 — Au13C CDCl3 {C:\Bruker\TopSpin3.6.2} 2111 1 — 75.48MHz
198.5
136.9
136.6
135.1
132.8
130.7
130.3
130.1
128.5
127.8
127.3
83.4
77.0 CDCl3
56.0
24.8
24.1
6.5
6.2
Cl
Cl
O
Ph
Bpin
4l
(13C NMR, CDCl3, 75 MHz)
f1 (ppm)

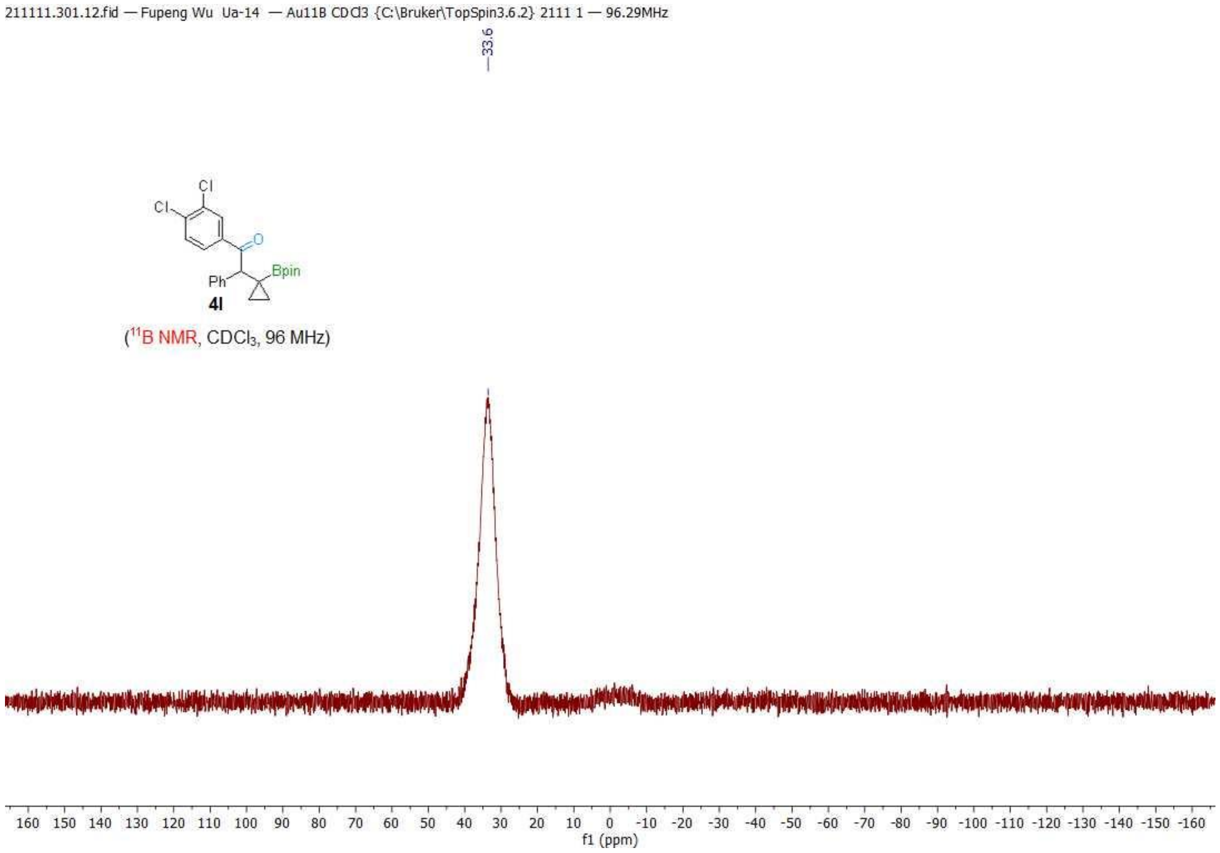
211111.301.12.fid — Fupeng Wu Ua-14 — Au11B CDCl3 {C:\Bruker\TopSpin3.6.2} 2111 1 — 96.29MHz
33.6
Cl
Cl
O
Bpin
Ph
4l
(11B NMR, CDCl3, 96 MHz)
160 150 140 130 120 110 100 90 80 70 60 50 40 30 20 10 0 -10 -20 -30 -40 -50 -60 -70 -80 -90 -100 -110 -120 -130 -140 -150 -160
f1 (ppm)

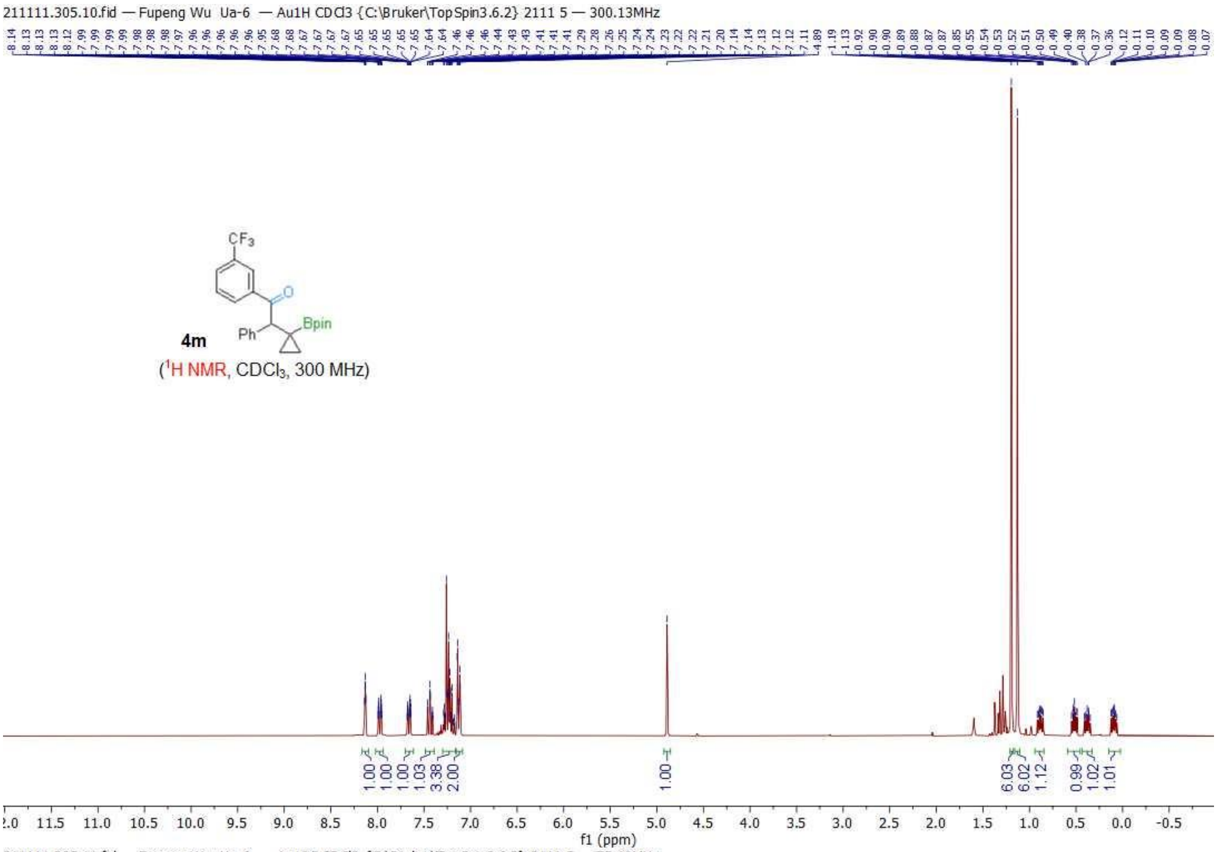
211111.305.10.fid — Fupeng Wu Ua-6 — Au1H CDCl3 {C:\Bruker\TopSpin3.6.2} 2111 5 — 300.13MHz
CF3
O
Bpin
Ph
4m
(1H NMR, CDCl3, 300 MHz)
1.00
1.00
1.00
1.03
3.38
2.00
1.00
6.03
6.02
1.12
0.99
1.02
1.01
f1 (ppm)

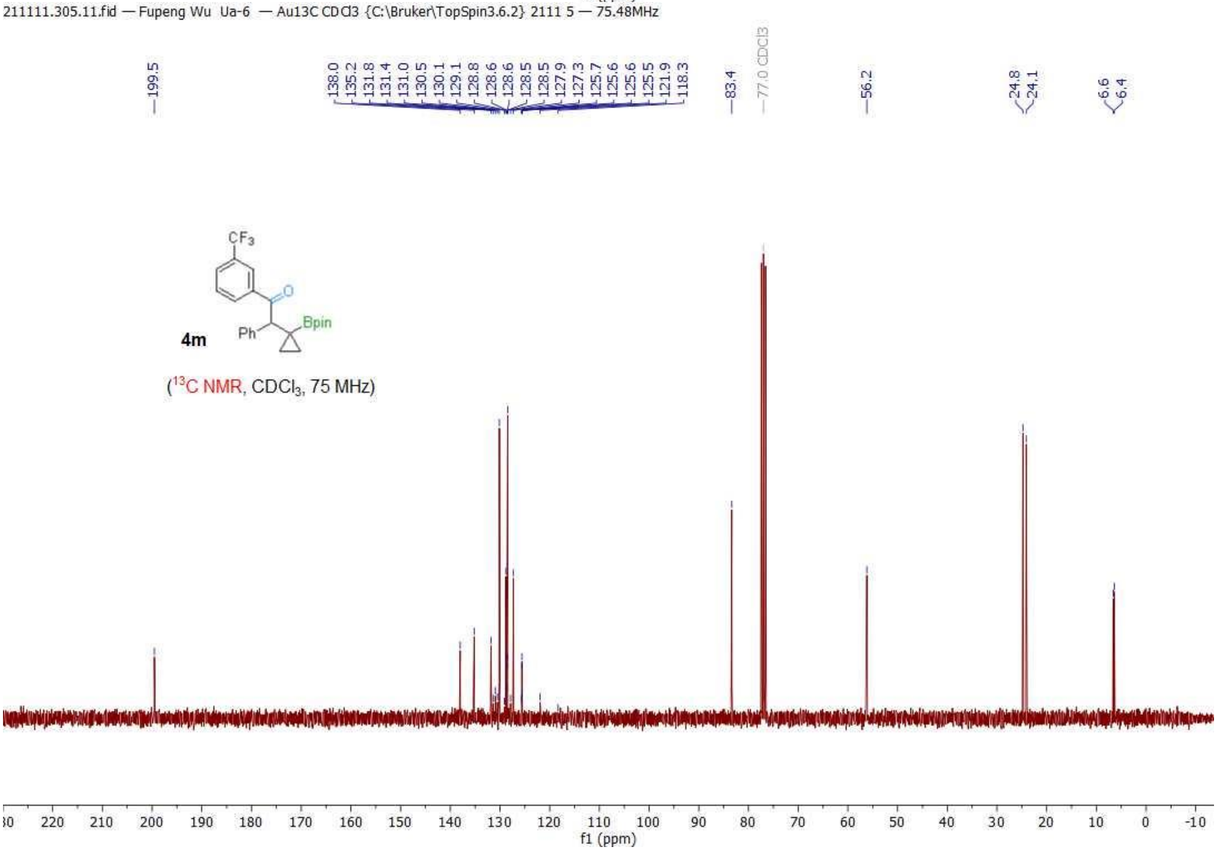
211111.305.11.fid — Fupeng Wu Ua-6 — Au13C CDCl3 {C:\Bruker\TopSpin3.6.2} 2111 5 — 75.48MHz
199.5
138.0
135.2
131.8
131.4
131.0
130.5
130.1
129.1
128.8
128.6
128.6
128.5
128.5
127.9
127.3
125.7
125.6
125.6
125.5
121.9
118.3
83.4
77.0 CDCl3
56.2
24.8
24.1
6.6
6.4
CF3
O
Bpin
Ph
4m
(13C NMR, CDCl3, 75 MHz)
f1 (ppm)

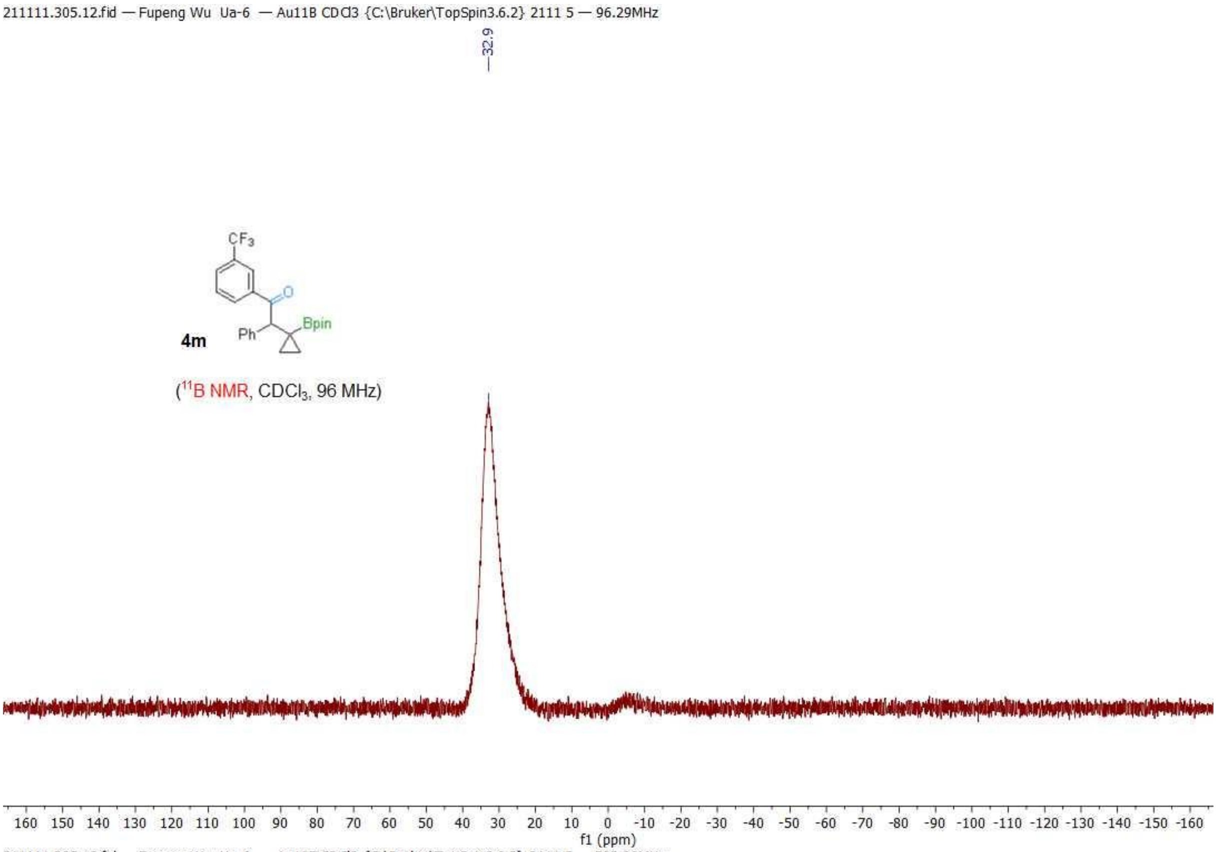
211111.305.12.fid — Fupeng Wu Ua-6 — Au11B CDCl3 {C:\Bruker\TopSpin3.6.2} 2111 5 — 96.29MHz
32.9
CF3
O
Bpin
Ph
4m
(11B NMR, CDCl3, 96 MHz)
160 150 140 130 120 110 100 90 80 70 60 50 40 30 20 10 0 -10 -20 -30 -40 -50 -60 -70 -80 -90 -100 -110 -120 -130 -140 -150 -160
f1 (ppm)

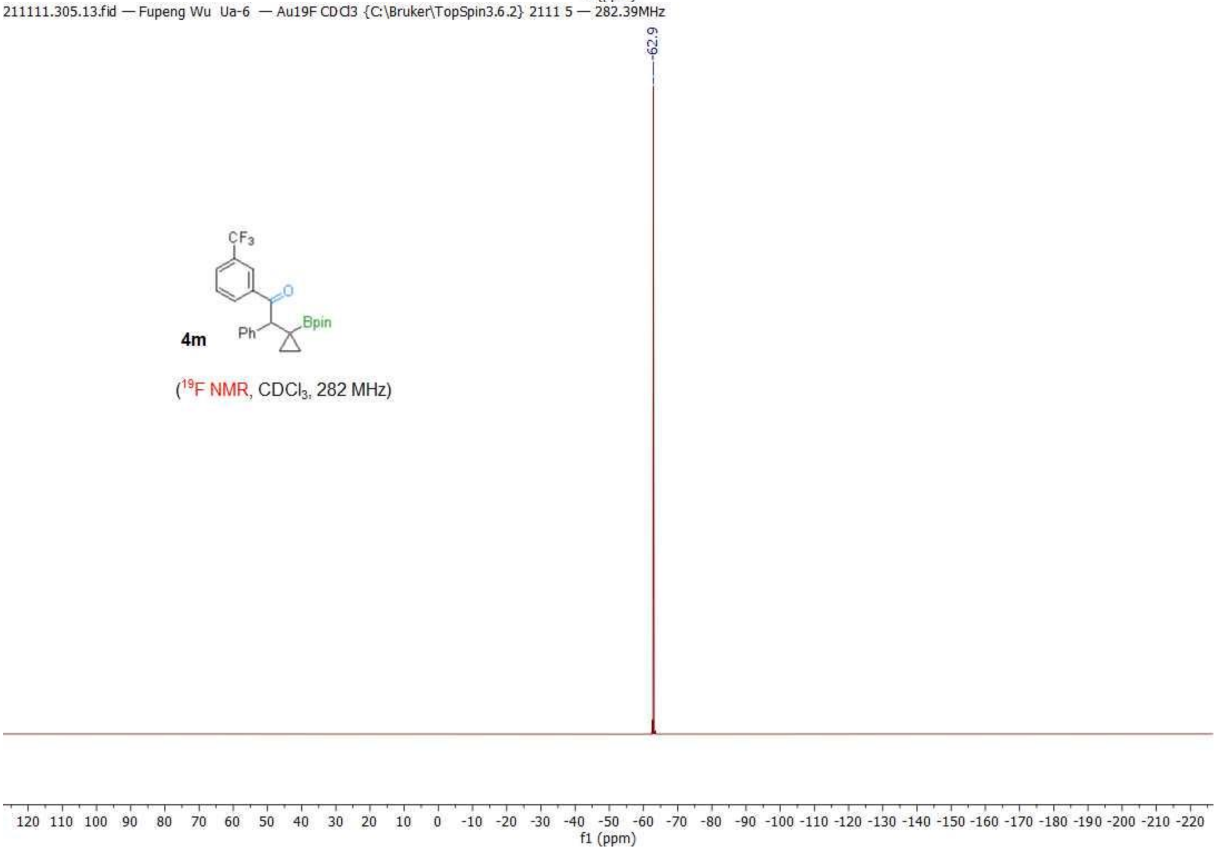
211111.305.13.fid — Fupeng Wu Ua-6 — Au19F CDCl3 {C:\Bruker\TopSpin3.6.2} 2111 5 — 282.39MHz
-62.9
CF3
O
Bpin
Ph
4m
(19F NMR, CDCl3, 282 MHz)
120 110 100 90 80 70 60 50 40 30 20 10 0 -10 -20 -30 -40 -50 -60 -70 -80 -90 -100 -110 -120 -130 -140 -150 -160 -170 -180 -190 -200 -210 -220
f1 (ppm)

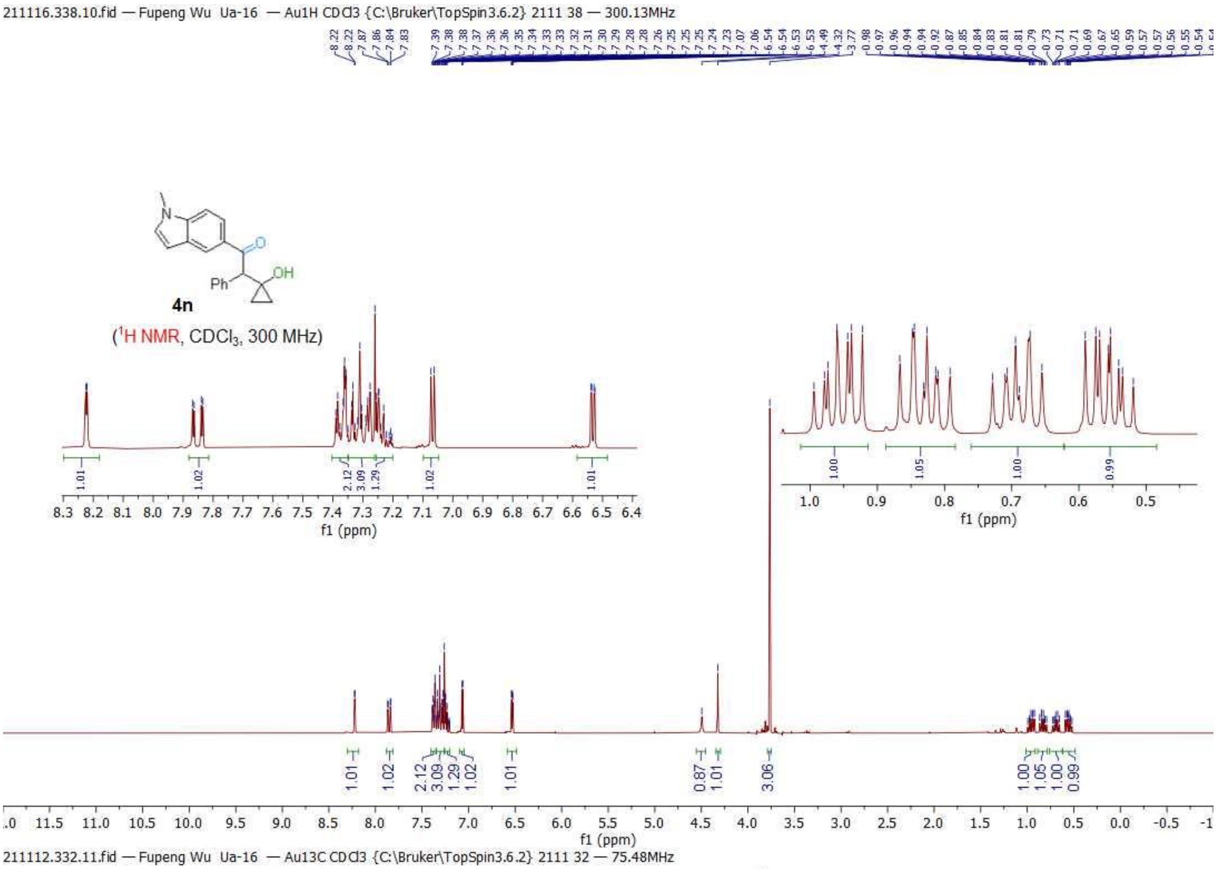
211116.338.10.fid — Fupeng Wu Ua-16 — Au1H CDCl3 {C:\Bruker\TopSpin3.6.2} 2111 38 — 300.13MHz
4n
(1H NMR, CDCl3, 300 MHz)
Ph
OH
N
O
f1 (ppm)

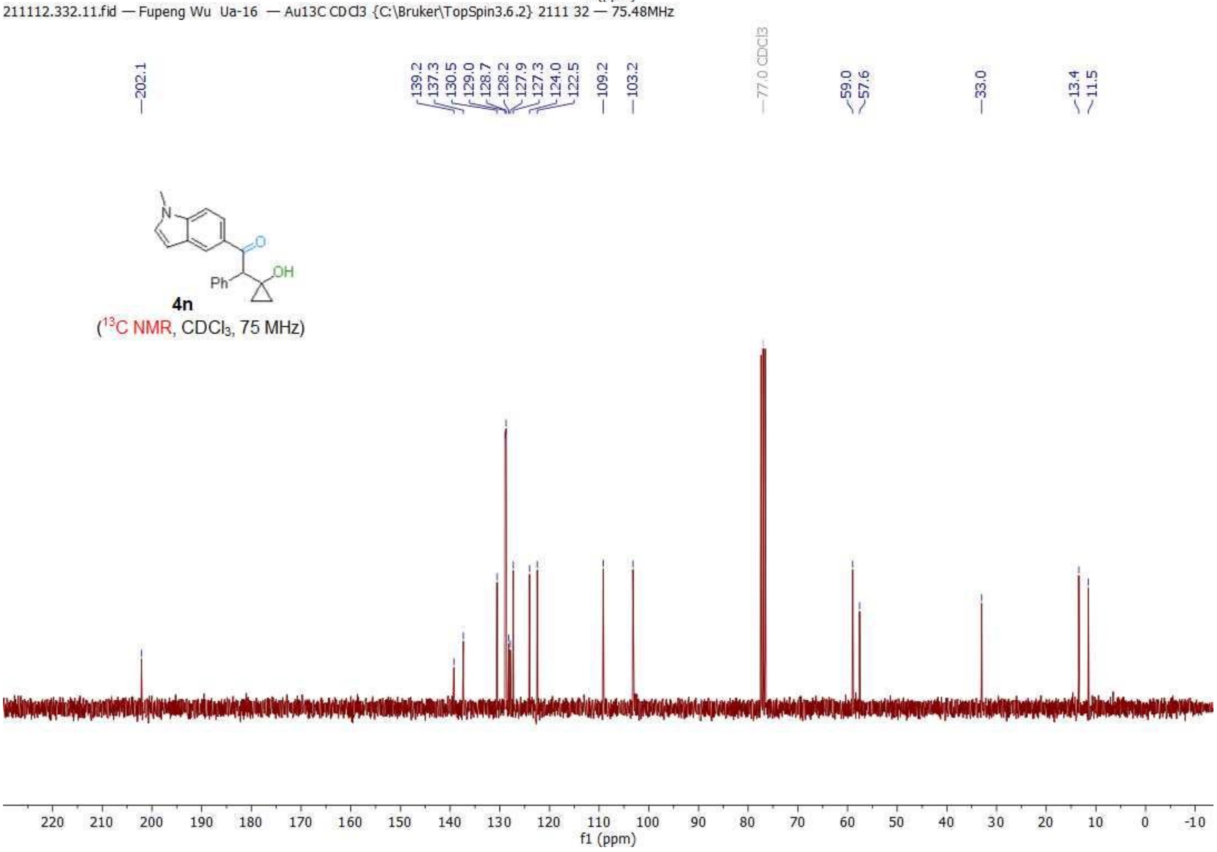
211112.332.11.fid — Fupeng Wu Ua-16 — Au13C CDCl3 {C:\Bruker\TopSpin3.6.2} 2111 32 — 75.48MHz
202.1
139.2
137.3
130.5
129.0
128.7
128.2
127.9
127.3
124.0
122.5
109.2
103.2
77.0 CDCl3
59.0
57.6
33.0
13.4
11.5
4n
(13C NMR, CDCl3, 75 MHz)
Ph
OH
N
O
f1 (ppm)

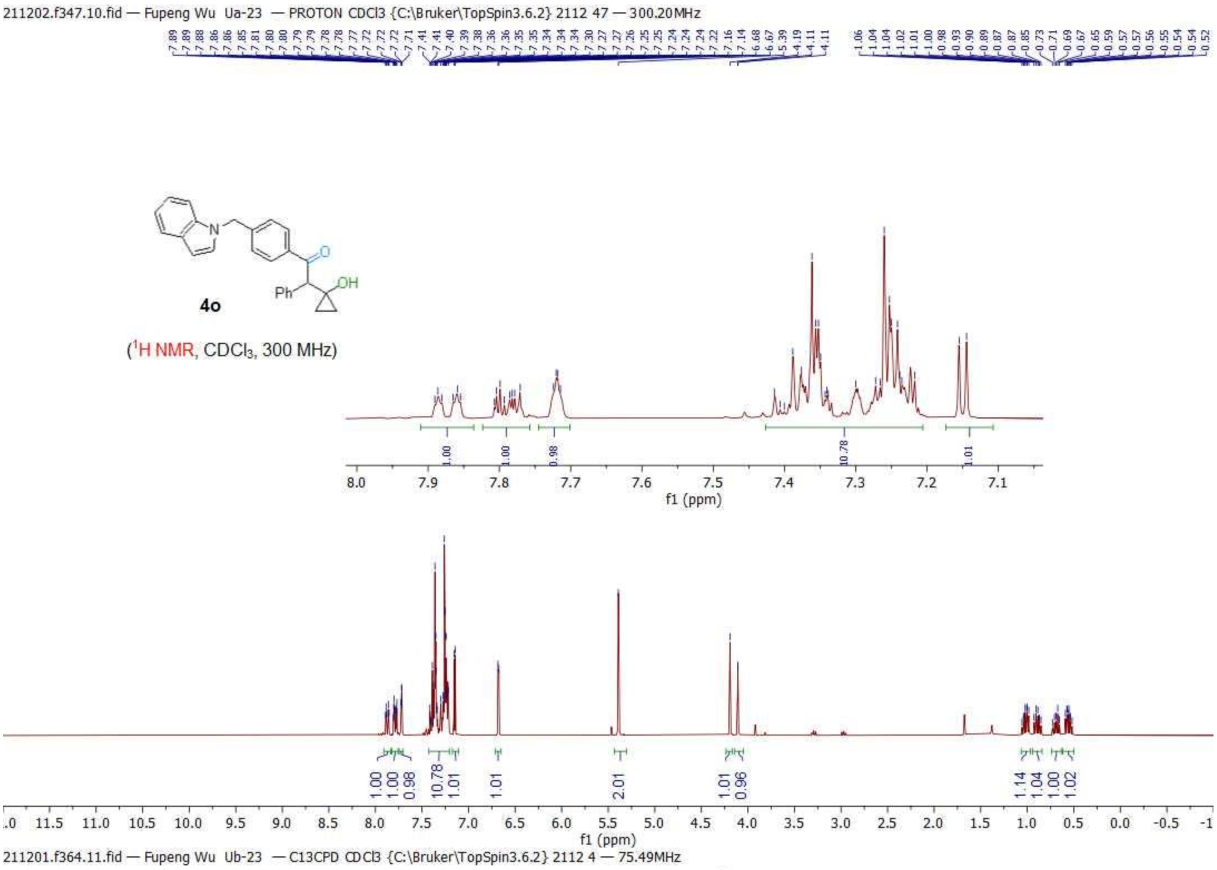
211202.f347.10.fid — Fupeng Wu Ua-23 — PROTON CDCl3 {C:\Bruker\TopSpin3.6.2} 2112 47 — 300.20MHz
4o
(1H NMR, CDCl3, 300 MHz)
f1 (ppm)

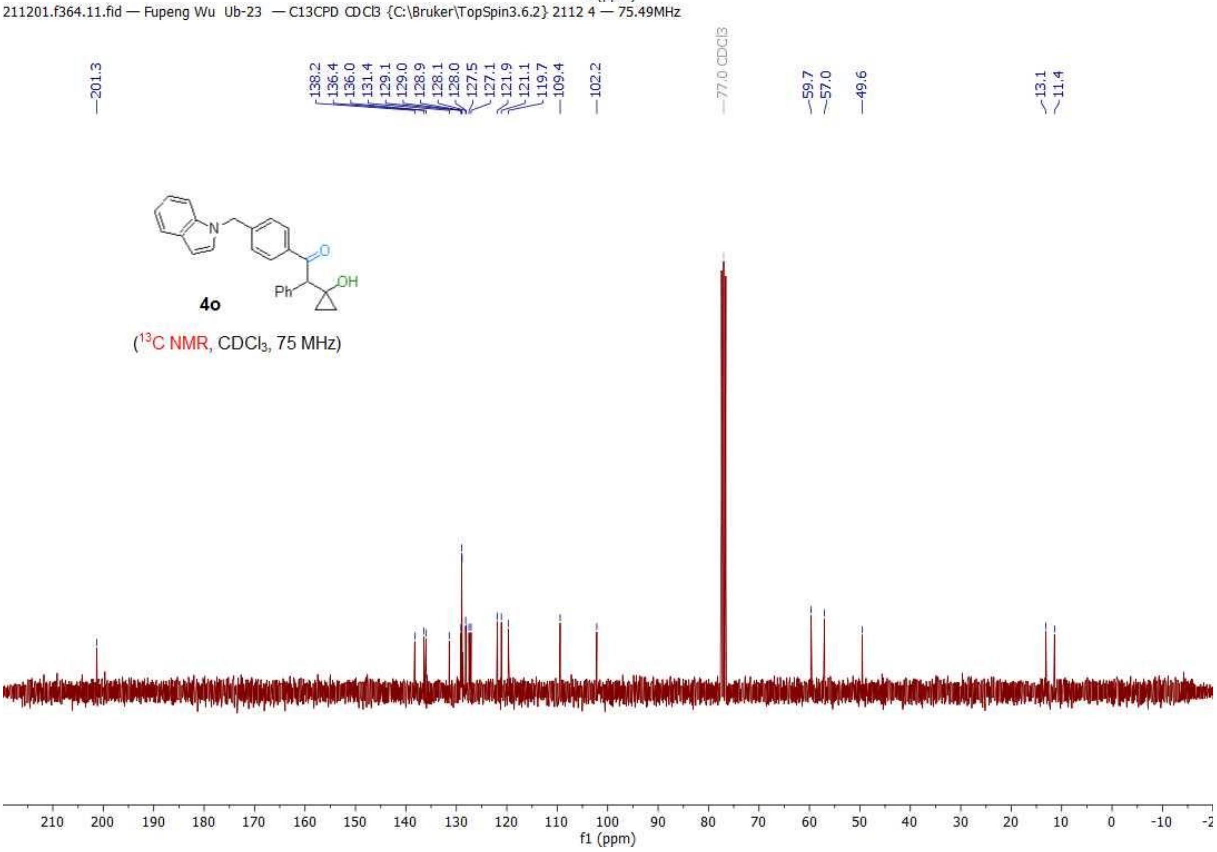
211201.f364.11.fid — Fupeng Wu Ub-23 — C13CPD CDCl3 {C:\Bruker\TopSpin3.6.2} 2112 4 — 75.49MHz
201.3
138.2 136.4 136.0 131.4 129.1 129.0 128.9 128.1 128.0 127.5 127.1 121.9 121.1 119.7 109.4
102.2
77.0 CDCl3
59.7 57.0
49.6
13.1 11.4
4o
(13C NMR, CDCl3, 75 MHz)
f1 (ppm)

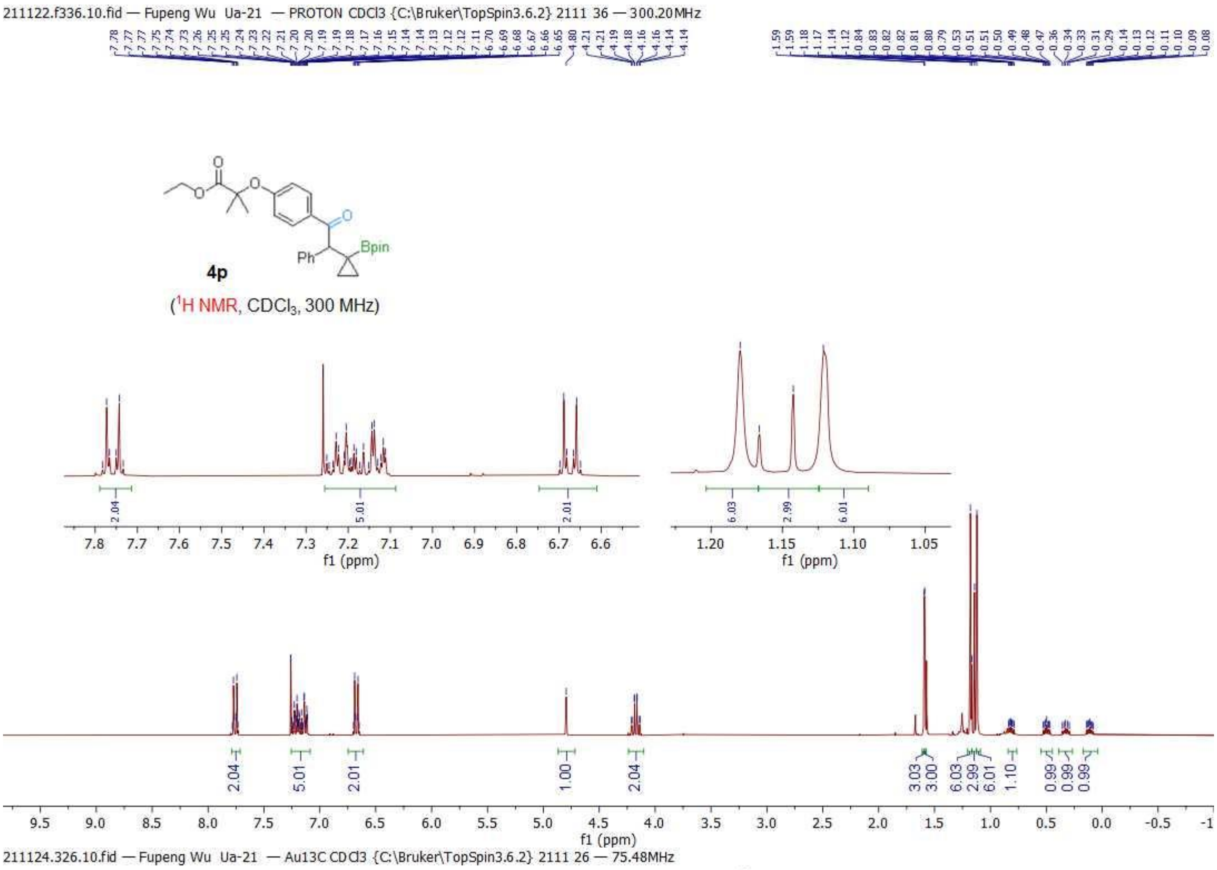
211122.f336.10.fid — Fupeng Wu Ua-21 — PROTON CDCl3 {C:\Bruker\TopSpin3.6.2} 2111 36 — 300.20MHz
7.78 7.77 7.75 7.74 7.73 7.26 7.25 7.25 7.24 7.23 7.22 7.21 7.20 7.20 7.19 7.19 7.18 7.17 7.16 7.15 7.14 7.14 7.13 7.12 7.12 7.12 7.11 6.70 6.69 6.68 6.67 6.66 6.65 4.80 4.21 4.21 4.19 4.18 4.16 4.16 4.14 4.14
1.59 1.59 1.18 1.17 1.14 1.12 0.84 0.83 0.82 0.82 0.81 0.80 0.79 0.53 0.51 0.51 0.50 0.49 0.48 0.47 0.36 0.34 0.33 0.31 0.29 0.14 0.13 0.12 0.11 0.10 0.09 0.08
O
O
Bpin
Ph
4p
(1H NMR, CDCl3, 300 MHz)
2.04
5.01
2.01
6.03
2.99
6.01
1.00
2.04
3.03
3.00
1.10
0.99
0.99
0.99
f1 (ppm)

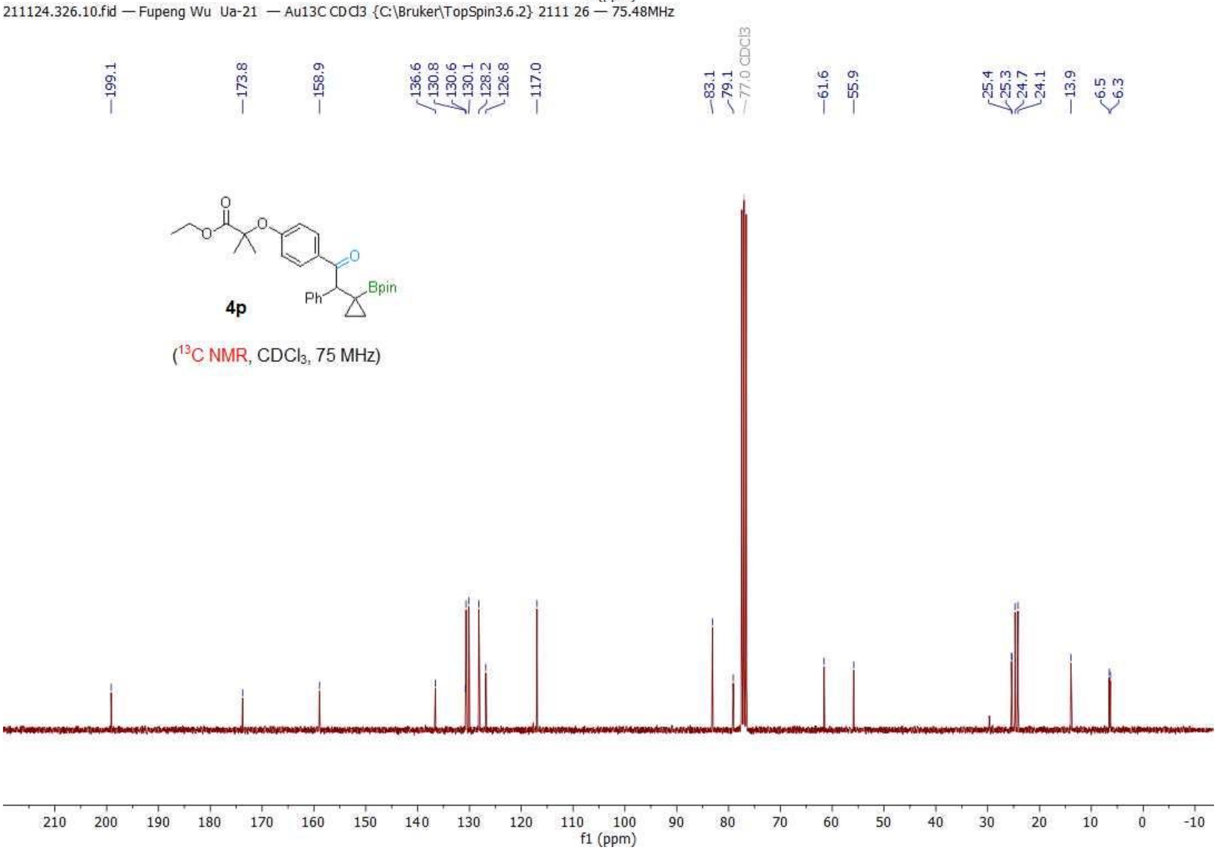
211124.326.10.fid — Fupeng Wu Ua-21 — Au13C CDCl3 {C:\Bruker\TopSpin3.6.2} 2111 26 — 75.48MHz
199.1
173.8
158.9
136.6 130.8 130.6 130.1 128.2 126.8
117.0
83.1 79.1 77.0 CDCl3
61.6
55.9
25.4 25.3 24.7 24.1
13.9
6.5 6.3
O
O
Bpin
Ph
4p
(13C NMR, CDCl3, 75 MHz)
f1 (ppm)

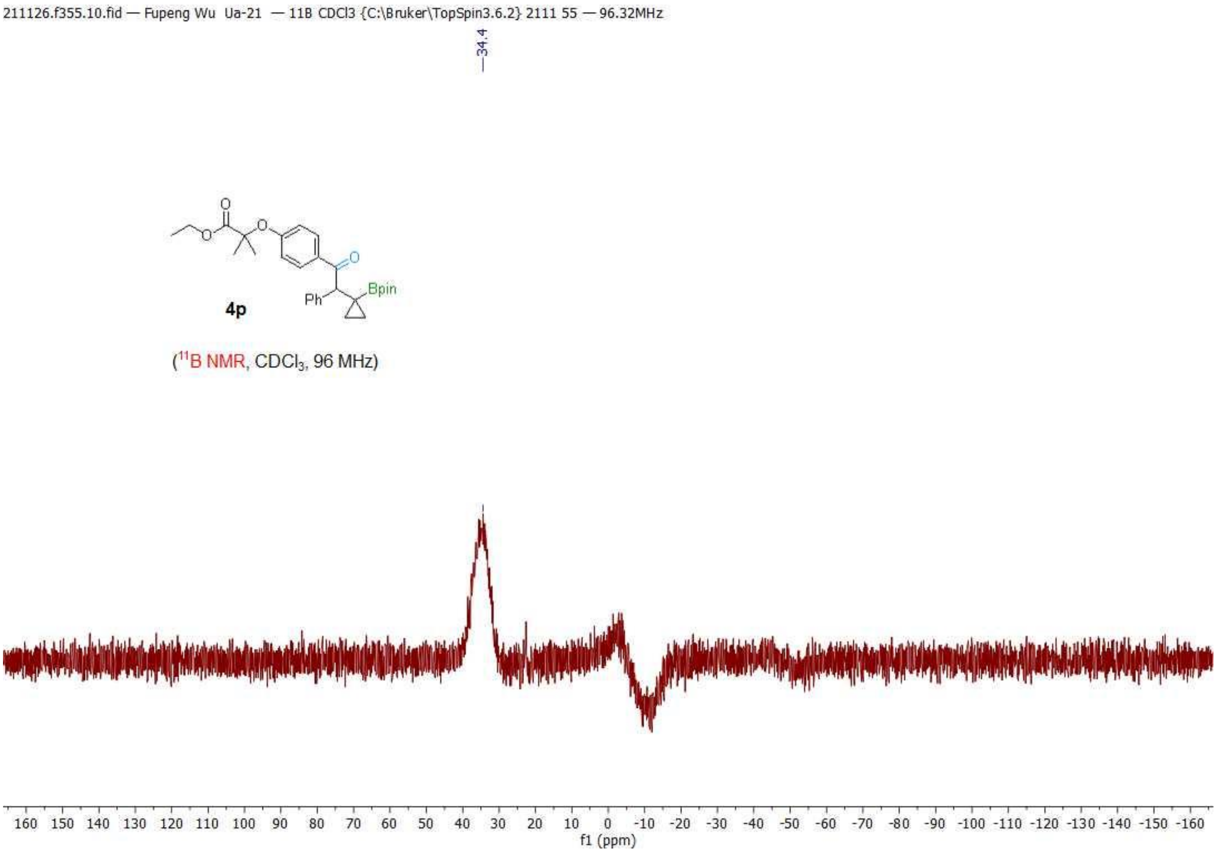
211126.f355.10.fid — Fupeng Wu Ua-21 — 11B CDCl3 {C:\Bruker\TopSpin3.6.2} 2111 55 — 96.32MHz
34.4
Ph
Bpin
4p
(^{11}B NMR, $CDCl_3$, 96 MHz)
160 150 140 130 120 110 100 90 80 70 60 50 40 30 20 10 0 -10 -20 -30 -40 -50 -60 -70 -80 -90 -100 -110 -120 -130 -140 -150 -160
f1 (ppm)

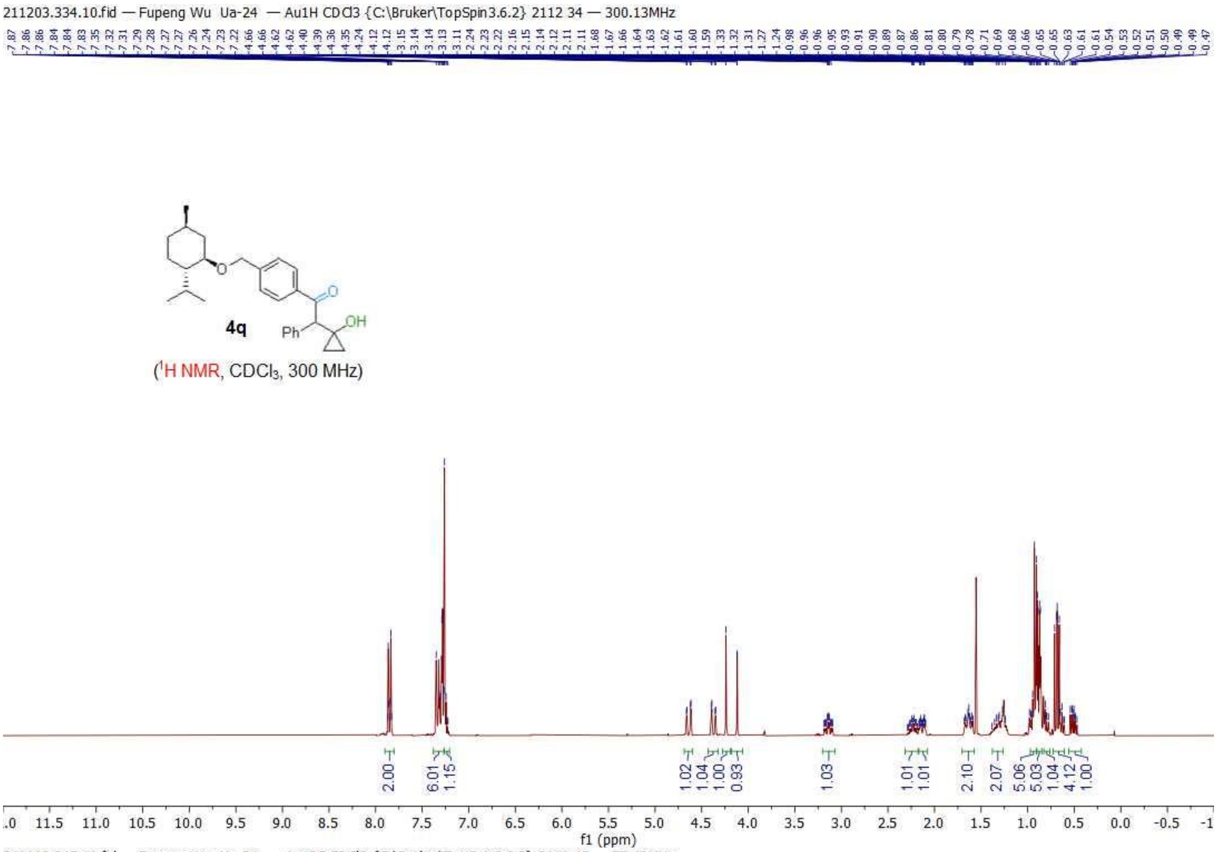
211203.334.10.fid — Fupeng Wu Ua-24 — Au1H CDCl3 {C:\Bruker\TopSpin3.6.2} 2112 34 — 300.13MHz
4q
Ph
OH
(1H NMR, CDCl3, 300 MHz)
f1 (ppm)

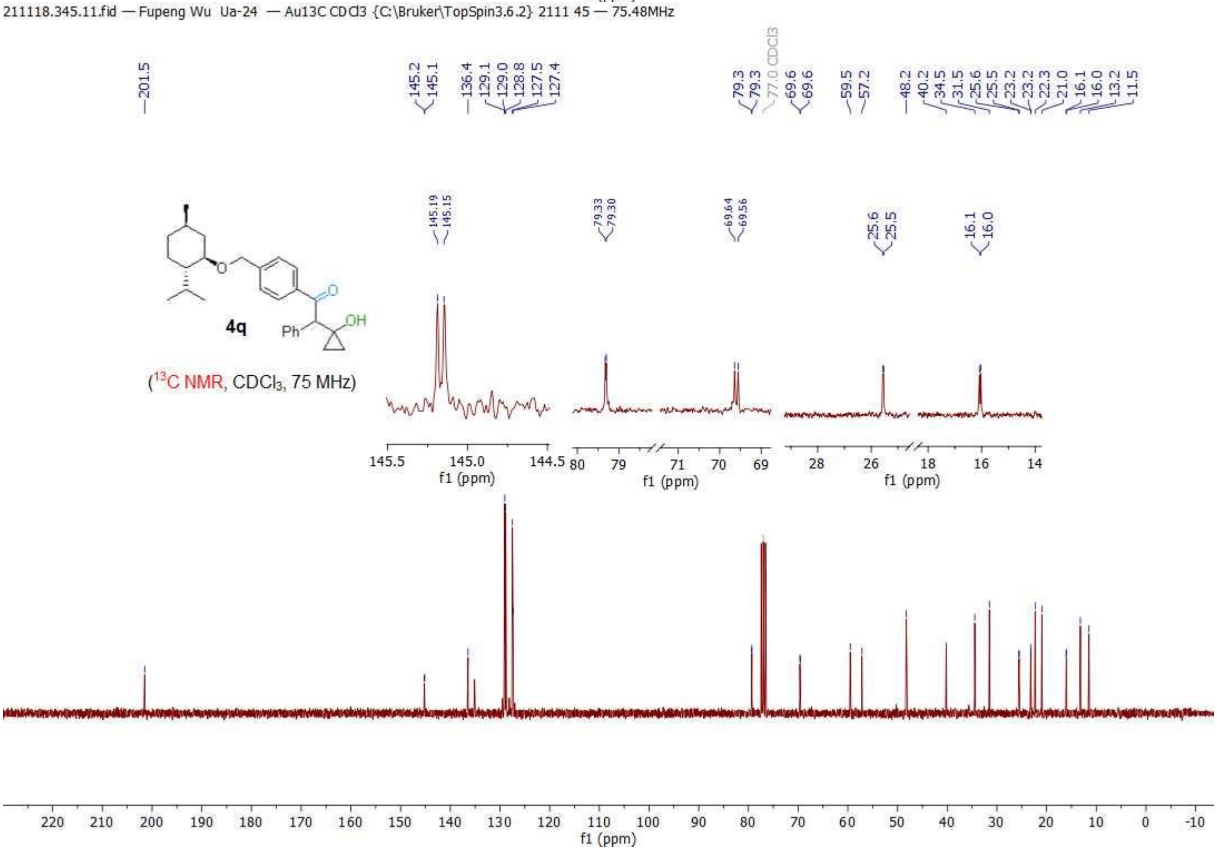
211118.345.11.fid — Fupeng Wu Ua-24 — Au13C CDCl3 {C:\Bruker\TopSpin3.6.2} 2111 45 — 75.48MHz
4q
Ph
OH
(13C NMR, CDCl3, 75 MHz)
f1 (ppm)

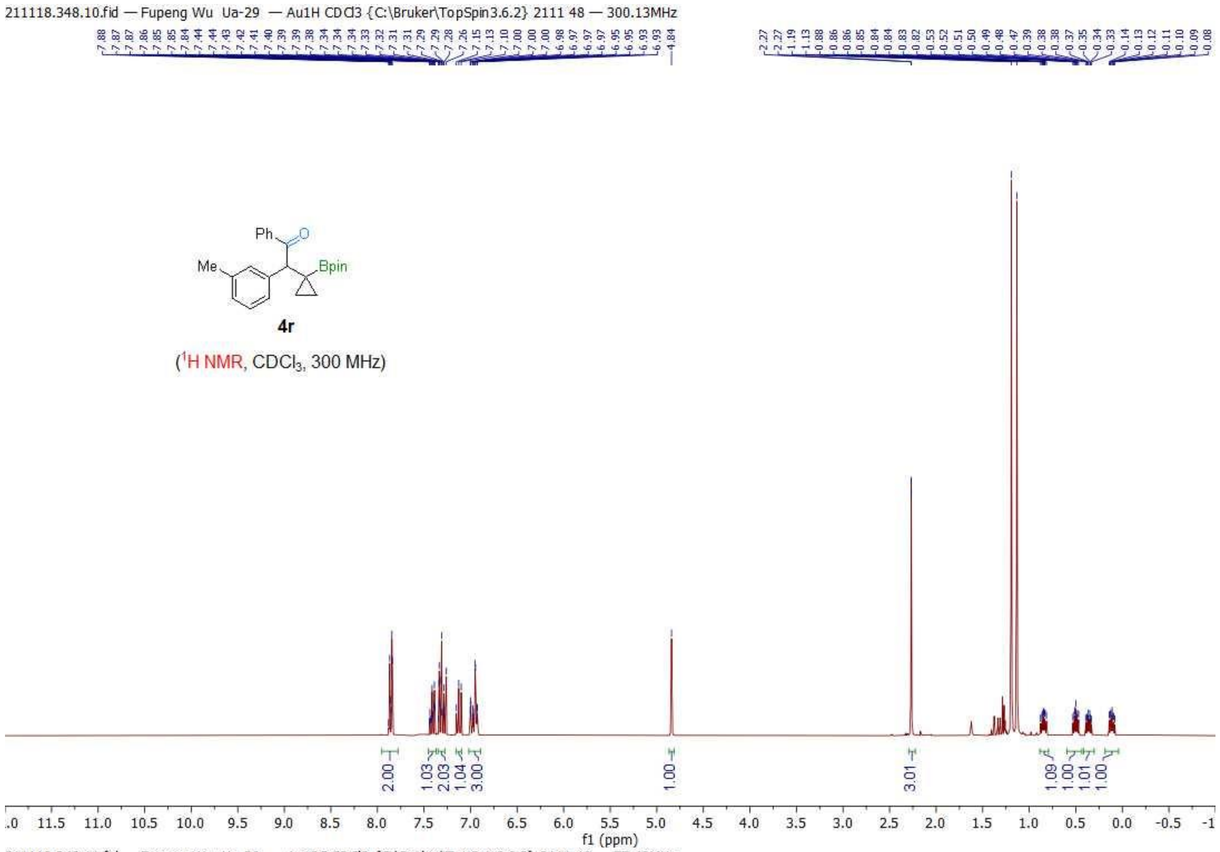
211118.348.10.fid — Fupeng Wu Ua-29 — Au1H CDCl3 {C:\Bruker\TopSpin3.6.2} 2111 48 — 300.13MHz
Ph
O
Me
Bpin
4r
(^{1}H NMR, CDCl$_3$, 300 MHz)
2.00
1.03
2.03
1.04
3.00
1.00
3.01
1.09
1.00
1.01
1.00
f1 (ppm)

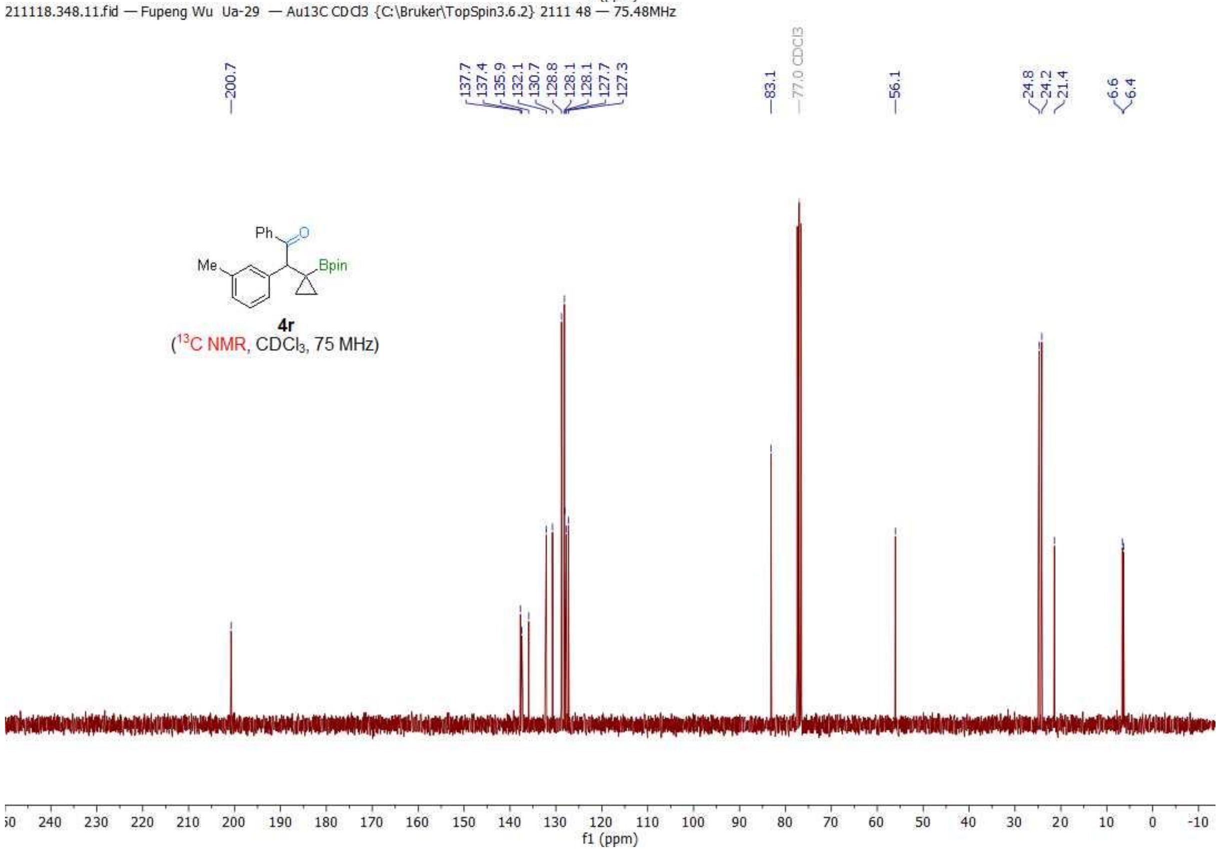
211118.348.11.fid — Fupeng Wu Ua-29 — Au13C CDCl3 {C:\Bruker\TopSpin3.6.2} 2111 48 — 75.48MHz
200.7
137.7
137.4
135.9
132.1
130.7
128.8
128.1
128.1
127.7
127.3
83.1
77.0 CDCl3
56.1
24.8
24.2
21.4
6.6
6.4
Ph
O
Me
Bpin
4r
(^{13}C NMR, CDCl$_3$, 75 MHz)
f1 (ppm)

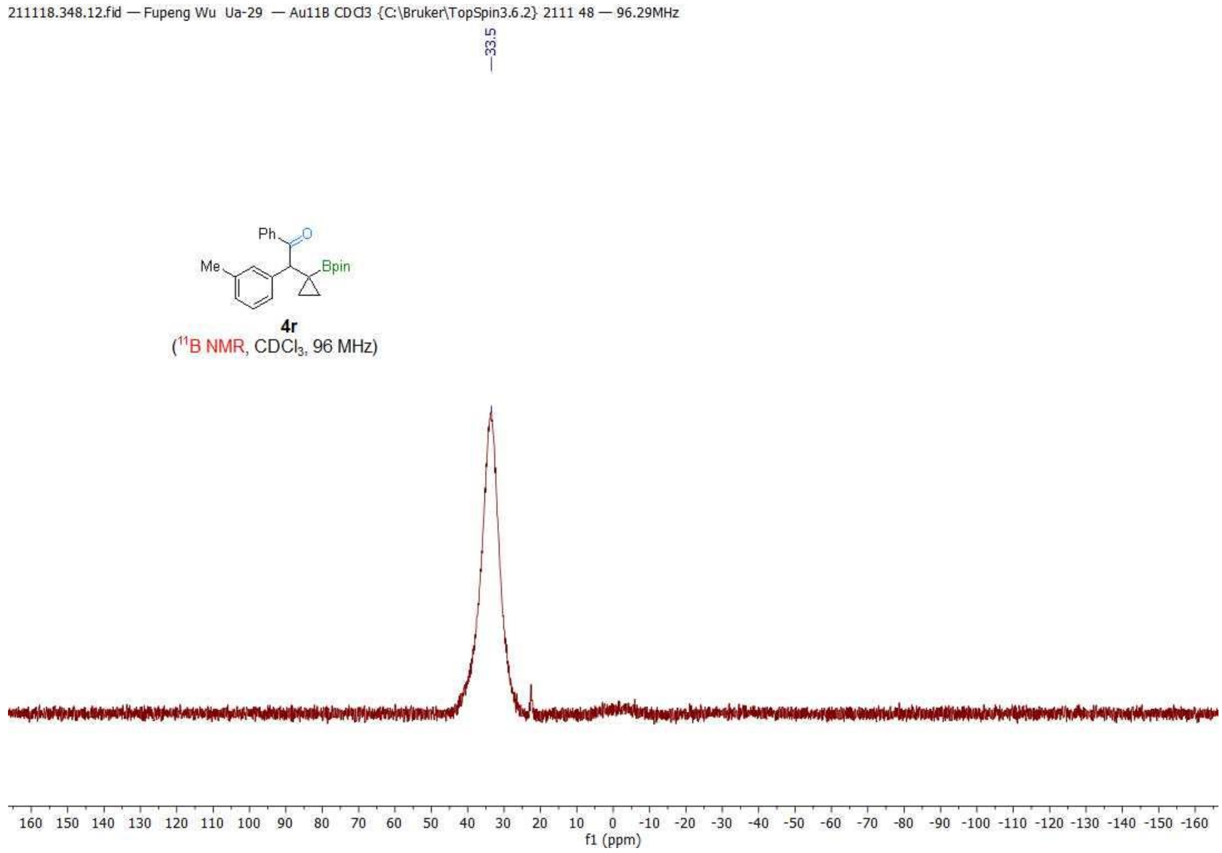
211118.348.12.fid — Fupeng Wu Ua-29 — Au11B CDCl3 {C:\Bruker\TopSpin3.6.2} 2111 48 — 96.29MHz
33.5
Ph
O
Me
Bpin
4r
(11B NMR, CDCl3, 96 MHz)
160 150 140 130 120 110 100 90 80 70 60 50 40 30 20 10 0 -10 -20 -30 -40 -50 -60 -70 -80 -90 -100 -110 -120 -130 -140 -150 -160
f1 (ppm)

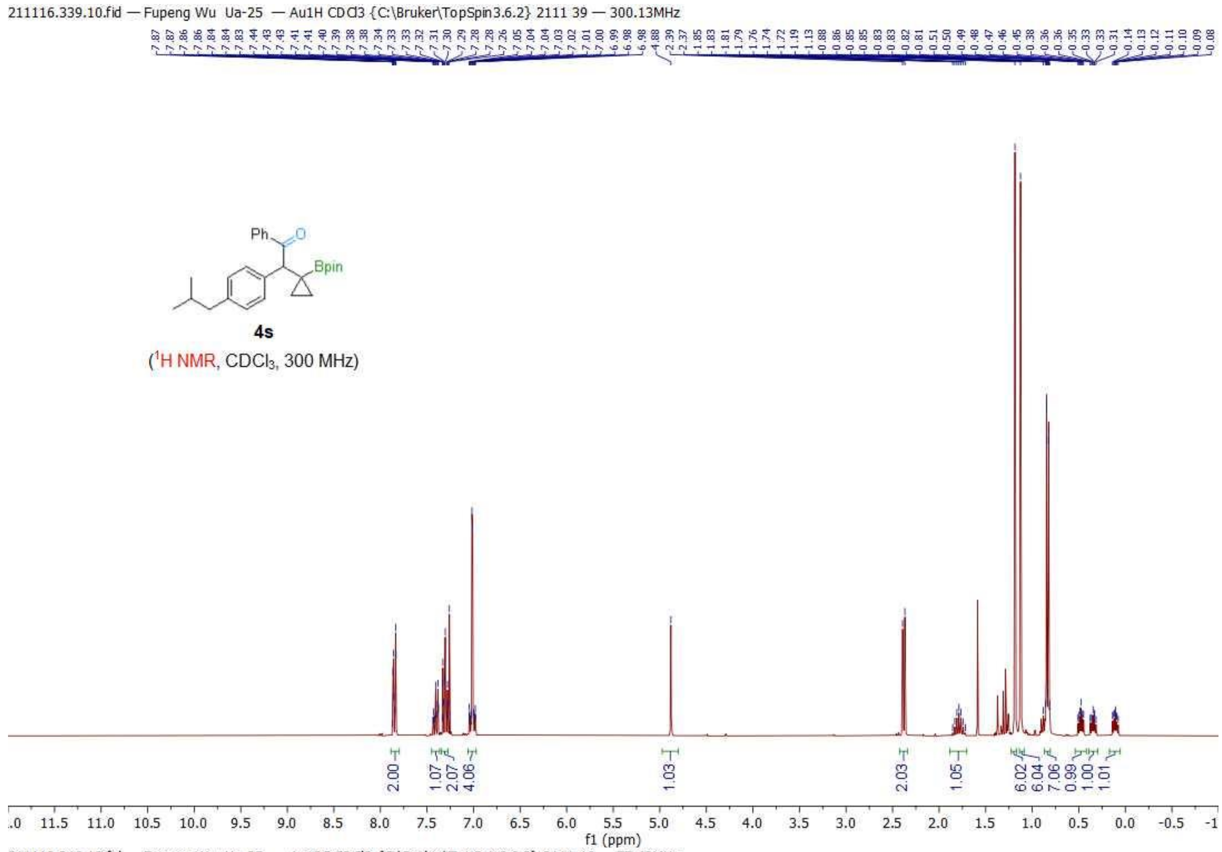
211116.339.10.fid — Fupeng Wu Ua-25 — Au1H CDCl3 {C:\Bruker\TopSpin3.6.2} 2111 39 — 300.13MHz
7.87 7.87 7.86 7.84 7.83 7.44 7.43 7.43 7.41 7.41 7.40 7.39 7.38 7.38 7.34 7.33 7.33 7.32 7.31 7.30 7.29 7.28 7.28 7.26 7.05 7.04 7.04 7.03 7.02 7.01 7.00 6.99 6.98 6.98 4.88 2.39 2.37 1.85 1.83 1.81 1.79 1.76 1.74 1.72 1.19 1.13 0.88 0.86 0.85 0.85 0.83 0.83 0.82 0.81 0.51 0.50 0.49 0.48 0.47 0.46 0.45 0.38 0.36 0.36 0.35 0.33 0.33 0.31 0.14 0.13 0.12 0.11 0.10 0.09 0.08
Ph
O
Bpin
4s
(1H NMR, CDCl3, 300 MHz)
2.00 1.07 2.07 4.06 1.03 2.03 1.05 6.02 6.04 7.06 0.99 1.00 1.01
f1 (ppm)

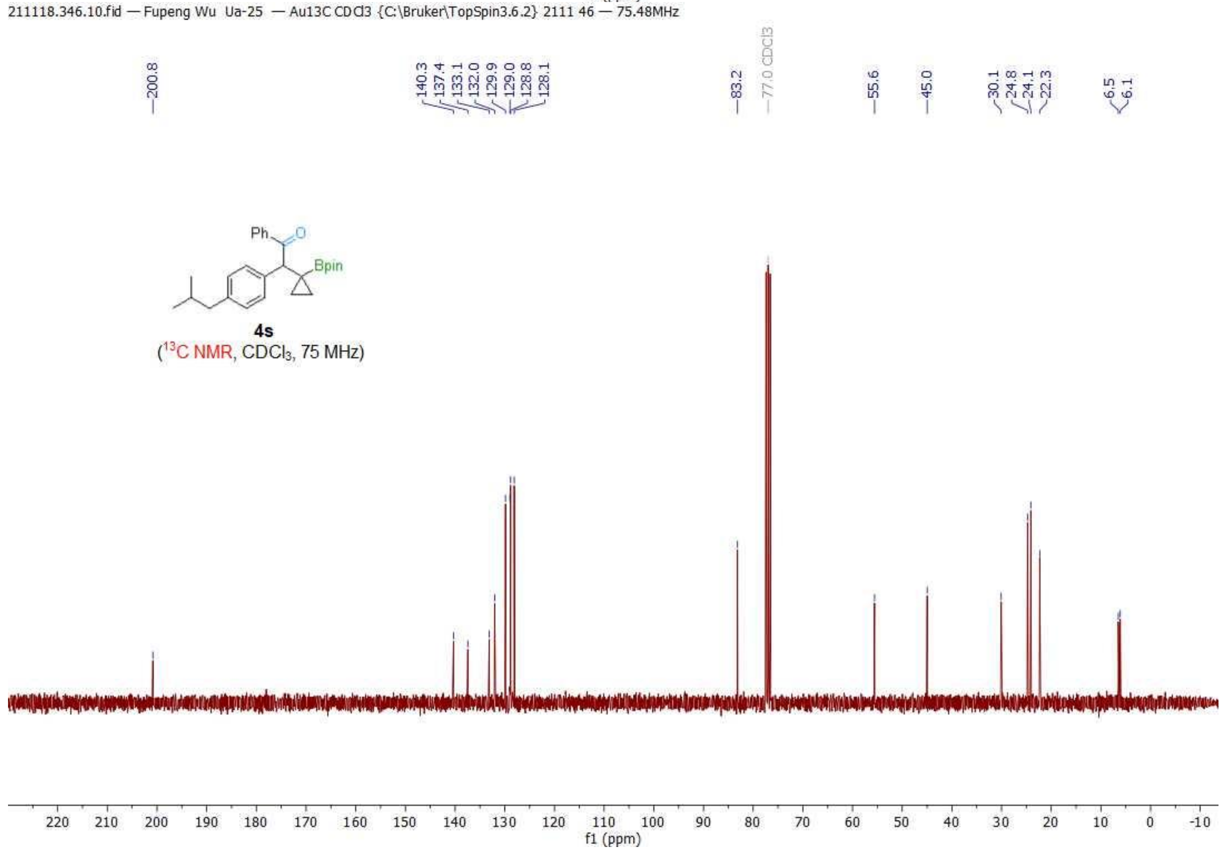
211118.346.10.fid — Fupeng Wu Ua-25 — Au13C CDCl3 {C:\Bruker\TopSpin3.6.2} 2111 46 — 75.48MHz
200.8
140.3 137.4 133.1 132.0 129.9 129.0 128.8 128.1
83.2
77.0 CDCl3
55.6
45.0
30.1 24.8 24.1 22.3
6.5 6.1
Ph
O
Bpin
4s
(13C NMR, CDCl3, 75 MHz)
f1 (ppm)

211118.346.11.fid — Fupeng Wu Ua-25 — Au11B CDCl3 {C:\Bruker\TopSpin3.6.2} 2111 46 — 96.29MHz

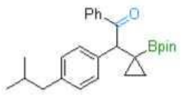

4s

(^{11}B NMR, $CDCl_3$, 96 MHz)

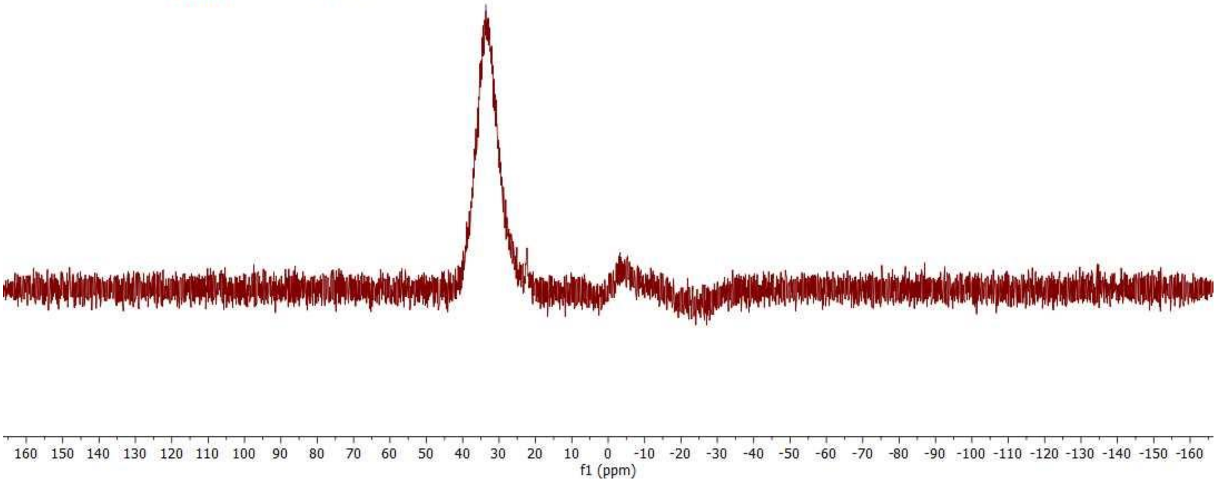

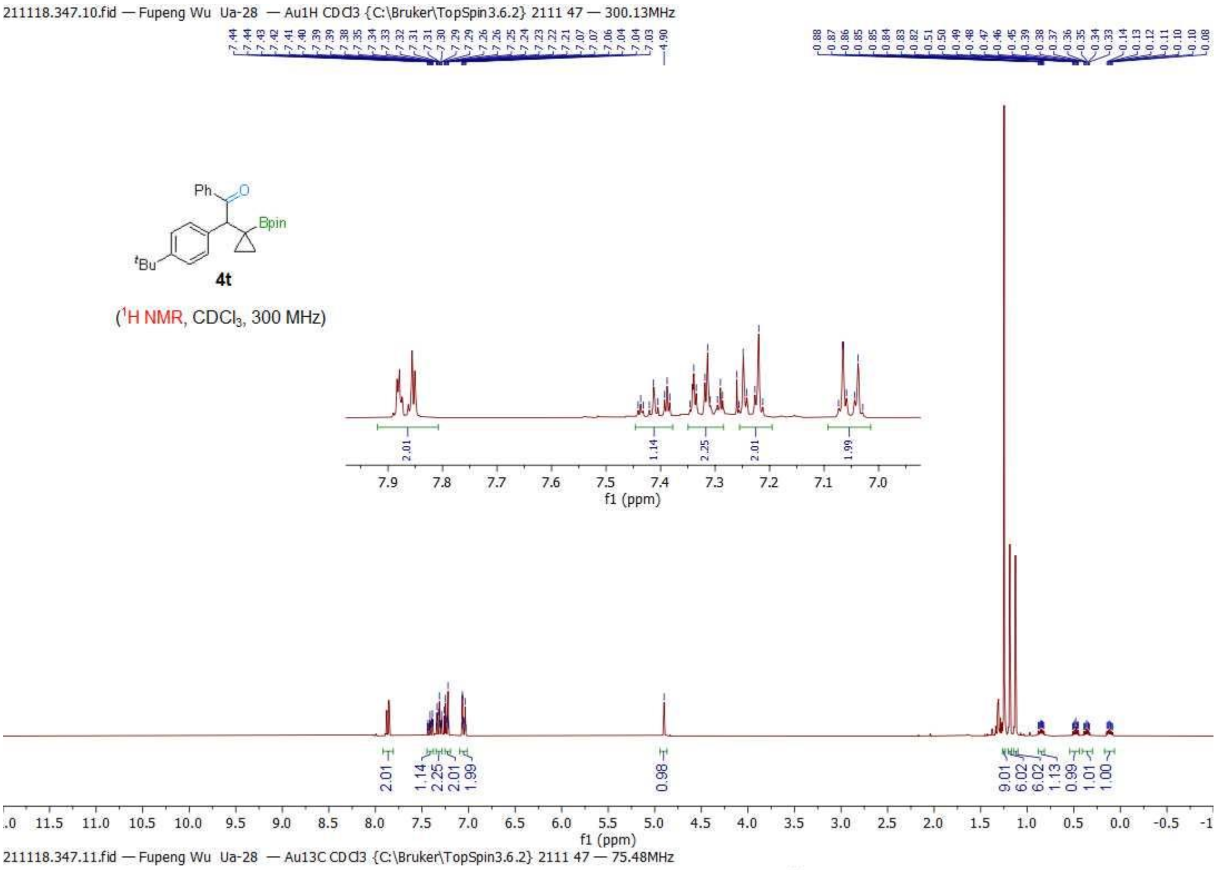
211118.347.10.fid — Fupeng Wu Ua-28 — Au1H CDCl3 {C:\Bruker\TopSpin3.6.2} 2111 47 — 300.13MHz
7.44 7.44 7.43 7.42 7.41 7.40 7.39 7.39 7.38 7.35 7.34 7.33 7.32 7.31 7.30 7.29 7.29 7.26 7.26 7.25 7.24 7.23 7.22 7.21 7.07 7.07 7.06 7.04 7.03 4.90
0.88 0.87 0.86 0.85 0.85 0.84 0.83 0.82 0.51 0.50 0.49 0.48 0.47 0.46 0.45 0.39 0.38 0.37 0.36 0.35 0.34 0.33 0.14 0.13 0.12 0.11 0.10 0.08
Ph
O
Bpin
tBu
4t
(1H NMR, CDCl3, 300 MHz)
2.01 1.14 2.25 2.01 1.99
2.01 1.14 2.25 2.01 1.99 0.98 9.01 6.02 6.02 1.13 0.99 1.01 1.00
f1 (ppm)

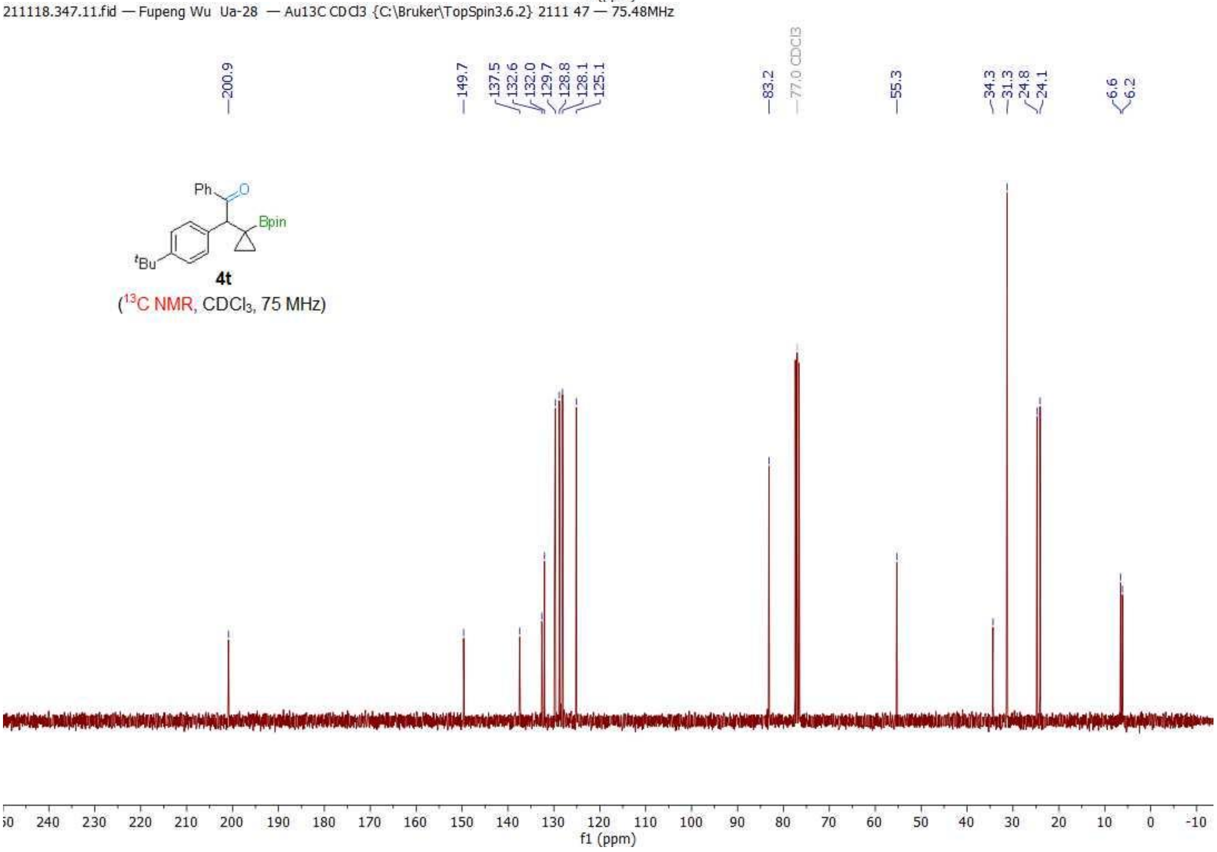
211118.347.11.fid — Fupeng Wu Ua-28 — Au13C CDCl3 {C:\Bruker\TopSpin3.6.2} 2111 47 — 75.48MHz
200.9
149.7
137.5
132.6
132.0
129.7
128.8
128.1
125.1
83.2
77.0 CDCl3
55.3
34.3
31.3
24.8
24.1
6.6
6.2
Ph
O
Bpin
tBu
4t
(13C NMR, CDCl3, 75 MHz)
f1 (ppm)

211118.347.12.fid — Fupeng Wu Ua-28 — Au11B CDCl3 {C:\Bruker\TopSpin3.6.2} 2111 47 — 96.29MHz

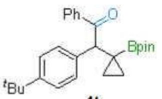

4t

(^{11}B NMR, $CDCl_3$, 96 MHz)

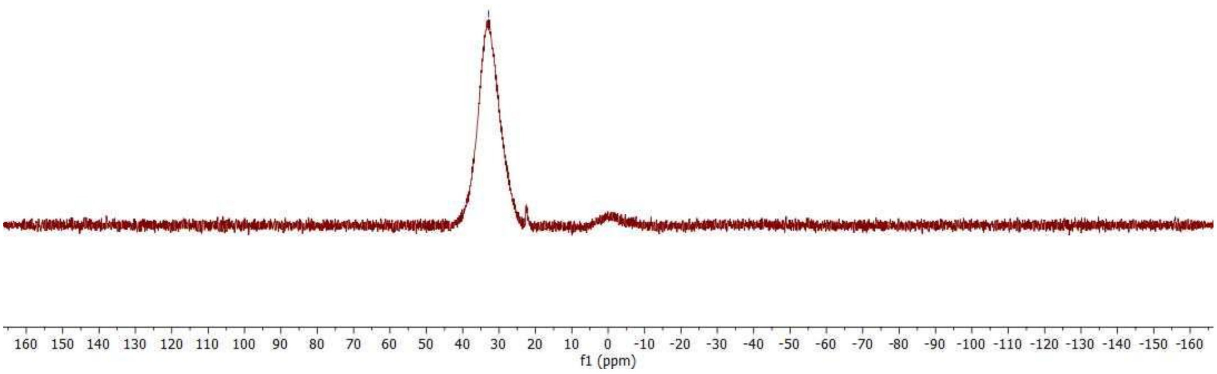

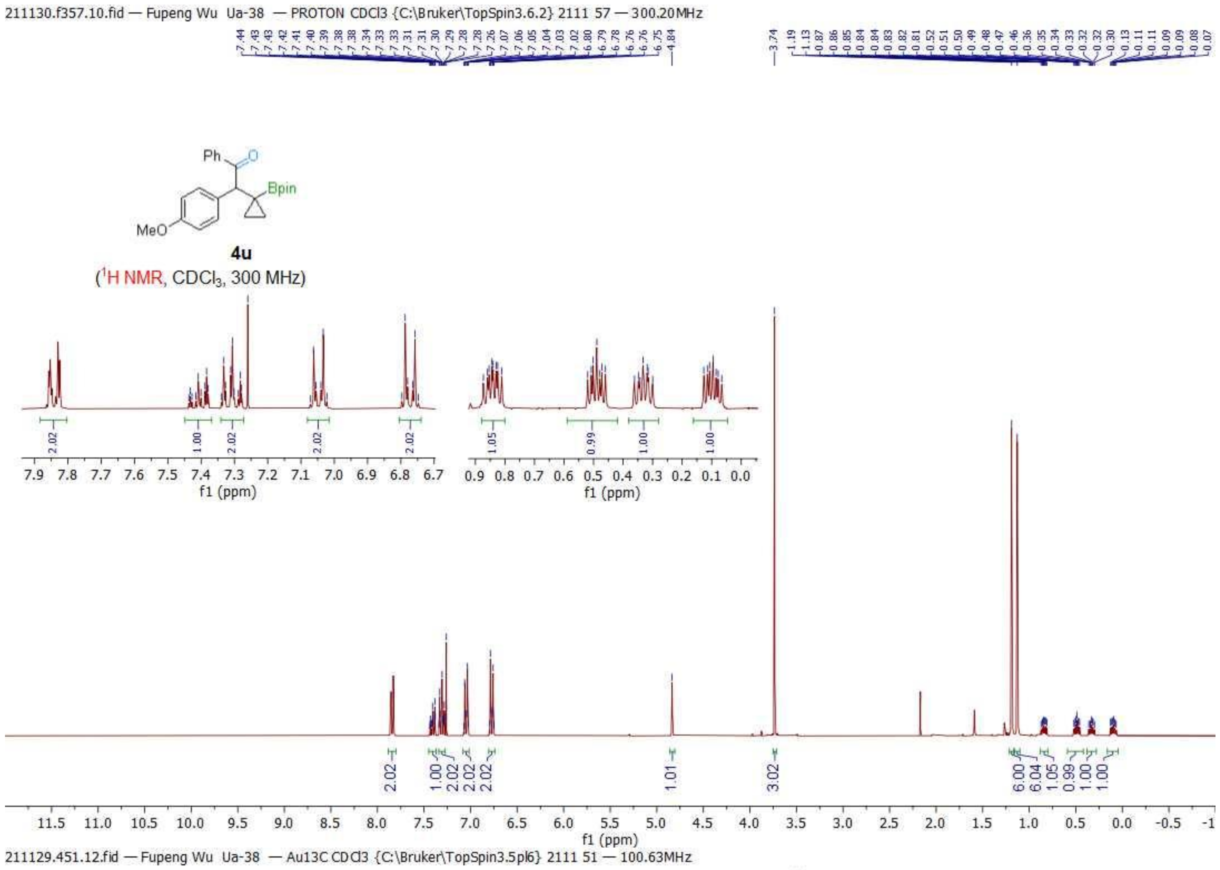
211130.f357.10.fid — Fupeng Wu Ua-38 — PROTON CDCl3 {C:\Bruker\TopSpin3.6.2} 2111 57 — 300.20MHz
Ph
O
Bpin
MeO
4u
(1H NMR, CDCl3, 300 MHz)
f1 (ppm)

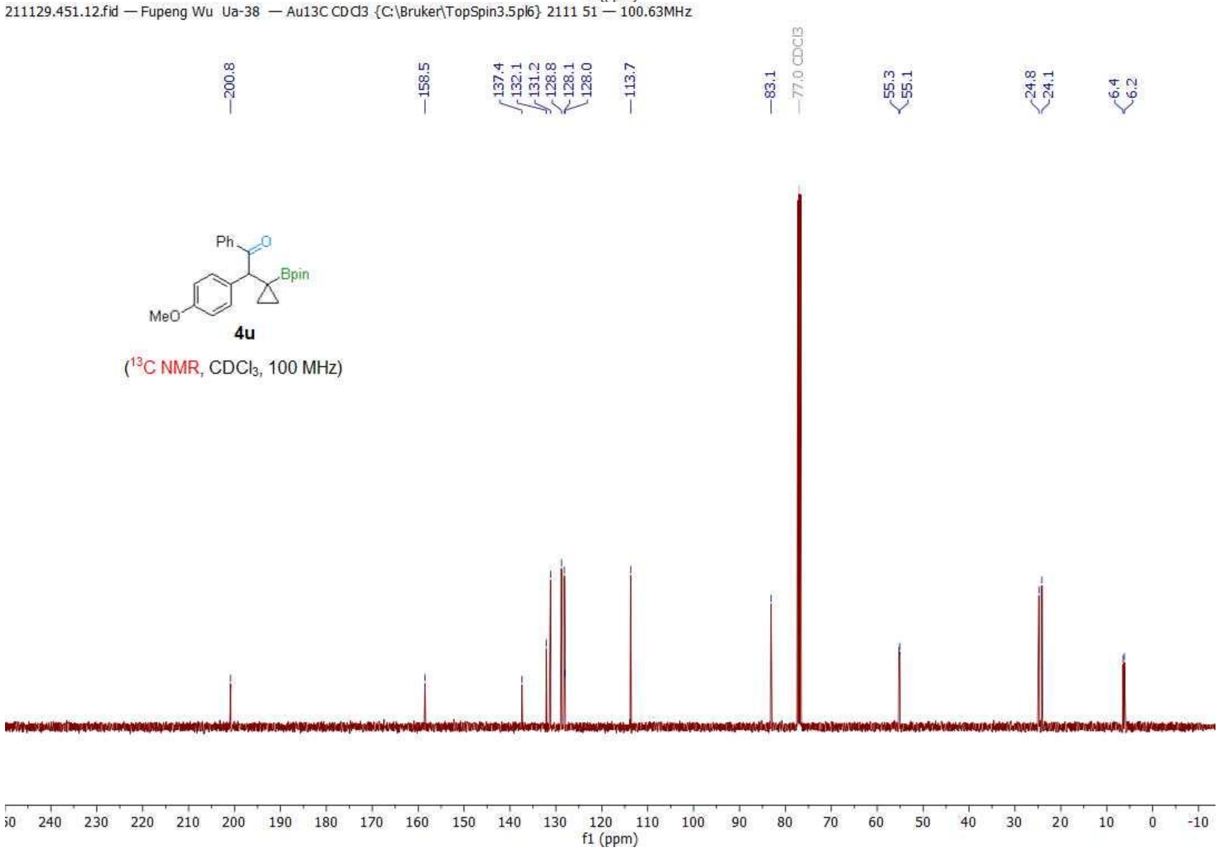
211129.451.12.fid — Fupeng Wu Ua-38 — Au13C CDCl3 {C:\Bruker\TopSpin3.5pl6} 2111 51 — 100.63MHz
200.8
158.5
137.4
132.1
131.2
128.8
128.1
128.0
113.7
83.1
77.0 CDCl3
55.3
55.1
24.8
24.1
6.4
6.2
Ph
O
Bpin
MeO
4u
(13C NMR, CDCl3, 100 MHz)
f1 (ppm)

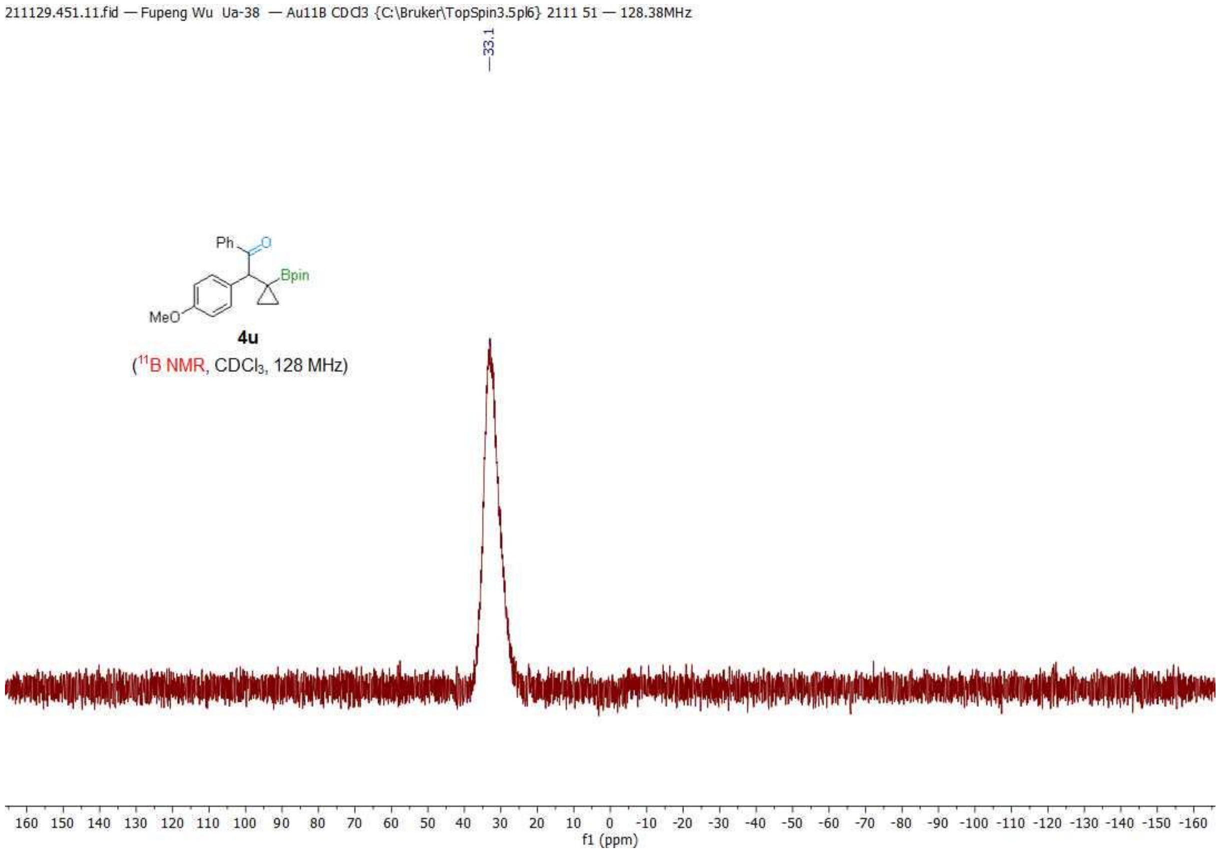
211129.451.11.fid — Fupeng Wu Ua-38 — Au11B CDCl3 {C:\Bruker\TopSpin3.5pl6} 2111 51 — 128.38MHz
—33.1
Ph
O
Bpin
MeO
4u
(11B NMR, CDCl3, 128 MHz)
160 150 140 130 120 110 100 90 80 70 60 50 40 30 20 10 0 -10 -20 -30 -40 -50 -60 -70 -80 -90 -100 -110 -120 -130 -140 -150 -160
f1 (ppm)

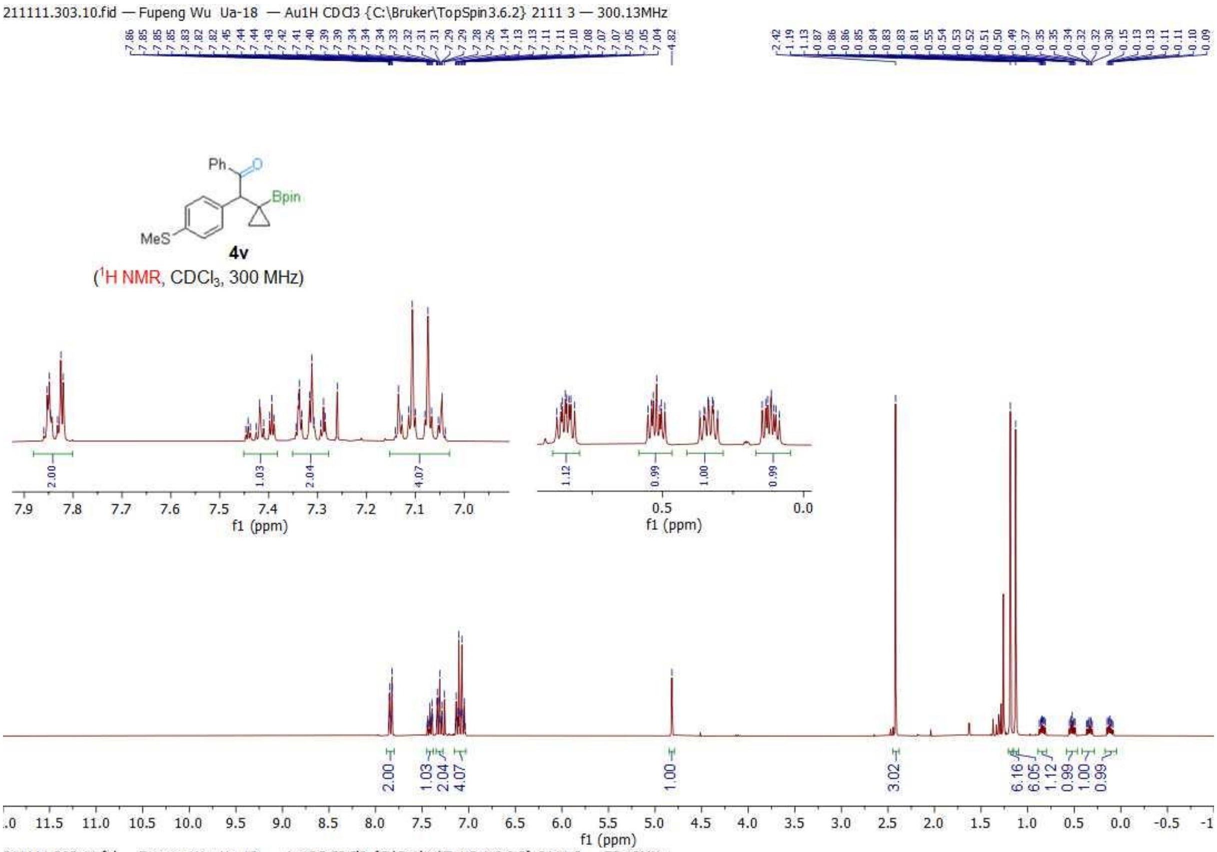
211111.303.10.fid — Fupeng Wu Ua-18 — Au1H CDCl3 {C:\Bruker\TopSpin3.6.2} 2111 3 — 300.13MHz
7.86 7.85 7.85 7.83 7.83 7.82 7.45 7.44 7.43 7.42 7.41 7.40 7.39 7.39 7.34 7.34 7.33 7.32 7.31 7.31 7.29 7.29 7.28 7.26 7.14 7.13 7.13 7.11 7.11 7.10 7.08 7.07 7.07 7.05 7.04 4.82
2.42 1.19 1.13 0.87 0.86 0.86 0.85 0.84 0.83 0.83 0.81 0.55 0.54 0.53 0.52 0.51 0.50 0.49 0.37 0.35 0.35 0.34 0.32 0.32 0.30 0.15 0.13 0.13 0.11 0.10 0.09
Ph
O
Bpin
MeS
4v
(1H NMR, CDCl3, 300 MHz)
2.00 1.03 2.04 4.07 1.00 3.02 6.16 6.05 1.12 0.99 1.00 0.99
f1 (ppm)

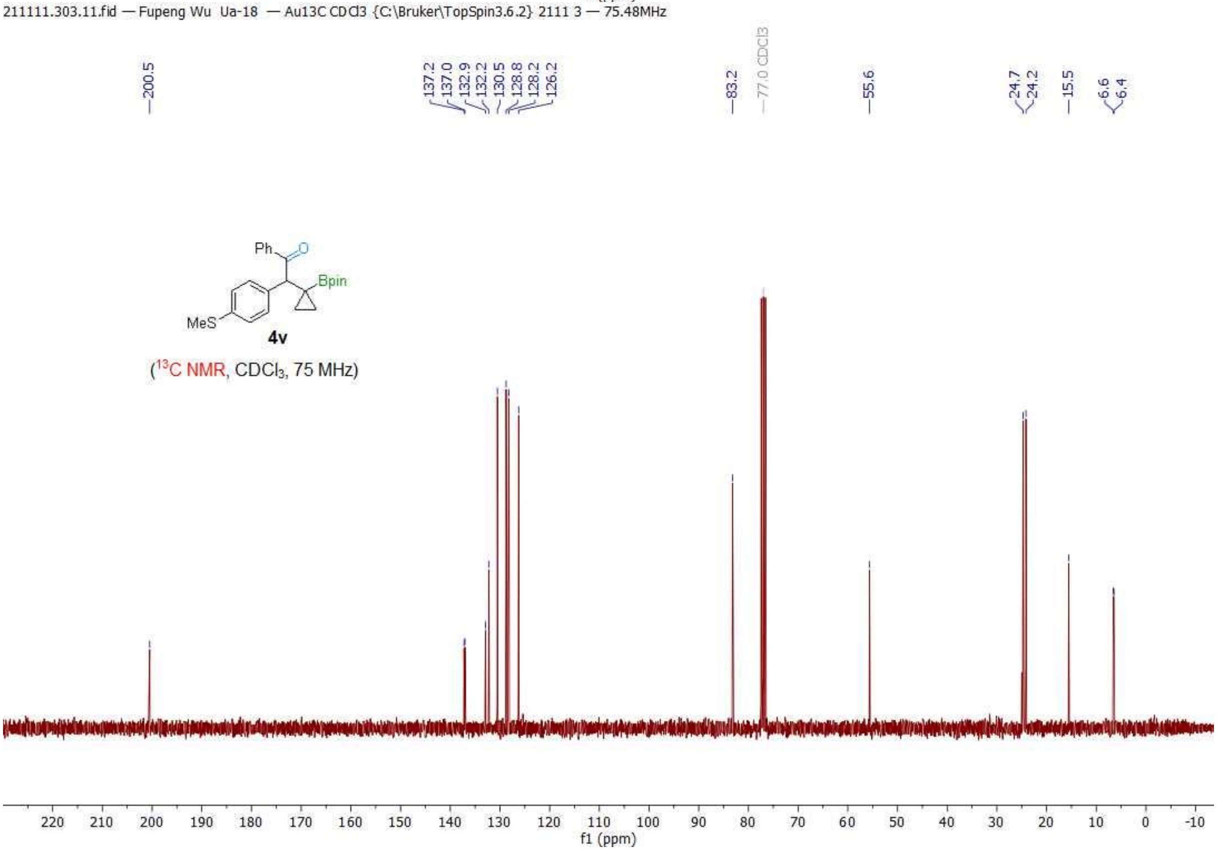
211111.303.11.fid — Fupeng Wu Ua-18 — Au13C CDCl3 {C:\Bruker\TopSpin3.6.2} 2111 3 — 75.48MHz
200.5
137.2 137.0 132.9 132.2 130.5 128.8 128.2 126.2
83.2
77.0 CDCl3
55.6
24.7 24.2
15.5
6.6 6.4
Ph
O
Bpin
MeS
4v
(13C NMR, CDCl3, 75 MHz)
f1 (ppm)

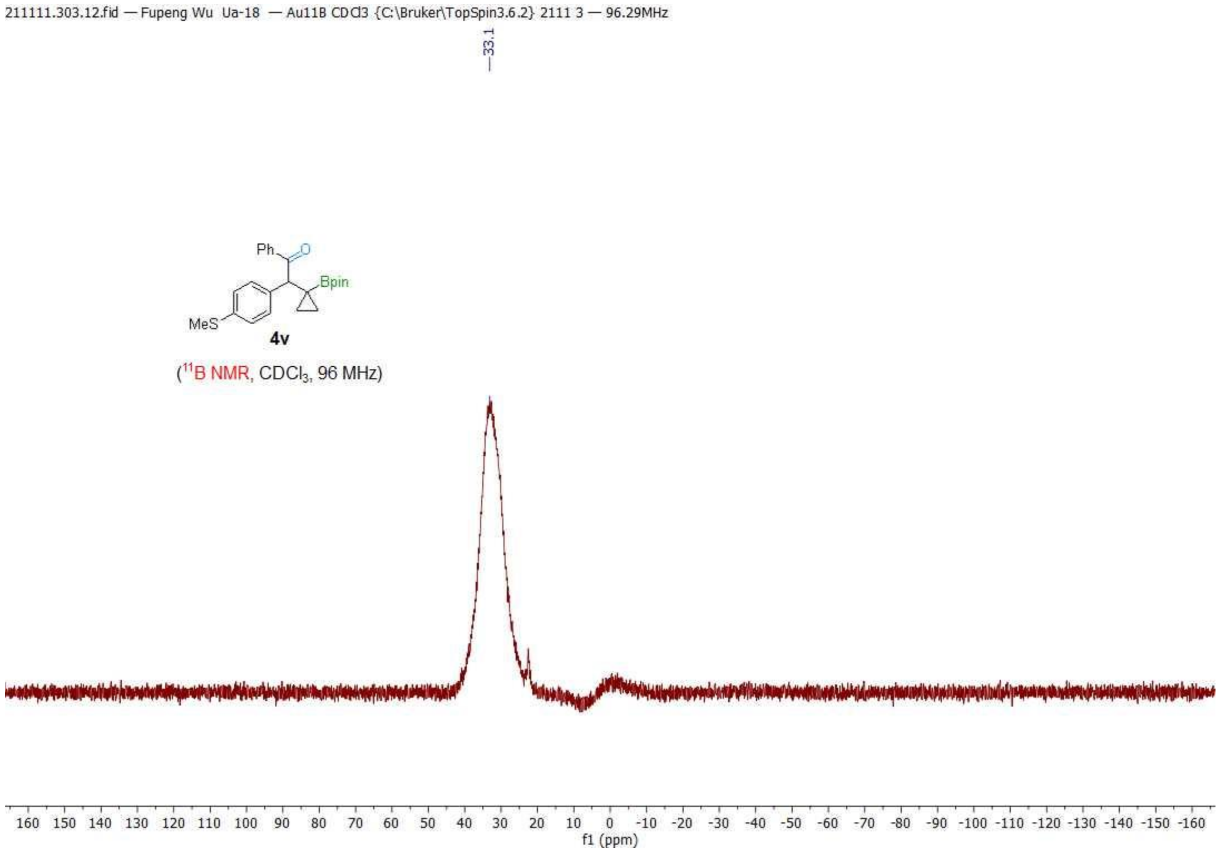
211111.303.12.fid — Fupeng Wu Ua-18 — Au11B CDCl3 {C:\Bruker\TopSpin3.6.2} 2111 3 — 96.29MHz
33.1
Ph
O
Bpin
MeS
4v
(11B NMR, CDCl3, 96 MHz)
160 150 140 130 120 110 100 90 80 70 60 50 40 30 20 10 0 -10 -20 -30 -40 -50 -60 -70 -80 -90 -100 -110 -120 -130 -140 -150 -160
f1 (ppm)

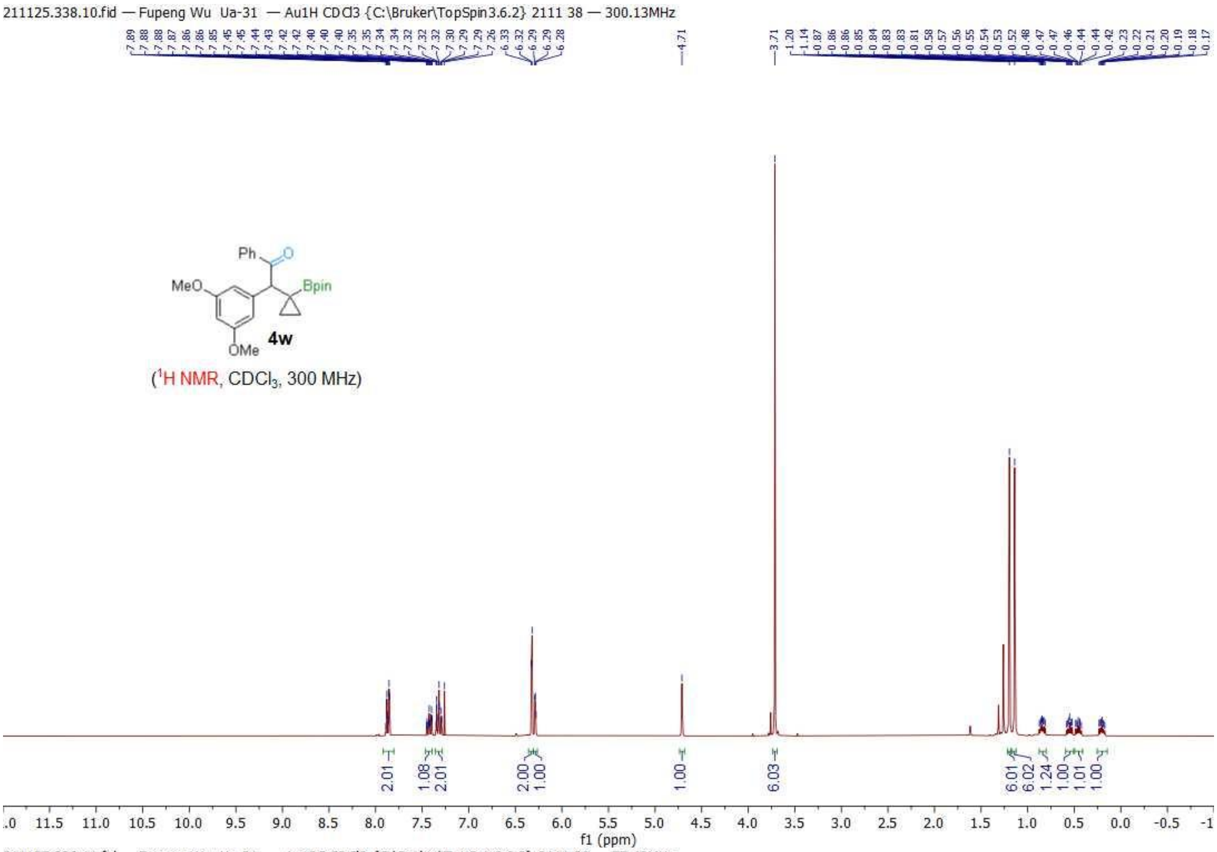
211125.338.10.fid — Fupeng Wu Ua-31 — Au1H CDCl3 {C:\Bruker\TopSpin3.6.2} 2111 38 — 300.13MHz
4.71
3.71
Ph
O
MeO
Bpin
OMe
4w
(1H NMR, CDCl3, 300 MHz)
2.01
1.08
2.01
2.00
1.00
1.00
6.03
6.01
6.02
1.24
1.00
1.01
1.00
f1 (ppm)

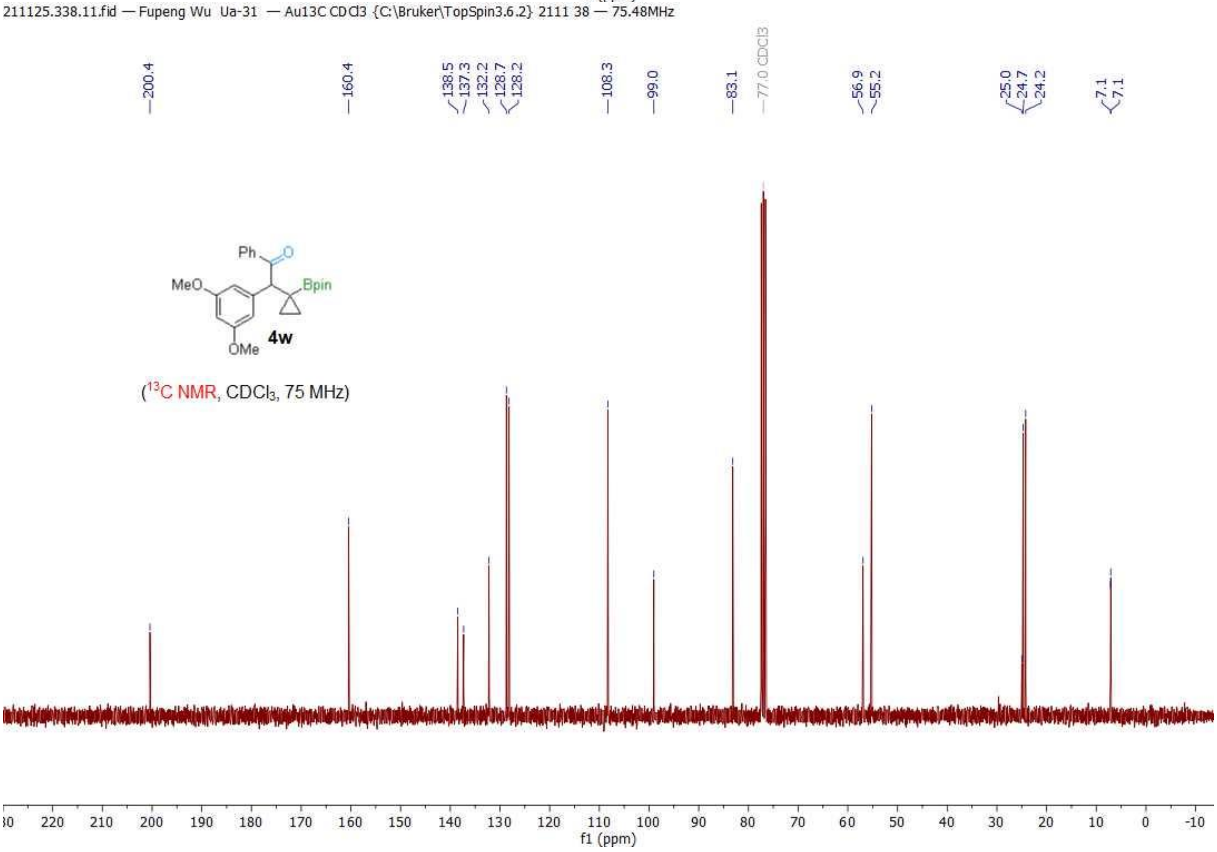
211125.338.11.fid — Fupeng Wu Ua-31 — Au13C CDCl3 {C:\Bruker\TopSpin3.6.2} 2111 38 — 75.48MHz
200.4
160.4
138.5
137.3
132.2
128.7
128.2
108.3
99.0
83.1
77.0 CDCl3
56.9
55.2
25.0
24.7
24.2
7.1
7.1
Ph
O
MeO
Bpin
OMe
4w
(13C NMR, CDCl3, 75 MHz)
f1 (ppm)

211125.338.12.fid — Fupeng Wu Ua-31 — Au11B CDCl3 {C:\Bruker\TopSpin3.6.2} 2111 38 — 96.29MHz

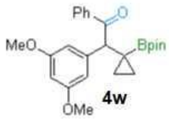

4w

(^{11}B NMR, $CDCl_3$, 96 MHz)

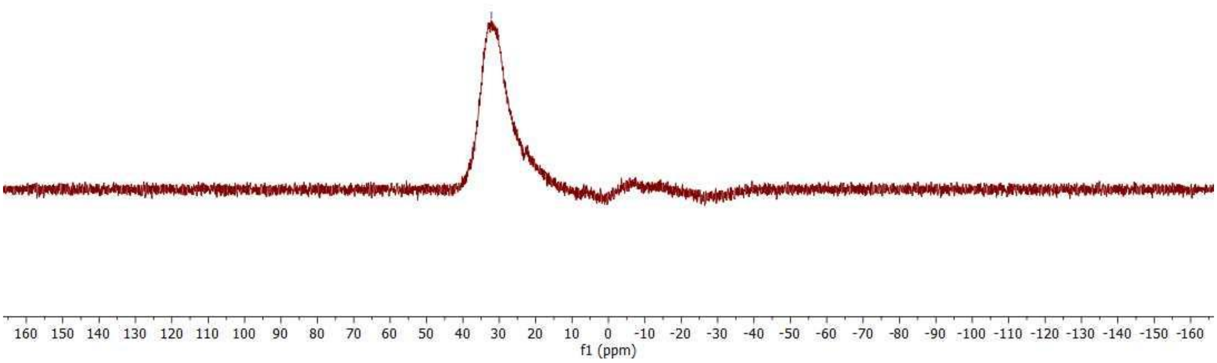

211130.f356.10.fid — Fupeng Wu Ua-36 — PROTON CDCl3 {C:\Bruker\TopSpin3.6.2} 2111 56 — 300.20MHz

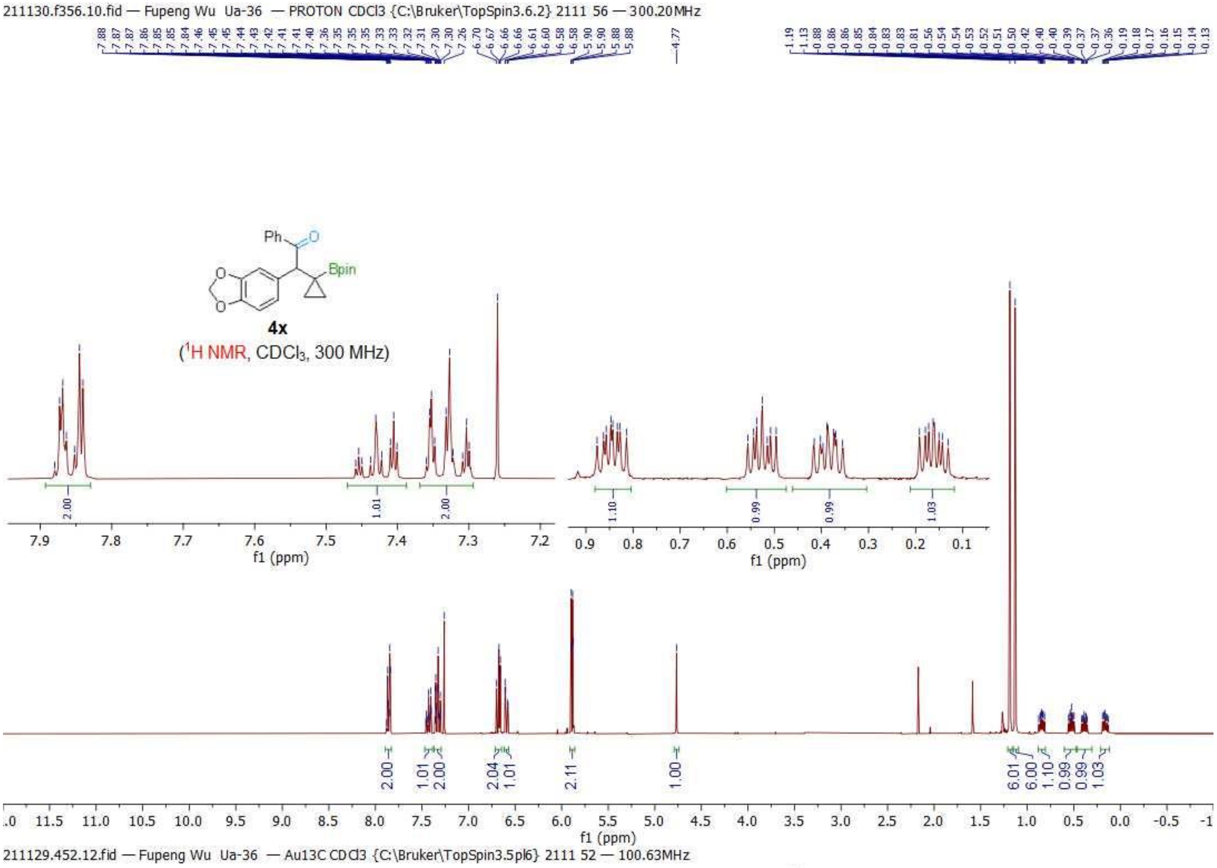

211129.452.12.fid — Fupeng Wu Ua-36 — Au13C CDCl3 {C:\Bruker\TopSpin3.5pl6} 2111 52 — 100.63MHz

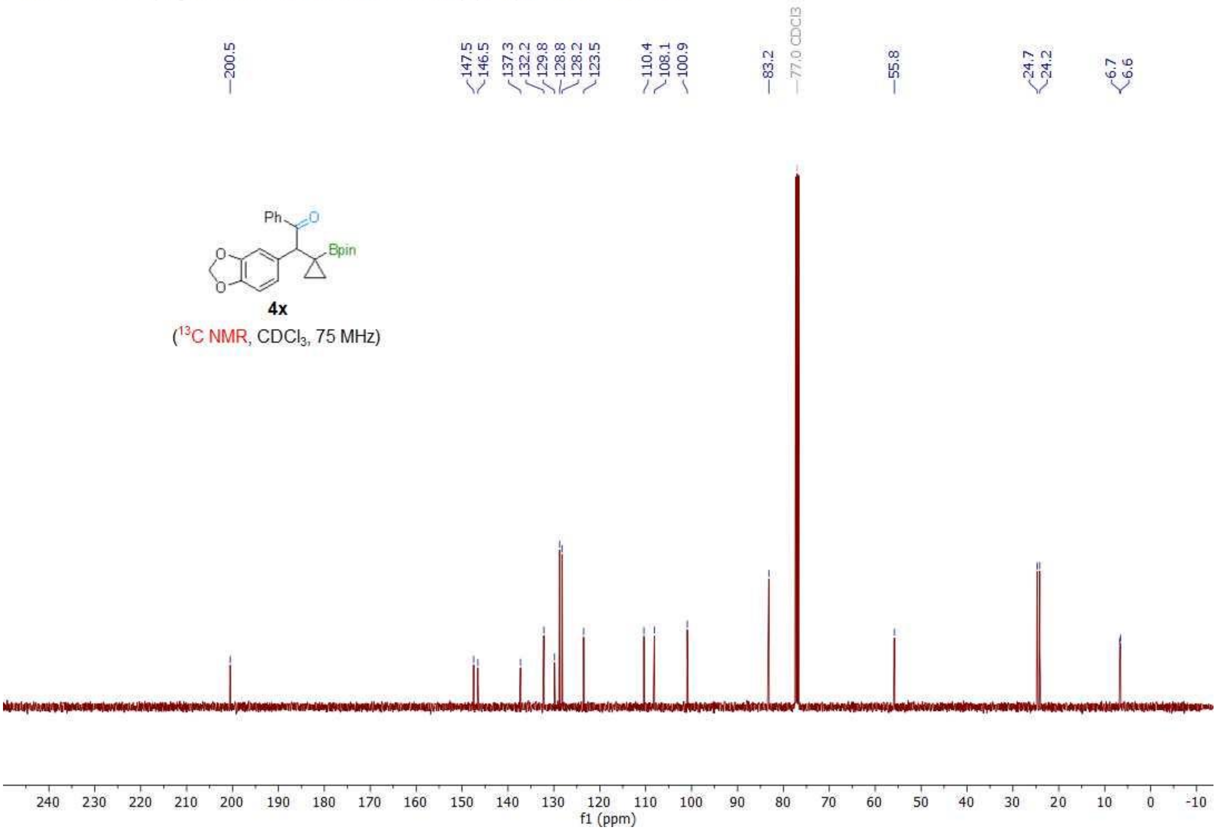

211129.452.11.fid — Fupeng Wu Ua-36 — Au11B CDCl3 {C:\Bruker\TopSpin3.5pl6} 2111 52 — 128.38MHz

32.9

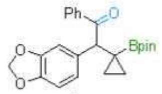

4x

(^{11}B NMR, CDCl$_3$, 96 MHz)

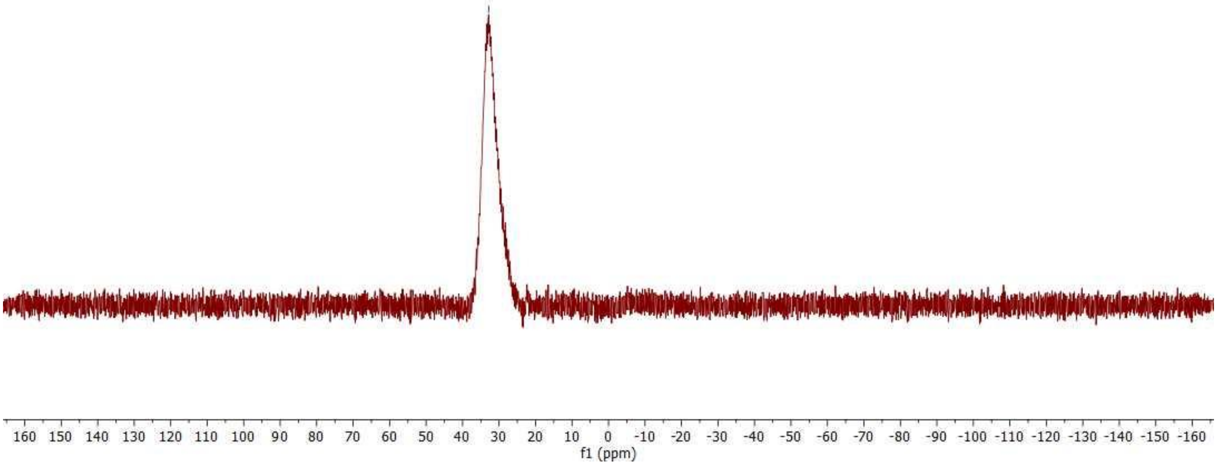

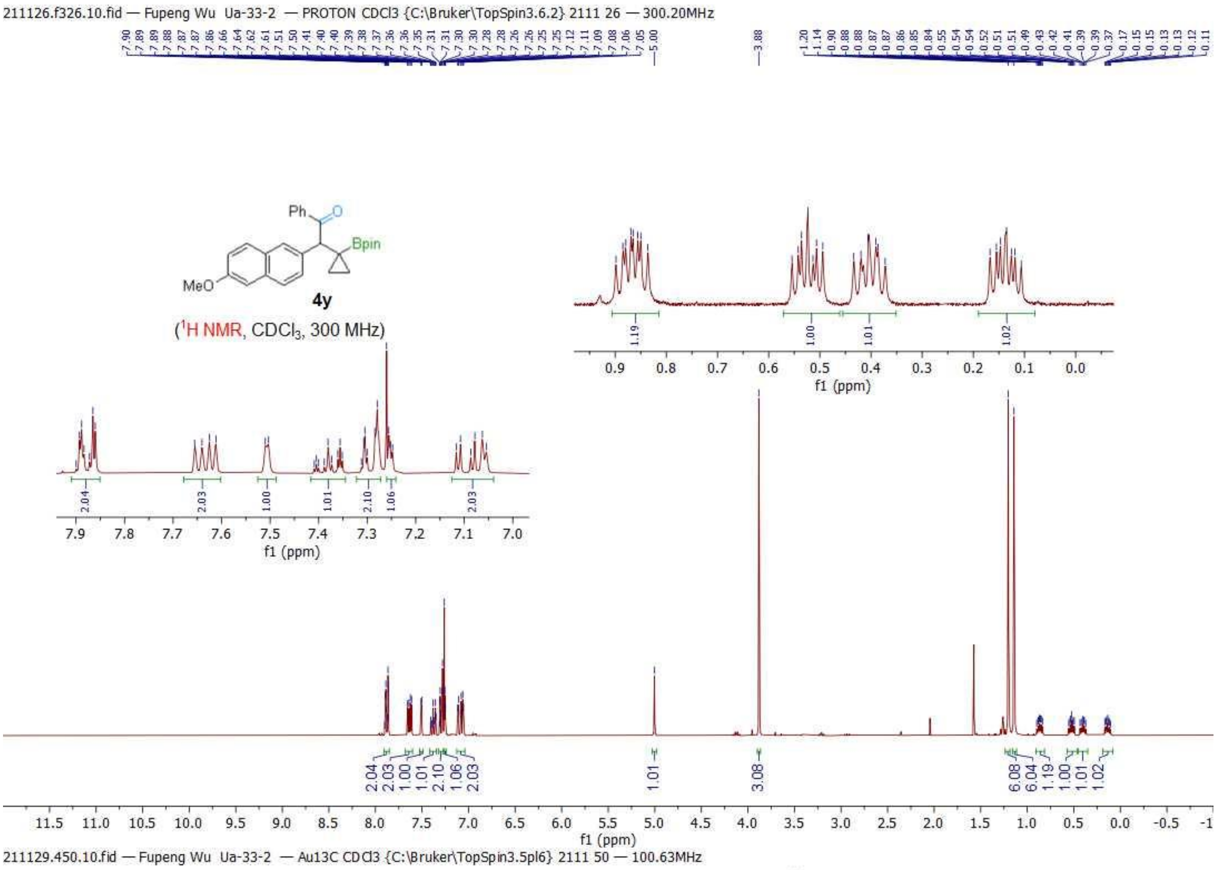
211126.f326.10.fid — Fupeng Wu Ua-33-2 — PROTON CDCl3 {C:\Bruker\TopSpin3.6.2} 2111 26 — 300.20MHz
Ph
O
Bpin
MeO
4y
(1H NMR, CDCl3, 300 MHz)
f1 (ppm)

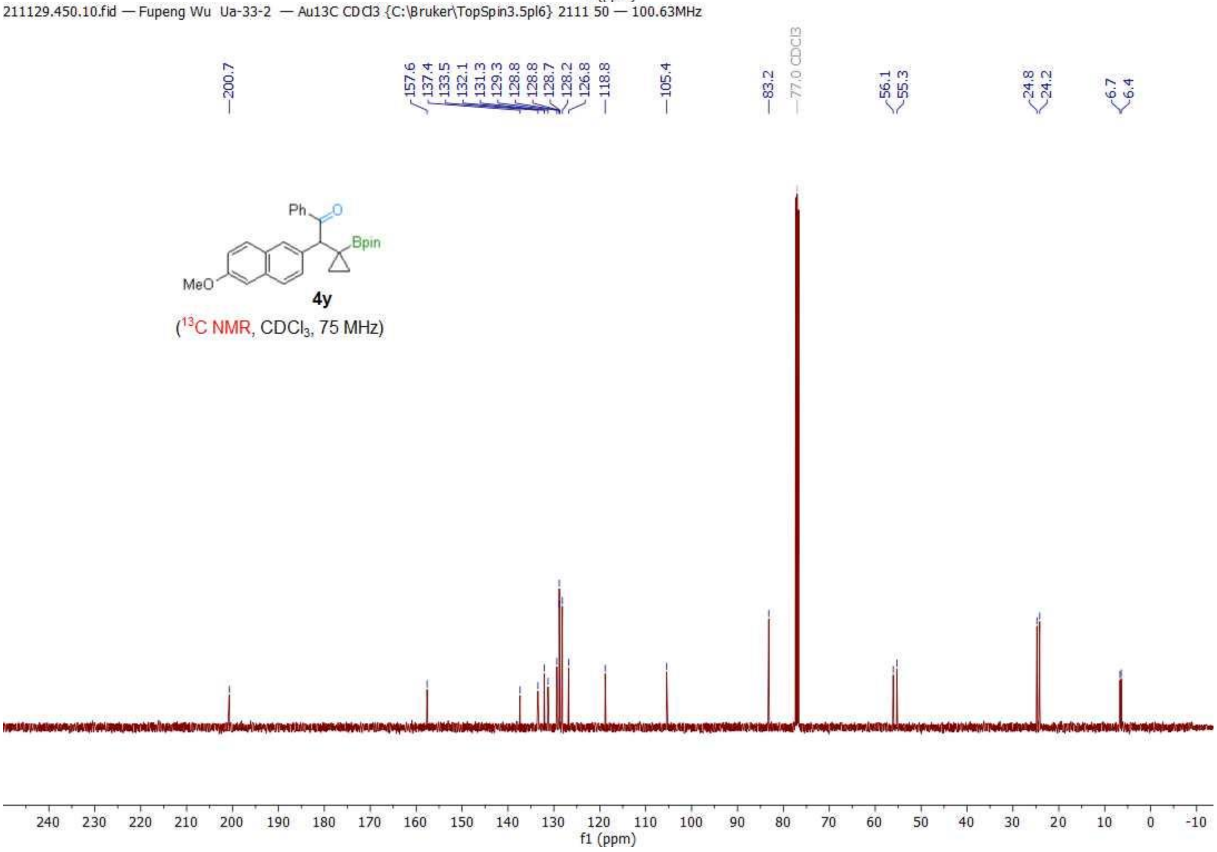
211129.450.10.fid — Fupeng Wu Ua-33-2 — Au13C CDCl3 {C:\Bruker\TopSpin3.5pl6} 2111 50 — 100.63MHz
200.7
157.6
137.4
133.5
132.1
131.3
129.3
128.8
128.8
128.7
128.2
126.8
118.8
105.4
83.2
77.0 CDCl3
56.1
55.3
24.8
24.2
6.7
6.4
Ph
O
Bpin
MeO
4y
(13C NMR, CDCl3, 75 MHz)
f1 (ppm)

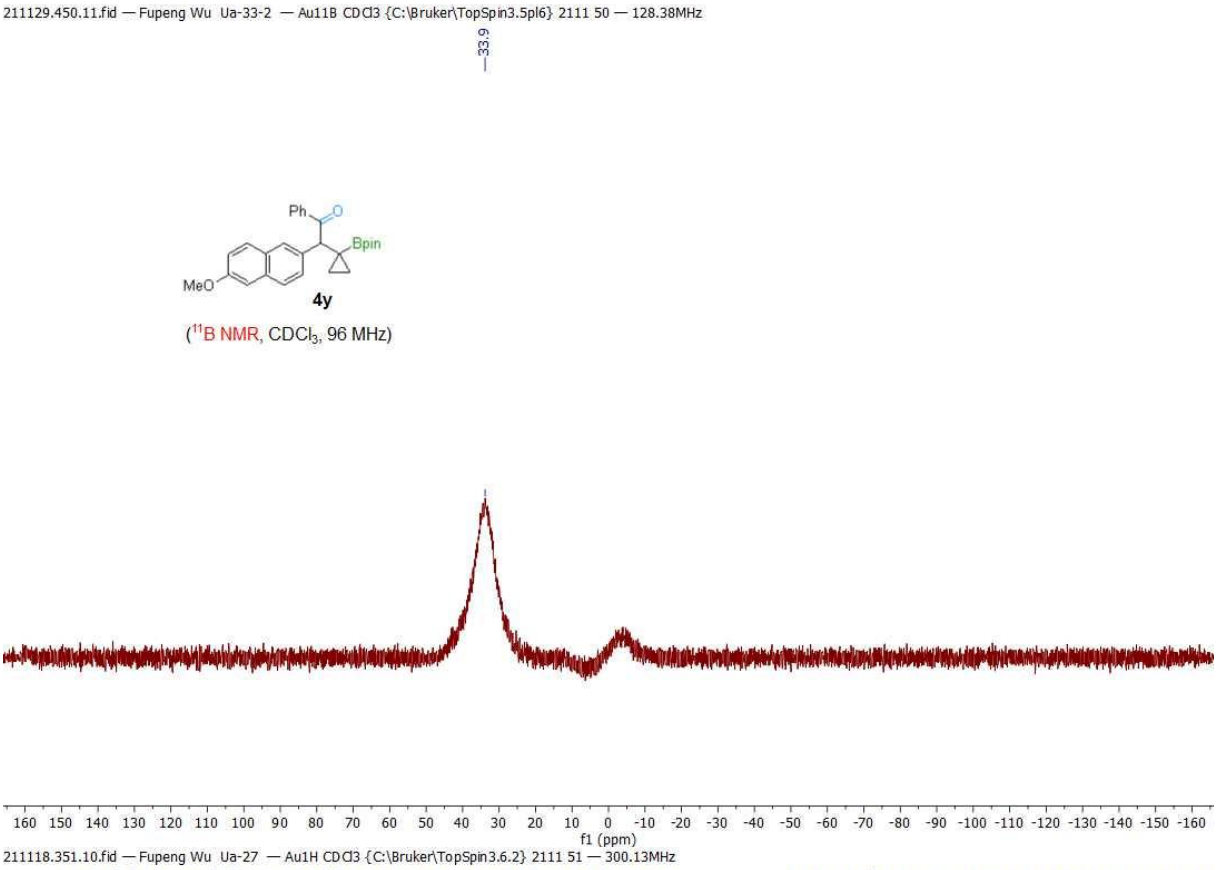

211129.450.11.fid — Fupeng Wu Ua-33-2 — Au11B CDCl3 {C:\Bruker\TopSpin3.5pl6} 2111 50 — 128.38MHz
33.9
Ph
O
Bpin
MeO
4y
(11B NMR, CDCl3, 96 MHz)
f1 (ppm)

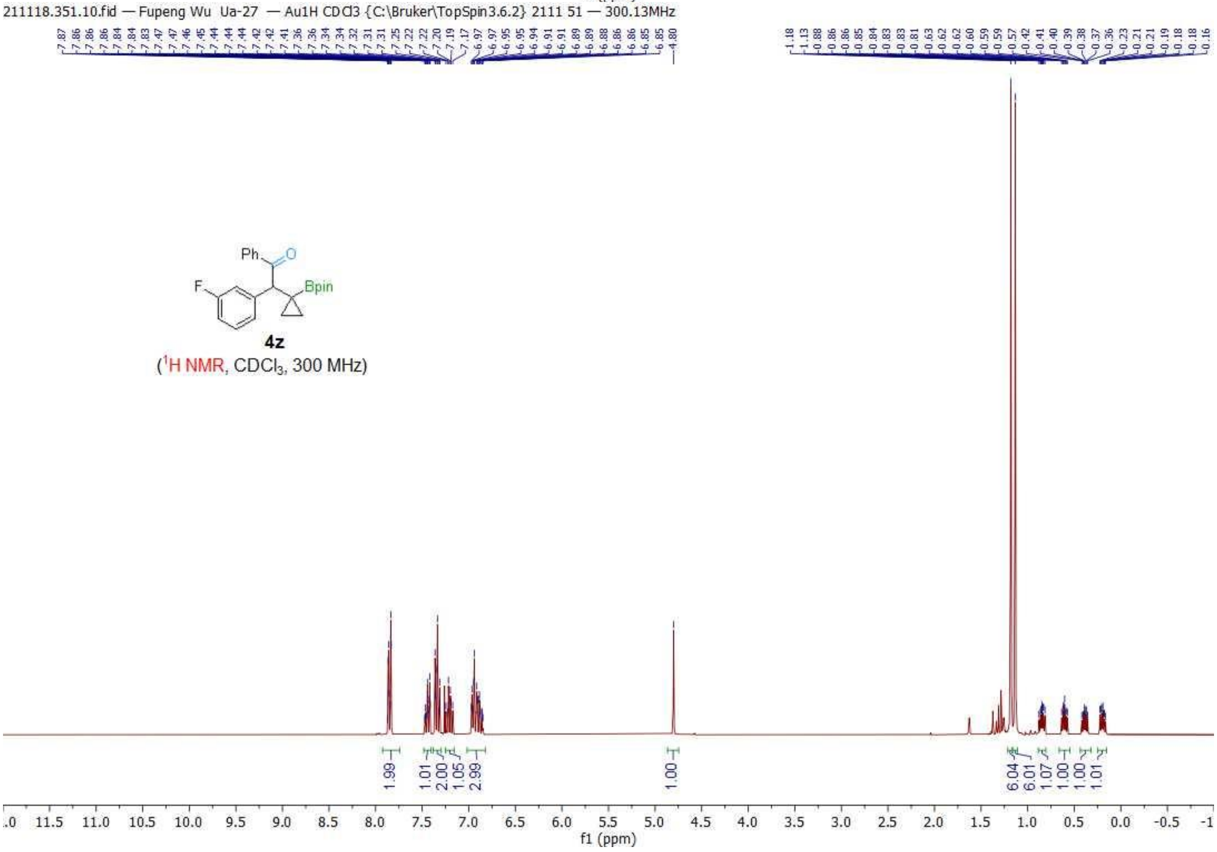

211118.351.10.fid — Fupeng Wu Ua-27 — Au1H CDCl3 {C:\Bruker\TopSpin3.6.2} 2111 51 — 300.13MHz
Ph
O
F
Bpin
4z
(1H NMR, CDCl3, 300 MHz)
1.99
1.01
2.00
1.05
2.99
1.00
6.04
6.01
1.07
1.00
1.00
1.01
f1 (ppm)

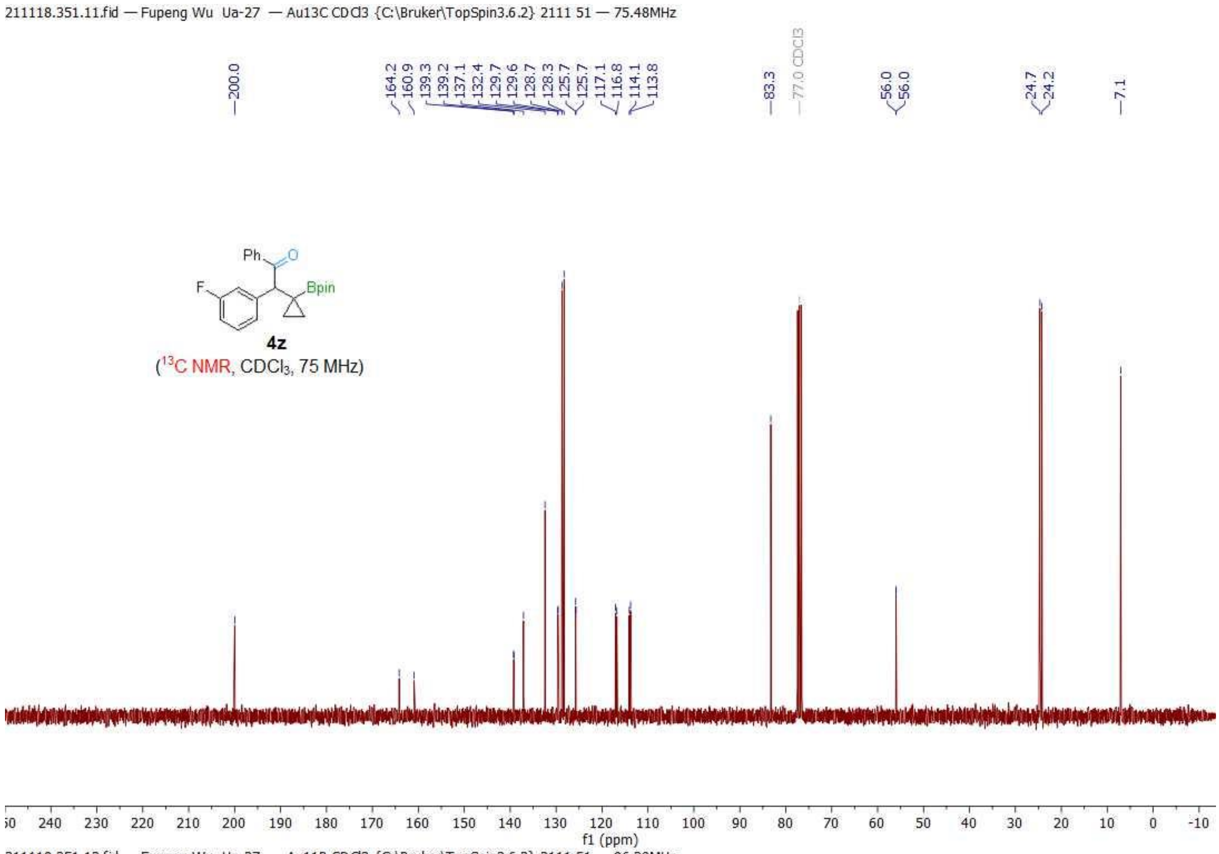
211118.351.11.fid — Fupeng Wu Ua-27 — Au13C CDCl3 {C:\Bruker\TopSpin3.6.2} 2111 51 — 75.48MHz
200.0
164.2
160.9
139.3
139.2
137.1
132.4
129.7
129.6
128.7
128.3
125.7
125.7
117.1
116.8
114.1
113.8
83.3
77.0 CDCl3
56.0
56.0
24.7
24.2
7.1
Ph
O
F
Bpin
4z
(13C NMR, CDCl3, 75 MHz)
f1 (ppm)

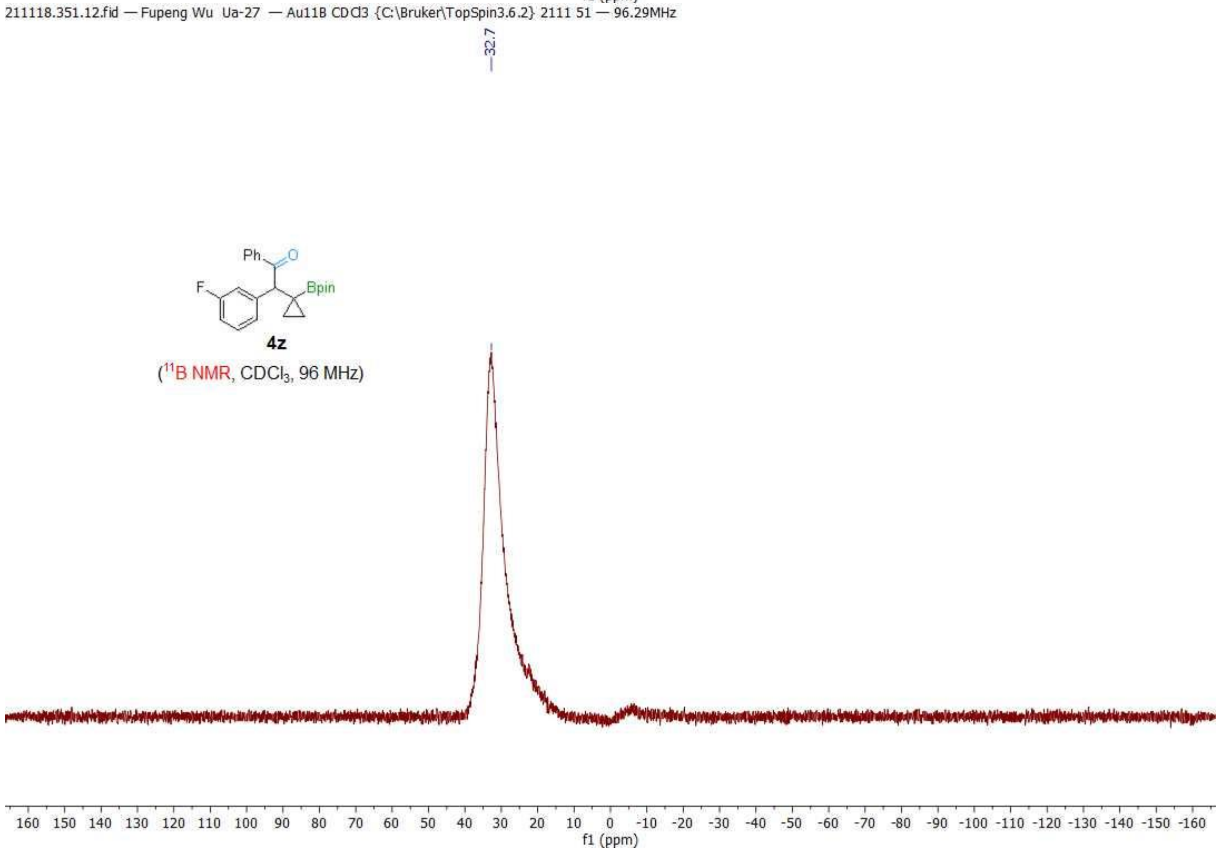
211118.351.12.fid — Fupeng Wu Ua-27 — Au11B CDCl3 {C:\Bruker\TopSpin3.6.2} 2111 51 — 96.29MHz
32.7
Ph
O
F
Bpin
4z
(11B NMR, CDCl3, 96 MHz)
f1 (ppm)

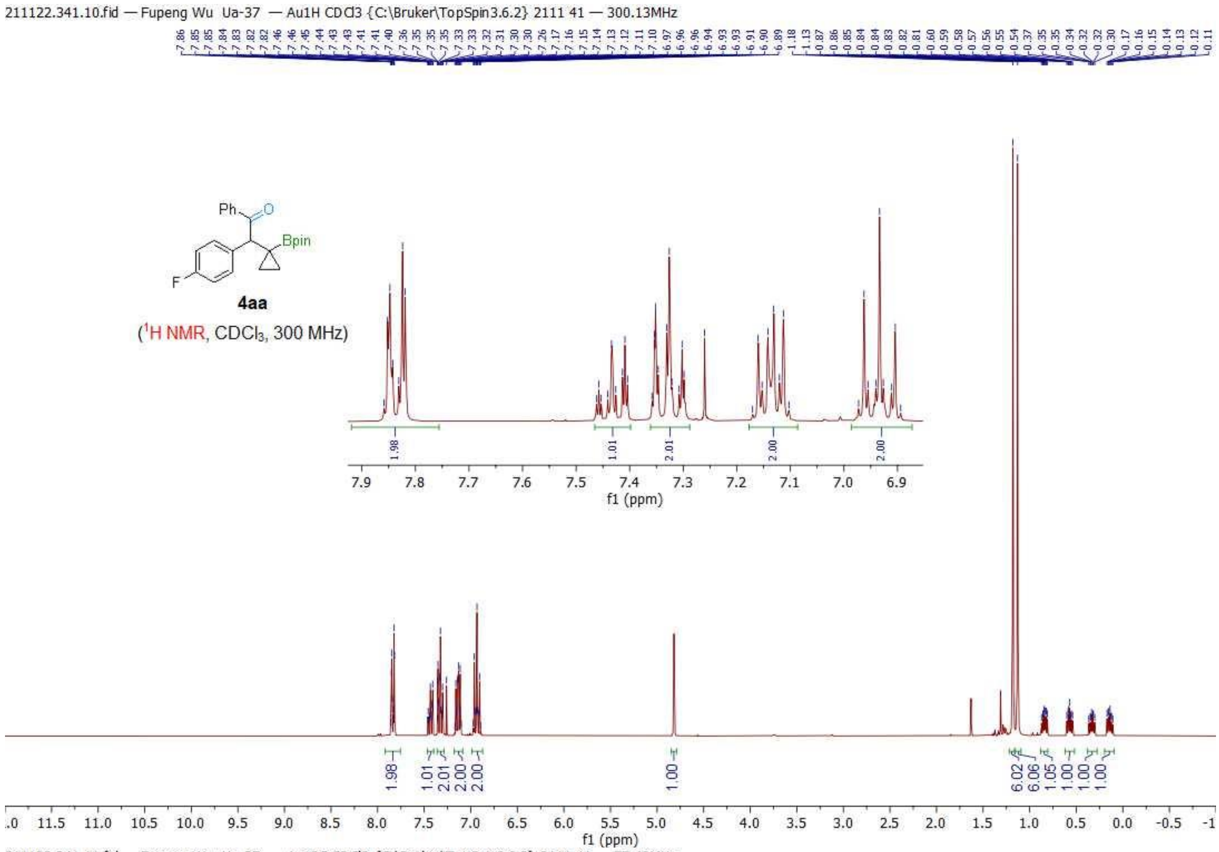
211122.341.10.fid — Fupeng Wu Ua-37 — Au1H CDCl3 {C:\Bruker\TopSpin3.6.2} 2111 41 — 300.13MHz
Ph
O
Bpin
F
4aa
(1H NMR, CDCl3, 300 MHz)
1.98
1.01
2.01
2.00
2.00
1.00
6.02
6.06
1.05
1.00
1.00
1.00
f1 (ppm)

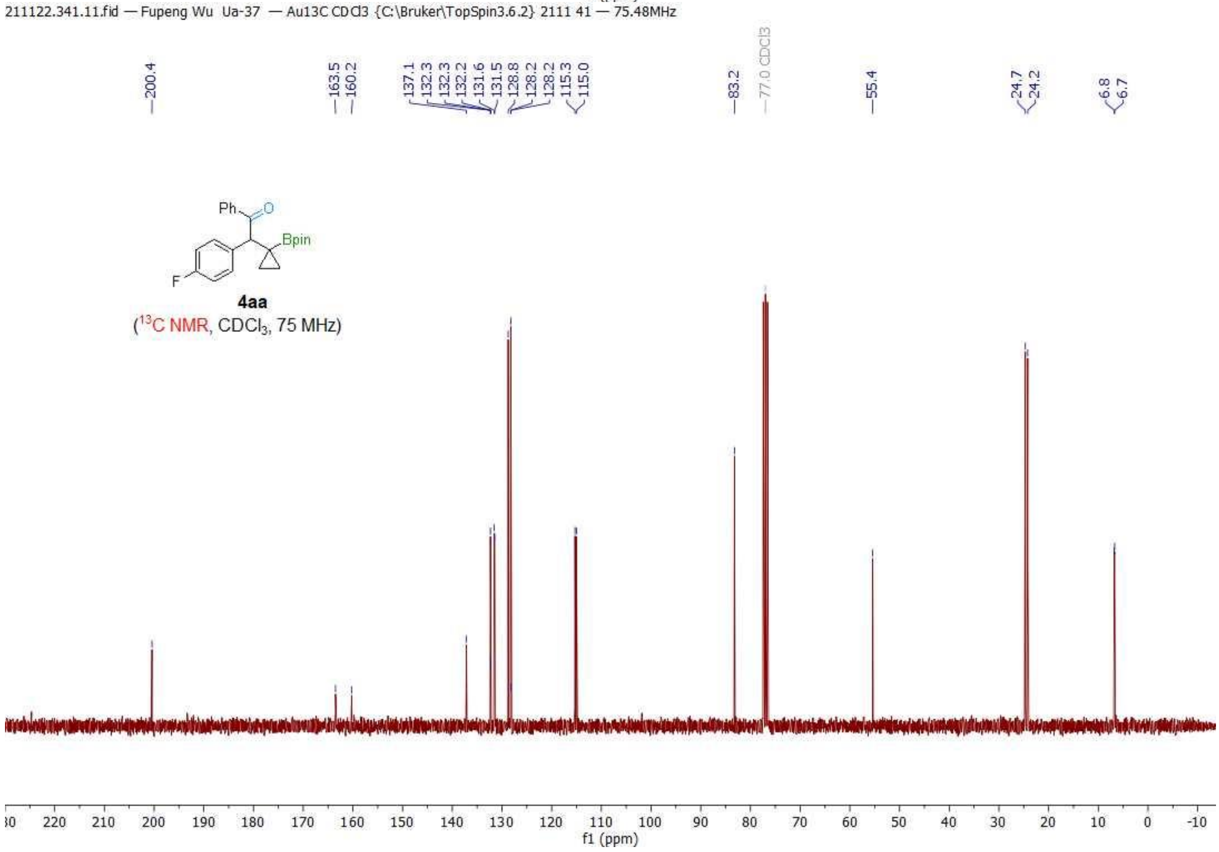
211122.341.11.fid — Fupeng Wu Ua-37 — Au13C CDCl3 {C:\Bruker\TopSpin3.6.2} 2111 41 — 75.48MHz
200.4
163.5
160.2
137.1
132.3
132.3
132.2
131.6
131.5
128.8
128.2
128.2
115.3
115.0
83.2
77.0 CDCl3
55.4
24.7
24.2
6.8
6.7
Ph
O
Bpin
F
4aa
(13C NMR, CDCl3, 75 MHz)
f1 (ppm)

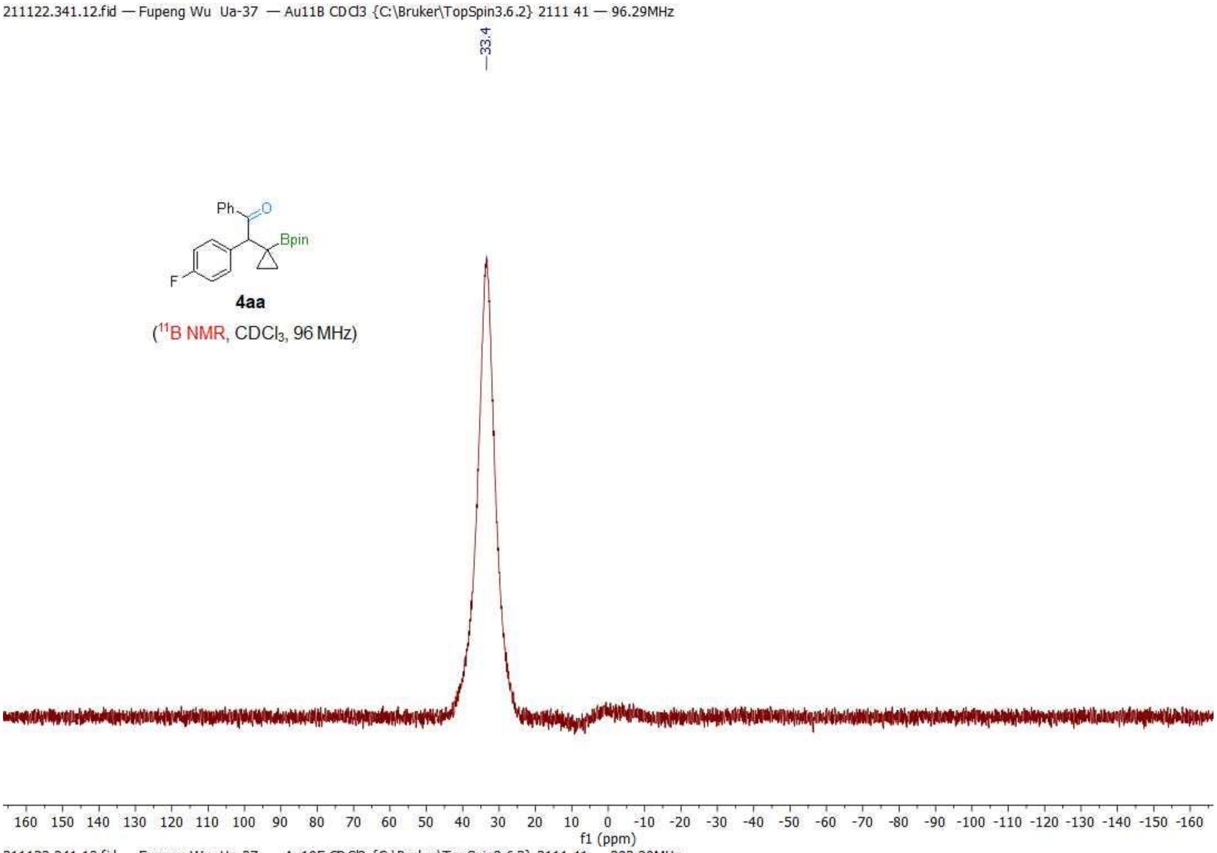
211122.341.12.fid — Fupeng Wu Ua-37 — Au11B CDCl3 {C:\Bruker\TopSpin3.6.2} 2111 41 — 96.29MHz
33.4
Ph
O
Bpin
F
4aa
(11B NMR, CDCl3, 96 MHz)
160 150 140 130 120 110 100 90 80 70 60 50 40 30 20 10 0 -10 -20 -30 -40 -50 -60 -70 -80 -90 -100 -110 -120 -130 -140 -150 -160
f1 (ppm)

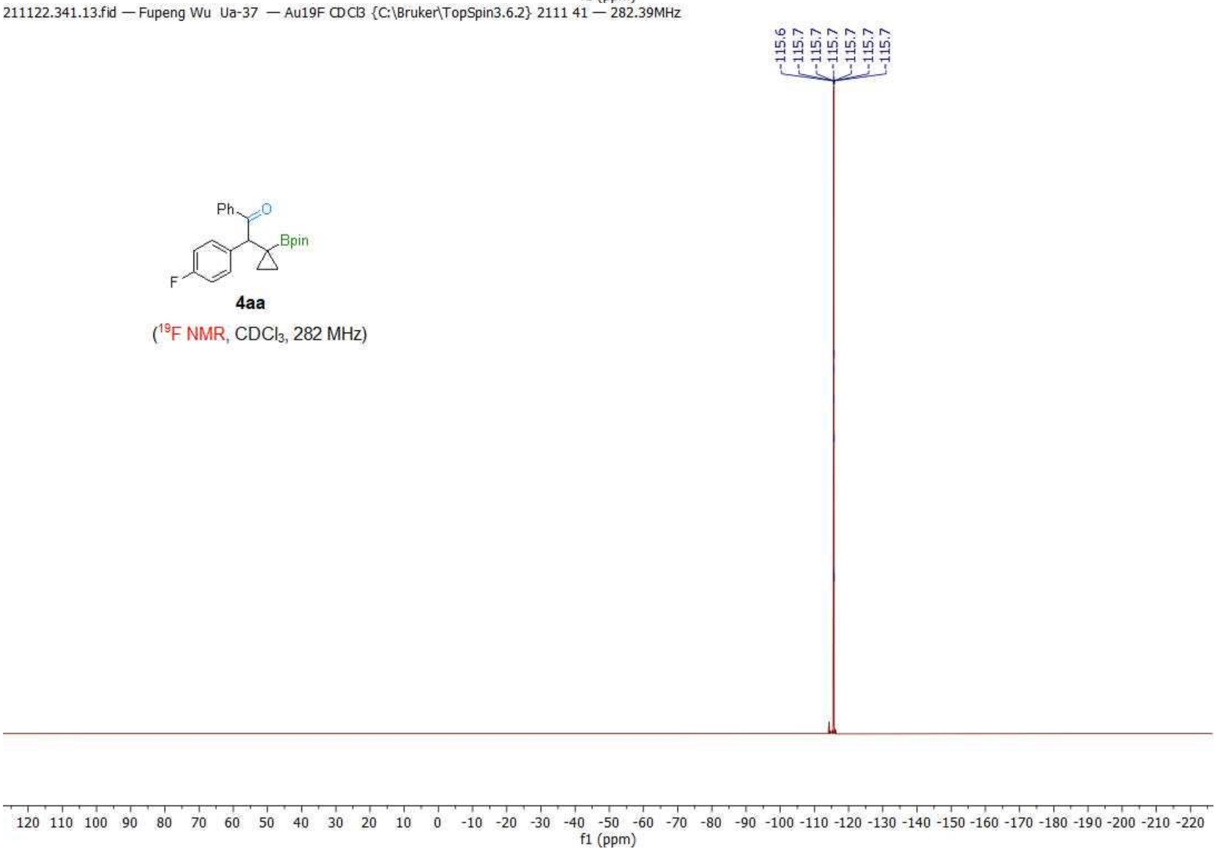
211122.341.13.fid — Fupeng Wu Ua-37 — Au19F CDCl3 {C:\Bruker\TopSpin3.6.2} 2111 41 — 282.39MHz
-115.6
-115.7
-115.7
-115.7
-115.7
-115.7
-115.7
Ph
O
Bpin
F
4aa
(19F NMR, CDCl3, 282 MHz)
120 110 100 90 80 70 60 50 40 30 20 10 0 -10 -20 -30 -40 -50 -60 -70 -80 -90 -100 -110 -120 -130 -140 -150 -160 -170 -180 -190 -200 -210 -220
f1 (ppm)

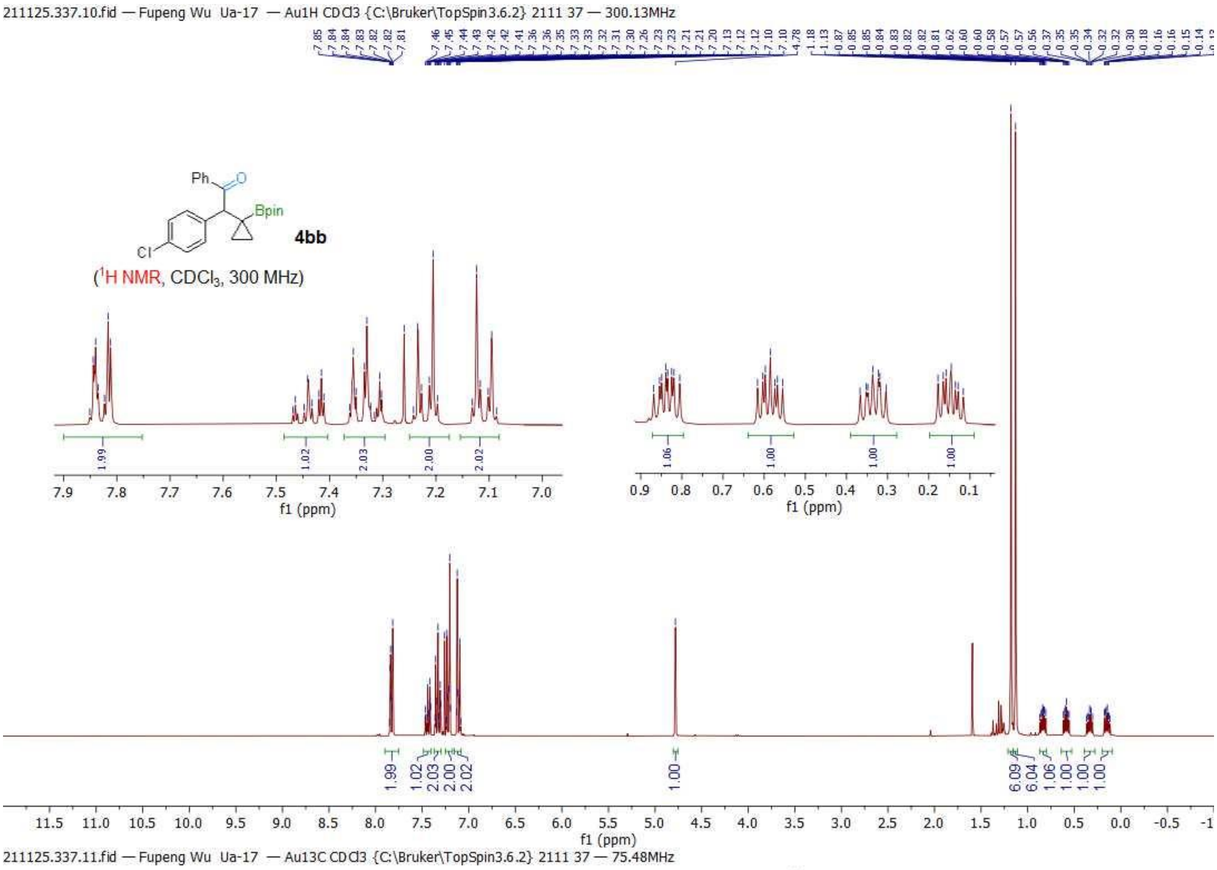
211125.337.10.fid — Fupeng Wu Ua-17 — Au1H CDCl3 {C:\Bruker\TopSpin3.6.2} 2111 37 — 300.13MHz
Ph
O
Bpin
Cl
4bb
(1H NMR, CDCl3, 300 MHz)
f1 (ppm)

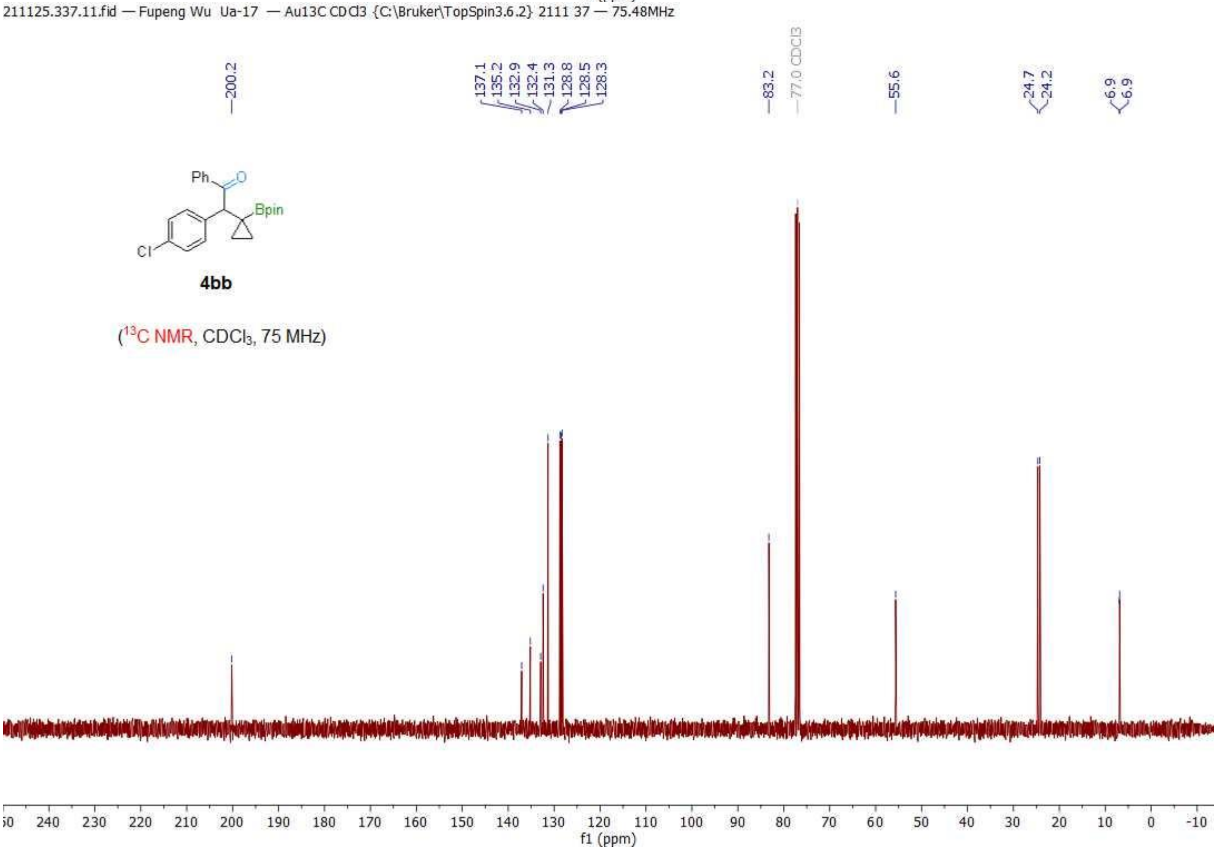
211125.337.11.fid — Fupeng Wu Ua-17 — Au13C CDCl3 {C:\Bruker\TopSpin3.6.2} 2111 37 — 75.48MHz
200.2
137.1
135.2
132.9
132.4
131.3
128.8
128.5
128.3
83.2
77.0 CDCl3
55.6
24.7
24.2
6.9
6.9
Ph
O
Bpin
Cl
4bb
(13C NMR, CDCl3, 75 MHz)
f1 (ppm)

211125.337.12.fid — Fupeng Wu Ua-17 — Au11B CDCl3 {C:\Bruker\TopSpin3.6.2} 2111 37 — 96.29MHz

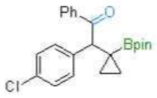

4bb

(^{11}B NMR, $CDCl_3$, 96 MHz)

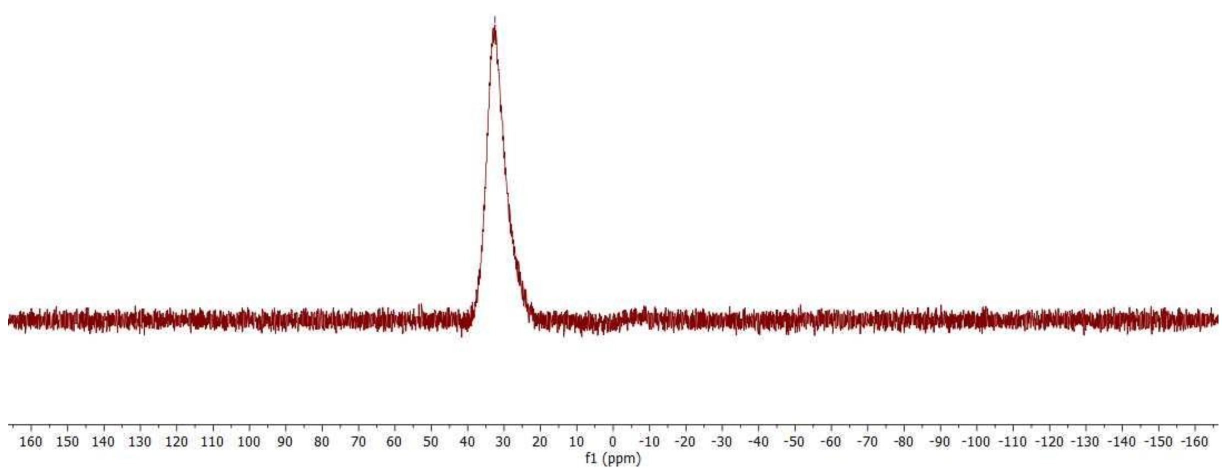

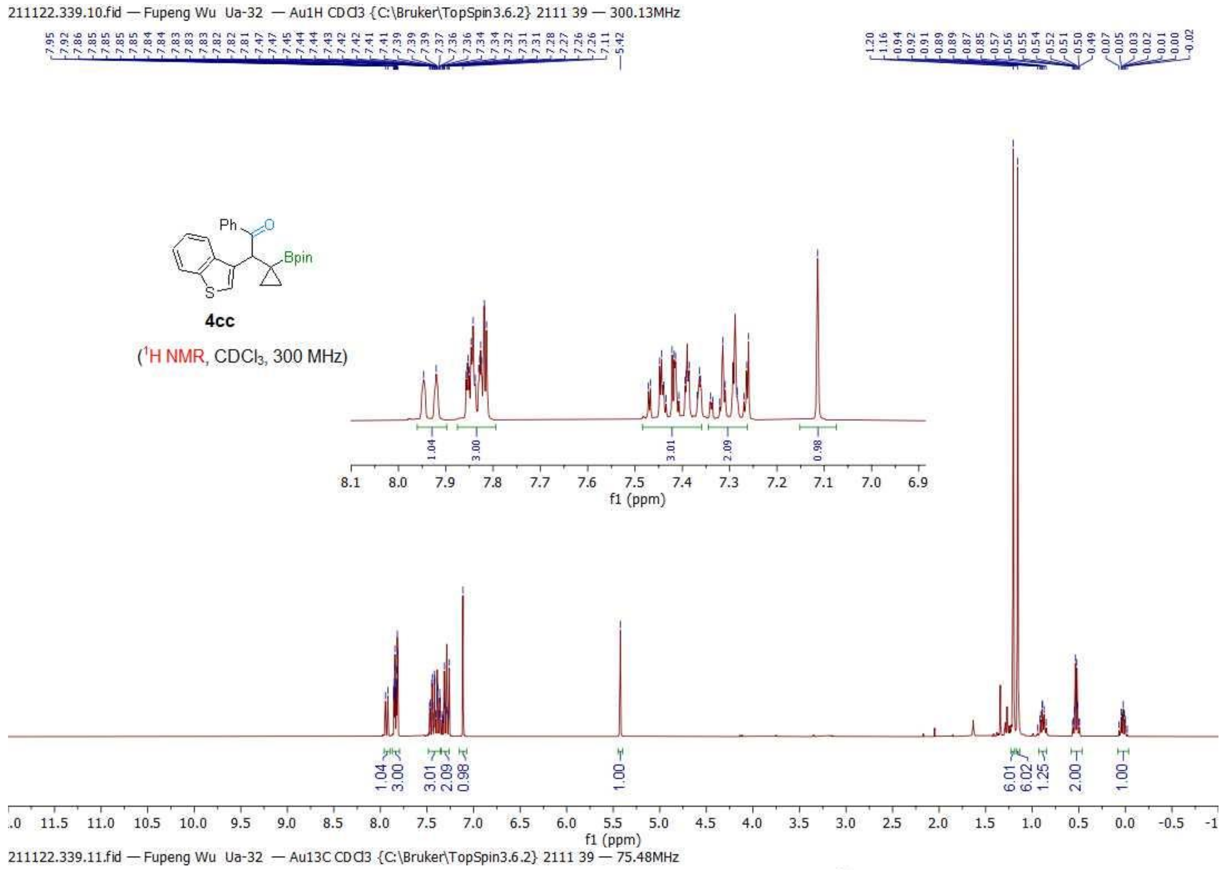
211122.339.10.fid — Fupeng Wu Ua-32 — Au1H CDCl3 {C:\Bruker\TopSpin3.6.2} 2111 39 — 300.13MHz
7.95 7.92 7.86 7.85 7.85 7.85 7.84 7.84 7.83 7.83 7.83 7.82 7.81 7.47 7.45 7.44 7.44 7.43 7.42 7.42 7.41 7.41 7.39 7.39 7.39 7.37 7.36 7.36 7.34 7.34 7.32 7.31 7.31 7.28 7.27 7.26 7.26 7.11 5.42
1.20 1.16 0.94 0.92 0.91 0.89 0.89 0.87 0.85 0.57 0.56 0.55 0.54 0.52 0.51 0.50 0.49 0.07 0.05 0.03 0.02 0.01 0.00 -0.02
Ph
O
Bpin
S
4cc
(1H NMR, CDCl3, 300 MHz)
1.04 3.00 3.01 2.09 0.98
1.04 3.00 3.01 2.09 0.98 1.00 6.01 6.02 1.25 2.00 1.00
f1 (ppm)

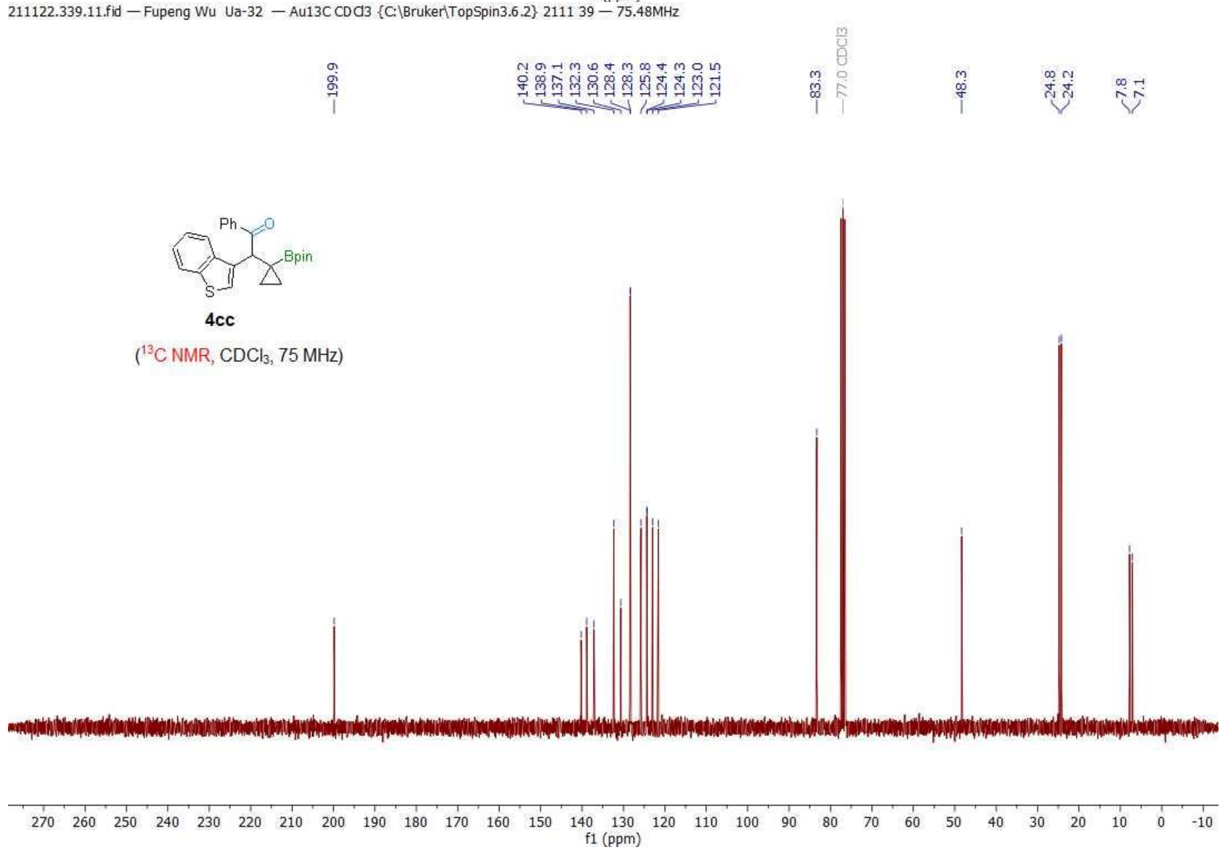
211122.339.11.fid — Fupeng Wu Ua-32 — Au13C CDCl3 {C:\Bruker\TopSpin3.6.2} 2111 39 — 75.48MHz
199.9
140.2 138.9 137.1 132.3 130.6 128.4 128.3 125.8 124.4 124.3 123.0 121.5
83.3
77.0 CDCl3
48.3
24.8 24.2
7.8 7.1
Ph
O
Bpin
S
4cc
(13C NMR, CDCl3, 75 MHz)
f1 (ppm)

211122.339.12.fid — Fupeng Wu Ua-32 — Au11B CDCl3 {C:\Bruker\TopSpin3.6.2} 2111 39 — 96.29MHz

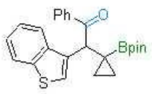

4cc

(^{11}B NMR, $CDCl_3$, 96 MHz)

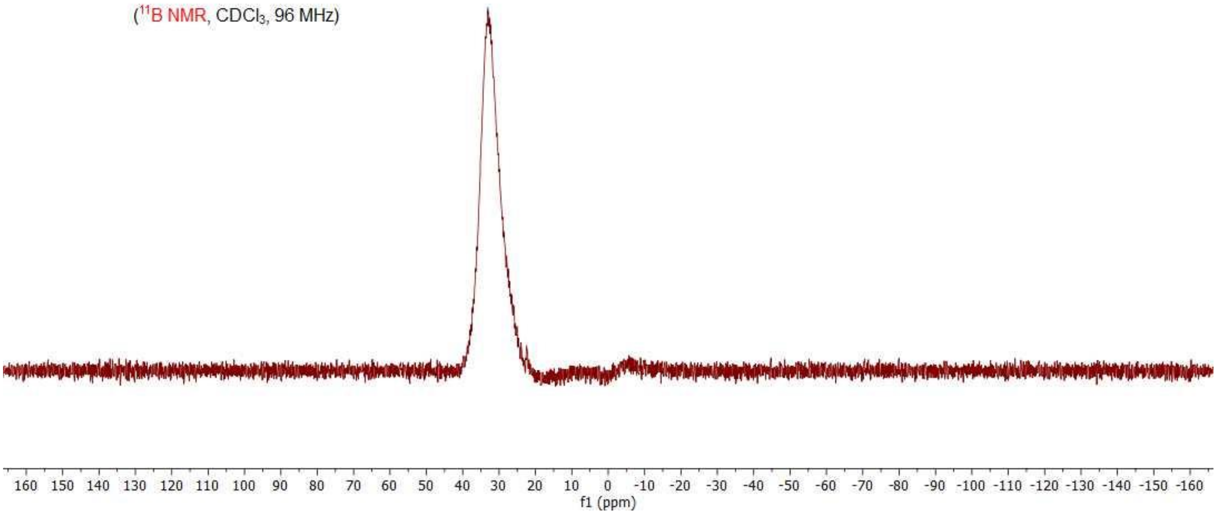

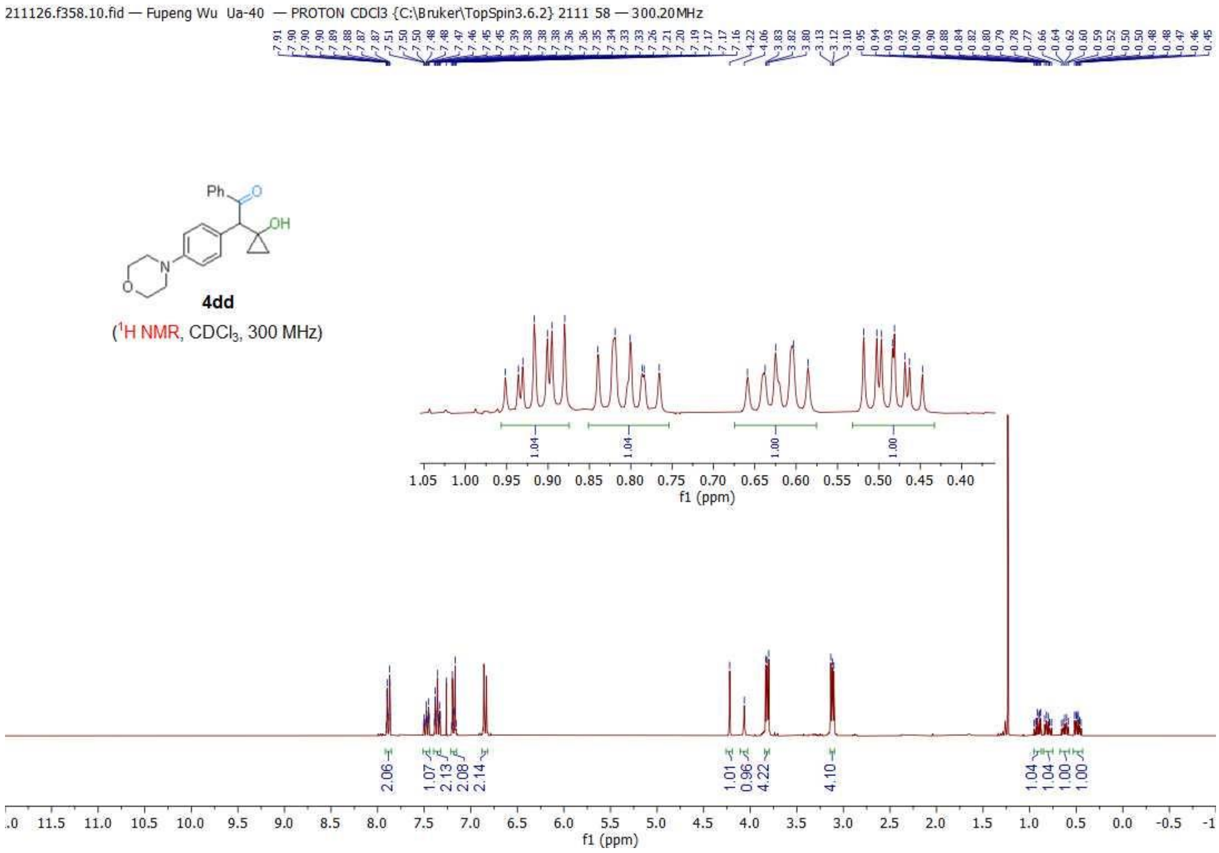
211126.f358.10.fid — Fupeng Wu Ua-40 — PROTON CDCl3 {C:\Bruker\TopSpin3.6.2} 2111 58 — 300.20MHz
Ph
O
OH
N
O
4dd
(¹H NMR, CDCl₃, 300 MHz)
f1 (ppm)

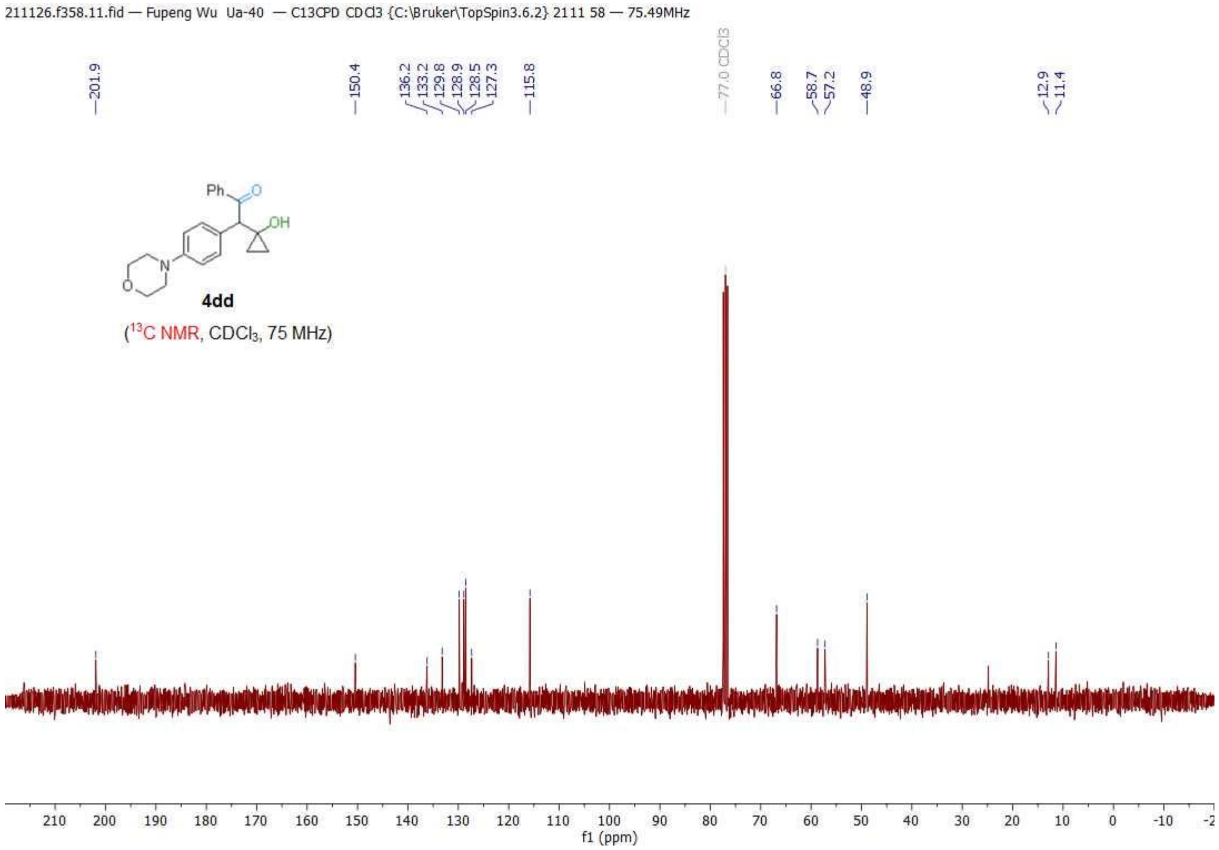
211126.f358.11.fid — Fupeng Wu Ua-40 — C13CPD CDCl3 {C:\Bruker\TopSpin3.6.2} 2111 58 — 75.49MHz
201.9
150.4
136.2
133.2
129.8
128.9
128.5
127.3
115.8
77.0 CDCl3
66.8
58.7
57.2
48.9
12.9
11.4
Ph
O
OH
N
O
4dd
(¹³C NMR, CDCl₃, 75 MHz)
f1 (ppm)

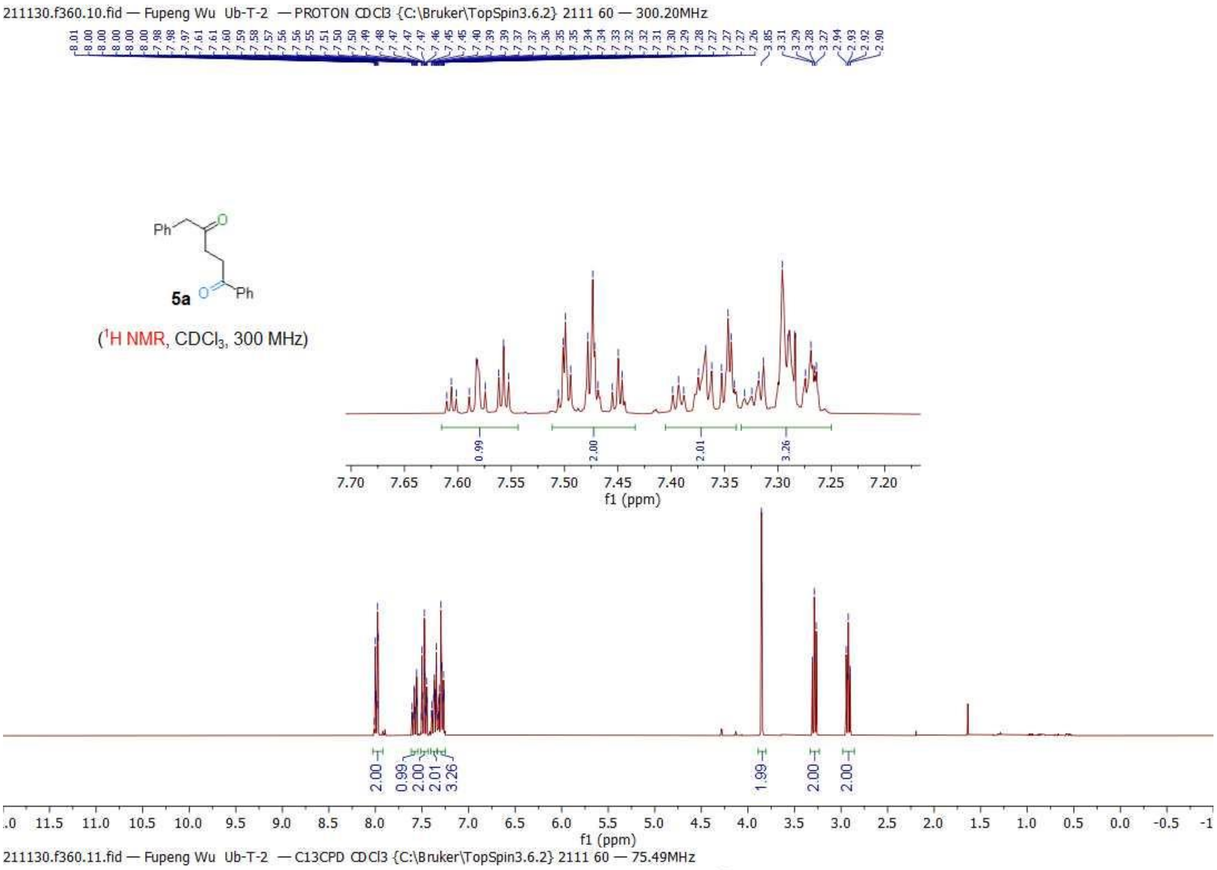
211130.f360.10.fid — Fupeng Wu Ub-T-2 — PROTON CDCl3 {C:\Bruker\TopSpin3.6.2} 2111 60 — 300.20MHz
5a
(1H NMR, CDCl3, 300 MHz)
f1 (ppm)

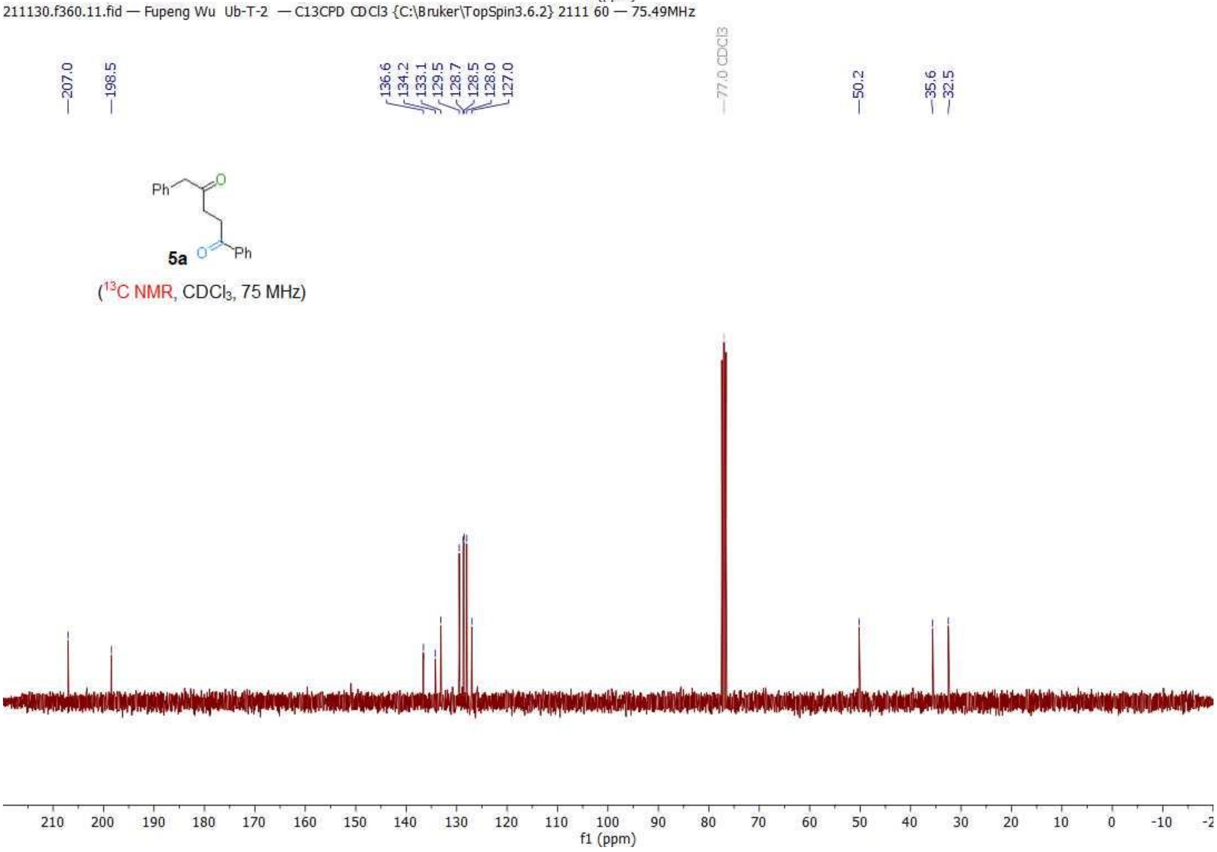
211130.f360.11.fid — Fupeng Wu Ub-T-2 — C13CPD CDCl3 {C:\Bruker\TopSpin3.6.2} 2111 60 — 75.49MHz
207.0
198.5
136.6
134.2
133.1
129.5
128.7
128.5
128.0
127.0
77.0 CDCl3
50.2
35.6
32.5
5a
(13C NMR, CDCl3, 75 MHz)
f1 (ppm)

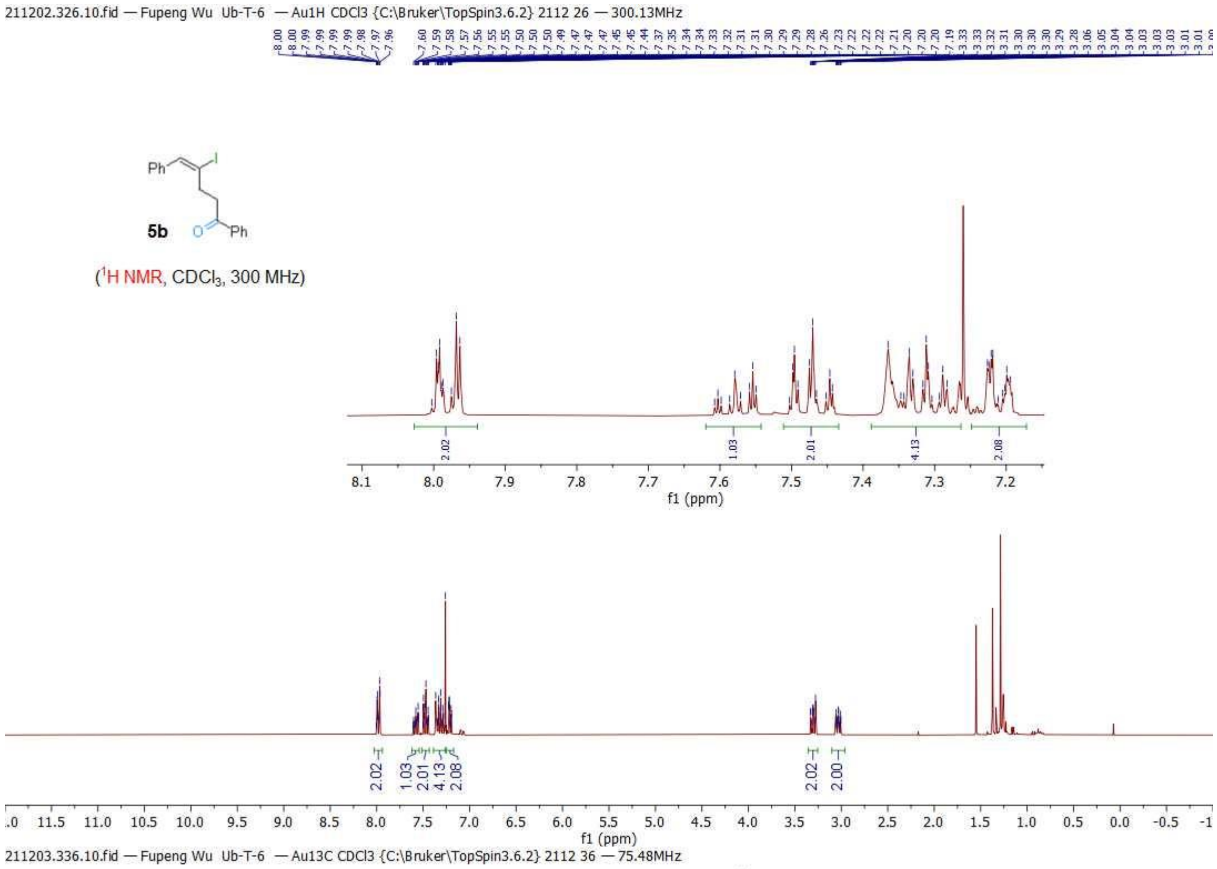
211202.326.10.fid — Fupeng Wu Ub-T-6 — Au1H CDCl3 {C:\Bruker\TopSpin3.6.2} 2112 26 — 300.13MHz
5b
(1H NMR, CDCl3, 300 MHz)
f1 (ppm)

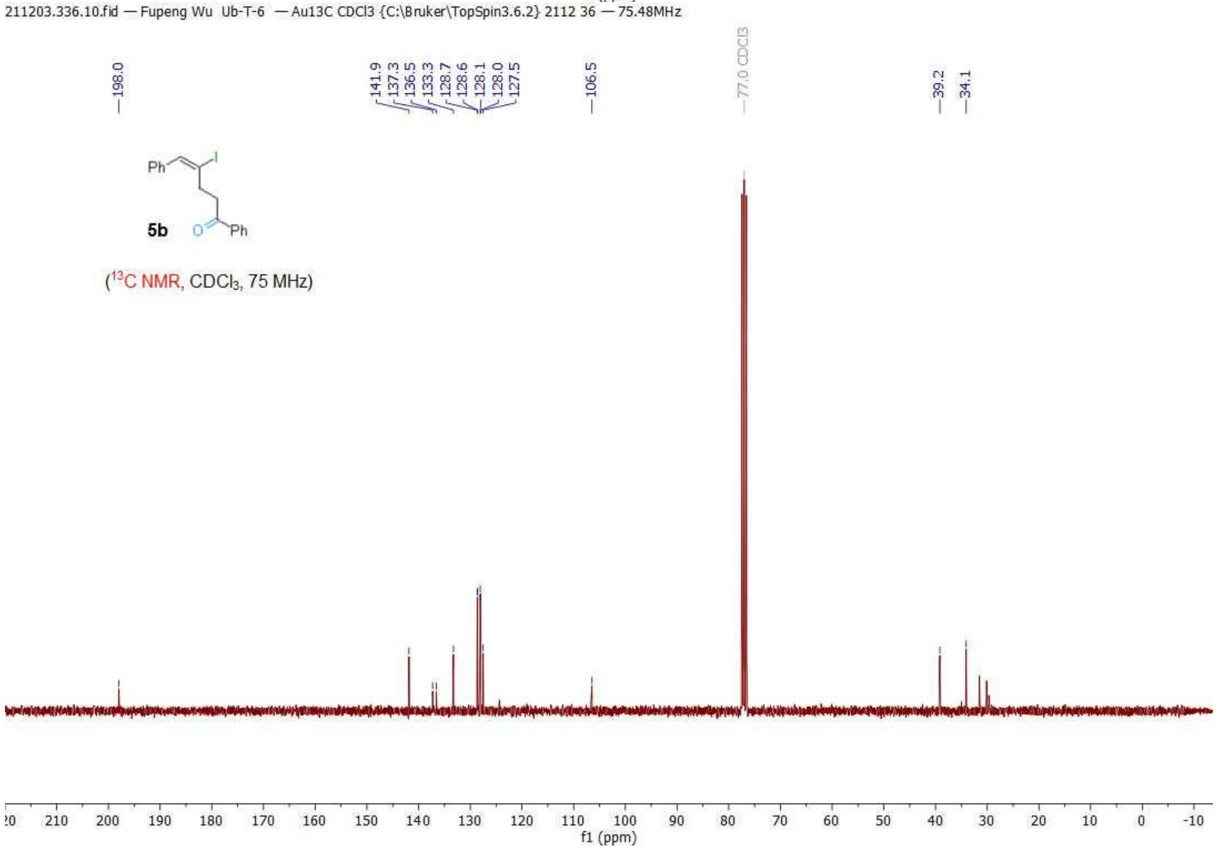
211203.336.10.fid — Fupeng Wu Ub-T-6 — Au13C CDCl3 {C:\Bruker\TopSpin3.6.2} 2112 36 — 75.48MHz
198.0
141.9
137.3
136.5
133.3
128.7
128.6
128.1
128.0
127.5
106.5
77.0 CDCl3
39.2
34.1
5b
(13C NMR, CDCl3, 75 MHz)
f1 (ppm)

211201.f366.10.fid — Fupeng Wu Ub-T-3 — PROTON CDCl3 {C:\Bruker\TopSpin3.6.2} 2112 6 — 300.20MHz

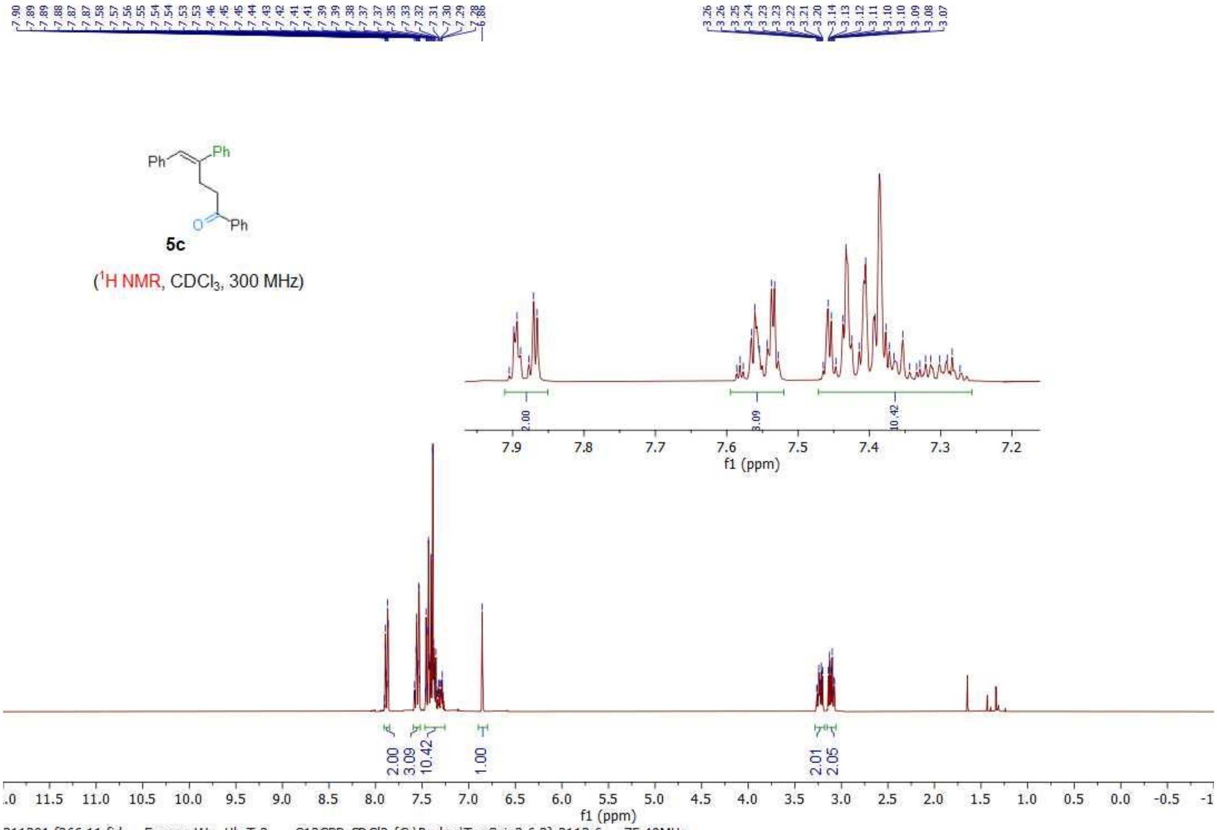

211201.f366.11.fid — Fupeng Wu Ub-T-3 — C13CPD CDCl3 {C:\Bruker\TopSpin3.6.2} 2112 6 — 75.49MHz

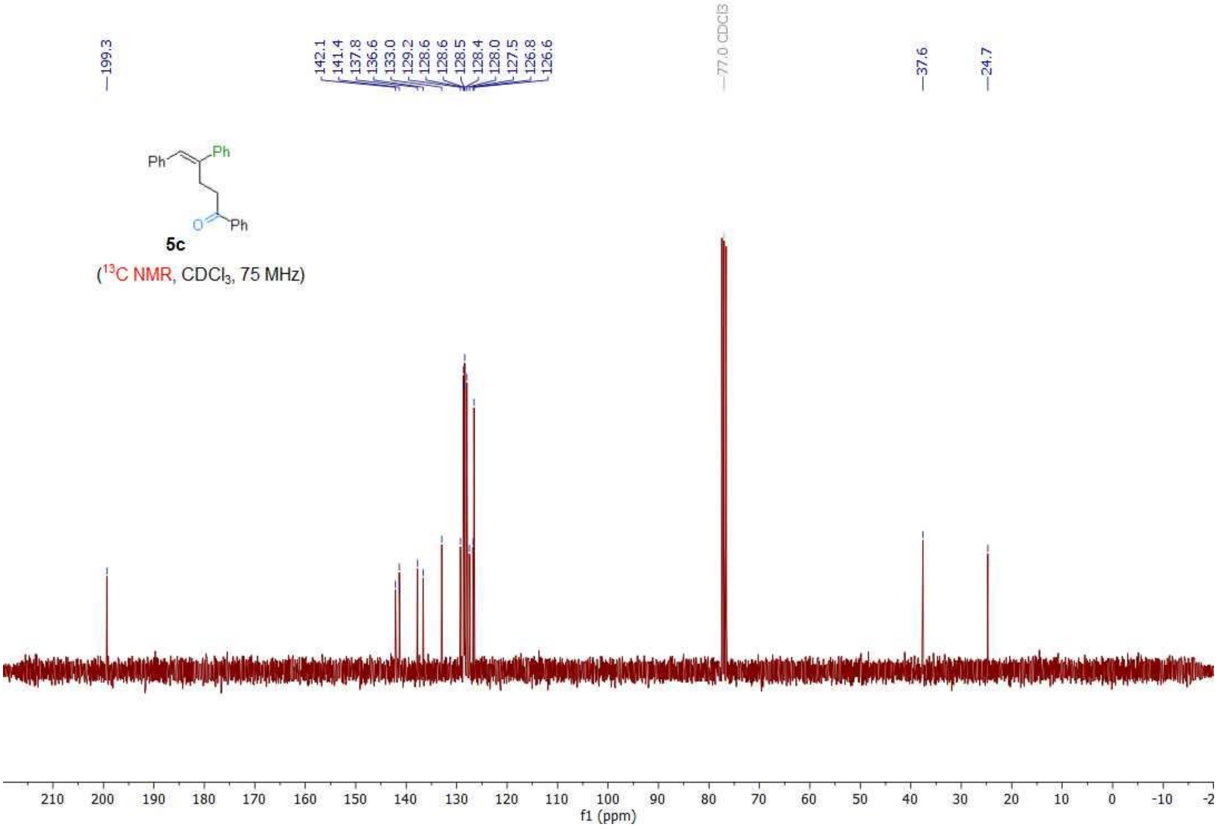

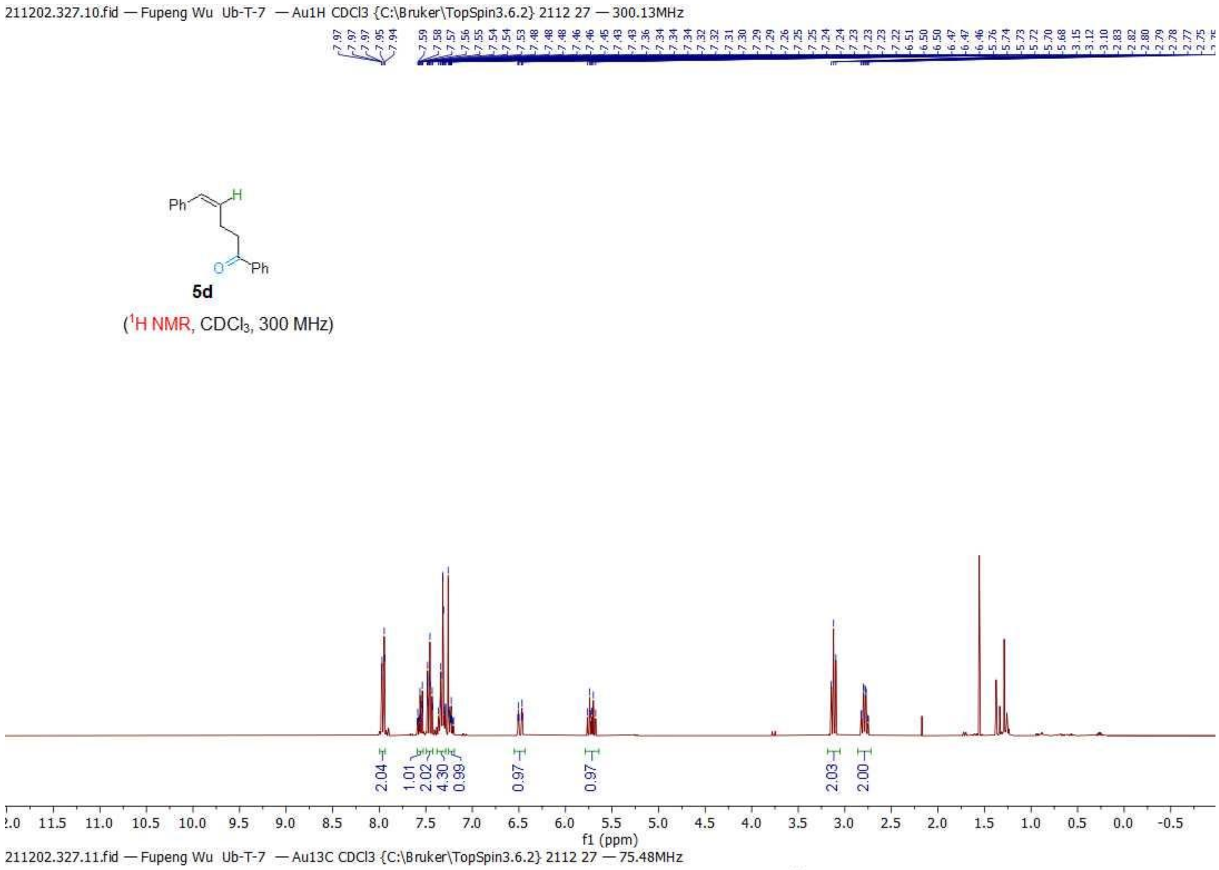
211202.327.10.fid — Fupeng Wu Ub-T-7 — Au1H CDCl3 {C:\Bruker\TopSpin3.6.2} 2112 27 — 300.13MHz
Ph
H
O
Ph
5d
(^{1}H NMR, CDCl$_3$, 300 MHz)
2.04
1.01
2.02
4.30
0.99
0.97
0.97
2.03
2.00
f1 (ppm)

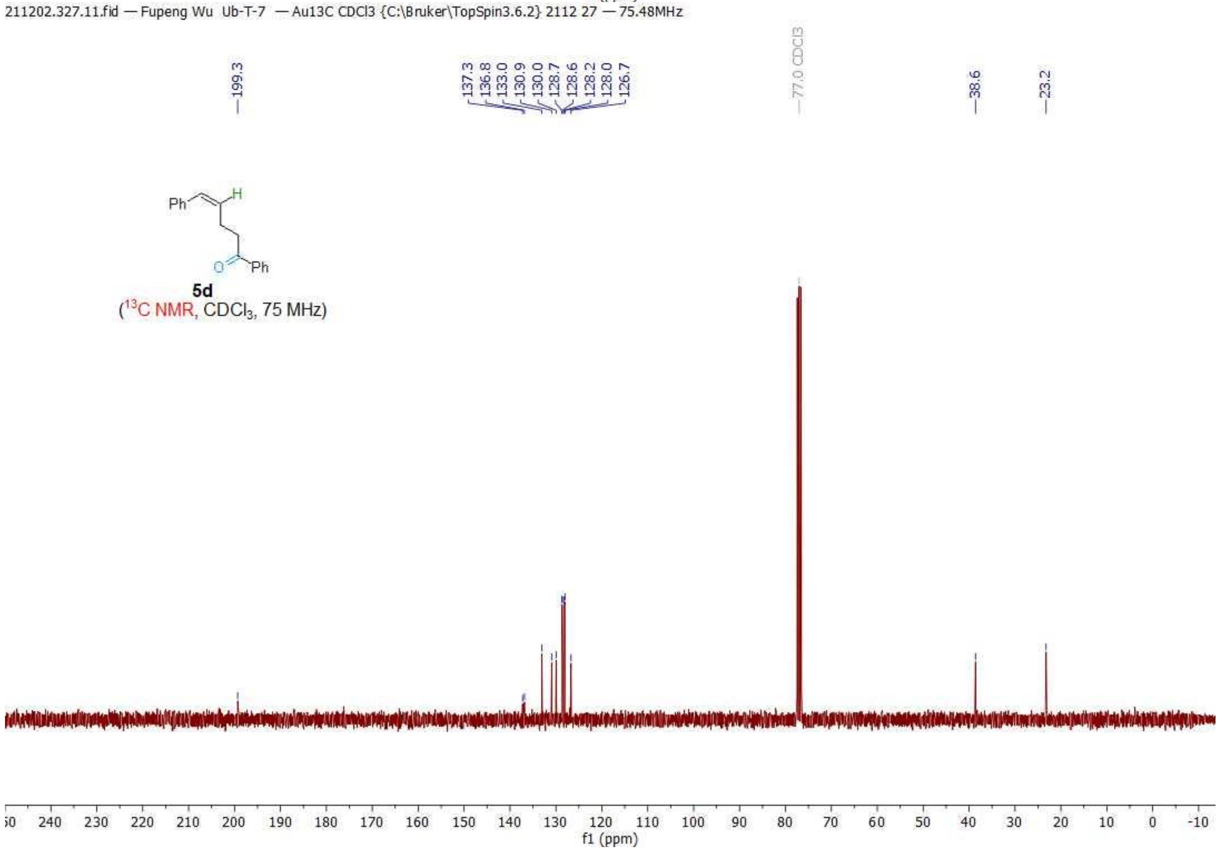
211202.327.11.fid — Fupeng Wu Ub-T-7 — Au13C CDCl3 {C:\Bruker\TopSpin3.6.2} 2112 27 — 75.48MHz
199.3
137.3
136.8
133.0
130.9
130.0
128.7
128.6
128.2
128.0
126.7
77.0 CDCl3
38.6
23.2
Ph
H
O
Ph
5d
(^{13}C NMR, CDCl$_3$, 75 MHz)
f1 (ppm)

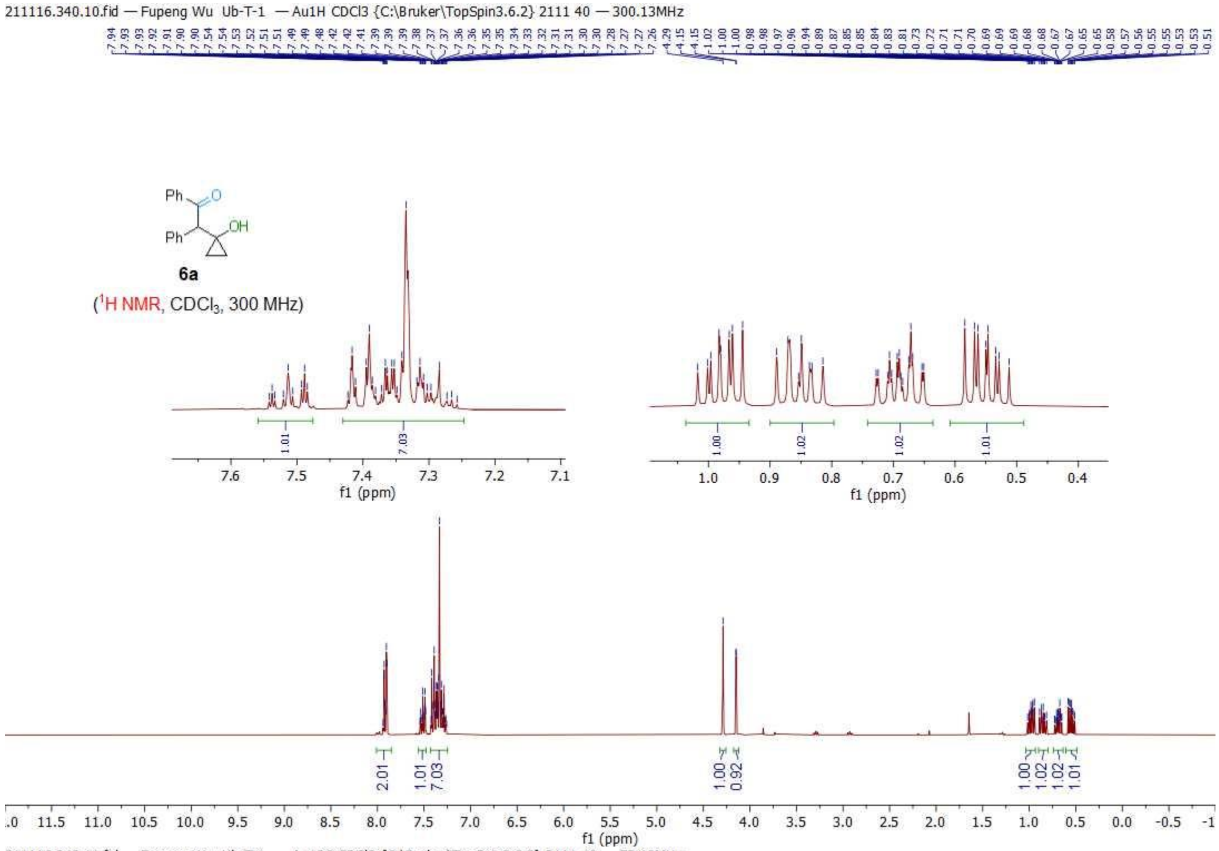
211116.340.10.fid — Fupeng Wu Ub-T-1 — Au1H CDCl3 {C:\Bruker\TopSpin3.6.2} 2111 40 — 300.13MHz
Ph
O
Ph
OH
6a
(1H NMR, CDCl3, 300 MHz)
f1 (ppm)

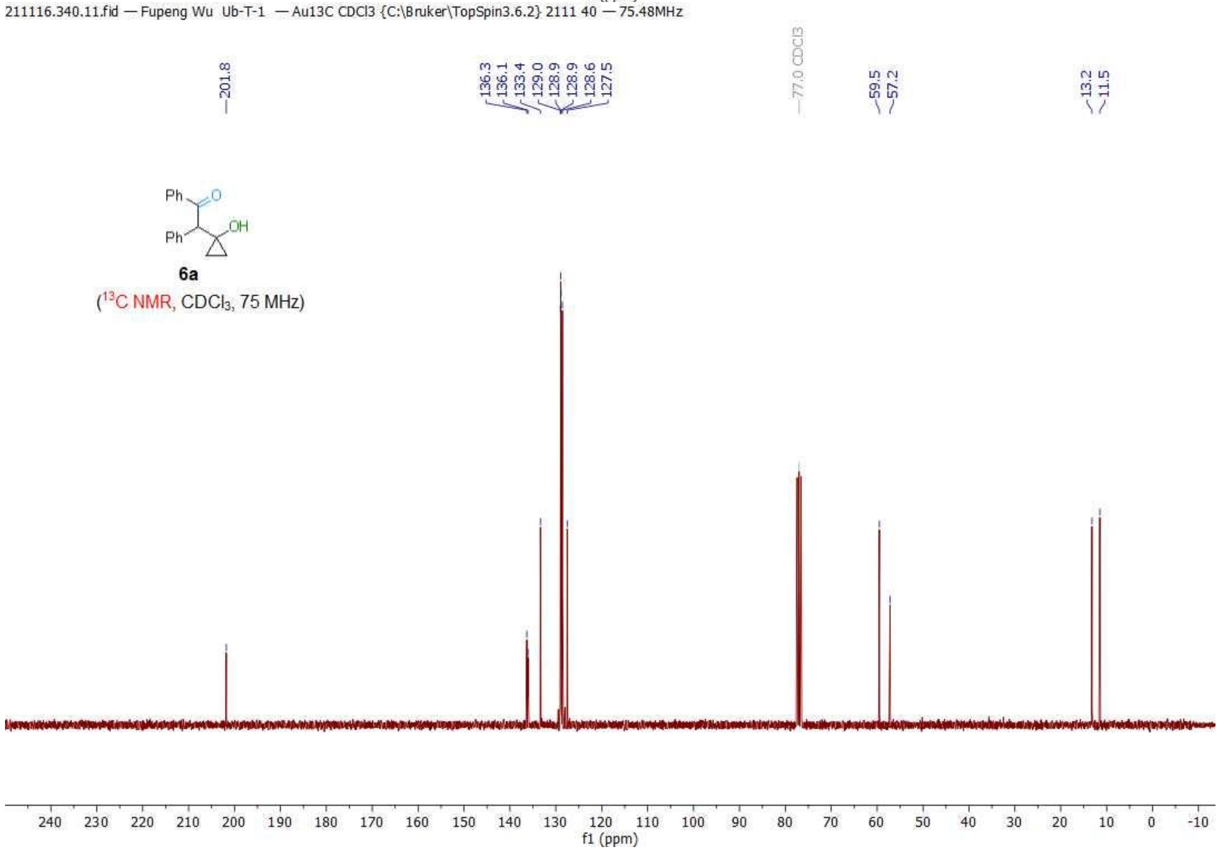
211116.340.11.fid — Fupeng Wu Ub-T-1 — Au13C CDCl3 {C:\Bruker\TopSpin3.6.2} 2111 40 — 75.48MHz
201.8
136.3
136.1
133.4
129.0
128.9
128.9
128.6
127.5
77.0 CDCl3
59.5
57.2
13.2
11.5
Ph
O
Ph
OH
6a
(13C NMR, CDCl3, 75 MHz)
f1 (ppm)

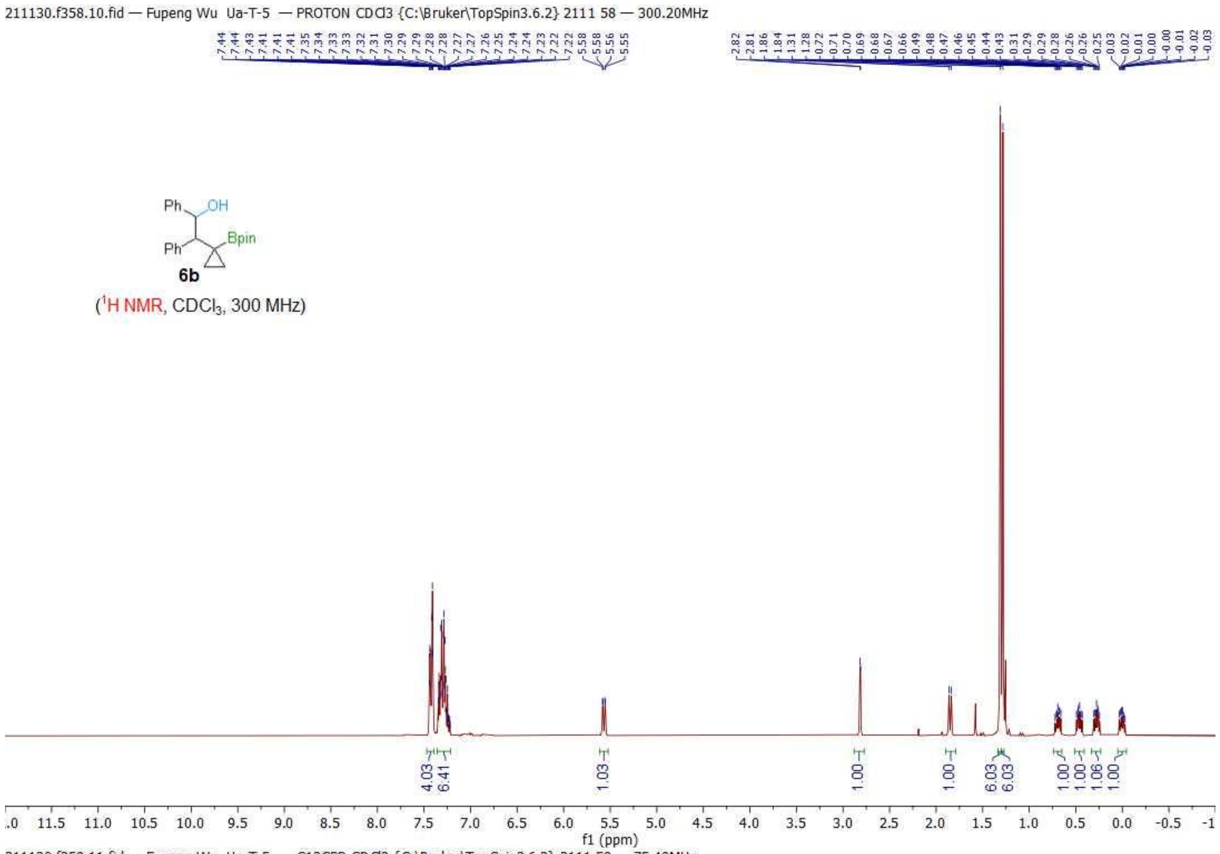
211130.f358.10.fid — Fupeng Wu Ua-T-5 — PROTON CDCl3 {C:\Bruker\TopSpin3.6.2} 2111 58 — 300.20MHz
Ph
OH
Ph
Bpin
6b
(1H NMR, CDCl3, 300 MHz)
4.03
6.41
1.03
1.00
1.00
6.03
6.03
1.00
1.00
1.06
1.00
f1 (ppm)

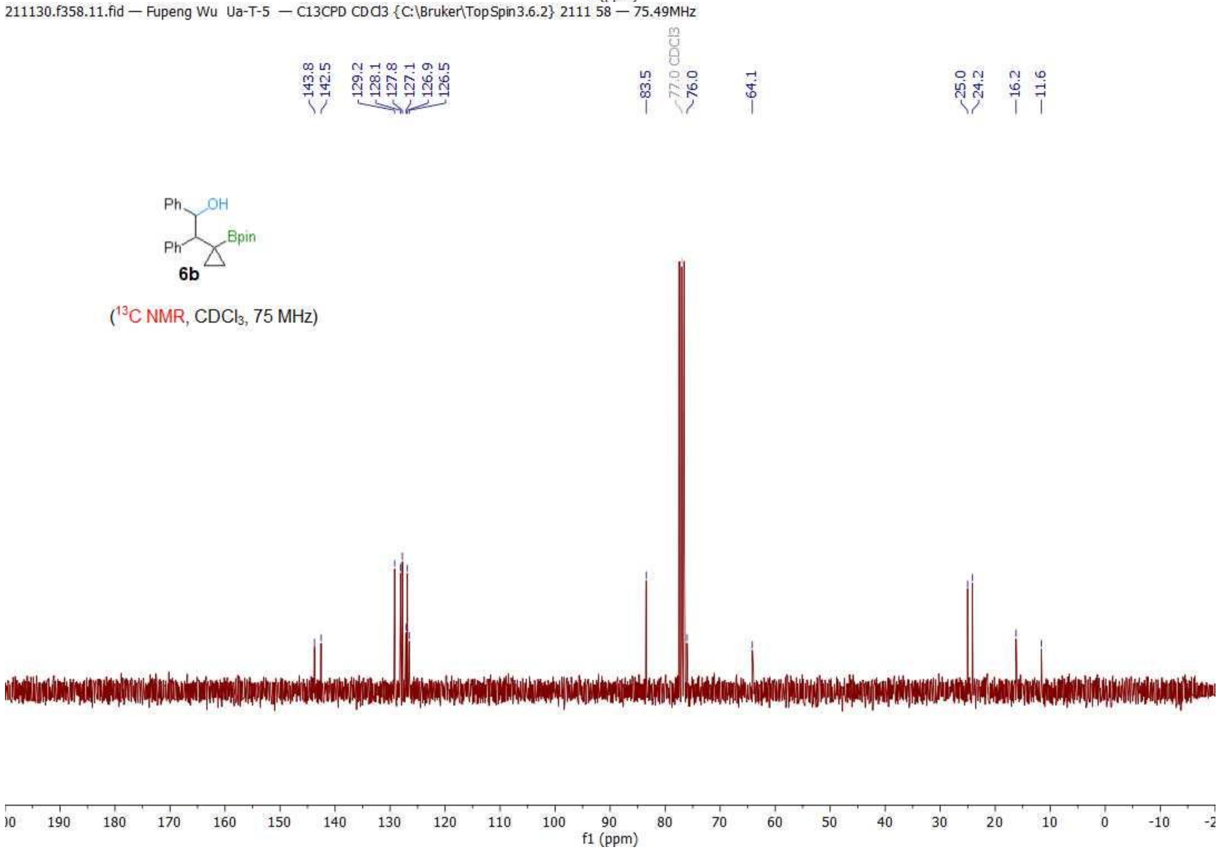
211130.f358.11.fid — Fupeng Wu Ua-T-5 — C13CPD CDCl3 {C:\Bruker\TopSpin3.6.2} 2111 58 — 75.49MHz
143.8
142.5
129.2
128.1
127.8
127.1
126.9
126.5
83.5
77.0 CDCl3
76.0
64.1
25.0
24.2
16.2
11.6
Ph
OH
Ph
Bpin
6b
(13C NMR, CDCl3, 75 MHz)
f1 (ppm)

211130.f358.12.fid — Fupeng Wu Ua-T-5 — 11B CDCl3 {C:\Bruker\TopSpin3.6.2} 2111 58 — 96.32MHz

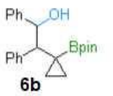

6b

(^{11}B NMR, CDCl$_3$, 96 MHz)

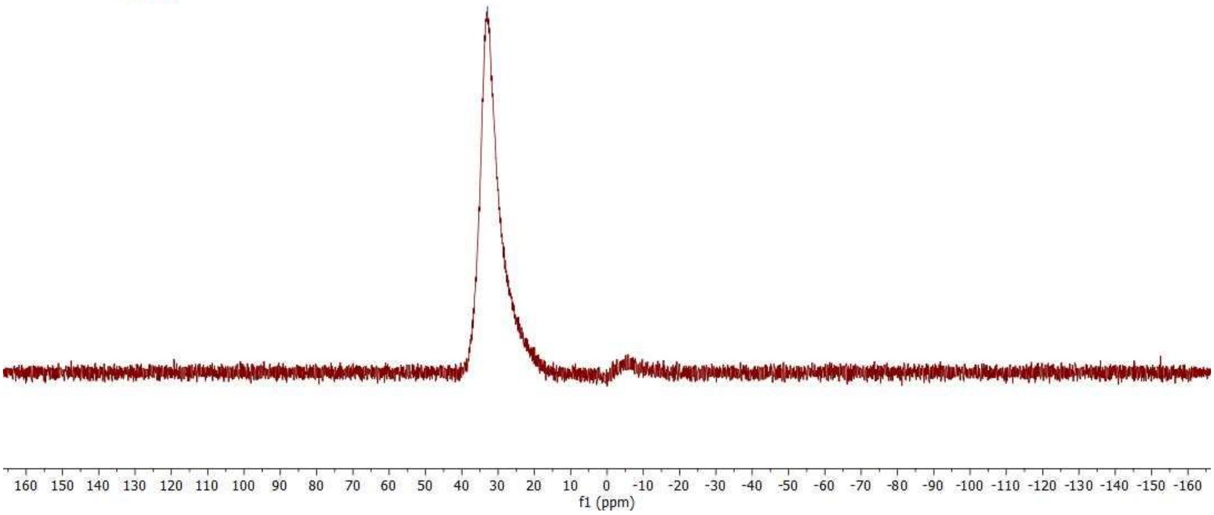

6c

(^{1}H NMR, CDCl$_3$, 300 MHz)

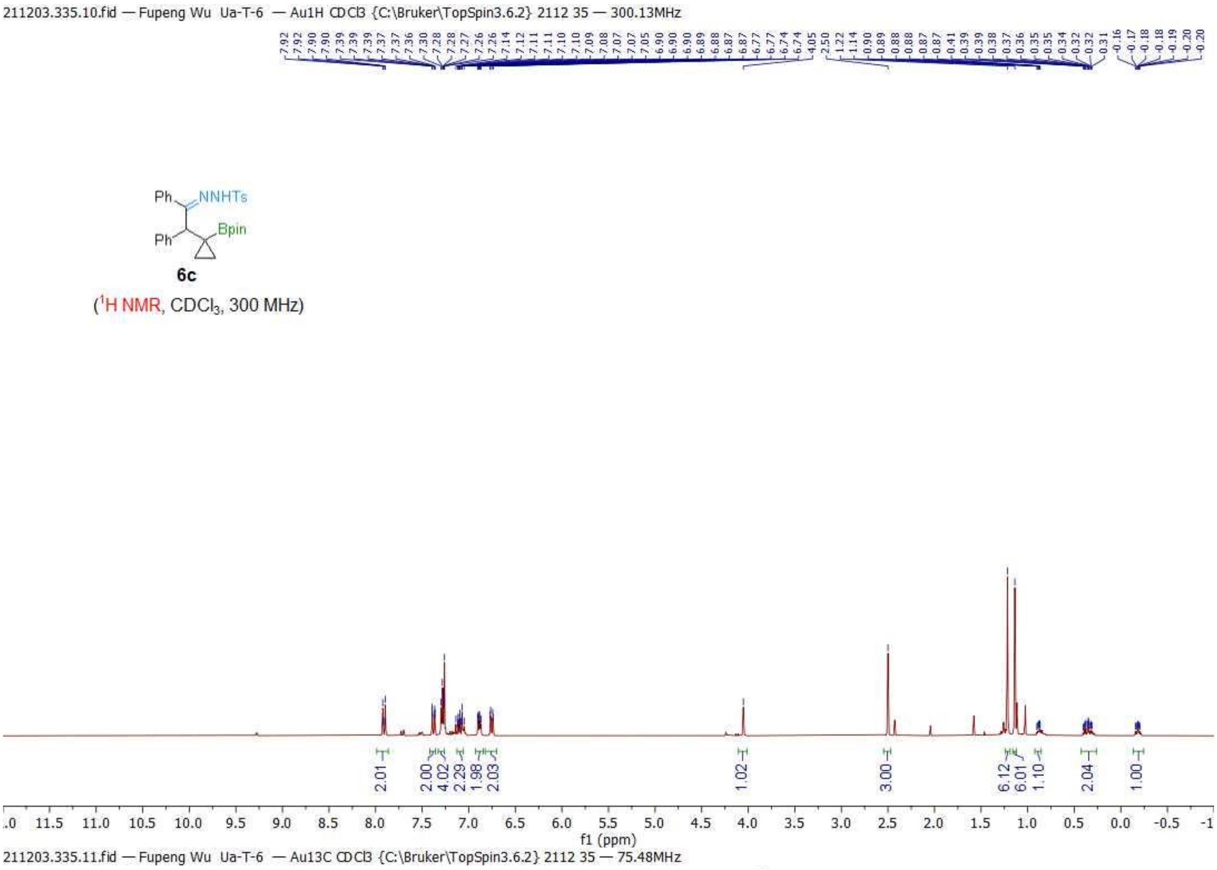

(^{13}C NMR, CDCl$_3$, 75 MHz)

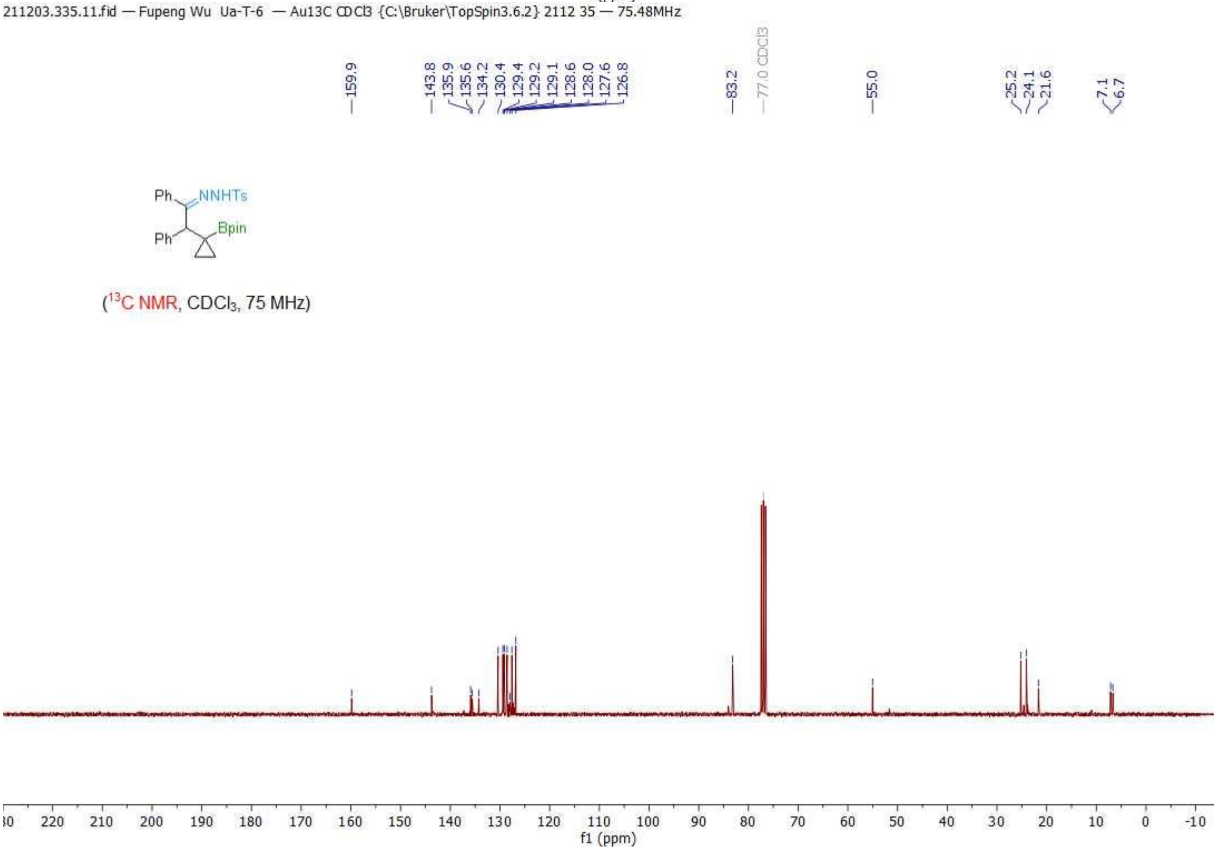

211210.f334.10.fid — Fupeng Wu Ua-T-6 — 11B(H-entk) CDCl3 {C:\Bruker\TopSpin3.6.2} 2112 34 — 96.32MHz

—33.5

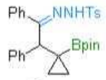

6c

(11B NMR, $CDCl_3$, 96 MHz)

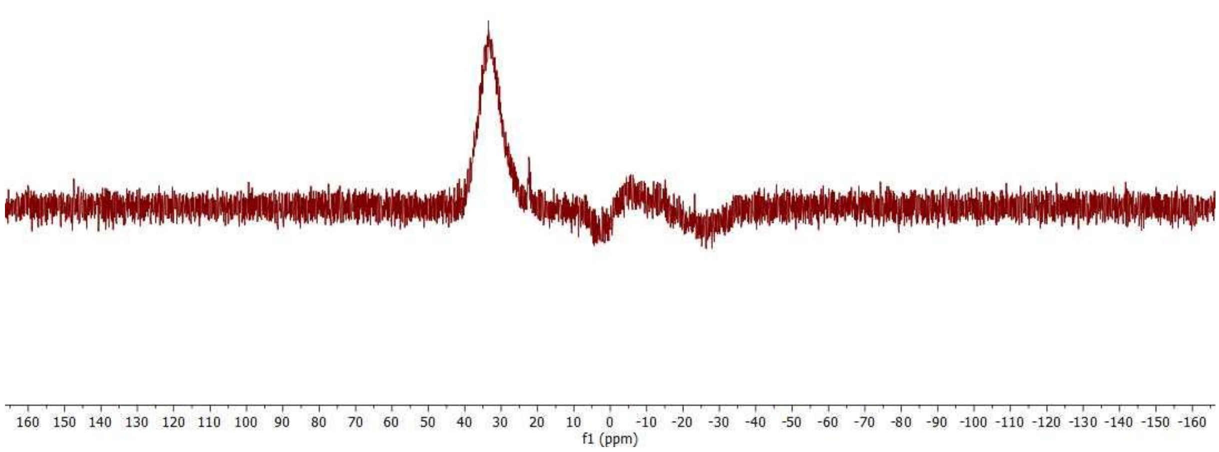

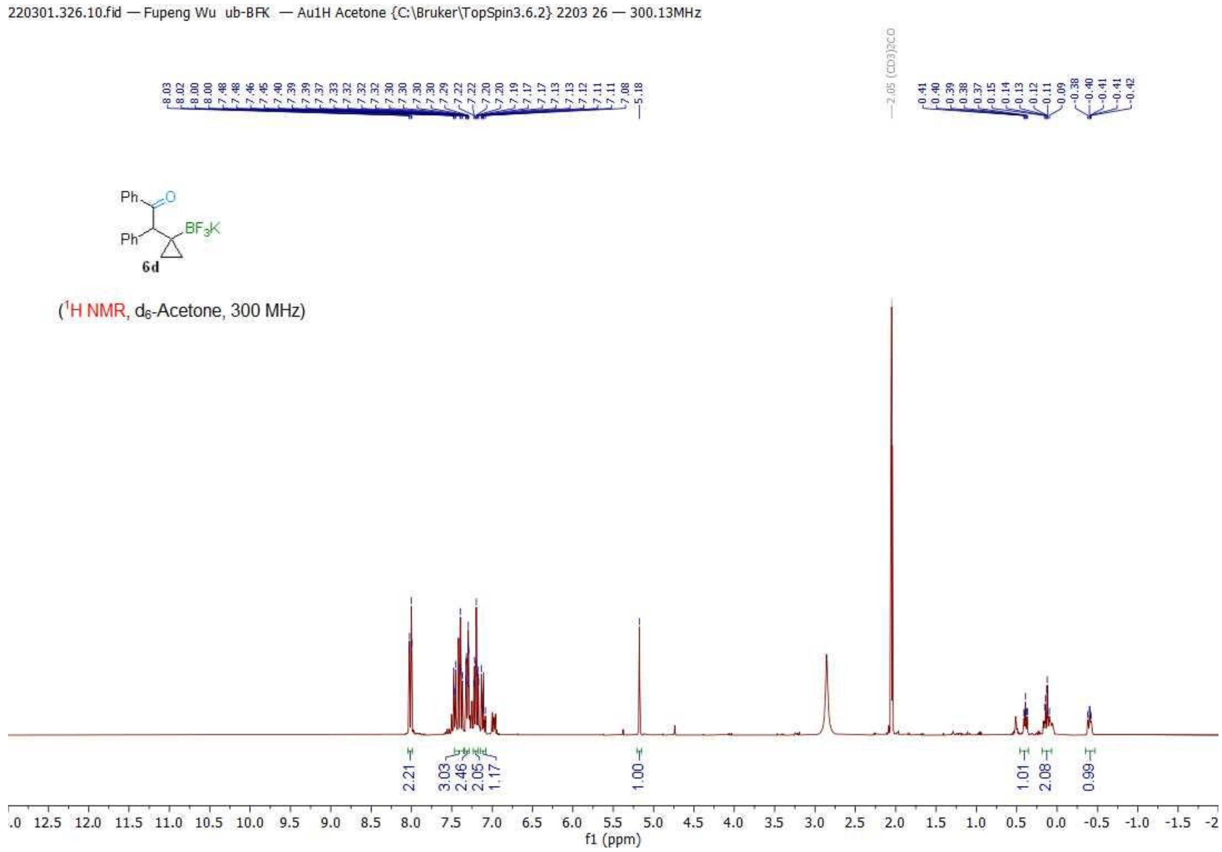
220301.326.10.fid — Fupeng Wu ub-BFK — Au1H Acetone {C:\Bruker\TopSpin3.6.2} 2203 26 — 300.13MHz
8.03 8.02 8.00 7.48 7.46 7.45 7.40 7.39 7.39 7.37 7.33 7.32 7.32 7.32 7.30 7.30 7.30 7.30 7.29 7.22 7.22 7.20 7.19 7.17 7.17 7.13 7.12 7.11 7.11 7.08 5.18
2.05 (CD3)2CO
0.41 0.40 0.39 0.38 0.37 0.15 0.14 0.13 0.12 0.11 0.09 -0.38 -0.40 -0.41 -0.42
Ph
O
Ph
BF3K
6d
(1H NMR, d6-Acetone, 300 MHz)
2.21
3.03
2.46
2.05
1.17
1.00
1.01
2.08
0.99
f1 (ppm)
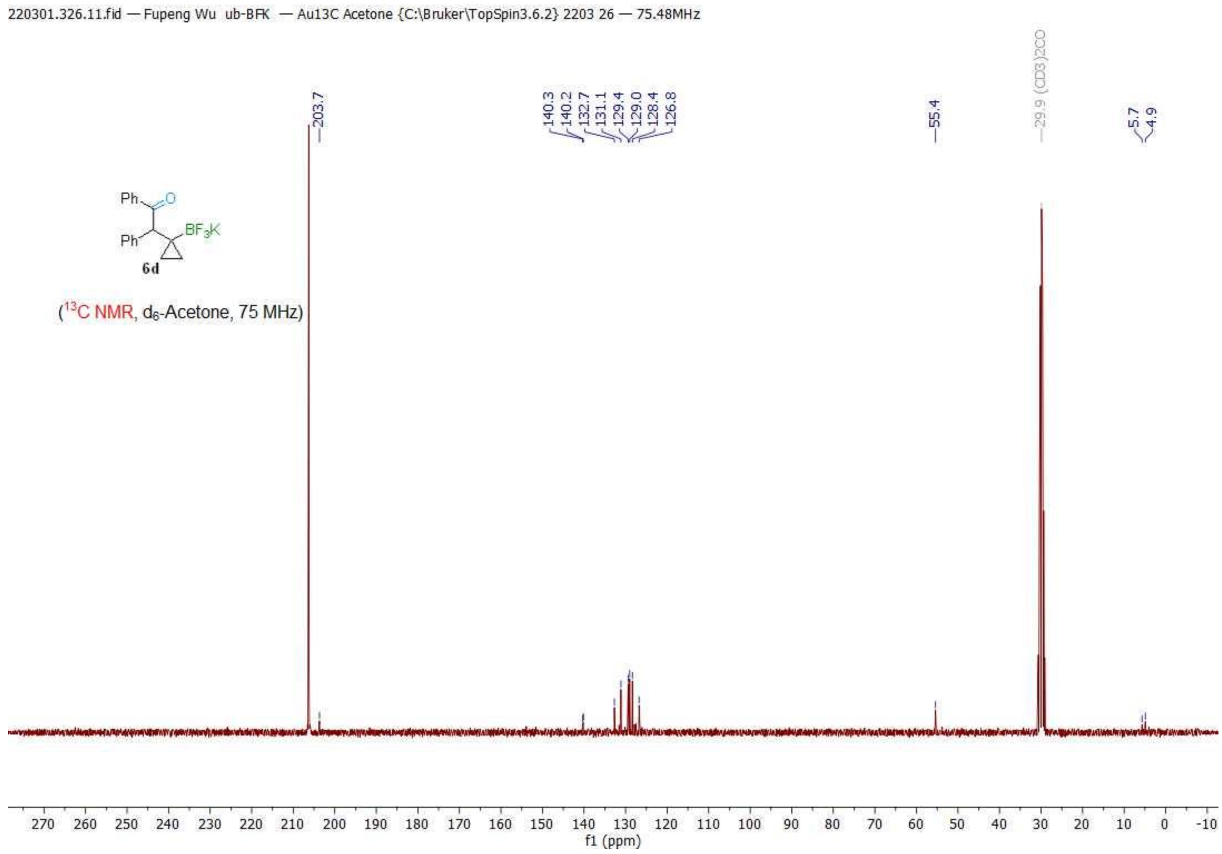
220301.326.11.fid — Fupeng Wu ub-BFK — Au13C Acetone {C:\Bruker\TopSpin3.6.2} 2203 26 — 75.48MHz
203.7
140.3 140.2 132.7 131.1 129.4 129.0 128.4 126.8
55.4
29.9 (CD3)2CO
5.7 4.9
Ph
O
Ph
BF3K
6d
(13C NMR, d6-Acetone, 75 MHz)
f1 (ppm)

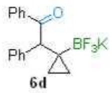

6d

(^{11}B NMR, d_6-Acetone, 96 MHz)

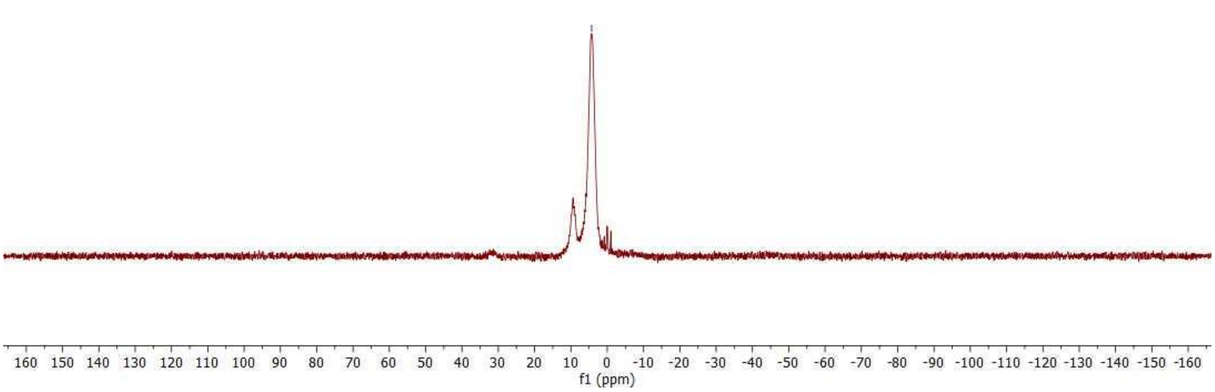

8.2 NMR spectra of the BCPs

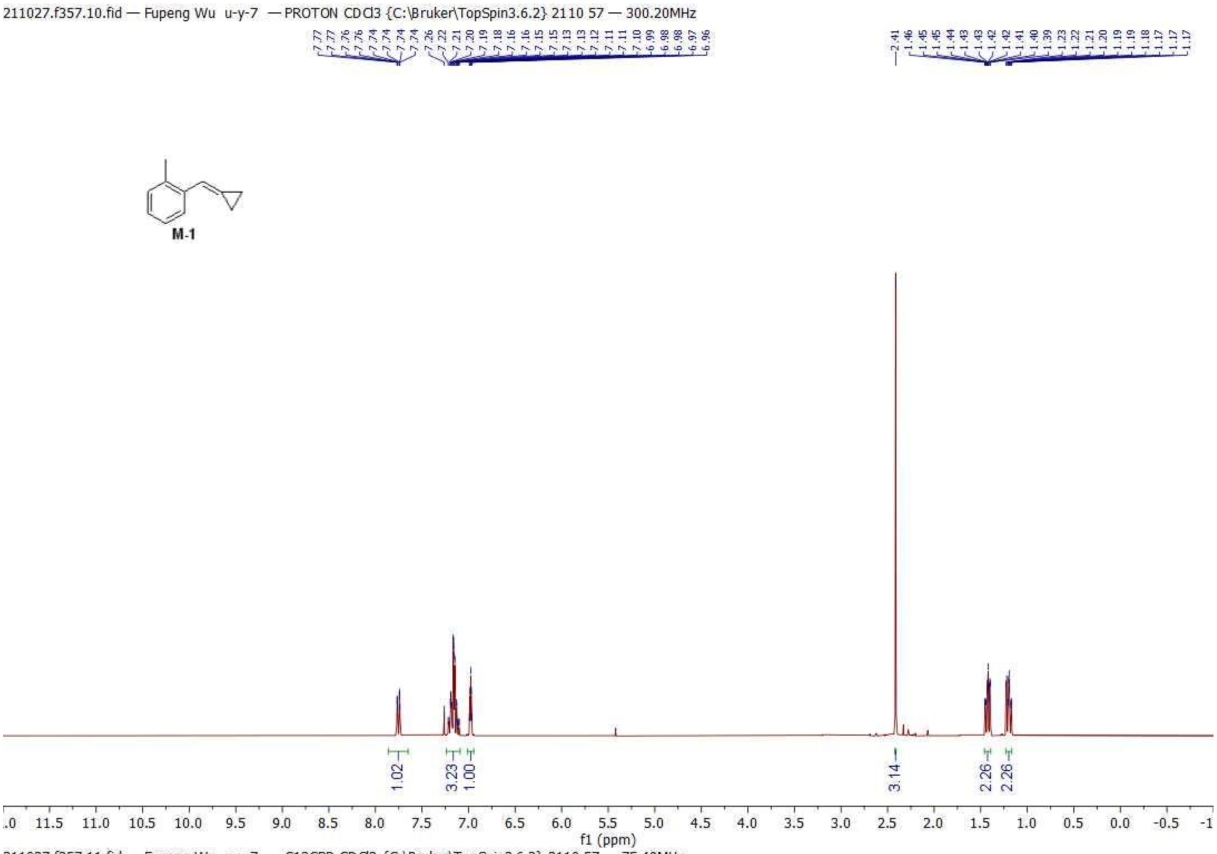

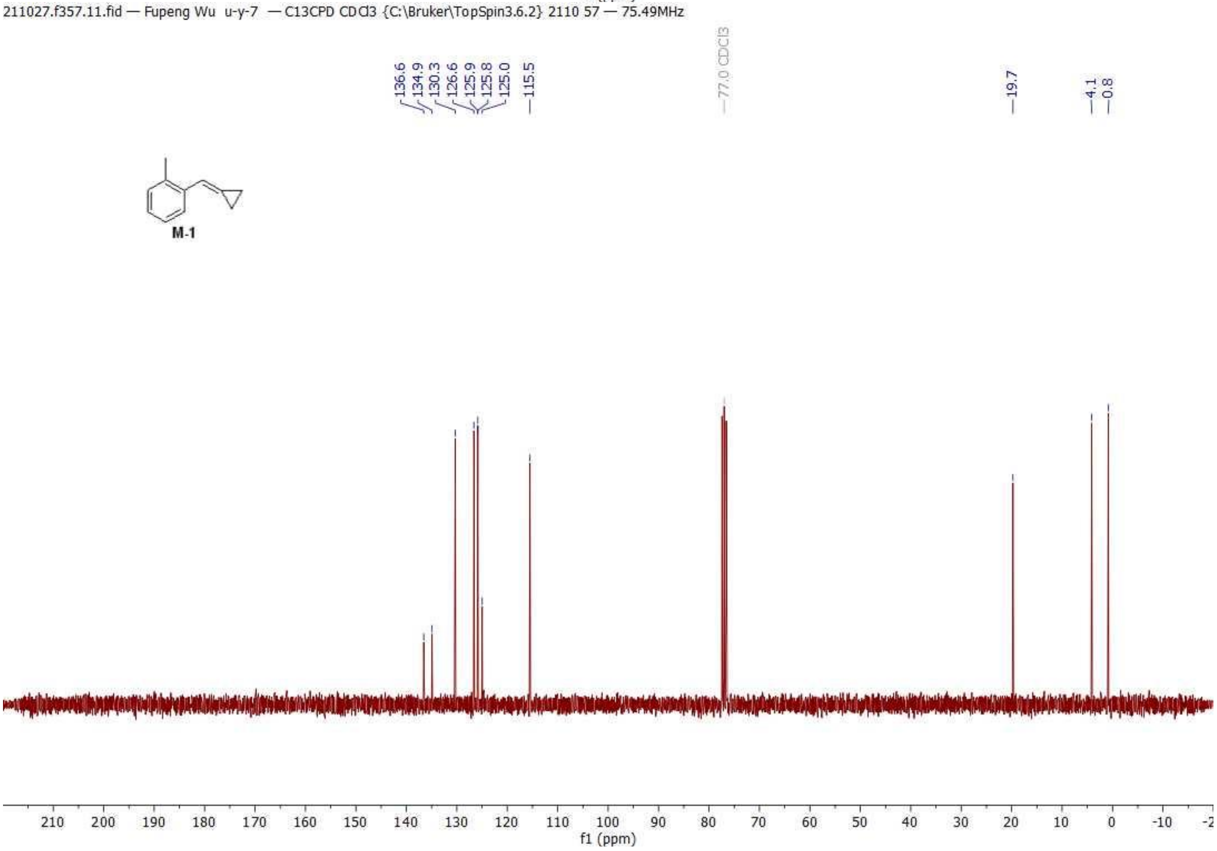

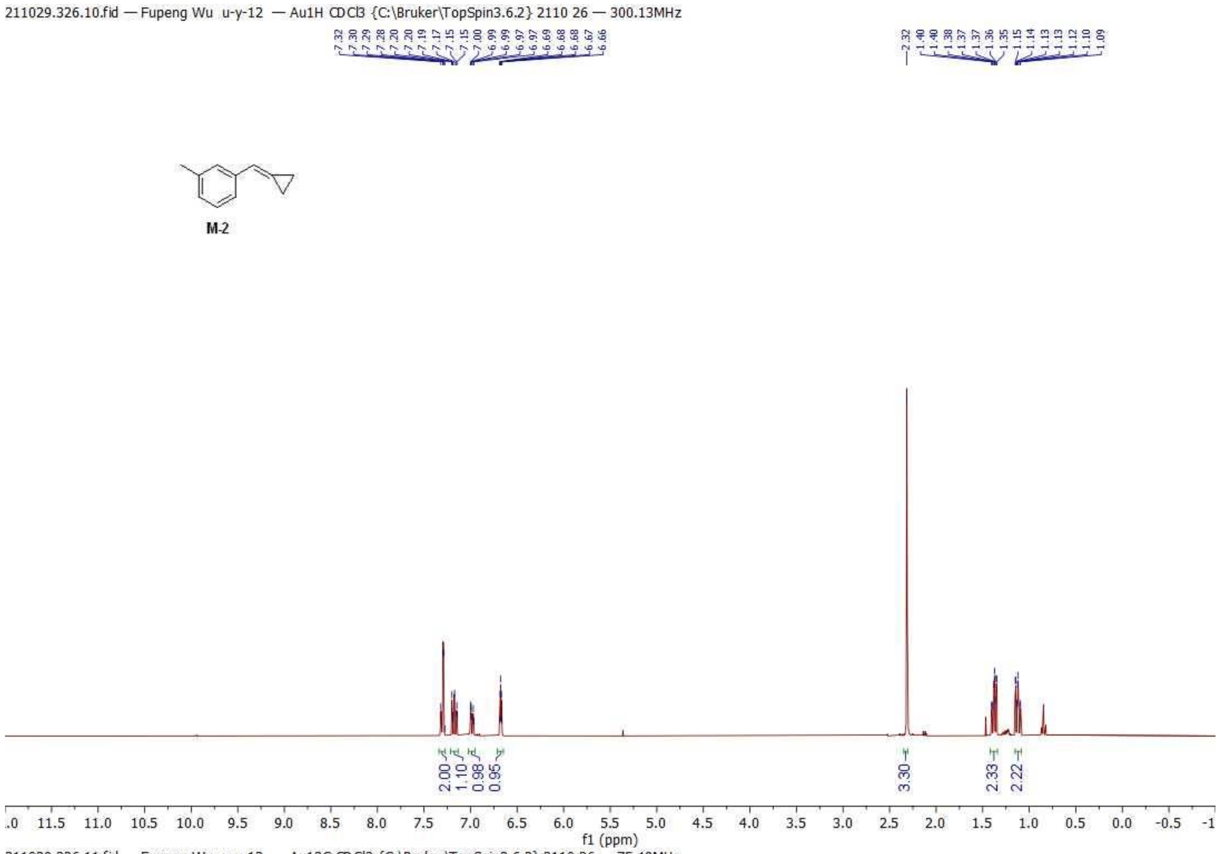
211029.326.10.fid — Fupeng Wu u-y-12 — Au1H CDCl3 {C:\Bruker\TopSpin3.6.2} 2110 26 — 300.13MHz
7.32 7.30 7.29 7.28 7.20 7.20 7.19 7.17 7.15 7.15 7.00 6.99 6.99 6.97 6.97 6.69 6.68 6.68 6.67 6.66
2.32 1.40 1.40 1.38 1.37 1.37 1.36 1.35 1.15 1.14 1.13 1.13 1.12 1.10 1.09
M-2
2.00 1.10 0.98 0.95
3.30
2.33 2.22
f1 (ppm)

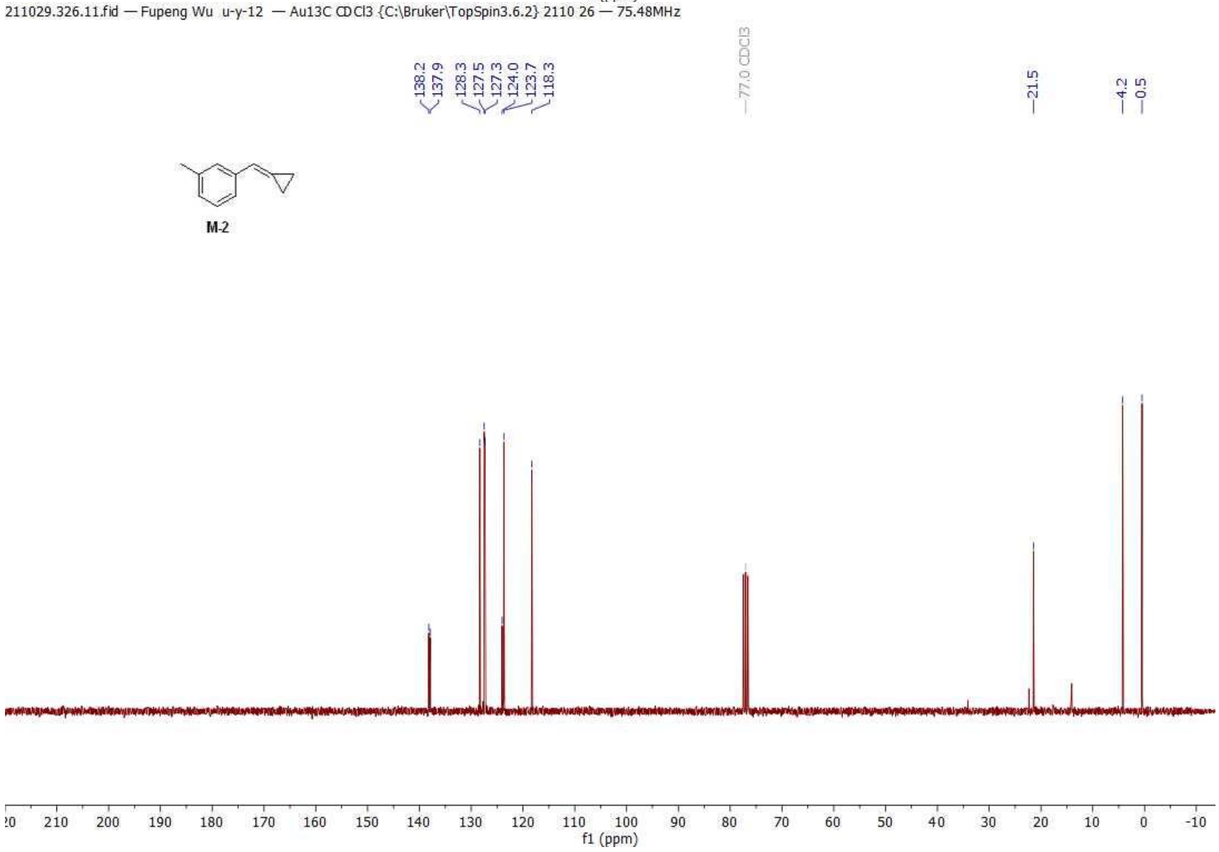
211029.326.11.fid — Fupeng Wu u-y-12 — Au13C CDCl3 {C:\Bruker\TopSpin3.6.2} 2110 26 — 75.48MHz
138.2 137.9 128.3 127.5 127.3 124.0 123.7 118.3
77.0 CDCl3
21.5
4.2 0.5
M-2
f1 (ppm)

211027.f360.10.fid — Fupeng Wu u-y-6 — PROTON CDCl3 {C:\Bruker\TopSpin3.6.2} 2110 60 — 300.20MHz

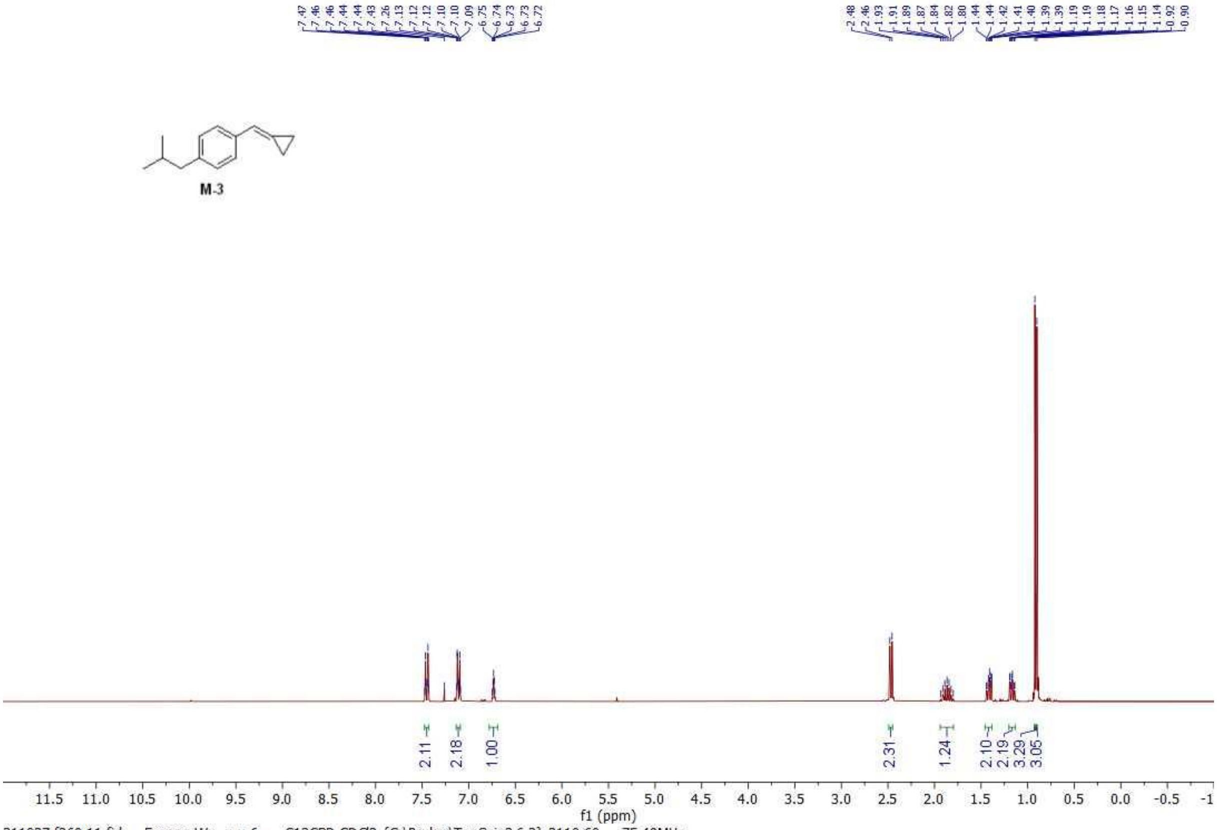

211027.f360.11.fid — Fupeng Wu u-y-6 — C13CPD CDCl3 {C:\Bruker\TopSpin3.6.2} 2110 60 — 75.49MHz

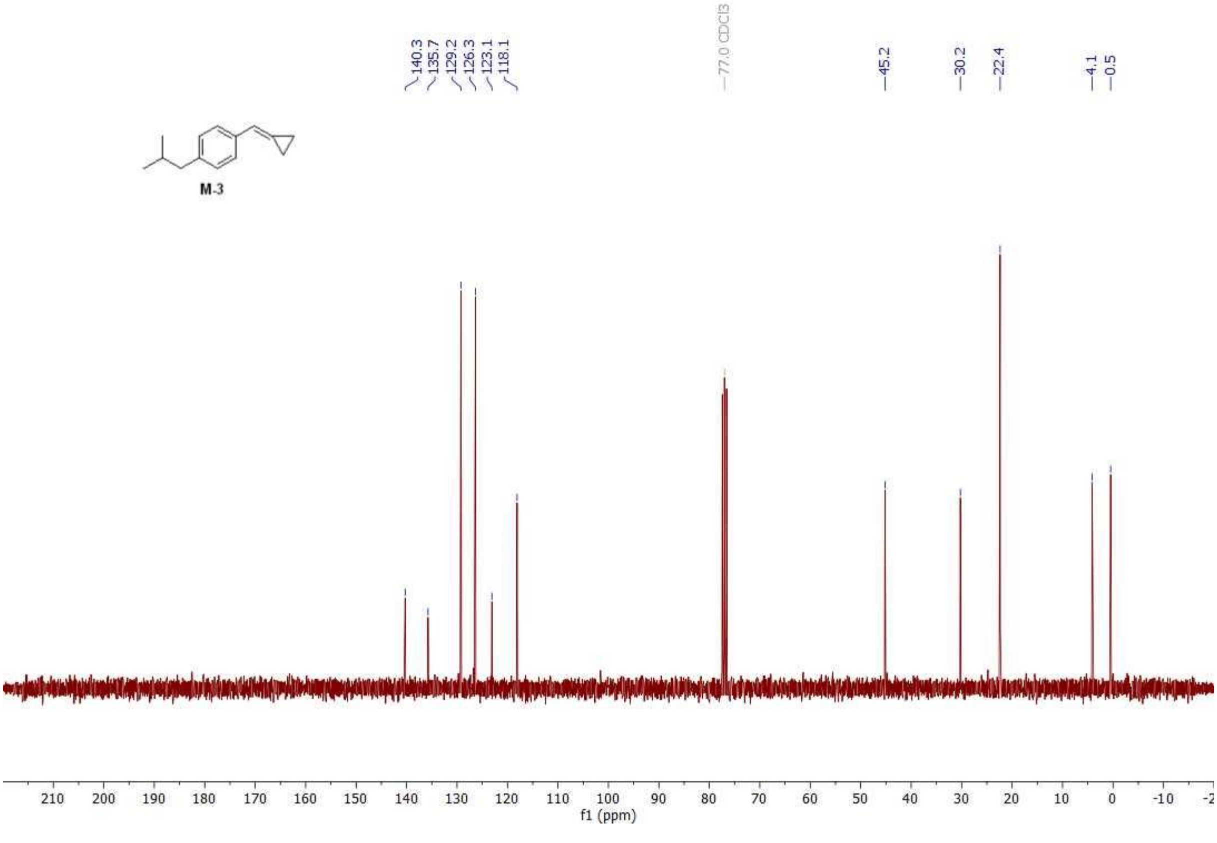

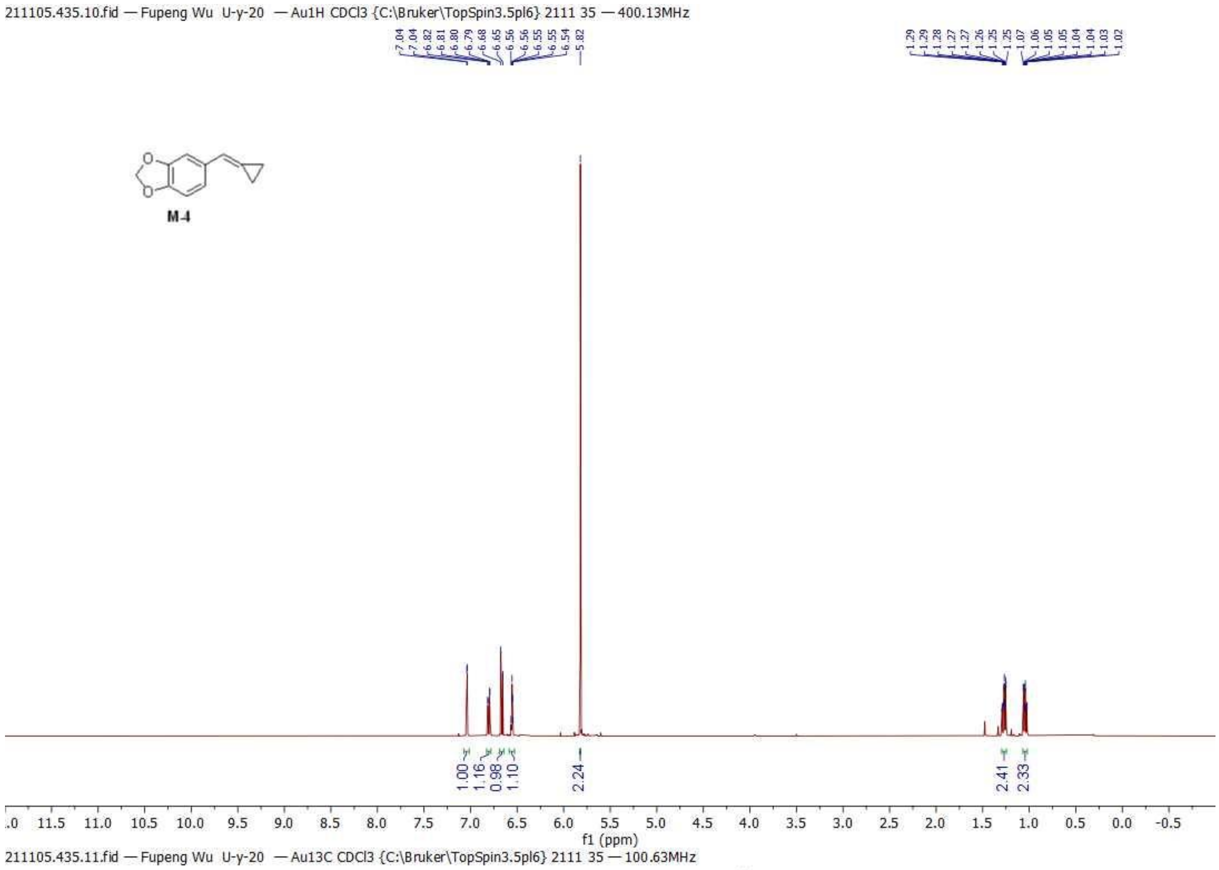
211105.435.10.fid — Fupeng Wu U-y-20 — Au1H CDCl3 {C:\Bruker\TopSpin3.5pl6} 2111 35 — 400.13MHz
M-4
f1 (ppm)

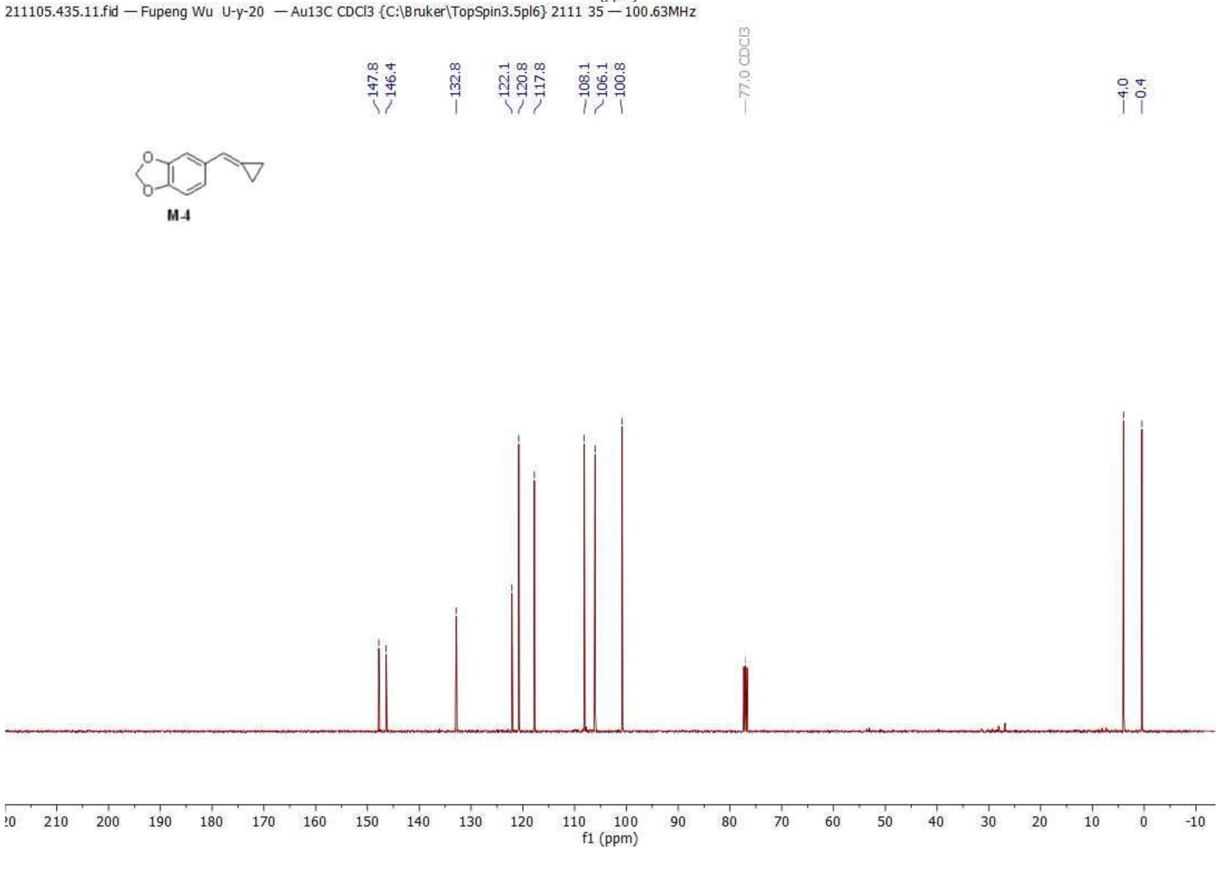
211105.435.11.fid — Fupeng Wu U-y-20 — Au13C CDCl3 {C:\Bruker\TopSpin3.5pl6} 2111 35 — 100.63MHz
M-4
f1 (ppm)

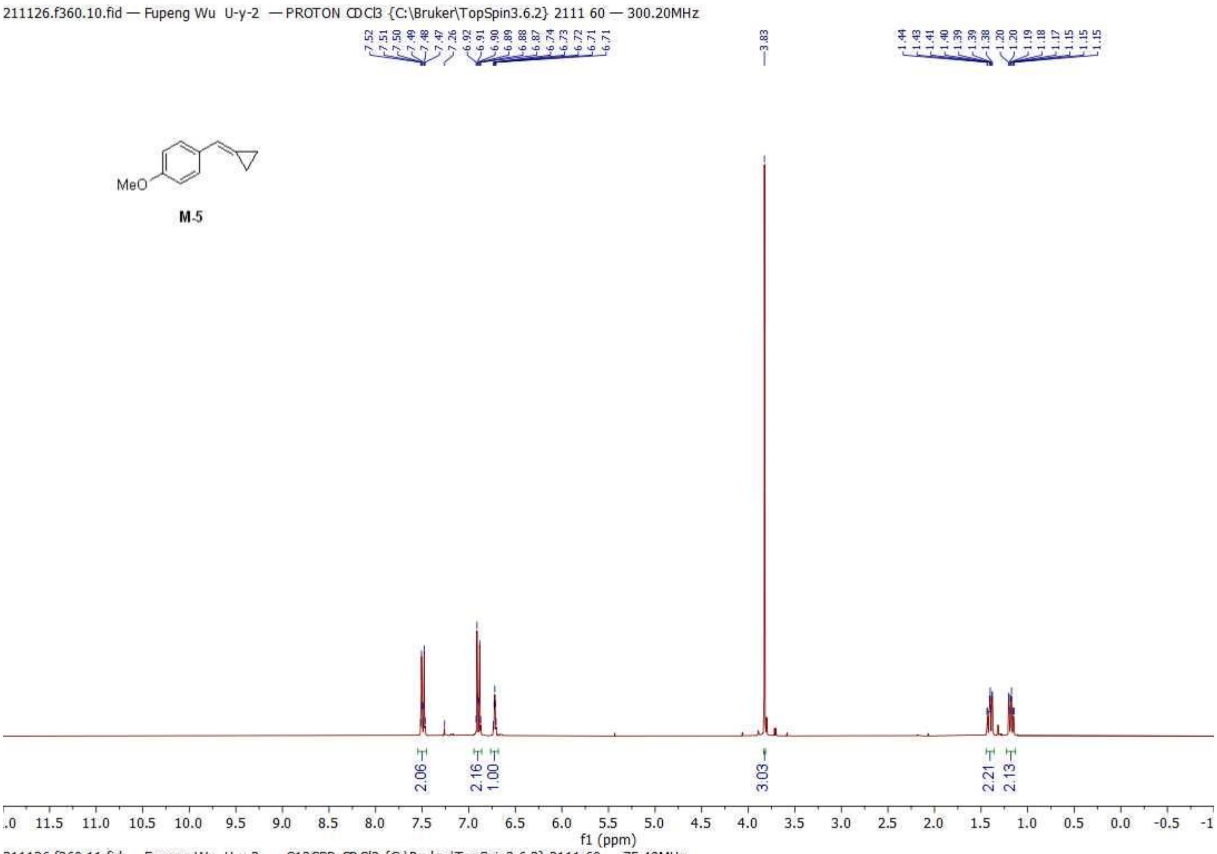
211126.f360.10.fid — Fupeng Wu U-y-2 — PROTON CDCl3 {C:\Bruker\TopSpin3.6.2} 2111 60 — 300.20MHz
7.52 7.51 7.50 7.49 7.48 7.47 7.26 6.92 6.91 6.90 6.89 6.88 6.87 6.74 6.73 6.72 6.71 6.71
3.83
1.44 1.43 1.41 1.40 1.39 1.39 1.38 1.20 1.20 1.19 1.18 1.17 1.15 1.15 1.15
MeO
M-5
2.06 2.16 1.00 3.03 2.21 2.13
f1 (ppm)

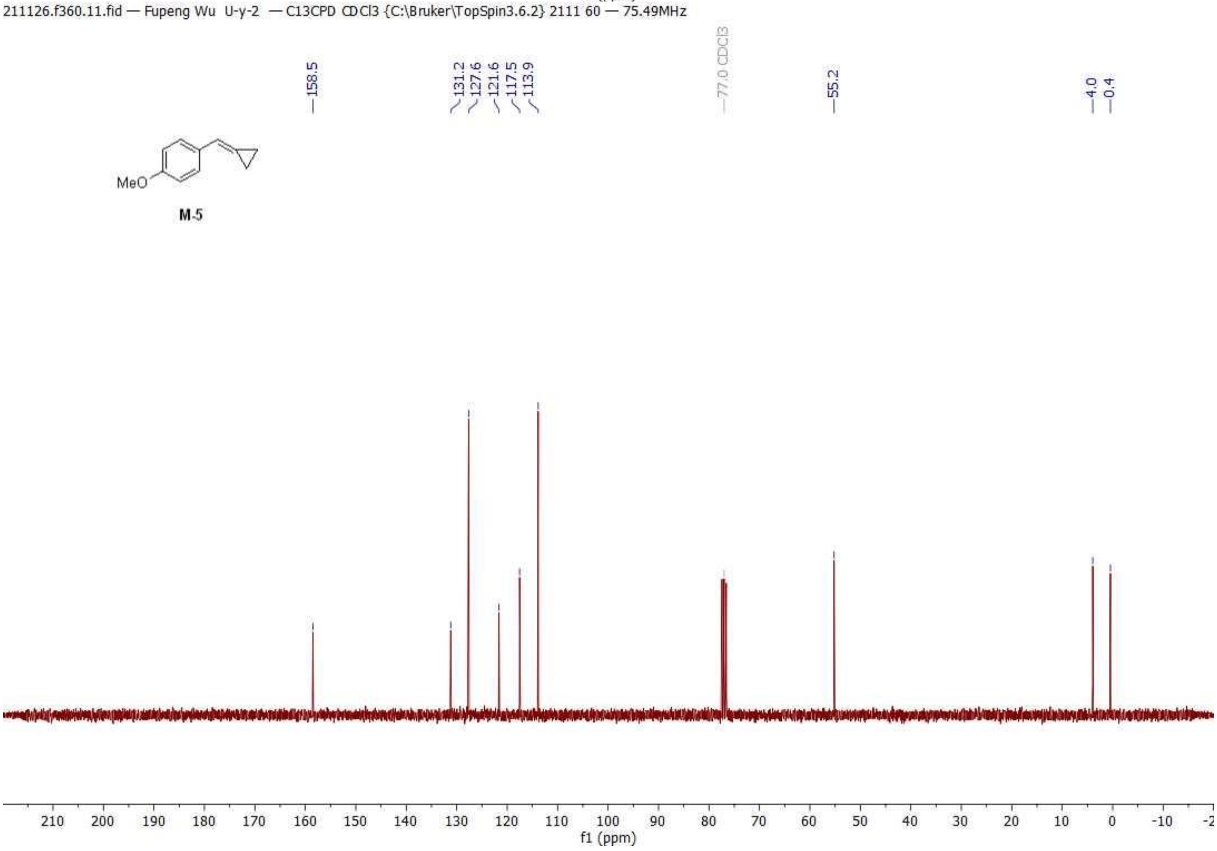
211126.f360.11.fid — Fupeng Wu U-y-2 — C13CPD CDCl3 {C:\Bruker\TopSpin3.6.2} 2111 60 — 75.49MHz
158.5
131.2 127.6 121.6 117.5 113.9
77.0 CDCl3
55.2
4.0 0.4
MeO
M-5
f1 (ppm)

211105.437.10.fid — Fupeng Wu U-y-17 — Au1H CDCl3 {C:\Bruker\TopSpin3.5pl6} 2111 37 — 400.13MHz

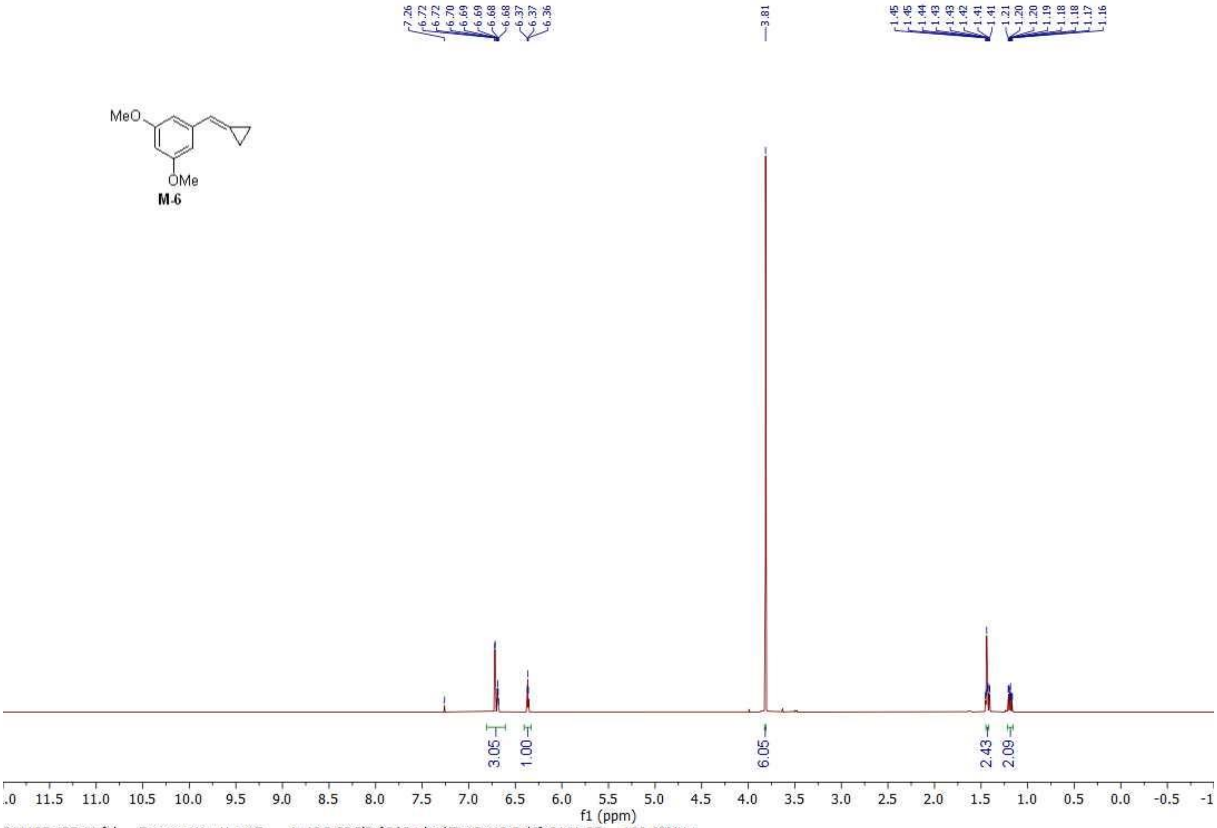

211105.437.11.fid — Fupeng Wu U-y-17 — Au13C CDCl3 {C:\Bruker\TopSpin3.5pl6} 2111 37 — 100.63MHz

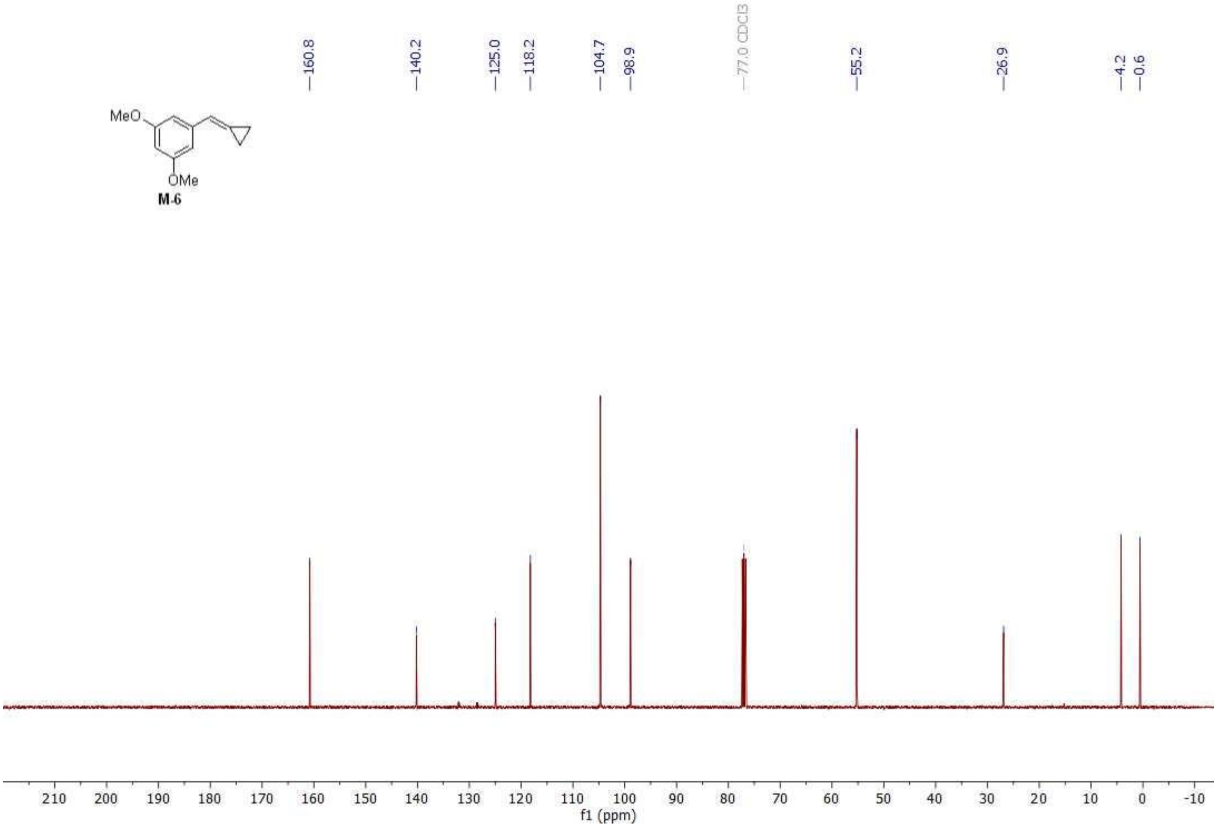

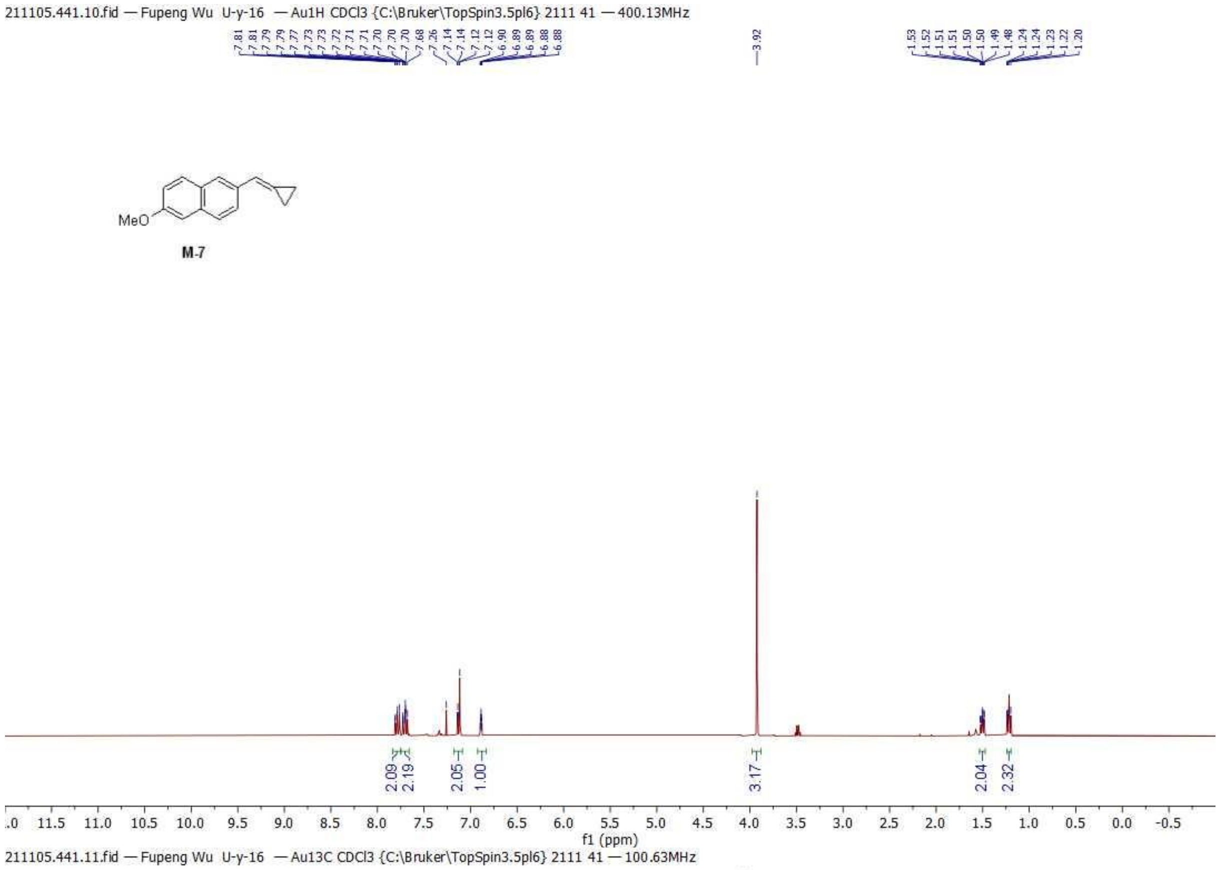
211105.441.10.fid — Fupeng Wu U-y-16 — Au1H CDCl3 {C:\Bruker\TopSpin3.5pl6} 2111 41 — 400.13MHz
7.81 7.81 7.79 7.79 7.77 7.77 7.73 7.73 7.72 7.71 7.71 7.70 7.70 7.70 7.68 7.26 7.14 7.14 7.12 7.12 6.90 6.89 6.89 6.88 6.88
3.92
1.53 1.52 1.51 1.51 1.50 1.50 1.49 1.48 1.24 1.24 1.23 1.22 1.20
MeO
M-7
2.09 2.19 2.05 1.00 3.17 2.04 2.32
f1 (ppm)

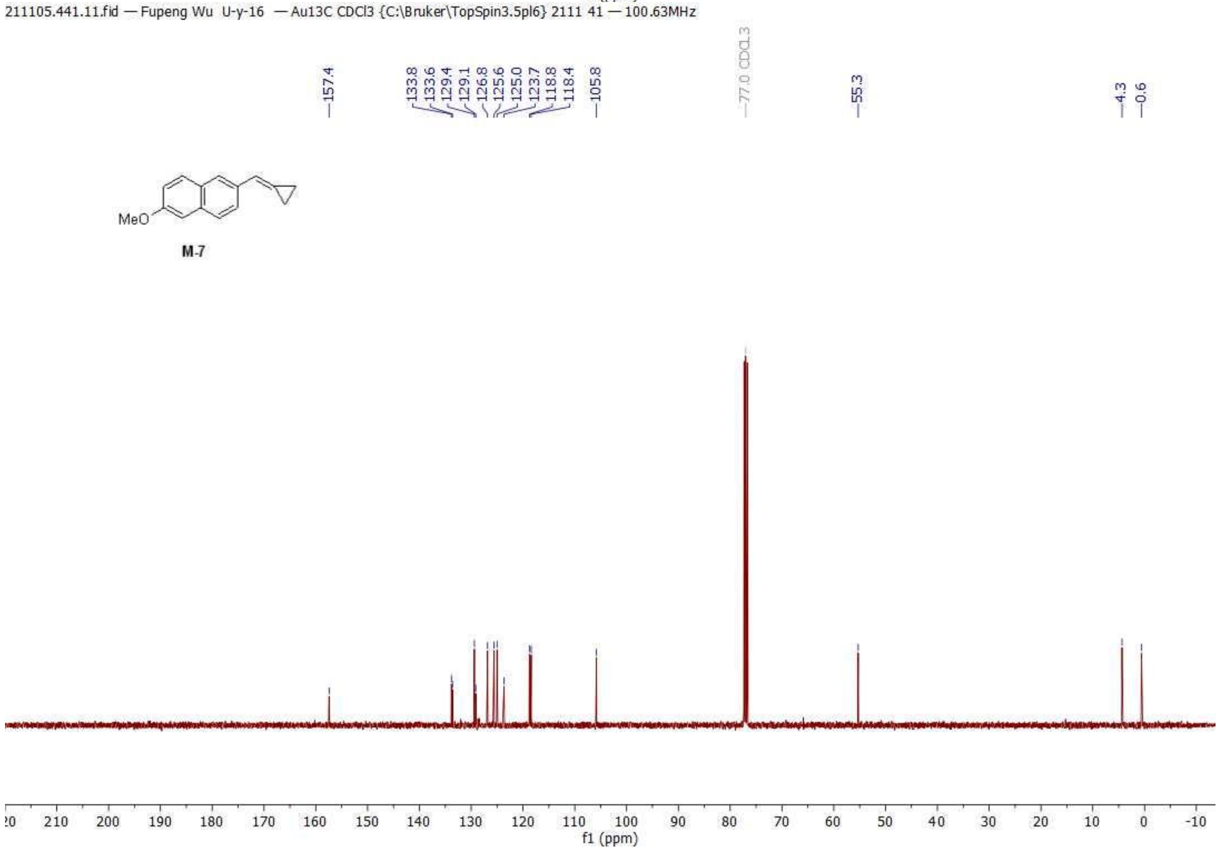
211105.441.11.fid — Fupeng Wu U-y-16 — Au13C CDCl3 {C:\Bruker\TopSpin3.5pl6} 2111 41 — 100.63MHz
157.4
133.8 133.6 129.4 129.1 126.8 125.6 125.0 123.7 118.8 118.4
105.8
77.0 CDCL3
55.3
4.3 0.6
MeO
M-7
f1 (ppm)

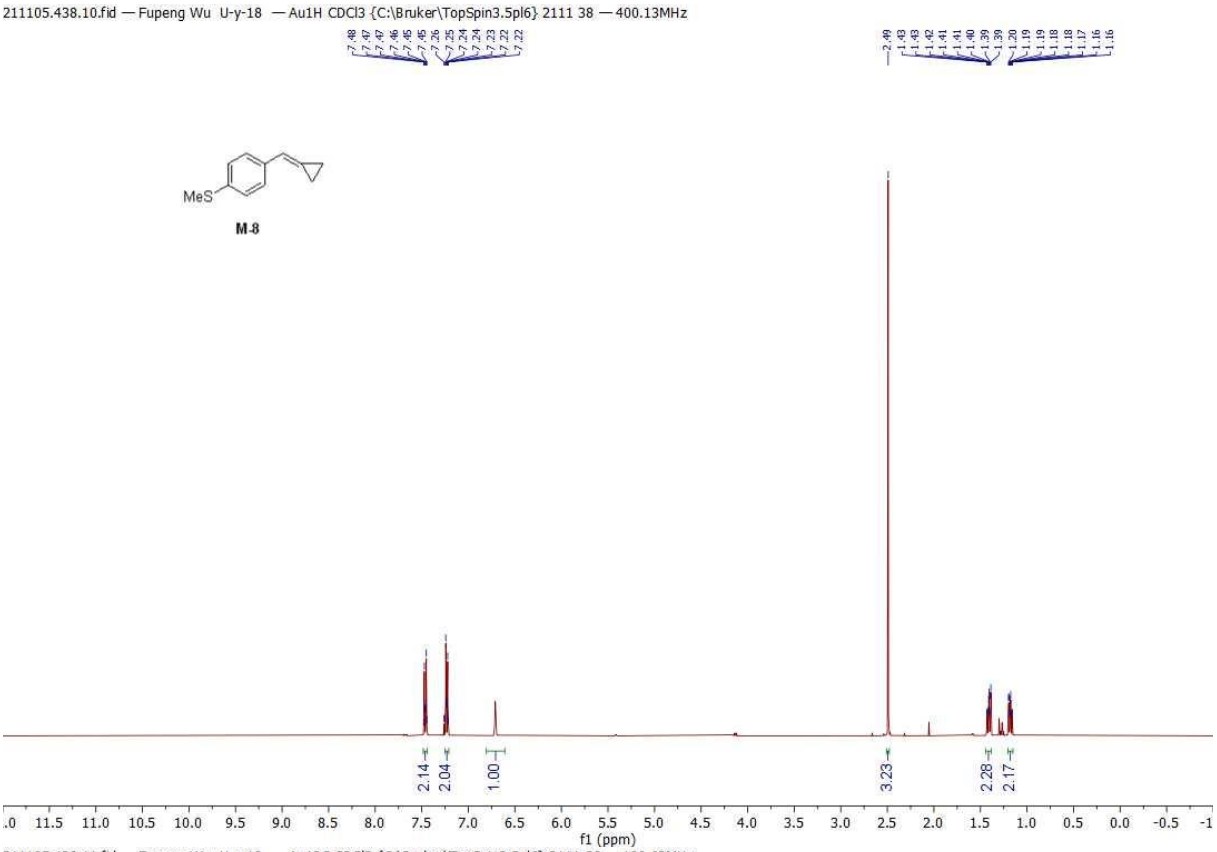
211105.438.10.fid — Fupeng Wu U-y-18 — Au1H CDCl3 {C:\Bruker\TopSpin3.5pl6} 2111 38 — 400.13MHz
7.48 7.47 7.47 7.46 7.45 7.45 7.26 7.25 7.24 7.24 7.23 7.22 7.22
2.49 1.43 1.43 1.42 1.41 1.41 1.40 1.39 1.39 1.20 1.19 1.19 1.18 1.18 1.17 1.16 1.16
MeS
M-8
2.14
2.04
1.00
3.23
2.28
2.17
f1 (ppm)

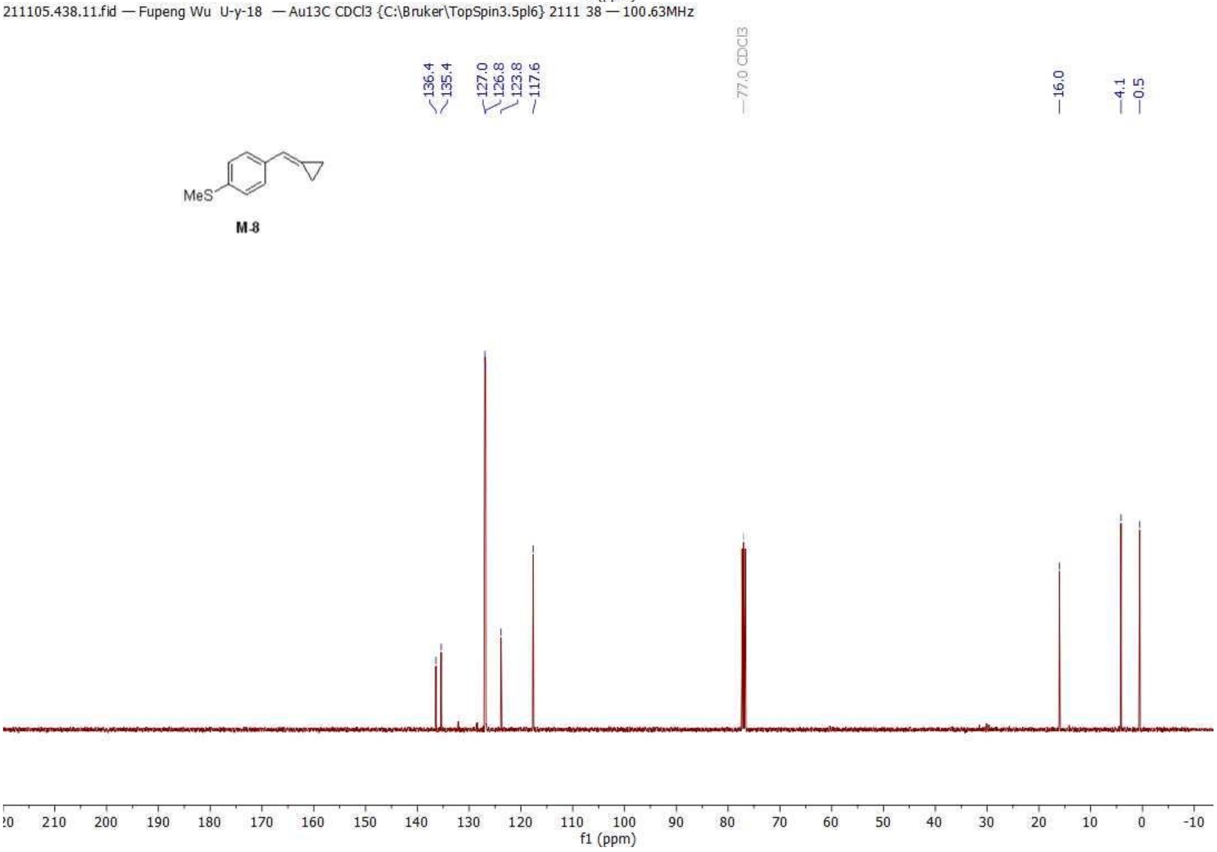
211105.438.11.fid — Fupeng Wu U-y-18 — Au13C CDCl3 {C:\Bruker\TopSpin3.5pl6} 2111 38 — 100.63MHz
136.4
135.4
127.0
126.8
123.8
117.6
77.0 CDCl3
16.0
4.1
0.5
MeS
M-8
f1 (ppm)

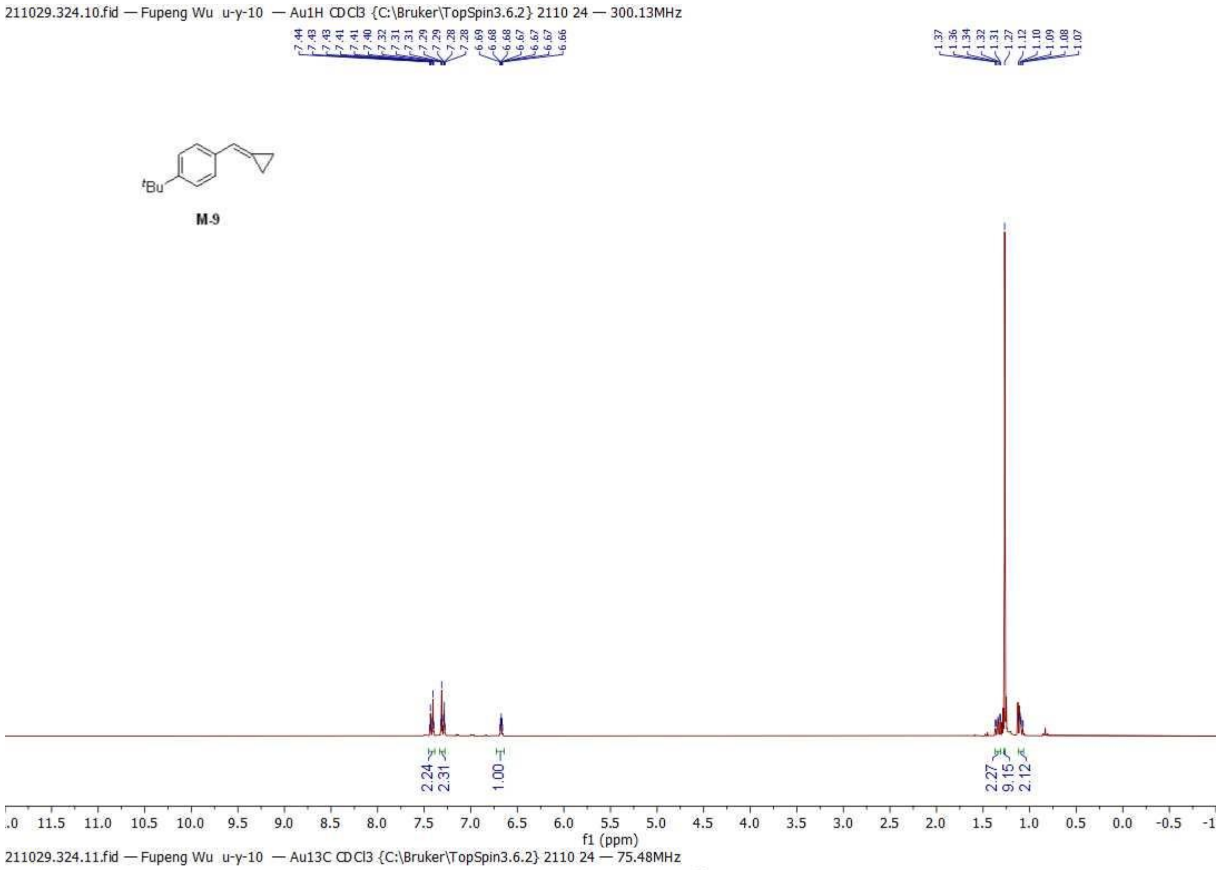
211029.324.10.fid — Fupeng Wu u-y-10 — Au1H CDCl3 {C:\Bruker\TopSpin3.6.2} 2110 24 — 300.13MHz
7.44 7.43 7.43 7.41 7.41 7.40 7.32 7.31 7.31 7.29 7.29 7.28 7.28 6.69 6.68 6.68 6.67 6.67 6.67 6.66
1.37 1.36 1.34 1.32 1.31 1.27 1.12 1.10 1.09 1.08 1.07
^{t}Bu
M-9
2.24 2.31 1.00 2.27 9.15 2.12
f1 (ppm)

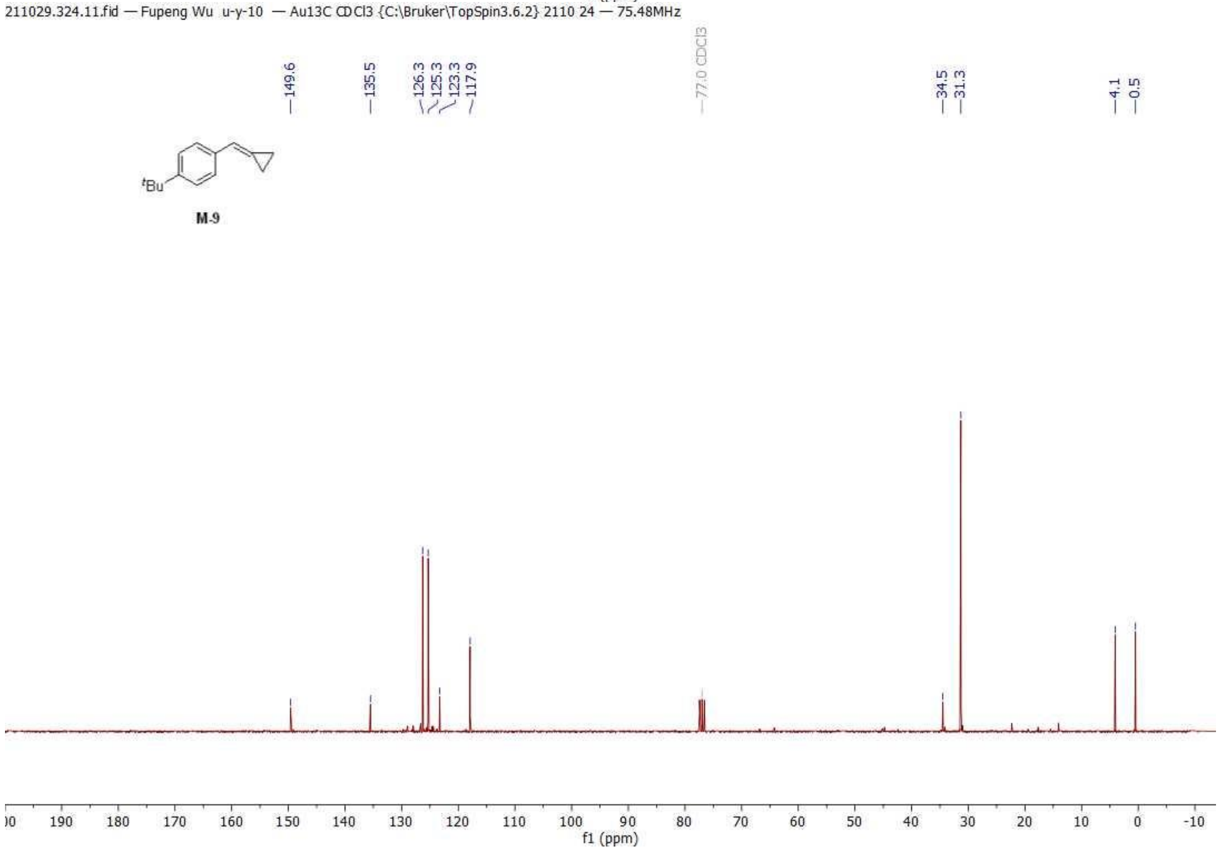
211029.324.11.fid — Fupeng Wu u-y-10 — Au13C CDCl3 {C:\Bruker\TopSpin3.6.2} 2110 24 — 75.48MHz
149.6 135.5 126.3 125.3 123.3 117.9 77.0 CDCl3 34.5 31.3 4.1 0.5
^{t}Bu
M-9
f1 (ppm)

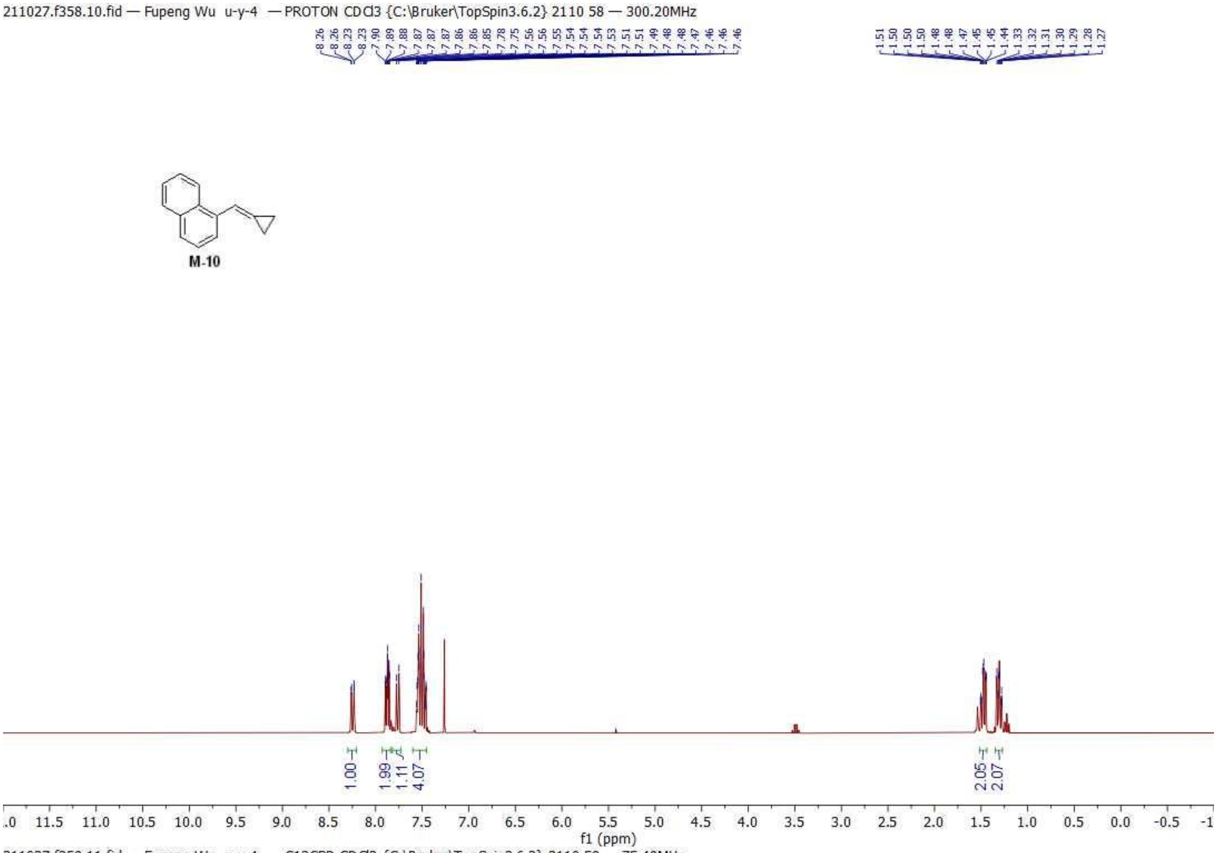
211027.f358.10.fid — Fupeng Wu u-y-4 — PROTON CDCl3 {C:\Bruker\TopSpin3.6.2} 2110 58 — 300.20MHz
8.26 8.26 8.23 8.23 7.90 7.89 7.88 7.87 7.87 7.86 7.86 7.85 7.78 7.75 7.56 7.56 7.55 7.54 7.54 7.54 7.53 7.51 7.51 7.49 7.48 7.48 7.47 7.46 7.46 7.46
1.51 1.50 1.50 1.48 1.48 1.47 1.45 1.45 1.44 1.33 1.32 1.31 1.30 1.29 1.28 1.27
M-10
1.00 1.99 1.11 4.07 2.05 2.07
f1 (ppm)

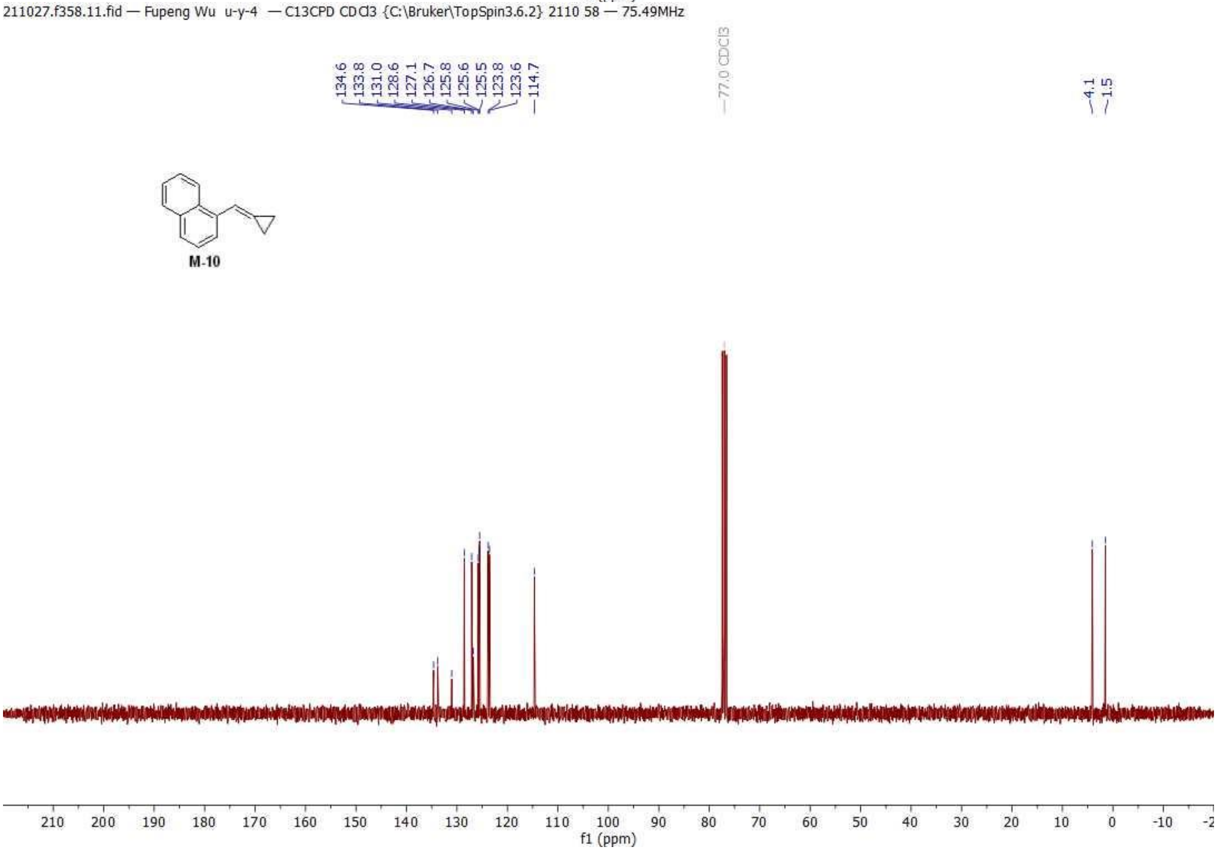
211027.f358.11.fid — Fupeng Wu u-y-4 — C13CPD CDCl3 {C:\Bruker\TopSpin3.6.2} 2110 58 — 75.49MHz
134.6 133.8 131.0 128.6 127.1 126.7 125.8 125.6 125.5 123.8 123.6 114.7
77.0 CDCl3
4.1 1.5
M-10
f1 (ppm)

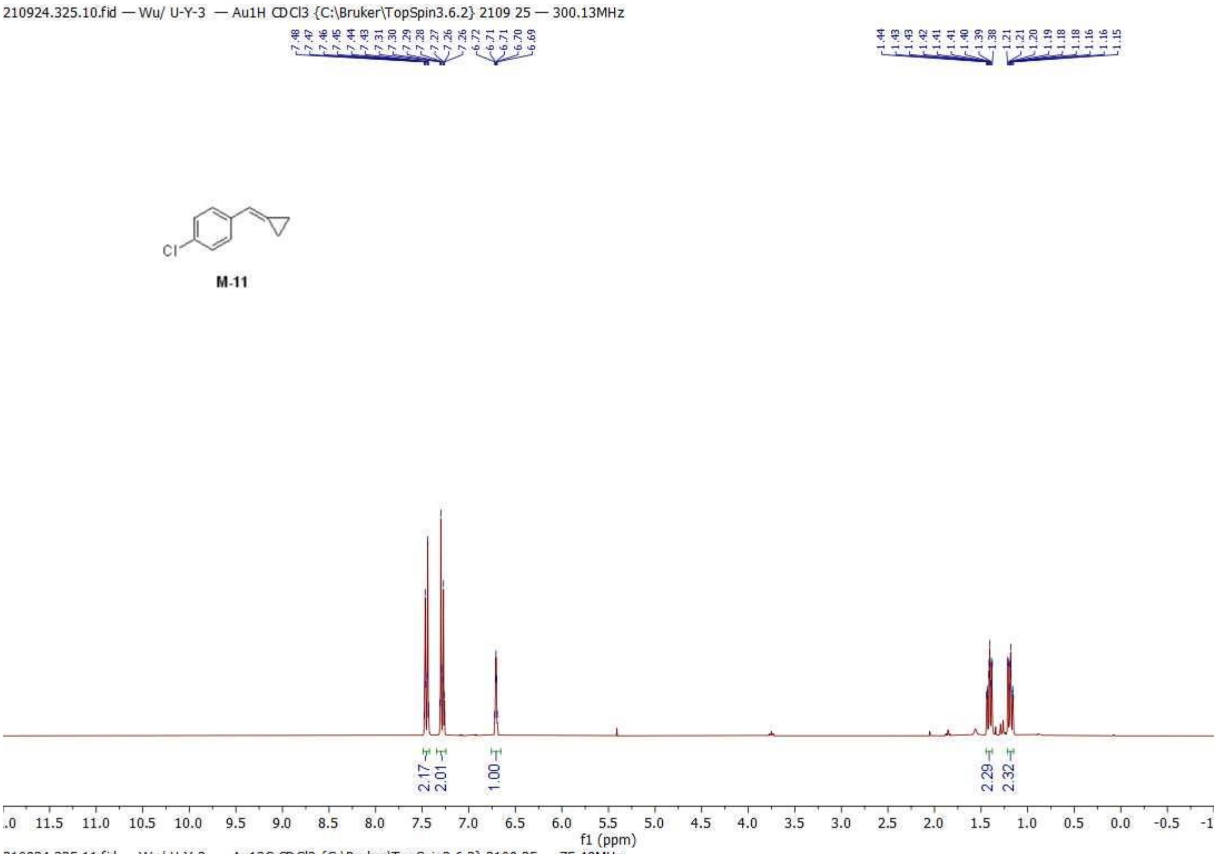
210924.325.10.fid — Wu/ U-Y-3 — Au1H CDCl3 {C:\Bruker\TopSpin3.6.2} 2109 25 — 300.13MHz
7.48 7.47 7.46 7.45 7.44 7.43 7.31 7.30 7.29 7.28 7.27 7.26 7.26 6.72 6.71 6.71 6.70 6.69
1.44 1.43 1.43 1.42 1.41 1.41 1.40 1.39 1.38 1.21 1.21 1.20 1.19 1.18 1.18 1.16 1.15
Cl
M-11
2.17 2.01 1.00 2.29 2.32
f1 (ppm)

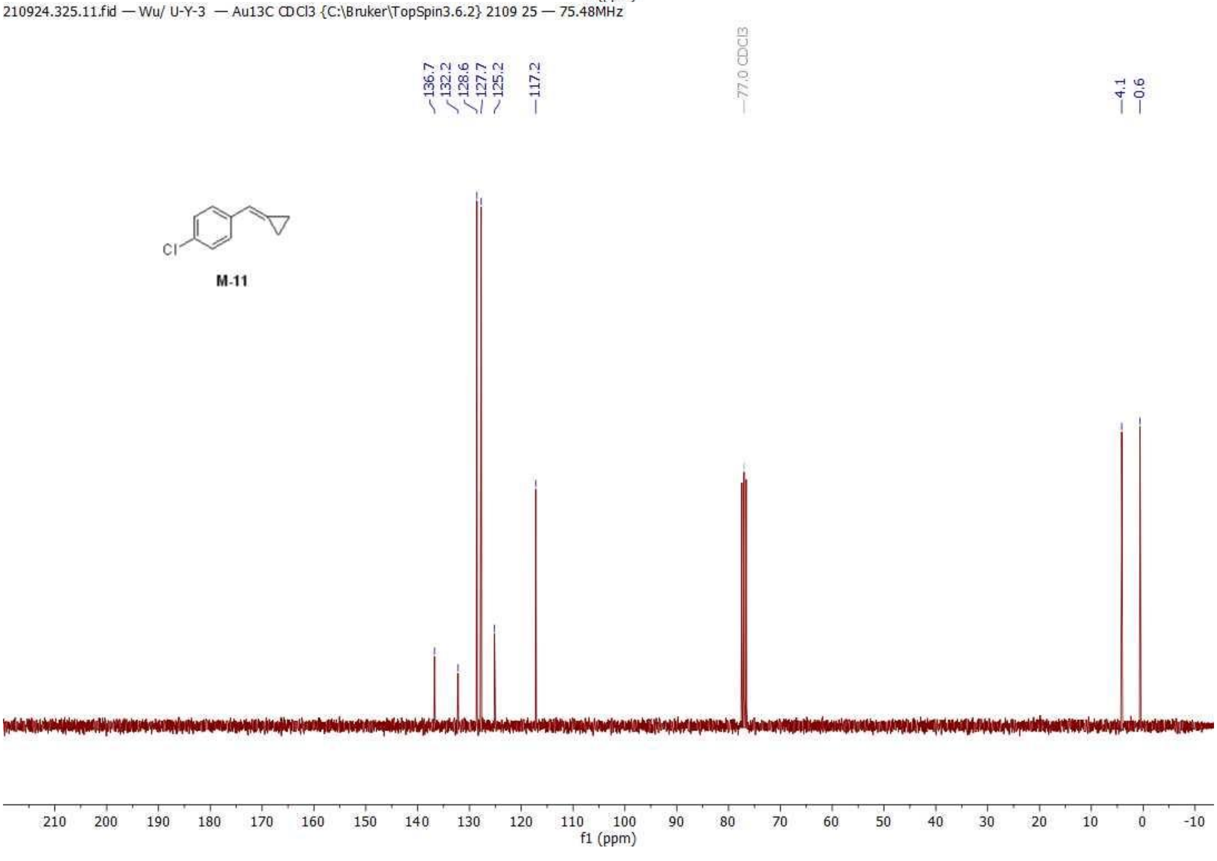
210924.325.11.fid — Wu/ U-Y-3 — Au13C CDCl3 {C:\Bruker\TopSpin3.6.2} 2109 25 — 75.48MHz
136.7 132.2 128.6 127.7 125.2 117.2
77.0 CDCl3
4.1 0.6
Cl
M-11
f1 (ppm)

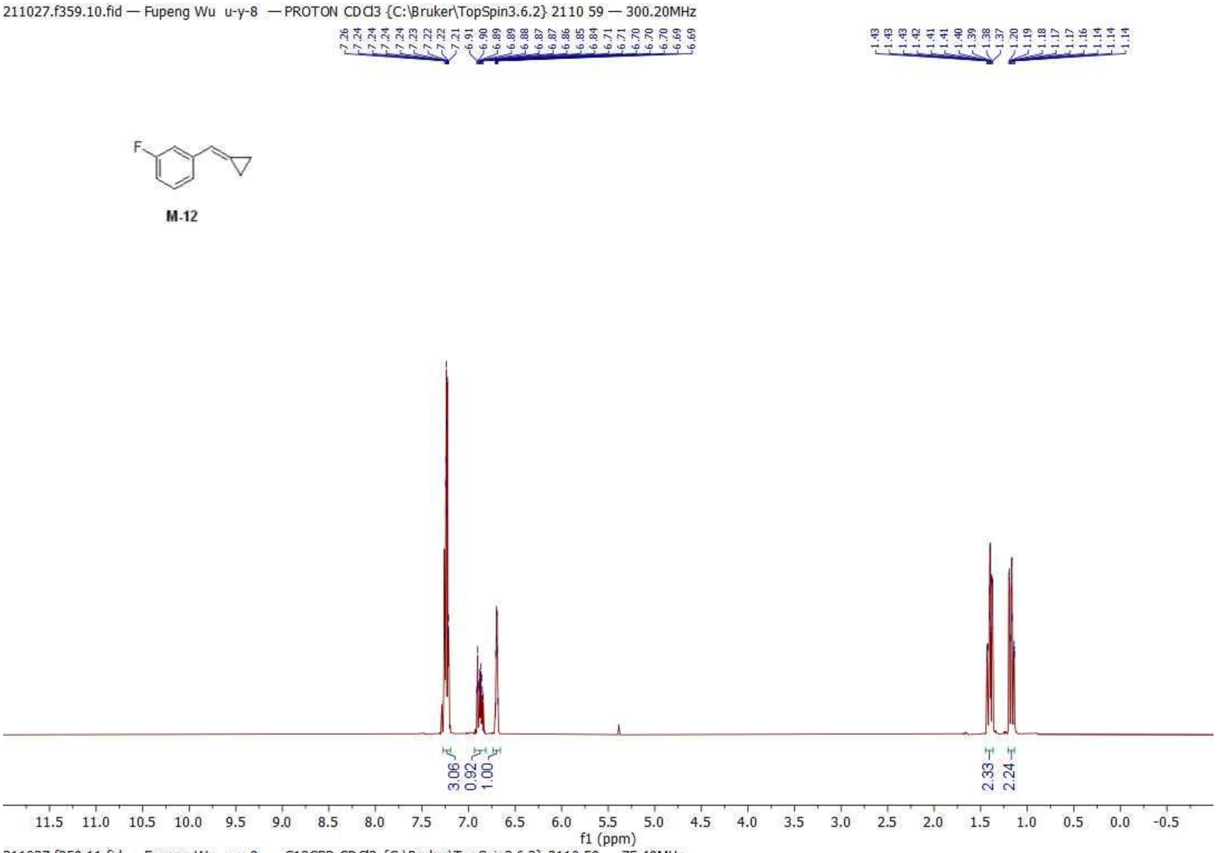
211027.f359.10.fid — Fupeng Wu u-y-8 — PROTON CDCl3 {C:\Bruker\TopSpin3.6.2} 2110 59 — 300.20MHz
7.26 7.24 7.24 7.24 7.23 7.22 7.22 7.21 6.91 6.90 6.89 6.88 6.87 6.87 6.86 6.85 6.84 6.71 6.70 6.70 6.69 6.69
1.43 1.43 1.43 1.42 1.41 1.41 1.40 1.39 1.38 1.37 1.20 1.19 1.18 1.17 1.16 1.14 1.14 1.14
F
M-12
3.06 0.92 1.00
2.33 2.24
11.5 11.0 10.5 10.0 9.5 9.0 8.5 8.0 7.5 7.0 6.5 6.0 5.5 5.0 4.5 4.0 3.5 3.0 2.5 2.0 1.5 1.0 0.5 0.0 -0.5
f1 (ppm)

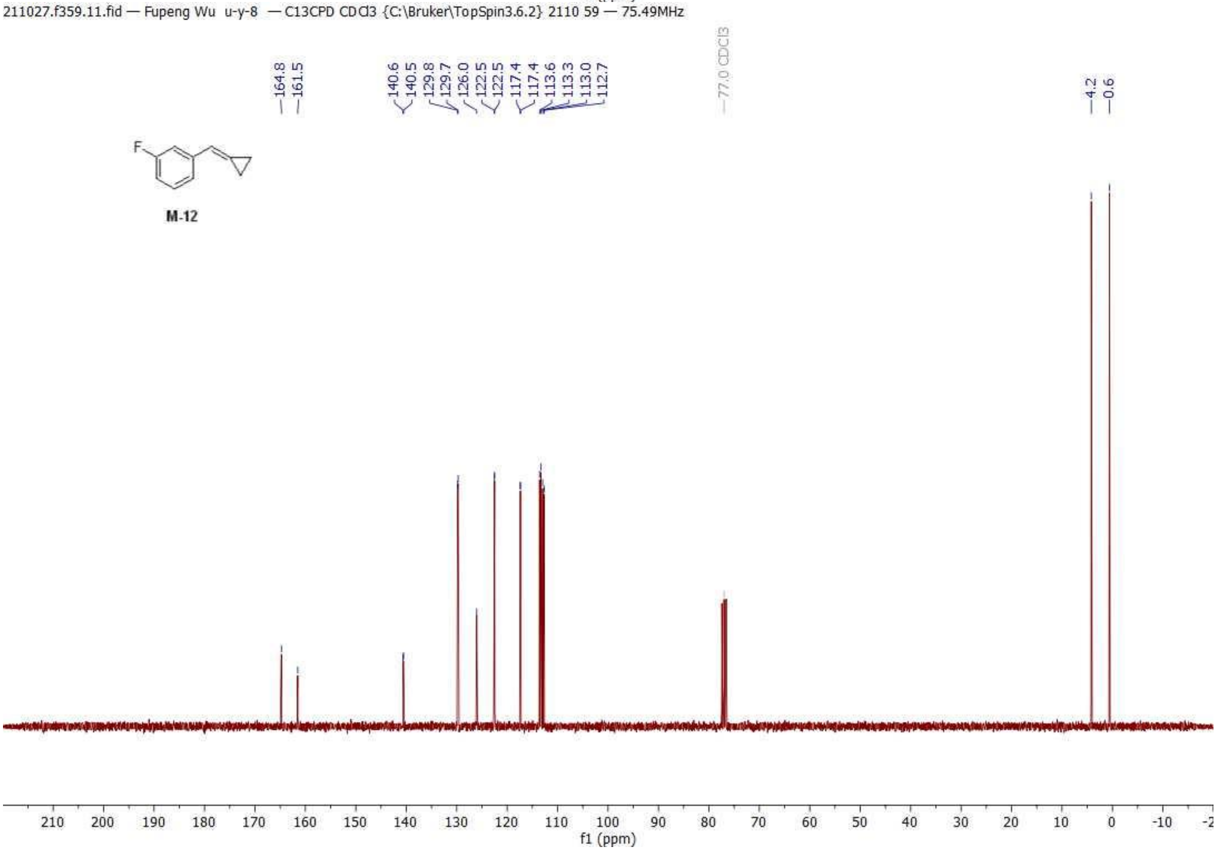
211027.f359.11.fid — Fupeng Wu u-y-8 — C13CPD CDCl3 {C:\Bruker\TopSpin3.6.2} 2110 59 — 75.49MHz
164.8 161.5
140.6 140.5 129.8 129.7 126.0 122.5 122.5 117.4 117.4 113.6 113.3 113.0 112.7
77.0 CDCl3
4.2 0.6
F
M-12
210 200 190 180 170 160 150 140 130 120 110 100 90 80 70 60 50 40 30 20 10 0 -10 -2
f1 (ppm)

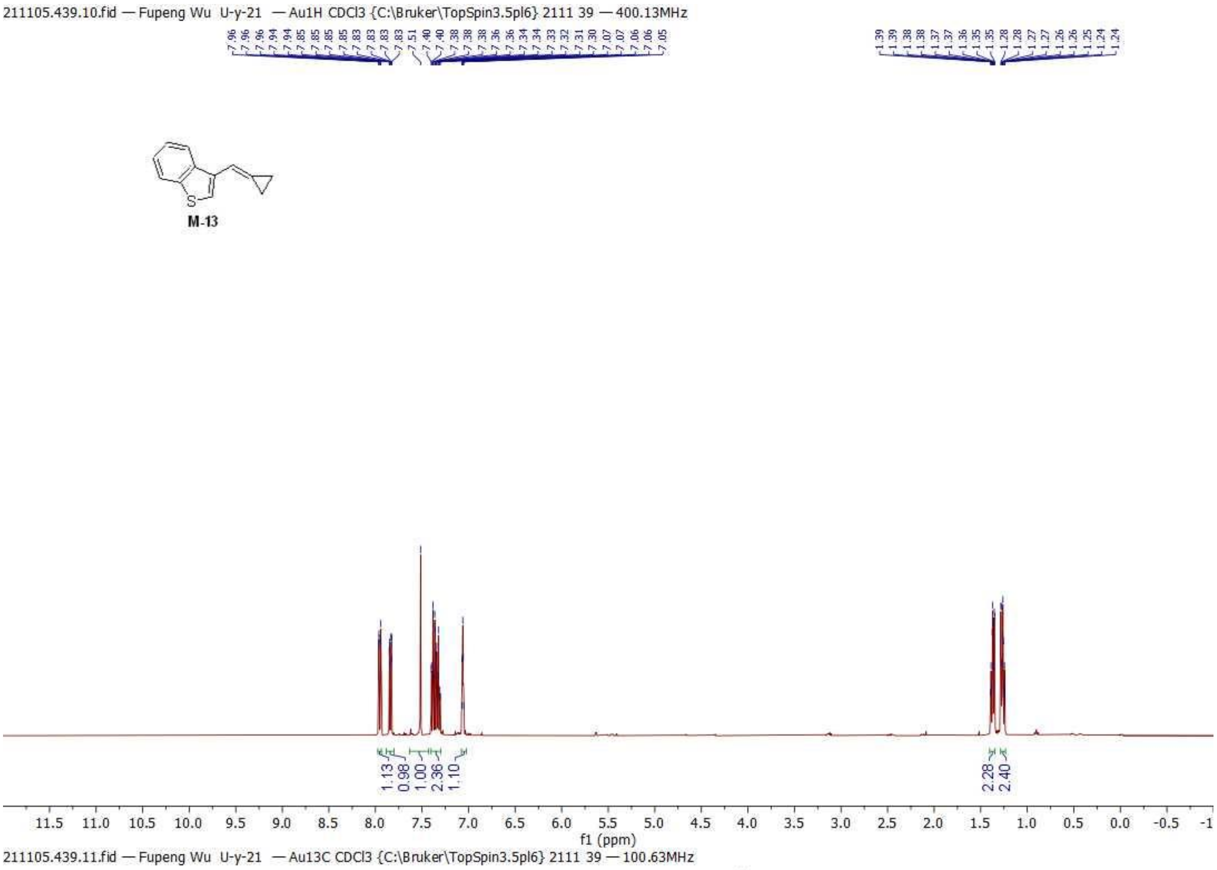
211105.439.10.fid — Fupeng Wu U-y-21 — Au1H CDCl3 {C:\Bruker\TopSpin3.5pl6} 2111 39 — 400.13MHz
7.96 7.96 7.96 7.94 7.94 7.85 7.85 7.85 7.85 7.83 7.83 7.83 7.83 7.51 7.40 7.40 7.38 7.38 7.38 7.36 7.36 7.34 7.34 7.33 7.32 7.31 7.30 7.07 7.06 7.06 7.05
1.39 1.39 1.38 1.38 1.37 1.37 1.36 1.35 1.35 1.28 1.28 1.27 1.27 1.26 1.26 1.25 1.24 1.24
S
M-13
1.13 0.98 1.00 2.36 1.10
2.28 2.40
11.5 11.0 10.5 10.0 9.5 9.0 8.5 8.0 7.5 7.0 6.5 6.0 5.5 5.0 4.5 4.0 3.5 3.0 2.5 2.0 1.5 1.0 0.5 0.0 -0.5 -1
f1 (ppm)

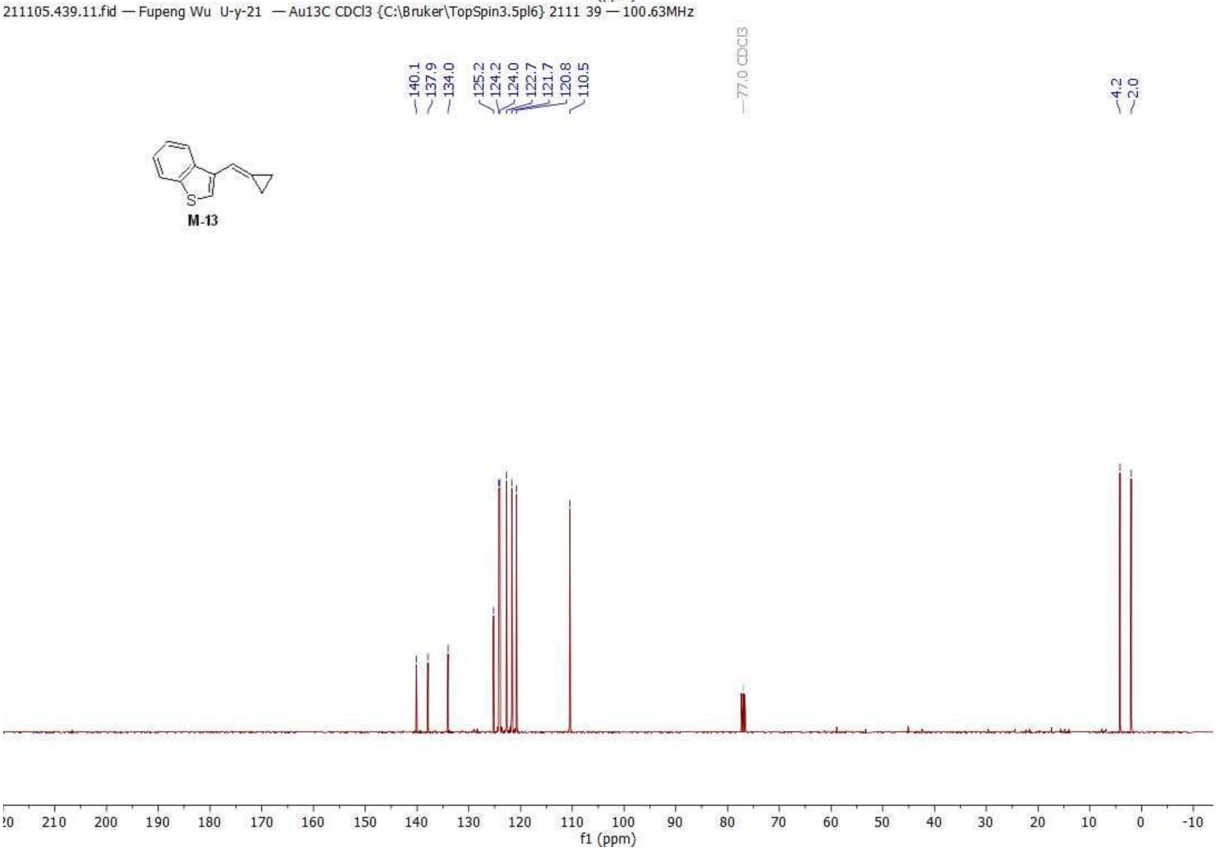
211105.439.11.fid — Fupeng Wu U-y-21 — Au13C CDCl3 {C:\Bruker\TopSpin3.5pl6} 2111 39 — 100.63MHz
140.1 137.9 134.0 125.2 124.2 124.0 122.7 121.7 120.8 110.5
77.0 CDCl3
4.2 2.0
S
M-13
210 200 190 180 170 160 150 140 130 120 110 100 90 80 70 60 50 40 30 20 10 0 -10
f1 (ppm)

211129.448.10.fid — Fupeng Wu U-y-28 — Au1H CDCl3 {C:\Bruker\TopSpin3.5pl6} 2111 48 — 400.13MHz

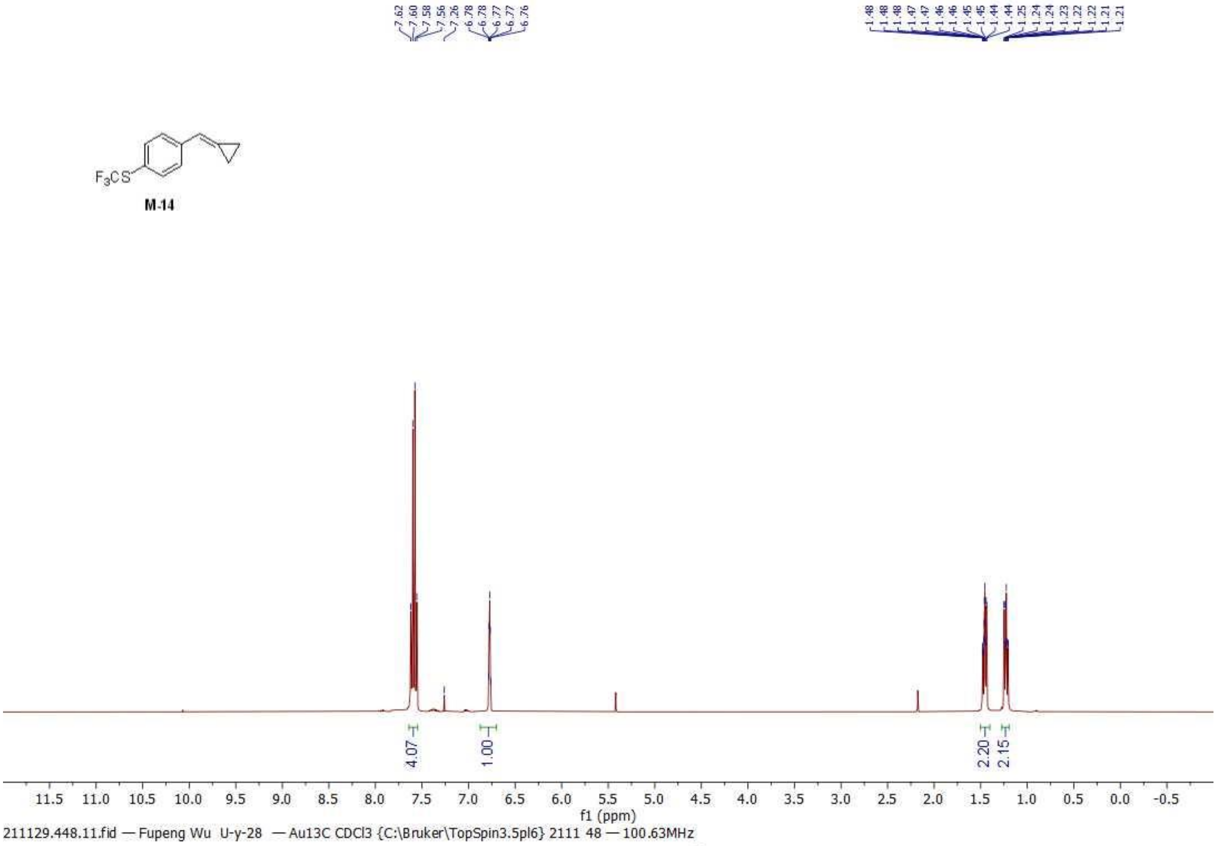

211129.448.11.fid — Fupeng Wu U-y-28 — Au13C CDCl3 {C:\Bruker\TopSpin3.5pl6} 2111 48 — 100.63MHz

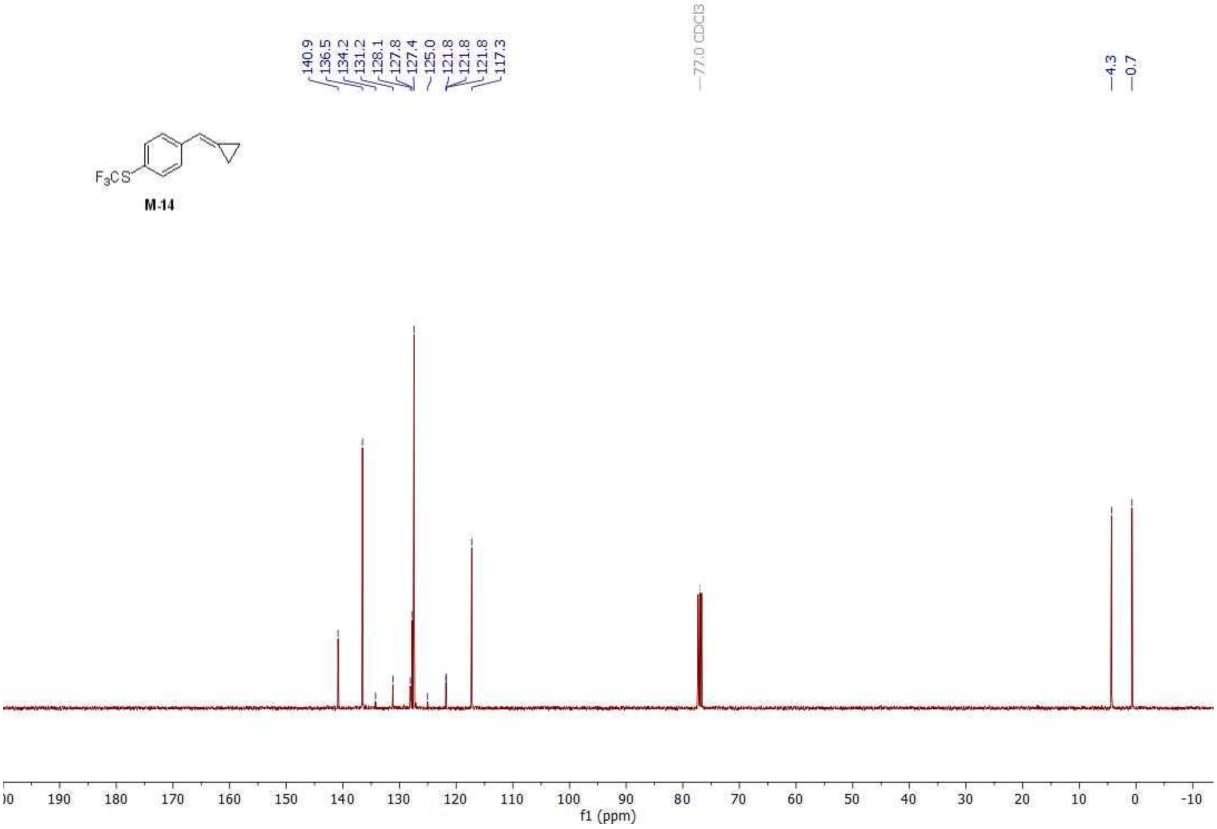

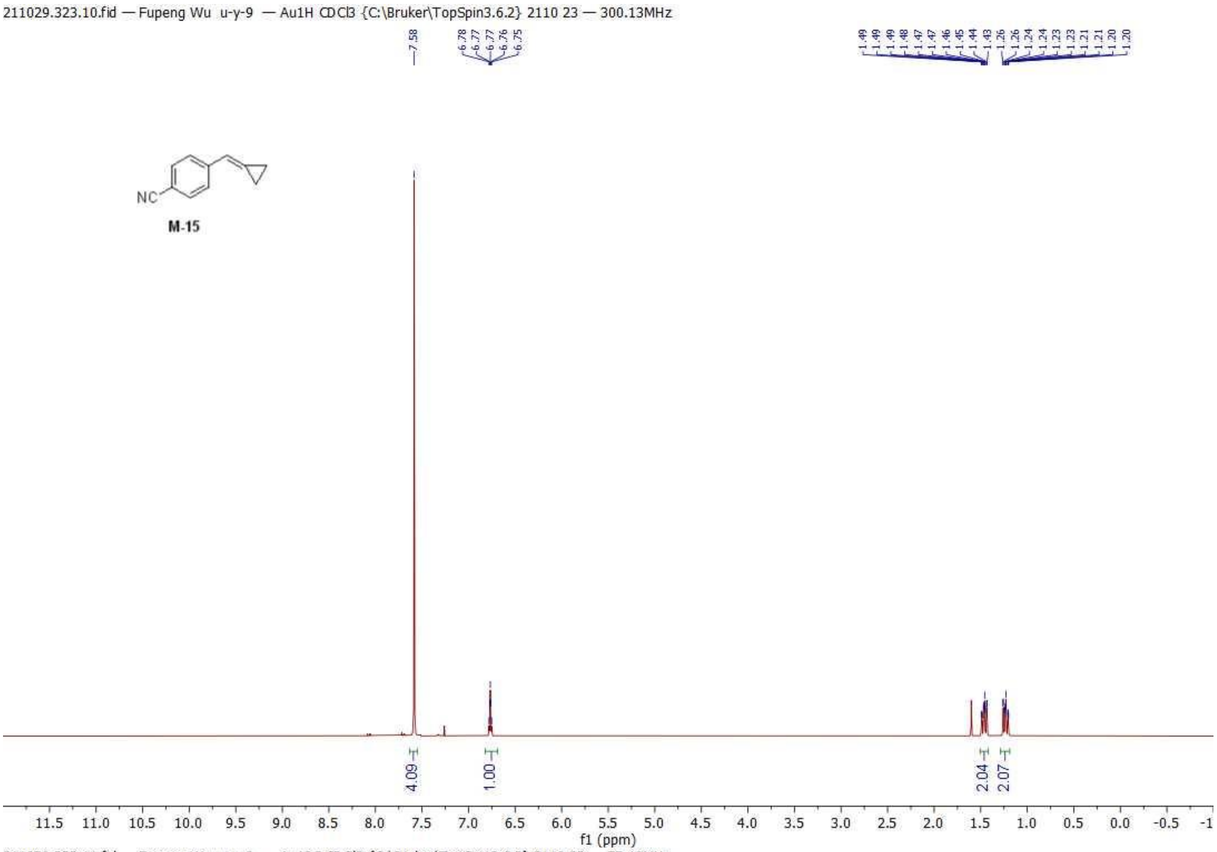
211029.323.10.fid — Fupeng Wu u-y-9 — Au1H CDCl3 {C:\Bruker\TopSpin3.6.2} 2110 23 — 300.13MHz
7.58
6.78
6.77
6.77
6.76
6.75
1.49
1.49
1.49
1.48
1.47
1.47
1.46
1.45
1.44
1.43
1.26
1.26
1.24
1.24
1.23
1.23
1.21
1.21
1.20
1.20
NC
M-15
4.09
1.00
2.04
2.07
f1 (ppm)

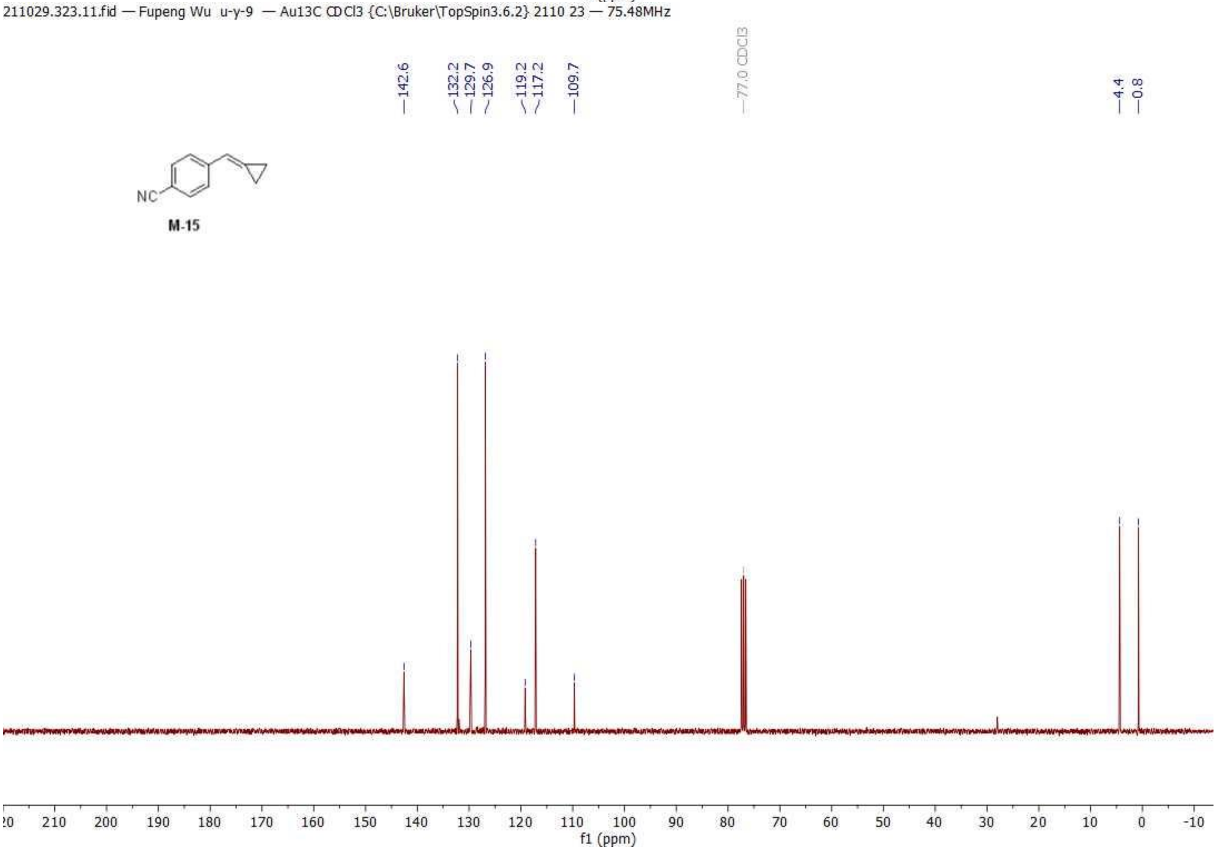
211029.323.11.fid — Fupeng Wu u-y-9 — Au13C CDCl3 {C:\Bruker\TopSpin3.6.2} 2110 23 — 75.48MHz
142.6
132.2
129.7
126.9
119.2
117.2
109.7
77.0 CDCl3
4.4
0.8
NC
M-15
f1 (ppm)

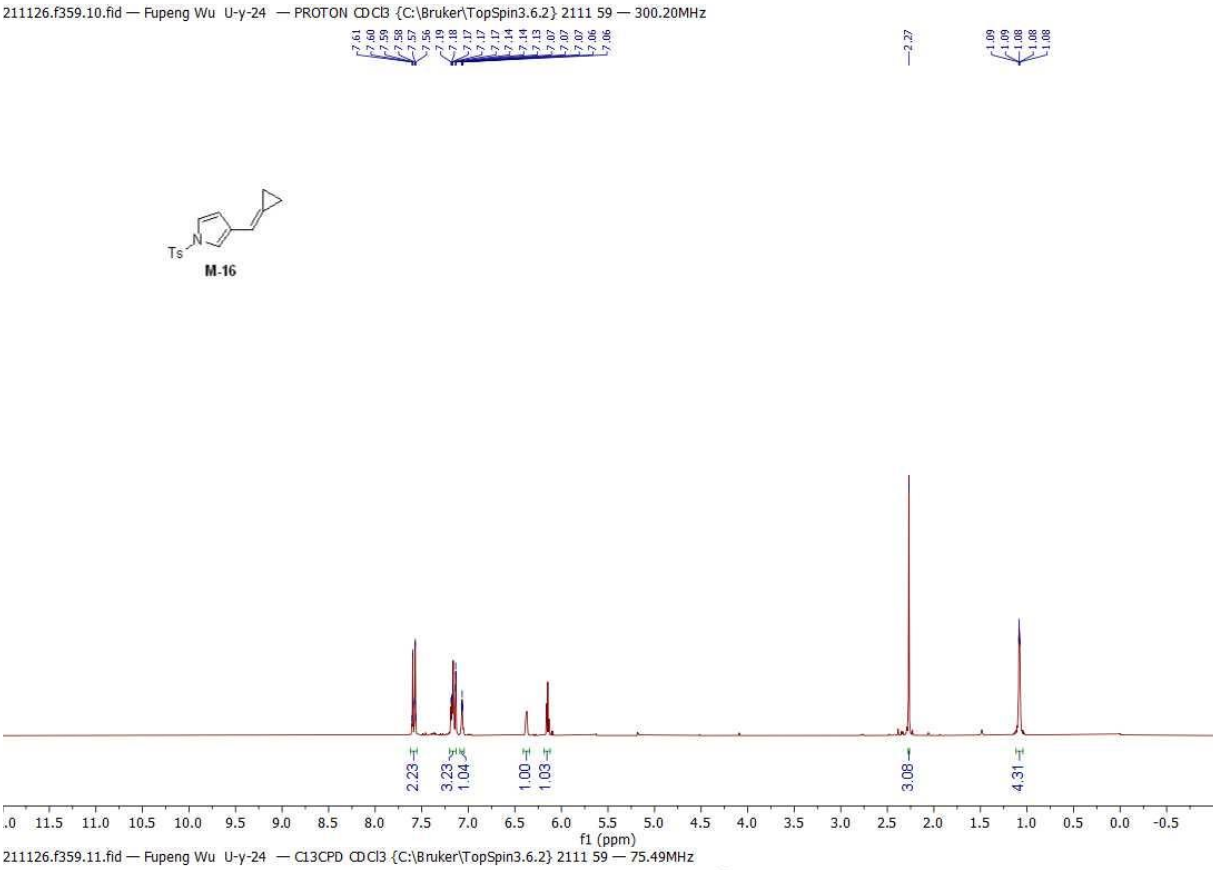
211126.f359.10.fid — Fupeng Wu U-y-24 — PROTON CDCl3 {C:\Bruker\TopSpin3.6.2} 2111 59 — 300.20MHz
7.61 7.60 7.59 7.58 7.57 7.56 7.19 7.18 7.17 7.17 7.14 7.14 7.13 7.07 7.07 7.06 7.06
2.27
1.09 1.09 1.08 1.08 1.08
Ts
N
M-16
2.23
3.23
1.04
1.00
1.03
3.08
4.31
f1 (ppm)

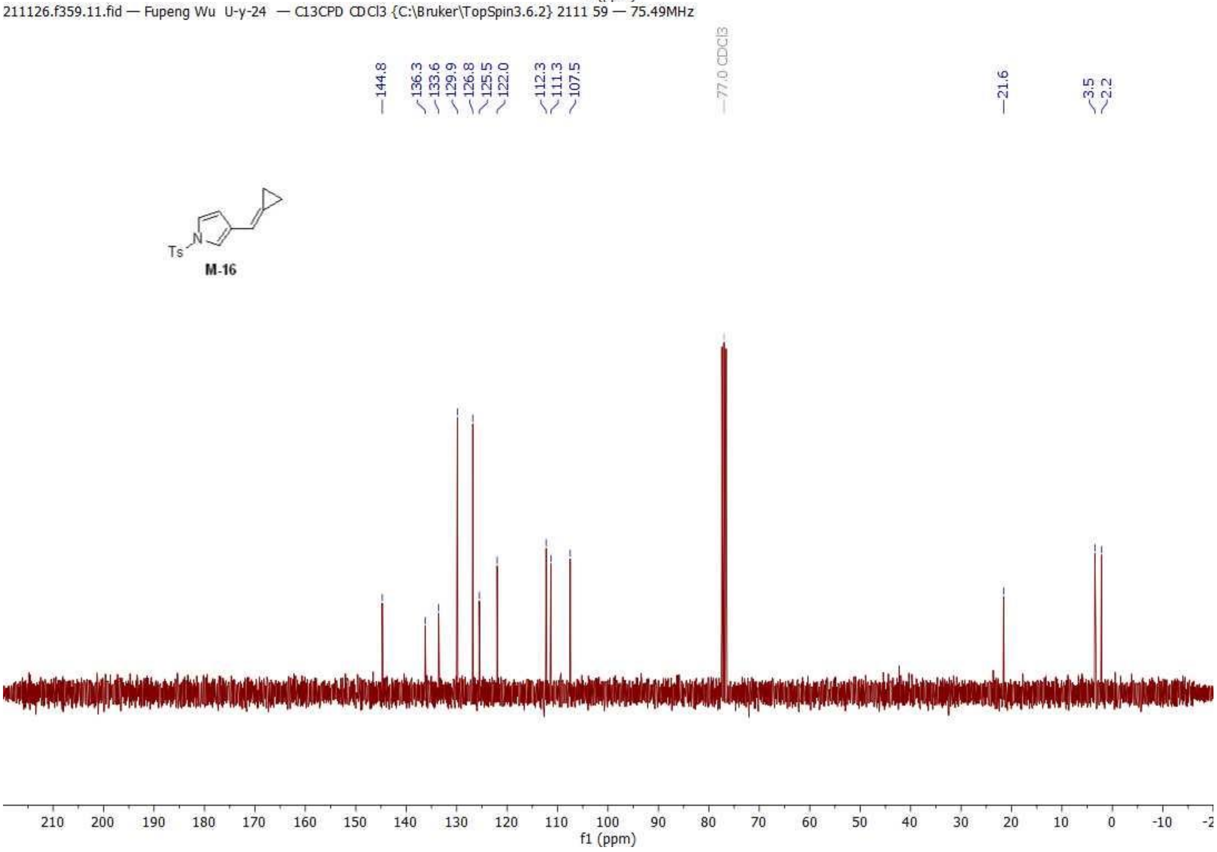
211126.f359.11.fid — Fupeng Wu U-y-24 — C13CPD CDCl3 {C:\Bruker\TopSpin3.6.2} 2111 59 — 75.49MHz
144.8
136.3 133.6 129.9 126.8 125.5 122.0
112.3 111.3 107.5
77.0 CDCl3
21.6
3.5 2.2
Ts
N
M-16
f1 (ppm)

9. Crystallographic reports for the x-ray structures

3a

checkCIF/PLATON report

Structure factors have been supplied for datablock(s) AX2220

THIS REPORT IS FOR GUIDANCE ONLY. IF USED AS PART OF A REVIEW PROCEDURE FOR PUBLICATION, IT SHOULD NOT REPLACE THE EXPERTISE OF AN EXPERIENCED CRYSTALLOGRAPHIC REFEREE.

No syntax errors found. CIF dictionary Interpreting this report

Datablock: AX2220

Bond precision: C-C = 0.0018 A Wavelength=0.71073

Cell:	a=12.4943(10)	b=9.9586(7)	c=16.7620(13)
	alpha=90	beta=95.6616(15)	gamma=90
Temperature:	150 K		

	Calculated	Reported
Volume	2075.5(3)	2075.5(3)
Space group	P 21/n	P 21/n
Hall group	-P 2yn	-P 2yn
Moiety formula	C23 H27 B O3	?
Sum formula	C23 H27 B O3	C23 H27 B O3
Mr	362.26	362.25
Dx,g cm-3	1.159	1.159
Z	4	4
Mu (mm-1)	0.074	0.074
F000	776.0	776.0
F000'	776.34	
h,k,lmax	17,13,23	17,13,22
Nref	5612	5596
Tmin,Tmax	0.981,0.994	0.960,0.990
Tmin'	0.964	

Correction method= # Reported T Limits: Tmin=0.960 Tmax=0.990
AbsCorr = MULTI-SCAN

Data completeness= 0.997 Theta(max)= 29.204

R(reflections)= 0.0452(4098) wR2(reflections)= 0.1209(5596)

S = 1.032 Npar= 248

The following ALERTS were generated. Each ALERT has the format
test-name_ALERT_alert-type_alert-level.
Click on the hyperlinks for more details of the test.

Alert level G

```
PLAT066_ALERT_1_G Predicted and Reported Tmin&Tmax Range Identical          ? Check
PLAT395_ALERT_2_G Deviating  X-O-Y    Angle From 120 for O2           107.1 Degree
PLAT395_ALERT_2_G Deviating  X-O-Y    Angle From 120 for O3           107.2 Degree
PLAT883_ALERT_1_G No Info/Value for _atom_sites_solution_primary .   Please Do !
PLAT912_ALERT_4_G Missing # of FCF Reflections Above STh/L=   0.600        16 Note
PLAT941_ALERT_3_G Average HKL Measurement Multiplicity ...........      4.0 Low
PLAT978_ALERT_2_G Number C-C Bonds with Positive Residual Density.       21 Info
```

0 **ALERT level A** = Most likely a serious problem - resolve or explain
0 **ALERT level B** = A potentially serious problem, consider carefully
0 **ALERT level C** = Check. Ensure it is not caused by an omission or oversight
7 **ALERT level G** = General information/check it is not something unexpected

2 ALERT type 1 CIF construction/syntax error, inconsistent or missing data
3 ALERT type 2 Indicator that the structure model may be wrong or deficient
1 ALERT type 3 Indicator that the structure quality may be low
1 ALERT type 4 Improvement, methodology, query or suggestion
0 ALERT type 5 Informative message, check

Datablock AX2220 - ellipsoid plot

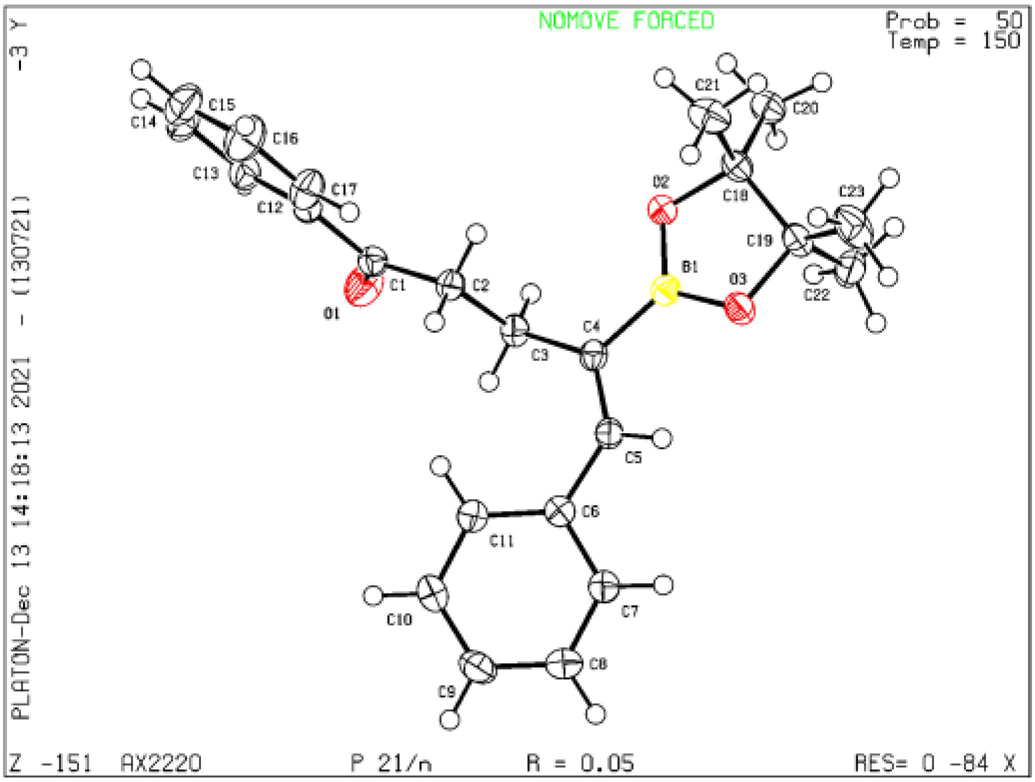

4a

checkCIF/PLATON report

Structure factors have been supplied for datablock(s) AX2232

THIS REPORT IS FOR GUIDANCE ONLY. IF USED AS PART OF A REVIEW PROCEDURE FOR PUBLICATION, IT SHOULD NOT REPLACE THE EXPERTISE OF AN EXPERIENCED CRYSTALLOGRAPHIC REFEREE.

No syntax errors found. CIF dictionary Interpreting this report

Datablock: AX2232

Bond precision:	C-C = 0.0029 A		Wavelength=0.71073
Cell:	a=12.9120(13)	b=10.1397(10)	c=15.5970(16)
	alpha=90	beta=90	gamma=90
Temperature:	150 K		

	Calculated	Reported
Volume	2042.0(4)	2042.0(4)
Space group	P n a 21	P n a 21
Hall group	P 2c -2n	P 2c -2n
Moiety formula	C23 H27 B O3	?
Sum formula	C23 H27 B O3	C23 H27 B O3
Mr	362.26	362.25
Dx,g cm-3	1.178	1.178
Z	4	4
Mu (mm-1)	0.076	0.076
F000	776.0	776.0
F000'	776.34	
h,k,lmax	17,13,21	17,13,21
Nref	5518[2854]	5505
Tmin,Tmax	0.978,0.993	0.980,0.990
Tmin'	0.978	

Correction method= # Reported T Limits: Tmin=0.980 Tmax=0.990
AbsCorr = MULTI-SCAN

Data completeness= 1.93/1.00 Theta(max)= 29.178

R(reflections)= 0.0375(4869) wR2(reflections)= 0.0945(5505)

S = 1.050 Npar= 248

The following ALERTS were generated. Each ALERT has the format
test-name_ALERT_alert-type_alert-level.
Click on the hyperlinks for more details of the test.

Alert level G

```
PLAT032_ALERT_4_G Std. Uncertainty on Flack Parameter Value High .      0.400 Report
PLAT066_ALERT_1_G Predicted and Reported Tmin&Tmax Range Identical          ? Check
PLAT395_ALERT_2_G Deviating  X-O-Y    Angle From 120 for O2             105.9 Degree
PLAT395_ALERT_2_G Deviating  X-O-Y    Angle From 120 for O3             107.0 Degree
PLAT792_ALERT_1_G Model has Chirality at C2            (Polar  SPGR)        S Verify
PLAT883_ALERT_1_G No Info/Value for _atom_sites_solution_primary .     Please Do !
PLAT978_ALERT_2_G Number C-C Bonds with Positive Residual Density.         13 Info
```

```
   0 ALERT level A - Most likely a serious problem - resolve or explain
   0 ALERT level B - A potentially serious problem, consider carefully
   0 ALERT level C - Check. Ensure it is not caused by an omission or oversight
   7 ALERT level G - General information/check it is not something unexpected

   3 ALERT type 1 CIF construction/syntax error, inconsistent or missing data
   3 ALERT type 2 Indicator that the structure model may be wrong or deficient
   0 ALERT type 3 Indicator that the structure quality may be low
   1 ALERT type 4 Improvement, methodology, query or suggestion
   0 ALERT type 5 Informative message, check
```

Datablock AX2232 - ellipsoid plot

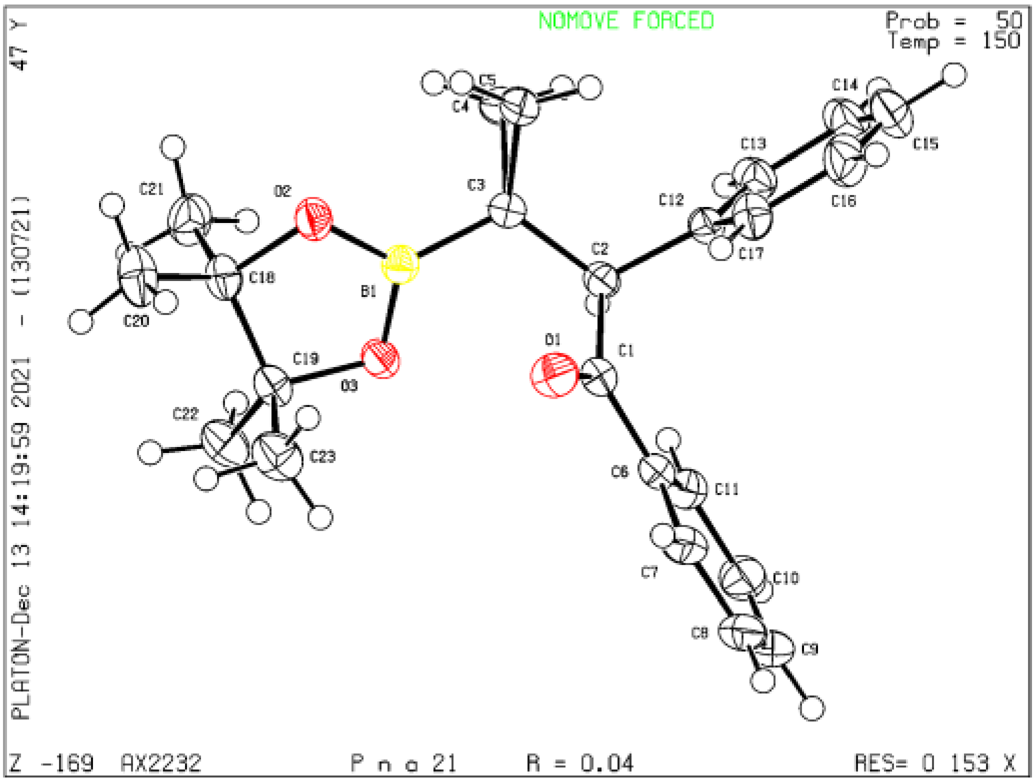

Supplement: SC-013-D2SC00840H-s001 [file SC-013-D2SC00840H-s001.pdf]
